# Direct radical functionalization of native sugars

**DOI:** 10.1038/s41586-024-07548-0

**Published:** 2024-06-19

**Authors:** Yi Jiang, Yi Wei, Qian-Yi Zhou, Guo-Quan Sun, Xia-Ping Fu, Nikita Levin, Yijun Zhang, Wen-Qiang Liu, NingXi Song, Shabaz Mohammed, Benjamin G. Davis, Ming Joo Koh

**Affiliations:** 1 Department of Chemistry, National University of Singapore, Singapore, Singapore; 2 The Rosalind Franklin Institute, Harwell Science and Innovation Campus, Didcot, UK; 3 Department of Pharmacology, University of Oxford, Oxford, UK; 4 Department of Chemistry, Southern University of Science and Technology, Shenzhen, China; 5 Department of Chemistry, University of Oxford, Oxford, UK; 6 Department of Biochemistry, University of Oxford, Oxford, UK

**Keywords:** Synthetic chemistry methodology, Carbohydrate chemistry

## Abstract

Naturally occurring (native) sugars and carbohydrates contain numerous hydroxyl groups of similar reactivity^1,2^. Chemists, therefore, rely typically on laborious, multi-step protecting-group strategies^3^ to convert these renewable feedstocks into reagents (glycosyl donors) to make glycans. The direct transformation of native sugars to complex saccharides remains a notable challenge. Here we describe a photoinduced approach to achieve site- and stereoselective chemical glycosylation from widely available native sugar building blocks, which through homolytic (one-electron) chemistry bypasses unnecessary hydroxyl group masking and manipulation. This process is reminiscent of nature in its regiocontrolled generation of a transient glycosyl donor, followed by radical-based cross-coupling with electrophiles on activation with light. Through selective anomeric functionalization of mono- and oligosaccharides, this protecting-group-free ‘cap and glycosylate’ approach offers straightforward access to a wide array of metabolically robust glycosyl compounds. Owing to its biocompatibility, the method was extended to the direct post-translational glycosylation of proteins.

## Abstract (web summary)

A radical-based method for functionalizing native sugars shows a way to remove typical protecting-group manipulations.

## Main

Widely distributed across the three domains of cellular life forms, carbohydrates play pivotal parts in many biological processes^4–7^. Nature often provides greatly altered function simply through the attachment of a glycosyl moiety. Because of their importance, substantial efforts have been devoted to accessing these saccharides and their conjugates to better understand their properties, functions and potential disease-related roles and to enable the discovery of sugar-based therapeutics^8–10^. The difficulty of extracting notable quantities of pure samples from nature has prompted chemists to secure most saccharides by synthetic means. To this end, non-enzymatic chemical glycosylation^11–15^ represents the cornerstone of carbohydrate chemistry by offering a reliable avenue to assemble a vast array of natural and non-natural glycoside entities. However, unlike enzymatic machineries that can mediate glycosylation by using unprotected polyhydroxylated glycosyl donors with excellent regiocontrol^16,17^, established chemical glycosylation methodologies are less precise and typically require cumbersome protecting-group strategies^3,11–15^ to overcome the problem of site selectivity. These complications are highlighted in the existing synthetic routes to *C*-glycosyl compounds^13^, a parallel carbohydrate class that is rarer in nature but has gained increasing prominence as robust and often more biologically potent surrogates of *O*-glycosides in developing medications to treat cancer, diabetes and other illnesses^18,19^.

**Fig. 1:**
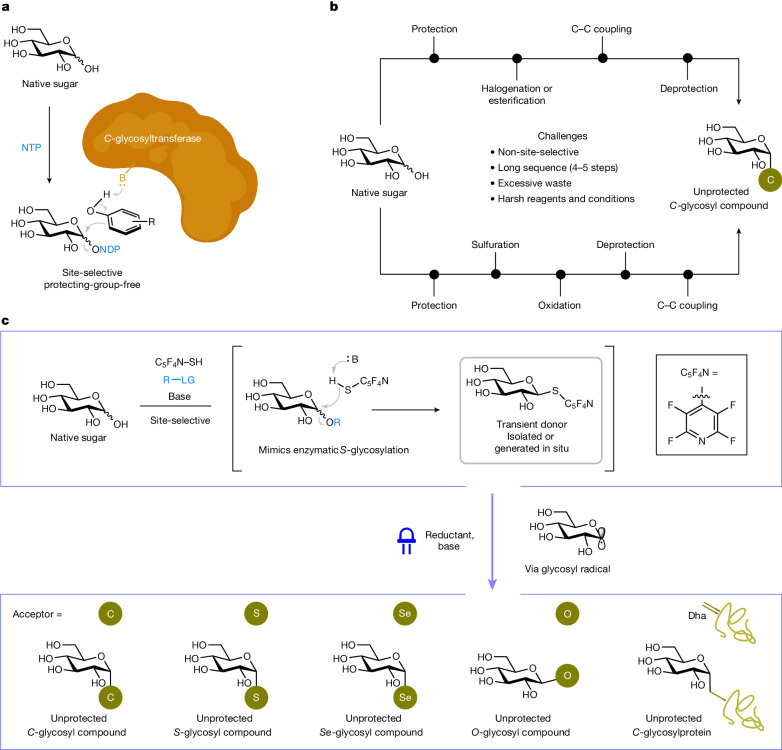
Design of a protecting-group-free ‘cap and glycosylate’ blueprint for direct functionalization of native sugars to robust glycosides. **a**, Enzymatic synthesis of *C*-glycosyl compounds. **b**, Challenges in the non-enzymatic chemical synthesis of unprotected *C*-glycosyl compounds. **c**, Our biomimetic approach to achieve site- and stereoselective anomeric functionalization of native sugars. R, functional group; LG, leaving group; B, base; NTP, nucleoside triphosphate; NDP, nucleoside diphosphate; Dha, dehydroalanine; C_5_F_4_N–SH, 2,3,5,6-tetrafluoropyridine-4-thiol; and C_5_F_4_N, 2,3,5,6-tetrafluoro-4-pyridyl.

In contrast to the highly site-selective nature of enzymatic *C*-glycosylation (Fig. 1a), the state-of-the-art advances in non-enzymatic chemical *C*-glycosylation often require multi-step reaction sequences (hydroxyl group protection, functionalization and deprotection) involving delicate control and/or harsh reaction conditions to transform fully unprotected native sugars (the most abundant form in nature) into tailored glycosyl precursors containing anomeric leaving groups such as halides^20–24^, esters^25–27^, sulfoxides^28,29^ or sulfones^30–32^, setting the stage for the ensuing carbon–carbon bond-forming reaction to deliver the desired unprotected *C*-glycosyl compound only after eventual deprotection (Fig. 1b). The practical drawbacks and inefficiencies of these approaches consequently limit their use in synthetic glycochemistry and prevent further applications under intricate biological conditions. Thus, enacting a regime that allows direct coupling of native sugars for broad-scope glycosylation^33^ to access stereoisomerically pure *C*-glycosyl compounds and other hydrolytically stable and medicinally important variants (such as *S*- and *Se*-glycosides)^34–36^ as well as *C*-linked glycoproteins is a longstanding goal in glycoscience research. However, this has remained unknown owing to numerous challenges associated with efficiency, selectivity and biocompatibility.

Inspired by reports of biological *S*-glycosylation in which *S*-glycosyltranferases mediate the formation of stable *S*-glycosidic linkages using unprotected nucleotide sugars generated from their native variants by regioselective anomeric phosphorylation^37,38^, we reasoned that a biomimetic approach could be adopted to preferentially activate and substitute the anomeric hydroxyl group (hemiacetal) in a native sugar in its cyclic form (capping). This would afford a thioglycoside intermediate that, under suitable conditions, could undergo stereocontrolled desulfurative cross-coupling^39–41^ with an appropriate reagent in a single operation (glycosylation). Just as in nature, the activated glycosyl donor that was temporarily generated remains traceless. However, several challenges have to be addressed for the success of this ‘cap and glycosylate’ strategy. First, the multiple hydroxyl groups must be distinguished to ensure selective masking of the hemiacetal to form a transient thioglycosyl donor. Second, the donor must be sufficiently reactive to participate in cross-coupling without competitive interference or reaction on other hydroxyl sites, which would otherwise result in undesired reactions and intractable mixtures. Added to these is the challenge of controlling the stereochemical outcome of anomeric functionalization in the context of a complex, polyhydroxylated carbohydrate residue.

Here we report a metal- and protecting-group-free blueprint that enables the direct anomeric functionalization of unprotected monosaccharides and oligosaccharides in their native forms by radical-based cross-coupling with various electrophiles under mild photoirradiation conditions (Fig. 1c). This ‘cap and glycosylate’ approach eliminates the need for pre-installation and removal of protecting groups, solving an enduring problem in the field and providing a general platform to accelerate the preparation of robust carbo-, thio- and selenoglycosyl compounds as well as *O*-glycosides in high regio- and stereoselectivity. We also show that the protocol is amenable to the direct chemical synthesis of unprotected *C*-glycosylproteins in a post-translational manner that is complementary to the analogous biological *O*- and *N*-glycosylation processes.

**Fig. 2:**
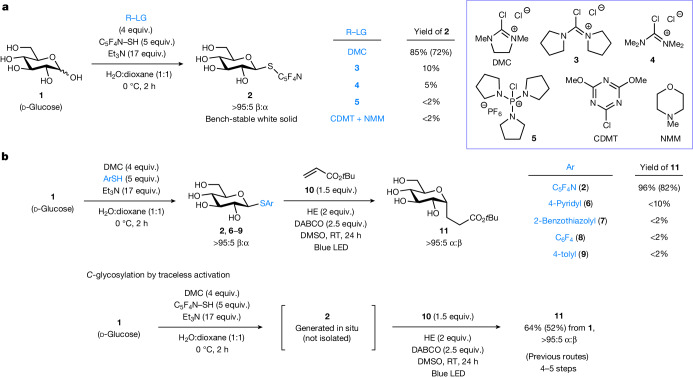
Reaction development. **a**, Selection of an appropriate activator for site-selective nucleophilic substitution. **b**, Identification of the most effective thioglycosyl donor for photoinduced cross-coupling. Yields were determined by ^1^H NMR analysis of the crude reaction mixture; yields in parentheses denote isolated yields. α:β Anomeric ratios were determined by ^1^H NMR and liquid chromatography–mass spectrometry (LC-MS) analysis. DMC, 2-chloro-1,3-dimethylimidazolinium chloride; CDMT, 2-chloro-4,6-dimethoxy-1,3,5-triazine; NMM, *N*-methylmorpholine; HE, Hantzsch ester (diethyl 1,4-dihydro-2,6-dimethyl-3,5-pyridinedicarboxylate); LED, light-emitting diode; RT, room temperature; and C_6_F_4_, 2,3,5,6-tetrafluorophenyl.

**Fig. 3:**
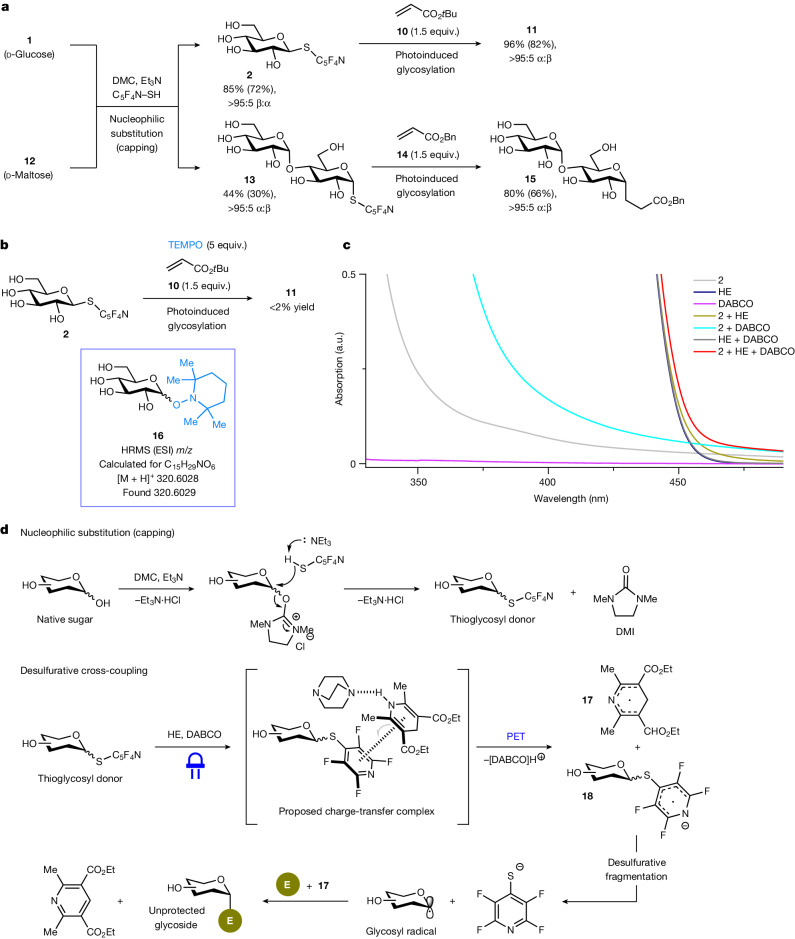
Mechanistic studies. **a**, Different anomers of the thioglycoside intermediate eventually converge to a stereoisomerically pure *C*-glycosyl product. **b**, Radical trap experiment supports the intermediacy of a glycosyl radical species. **c**, UV–vis absorption spectra of reaction components in DMSO. **d**, Plausible mechanisms for native sugar activation and photoinduced cross-coupling. Yields were determined by ^1^H NMR analysis of the crude reaction mixture; yields in parentheses denote isolated yields. α:β Anomeric ratios were determined by ^1^H NMR and LC-MS analysis. ESI, electrospray ionization; E, electrophile.

Considering the susceptibility of certain transition metals to inhibition by polar hydroxyl groups^42^, we sought to engineer a metal-free protocol that harnesses the reactivity of electron-deficient alkyl sulfides to undergo desulfurative transformations on photoactivation^39–41^. We first evaluated reaction parameters that promote regioselective nucleophilic substitution (capping) using d-glucose **1** as the model substrate (Supplementary Table 3). Taking advantage of the greater acidity of the anomeric OH with respect to other hydroxyl units^43^, various activating agents (R−LG) were examined to convert **1** to its bench-stable 2,3,5,6-tetrafluoropyridine-4-thioglycoside derivative **2** under weakly basic conditions (Fig. 2a). In the presence of commercially available 2-chloro-1,3-dimethylimidazolinium chloride (DMC) as activator and triethylamine as base, **2** was obtained in 85% yield (72% isolated yield) and more than 95:5 β:α ratio at 0 °C within 2 h. In our hands, **2** (white solid) could be stored in air on the bench over months without noticeable decomposition. It is worth noting that the C1 stereochemistry of this *S*-glycosyl donor is inconsequential as it will be transformed into a glycosyl radical species during the course of C−C bond formation in the subsequent step (Fig. 3a). Other analogues of DMC (**3** and **4**) led to markedly diminished yields, whereas other commonly used reagents such as chlorophosphonium salt **5** and a combination of 2-chloro-4,6-dimethoxy-1,3,5-triazine (CDMT) and *N*-methylmorpholine (NMM) failed to promote the reaction.

With DMC identified as the most effective activator, we used the nucleophilic substitution conditions to synthesize not only **2** but also a range of unprotected (hetero)aryl thioglucosides (**6**−**9**) for comparison. To drive glycosylation, we subjected the thioglucosides to a reaction with acrylate **10** under visible light illumination^30^. After an extensive survey of conditions (Supplementary Table 4), we discovered that **2** underwent desulfurative C−C coupling to deliver unprotected *C*-alkyl glucoside **11** in 96% yield (82% isolated yield) and more than 95% α selectivity using a combination of Hantzsch ester as reductant, 1,4-diazabicyclo[2.2.2]octane (DABCO) and dimethyl sulfoxide (DMSO) as solvent under blue LED irradiation at ambient temperature (Fig. 2b). To our knowledge, this reaction represents the first successful use of 2,3,5,6-tetrafluoropyridine-4-thioglycoside as a new class of glycosyl donor in chemical glycosylation.

By contrast, poor conversion was observed with the less redox-active *S*-glucosides derived from other less electron-withdrawing (hetero)aryl thiols (**6**−**9**), highlighting the importance of the fluorinated heteroaromatic moiety for photoinduced cross-coupling^39–41^. This was supported by cyclic voltammetry studies showing that **2** has the least negative reduction potential (Supplementary Figs. 2–6), which is comparable to that of a redox-active heteroaryl glycosyl sulfone^30^. By contrast, excluding the light source, Hantzsch ester or DABCO was detrimental to the reaction, and changing the base or solvent led to lower yields. To demonstrate the power of the ‘cap and glycosylate’ approach by traceless activation, we showed that α-**11** could be generated from **1** in a single sequence without the need for isolating the *S*-glycosyl intermediate **2**. The overall step efficiency and yield (64% yield and 52% isolated yield) offer marked advantages over previous chemical *C*-glycosylation approaches that require multiple steps (Fig. 1b).

**Fig. 4:**
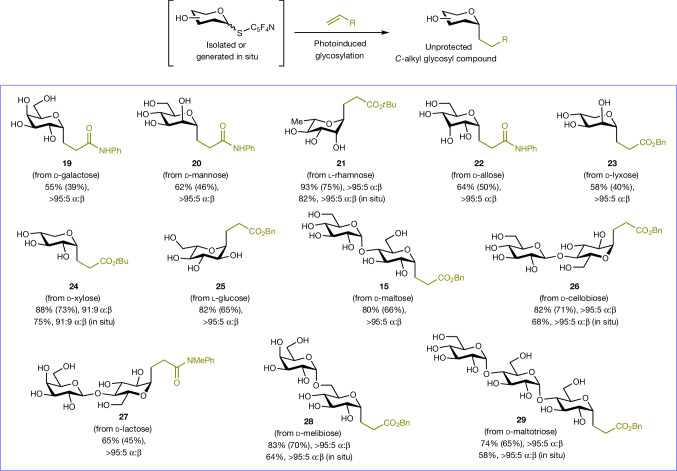
Scope of the reaction with various native sugars. Cross-coupling of mono- and oligosaccharides through unprotected glycosyl donors to directly afford unprotected *C*-alkyl glycosyl compounds. Yields were determined by ^1^H NMR analysis of the crude reaction mixture; yields in parentheses denote isolated yields. α:β Anomeric ratios were determined by ^1^H NMR and LC-MS analysis. Bn, benzyl.

Experiments were conducted to gain insight into the individual processes for native sugar activation and cross-coupling. As shown in Fig. 2a, nucleophilic substitution of d-glucose **1** afforded 2,3,5,6-tetrafluoropyridine-4-thioglycoside **2** in 85% yield (72% isolated yield) and more than 95:5 β:α ratio. On the contrary, we found that the corresponding thioglycoside **13** was secured in 44% yield (30% isolated yield) and more than 95:5 α:β ratio from d-maltose **12** under the same established conditions (Fig. 3a). In solution, the α and β anomers of native sugars (**1,** **12**) can interconvert and exist in equilibrium; each anomer individually reacts with DMC before undergoing stereoinvertive nucleophilic displacement^43^ by the thiol (Supplementary Fig. 7). Alternatively, the 2-OH group of the DMC-activated β anomeric intermediate may engage in neighbouring group participation by an intramolecular nucleophilic attack to generate a 1,2-anhydro species^43^, which is susceptible to site-selective ring cleavage by the thiol nucleophile. This pathway is probably insignificant in the reaction leading to **13**, given that β-**13** was detected in minor amounts. For other saccharides (Fig. 4), the various pathways for nucleophilic substitution may be favoured to different extents in the reaction system^43^. The structure of the 2,3,5,6-tetrafluoropyridine-4-thioglycoside derived from d-mannose was confirmed by X-ray crystallographic analysis (Supplementary Information section 7).

Subjecting **2** and **13** separately to standard cross-coupling conditions with an acrylate gave **11** and **15**, respectively, both of which possess the same sense of anomeric selectivity (Fig. 3a). This notably implies that, unlike heterolytic glycosylations, the C1 stereochemistry of the *S*-glycosyl donor is inconsequential, highlighting the distinct advantage of the present strategy in transforming mixtures of unprotected native sugar isomers, through their thioglycoside derivatives, into stereoisomerically pure glycosides in a streamlined fashion. In a separate study, the addition of exogenous (2,2,6,6-tetramethylpiperidin-1-yl)oxyl (TEMPO) inhibited the photoinduced transformation of **2** to **11** (Fig. 3b). High-resolution mass spectrometry (HRMS) analysis revealed the formation of a complex that can be ascribed to a TEMPO-glycoside adduct **16**, providing evidence that a sufficiently long-lived glycosyl (anomeric) radical species is generated during the process. These processes are in contrast to heterolytic glycosylations that essentially lack the formation of a clear intermediate species (for example, glycosyl cation).

We further explored the nature of the photoinduced reaction (using **2** as the model substrate) through ultraviolet–visible absorption (UV–vis) spectroscopy (Fig. 3c). Independent absorption spectra of **2** and DABCO revealed bands largely in the UV region, and a mixture of these two components led only to a small redshift that extends into the visible region (>400 nm). By contrast, a DMSO solution of Hantzsch ester exhibited strong absorption in the visible region, but no noticeable changes were observed with a mixture of Hantzsch ester and DABCO. A mixture of **2** and Hantzsch ester showed a slight bathochromic shift, which was amplified when **2**, Hantzsch ester and DABCO were combined together in solution. These results indicate the generation of a putative ternary complex^44,45^ between **2**, Hantzsch ester and DABCO, which is proposed to absorb visible light and undergo fragmentation to the glycosyl radical.

The studies presented here support a mechanism as shown in Fig. 3d. Site-selective capping of the more acidic anomeric hydroxyl motif by DMC forms an activated leaving group that undergoes facile nucleophilic attack by 2,3,5,6-tetrafluoropyridine-4-thiol under basic conditions, driven by concomitant generation of 1,3-dimethylimidazolidin-2-one (DMI) as a by-product^43^. Formation of a 1,2-anhydro species before nucleophilic substitution could also occur and cannot be completely ruled out (Supplementary Fig. 7). The resulting thioglycoside intermediate is postulated to associate with Hantzsch ester and DABCO in solution, affording a ternary complex that can absorb visible light to trigger photoinduced electron transfer (PET)^46^. Consistent with previously documented reactions^39–41^, the highly electrophilic nature of the fluorinated heteroaryl motif renders the thioglycoside sufficiently redox-active for PET. This delivers dihydropyridine radical **17** and a radical anion **18**, which is prone to desulfurative fragmentation to give a glycosyl radical species and 2,3,5,6-tetrafluoropyridine-4-thiolate (the conjugate acid was detected in the reaction mixture). Subsequent reaction of the glycosyl radical with an electrophilic cross-coupling partner, facilitated by **17**, proceeds in a stereoselective manner under kinetic control^30,47,48^ to give the desired unprotected glycoside.

The generality of our protecting-group-free protocol was highlighted by the wide spectrum of native mono- and oligosaccharides that could be reliably transformed into fully unprotected *C*-alky glycosyl compounds (Fig. 4) through their 2,3,5,6-tetrafluoropyridine-4-thioglycoside precursors, which were either isolated or generated in situ and used (without purification) for cross-coupling. Representative examples include pyranoside products constructed from biomass-derived monosaccharides (**19**−**21**, **24**), rare sugars (**22**, **23**) and non-natural l-glucose (**25**). More complex glycans from natural sources also served as effective substrates to deliver the corresponding *C*-alkyl glycosyl compounds (**15**, **26**−**29**) in good efficiency. Across the board, good to excellent stereocontrol was observed.

**Fig. 5:**
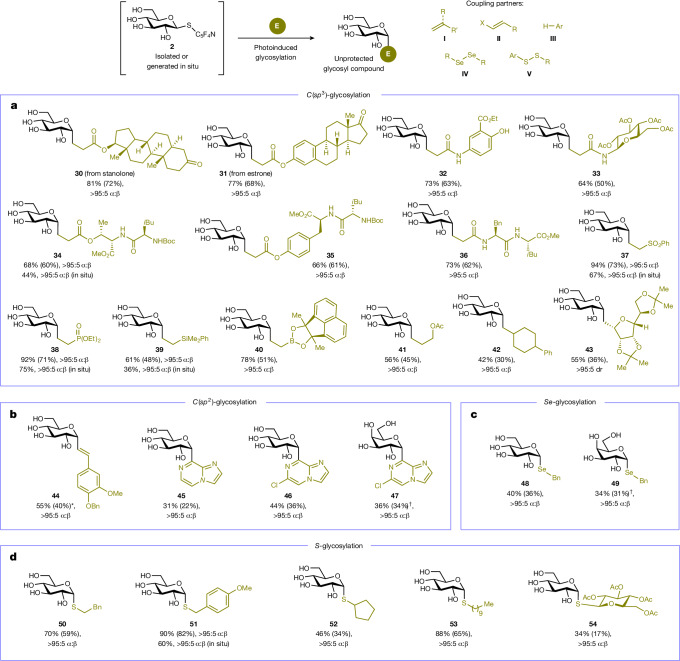
Synthesis of diverse classes of robust glycosides and glycoconjugates. **a,** *C*-Alkyl glycosyl compounds by reaction with **I**. **b,** *C*-Alkenyl and *C*-heteroaryl glycosyl compounds by reaction with **II** (for **44**) and **III** (for **45**–**47**). **c,** *Se*-Glycosides by reaction with **IV**. **d,** *S*-Glycosides by reaction with **V**. Yields were determined by ^1^H NMR analysis of the crude reaction mixture; yields in parentheses denote isolated yields. α:β Anomeric ratios, diastereomeric ratios (dr) and *E*:*Z* ratios were determined by ^1^H NMR and LC-MS analysis. The asterisk indicates the value obtained as a 77:23 *E*:*Z* mixture. The dagger indicates d-galactose was used. Ar, aryl; X, halide; Ac, acetyl; Boc, *tert*-butyloxycarbonyl.

Besides α,β-unsaturated carbonyl compounds, other alkenes were investigated as cross-coupling partners (Fig. 5a). Densely functionalized acrylates and acrylamides conjugated to biologically active compounds (**30,** **31**), an aminosalicylate (**32**), an amino sugar (**33**) and oligopeptides (**34**−**36**) were compatible substrates, providing access to highly polar *C*-glycosylated conjugates bearing multiple acidic and basic sites. This offers a straightforward way to glycosylate complex molecules with native sugars for various applications, including the design of sugar-based peptidomimetics^23,28^. Other Michael acceptors such as vinyl sulfone (**37**), vinylphosphonate (**38**) and vinylboronate (**40**) as well as less electrophilic vinyl silane (**39**) and allyl acetate (**41**) also underwent efficient reaction to furnish the desired *C*-alkyl glycosyl adducts bearing functional groups that could serve as useful synthetic handles for further manipulations. Of particular note, cross-coupling was found to proceed even in the presence of a less-activated alkyl-substituted alkene (**42**). Metabolically stable pseudo-oligosaccharide^49^ building blocks such as *C*-glycosidic disaccharide **43** featuring two newly formed stereocentres could be expeditiously assembled with complete stereocontrol through reaction with an *exo*-glucal as radical acceptor.

To showcase the versatility of the ‘cap and glycosylate’ approach in securing other categories of unprotected saccharides, we replaced the alkene coupling partner with other electrophilic reagents that could participate as radical acceptors. Using a haloalkene reagent (Fig. 5b), a *C*-alkenyl glycosyl compound (**44**) was successfully secured in high anomeric selectivity; this process is postulated to proceed through a glycosyl radical addition–reduction–β halide elimination pathway^24^. *C*-Heteroaryl glycosylation could also be realized by direct coupling with heteroarenes under acid-free conditions, delivering unprotected **45**−**47** selectively at the most electron-deficient sites, which is congruent with a previous report involving fully protected glycosyl radicals^50^. Our heteroarylation approach is complementary to a previously reported metallaphotoredox-enabled deoxygenative strategy (incompatible with native sugars) that involved pre-activation of an exposed anomeric hydroxyl followed by cross-coupling with a heteroaryl halide^51^.

**Extended Data Fig. 1:**
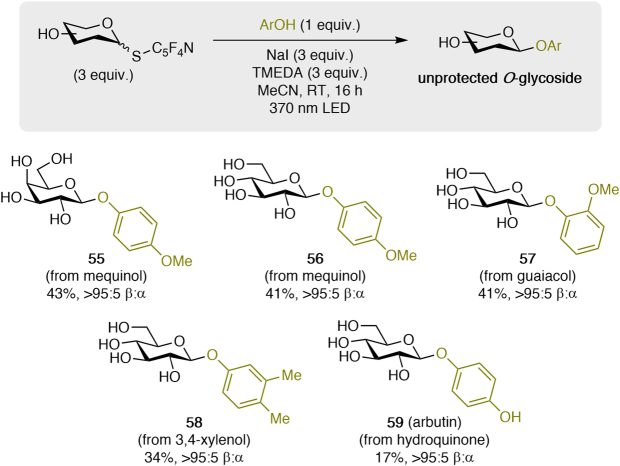
Preliminary results in photoinduced *O*-glycosylation. Reaction scope using different sugars and phenols. Yields denote isolated yields. β:α Anomeric ratios were determined by ^1^H NMR and LC-MS analysis. TMEDA, tetramethylethylenediamine.

Beyond *C*-glycosylation, we extended the protecting-group-free reaction manifold to the preparation of other glycomimetics such as selenoglycosides (Fig. 5c) and thioglycosides (Fig. 5d). Along with *C*-glycosyl compounds, these entities have found many applications as robust substitutes of the naturally occurring *O*-saccharides, thus efficient ways to synthesize them in high stereochemical purity are highly desirable. Both unprotected *Se*-glycosides (**48,** **49**) and *S*-glycosides (**50**−**54**) were accessible through reaction with diselenide or disulfide reagents^52^, respectively, comparing favourably with previous protocols that relied on laborious preparation of glycosyl precursors. It is to be noted that **50**−**54** were exclusively isolated as α anomers (compared with β anomers from nucleophilic substitution in Fig. 2). Similar to the *C*-glycosyl cases in Figs. 2 and 4, the observed stereochemical outcome for **48**−**54** could be rationalized by the stabilizing orbital interaction between the nonbonding electron pair of the ring oxygen and the σ* of the incipient bond at the anomeric carbon in the transition state, as the glycosyl radical reacts with the electrophile^47,48^ (Fig. 3d). *O*-glycosylation^53^ with phenols could also be achieved by tuning the photoinduced cross-coupling conditions using iodide as reductant^40,45^ (Extended Data Fig. 1).

Encouraged by our successful efforts in small-molecule glycosyl compound synthesis, we attempted to test our ‘cap and glycosylate’ protocol in the synthesis of glycoproteins, which are known to mediate numerous essential biological processes. In nature, glycoproteins are typically formed by linking sugar units to *O*- or *N*-containing side chains of amino acid residues serine, threonine or asparagine using glycosyltransferases, such as the attachment of *O*-linked-β-d-*N*-acetylglucosamine (*O*-GlcNAc) to serine or threonine residues by *O*-GlcNAc transferase. However, this glycosylation can be reversed by intracellular glycosidases, and such a write-and-erase dynamic process makes it challenging to probe the biological functions of glycoproteins. In this context, chemical approaches to generate non-cleavable glycoproteins (such as *C*-glycosylproteins) offer alternative and promising strategies for systematically investigating glycoprotein functions. Nevertheless, post-translational chemical glycosylation of proteins, particularly the attachment of sugar units to proteins by direct anomeric functionalization, is largely unexplored in synthetic carbohydrate and protein chemistry^54^. This may be ascribed to the lack of suitable unprotected glycosyl precursors that are stable yet sufficiently reactive, as well as the stringent requirements for biocompatible conditions, including water compatibility (which quenches heterolytic chemical glycosyl donors), ability to remain non-destructive to biological substrates and low reactivity towards the biogenic functional groups that are present in most biological environments^55^. Owing to the insolubility of Hantzsch ester in the necessary aqueous medium, photoinduced cross-coupling conditions were instead based on the formation of charge-transfer complexes between 2,3,5,6-tetrafluoropyridine-4-thioglycoside and bis(catecholato)diboron (B_2_Cat_2_) as reductant^41^ (Supplementary Tables 6–8).

**Fig. 6:**
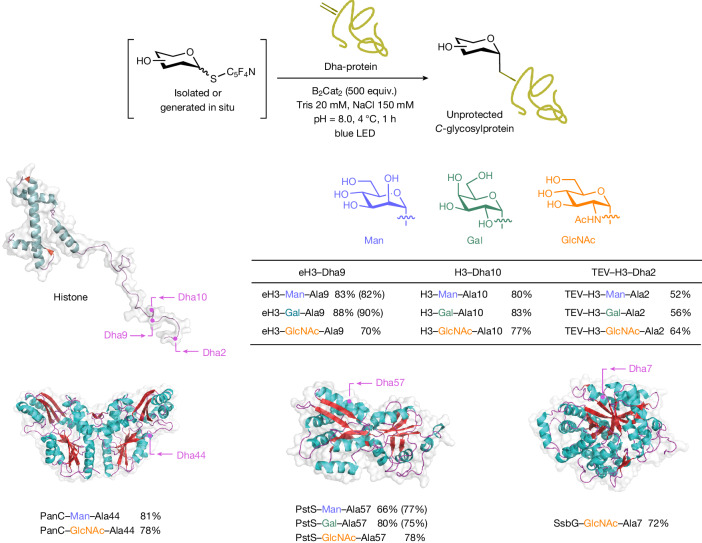
Application to direct post-translational chemical glycosylation of proteins. Glycosylation of proteins by cross-coupling of representative native sugars (through capping as thioglycosyl donors) to afford unprotected *C*-alkyl glycosylproteins. Yields were determined by LC-MS analysis based on conversion of the protein substrate; yields in parentheses denote reactions with in situ-generated and unpurified thioglycosyl donors. Tris, 2-amino-2-(hydroxylmethyl)-propane-1,3-diol; B_2_Cat_2_, bis(catecholato)diboron; Man, d-mannosyl; Gal, d-galactosyl; GlcNAc, *N*-acetyl-d-glucosaminyl.

After identifying the optimal conditions (500 equiv. of B_2_Cat_2_, 4 °C, 1 h, pH 8.0 in Tris buffer) as shown in Fig. 6, three mammalian glycoprotein sugars (d-mannose, d-galactose and *N*-acetylglucosamine) were selected to react with dehydroalanine (Dha)-tagged proteins^56,57^ with varying architectures and functions, including histone H3 (a small α-helical nuclear protein), PanC (*Mycobacterium tuberculosis* pantothenate synthetase enzyme)^58^, PstS (a bacterial phosphate transport protein)^59^ and SsβG (an αβ_8_ TIM (triose-phosphate isomerase) barrel enzyme)^60^. In the event, all the examined proteins were found to be competent glycosyl radical acceptors under the established conditions, with the desired *C*-alkyl glycosylproteins secured in good to excellent yields across the board regardless of their size and fold. The stereochemistry of the newly formed C−C bond at the anomeric carbon (α selectivity) is presumed to be identical to that of small-molecule glycosylation (Fig. 4). Notably, histone H3–GlcNAc–Ala10 generates a non-cleavable mimetic of the reported epigenetic mark GlcNAc–Ser10 (ref. ^61^); access to this glycoprotein conjugate may shed light on the poorly understood biological role of this post-translational modification process. Similar efficiencies were also observed in the reactions of different thioglycosyl donors with each given protein (about 85% conversion for eH3–Dha9, about 80% conversion for H3–Dha10, about 55% conversion for TEV H3–Dha2, about 80% conversion for PanC–Dha44 and about 70% conversion for PstS-Dha57). About 5% of a minor product featuring two units of GlcNAc addition was detected for PstS–GlcNAc–Ala57, which we ascribe to non-specific glycosylation of lysine residues^62^ (Supplementary Table 9 and Supplementary Fig. 15). Similar to the examples in Figs. 2b, 4 and 5, traceless activation by in situ formation of the *S*-glycosyl intermediates could be implemented without compromising on protein glycosylation efficiency, thereby exemplifying the power of our protecting-group-free ‘cap and glycosylate’ approach allowing native sugars to be directly used for glycosylating proteins post-translationally.

## Online content

Any methods, additional references, Nature Portfolio reporting summaries, source data, extended data, supplementary information, acknowledgements, peer review information; details of author contributions and competing interests; and statements of data and code availability are available at 10.1038/s41586-024-07548-0.

### Supplementary information

Supplementary Information This file contains the following 11 sections: (1) General information; (2) Preparation of substrates; (3) Analytical data of substrates; (4) Optimization studies and experimental procedures; (5) Analytical data of products; (6) Mechanistic studies; (7) X-ray crystallographic data; (8) Glycosylation of proteins; (9) Preliminary results for photoinduced *O*-glycosylation; (10) References; and (11) NMR spectra.

## Data Availability

The crystallographic data are available free of charge from the Cambridge Crystallographic Data Centre under reference no. CCDC-2263895 (**S2**). MS/MS raw data files have been uploaded to the PRIDE repository under reference no. PXD046389. All other data are available in the main text or the Supplementary Information.
