# Supplementary material for: Direct radical functionalization of native sugars
Source: Nature. 2024 Jun 19;631(8020):319–27. doi: 10.1038/s41586-024-07548-0 (PMC11236704; doi:10.1038/s41586-024-07548-0)

nature portfolio

https://doi.org/10.1038/s41586-024-07548-0

Supplementary information

Direct radical functionalization of native sugars

In the format provided by the authors and unedited

Supplementary Information

Direct radical functionalization of native sugars

Yi Jiang†, Yi Wei†, Qian-Yi Zhou, Guo-Quan Sun, Xia-Ping Fu, Nikita Levin, Yijun Zhang, Wen-Qiang Liu, NingXi Song, Shabaz Mohammed, Benjamin G. Davis*, Ming Joo Koh*

*Corresponding authors: Ming Joo Koh, chmkmj@nus.edu.sg; Benjamin G. Davis, ben.davis@chem.ox.ac.uk

Table of Contents

1. General information S1

2. Preparation of substrates S2

2.1. Preparation of DMC and its analogues S2

2.2. Preparation of *S*-glycosyl donors S3

2.3. Preparation of glycosyl radical acceptors S5

3. Analytical data of substrates S6

4. Optimization studies and experimental procedures S30

4.1. Photoinduced reaction set up S30

4.2. Optimization for anomeric functionalization of native sugars S31

4.3. General procedure for photoinduced glycosylation S32

4.4. General procedure for photoinduced glycosylation (traceless) S33

5. Analytical data of products S34

6. Mechanistic studies S62

6.1. Glycosyl radical trap experiment S62

6.2. UV/vis spectroscopy studies S63

6.3. Cyclic voltammetry studies S64

6.4. Proposed mechanism S67

7. X-ray crystallographic data S68

8. Glycosylation of proteins S70

8.1. Preparation of proteins **S70**

8.2. Optimization of protein glycosylation conditions **S73**

8.3. General procedure for photoinduced glycosylation of proteins **S74**

8.4. Glycoproteins mass spectrometry analysis **S75**

8.5. LC-MS/MS analysis of glycoproteins **S84**

9. Preliminary results for photoinduced *O*-glycosylation **S90**

9.1. Optimization of *O*-glycosylation reaction conditions **S90**

9.2. Analytic data of *O*-glycosides **S91**

10. References **S94**

11. NMR spectra **S96**

1. General information

All commercial reagents were used without additional purification, unless otherwise stated. Anhydrous solvents were purchased from commercial suppliers and transferred under an argon atmosphere. NMR spectra were recorded on Bruker 400 MHz and Bruker DPX 500 MHz spectrometer. Chemical shifts are reported in ppm from tetramethylsilane with the solvent resonance resulting from incomplete deuterium incorporation as the internal standard ($CDCl_3$: δ 7.26 ppm, Methanol-d_4: 3.31 ppm, D_2O: 4.79 ppm). Data is reported as follows: chemical shift, integration, multiplicity (s = singlet, d = doublet, t = triplet, q = quartet, br = broad, m = multiplet, dt = doublet of triplet, dd = double doublet, ddd = doublet of a double doublet), and coupling constants (Hz). ^{13}C NMR spectra were recorded on Bruker 400 MHz and Bruker DPX 500 MHz spectrometer with complete proton decoupling. Chemical shifts are reported in ppm from tetramethylsilane with the solvent resonance as the internal standard ($CDCl_3$: δ 77.20 ppm, Methanol-d_4: 49.00 ppm). ^{19}F NMR spectra were recorded on Bruker 400 MHz and Bruker DPX 500 MHz spectrometer with complete proton decoupling or proton coupling. LC-MS analysis was performed on Shimadzu LCMS-2020. Further purification was performed on Shimadzu LC-20AP. High-resolution mass spectrometric data (HRMS) was obtained using Agilent 7200 Q-TOF and Bruker MicroTOF-Qll (APCI, or Electrospray ionization, ESI). UV/vis absorption spectroscopic studies were performed on JASCO V-570 spectrophotometer. Cyclic voltammetry studies were performed on Autolab PGSTAT302N. Protein glycosylation reactions were performed on the Zinsser Analytic off-deck irradiation system with reaction positions irradiated by Lumidox II 96-LED arrays. Intact protein samples were analyzed on Waters Xevo G2-XS QTof mass spectrometers equipped with a Waters Acquity UPLC. Separation was achieved using a Thermo Scientific ProSwift RP-2H monolithic column (4.6 mm × 50 mm). UltiMate 3000 nanoUHPLC system (Thermo Fisher Scientific) coupled to an Orbitrap QExactive (Thermo Fisher Scientific) was used for LC-MS/MS studies. Values for α/β, *d.r.* and crude yields of products were determined by ^{1}H NMR, ^{19}F NMR and LC-MS analysis.

Solvents

N,*N*-Dimethylacetamide (anhydrous), *N*,*N*-dimethylformamide (anhydrous) and dimethyl sulfoxide (anhydrous) were purchased from Sigma-Aldrich or Acros and used as received. All purification procedures of products were carried out with reagent grade solvents.

Materials

Unless otherwise noted, sugars and other commercial reagents were purchased from Sigma-Aldrich, Alfa Aesar, BLDpharm or other commercial suppliers and were used as received.

2. Preparation of substrates

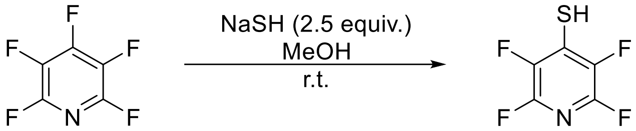

According to a known procedure[1], an open flask containing a solution of sodium hydrosulfide hydrate (2.5 equiv., 730 mmol, 54.1 g) in methanol (100 mL) was immersed into a room temperature water bath. Pentafluoropyridine (1.0 equiv., 292 mmol, 49.4 g) was added dropwise, maintaining the reaction temperature below 30 °C. The resulting cloudy viscous solution was stirred for 20 minutes at room temperature and volatile components were evaporated under reduced pressure. The solid residue was carefully quenched with concentrated hydrochloric acid (80 mL) (***Caution: H_2S evolution***). The product was extracted with petroleum ether (1×70 mL and 2×35 mL). The combined organic phases were evaporated under ambient pressure, and the residue was distilled under vacuum collecting the fraction boiling at 70-72 °C (56 mbar) to afford 51.5 g (96%) of 2,3,5,6-tetraflupropyridine-4-thiol as colorless fluid liquid, which solidifies at room temperature. Colorless crystals, melting point: 27-29 °C.

2.1. Preparation of DMC and its analogues

General procedure A: preparation of 2-chloro-1,3-dimethylimidazolinium chloride (DMC) and its analogues.

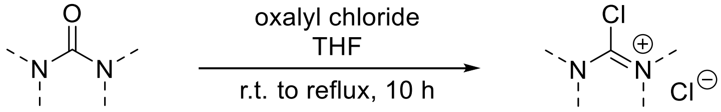

According to a known procedure[2], under argon atmosphere, to a solution of the urea derivative (1.0 equiv., 0.1 mol) in anhydrous THF (50 mL) was added oxalyl chloride (1.0 equiv., 0.1 mmol) at room temperature during a time of 10 minutes. Then, the reaction mixture was stirred under reflux for 10 hours. After cooling to room temperature, the precipitate was filtered and washed with diethyl ether and dried *in vacuo*. (*Note: quick action is needed because the products are hygroscopic*.)

2.2. Preparation of *S*-glycosyl donors

Table S1. Scope of *S*-glycosyl donors

D-Glucose

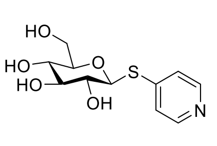

6, (58%) yield
α:β < 5:95

D-Glucose

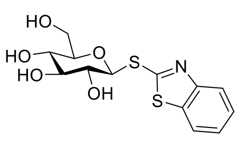

7, (47%) yield
α:β < 5:95

D-Glucose

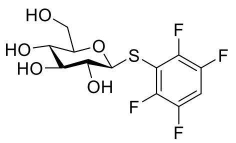

8, (66%) yield
α:β < 5:95

D-Glucose

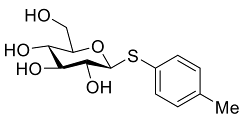

9, (71%) yield
α:β < 5:95

D-Glucose

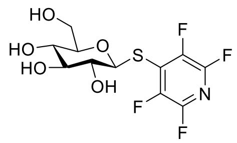

2, 85% (72%) yield
α:β < 5:95
3 mmol scale: 65% (54%) yield
α:β = 12:88

D-Galactose

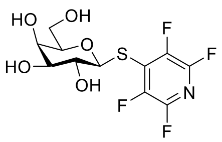

S1, 25% (16%) yield
α:β = 10:90

D-Mannose

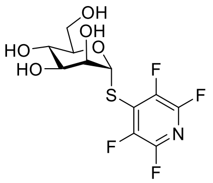

S2, 68% (54%) yield
α:β > 95:5

L-Rhamnose

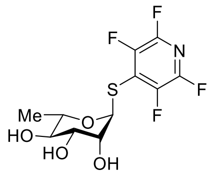

S3, 68% (51%) yield
α:β > 95:5

D-Allose

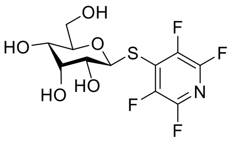

S4, 55% (40%) yield
α:β = 9:91

D-Lyxose

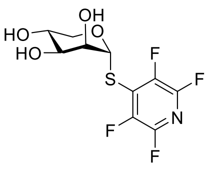

S5, 83% (66%) yield
α:β > 95:5

D-Xylose

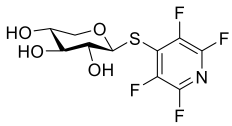

S6, 83% (62%) yield
α:β = 9:91

L-Glucose

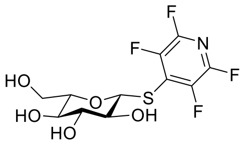

S7, 74% (59%) yield
α:β = 5:95

Maltose

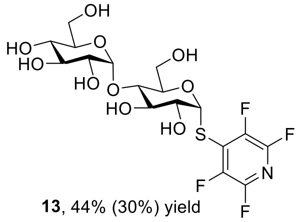

13, 44% (30%) yield
α:β > 95:5

Cellobiose

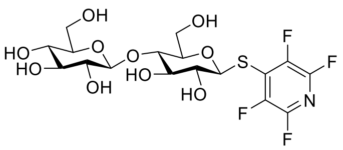

S8, 58% (49%) yield
α:β < 5:95

Lactose

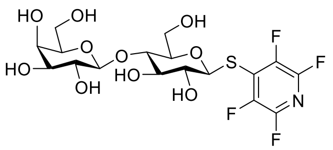

S9, 53% (41%) yield
α:β < 5:95

Melibiose

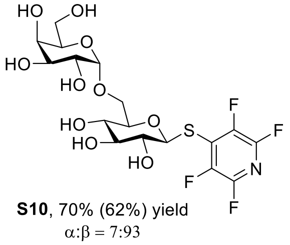

S10, 70% (62%) yield
α:β = 7:93

Maltotriose

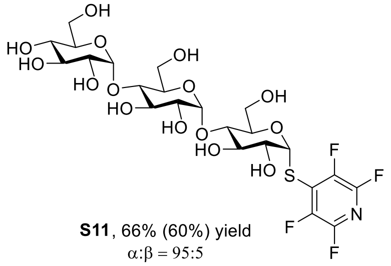

S11, 66% (60%) yield
α:β = 95:5

GlcNAc

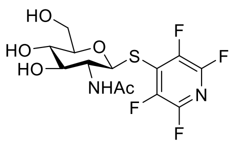

S35, 88% (75%) yield
α:β < 5:95

α:β ratio was determined by ^{1}H NMR and LC-MS analysis, yield was determined by ^{19}F-NMR analysis, isolated yield in parentheses

General procedure B: synthesis of *S*-glycosyl donors **6**-**9**.

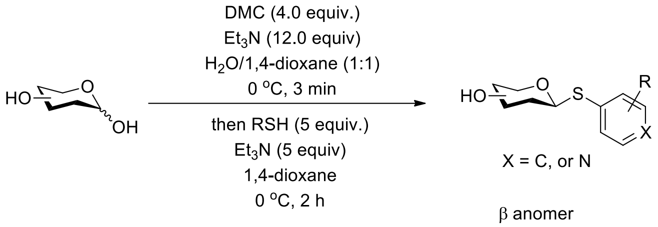

Under air, a 4 mL vial was charged with a magnetic stirring bar, sugar (1.0 equiv., 0.2 mmol), H_2O (0.4 mL), 1,4-dioxane (0.4 mL) and Et_3N (12.0 equiv. 2.4 mmol). The vial was transferred to an ice water bath and cooled to 0 °C over 3 minutes with stirring. Then, DMC (4.0 equiv., 0.8 mmol) in H_2O (0.2 mL) was added dropwise to the vial and the mixture was stirred for 3 minutes. After that, a solution of the corresponding (hetero)aryl thiol (5.0 equiv., 1.0 mmol) and Et_3N (5.0 equiv., 1.0 mmol) in 1,4-dioxane (0.2 mL) was added dropwise to the vial and the mixture was stirred for 2 hours at 0 °C. Upon completion, all volatiles were directly removed by rotary evaporator, and the residue was purified by silica gel flash column chromatography to give the desired product (eluent: $CHCl_3$/MeOH = 15/1 ~ 5/1). (*Note:* ***6**-**9** were further purified by reverse phase preparative HPLC to remove Et_3N•HCl impurity.*)

General procedure C: synthesis of 2,3,5,6-tetrafluoro-4-pyridinethioglycosyl donors.

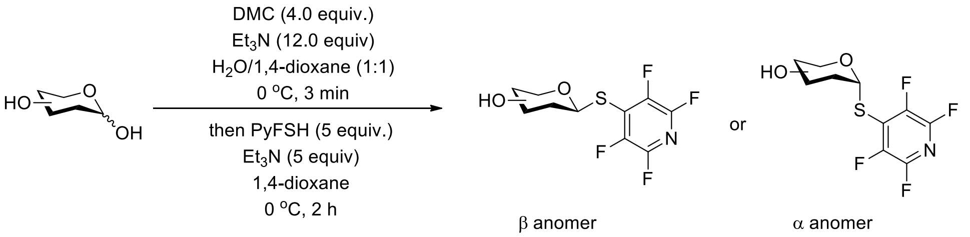

Under air, a 4 mL vial was charged with a magnetic stirring bar, sugar (1.0 equiv., 0.2 mmol), H_2O (0.4 mL), 1,4-dioxane (0.4 mL) and Et_3N (12.0 equiv., 2.4 mmol). The vial was transferred to an ice water bath and cooled to 0 °C over 3 minutes with stirring. Then, DMC (4.0 equiv., 0.8 mmol) in H_2O (0.2 mL) added dropwise to the vial and the mixture was stirred for 3 minutes. After that, a solution of 2,3,5,6-tetrafluoropyridine-4-thiol (PyFSH) (5.0 equiv., 1.0 mmol) and Et_3N (5.0 equiv. 1.0 mmol) in 1,4-dioxane (0.2 mL) was dropwise added to the vial and the mixture was stirred for 2 hours at 0 °C. Upon completion, all volatiles were directly removed by rotary evaporator, the residue was purified by silica gel flash column chromatography to give the

desired product (eluent: $CHCl_3$/MeOH = 15/1 ~ 5/1). (*Note: 0.8 mL H_2O was used for dissolving lactose and cellobiose.* ***2*** *and* ***S1-S7*** *were further purified by reverse phase preparative HPLC to remove Et_3N•HCl impurity.*)

2.3. Preparation of glycosyl radical acceptors

Table S2. Scope of glycosyl radical acceptors

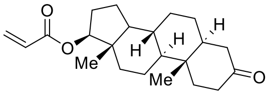

S12

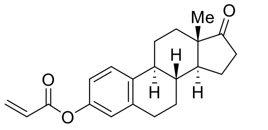

S13

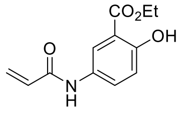

S14

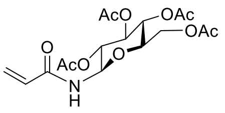

S15

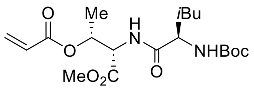

S16

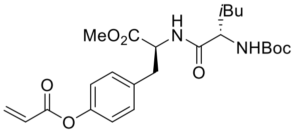

S17

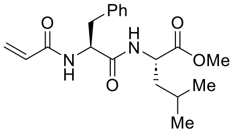

S18

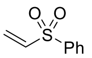

S19

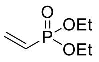

S20

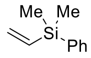

S21

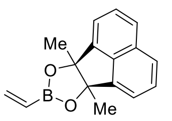

S22

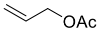

S23

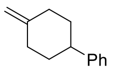

S24

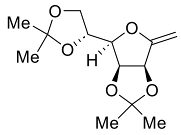

S25

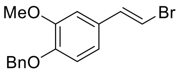

S26

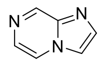

S27

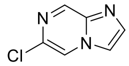

S28

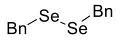

S29

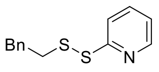

S30

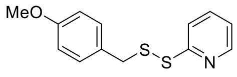

S31

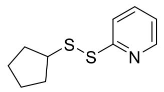

S32

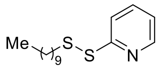

S33

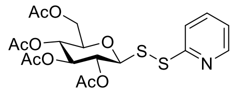

S34

Acceptors **S12-S18**[3], **S22**[4], **S24**[5], **S25**[6], **S26**[7], **S30-S31**[8], **S32-S33**[9], **S34**[10] are known compounds, which were prepared according to the reported methods. Acceptors **S19-S21**, **S23** and **S27-S29** are commercially available substrates.

3. Analytical data of substrates

2,3,5,6-Tetraflupropyridine-4-thiol (PyFSH):

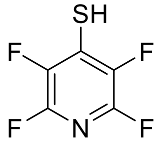

The title compound was prepared according to a known procedure[1] and obtained by distillation (Yield: 96%, 51.5 g).

^{1}H NMR (400 MHz, Chloroform-*d*) δ 4.14 (s, 1H); ^{13}C NMR (101 MHz, Chloroform-*d*) δ 144.67 – 144.34 (m), 142.24 – 141.91 (m), 140.03 – 139.67 (m), 137.49 – 137.13 (m), 129.29 – 128.83 (m); ^{19}F NMR (377 MHz, Chloroform-*d*) δ -90.88 – -91.10 (m, 2F), -139.71 – -139.96 (m, 2F).

NMR data is consistent with the literature report.[1]

2-Chloro-1,3-dimethylimidazolinium chloride (DMC):

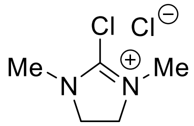

The title compound was prepared according to the **General procedure A** from 1,3-dimethylimidazolidin-2-one (1.0 equiv., 0.1 mol), oxalyl chloride (1.0 equiv., 0.1 mol) and THF (50 mL). The title compound was obtained by filtration as light brown solid (Yield: 71%, 11.93 g). This compound is also commercially available from Sigma-Aldrich.

^{1}H NMR (400 MHz, DMSO-d_6) δ 3.16 (s, 4H), 2.59 (s, 6H); ^{13}C NMR (101 MHz, DMSO-d_6) δ 161.58, 44.70, 31.36.

NMR data is consistent with the literature report.[2]

DMC analogue (3):

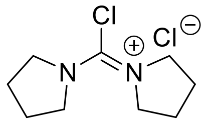

The title compound was prepared according to the **General procedure A** from di(pyrrolidin-1-yl)methanone (1.0 equiv., 0.1 mol), oxalyl chloride (1.0 equiv., 0.1 mol) and THF (50 mL). The title compound was obtained by filtration as brown solid (Yield: 68%, 15.10 g).

^{1}H NMR (400 MHz, DMSO-d_6) δ 3.29 – 3.20 (m, 8H), 1.77 – 1.68 (m, 8H); ^{13}C NMR (101 MHz, DMSO-d_6) δ 159.94, 47.68, 25.14.

NMR data is consistent with the literature report.[2]

DMC analogue (4):

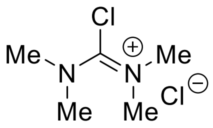

The title compound was prepared according to the **General procedure A** from 1,1,3,3-tetramethylurea (1.0 equiv., 0.1 mol), oxalyl chloride (1.0 equiv., 0.1 mol) and THF (50 mL). The title compound was obtained by filtration as light brown solid (Yield: 75%, 12.75 g).

^{1}H NMR (400 MHz, DMSO-d_6) δ 2.67 (s, 12H); ^{13}C NMR (101 MHz, DMSO-d_6) δ 164.35, 38.47.

NMR data is consistent with the literature report.[2]

(2*R*,3*S*,4*S*,5*R*,6*S*)-2-(Hydroxymethyl)-6-(pyridin-4-ylthio)tetrahydro-2*H*-pyran-3,4,5-triol (6):

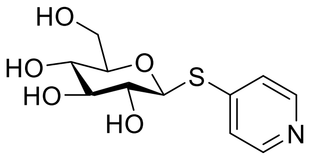

The title compound was prepared according to the **General procedure B** from D-glucose (1.0 equiv., 0.2 mmol), pyridine-4-thiol (5.0 equiv., 1.0 mmol), Et_3N (17.0 equiv., 3.4 mmol), DMC (4.0 equiv., 0.8 mmol), H_2O/1,4-dioxane (0.6 mL/0.6 mL). After silica gel flash column chromatography purification (eluent: $CHCl_3$/MeOH = 15/1 ~ 5/1), the title compound was further purified by reverse phase preparative HPLC to remove Et_3N•HCl impurity, and obtained as white solid (Isolated yield: 58%, 31.7 mg, α:β < 5:95).

Reverse phase details: Column information: Shim-pack GIST, 5 μm C18 column, 20 mm x 250 nm. Flow rate = 10 mL/min. Temperature = 25°C. Solvents used for the eluents were MeCN and H_2O. The eluent was kept constant at 5% MeCN for 5 min, then raised from 5% MeCN to 15% MeCN and kept constant at 15% MeCN for 10 min, then raised from 15% MeCN to 25% MeCN and kept constant at 25% MeCN for 5 min, then raised from 25% MeCN to 95% MeCN and kept constant at 95% MeCN for 5 min. The product was generally eluted at 15% ~ 25% MeCN

constant.

^{1}H NMR (500 MHz, Methanol-d_4) δ 8.37 – 8.31 (m, 2H), 7.52 – 7.42 (m, 2H), 4.96 (d, *J* = 9.8 Hz, 1H), 3.91 (dd, *J* = 12.2, 2.2 Hz, 1H), 3.68 (dd, *J* = 12.2, 6.0 Hz, 1H), 3.51 – 3.44 (m, 2H), 3.40 – 3.34 (m, 2H); ^{13}C NMR (126 MHz, Methanol-d_4) δ 150.69, 149.47, 123.75, 86.33, 82.15, 79.63, 73.77, 71.24, 62.69.

$[\alpha]_D^{25}$: -89.6 (c = 1.0, MeOH).

(2*S*,3*R*,4*S*,5*S*,6*R*)-2-(Benzo[*d*]thiazol-2-ylthio)-6-(hydroxymethyl)tetrahydro-2*H*-pyran-3,4,5-triol (7):

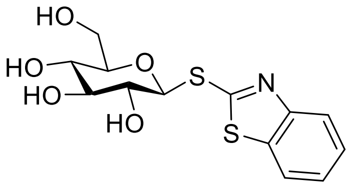

The title compound was prepared according to the **General procedure B** from D-glucose (1.0 equiv., 0.2 mmol), benzo[*d*]thiazole-2-thiol (5.0 equiv., 1.0 mmol), Et_3N (17.0 equiv., 3.4 mmol), DMC (4.0 equiv., 0.8 mmol), H_2O/1,4-dioxane (0.6 mL/0.6 mL). After silica gel flash column chromatography purification (eluent: $CHCl_3$/MeOH = 15/1 ~ 5/1), the title compound was further purified by reverse phase preparative HPLC to remove Et_3N•HCl impurity, and obtained as white solid (Isolated yield: 47%, 30.9 mg, α:β < 5:95).

Reverse phase details: Column information: Shim-pack GIST, 5 μm C18 column, 20 mm x 250 mm. Flow rate = 10 mL/min. Temperature = 25°C. Solvents used for the eluents were MeCN and H_2O. The eluent was kept constant at 5% MeCN for 5 min, then raised from 5% MeCN to 15% MeCN and kept constant at 15% MeCN for 10 min, then raised from 15% MeCN to 25% MeCN and kept constant at 25% MeCN for 5 min, then raised from 25% MeCN to 95% MeCN and kept constant at 95% MeCN for 5 min. The product was generally eluted at 15% ~ 25% MeCN constant.

^{1}H NMR (500 MHz, Methanol-d_4) δ 7.92 – 7.88 (m, 1H), 7.84 (dt, *J* = 8.2, 0.9 Hz, 1H), 7.46 (ddd, *J* = 8.3, 7.2, 1.2 Hz, 1H), 7.37 (ddd, *J* = 8.3, 7.2, 1.2 Hz, 1H), 5.19 (d, *J* = 9.7 Hz, 1H), 3.93 (dd, *J* = 12.3, 2.1 Hz, 1H), 3.76 (dd, *J* = 12.3, 4.9 Hz, 1H), 3.53 – 3.42 (m, 4H); ^{13}C NMR (126 MHz, Methanol-d_4) δ 166.79, 153.70, 136.73, 127.51, 126.01, 122.42, 122.35, 87.99, 82.46, 79.49, 73.88, 70.97, 62.49.

$[\alpha]_D^{25}$: -52.9 (c = 1.0, MeOH).

(2*R*,3*S*,4*S*,5*R*,6*S*)-2-(Hydroxymethyl)-6-((2,3,5,6-tetrafluorophenyl)thio)tetrahydro-2*H*-pyran-3,4,5-triol (8):

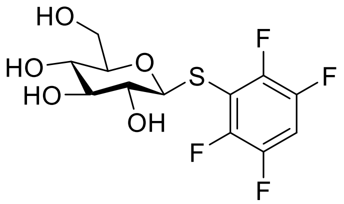

The title compound was prepared according to the **General procedure B** from D-glucose (1.0 equiv., 0.2 mmol), 2,3,5,6-tetrafluorobenzenethiol (5.0 equiv., 1.0 mmol), Et_3N (17.0 equiv., 3.4 mmol), DMC (4.0 equiv., 0.8 mmol), H_2O/1,4-dioxane (0.6 mL/0.6 mL). After silica gel flash column chromatography purification (eluent: $CHCl_3$/MeOH = 15/1 ~ 5/1), the title compound was further purified by reverse phase preparative HPLC to remove Et_3N•HCl impurity, and obtained as viscous gel (Isolated yield: 66%, 45.4 mg, α:β < 5:95).

Reverse phase details: Column information: Shim-pack GIST, 5 μm C18 column, 20 mm x 250 mm. Flow rate = 10 mL/min. Temperature = 25°C. Solvents used for the eluents were MeCN and H_2O. The eluent was kept constant at 5% MeCN for 5 min, then raised from 5% MeCN to 15% MeCN and kept constant at 15% MeCN for 10 min, then raised from 15% MeCN to 25% MeCN and kept constant at 25% MeCN for 5 min, then raised from 25% MeCN to 95% MeCN and kept constant at 95% MeCN for 5 min. The product was generally eluted at 15% ~ 25% MeCN constant.

^{1}H NMR (400 MHz, Methanol-d_4) δ 7.47 – 7.38 (m, 1H), 4.81 (d, *J* = 9.6 Hz, 1H), 3.74 (dd, *J* = 12.1, 2.3 Hz, 1H), 3.60 (dd, *J* = 12.1, 5.4 Hz, 1H), 3.41 – 3.33 (m, 2H), 3.28 (t, *J* = 9.0 Hz, 1H), 3.22 (ddd, *J* = 9.3, 5.4, 2.3 Hz, 1H); ^{13}C NMR (126 MHz, Methanol-d_4) δ 149.41 – 149.25 (m), 148.41 – 148.16 (m), 147.44 – 147.30 (m), 146.44 – 146.19 (m), 113.67 (t, *J* = 20.4 Hz), 107.78 (t, *J* = 23.5 Hz), 87.03 (d, *J* = 2.5 Hz), 82.23, 79.46, 75.39, 71.15, 62.54; ^{19}F NMR (377 MHz, Methanol-d_4) δ -134.82 – -134.99 (m, 2F), -140.80 – -141.02 (m, 2F).

$[\alpha]_D^{25}$: -69.3 (c = 1.0, MeOH).

(2*R*,3*S*,4*S*,5*R*,6*S*)-2-(Hydroxymethyl)-6-(*p*-tolylthio)tetrahydro-2*H*-pyran-3,4,5-triol (9):

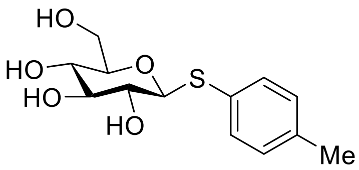

The title compound was prepared according to the **General procedure B** from D-glucose (1.0 equiv., 0.2 mmol), 4-methylbenzenethiol (5.0 equiv., 1.0 mmol), Et_3N (17.0 equiv., 3.4 mmol), DMC (4.0 equiv., 0.8 mmol), H_2O/1,4-dioxane (0.6 mL/0.6 mL). After silica gel flash column chromatography purification (eluent: $CHCl_3$/MeOH = 15/1 ~ 5/1), the title compound was further purified by reverse phase preparative HPLC to remove Et_3N•HCl impurity, and obtained as white solid (Isolated yield: 71%, 40.6 mg, α:β < 5:95).

Reverse phase details: Column information: Shim-pack GIST, 5 μm C18 column, 20 mm x 250 mm. Flow rate = 10 mL/min. Temperature = 25°C. Solvents used for the eluents were MeCN and H_2O. The eluent was kept constant at 5% MeCN for 5 min, then raised from 5% MeCN to 15% MeCN and kept constant at 15% MeCN for 10 min, then raised from 15% MeCN to 25% MeCN and kept constant at 25% MeCN for 5 min, then raised from 25% MeCN to 95% MeCN and kept constant at 95% MeCN for 5 min. The product was generally eluted at 15% ~ 25% MeCN constant.

^{1}H NMR (400 MHz, Methanol-d_4) δ 7.49 – 7.43 (m, 2H), 7.12 (d, J = 8.0 Hz, 2H), 4.52 (d, J = 9.7 Hz, 1H), 3.86 (dd, J = 12.0, 1.7 Hz, 1H), 3.70 – 3.63 (m, 1H), 3.41 – 3.35 (m, 1H), 3.30 – 3.24 (m, 2H), 3.19 (dd, J = 9.8, 8.7 Hz, 1H), 2.30 (s, 3H); ^{13}C NMR (101 MHz, Methanol-d_4) δ 138.71, 133.46, 131.12, 130.50, 89.57, 81.93, 79.59, 73.62, 71.30, 62.84, 21.09.

$[\alpha]_D^{25}$: -47.1 (c = 1.0, MeOH).

(2*R*,3*S*,4*S*,5*R*,6*S*)-2-(Hydroxymethyl)-6-((perfluoropyridin-4-yl)thio)tetrahydro-2*H*-pyran-3,4,5-triol (2):

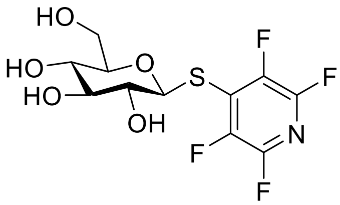

The title compound was prepared according to the **General procedure C** from D-glucose (1.0 equiv., 0.2 mmol), PyFSH (5.0 equiv., 1.0 mmol), Et_3N (17.0 equiv., 3.4 mmol), DMC (4.0 equiv.,

0.8 mmol), H_2O/1,4-dioxane (0.6 mL/0.6 mL). After silica gel flash column chromatography purification (eluent: $CHCl_3$/MeOH = 15/1 ~ 5/1), the title compound was further purified by reverse phase preparative HPLC to remove Et_3N•HCl impurity, and obtained as white solid (^{19}F NMR yield: 85%; isolated yield: 72%, 49.7 mg, α:β < 5:95).

3 mmol scale reaction: Under air atmosphere, to a 50 mL round-bottom flask was charged with a magnetic stirring bar, sugar (1.0 equiv., 3.0 mmol), H_2O (6.0 mL), 1,4-dioxane (6.0 mL) and Et_3N (12.0 equiv., 36.0 mmol) were added. The mixture was transferred to ice water bath and cold for 3 minutes. Then, DMC (4.0 equiv., 12.0 mmol) in H_2O (3.0 mL) was dropwise added to the pre-stirred sugar solution and kept stirring for 3 minutes. After that, a solution of PyFSH (5.0 equiv., 15.0 mmol) and Et_3N (5.0 equiv., 15.0 mmol) in 1,4-dioxane (3.0 mL) was dropwise added to the mixture and kept stirring for 2 hours. Upon completion, all volatiles were directly removed by rotary evaporator, and the residue was purified by silica gel flash column chromatography (eluent: $CHCl_3$/MeOH = 15/1 to 5/1) to give the desired product with Et_3N•HCl impurity, which was further purified by reverse phase preparative HPLC to give the title compound as white solid (^{19}F NMR yield: 65%; isolated yield: 54%, 559 mg, α:β = 12:88).

Reverse phase details: Column information: Shim-pack GIST, 5 μm C18 column, 20 mm x 250 mm. Flow rate = 10 mL/min. Temperature = 25°C. Solvents used for the eluents were MeCN and H_2O. The eluent was kept constant at 5% MeCN for 5 min, then raised from 5% MeCN to 15% MeCN and kept constant at 15% MeCN for 10 min, then raised from 15% MeCN to 25% MeCN and kept constant at 25% MeCN for 5 min, then raised from 25% MeCN to 95% MeCN and kept constant at 95% MeCN for 5 min. The product was generally eluted at 15% ~ 25% MeCN constant.

^1H NMR (500 MHz, Deuterium Oxide) δ **5.15 (d, *J* = 9.8 Hz, 1H, anomeric H)**, 3.83 (dd, *J* = 12.5, 2.1 Hz, 1H), 3.66 (dd, *J* = 12.5, 5.4 Hz, 1H), 3.55 (t, *J* = 8.7 Hz, 1H), 3.49 – 3.41 (m, 3H); ^{13}C NMR (126 MHz, Deuterium Oxide) δ 144.45 – 144.18 (m), 143.03 – 1442.76 (m), 142.51 – 142.25 (m), 141.00 – 140.73 (m), 127.27 – 126.95 (m), 84.64 (t, *J* = 3.2 Hz), 80.23, 77.03, 72.96, 69.16, 60.48; ^{19}F NMR (377 MHz, Deuterium Oxide) δ -92.46 – -92.78 (m, 2F), -136.24 – -136.50 (m, 2F); HRMS (ESI) m/z calcd for $C_{11}H_{11}F_4NNaO_5S$ [$(M+Na)^+$]: 368.0186, found: 368.0181.

$[\alpha]_D^{25}$: -61.9 (c = 1.0, MeOH).

(2*R*,3*R*,4*S*,5*R*,6*S*)-2-(Hydroxymethyl)-6-((perfluoropyridin-4-yl)thio)tetrahydro-2*H*-pyran-3,4,5-triol (S1):

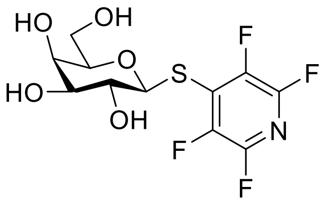

The title compound was prepared according to the **General procedure C** from D-galactose (1.0 equiv., 0.2 mmol), PyFSH (5.0 equiv., 1.0 mmol), Et_3N (17.0 equiv., 3.4 mmol), DMC (4.0 equiv., 0.8 mmol), H_2O/1,4-dioxane (0.6 mL/0.6 mL). After silica gel flash column chromatography purification (eluent: $CHCl_3$/MeOH = 15/1 ~ 5/1), the title compound was further purified by preparative HPLC to remove Et_3N•HCl impurity, and obtained as viscous gel (^{19}F NMR yield: 25%; isolated yield: 16%, 11.0 mg, α:β = 10:90).

Reverse phase details: Column information: Shim-pack GIST, 5 μm C18 column, 20 mm x 250 mm. Flow rate = 10 mL/min. Temperature = 25°C. Solvents used for the eluents were MeCN and H_2O. The eluent was kept constant at 5% MeCN for 5 min, then raised from 5% MeCN to 15% MeCN and kept constant at 15% MeCN for 10 min, then raised from 15% MeCN to 25% MeCN and kept constant at 25% MeCN for 5 min, then raised from 25% MeCN to 95% MeCN and kept constant at 95% MeCN for 5 min. The product was generally eluted at 15% ~ 25% MeCN constant.

^{1}H NMR (400 MHz, Methanol-*d*$_4$) δ **5.13 (d, *J* = 9.6 Hz, 1H, anomeric H)**, 3.89 (dd, *J* = 3.4, 1.1 Hz, 1H), 3.68 (t, *J* = 9.4 Hz, 1H), 3.64 – 3.60 (m, 2H), 3.59 – 3.55 (m, 1H), 3.53 (dd, *J* = 9.2, 3.4 Hz, 1H); ^{13}C NMR (126 MHz, Methanol-*d*$_4$) δ 145.73 – 145.45 (m), 143.95 – 143.67 (m), 143.64 – 143.52 (m), 141.92 – 141.65 (m), 130.30 – 129.97 (m), 85.58 (t, *J* = 4.1 Hz), 81.32, 76.07, 72.59, 70.39, 62.34; ^{19}F NMR (377 MHz, Methanol-*d*$_4$) δ -94.75 – -94.99 (m, 2F), -139.08 – -139.37 (m, 2F); HRMS (ESI) m/z calcd for $C_{11}H_{11}F_4NNaO_5S$ [$(M+Na)^+$]: 368.0186, found: 368.0184.

$[\alpha]_D^{25}$: -96.2 (c = 1.0, MeOH).

(2*R*,3*S*,4*S*,5*S*,6*R*)-2-(Hydroxymethyl)-6-((perfluoropyridin-4-yl)thio)tetrahydro-2*H*-pyran-3,4,5-triol (S2):

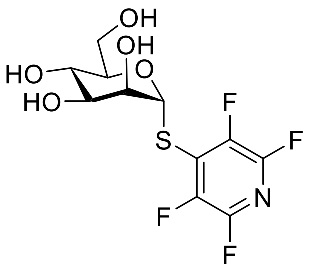

The title compound was prepared according to the **General procedure C** from D-mannose (1.0 equiv., 0.2 mmol), PyFSH (5.0 equiv., 1.0 mmol), Et_3N (17.0 equiv., 3.4 mmol), DMC (4.0 equiv., 0.8 mmol), H_2O/1,4-dioxane (0.6 mL/0.6 mL). After silica gel flash column chromatography purification (eluent: $CHCl_3$/MeOH = 15/1 ~ 5/1), the title compound was further purified by preparative HPLC to remove Et_3N•HCl impurity, and obtained as white solid (^{19}F NMR yield: 68%; isolated yield: 54%, 37.3 mg, α:β > 95:5).

Reverse phase details: Column information: Shim-pack GIST, 5 μm C18 column, 20 mm x 250 mm. Flow rate = 10 mL/min. Temperature = 25°C. Solvents used for the eluents were MeCN and H_2O. The eluent was kept constant at 5% MeCN for 5 min, then raised from 5% MeCN to 15% MeCN and kept constant at 15% MeCN for 10 min, then raised from 15% MeCN to 25% MeCN and kept constant at 25% MeCN for 5 min, then raised from 25% MeCN to 95% MeCN and kept constant at 95% MeCN for 5 min. The product was generally eluted at 15% ~ 25% MeCN constant.

^{1}H NMR (500 MHz, Methanol-d_4) δ **5.87 (d, *J* = 1.4 Hz, 1H, anomeric H)**, 4.10 (dd, *J* = 3.5, 1.5 Hz, 1H), 3.84 – 3.79 (m, 2H), 3.72 (dd, *J* = 12.1, 2.3 Hz, 1H), 3.66 (t, *J* = 9.6 Hz, 1H), 3.59 (dd, *J* = 12.1, 6.4 Hz, 1H); ^{13}C NMR (126 MHz, Methanol-d_4) δ 145.92 – 145.64 (m), 145.24 – 144.96 (m), 143.98 – 143.69 (m), 143.21 – 142.93 (m), 128.32 – 127.99 (m), 87.99 (t, *J* = 3.2 Hz), 77.04, 72.75, 72.66, 68.39, 62.29; ^{19}F NMR (377 MHz, Methanol-d_4) δ -93.63 – -93.87 (m, 2F), -137.58 – -137.80 (m, 2F); HRMS (ESI) m/z calcd for $C_{11}H_{11}F_4NNaO_5S$ [$(M+Na)^+$]: 368.0186, found: 368.0187.

$[\alpha]_D^{25}$: +249.3 (c = 1.0, MeOH).

(2*S*,3*R*,4*R*,5*R*,6*S*)-2-Methyl-6-((perfluoropyridin-4-yl)thio)tetrahydro-2*H*-pyran-3,4,5-triol (S3):

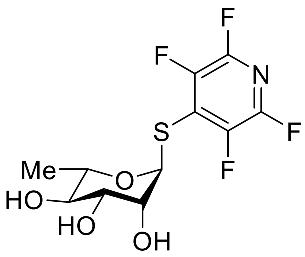

The title compound was prepared according to the **General procedure C** from L-rhamnose (1.0 equiv., 0.2 mmol), PyFSH (5.0 equiv., 1.0 mmol), Et_3N (17.0 equiv., 3.4 mmol), DMC (4.0 equiv., 0.8 mmol), H_2O/1,4-dioxane (0.6 mL/0.6 mL). After silica gel flash column chromatography purification (eluent: $CHCl_3$/MeOH = 15/1 ~ 5/1), the title compound was further purified by preparative HPLC to remove Et_3N•HCl impurity, and obtained as viscous gel (^{19}F NMR yield: 68%; isolated yield: 51%, 33.6 mg, α:β > 95:5).

Reverse phase details: Column information: Shim-pack GIST, 5 μm C18 column, 20 mm x 250 mm. Flow rate = 10 mL/min. Temperature = 25°C. Solvents used for the eluents were MeCN and H_2O. The eluent was kept constant at 5% MeCN for 5 min, then raised from 5% MeCN to 15% MeCN and kept constant at 15% MeCN for 10 min, then raised from 15% MeCN to 25% MeCN and kept constant at 25% MeCN for 5 min, then raised from 25% MeCN to 95% MeCN and kept constant at 95% MeCN for 5 min. The product was generally eluted at 15% ~ 25% MeCN constant.

^1H NMR (400 MHz, Methanol-d_4) δ **5.73 (s, 1H, anomeric H)**, 4.11 (dd, J = 3.5, 1.5 Hz, 1H), 3.91 (dq, J = 9.2, 6.2 Hz, 1H), 3.75 (dd, J = 9.4, 3.5 Hz, 1H), 3.47 (t, J = 9.4 Hz, 1H), 1.18 (d, J = 6.2 Hz, 3H); ^{13}C NMR (126 MHz, Methanol-d_4) δ 145.91 – 145.63 (m), 145.12 – 144.85 (m), 143.96 – 143.69 (m), 143.09 – 142.81 (m), 129.02 – 128.69 (m), 88.92 (t, J = 3.1 Hz), 73.53, 73.13, 72.47, 72.31, 17.71; ^{19}F NMR (377 MHz, Methanol-d_4) δ -93.44 – -93.62 (m, 2F), -137.83 – -138.07 (m, 2F); HRMS (ESI) m/z calcd for $C_{11}H_{11}F_4NO_4S$ [M^+]: 329.0345, found: 329.0351. $[\alpha]_D^{25}$: -300.0 (c = 1.0, MeOH).

(2*R*,3*S*,4*R*,5*R*,6*S*)-2-(Hydroxymethyl)-6-((perfluoropyridin-4-yl)thio)tetrahydro-2*H*-pyran-3,4,5-triol (S4):

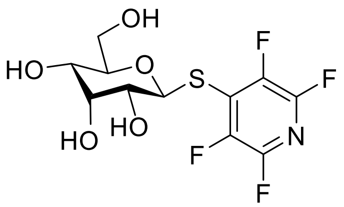

The title compound was prepared according to the **General procedure C** from D-allose (1.0 equiv., 0.2 mmol), PyFSH (5.0 equiv., 1.0 mmol), Et_3N (17.0 equiv., 3.4 mmol), DMC (4.0 equiv., 0.8 mmol), H_2O/1,4-dioxane (0.6 mL/0.6 mL). After silica gel flash column chromatography purification (eluent: $CHCl_3$/MeOH = 15/1 ~ 5/1), the title compound was further purified by preparative HPLC to remove Et_3N•HCl impurity, and obtained as viscous gel (^{19}F NMR yield: 55%; isolated yield: 40%, 27.6 mg, α:β = 9:91).

Reverse phase details: Column information: Shim-pack GIST, 5 μm C18 column, 20 mm x 250 mm. Flow rate = 10 mL/min. Temperature = 25°C. Solvents used for the eluents were MeCN and H_2O. The eluent was kept constant at 5% MeCN for 5 min, then raised from 5% MeCN to 15% MeCN and kept constant at 15% MeCN for 10 min, then raised from 15% MeCN to 25% MeCN and kept constant at 25% MeCN for 5 min, then raised from 25% MeCN to 95% MeCN and kept constant at 95% MeCN for 5 min. The product was generally eluted at 15% ~ 25% MeCN constant.

^{1}H NMR (400 MHz, Methanol-d_4) δ **5.54 (d, *J* = 9.7 Hz, 1H, anomeric H)**, 4.09 (t, *J* = 3.0 Hz, 1H), 3.75 (dd, *J* = 11.9, 2.2 Hz, 1H), 3.65 (ddd, *J* = 9.9, 5.8, 2.1 Hz, 1H), 3.59 – 3.54 (m, 1H), 3.54 – 3.48 (m, 2H); ^{13}C NMR (126 MHz, Methanol-d_4) δ 145.77 – 145.49 (m), 143.88 – 143.70 (m), 143.70 – 143.57 (m), 141.85 – 141.58 (m), 130.69 – 130.40 (m), 82.24 (t, *J* = 4.4 Hz), 78.50, 73.19, 72.95, 68.53, 62.77; ^{19}F NMR (377 MHz, Methanol-d_4) δ -95.04 – -95.22 (m, 2F), -139.52 – -139.89 (m, 2F); HRMS (ESI) m/z calcd for $C_{11}H_{11}F_4NNaO_5S$ [(M+Na)$^+$]: 368.0186, found: 368.0171.

$[\alpha]_D^{25}$: +30.3 (c = 1.0, MeOH).

(2*R*,3*S*,4*S*,5*R*)-2-((Perfluoropyridin-4-yl)thio)tetrahydro-2*H*-pyran-3,4,5-triol (S5):

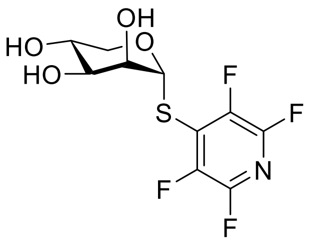

The title compound was prepared according to the **General procedure C** from D-lyxose (1.0 equiv., 0.2 mmol), PyFSH (5.0 equiv., 1.0 mmol), Et_3N (17.0 equiv., 3.4 mmol), DMC (4.0 equiv., 0.8 mmol), H_2O/1,4-dioxane (0.6 mL/0.6 mL). After silica gel flash column chromatography purification (eluent: $CHCl_3$/MeOH = 15/1 ~ 5/1), the title compound was further purified by preparative HPLC to remove Et_3N•HCl impurity, and obtained as viscous gel (^{19}F NMR yield: 83%; isolated yield: 66%, 41.6 mg, α:β > 95:5).

Reverse phase details: Column information: Shim-pack GIST, 5 μm C18 column, 20 mm x 250 mm. Flow rate = 10 mL/min. Temperature = 25°C. Solvents used for the eluents were MeCN and H_2O. The eluent was kept constant at 5% MeCN for 5 min, then raised from 5% MeCN to 15% MeCN and kept constant at 15% MeCN for 10 min, then raised from 15% MeCN to 25% MeCN and kept constant at 25% MeCN for 5 min, then raised from 25% MeCN to 95% MeCN and kept constant at 95% MeCN for 5 min. The product was generally eluted at 15% ~ 25% MeCN constant.

^{1}H NMR (400 MHz, Methanol-*d*$_4$) δ **5.65 (d, *J* = 5.0 Hz, 1H, anomeric H)**, 3.99 (dd, *J* = 5.1, 2.2 Hz, 1H), 3.85 – 3.80 (m, 2H), 3.78 – 3.67 (m, 2H); ^{13}C NMR (126 MHz, Methanol-*d*$_4$) δ 145.84 – 145.56 (m), 144.64 – 144.36 (m), 143.90 – 143.62 (m), 142.61 – 142.33 (m), 129.58 – 129.27 (m), 86.85, 72.48, 71.73, 69.11, 67.07; ^{19}F NMR (377 MHz, Methanol-*d*$_4$) δ -93.94 – -94.16 (m, 2F), -138.27 – -138.68 (m, 2F).

$[\alpha]_D^{25}$: +176.4 (c = 1.0, MeOH).

(2*R*,3*R*,4*R*,5*S*)-2-(Hydroxymethyl)-5-((perfluoropyridin-4-yl)thio)tetrahydrofuran-3,4-diol (S6):

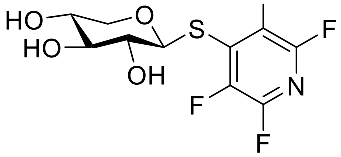

The title compound was prepared according to the **General procedure C** from D-xylose (1.0 equiv., 0.2 mmol), PyFSH (5.0 equiv., 1.0 mmol), Et_3N (17.0 equiv., 3.4 mmol), DMC (4.0 equiv., 0.8 mmol), H_2O/1,4-dioxane (0.6 mL/0.6 mL). After silica gel flash column chromatography purification (eluent: $CHCl_3$/MeOH = 15/1 ~ 5/1), the title compound was further purified by preparative HPLC to remove Et_3N•HCl impurity, and obtained as viscous gel (^{19}F NMR yield: 83%; isolated yield: 62%, 39.0 mg, α:β = 9:91).

Reverse phase details: Column information: Shim-pack GIST, 5 μm C18 column, 20 mm x 250 mm. Flow rate = 10 mL/min. Temperature = 25°C. Solvents used for the eluents were MeCN and H_2O. The eluent was kept constant at 5% MeCN for 5 min, then raised from 5% MeCN to 15% MeCN and kept constant at 15% MeCN for 10 min, then raised from 15% MeCN to 25% MeCN and kept constant at 25% MeCN for 5 min, then raised from 25% MeCN to 95% MeCN and kept constant at 95% MeCN for 5 min. The product was generally eluted at 15% ~ 25% MeCN constant.

^{1}H NMR (400 MHz, Methanol-d_4) δ **5.13 (d, *J* = 8.0 Hz, 1H, anomeric H)**, 3.98 (dd, *J* = 11.5, 4.9 Hz, 1H), 3.61 – 3.51 (m, 1H), 3.47 – 3.36 (m, 2H), 3.28 (dd, *J* = 11.5, 9.4 Hz, 1H); ^{13}C NMR (126 MHz, Methanol-d_4) δ 145.75 – 145.48 (m), 144.16 – 143.88 (m), 143.82 – 143.54 (m), 142.13 – 141.85 (m), 130.19 – 129.90 (m), 86.47 (t, *J* = 3.7 Hz), 78.09, 75.14, 70.66, 70.03; ^{19}F NMR (377 MHz, Methanol-d_4) δ -94.15 – -94.51 (m, 2F), -137.97 – -139.05 (m, 2F); HRMS (ESI) m/z calcd for $C_{10}H_9F_4NNaO_4S$ [$(M+Na)^+$]: 338.0081, found: 338.0079.

$[\alpha]_D^{25}$: -74.1 (c = 1.0, MeOH).

(2*S*,3*R*,4*R*,5*S*,6*S*)-2-(Hydroxymethyl)-6-((perfluoropyridin-4-yl)thio)tetrahydro-2*H*-pyran-3,4,5-triol (S7):

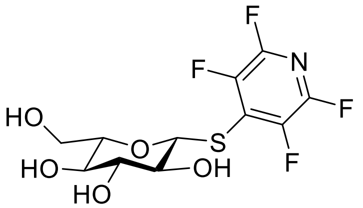

The title compound was prepared according to the **General procedure C** from L-glucose (1.0 equiv., 0.2 mmol), PyFSH (5.0 equiv., 1.0 mmol), Et_3N (17.0 equiv., 3.4 mmol), DMC (4.0 equiv., 0.8 mmol), H_2O/1,4-dioxane (0.6 mL/0.6 mL). After silica gel flash column chromatography

purification (eluent: $CHCl_3$/MeOH = 15/1 ~ 5/1), the title compound was further purified by preparative HPLC to remove Et_3N•HCl impurity, and obtained as viscous gel (^{19}F NMR yield: 74%; isolated yield: 59%, 40.7 mg, α:β = 5:95).

Reverse phase details: Column information: Shim-pack GIST, 5 μm C18 column, 20 mm x 250 mm. Flow rate = 10 mL/min. Temperature = 25°C. Solvents used for the eluents were MeCN and H_2O. The eluent was kept constant at 5% MeCN for 5 min, then raised from 5% MeCN to 15% MeCN and kept constant at 15% MeCN for 10 min, then raised from 15% MeCN to 25% MeCN and kept constant at 25% MeCN for 5 min, then raised from 25% MeCN to 95% MeCN and kept constant at 95% MeCN for 5 min. The product was generally eluted at 15% ~ 25% MeCN constant.

^{1}H NMR (400 MHz, Methanol-d_4) δ **5.18 (d, *J* = 9.5 Hz, 1H, anomeric H)**, 3.77 (dd, *J* = 12.2, 1.5 Hz, 1H), 3.61 – 3.54 (m, 1H), 3.44 – 3.38 (m, 1H), 3.37 – 3.31 (m, 3H); ^{13}C NMR (126 MHz, Methanol-d_4) δ 145.76 – 145.48 (m), 143.89 – 143.69 (m), 143.67 – 143.55 (m), 141.86 – 141.58 (m), 130.18 – 129.90 (m), 84.91 (t, *J* = 4.3 Hz), 82.61, 79.49, 75.61, 71.24, 62.51; ^{19}F NMR (377 MHz, Methanol-d_4) δ -94.74 – -94.97 (m, 2F), -139.30 – -139.58 (m, 2F); HRMS (ESI) m/z calcd for $C_{11}H_{11}F_4NNaO_5S$ $[(M+Na)^+]$: 368.0186, found: 368.0187.
$[\alpha]_D^{25}$: +24.4 (c = 1.0, MeOH).

(2*R*,3*R*,4*S*,5*S*,6*R*)-2-(((2*R*,3*S*,4*R*,5*R*,6*R*)-4,5-Dihydroxy-2-(hydroxymethyl)-6-((perfluoropyridin-4-yl)thio)tetrahydro-2*H*-pyran-3-yl)oxy)-6-(hydroxymethyl)tetrahydro-2*H*-pyran-3,4,5-triol (13):

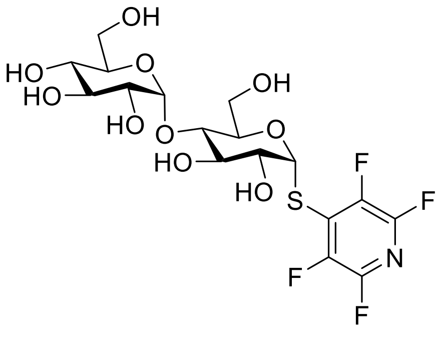

The title compound was prepared according to the **General procedure C** from maltose (1.0 equiv., 0.2 mmol), PyFSH (5.0 equiv., 1.0 mmol), Et_3N (17.0 equiv., 3.4 mmol), DMC (4.0 equiv., 0.8 mmol), H_2O/1,4-dioxane (0.6 mL/0.6 mL). After silica gel flash column chromatography

purification (eluent: $CHCl_3$/MeOH = 15/1 ~ 3/1), the title compound was obtained as viscous gel (^{19}F NMR yield: 44%; isolated yield: 30%, 30.4 mg, α:β > 95:5).

^{1}H NMR (500 MHz, Methanol-d_4) δ 5.21 (d, J = 4.7 Hz, 1H), **5.20 (d, J = 1.2 Hz, 1H, anomeric H)**, 3.85 – 3.81 (m, 1H), 3.79 – 3.72 (m, 2H), 3.72 – 3.59 (m, 5H), 3.48 – 3.42 (m, 2H), 3.42 – 3.38 (m, 1H), 3.26 (t, J = 9.3 Hz, 1H); ^{13}C NMR (126 MHz, Methanol-d_4) δ 145.73, – 145.46 (m), 143.84 – 143.75 (m), 143.69 – 143.53 (m), 141.81 – 141.53 (m), 130.13 – 129.84 (m), 102.72, 84.95 (t, J = 4.1 Hz), 81.08, 80.37, 79.16, 75.19, 74.93, 74.73, 74.03, 71.51, 62.60, 61.87; ^{19}F NMR (377 MHz, Methanol-d_4) δ -93.81 – -95.57 (m, 2F), -138.26 – -140.22 (m, 2F); HRMS (ESI) m/z calcd for $C_{17}H_{22}F_4NO_{10}S$ [(M+H)$^+$]: 508.0895, found: 508.0894.

$[\alpha]_D^{25}$: +12.6 (c = 1.0, MeOH).

(2*S*,3*R*,4*S*,5*S*,6*R*)-2-(((2*R*,3*S*,4*R*,5*R*,6*S*)-4,5-Dihydroxy-2-(hydroxymethyl)-6-((perfluoropyridin-4-yl)thio)tetrahydro-2*H*-pyran-3-yl)oxy)-6-(hydroxymethyl)tetrahydro-2*H*-pyran-3,4,5-triol (S8):

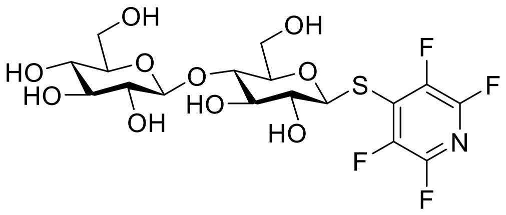

The title compound was prepared according to the **General procedure C** from cellobiose (1.0 equiv., 0.2 mmol), PyFSH (5.0 equiv., 1.0 mmol), Et_3N (17.0 equiv., 3.4 mmol), DMC (4.0 equiv., 0.8 mmol), H_2O/1,4-dioxane (1.0 mL/0.6 mL). After silica gel flash column chromatography purification (eluent: $CHCl_3$/MeOH = 15/1 ~ 3/1), the title compound was obtained as viscous gel (^{19}F NMR yield: 58%; isolated yield: 49%, 49.7 mg, α:β < 5:95).

^{1}H NMR (500 MHz, Methanol-d_4) δ **5.20 (d, J = 9.8 Hz, 1H, anomeric H)**, 4.42 (d, J = 7.8 Hz, 1H), 3.89 (dd, J = 11.9, 2.3 Hz, 1H), 3.80 – 3.75 (m, 2H), 3.67 (dd, J = 11.9, 5.8 Hz, 1H), 3.63 (t, J = 9.1 Hz, 1H), 3.58 (t, J = 8.6 Hz, 1H), 3.46 (dt, J = 9.5, 3.3 Hz, 1H), 3.44 – 3.40 (m, 1H), 3.37 (dd, J = 9.1, 4.5 Hz, 2H), 3.33 – 3.31 (m, 1H), 3.23 (dd, J = 9.2, 7.8 Hz, 1H); ^{13}C NMR (126 MHz, Methanol-d_4) δ 145.73 – 145.47 (m), 143.86 – 143.75 (m), 143.67 – 143.54 (m), 141.83 – 141.56 (m), 130.10 – 129.82 (m), 104.43, 84.81 (t, J = 4.1 Hz), 81.06, 79.87, 78.04, 77.69, 75.37, 74.84, 71.33, 62.33, 61.51; ^{19}F NMR (377 MHz, Methanol-d_4) δ -94.49 – -95.05 (m, 2F), -139.13 – -139.48 (m, 2F); HRMS (ESI) m/z calcd for $C_{17}H_{22}F_4NO_{10}S$ [(M+H)$^+$]: 508.0895, found:

508.0898.

$[\alpha]_D^{25}$: -50.2 (c = 1.0, MeOH).

(2*S*,3*R*,4*S*,5*R*,6*R*)-2-(((2*R*,3*S*,4*R*,5*R*,6*S*)-4,5-Dihydroxy-2-(hydroxymethyl)-6-((perfluoropyridin-4-yl)thio)tetrahydro-2*H*-pyran-3-yl)oxy)-6-(hydroxymethyl)tetrahydro-2*H*-pyran-3,4,5-triol (S9):

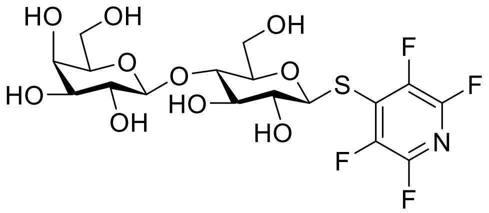

The title compound was prepared according to the **General procedure C** from lactose (1.0 equiv., 0.2 mmol), PyFSH (5.0 equiv., 1.0 mmol), Et_3N (17.0 equiv., 3.4 mmol), DMC (4.0 equiv., 0.8 mmol), H_2O/1,4-dioxane (1.0 mL/0.6 mL). After silica gel flash column chromatography purification (eluent: $CHCl_3$/MeOH = 15/1 ~ 3/1), the title compound was obtained as viscous gel (^{19}F NMR yield: 53%; isolated yield: 41%, 41.5 mg, α:β < 5:95).

^{1}H NMR (500 MHz, Methanol-d_4) δ **5.21 (d, *J* = 9.7 Hz, 1H, anomeric H)**, 4.37 (d, *J* = 7.4 Hz, 1H), 3.83 (dd, *J* = 3.2, 1.0 Hz, 1H), 3.81 – 3.75 (m, 3H), 3.72 (dd, *J* = 11.5, 4.5 Hz, 1H), 3.66 – 3.58 (m, 3H), 3.57 – 3.49 (m, 2H), 3.47 (dt, *J* = 9.3, 3.4 Hz, 1H), 3.42 (t, *J* = 9.0 Hz, 1H); ^{13}C NMR (126 MHz, Methanol-d_4) δ 145.74 – 145.47 (m), 143.88 – 143.79 (m), 143.68 – 143.54 (m), 141.85 – 141.57 (m), 130.09 – 129.80 (m), 104.98, 84.82 (t, *J* = 4.1 Hz), 81.04, 79.99, 77.69, 77.04, 75.31, 74.65, 72.44, 70.31, 62.52, 61.59; ^{19}F NMR (377 MHz, Methanol-d_4) δ -94.49 – -94.84 (m, 2F), -139.08 – -139.47 (m, 2F); HRMS (ESI) m/z calcd for $C_{17}H_{22}F_4NO_{10}S$ $[(M+H)^+]$: 508.0895, found: 508.0894.

$[\alpha]_D^{25}$: -32.1 (c = 1.0, MeOH).

(2*R*,3*R*,4*S*,5*R*,6*S*)-2-(Hydroxymethyl)-6-(((2*R*,3*S*,4*S*,5*R*,6*S*)-3,4,5-trihydroxy-6-((perfluoropyridin-4-yl)thio)tetrahydro-2*H*-pyran-2-yl)methoxy)tetrahydro-2*H*-pyran-3,4,5-triol (S10):

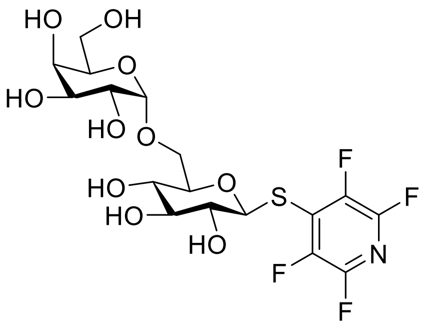

The title compound was prepared according to the **General procedure C** from melibiose (1.0 equiv., 0.2 mmol), PyFSH (5.0 equiv., 1.0 mmol), Et_3N (17.0 equiv., 3.4 mmol), DMC (4.0 equiv., 0.8 mmol), H_2O/1,4-dioxane (0.6 mL/0.6 mL). After silica gel flash column chromatography purification (eluent: $CHCl_3$/MeOH = 15/1 ~ 3/1), the title compound was obtained as viscous gel (^{19}F NMR yield: 70%; isolated yield: 62%, 62.8 mg, α:β = 7:93).

^{1}H NMR (500 MHz, Methanol-*d*$_4$) δ **5.16 (d, *J* = 9.7 Hz, 1H, anomeric H)**, 4.77 (d, *J* = 3.8 Hz, 1H), 3.86 – 3.81 (m, 2H), 3.70 (ddd, *J* = 18.1, 10.6, 2.9 Hz, 2H), 3.63 – 3.60 (m, 3H), 3.55 (ddd, *J* = 9.9, 5.8, 2.0 Hz, 1H), 3.44 (t, *J* = 8.8 Hz, 1H), 3.40 – 3.33 (m, 3H); ^{13}C NMR (126 MHz, Methanol-*d*$_4$) δ 145.71– 145.45 (m), 144.04 – 143.77 (m), 143.77 – 143.51 (m), 142.01 – 141.74 (m), 129.95 – 129.67 (m), 100.04, 85.31 (t, *J* = 3.8 Hz), 80.84, 79.32, 75.36, 72.00, 71.36, 71.30, 70.95, 70.14, 67.93, 62.54; ^{19}F NMR (377 MHz, Methanol-*d*$_4$) δ -93.86 – -94.39 (m, 2F), -138.28 – -139.41 (m, 2F); HRMS (ESI) m/z calcd for $C_{17}H_{22}F_4NO_{10}S$ [$(M+H)^+$]: 508.0895, found: 508.0898.

$[\alpha]_D^{25}$: +46.9 (c = 1.0, MeOH).

(2*R*,3*R*,4*S*,5*S*,6*R*)-2-(((2*R*,3*S*,4*R*,5*R*,6*R*)-6-(((2*R*,3*S*,4*R*,5*R*,6*R*)-4,5-Dihydroxy-2-(hydroxymethyl)-6-((perfluoropyridin-4-yl)thio)tetrahydro-2*H*-pyran-3-yl)oxy)-4,5-dihydroxy-2-(hydroxymethyl)tetrahydro-2*H*-pyran-3-yl)oxy)-6-(hydroxymethyl)tetrahydro-2*H*-pyran-3,4,5-triol (S11):

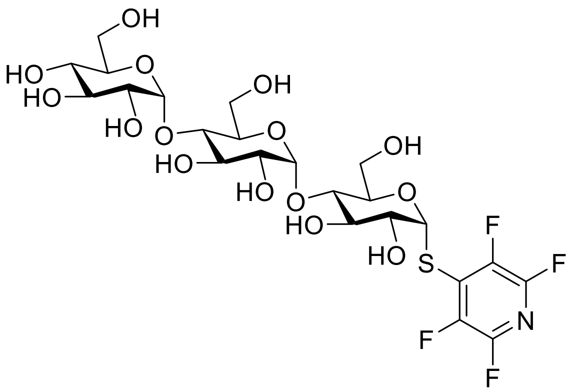

The title compound was prepared according to the **General procedure C** from maltotriose (1.0 equiv., 0.2 mmol), PyFSH (5.0 equiv., 1.0 mmol), Et_3N (17.0 equiv., 3.4 mmol), DMC (4.0 equiv., 0.8 mmol), H_2O/1,4-dioxane (0.6 mL/0.6 mL). After silica gel flash column chromatography purification (eluent: $CHCl_3$/MeOH = 15/1 ~ 2/1), the title compound was obtained as viscous gel (^{19}F NMR yield: 66%; isolated yield: 60%, 80.3 mg, α:β = 95:5).

^{1}H NMR (500 MHz, Methanol-d_4) δ **5.21 (d, *J* = 1.4 Hz, 1H, anomeric H)**, 5.20 (d, *J* = 4.4 Hz, 1H), 5.15 (d, *J* = 3.9 Hz, 1H), 3.88 – 3.78 (m, 4H), 3.77 – 3.65 (m, 6H), 3.61 (td, *J* = 9.3, 8.0 Hz, 2H), 3.53 – 3.48 (m, 2H), 3.45 (ddd, *J* = 11.5, 9.8, 5.8 Hz, 2H), 3.40 (t, *J* = 9.1 Hz, 1H), 3.27 (t, *J* = 9.3 Hz, 1H); ^{13}C NMR (126 MHz, Methanol-d_4) δ 145.75 – 145.47 (m), 143.87 – 143.68 (m), 143.67 – 143.54 (m), 141.84 – 141.56 (m), 129.96 – 129.82 (m), 102.89, 102.51, 84.95 (t, *J* = 4.1 Hz), 81.31, 81.08, 80.37, 79.15, 75.22, 74.99, 74.88, 74.73, 74.14, 73.67, 73.30, 71.49, 62.64, 62.00, 61.95; ^{19}F NMR (377 MHz, Methanol-d_4) δ -94.50 – -94.83 (m, 2F), -139.02 – -139.42 (m, 2F); HRMS (ESI) m/z calcd for $C_{23}H_{31}F_4NNaO_{15}S$ [$(M+Na)^+$]: 692.1243, found: 692.1245.

$[\alpha]_D^{25}$: +91.2 (c = 1.0, MeOH).

***N*-((2*S*,3*R*,4*R*,5*S*,6*R*)-4,5-Dihydroxy-6-(hydroxymethyl)-2-((perfluoropyridin-4-yl)thio)tetra hydro-2*H*-pyran-3-yl)acetamide (S35):**

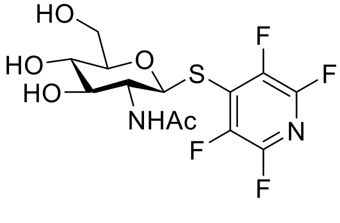

The title compound was prepared according to the **General procedure C** with slight modification, from *N*-Acetylglucosamine (1.0 equiv., 0.2 mmol), PyFSH (5.0 equiv., 1.0 mmol),

Et_3N (10.0 equiv., 2.0 mmol), DMC (4.0 equiv., 0.8 mmol), H_2O/1,4-dioxane (0.6 mL/0.6 mL). After silica gel flash column chromatography purification (eluent: $CHCl_3$/MeOH = 15/1 ~ 5/1), the obtained compound was further washed with small amount of water to remove soluble Et_3N• HCl impurity, and pure compound was obtained by filtration as white solid (^{19}F NMR yield: 88%; isolated yield: 75%, 57.9 mg, α:β < 5:95).

^{1}H NMR (400 MHz, Methanol-d_4) δ 5.29 (**d, *J* = 10.3 Hz, 1H, anomeric H**), 3.85 (t, *J* = 10.0 Hz, 1H), 3.79 (dd, *J* = 12.2, 2.1 Hz, 1H), 3.60 (dd, *J* = 12.2, 5.8 Hz, 1H), 3.51 (dd, *J* = 9.8, 8.4 Hz, 1H), 3.35 (dd, *J* = 9.8, 8.3 Hz, 1H), 3.30 – 3.26 (m, 1H), 2.00 (s, 3H); ^{13}C NMR (101 MHz, Methanol-d_4) δ 173.75, 146.09 – 145.73 (m), 144.34 – 143.99 (m), 143.68 – 143.29 (m), 141.80 – 141.46 (m), 129.93 – 129.52 (m), 84.81 (t, *J* = 4.1 Hz), 82.70, 76.86, 71.67, 62.60, 56.95, 22.82; ^{19}F NMR (376 MHz, Methanol-d_4) δ -94.42 – -94.68 (m, 2F), -139.07 – -139.31 (m, 2F); HRMS (ESI) m/z calcd for $C_{13}H_{14}F_4N_2NaO_5S$ [$(M+Na)^+$]: 409.0452, found: 409.0458.

(5*S*,8*R*,9*S*,10*S*,13*S*,17*S*)-10,13-Dimethyl-3-oxohexadecahydro-1*H*-cyclopenta[*a*]phenanthren-17-yl acrylate (S12):

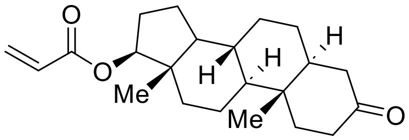

The title compound was prepared according to a known procedure, NMR data is consistent with the literature report.[3]

^{1}H NMR (400 MHz, Chloroform-*d*) δ 6.37 (dd, *J* = 17.4, 1.6 Hz, 1H), 6.11 (dd, *J* = 17.3, 10.4 Hz, 1H), 5.79 (dd, *J* = 10.4, 1.6 Hz, 1H), 4.67 (dd, *J* = 9.2, 7.7 Hz, 1H), 2.43 – 2.24 (m, 3H), 2.23 – 2.14 (m, 1H), 2.08 (ddd, *J* = 15.1, 4.0, 2.1 Hz, 1H), 2.01 (ddd, *J* = 13.2, 6.5, 2.4 Hz, 1H), 1.80 – 1.68 (m, 2H), 1.67 – 1.59 (m, 2H), 1.57 – 1.44 (m, 3H), 1.40 – 1.26 (m, 5H), 1.19 (td, *J* = 12.9, 4.1 Hz, 1H), 1.12 – 1.04 (m, 1H), 1.01 (s, 3H), 0.93 (tt, *J* = 12.2, 6.1 Hz, 1H), 0.83 (s, 3H), 0.80 – 0.71 (m, 1H); ^{13}C NMR (126 MHz, Chloroform-*d*) δ 212.08, 166.43, 130.36, 129.04, 82.98, 53.89, 50.78, 46.78, 44.82, 43.03, 38.66, 38.28, 37.04, 35.89, 35.36, 31.40, 28.94, 27.74, 23.75, 21.09, 12.34, 11.65.

(8*R*,9*S*,13*S*,14*S*)-13-Methyl-17-oxo-7,8,9,11,12,13,14,15,16,17-decahydro-6*H*-cyclopenta[*a*]phenanthren-3-yl acrylate (S13):

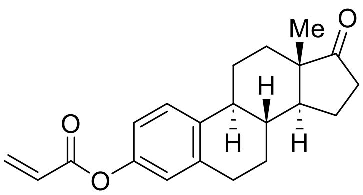

The title compound was prepared according to a known procedure, NMR data is consistent with the literature report.[3]

^{1}H NMR (400 MHz, Chloroform-*d*) δ 7.30 (d, *J* = 8.4 Hz, 1H), 6.93 – 6.84 (m, 2H), 6.59 (dd, *J* = 17.3, 1.3 Hz, 1H), 6.31 (dd, *J* = 17.3, 10.4 Hz, 1H), 6.00 (dd, *J* = 10.4, 1.3 Hz, 1H), 2.96 – 2.87 (m, 2H), 2.57 – 2.46 (m, 1H), 2.45 – 2.36 (m, 1H), 2.35 – 2.24 (m, 1H), 2.15 (dt, *J* = 18.5, 8.6 Hz, 1H), 2.09 – 1.94 (m, 3H), 1.67 – 1.58 (m, 3H), 1.54 – 1.41 (m, 3H), 0.91 (s, 3H); ^{13}C NMR (126 MHz, Chloroform-*d*) δ 220.97, 165.02, 148.62, 138.20, 137.61, 132.58, 128.18, 126.59, 121.69, 118.86, 50.60, 48.12, 44.33, 38.17, 36.03, 31.72, 29.57, 26.51, 25.92, 21.76, 14.00.

Ethyl 5-acrylamido-2-hydroxybenzoate (S14):

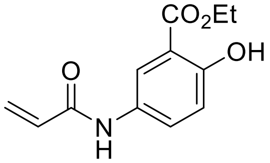

The title compound was prepared according to a known procedure, NMR data is consistent with the literature report.[3]

^{1}H NMR (400 MHz, Chloroform-*d*) δ 10.73 (br., 1H), 8.11 (d, *J* = 2.7 Hz, 1H), 7.58 (dd, *J* = 9.0, 2.7 Hz, 1H), 7.43 (s, 1H), 6.94 (d, *J* = 8.9 Hz, 1H), 6.43 (d, *J* = 16.8 Hz, 1H), 6.23 (dd, *J* = 16.9, 10.2 Hz, 1H), 5.76 (d, *J* = 10.2 Hz, 1H), 4.39 (q, *J* = 7.1 Hz, 2H), 1.39 (t, *J* = 7.1 Hz, 3H); ^{13}C NMR (101 MHz, Chloroform-*d*) δ 169.97, 163.77, 158.90, 131.03, 129.39, 128.78, 128.04, 121.84, 118.17, 112.59, 61.86, 14.36.

(2*R*,3*R*,4*S*,5*R*,6*R*)-2-(Acetoxymethyl)-6-acrylamidotetrahydro-2*H*-pyran-3,4,5-triyl triacetate (S15):

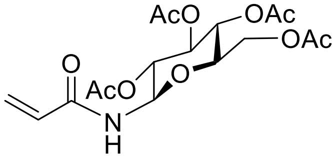

The title compound was prepared according to a known procedure, NMR data is consistent with the literature report.[3]

^{1}H NMR (400 MHz, Chloroform-*d*) δ 6.71 (d, *J* = 9.3 Hz, 1H), 6.26 (dd, *J* = 17.1, 1.1 Hz, 1H), 6.03 (dd, *J* = 17.0, 10.4 Hz, 1H), 5.69 (dd, *J* = 10.3, 1.1 Hz, 1H), 5.30 (q, *J* = 9.7 Hz, 2H), 5.02 (t, *J* = 9.7 Hz, 1H), 4.92 (t, *J* = 9.6 Hz, 1H), 4.27 (dd, *J* = 12.5, 4.4 Hz, 1H), 4.04 (dd, *J* = 12.5, 2.1 Hz, 1H), 3.82 (ddd, *J* = 10.2, 4.5, 2.2 Hz, 1H), 2.02 (s, 3H), 1.98 (d, *J* = 1.5 Hz, 6H), 1.97 (s, 3H); ^{13}C NMR (101 MHz, Chloroform-*d*) δ 171.04, 170.71, 169.94, 169.68, 165.64, 130.12, 128.59, 78.37, 73.67, 72.86, 70.74, 68.27, 61.80, 20.76, 20.68, 20.63, 20.61.

Methyl *O*-acryloyl-*N*-((*tert*-butoxycarbonyl)-*D*-leucyl)-*L*-threoninate (S16):

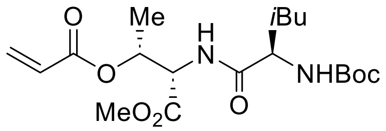

The title compound was prepared according to a known procedure, NMR data is consistent with the literature report.[3]

^{1}H NMR (400 MHz, Chloroform-*d*) δ 6.79 (d, *J* = 9.4 Hz, 1H), 6.40 (d, *J* = 17.3 Hz, 1H), 6.06 (ddd, *J* = 17.3, 10.4, 1.0 Hz, 1H), 5.84 (dt, *J* = 10.4, 1.2 Hz, 1H), 5.48 (qd, *J* = 6.4, 2.7 Hz, 1H), 4.92 (s, 1H), 4.80 (dd, *J* = 9.3, 2.7 Hz, 1H), 4.21 – 4.10 (m, 1H), 3.70 (s, 3H), 1.75 – 1.63 (m, 2H), 1.54 – 1.46 (m, 1H), 1.42 (s, 9H), 1.27 (d, *J* = 6.4 Hz, 3H), 0.94 (t, *J* = 6.4 Hz, 6H); ^{13}C NMR (101 MHz, Chloroform-*d*) δ 173.12, 170.05, 165.02, 155.89, 131.82, 128.04, 80.35, 70.88, 55.48, 53.29, 52.84, 40.79, 28.44, 24.85, 23.02, 22.20, 16.95.

4-((*S*)-2-((*S*)-2-((*tert*-Butoxycarbonyl)amino)-4-methylpentanamido)-3-methoxy-3-oxopropyl)phenyl acrylate (S17):

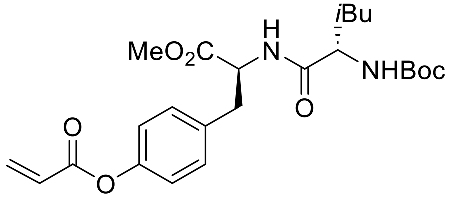

The title compound was prepared according to a known procedure, NMR data is consistent with the literature report.[3]

^{1}H NMR (400 MHz, Chloroform-*d*) δ 7.15 – 7.11 (m, 2H), 7.07 – 7.03 (m, 2H), 6.62 – 6.53 (m,

2H), 6.30 (dd, J = 17.3, 10.4 Hz, 1H), 6.00 (dd, J = 10.4, 1.3 Hz, 1H), 4.84 (dt, J = 7.8, 5.9 Hz, 2H), 4.13 – 4.02 (m, 1H), 3.70 (s, 3H), 3.18 – 3.04 (m, 2H), 1.68 – 1.57 (m, 2H), 1.43 (s, 10H), 0.91 (dd, J = 6.3, 5.1 Hz, 6H); ^{13}C NMR (101 MHz, Chloroform-*d*) δ 172.37, 171.72, 164.59, 155.67, 149.83, 133.64, 132.69, 130.51, 128.09, 121.74, 80.25, 53.25, 52.51, 41.24, 37.52, 28.44, 24.86, 23.04, 22.08.

Methyl acryloyl-*L*-phenylalanyl-*L*-leucinate (S18):

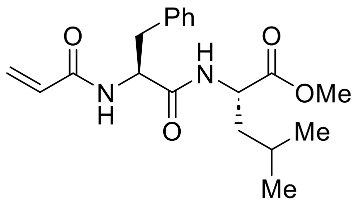

The title compound was prepared according to a known procedure, NMR data is consistent with the literature report.[3]

^{1}H NMR (400 MHz, Chloroform-*d*) δ 7.21 – 7.09 (m, 5H), 6.92 (d, J = 8.1 Hz, 1H), 6.87 (d, J = 7.9 Hz, 1H), 6.19 (dd, J = 17.0, 1.6 Hz, 1H), 6.06 (dd, J = 17.0, 10.1 Hz, 1H), 5.55 (dd, J = 10.1, 1.7 Hz, 1H), 4.85 (q, J = 6.9 Hz, 1H), 4.45 (td, J = 8.3, 5.3 Hz, 1H), 3.63 (s, 3H), 3.09 – 2.95 (m, 2H), 1.57 – 1.35 (m, 3H), 0.79 (d, J = 6.2 Hz, 6H); ^{13}C NMR (101 MHz, Chloroform-*d*) δ 172.90, 171.38, 165.54, 136.66, 130.59, 129.59, 128.61, 127.17, 127.01, 54.49, 52.37, 51.14, 41.26, 38.62, 24.87, 22.79, 22.03.

(6b*R*,9a*S*)-6b,9a-Dimethyl-8-vinyl-6b,9a-dihydroacenaphtho[1,2-*d*][1,3,2]dioxaborole (S22):

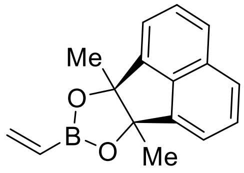

The title compound was prepared according to a known procedure, NMR data is consistent with the literature report.[4]

^{1}H NMR (400 MHz, Chloroform-*d*) δ 7.80 (dd, J = 7.7, 1.3 Hz, 2H), 7.64 – 7.56 (m, 4H), 6.19 – 6.10 (m, 1H), 5.98 (dd, J = 13.8, 4.2 Hz, 1H), 5.87 – 5.78 (m, 1H), 1.83 (s, 6H); ^{13}C NMR (101 MHz, Chloroform-*d*) δ 144.84, 137.49, 134.87, 131.52, 128.64, 125.45, 119.65, 92.10, 22.25.

(4-Methylenecyclohexyl)benzene (S24):

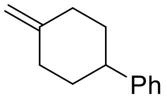

The title compound was prepared according to a known procedure, NMR data is consistent with the literature report.[5]

^{1}H NMR (400 MHz, Chloroform-*d*) δ 7.40 – 7.33 (m, 2H), 7.31 – 7.22 (m, 3H), 4.76 (dd, *J* = 3.6, 1.8 Hz, 2H), 2.75 (tt, *J* = 12.2, 3.1 Hz, 1H), 2.53 – 2.48 (m, 2H), 2.34 – 2.21 (m, 2H), 2.11 – 2.01 (m, 2H), 1.68 – 1.56 (m, 2H); ^{13}C NMR (101 MHz, Chloroform-*d*) δ 148.98, 147.03, 128.53, 127.01, 126.16, 107.55, 44.32, 35.69, 35.33.

(3a*S*,4*R*,6a*S*)-4-((*R*)-2,2-Dimethyl-1,3-dioxolan-4-yl)-2,2-dimethyl-6-methylenetetrahydrofuro[3,4-*d*][1,3]dioxole (S25):

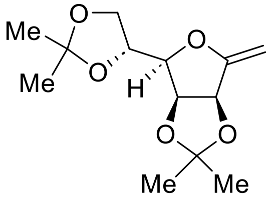

The title compound was prepared according to a known procedure, NMR data is consistent with the literature report.[6]

^{1}H NMR (400 MHz, Chloroform-*d*) δ 5.06 (dt, *J* = 6.0, 1.3 Hz, 1H), 4.77 (dd, *J* = 5.9, 3.9 Hz, 1H), 4.48 (d, *J* = 1.8 Hz, 1H), 4.46 – 4.40 (m, 1H), 4.26 (dd, *J* = 2.0, 1.1 Hz, 1H), 4.13 (dd, *J* = 8.8, 6.1 Hz, 1H), 4.07 (dd, *J* = 8.8, 4.7 Hz, 1H), 4.04 (dd, *J* = 7.5, 3.9 Hz, 1H), 1.49 (s, 3H), 1.45 (s, 3H), 1.38 (s, 6H); ^{13}C NMR (101 MHz, Chloroform-*d*) δ 161.62, 113.62, 109.44, 86.59, 82.41, 80.13, 78.62, 73.37, 66.67, 27.01, 26.89, 25.88, 25.35.

(*E*)-1-(Benzyloxy)-4-(2-bromovinyl)-2-methoxybenzene (S26):

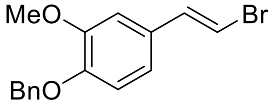

The title compound was prepared according to a known procedure, NMR data is consistent with the literature report.[7]

^{1}H NMR (400 MHz, Chloroform-*d*) δ 7.43 (d, *J* = 6.7 Hz, 2H), 7.40 – 7.35 (m, 2H), 7.33 – 7.28

(m, 1H), 7.02 (d, J = 13.9 Hz, 1H), 6.85 – 6.76 (m, 3H), 6.62 (d, J = 13.9 Hz, 1H), 5.16 (s, 2H), 3.90 (s, 3H); ^{13}C NMR (101 MHz, Chloroform-*d*) δ 149.94, 148.62, 137.02, 136.95, 129.66, 128.75, 128.09, 127.40, 119.42, 114.05, 109.32, 104.63, 71.13, 56.18.

2-(Phenethyldisulfaneyl)pyridine (S30):

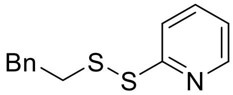

The title compound was prepared according to a known procedure, NMR data is consistent with the literature report.[8]

^{1}H NMR (400 MHz, Chloroform-*d*) δ 8.50 (dt, J = 4.8, 1.3 Hz, 1H), 7.70 (dd, J = 8.1, 1.2 Hz, 1H), 7.64 (td, J = 7.7, 1.8 Hz, 1H), 7.34 – 7.28 (m, 2H), 7.27 – 7.19 (m, 3H), 7.10 (ddd, J = 7.2, 4.8, 1.2 Hz, 1H), 3.09 – 3.02 (m, 4H); ^{13}C NMR (101 MHz, Chloroform-*d*) δ 160.44, 149.76, 139.76, 137.12, 128.75, 128.65, 126.62, 120.72, 119.74, 40.13, 35.45.

2-((4-Methoxybenzyl)disulfaneyl)pyridine (S31):

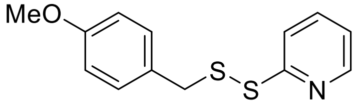

The title compound was prepared according to a known procedure.[8]

^{1}H NMR (400 MHz, Chloroform-*d*) δ 8.42 (dt, J = 4.8, 1.4 Hz, 1H), 7.56 – 7.49 (m, 2H), 7.24 – 7.19 (m, 2H), 7.06 – 6.99 (m, 1H), 6.79 – 6.75 (m, 2H), 3.98 (s, 2H), 3.75 (s, 3H); ^{13}C NMR (101 MHz, Chloroform-*d*) δ 160.37, 159.29, 149.59, 136.89, 130.70, 128.60, 120.54, 119.67, 114.12, 55.42, 43.28.

2-(Cyclopentyldisulfaneyl)pyridine (S32):

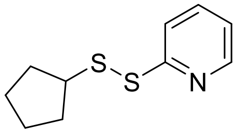

The title compound was prepared according to a known procedure, NMR data is consistent with the literature report.[9]

^{1}H NMR (400 MHz, Chloroform-*d*) δ 8.43 – 8.41 (m, 1H), 7.75 – 7.72 (m, 1H), 7.65 – 7.57 (m, 1H), 7.04 – 7.02 (m, 1H), 3.41 – 3.32 (m, 1H), 1.97 – 1.90 (m, 2H), 1.76 – 1.65 (m, 4H), 1.60 –

1.52 (m, 2H); ^{13}C NMR (101 MHz, Chloroform-*d*) δ 161.16, 149.49, 137.04, 120.52, 119.65, 50.37, 32.91, 24.78.

2-(decyldisulfaneyl)pyridine (S33):

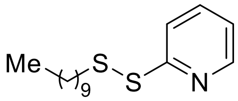

The title compound was prepared according to a known procedure, NMR data is consistent with the literature report.[9]

^{1}H NMR (400 MHz, Chloroform-*d*) δ 8.46 – 8.40 (m, 1H), 7.73 – 7.70 (m, 1H), 7.65 – 7.59 (m, 1H), 7.07– 7.03 (m, 1H), 2.80 – 2.74 (m, 2H), 1.71– 1.62 (m, 2H), 1.39 –1.32 (m, 2H), 1.27– 1.18 (m, 12H), 0.88 – 0.83 (m, 3H); ^{13}C NMR (101 MHz, Chloroform-*d*) δ 160.87, 149.63, 137.10, 137.08, 120.57, 119.68, 119.66, 39.18, 32.01, 29.66, 29.60, 29.42, 29.31, 29.07, 28.62, 22.81, 14.25.

(2*R*,3*R*,4*S*,5*R*,6*S*)-2-(Acetoxymethyl)-6-(pyridin-2-yldisulfaneyl)tetrahydro-2*H*-pyran-3,4,5-triyl triacetate (S34):

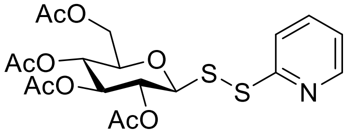

The title compound was prepared according to a known procedure, NMR data is consistent with the literature report.[10]

^{1}H NMR (400 MHz, Chloroform-*d*) δ 8.43 (ddd, *J* = 4.9, 1.9, 0.9 Hz, 1H), 7.87 (dt, *J* = 8.2, 1.0 Hz, 1H), 7.65 (ddd, *J* = 8.2, 7.5, 1.8 Hz, 1H), 7.13 (ddd, *J* = 7.5, 4.9, 1.1 Hz, 1H), 5.28 – 5.19 (m, 2H), 5.06 (ddd, *J* = 9.6, 6.8, 2.7 Hz, 1H), 4.73 – 4.65 (m, 1H), 4.04 (d, *J* = 3.7 Hz, 2H), 3.70 (dt, *J* = 10.1, 3.6 Hz, 1H), 2.08 (s, 3H), 2.01 (s, 6H), 1.91 (s, 3H); ^{13}C NMR (101 MHz, Chloroform-*d*) δ 165.29, 165.06, 164.20, 164.09, 154.86, 143.71, 131.90, 115.86, 115.50, 83.03, 70.91, 68.58, 64.34, 62.81, 56.71, 15.58, 15.49, 15.45.

4. Optimization studies and experimental procedures

4.1. Photoinduced reaction set up

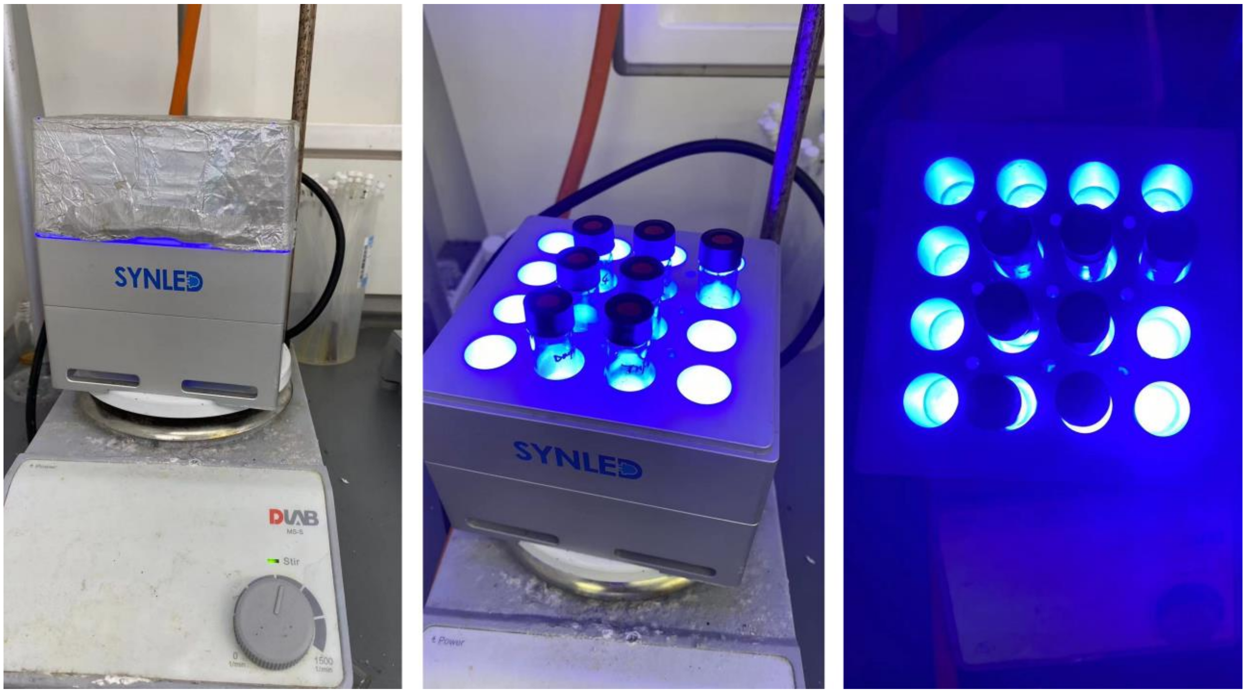

Photoinduced reaction set up: The photoreactor was purchased from Shenzhen Bamboo Bio-tech Co. Ltd. Input: AC100-240 V 50/60 Hz; Output: 12 W; wavelength: 460-470 nm (blue LED).

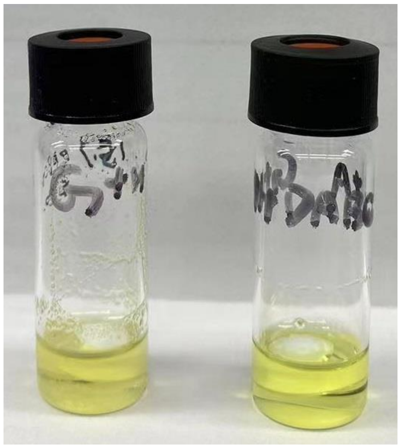

Left vial (at the start of reaction): pale yellow mixture

Right vial (upon reaction completion): bright yellow mixture

4.2. Optimization for anomeric functionalization of native sugars

Table S3. Optimization for the synthesis of *S*-glycosyl donors[a]

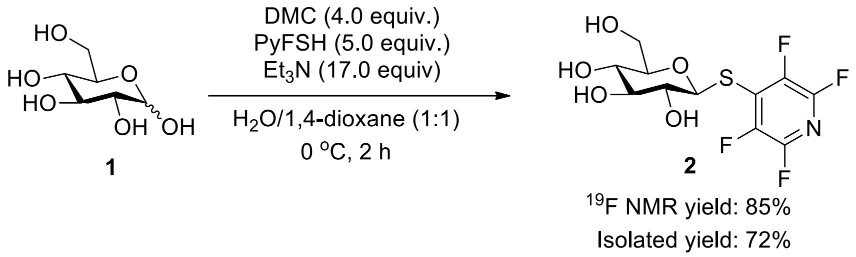

Entry	Variation from the standard conditions	^{19}F NMR yields
1	D_2O/MeCN (1:1) as solvent	63%
2	H_2O/MeCN (1:1) as solvent	70%
3	D_2O/dioxane (1:1) as solvent	75%
4	**3-5** or CDMT/NMM instead of DMC	10%, 5%, <2%, <2%
5	reaction for 24 h	86%

[a]Reaction conditions: **1** (1.0 equiv., 0.2 mmol), DMC (4.0 equiv, 0.8 mmol), PyFSH (5.0 equiv., 0.1 mmol), Et_3N (17.0 equiv, 3.4 mmol), H_2O/1,4-dioxane (0.6 mL/0.6 mL). Yields were determined by ^{19}F NMR with trifluorotoluene as the internal standard. DMC = 2-chloro-1,3-dimethylimidazolinium chloride, PyFSH = 2,3,5,6-tetrafluoropyridine-4-thiol.

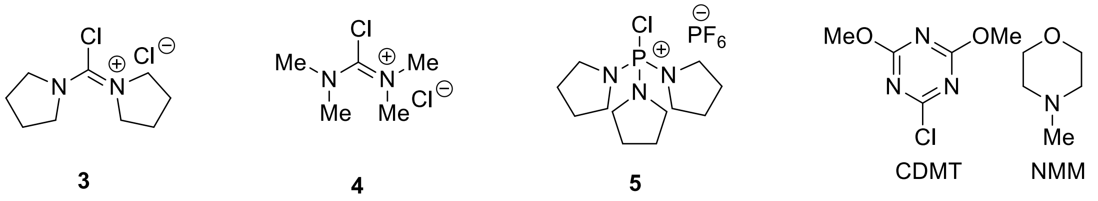

Table S4. Optimization for the photoinduced synthesis of glycosyl compounds[a]

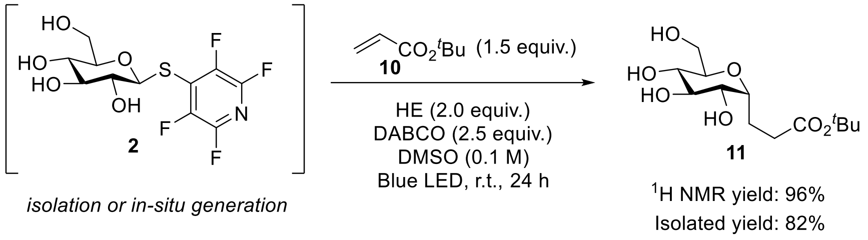

Entry	Variation from the standard conditions	^{1}H NMR yields
1	Et_3N, DIPEA, DBU, BTMG, Quinuclidine as base	76%, 38%, 78%, 15%, 86%
2	DMA, DMF, MeCN, Dioxane as solvent	92%, 94%, 65%, <10%
3	**6**, **7**, **8**, **9** as glycosyl donor	< 10%, < 2%, < 2%, < 2%
4	w/o HE or *hv*	< 2%, < 2%
5	w/o base	< 2%
6	**2** was generated *in situ* used w/o isolation	64% (52%)[b]
7	open to air	42%
8	0.1 mL H_2O added	77%

[a]Reaction conditions: 2 (1.0 equiv., 0.05 mmol), 10 (1.5 equiv., 0.075 mmol), HE (2.0 equiv., 0.1 mmol), DABCO (2.5 equiv., 0.125 mmol), DMSO (0.1 M), 12 W Blue LED, r.t., 24 h. [b]The yield was based on D-glucose as the limting reagent. Yields were determined by ^{1}H NMR with mesitylene as the internal standard, isolated yields in the parentheses. r.t. = room temperature, HE = Hantzsch ester, DABCO = 1,4-diazabicyclo[2.2.2]octane.

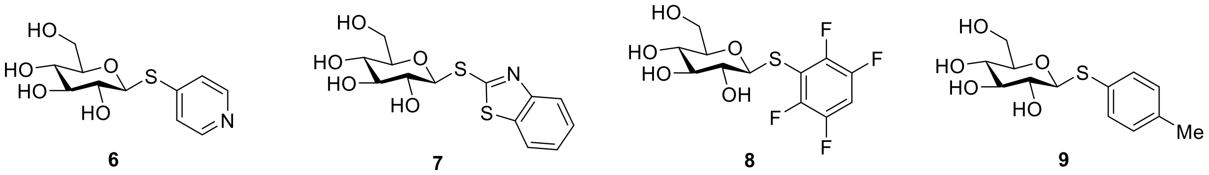

4.3. General procedure for photoinduced glycosylation

General procedure D: using isolated *S*-glycosyl donors

Under air, a 4 mL vial equipped with a magnetic stir bar was added glycosyl donor (1.0 equiv., 0.05 mmol), Hantzsch ester (2.0 equiv., 0.1 mmol), DABCO (2.5 equiv., 0.125 mmol) and radical acceptor (1.5 equiv., 0.075 mmol) (if solid). The reaction vial was then transferred into a glovebox under nitrogen atmosphere, followed by the addition of anhydrous DMSO (0.5 mL) and radical acceptor (1.5 equiv., 0.075 mmol) (if liquid). The reaction vial was sealed and taken out of the glovebox. The mixture was allowed to vigorously stir at room temperature under 12 W blue LED illumination for 24 hours. After the reaction was complete, DMSO was evaporated by attaching the oil pump to the rotary evaporator and the residue was purified by flash silica gel column chromatography to afford the pure product (eluent: $CHCl_3$/MeOH = 15/1 ~ 5/1). (*Note: 2.0 equivalents of glycosyl radical acceptor were used for product* ***15, 19-29****; 3.0 equivalents of*

glycosyl radical acceptor were used for product ***38-39***, ***41***, ***43-44***, ***46-54****; 5.0 equivalents of glycosyl radical acceptor were used for product* ***42*** *and* ***45****; triethylamine was used as the base for products* ***31*** *and* ***35***, *DMA was used as the solvent for products* ***48*** *and* ***49***.)

4.4. General procedure for photoinduced glycosylation (traceless)

General procedure E: without isolating *S*-glycosyl donors

The corresponding *S*-glycosyl donor was generated according to the **General procedure C** and transferred into glovebox after all the volatiles were removed. The residue was re-dissolved in anhydrous DMSO (0.1 M) and the insoluble salts were filtered before the solution was transferred into a 4 mL vial containing glycosyl radical acceptor, Hantzsch ester and DABCO. The reaction vial was sealed and taken out of the glovebox. The mixture was allowed to vigorously stir at room temperature under 12 W blue LED illumination for 24 hours. After the reaction was complete, DMSO was evaporated by attaching the oil pump to the rotary evaporator and the residue was re-dissolved in Methanol-d_4 with mesitylene as the internal standard for subsequent crude ^{1}H NMR analysis and LC-MS analysis.

5. Analytical data of products

***tert*-Butyl 3-((2*R*,3*R*,4*R*,5*S*,6*R*)-3,4,5-trihydroxy-6-(hydroxymethyl)tetrahydro-2*H*-pyran-2-yl)propanoate (11):**

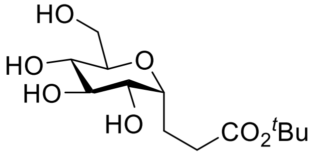

The title compound was prepared according to the **General procedure D** from **2** (1.0 equiv., 0.05 mmol), *tert*-butyl acrylate (1.5 equiv., 0.075 mmol), Hantzsch ester (2.0 equiv., 0.1 mmol), DABCO (2.5 equiv., 0.125 mmol), anhydrous DMSO (0.5 mL). The residue was subjected to flash silica gel column chromatography (eluent: $CHCl_3$/MeOH = 15/1 ~ 5/1) to give the pure product as viscous gel (^{1}H NMR yield: 96%, α:β > 95:5; isolated yield: 12.0 mg, 82%).

Glycosylation *in situ*: the title compound was prepared according to the **General procedure E** with D-glucose (1.0 equiv., 0.2 mmol), *tert*-butyl acrylate (1.5 equiv., 0.3 mmol), Hantzsch ester (2.0 equiv., 0.4 mmol), DABCO (2.5 equiv., 0.5 mmol), anhydrous DMSO (2 mL). The residue was re-dissolved in Methanol-d_4 with mesitylene (0.1 mmol) as the internal standard for ^{1}H NMR analysis (^{1}H NMR yield: 64%, α:β > 95:5; isolated yield: 30.4 mg, 52%; the yield was based on D-glucose as the limiting reagent).

^{1}H NMR (500 MHz, Methanol-d_4) δ **3.88 (ddd, *J* = 10.5, 5.7, 4.3 Hz, 1H, anomeric H)**, 3.78 (dd, *J* = 11.8, 2.5 Hz, 1H), 3.65 – 3.57 (m, 2H), 3.52 (dd, *J* = 9.4, 8.4 Hz, 1H), 3.38 (ddd, *J* = 9.7, 5.8, 2.6 Hz, 1H), 3.24 (dd, *J* = 9.6, 8.4 Hz, 1H), 2.42 (ddd, *J* = 16.0, 8.9, 5.9 Hz, 1H), 2.27 (ddd, *J* = 16.0, 8.7, 7.1 Hz, 1H), 1.99 – 1.87 (m, 2H), 1.45 (s, 9H); ^{13}C NMR (101 MHz, Methanol-d_4) δ 174.92, 81.50, 76.45, 75.17, 74.62, 72.96, 72.28, 63.10, 32.55, 28.35, 21.47; HRMS (ESI) m/z calcd for $C_{13}H_{24}NaO_7$ [(M+Na)$^+$]: 315.1414, found: 315.1418.

$[\alpha]_D^{25}$: +34.5 (c = 1.0, MeOH).

***N*-Phenyl-3-((2*R*,3*R*,4*R*,5*R*,6*R*)-3,4,5-trihydroxy-6-(hydroxymethyl)tetrahydro-2*H*-pyran-2-yl)propenamide (19):**

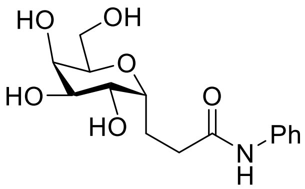

The title compound was prepared according to the **General procedure D** from **S1** (1.0 equiv., 0.05 mmol), *N*-phenylacrylamide (2.0 equiv., 0.1 mmol), Hantzsch ester (2.0 equiv., 0.1 mmol), DABCO (2.5 equiv., 0.125 mmol), anhydrous DMSO (0.5 mL). The residue was subjected to flash silica gel column chromatography (eluent: $CHCl_3$/MeOH = 15/1 ~ 5/1) to give the pure product as viscous gel (^{1}H NMR yield: 55%, α:β > 95:5; isolated yield: 6.1 mg, 39%).

^{1}H NMR (500 MHz, Methanol-d_4) δ 7.57 – 7.53 (m, 2H), 7.31 – 7.27 (m, 2H), 7.10 – 7.06 (m, 1H), **4.00 (ddd, *J* = 10.8, 5.4, 4.1 Hz, 1H, anomeric H)**, 3.94 – 3.90 (m, 2H), 3.80 (dd, *J* = 11.0, 7.1 Hz, 1H), 3.73 (ddd, *J* = 6.9, 4.2, 2.3 Hz, 1H), 3.70 (d, *J* = 3.4 Hz, 1H), 3.68 (dd, *J* = 5.7, 3.7 Hz, 1H), 2.52 (ddd, *J* = 14.4, 8.4, 5.9 Hz, 1H), 2.44 (dt, *J* = 14.5, 7.8 Hz, 1H), 2.09 – 1.98 (m, 2H); ^{13}C NMR (126 MHz, Methanol-d_4) δ 174.56, 139.92, 129.75, 125.10, 121.28, 75.32, 74.07, 71.90, 70.33, 70.25, 62.38, 34.44, 22.62; HRMS (ESI) m/z calcd for $C_{15}H_{21}NNaO_6$ [$(M+Na)^+$]: 334.1261, found: 334.1266.

$[\alpha]_D^{25}$: +56.0 (c = 1.0, MeOH).

***N*-Phenyl-3-((2*R*,3*S*,4*R*,5*S*,6*R*)-3,4,5-trihydroxy-6-(hydroxymethyl)tetrahydro-2*H*-pyran-2-yl)propenamide (20):**

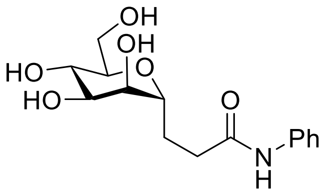

The title compound was prepared according to the **General procedure D** from **S2** (1.0 equiv., 0.05 mmol), *N*-phenylacrylamide (2.0 equiv., 0.1 mmol), Hantzsch ester (2.0 equiv., 0.1 mmol), DABCO (2.5 equiv., 0.125 mmol), anhydrous DMSO (0.5 mL). The residue was subjected to flash silica gel column chromatography (eluent: $CHCl_3$/MeOH = 15/1 ~ 5/1) to give the pure product as viscous gel (^{1}H NMR yield: 62%, α:β > 95:5; isolated yield: 7.2 mg, 46%).

^{1}H NMR (400 MHz, Methanol-d_4) δ 7.58 – 7.53 (m, 2H), 7.33 – 7.26 (m, 2H), 7.08 (tt, *J* = 7.2, 1.2 Hz, 1H), **3.90 (dt, *J* = 11.0, 3.2 Hz, 1H, anomeric H)**, 3.80 – 3.70 (m, 4H), 3.64 (t, *J* = 8.4 Hz, 1H), 3.50 (ddd, *J* = 8.6, 5.5, 3.3 Hz, 1H), 2.50 (h, *J* = 7.2, 6.8 Hz, 2H), 2.12 (dddd, *J* = 13.7, 11.1, 6.2, 3.7 Hz, 1H), 1.92 – 1.83 (m, 1H); ^{13}C NMR (126 MHz, Methanol-d_4) δ 174.04, 139.88, 129.75, 125.13, 121.25, 77.69, 76.10, 72.87, 72.75, 69.37, 62.81, 34.17, 25.82; HRMS (ESI) m/z calcd for $C_{15}H_{21}NNaO_6$ [$(M+Na)^+$]: 334.1261, found: 334.1270.

[α]$_D^{25}$: +23.3 (c = 1.0, MeOH).

***tert*-Butyl 3-((2*S*,3*R*,4*R*,5*R*,6*S*)-3,4,5-trihydroxy-6-methyltetrahydro-2*H*-pyran-2-yl) propanoate (21):**

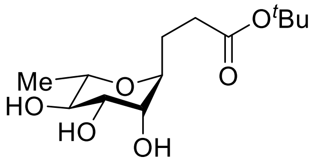

The title compound was prepared according to the **General procedure D** from **S3** (1.0 equiv., 0.05 mmol), *tert*-butyl acrylate (2.0 equiv., 0.1 mmol), Hantzsch ester (2.0 equiv., 0.1 mmol), DABCO (2.5 equiv., 0.125 mmol), anhydrous DMSO (0.5 mL). The residue was subjected to flash silica gel column chromatography (eluent: $CHCl_3$/MeOH = 15/1 ~ 5/1) to give the pure product as viscous gel (^{1}H NMR yield: 93%, α:β > 95:5; isolated yield: 10.4 mg, 75%).

Glycosylation *in situ*: the title compound was prepared according to the **General procedure E** with L-rhamnose (1.0 equiv., 0.2 mmol), *tert*-butyl acrylate (2.0 equiv., 0.4 mmol), Hantzsch ester (2.0 equiv., 0.4 mmol), DABCO (2.5 equiv., 0.5 mmol), anhydrous DMSO (2 mL). The residue was re-dissolved in Methanol-d_4 with mesitylene (0.1 mmol) as the internal standard for ^{1}H NMR analysis (^{1}H NMR yield: 82%, α:β > 95:5; the yield was based on **S3** as the limiting reagent).

^{1}H NMR (400 MHz, Methanol-d_4) δ **3.79 (ddd, *J* = 10.9, 4.1, 2.5 Hz, 1H, anomeric H)**, 3.74 (t, *J* = 3.0 Hz, 1H), 3.64 (dd, *J* = 8.5, 3.4 Hz, 1H), 3.48 (dq, *J* = 8.5, 6.0 Hz, 1H), 3.40 (t, *J* = 8.5 Hz, 1H), 2.37 – 2.26 (m, 2H), 1.99 (dddd, *J* = 13.9, 10.9, 7.4, 6.2 Hz, 1H), 1.73 (dtd, *J* = 14.4, 7.8, 4.1 Hz, 1H), 1.45 (s, 9H), 1.26 (d, *J* = 6.1 Hz, 3H); ^{13}C NMR (101 MHz, Methanol-d_4) δ 174.41, 81.59, 77.84, 74.34, 73.01, 72.62, 71.08, 32.72, 28.34, 25.08, 18.30; HRMS (ESI) m/z calcd for $C_{13}H_{24}NaO_6$ [(M+Na)$^+$]: 299.1465, found: 299.1470.

[α]$_D^{25}$: -24.3 (c = 1.0, MeOH).

***N*-Phenyl-3-((2*R*,3*R*,4*S*,5*S*,6*R*)-3,4,5-trihydroxy-6-(hydroxymethyl)tetrahydro-2H-pyran-2-yl)propenamide (22):**

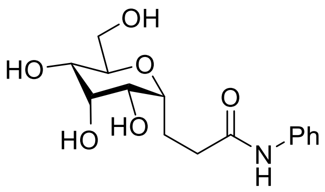

The title compound was prepared according to the **General procedure D** from **S4** (1.0 equiv., 0.05 mmol), *N*-phenylacrylamide (2.0 equiv., 0.1 mmol), Hantzsch ester (2.0 equiv., 0.1 mmol), DABCO (2.5 equiv., 0.125 mmol), anhydrous DMSO (0.5 mL). The residue was subjected to flash silica gel column chromatography (eluent: $CHCl_3$/MeOH = 15/1 ~ 5/1) to give the pure product as viscous gel (^{1}H NMR yield: 64%, α:β > 95:5; isolated yield: 7.8 mg, 50%).

^{1}H NMR (500 MHz, Methanol-d_4) δ 7.58 – 7.53 (m, 2H), 7.32 – 7.26 (m, 2H), 7.08 (tt, *J* = 7.4, 1.2 Hz, 1H), 3.89 (dt, *J* = 7.0, 4.7 Hz, 1H), 3.84 (t, *J* = 3.3 Hz, 1H), **3.78 – 3.69 (m, 4H, one of them is anomeric H)**, 3.69 – 3.67 (m, 1H), 2.56 – 2.45 (m, 2H), 2.28 (dddd, *J* = 14.0, 10.1, 7.6, 6.2 Hz, 1H), 1.95 (dtd, *J* = 14.3, 7.9, 3.7 Hz, 1H); ^{13}C NMR (126 MHz, Methanol-d_4) δ 174.71, 139.90, 129.74, 125.11, 121.28, 77.20, 74.44, 71.75, 69.85, 69.83, 61.42, 34.52, 26.88; HRMS (ESI) m/z calcd for $C_{15}H_{21}NNaO_6$ [(M+Na)$^+$]: 334.1261, found: 334.1264.

$[\alpha]_D^{25}$: -29.0 (c = 1.0, MeOH).

Benzyl 3-((2*R*,3*S*,4*S*,5*R*)-3,4,5-trihydroxytetrahydro-2*H*-pyran-2-yl)propanoate (23):

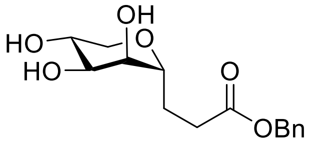

The title compound was prepared according to the **General procedure D** from **S5** (1.0 equiv., 0.05 mmol), benzyl acrylate (2.0 equiv., 0.1 mmol), Hantzsch ester (2.0 equiv., 0.1 mmol), DABCO (2.5 equiv., 0.125 mmol), anhydrous DMSO (0.5 mL). The residue was subjected to flash silica gel column chromatography (eluent: $CHCl_3$/MeOH = 15/1 ~ 5/1) to give the pure product as viscous gel (^{1}H NMR yield: 58%, α:β > 95:5; isolated yield: 5.9 mg, 40%).

^{1}H NMR (500 MHz, Methanol-d_4) δ 7.39 – 7.27 (m, 5H), 5.11 (s, 2H), 3.86 – 3.83 (m, 1H), 3.75 (dd, *J* = 12.2, 1.6 Hz, 1H), 3.66 (dt, *J* = 3.6, 1.7 Hz, 1H), 3.59 (dt, *J* = 12.3, 1.4 Hz, 1H), 3.53 (dd, *J* = 9.6, 3.2 Hz, 1H), **3.46 (td, *J* = 9.4, 2.8 Hz, 1H, anomeric H)**, 2.55 (ddd, *J* = 16.0, 9.2, 5.8 Hz, 1H), 2.46 (ddd, *J* = 15.9, 8.8, 6.7 Hz, 1H), 2.16 (dddd, *J* = 14.1, 9.3, 6.7, 2.8 Hz, 1H), 1.75 – 1.66 (m, 1H); ^{13}C NMR (126 MHz, Methanol-d_4) δ 175.43, 137.75, 129.51, 129.14, 129.12, 76.00,

71.71, 71.61, 69.95, 67.92, 67.18, 31.31, 28.41; HRMS (ESI) m/z calcd for $C_{15}H_{21}O_6$ [(M+H)$^+$]: 297.1333, found: 297.1336.
[α]$_D^{25}$: -56.7 (c = 1.0, MeOH).

***tert*-Butyl 3-((2*S*,3*R*,4*R*,5*R*)-3,4-dihydroxy-5-(hydroxymethyl)tetrahydrofuran-2-yl) propanoate (24):**

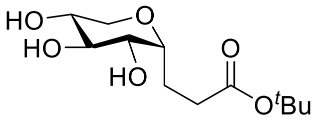

The title compound was prepared according to the **General procedure D** from **S6** (1.0 equiv., 0.05 mmol), *tert*-butyl acrylate (2.0 equiv., 0.1 mmol), Hantzsch ester (2.0 equiv., 0.1 mmol), DABCO (2.5 equiv., 0.125 mmol), anhydrous DMSO (0.5 mL). The residue was subjected to flash silica gel column chromatography (eluent: $CHCl_3$/MeOH = 15/1 ~ 5/1) to give the pure product as viscous gel (^{1}H NMR yield: 88%, α:β = 91:9; isolated yield: 9.6 mg, 73%).

Glycosylation *in situ*: the title compound was prepared according to the **General procedure E** with D-xylose (1.0 equiv., 0.2 mmol), *tert*-butyl acrylate (2.0 equiv., 0.4 mmol), Hantzsch ester (2.0 equiv., 0.4 mmol), DABCO (2.5 equiv., 0.5 mmol), anhydrous DMSO (2 mL). The residue was re-dissolved in Methanol-*d₄* with mesitylene (0.1 mmol) as the internal standard for ^{1}H NMR analysis (^{1}H NMR yield: 75%, α:β = 91:9; the yield was based on **S6** as the limiting reagent).

^{1}H NMR (400 MHz, Methanol-d_4) δ 3.86 – 3.79 (m, 2H), 3.72 (dt, *J* = 12.6, 1.6 Hz, 1H), **3.67 (ddd, *J* = 9.5, 4.4, 1.7 Hz, 1H, anomeric H)**, 3.51 (dq, *J* = 3.8, 1.8 Hz, 1H), 3.43 (dt, *J* = 3.3, 1.5 Hz, 1H), 2.40 – 2.26 (m, 2H), 1.97 (dddd, *J* = 14.2, 9.5, 7.7, 6.6 Hz, 1H), 1.77 – 1.66 (m, 1H), 1.45 (s, 9H); ^{13}C NMR (101 MHz, Methanol-d_4) δ 174.93, 81.51, 75.70, 72.01, 70.31, 70.11, 69.03, 32.73, 28.34, 27.13; HRMS (ESI) m/z calcd for $C_{12}H_{22}NaO_6$ [(M+Na)$^+$]: 285.1309, found: 285.1315.
[α]$_D^{25}$: -28.4 (c = 1.0, MeOH).

Benzyl 3-((2*S*,3*S*,4*S*,5*R*,6*S*)-3,4,5-trihydroxy-6-(hydroxymethyl)tetrahydro-2*H*-pyran-2-yl) propanoate (25):

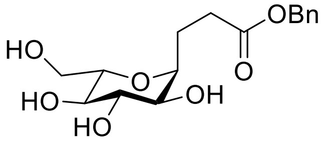

The title compound was prepared according to the **General procedure D** from **S7** (1.0 equiv., 0.05 mmol), benzyl acrylate (2.0 equiv., 0.1 mmol), Hantzsch ester (2.0 equiv., 0.1 mmol), DABCO (2.5 equiv., 0.125 mmol), anhydrous DMSO (0.5 mL). The residue was subjected to flash silica gel column chromatography (eluent: $CHCl_3$/MeOH = 15/1 ~ 5/1) to give the pure product as viscous gel (^{1}H NMR yield: 82%, α:β > 95:5; isolated yield: 10.6 mg, 65%).

^{1}H NMR (500 MHz, Methanol-d_4) δ 7.38 – 7.28 (m, 5H), 5.12 (s, 2H), **3.91 (dt, *J* = 10.5, 5.6 Hz, 1H, anomeric H)**, 3.75 (dd, *J* = 11.8, 2.5 Hz, 1H), 3.64 – 3.57 (m, 2H), 3.52 (dd, *J* = 9.5, 8.5 Hz, 1H), 3.39 (ddd, *J* = 9.6, 5.8, 2.5 Hz, 1H), 3.24 (dd, *J* = 9.6, 8.5 Hz, 1H), 2.59 – 2.51 (m, 1H), 2.47 – 2.40 (m, 1H), 2.05 – 1.95 (m, 2H); ^{13}C NMR (126 MHz, Methanol-d_4) δ 175.14, 137.66, 129.53, 129.21, 129.16, 76.53, 75.14, 74.62, 72.91, 72.24, 67.28, 63.06, 31.36, 21.35; HRMS (ESI) m/z calcd for $C_{16}H_{22}NaO_7$ [(M+Na)$^+$]: 349.1258, found: 349.1260.

$[\alpha]_D^{25}$: -92.4 (c = 1.0, MeOH).

Benzyl 3-((2*R*,3*R*,4*R*,5*S*,6*R*)-3,4-dihydroxy-6-(hydroxymethyl)-5-(((2*S*,3*R*,4*S*,5*S*,6*R*)-3,4,5-trihydroxy-6-(hydroxymethyl)tetrahydro-2*H*-pyran-2-yl)oxy)tetrahydro-2*H*-pyran-2-yl)propanoate (15):

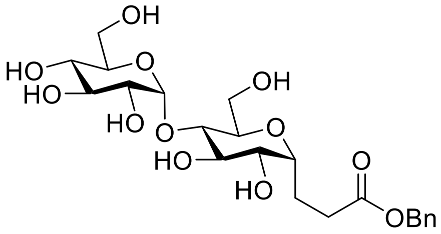

The title compound was prepared according to the **General procedure D** from **13** (1.0 equiv., 0.05 mmol), benzyl acrylate (2.0 equiv., 0.1 mmol), Hantzsch ester (2.0 equiv., 0.1 mmol), DABCO (2.5 equiv., 0.125 mmol), anhydrous DMSO (0.5 mL). The residue was subjected to flash silica gel column chromatography (eluent: $CHCl_3$/MeOH = 15/1 ~ 3/1) to give the pure product as viscous gel (^{1}H NMR yield: 80%, α:β > 95:5; isolated yield: 16.1 mg, 66%).

^{1}H NMR (500 MHz, Methanol-d_4) δ 7.39 – 7.28 (m, 5H), 5.14 (d, *J* = 3.8 Hz, 1H), 5.12 (s, 2H), **3.91 (td, *J* = 7.4, 5.7 Hz, 1H, anomeric H)**, 3.83 – 3.74 (m, 4H), 3.68 – 3.61 (m, 4H), 3.50 (dd, *J* = 4.7, 2.2 Hz, 2H), 3.44 (dd, *J* = 9.7, 3.8 Hz, 1H), 3.27 (t, *J* = 9.3 Hz, 1H), 2.53 (dt, *J* = 16.3, 7.3 Hz, 1H), 2.48 – 2.41 (m, 1H), 2.00 (q, *J* = 7.6 Hz, 2H); ^{13}C NMR (126 MHz, Methanol-d_4) δ 175.12, 137.64, 129.55, 129.22, 129.19, 102.48, 81.30, 76.17, 75.06, 74.69, 74.50, 74.14, 73.56, 72.47, 71.53, 67.30, 62.69, 62.36, 31.44, 21.58; HRMS (ESI) m/z calcd for $C_{22}H_{32}NaO_{12}$ [(M+Na)$^+$]: 511.1786, found: 511.1793.
$[\alpha]_D^{25}$: +113.3 (c = 1.0, MeOH).

Benzyl 3-((2*R*,3*R*,4*R*,5*S*,6*R*)-3,4,5-trihydroxy-6-((((2*S*,3*R*,4*S*,5*R*,6*R*)-3,4,5-trihydroxy-6-(hydroxymethyl)tetrahydro-2*H*-pyran-2-yl)oxy)methyl)tetrahydro-2*H*-pyran-2-yl)propanoate (26):

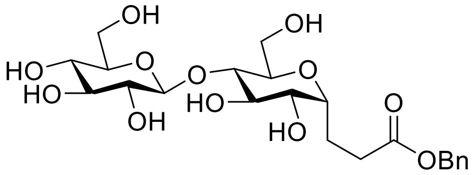

The title compound was prepared according to the **General procedure D** from **S8** (1.0 equiv., 0.05 mmol), benzyl acrylate (2.0 equiv., 0.1 mmol), Hantzsch ester (2.0 equiv., 0.1 mmol), DABCO (2.5 equiv., 0.125 mmol), anhydrous DMSO (0.5 mL). The residue was subjected to flash silica gel column chromatography (eluent: $CHCl_3$/MeOH = 15/1 ~ 3/1) to give the pure product as viscous gel (^{1}H NMR yield: 82%, α:β > 95:5; isolated yield: 17.3 mg, 71%).

Glycosylation *in situ*: the title compound was prepared according to the **General procedure E** with cellobiose (1.0 equiv., 0.2 mmol), benzyl acrylate (2.0 equiv., 0.4 mmol), Hantzsch ester (2.0 equiv., 0.4 mmol), DABCO (2.5 equiv., 0.5 mmol), anhydrous DMSO (2 mL). The residue was re-dissolved in Methanol-d_4 with mesitylene (0.1 mmol) as the internal standard for ^{1}H NMR analysis (^{1}H NMR yield: 68%, α:β > 95:5; the yield was based on **S8** as the limiting reagent).

^{1}H NMR (500 MHz, Methanol-d_4) δ 7.41 – 7.26 (m, 5H), 5.12 (s, 2H), 4.38 (d, *J* = 7.8 Hz, 1H), 3.89 (ddd, *J* = 11.9, 7.5, 3.3 Hz, 2H), 3.83 – 3.72 (m, 2H), 3.66 (hept, *J* = 5.5 Hz, 3H), 3.51 (q, *J* = 3.8 Hz, 2H), 3.40 – 3.32 (m, 3H), 3.22 (dd, *J* = 9.1, 7.8 Hz, 1H), 2.52 (dt, *J* = 16.3, 7.3 Hz, 1H), 2.44 (dt, *J* = 16.0, 7.5 Hz, 1H), 2.00 (dt, *J* = 9.4, 7.2 Hz, 2H); ^{13}C NMR (126 MHz, Methanol-d_4)

δ 175.07, 137.63, 129.54, 129.21, 129.18, 104.57, 81.35, 78.08, 77.79, 76.33, 74.92, 73.50, 72.98, 72.63, 71.39, 67.30, 62.42, 62.13, 31.42, 21.27; HRMS (ESI) m/z calcd for $C_{22}H_{32}NaO_{12}$ $[(M+Na)^+]$: 511.1786, found: 511.1791.
$[\alpha]_D^{25}$: +48.5 (c = 1.0, MeOH).

Benzyl 3-((2*R*,3*R*,4*R*,5*S*,6*R*)-3,4-dihydroxy-6-(hydroxymethyl)-5-(((2*R*,3*R*,4*S*,5*S*,6*R*)-3,4,5-trihydroxy-6-(hydroxymethyl)tetrahydro-2*H*-pyran-2-yl)oxy)tetrahydro-2*H*-pyran-2-yl)propanoate (27):

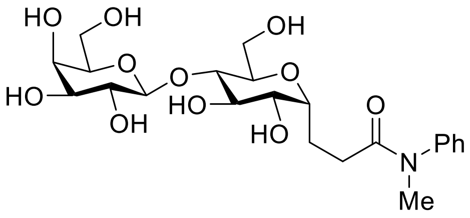

The title compound was prepared according to the **General procedure D** from **S9** (1.0 equiv., 0.05 mmol), *N*-methyl-*N*-phenylacrylamide (2.0 equiv., 0.1 mmol), Hantzsch ester (2.0 equiv., 0.1 mmol), DABCO (2.5 equiv., 0.125 mmol), anhydrous DMSO (0.5 mL). The residue was subjected to flash silica gel column chromatography (eluent: $CHCl_3$/MeOH = 15/1 ~ 3/1) to give the pure product as viscous gel (^1H NMR yield: 65%, α:β > 95:5; isolated yield: 11.0 mg, 45%).
^1H NMR (500 MHz, Methanol-d_4) δ 7.48 (t, *J* = 7.6 Hz, 2H), 7.40 (t, *J* = 7.4 Hz, 1H), 7.34 – 7.29 (m, 2H), 4.32 (d, *J* = 7.6 Hz, 1H), 3.82 (dd, *J* = 3.3, 1.0 Hz, 1H), **3.80 – 3.69 (m, 4H, one of them is anomeric H)**, 3.64 – 3.51 (m, 5H), 3.49 (td, *J* = 6.8, 3.3 Hz, 2H), 3.30 – 3.26 (m, 1H), 3.25 (s, 3H), 2.26 (dt, *J* = 14.9, 7.3 Hz, 1H), 2.09 (dt, *J* = 15.2, 7.5 Hz, 1H), 1.91 (q, *J* = 7.2 Hz, 2H); ^{13}C NMR (126 MHz, Methanol-d_4) δ 175.20, 145.26, 131.08, 129.31, 128.52, 105.03, 80.99, 77.04, 76.57, 74.78, 73.50, 72.59, 72.53, 70.32, 62.52, 61.82, 37.82, 31.31, 21.78; HRMS (ESI) m/z calcd for $C_{22}H_{33}NNaO_{11}$ $[(M+Na)^+]$: 510.1946, found: 510.1949.
$[\alpha]_D^{25}$: +56.2 (c = 1.0, MeOH).

3-((2*R*,3*R*,4*R*,5*S*,6*R*)-3,4-Dihydroxy-6-(hydroxymethyl)-5-(((2*S*,3*R*,4*S*,5*R*,6*R*)-3,4,5-trihydroxy-6-(hydroxymethyl)tetrahydro-2*H*-pyran-2-yl)oxy)tetrahydro-2*H*-pyran-2-yl)-*N*-methyl-*N*-phenylpropanamide (28):

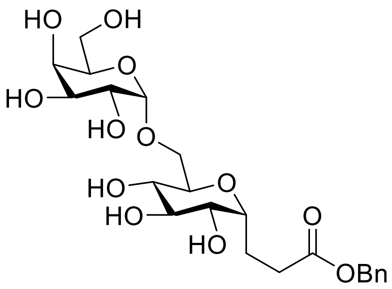

The title compound was prepared according to the **General procedure D** from **S10** (1.0 equiv., 0.05 mmol), benzyl acrylate (2.0 equiv., 0.1 mmol), Hantzsch ester (2.0 equiv., 0.1 mmol), DABCO (2.5 equiv., 0.125 mmol), anhydrous DMSO (0.5 mL). The residue was subjected to flash silica gel column chromatography (eluent: $CHCl_3$/MeOH = 15/1 ~ 3/1) to give the pure product as viscous gel (^{1}H NMR yield: 83%, α:β > 95:5; isolated yield: 17.1 mg, 70%).

Glycosylation *in situ*: the title compound was prepared according to the **General procedure E** with melibiose (1.0 equiv., 0.2 mmol), benzyl acrylate (2.0 equiv., 0.4 mmol), Hantzsch ester (2.0 equiv., 0.4 mmol), DABCO (2.5 equiv., 0.5 mmol), anhydrous DMSO (2 mL). The residue was re-dissolved in Methanol-d_4 with mesitylene (0.1 mmol) as the internal standard for ^{1}H NMR analysis (^{1}H NMR yield: 64%, α:β > 95:5; the yield was based on **S10** as the limiting reagent).

^{1}H NMR (500 MHz, Methanol-d_4) δ 7.38 – 7.29 (m, 5H), 5.13 (s, 2H), 3.97 – 3.90 (m, 2H), 3.90 – 3.86 (m, 2H), 3.77 – 3.75 (m, 2H), 3.71 – 3.68 (m, 2H), 3.64 – 3.57 (m, 3H), 3.56 – 3.51 (m, 1H), 3.35 (s, 2H), 2.58 – 2.44 (m, 2H), 2.08 – 1.97 (m, 2H); ^{13}C NMR (126 MHz, Methanol-d_4) δ 175.04, 137.64, 129.57, 129.19, 129.17, 100.02, 76.83, 75.13, 73.12, 72.77, 72.22, 72.04, 71.61, 71.07, 70.38, 67.67, 67.31, 62.68, 31.34, 21.22; HRMS (ESI) m/z calcd for $C_{22}H_{32}NaO_{12}$ [(M+Na)$^+$]: 511.1786, found: 511.1789.

$[\alpha]_D^{25}$: +97.4 (c = 1.0, MeOH).

Benzyl 3-((2*R*,3*R*,4*R*,5*S*,6*R*)-5-(((2*R*,3*R*,4*R*,5*S*,6*R*)-3,4-dihydroxy-6-(hydroxymethyl)-5-(((2*R*,3*R*,4*S*,5*S*,6*R*)-3,4,5-trihydroxy-6-(hydroxymethyl)tetrahydro-2*H*-pyran-2-yl)oxy)tetrahydro-2*H*-pyran-2-yl)oxy)-3,4-dihydroxy-6-(hydroxymethyl)tetrahydro-2*H*-pyran-2-yl)propanoate (29):

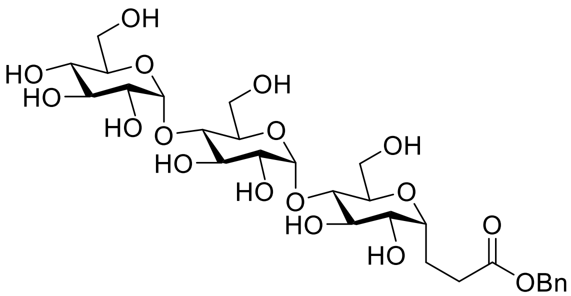

The title compound was prepared according to the **General procedure D** from **S11** (1.0 equiv., 0.05 mmol), benzyl acrylate (2.0 equiv., 0.1 mmol), Hantzsch ester (2.0 equiv., 0.1 mmol), DABCO (2.5 equiv., 0.125 mmol), anhydrous DMSO (0.5 mL). The residue was subjected to flash silica gel column chromatography (eluent: $CHCl_3$/MeOH = 15/1 ~ 2/1) to give the pure product as viscous gel (^{1}H NMR yield: 74%, α:β > 95:5; isolated yield: 21.0 mg, 65%).

Glycosylation *in situ*: the title compound was prepared according to the **General procedure E** with maltotriose (1.0 equiv., 0.2 mmol), benzyl acrylate (2.0 equiv., 0.4 mmol), Hantzsch ester (2.0 equiv., 0.4 mmol), DABCO (2.5 equiv., 0.5 mmol), anhydrous DMSO (2 mL). The residue was re-dissolved in Methanol-*d₄* with mesitylene (0.1 mmol) as the internal standard for ^{1}H NMR analysis (^{1}H NMR yield: 58%, α:β > 95:5; the yield was based on **S11** as the limiting reagent).

^{1}H NMR (500 MHz, Methanol-d_4) δ 7.40 – 7.31 (m, 5H), 5.18 (d, *J* = 3.8 Hz, 1H), 5.16 (d, *J* = 3.8 Hz, 1H), 5.14 (s, 2H), 3.96 – 3.85 (m, 3H), 3.85 – 3.80 (m, 3H), 3.77 – 3.70 (m, 4H), 3.70 – 3.62 (m, 3H), 3.56 – 3.51 (m, 4H), 3.48 (dd, *J* = 9.7, 3.8 Hz, 1H), 3.30 (d, *J* = 9.2 Hz, 1H), 2.57 – 2.44 (m, 2H), 2.03 (dt, *J* = 13.3, 7.6 Hz, 2H); ^{13}C NMR (126 MHz, Methanol-d_4) δ 175.15, 137.61, 129.55, 129.21, 102.90, 102.18, 81.33, 81.11, 76.06, 75.04, 74.91, 74.77, 74.26, 74.20, 73.68, 73.23, 72.38, 71.55, 67.32, 62.69, 62.30, 62.02, 31.47, 21.66; HRMS (ESI) m/z calcd for $C_{28}H_{42}NaO_{17}$ [(M+Na)$^+$]: 673.2314, found: 673.2319.

$[\alpha]_D^{25}$: +74.5 (c = 1.0, MeOH).

(5*S*,8*R*,9*S*,10*S*,13*S*,17*S*)-10,13-Dimethyl-3-oxohexadecahydro-1*H*-cyclopenta[*a*]phenanthren-17-yl 3-((2*R*,3*R*,4*R*,5*S*,6*R*)-3,4,5-trihydroxy-6-(hydroxymethyl)tetrahydro-2*H*-pyran-2-yl)propanoate (30):

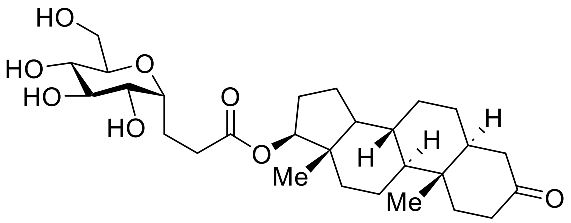

The title compound was prepared according to the **General procedure D** from **2** (1.0 equiv., 0.05 mmol), **S12** (1.5 equiv., 0.075 mmol), Hantzsch ester (2.0 equiv., 0.1 mmol), DABCO (2.5 equiv., 0.125 mmol), anhydrous DMSO (0.5 mL). The residue was subjected to flash silica gel column chromatography (eluent: $CHCl_3$/MeOH = 15/1 ~ 5/1) to give the pure product as viscous gel (^{1}H NMR yield: 81%, α:β > 95:5; isolated yield: 18.3 mg, 72%).

^{1}H NMR (500 MHz, Methanol-d_4) δ 4.61 (dd, *J* = 9.2, 7.8 Hz, 1H), **3.90 (dt, *J* = 10.5, 5.3 Hz, 1H, anomeric H)**, 3.77 (dd, *J* = 11.8, 2.5 Hz, 1H), 3.67 – 3.57 (m, 2H), 3.53 (t, *J* = 8.9 Hz, 1H), 3.40 (ddd, *J* = 8.6, 5.7, 2.5 Hz, 1H), 3.26 (t, *J* = 8.9 Hz, 1H), 2.54 – 2.43 (m, 2H), 2.43 – 2.31 (m, 2H), 2.27 – 2.19 (m, 1H), 2.19 – 2.10 (m, 1H), 2.10 – 1.89 (m, 4H), 1.80 – 1.71 (m, 2H), 1.70 – 1.60 (m, 2H), 1.58 – 1.50 (m, 3H), 1.44 – 1.30 (m, 5H), 1.23 – 1.09 (m, 2H), 1.07 (s, 3H), 1.01 – 0.93 (m, 1H), 0.84 (d, *J* = 12.2 Hz, 3H), 0.82 (s, 1H); ^{13}C NMR (126 MHz, Methanol-d_4) δ 214.78, 175.48, 84.21, 76.42, 75.09, 74.66, 72.88, 72.20, 63.02, 55.15, 51.91, 48.11, 45.44, 43.93, 39.77, 38.89, 38.17, 36.88, 36.51, 32.43, 31.50, 29.91, 28.55, 24.51, 22.06, 21.50, 12.60, 11.71; HRMS (ESI) m/z calcd for $C_{28}H_{44}NaO_8$ [$(M+Na)^+$]: 531.2928, found: 531.2930.

$[\alpha]_D^{25}$: +41.8 (c = 1.0, MeOH).

(8*R*,9*S*,13*S*,14*S*)-13-Methyl-17-oxo-7,8,9,11,12,13,14,15,16,17-decahydro-6*H*-cyclopenta[*a*]phenanthren-3-yl 3-((2*R*,3*R*,4*R*,5*S*,6*R*)-3,4,5-trihydroxy-6-(hydroxymethyl)tetrahydro-2*H*-pyran-2-yl)propanoate (31):

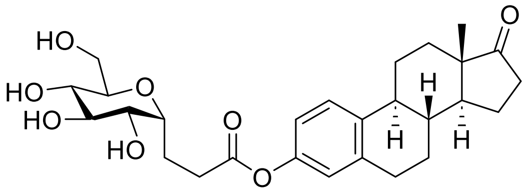

The title compound was prepared according to the **General procedure D** from **2** (1.0 equiv., 0.05 mmol), **S13** (1.5 equiv., 0.075 mmol), Hantzsch ester (2.0 equiv., 0.1 mmol), triethylamine (2.5 equiv., 0.125 mmol), anhydrous DMSO (0.5 mL). The residue was subjected to flash silica

gel column chromatography (eluent: $CHCl_3$/MeOH = 15/1 ~ 5/1) to give the pure product as viscous gel (^{1}H NMR yield: 77%, α:β > 95:5; isolated yield: 16.6 mg, 68%).

^{1}H NMR (500 MHz, Methanol-d_4) δ 7.30 (d, *J* = 8.4 Hz, 1H), 6.86 – 6.79 (m, 2H), **3.99 (dt, *J* = 10.5, 5.5 Hz, 1H, anomeric H)**, 3.78 (dd, *J* = 11.8, 2.5 Hz, 1H), 3.67 – 3.61 (m, 2H), 3.56 (dd, *J* = 9.5, 8.6 Hz, 1H), 3.44 (ddd, *J* = 9.6, 5.7, 2.5 Hz, 1H), 3.29 – 3.26 (m, 1H), 2.90 (dd, *J* = 7.7, 3.3 Hz, 2H), 2.77 – 2.70 (m, 1H), 2.62 (dd, *J* = 16.6, 7.6 Hz, 1H), 2.53 – 2.47 (m, 1H), 2.47 – 2.42 (m, 1H), 2.31 (t, *J* = 11.0 Hz, 1H), 2.18 – 2.12 (m, 1H), 2.11 – 2.02 (m, 4H), 1.93 – 1.88 (m, 1H), 1.71 – 1.61 (m, 2H), 1.60 – 1.53 (m, 2H), 1.51 – 1.45 (m, 2H), 0.93 (s, 3H); ^{13}C NMR (126 MHz, Methanol-d_4) δ 223.65, 174.15, 150.17, 139.22, 138.59, 127.31, 122.66, 119.87, 76.51, 75.16, 74.71, 72.93, 72.22, 63.02, 51.63, 45.45, 39.53, 36.73, 32.78, 31.39, 30.38, 27.48, 26.96, 22.50, 21.35; HRMS (ESI) m/z calcd for $C_{27}H_{36}NaO_8$ [$(M+Na)^+$]: 511.2302, found: 511.2302.

$[\alpha]_D^{25}$: +80.6 (c = 1.0, MeOH).

Ethyl 2-hydroxy-5-(3-((2*R*,3*R*,4*R*,5*S*,6*R*)-3,4,5-trihydroxy-6-(hydroxymethyl)tetrahydro-2*H*-pyran-2-yl)propanamido)benzoate (32):

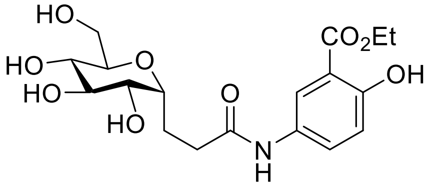

The title compound was prepared according to the **General procedure D** from **2** (1.0 equiv., 0.05 mmol), **S14** (1.5 equiv., 0.075 mmol), Hantzsch ester (2.0 equiv., 0.1 mmol), DABCO (2.5 equiv., 0.125 mmol), anhydrous DMSO (0.5 mL). The residue was subjected to flash silica gel column chromatography (eluent: $CHCl_3$/MeOH = 15/1 ~ 5/1) to give the pure product as viscous gel (^{1}H NMR yield: 73%, α:β > 95:5; isolated yield: 12.6 mg, 63%).

^{1}H NMR (400 MHz, Methanol-d_4) δ 8.10 (d, *J* = 2.7 Hz, 1H), 7.63 (dd, *J* = 8.9, 2.7 Hz, 1H), 6.91 (d, *J* = 9.0 Hz, 1H), 4.42 (q, *J* = 7.1 Hz, 2H), **3.94 (dt, *J* = 10.5, 5.6 Hz, 1H, anomeric H)**, 3.80 (dd, *J* = 11.7, 2.4 Hz, 1H), 3.70 – 3.59 (m, 2H), 3.56 (dd, *J* = 9.5, 8.3 Hz, 1H), 3.47 (ddd, *J* = 8.6, 5.9, 2.5 Hz, 1H), 3.25 (dd, *J* = 9.5, 8.3 Hz, 1H), 2.51 (dt, *J* = 14.5, 7.2 Hz, 1H), 2.41 (dt, *J* = 14.7, 7.7 Hz, 1H), 2.06 (dt, *J* = 9.7, 6.9 Hz, 2H), 1.41 (t, *J* = 7.2 Hz, 3H); ^{13}C NMR (126 MHz, Methanol-d_4) δ 174.31, 171.11, 159.37, 131.68, 129.75, 122.58, 118.49, 113.35, 76.62, 75.15,

74.62, 72.96, 72.30, 63.11, 62.72, 33.96, 22.16, 14.47; HRMS (ESI) m/z calcd for $C_{18}H_{25}NNaO_9$ [(M+Na)$^+$]: 422.1422, found: 422.1422.

[α]$_D^{25}$: +30.7 (c = 1.0, MeOH).

(2*R*,3*R*,4*S*,5*R*,6*R*)-2-(Acetoxymethyl)-6-(3-((2*R*,3*R*,4*R*,5*S*,6*R*)-3,4,5-trihydroxy-6-(hydroxymethyl)tetrahydro-2*H*-pyran-2-yl)propanamido)tetrahydro-2*H*-pyran-3,4,5-triyl triacetate (33):

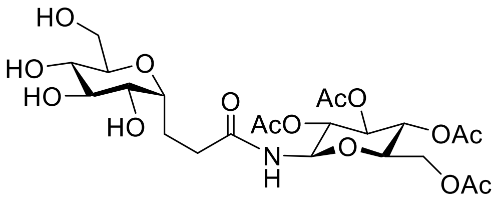

The title compound was prepared according to the **General procedure D** from **2** (1.0 equiv., 0.05 mmol), **S15** (1.5 equiv., 0.075 mmol), Hantzsch ester (2.0 equiv., 0.1 mmol), DABCO (2.5 equiv., 0.125 mmol), anhydrous DMSO (0.5 mL). The residue was subjected to flash silica gel column chromatography (eluent: $CHCl_3$/MeOH = 15/1 ~ 5/1) to give the pure product as viscous gel (^{1}H NMR yield: 64%, α:β > 95:5; isolated yield: 14.0 mg, 50%).

^{1}H NMR (400 MHz, Methanol-*d*$_4$) δ 5.35 – 5.27 (m, 2H), 5.01 (dt, *J* = 19.1, 9.6 Hz, 2H), 4.26 (dd, *J* = 12.4, 4.4 Hz, 1H), 4.10 (dd, *J* = 12.4, 2.2 Hz, 1H), **3.93 (ddd, *J* = 10.0, 4.3, 2.2 Hz, 1H, anomeric H)**, 3.86 (dt, *J* = 8.7, 5.9 Hz, 1H), 3.79 (dd, *J* = 11.7, 2.3 Hz, 1H), 3.64 (dd, *J* = 11.7, 6.1 Hz, 1H), 3.59 (dd, *J* = 9.3, 5.7 Hz, 1H), 3.52 (t, *J* = 8.8 Hz, 1H), 3.42 (ddd, *J* = 8.7, 6.0, 2.3 Hz, 1H), 3.27 – 3.20 (m, 1H), 2.36 (dt, *J* = 14.5, 7.2 Hz, 1H), 2.25 (dt, *J* = 14.8, 7.8 Hz, 1H), 2.03 (s, 3H), 2.02 (s, 3H), 2.01 (s, 3H), 1.98 (s, 3H), 1.97 – 1.90 (m, 2H); ^{13}C NMR (101 MHz, Methanol-*d*$_4$) δ 176.68, 172.37, 171.60, 171.45, 171.34, 78.96, 76.25, 75.10, 74.82, 74.68, 74.65, 72.88, 72.26, 72.11, 69.60, 63.12, 33.20, 22.11, 20.61, 20.56; HRMS (ESI) m/z calcd for $C_{23}H_{35}NNaO_{15}$ [(M+Na)$^+$]: 588.1899, found: 588.1903.

[α]$_D^{25}$: +26.3 (c = 1.0, MeOH).

Methyl *N*-((*tert*-butoxycarbonyl)-*D*-leucyl)-*O*-(3-((2*R*,3*R*,4*R*,5*S*,6*R*)-3,4,5-trihydroxy-6-

(hydroxymethyl)tetrahydro-2*H*-pyran-2-yl)propanoyl)-*L*-threoninate(34):

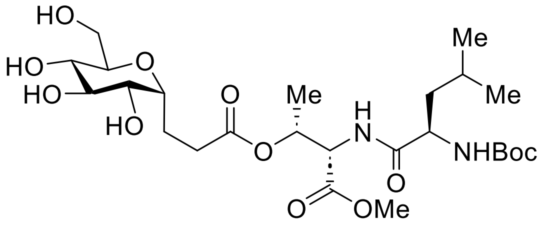

The title compound was prepared according to the **General procedure D** from **2** (1.0 equiv., 0.05 mmol), **S16** (1.5 equiv., 0.075 mmol), Hantzsch ester (2.0 equiv., 0.1 mmol), DABCO (2.5 equiv., 0.125 mmol), anhydrous DMSO (0.5 mL). The residue was subjected to flash silica gel column chromatography (eluent: $CHCl_3$/MeOH = 15/1 ~ 5/1) to give the pure product as viscous gel (^{1}H NMR yield: 68%, α:β > 95:5; isolated yield: 16.9 mg, 60%).

Glycosylation *in situ*: the title compound was prepared according to the **General procedure E** with D-glucose (1.0 equiv., 0.2 mmol), **S16** (1.5 equiv., 0.3 mmol), Hantzsch ester (2.0 equiv., 0.4 mmol), DABCO (2.5 equiv., 0.5 mmol), anhydrous DMSO (2 mL). The residue was re-dissolved in Methanol-d_4 with mesitylene (0.1 mmol) as the internal standard for ^{1}H NMR analysis (^{1}H NMR yield: 44%, α:β > 95:5; the yield was based on **2** as the limiting reagent).

^{1}H NMR (500 MHz, Methanol-d_4) δ 5.44 – 5.36 (m, 1H), 4.73 (d, *J* = 3.1 Hz, 1H), 4.20 (dd, *J* = 9.2, 5.9 Hz, 1H), **3.89 (dt, *J* = 10.5, 5.0 Hz, 1H, anomeric H)**, 3.79 (dd, *J* = 11.8, 2.4 Hz, 1H), 3.72 (s, 3H), 3.64 (dd, *J* = 11.8, 5.9 Hz, 1H), 3.61 (dd, *J* = 9.4, 5.8 Hz, 1H), 3.53 (dd, *J* = 9.4, 8.5 Hz, 1H), 3.40 (ddd, *J* = 8.7, 5.9, 2.6 Hz, 1H), 3.25 (dd, *J* = 9.5, 8.4 Hz, 1H), 2.49 (ddd, *J* = 16.2, 8.4, 6.0 Hz, 1H), 2.38 (dt, *J* = 16.0, 7.7 Hz, 1H), 2.02 – 1.90 (m, 2H), 1.76 – 1.68 (m, 1H), 1.59 – 1.51 (m, 2H), 1.45 (s, 9H), 1.25 (d, *J* = 6.4 Hz, 3H), 0.96 (dd, *J* = 13.5, 6.6 Hz, 6H); ^{13}C NMR (126 MHz, Methanol-d_4) δ 176.34, 174.20, 171.38, 157.88, 80.64, 76.25, 75.10, 74.71, 72.89, 72.23, 71.52, 63.07, 56.79, 54.46, 53.17, 41.86, 31.19, 28.73, 25.90, 23.43, 21.98, 21.37, 17.01; HRMS (ESI) m/z calcd for $C_{25}H_{44}N_2NaO_{12}$ [$(M+Na)^+$]: 587.2786, found: 587.2790.

$[\alpha]_D^{25}$: +18.6 (c = 1.0, MeOH).

Methyl (*S*)-2-((*S*)-2-((*tert*-butoxycarbonyl)amino)-4-methylpentanamido)-3-(4-((3-((2*R*,3*R*,4*R*,5*S*,6*R*)-3,4,5-trihydroxy-6-(hydroxymethyl)tetrahydro-2*H*-pyran-2-yl)propanoyl)oxy)phenyl)propanoate (35):

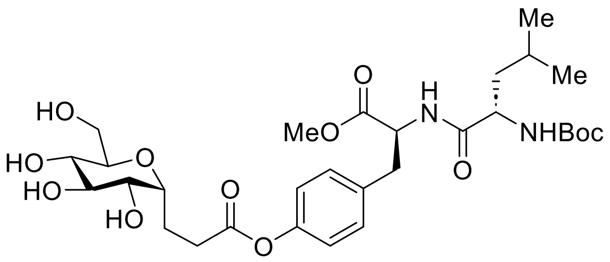

The title compound was prepared according to the **General procedure D** from **2** (1.0 equiv., 0.05 mmol), **S17** (1.5 equiv., 0.075 mmol), Hantzsch ester (2.0 equiv., 0.1 mmol), triethylamine (2.5 equiv., 0.125 mmol), anhydrous DMSO (0.5 mL). The residue was subjected to flash silica gel column chromatography (eluent: $CHCl_3$/MeOH = 15/1 ~ 5/1) to give the pure product as viscous gel (^{1}H NMR yield: 66%, α:β > 95:5; isolated yield: 19.1 mg, 61%).

^{1}H NMR (400 MHz, Methanol-d_4) δ 7.22 (d, *J* = 8.3 Hz, 2H), 7.02 (d, *J* = 8.4 Hz, 2H), 4.68 (dd, *J* = 8.0, 5.8 Hz, 1H), 4.09 – 4.02 (m, 1H), **3.99 (dt, *J* = 10.5, 5.6 Hz, 1H, anomeric H)**, 3.78 (dd, *J* = 11.8, 2.5 Hz, 1H), 3.71 – 3.61 (m, 5H), 3.55 (t, *J* = 9.0 Hz, 1H), 3.44 (ddd, *J* = 8.6, 5.7, 2.5 Hz, 1H), 3.27 (dd, *J* = 9.6, 8.5 Hz, 1H), 3.16 (dd, *J* = 13.9, 5.7 Hz, 1H), 3.04 (dd, *J* = 13.9, 7.8 Hz, 1H), 2.75 (dt, *J* = 16.5, 7.2 Hz, 1H), 2.63 (dt, *J* = 16.3, 7.6 Hz, 1H), 2.09 (q, *J* = 10.1, 8.7 Hz, 2H), 1.65 – 1.59 (m, 1H), 1.43 (s, 11H), 0.93 (d, *J* = 6.6 Hz, 3H), 0.90 (d, *J* = 6.5 Hz, 3H); ^{13}C NMR (126 MHz, Methanol-d_4) δ 175.58, 173.83, 173.07, 151.23, 135.59, 131.36, 122.73, 116.27, 80.64, 76.47, 75.16, 74.71, 72.92, 72.22, 63.04, 54.86, 54.52, 52.74, 42.17, 37.66, 31.35, 28.72, 25.86, 23.33, 21.95, 21.33; HRMS (ESI) m/z calcd for $C_{30}H_{46}N_2NaO_{12}$ [$(M+Na)^+$]: 649.2943, found: 649.2950.

$[\alpha]_D^{25}$: +40.9 (c = 1.0, MeOH).

Methyl (3-((2*R*,3*R*,4*R*,5*S*,6*R*)-3,4,5-trihydroxy-6-(hydroxymethyl)tetrahydro-2*H*-pyran-2-yl)propanoyl)-*L*-phenylalanyl-*L*-leucinate (36):

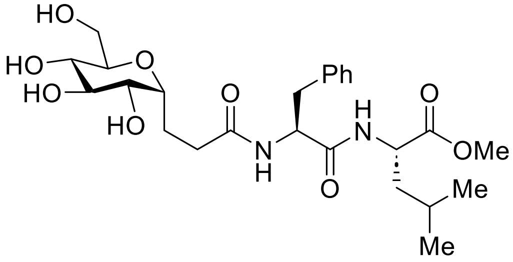

The title compound was prepared according to the **General procedure D** from **2** (1.0 equiv., 0.05 mmol), **S18** (1.5 equiv., 0.075 mmol), Hantzsch ester (2.0 equiv., 0.1 mmol), DABCO (2.5

equiv., 0.125 mmol), anhydrous DMSO (0.5 mL). The residue was subjected to flash silica gel column chromatography (eluent: $CHCl_3$/MeOH = 15/1 ~ 5/1) to give the pure product as viscous gel (^{1}H NMR yield: 73%, α:β > 95:5; isolated yield: 15.8 mg, 62%).

^{1}H NMR (400 MHz, Methanol-d_4) δ 7.33 – 7.17 (m, 5H), 4.66 (dd, *J* = 9.6, 5.2 Hz, 1H), 4.48 (dd, *J* = 8.9, 6.0 Hz, 1H), **3.91 – 3.84 (m, 2H, one of them is anomeric H)**, 3.69 (s, 3H), 3.63 – 3.54 (m, 2H), 3.49 (t, *J* = 8.9 Hz, 1H), 3.41 (ddd, *J* = 9.2, 6.9, 2.2 Hz, 1H), 3.21 – 3.13 (m, 2H), 2.84 (dd, *J* = 13.9, 9.6 Hz, 1H), 2.32 – 2.15 (m, 2H), 1.95 – 1.86 (m, 1H), 1.84 – 1.74 (m, 1H), 1.72 – 1.65 (m, 1H), 1.64 – 1.59 (m, 2H), 0.94 (dd, *J* = 16.3, 6.3 Hz, 6H); ^{13}C NMR (126 MHz, Methanol-d_4) δ 175.77, 174.32, 173.98, 138.48, 130.30, 129.47, 127.78, 75.82, 75.23, 74.76, 72.91, 72.54, 63.36, 55.85, 52.71, 52.11, 41.48, 38.94, 32.73, 25.83, 23.31, 22.37, 21.81; HRMS (ESI) m/z calcd for $C_{25}H_{38}N_2NaO_9$ [(M+Na)$^+$]: 533.2470, found: 533.2473.

$[\alpha]_D^{25}$: +15.3 (c = 1.0, MeOH).

(2*R*,3*S*,4*R*,5*R*,6*R*)-2-(Hydroxymethyl)-6-(2-(phenylsulfonyl)ethyl)tetrahydro-2*H*-pyran-3,4,5-triol (37):

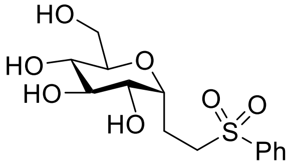

The title compound was prepared according to the **General procedure D** from **2** (1.0 equiv., 0.05 mmol), **S19** (1.5 equiv., 0.075 mmol), Hantzsch ester (2.0 equiv., 0.1 mmol), DABCO (2.5 equiv., 0.125 mmol), anhydrous DMSO (0.5 mL). The residue was subjected to flash silica gel column chromatography (eluent: $CHCl_3$/MeOH = 15/1 ~ 5/1) to give the pure product as viscous gel (^{1}H NMR yield: 94%, α:β > 95:5; isolated yield: 12.1 mg, 73%).

Glycosylation *in situ*: the title compound was prepared according to the **General procedure E** with D-glucose (1.0 equiv., 0.2 mmol), **S19** (1.5 equiv., 0.3 mmol), Hantzsch ester (2.0 equiv., 0.4 mmol), DABCO (2.5 equiv., 0.5 mmol), anhydrous DMSO (2 mL). The residue was re-dissolved in Methanol-d4-d_4 with mesitylene (0.1 mmol) as the internal standard for ^{1}H NMR analysis (^{1}H NMR yield: 67%, α:β > 95:5; the yield was based on **2** as the limiting reagent).

^{1}H NMR (500 MHz, Methanol-d_4) δ 8.00 – 7.89 (m, 2H), 7.78 – 7.69 (m, 1H), 7.65 (dd, *J* = 8.4, 7.1 Hz, 2H), **3.92 (dt, *J* = 10.6, 5.8 Hz, 1H, anomeric H)**, 3.76 (dd, *J* = 11.8, 2.3 Hz, 1H), 3.61 –

3.54 (m, 2H), 3.45 – 3.35 (m, 2H), 3.28 – 3.15 (m, 3H), 2.08 – 1.98 (m, 2H); ^{13}C NMR (101 MHz, Methanol-d_4) δ 140.36, 135.08, 130.58, 129.11, 75.29, 74.98, 72.59, 72.08, 63.06, 53.36, 19.95; HRMS (ESI) m/z calcd for $C_{14}H_{20}NaO_7S$ [(M+Na)$^+$]: 355.0822, found: 355.0827.
$[\alpha]_D^{25}$: +53.8 (c = 1.0, MeOH).

Diethyl (2-((2*R*,3*R*,4*R*,5*S*,6*R*)-3,4,5-trihydroxy-6-(hydroxymethyl)tetrahydro-2*H*-pyran-2-yl)ethyl)phosphonate (38):

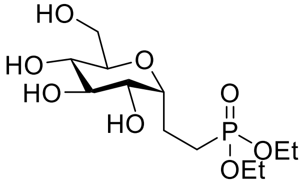

The title compound was prepared according to the **General procedure D** from **2** (1.0 equiv., 0.05 mmol), **S20** (3.0 equiv., 0.15 mmol), Hantzsch ester (2.0 equiv., 0.1 mmol), DABCO (2.5 equiv., 0.125 mmol), anhydrous DMSO (0.5 mL). The residue was subjected to flash silica gel column chromatography (eluent: $CHCl_3$/MeOH = 15/1 ~ 5/1) to give the pure product as viscous gel (^{1}H NMR yield: 92%, α:β > 95:5; isolated yield: 11.6 mg, 71%).
Glycosylation *in situ*: the title compound was prepared according to the **General procedure E** with D-glucose (1.0 equiv., 0.2 mmol), **S20** (3.0 equiv., 0.6 mmol), Hantzsch ester (2.0 equiv., 0.4 mmol), DABCO (2.5 equiv., 0.5 mmol), anhydrous DMSO (2 mL). The residue was re-dissolved in Methanol-d_4 with mesitylene (0.1 mmol) as the internal standard for ^{1}H NMR analysis (^{1}H NMR yield: 75%, α:β > 95:5; the yield was based on **2** as the limiting reagent).
^{1}H NMR (500 MHz, Methanol-d_4) δ 4.15 – 4.07 (m, 4H), **3.89 (ddd, *J* = 10.3, 5.8, 3.9 Hz, 1H, anomeric H)**, 3.81 (dd, *J* = 11.8, 2.4 Hz, 1H), 3.65 – 3.58 (m, 2H), 3.51 (t, *J* = 9.0 Hz, 1H), 3.36 (td, *J* = 6.5, 3.1 Hz, 1H), 3.21 (dd, *J* = 9.5, 8.6 Hz, 1H), 2.09 – 1.99 (m, 1H), 1.99 – 1.88 (m, 2H), 1.82 – 1.72 (m, 1H), 1.33 (t, *J* = 7.0 Hz, 6H); ^{13}C NMR (126 MHz, Methanol-d_4) δ 76.93, 76.80, 75.15, 74.80, 72.87, 72.32, 63.25 (d, *J* = 7.0 Hz), 63.20, 21.82 (d, *J* = 142.3 Hz) 19.14 (d, *J* = 4.0 Hz), 16.73 (d, *J* = 6.0 Hz); HRMS (ESI) m/z calcd for $C_{12}H_{25}NaO_8P$ [(M+Na)$^+$]: 351.1179, found: 351.1184.
$[\alpha]_D^{25}$: +46.3 (c = 1.0, MeOH).

(2*R*,3*R*,4*R*,5*S*,6*R*)-2-(2-(Dimethyl(phenyl)silyl)ethyl)-6-(hydroxymethyl)tetrahydro-2*H*-pyran-3,4,5-triol (39):

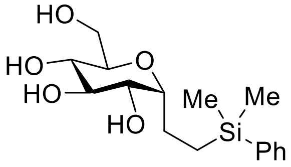

The title compound was prepared according to the **General procedure D** from **2** (1.0 equiv., 0.05 mmol), **S21** (3.0 equiv., 0.15 mmol), Hantzsch ester (2.0 equiv., 0.1 mmol), DABCO (2.5 equiv., 0.125 mmol), anhydrous DMSO (0.5 mL). The residue was subjected to flash silica gel column chromatography (eluent: $CHCl_3$/MeOH = 15/1 ~ 5/1) to give the pure product as viscous gel (^{1}H NMR yield: 61%, α:β > 95:5; isolated yield: 7.8 mg, 48%).

Glycosylation *in situ*: the title compound was prepared according to the **General procedure E** with D-glucose (1.0 equiv., 0.2 mmol), **S21** (3.0 equiv., 0.6 mmol), Hantzsch ester (2.0 equiv., 0.4 mmol), DABCO (2.5 equiv., 0.5 mmol), anhydrous DMSO (2 mL). The residue was re-dissolved in Methanol-d_4 with mesitylene (0.1 mmol) as the internal standard for ^{1}H NMR analysis (^{1}H NMR yield: 36%, α:β > 95:5; the yield was based on **2** as the limiting reagent).

^{1}H NMR (500 MHz, Methanol-d_4) δ 7.54 – 7.49 (m, 2H), 7.34 – 7.30 (m, 3H), **3.78 – 3.74 (m, 2H, one of them is anomeric H)**, 3.62 (dd, *J* = 11.8, 5.5 Hz, 1H), 3.58 (dd, *J* = 9.5, 5.8 Hz, 1H), 3.45 (dd, *J* = 9.5, 8.3 Hz, 1H), 3.27 (ddd, *J* = 9.5, 5.6, 2.5 Hz, 1H), 3.22 (dd, *J* = 9.6, 8.3 Hz, 1H), 1.72 – 1.57 (m, 2H), 1.06 (ddd, *J* = 14.7, 12.9, 4.2 Hz, 1H), 0.62 (ddd, *J* = 14.7, 12.2, 5.0 Hz, 1H), 0.28 (d, *J* = 2.9 Hz, 6H); ^{13}C NMR (126 MHz, Methanol-d_4) δ 140.16, 134.59, 129.92, 128.82, 79.56, 75.21, 74.26, 73.25, 72.39, 63.15, 19.81, 11.82, -2.88, -3.03; HRMS (ESI) m/z calcd for $C_{16}H_{26}NaO_5Si$ [(M+Na)$^+$]: 349.1442, found: 349.1443.

$[\alpha]_D^{25}$: +32.8 (c = 1.0, MeOH).

(2*R*,3*R*,4*R*,5*S*,6*R*)-2-(2-((6b*R*,9a*S*)-6*b*,9*a*-Dimethyl-6*b*,9*a*-dihydroacenaphtho[1,2-*d*][1,3,2]dioxaborol-8-yl)ethyl)-6-(hydroxymethyl)tetrahydro-2*H*-pyran-3,4,5-triol (40):

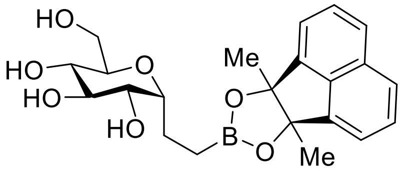

The title compound was prepared according to the **General procedure D** from **2** (1.0 equiv., 0.05 mmol), **S22** (1.5 equiv., 0.075 mmol), Hantzsch ester (2.0 equiv., 0.1 mmol), DABCO (2.5 equiv., 0.125 mmol), anhydrous DMSO (0.5 mL). The residue was subjected to flash silica gel column chromatography (eluent: $CHCl_3$/MeOH = 15/1 ~ 5/1) to give the pure product as viscous gel (^{1}H NMR yield: 78%, α:β > 95:5; isolated yield: 10.6 mg, 51%).

^{1}H NMR (500 MHz, Methanol-*d*$_4$) δ 7.82 – 7.78 (m, 2H), 7.60 (dd, *J* = 8.2, 6.9 Hz, 2H), 7.53 (d, *J* = 6.9 Hz, 2H), **3.71 (dt, *J* = 10.4, 5.5 Hz, 1H, anomeric H)**, 3.60 (dd, *J* = 11.8, 2.5 Hz, 1H), 3.53 – 3.45 (m, 3H), 3.26 (ddd, *J* = 9.4, 5.5, 2.6 Hz, 1H), 3.18 (dd, *J* = 9.6, 8.2 Hz, 1H), 1.76 (d, *J* = 2.6 Hz, 6H), 1.70 – 1.62 (m, 2H), 0.88 (ddd, *J* = 15.7, 8.6, 6.2 Hz, 1H), 0.66 (dt, *J* = 16.5, 8.0 Hz, 1H); ^{13}C NMR (126 MHz, Methanol-*d*$_4$) δ 132.89, 129.55, 126.29, 126.27, 120.53, 120.51, 93.10, 78.51, 75.15, 74.17, 73.22, 72.29, 62.97, 22.38, 22.29, 19.98; HRMS (ESI) m/z calcd for $C_{22}H_{27}BNaO_7$ [(M+Na)$^+$]: 437.1742, found: 437.1752.

$[\alpha]_D^{25}$: +34.4 (c = 1.0, MeOH).

3-((2*R*,3*R*,4*R*,5*S*,6*R*)-3,4,5-Trihydroxy-6-(hydroxymethyl)tetrahydro-2*H*-pyran-2-yl)propyl acetate (41):

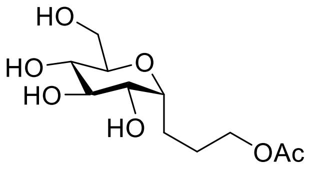

The title compound was prepared according to the **General procedure D** from **2** (1.0 equiv., 0.05 mmol), **S23** (3.0 equiv., 0.15 mmol), Hantzsch ester (2.0 equiv., 0.1 mmol), DABCO (2.5 equiv., 0.125 mmol), anhydrous DMSO (0.5 mL). The residue was subjected to flash silica gel column chromatography (eluent: $CHCl_3$/MeOH = 15/1 ~ 5/1) to give the pure product as viscous gel (^{1}H NMR yield: 56%, α:β > 95:5; isolated yield: 5.9 mg, 45%).

^{1}H NMR (500 MHz, Methanol-*d*$_4$) δ 4.12 (td, *J* = 6.4, 3.4 Hz, 2H), **3.90 (dt, *J* = 7.9, 6.0 Hz, 1H, anomeric H)**, 3.78 (dd, *J* = 11.7, 2.5 Hz, 1H), 3.63 (dd, *J* = 11.7, 5.8 Hz, 1H), 3.59 (dd, *J* = 9.5, 5.8 Hz, 1H), 3.52 (dd, *J* = 9.5, 8.5 Hz, 1H), 3.39 (ddd, *J* = 9.6, 5.7, 2.5 Hz, 1H), 3.25 (dd, *J* = 9.6, 8.5 Hz, 1H), 2.03 (s, 3H), 1.88 – 1.79 (m, 1H), 1.76 – 1.70 (m, 2H), 1.65 (ddd, *J* = 12.5, 8.4, 6.2 Hz, 1H); ^{13}C NMR (126 MHz, Methanol-*d*$_4$) δ 173.12, 76.92, 75.20, 74.47, 73.02, 72.36, 65.57,

63.12, 26.01, 22.05, 20.84; HRMS (ESI) m/z calcd for $C_{11}H_{20}NaO_7$ [$(M+Na)^+$]: 287.1101, found: 287.1108.

$[\alpha]_D^{25}$: +32.7 (c = 1.0, MeOH).

(2*R*,3*S*,4*R*,5*R*,6*R*)-2-(Hydroxymethyl)-6-((4-phenylcyclohexyl)methyl)tetrahydro-2*H*-pyran-3,4,5-triol (42):

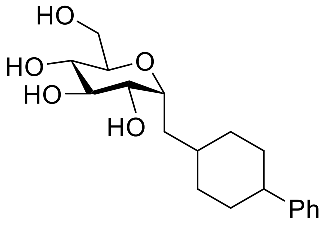

The title compound was prepared according to the **General procedure D** from **2** (1.0 equiv., 0.05 mmol), **S24** (5.0 equiv., 0.25 mmol), Hantzsch ester (2.0 equiv., 0.1 mmol), DABCO (2.5 equiv., 0.125 mmol), anhydrous DMSO (0.5 mL). The residue was subjected to flash silica gel column chromatography (eluent: $CHCl_3$/MeOH = 15/1 ~ 5/1) to give the pure product as viscous gel (^{1}H NMR yield: 42%, α:β > 95:5; isolated yield: 5.0 mg, 30%).

^{1}H NMR (500 MHz, Methanol-d_4) δ 7.26 – 7.18 (m, 4H), 7.15 – 7.10 (m, 1H), **4.08 (ddd, *J* = 11.8, 5.6, 3.1 Hz, 1H, anomeric H)**, 3.80 (dd, *J* = 11.7, 2.6 Hz, 1H), 3.70 – 3.65 (m, 1H), 3.60 (dd, *J* = 9.5, 5.6 Hz, 1H), 3.55 (dd, *J* = 9.5, 8.2 Hz, 1H), 3.45 (ddd, *J* = 9.6, 5.5, 2.6 Hz, 1H), 3.27 (dd, *J* = 9.6, 8.2 Hz, 1H), 2.47 (tt, *J* = 12.1, 3.4 Hz, 1H), 2.05 (dt, *J* = 12.8, 2.8 Hz, 1H), 1.92 – 1.85 (m, 3H), 1.75 – 1.69 (m, 1H), 1.60 – 1.43 (m, 4H), 1.27 – 1.17 (m, 1H), 1.06 (qd, *J* = 12.9, 3.4 Hz, 1H); ^{13}C NMR (126 MHz, Methanol-d_4) δ 148.97, 129.27, 127.78, 126.81, 75.17, 74.70, 74.51, 73.06, 72.49, 63.14, 46.03, 35.85, 35.67, 35.40, 34.53, 33.59, 32.70; HRMS (ESI) m/z calcd for $C_{19}H_{28}NaO_5$ [$(M+Na)^+$]: 359.1829, found: 359.1835.

$[\alpha]_D^{25}$: +44.0 (c = 1.0, MeOH).

(2*R*,3*R*,4*R*,5*S*,6*R*)-2-(((3*aR*,4*S*,6*R*,6*aS*)-6-((*R*)-2,2-Dimethyl-1,3-dioxolan-4-yl)-2,2-dimethyltetrahydrofuro[3,4-*d*][1,3]dioxol-4-yl)methyl)-6-(hydroxymethyl)tetrahydro-2*H*-pyran-3,4,5-triol (43):

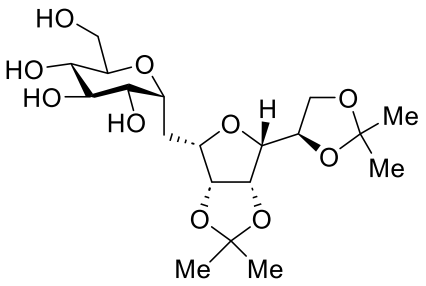

The title compound was prepared according to the **General procedure D** from **2** (1.0 equiv., 0.05 mmol), **S25** (3.0 equiv., 0.15 mmol), Hantzsch ester (2.0 equiv., 0.1 mmol), DABCO (2.5 equiv., 0.125 mmol), anhydrous DMSO (0.5 mL). The residue was subjected to flash silica gel column chromatography (eluent: $CHCl_3$/MeOH = 15/1 ~ 5/1) to give the pure product as viscous gel (^{1}H NMR yield: 55%, α:β > 95:5; isolated yield: 7.6 mg, 36%).

^{1}H NMR (500 MHz, Methanol-d_4) δ 4.77 (dd, *J* = 6.1, 3.4 Hz, 1H), 4.73 (dd, *J* = 6.1, 3.5 Hz, 1H), 4.34 (q, *J* = 6.3 Hz, 1H), 4.13 (dt, *J* = 10.4, 5.0 Hz, 1H), 4.05 (dd, *J* = 8.4, 6.4 Hz, 1H), 3.97 (dd, *J* = 8.4, 6.0 Hz, 1H), 3.79 (dd, *J* = 11.8, 2.5 Hz, 1H), **3.75 (ddd, *J* = 8.7, 5.9, 3.4 Hz, 1H, anomeric H)**, 3.65 – 3.54 (m, 4H), 3.48 (ddd, *J* = 8.8, 6.0, 2.5 Hz, 1H), 3.25 (dd, *J* = 9.5, 8.0 Hz, 1H), 2.16 – 2.04 (m, 2H), 1.42 (s, 3H), 1.39 (s, 3H), 1.33 (s, 3H), 1.31 (s, 3H); ^{13}C NMR (126 MHz, Methanol-d_4) δ 113.07, 109.87, 82.76, 82.18, 82.05, 80.48, 75.07, 74.96, 74.64, 74.39, 72.90, 72.29, 67.77, 63.09, 26.90, 26.11, 25.51, 25.28, 24.79; HRMS (ESI) m/z calcd for $C_{19}H_{32}NaO_{10}$ [(M+Na)$^+$]: 443.1888, found: 443.1895.

$[\alpha]_D^{25}$: +30.4 (c = 1.0, MeOH).

(2*R*,3*R*,4*R*,5*S*,6*R*)-2-((*E*)-4-(Benzyloxy)-3-methoxystyryl)-6-(hydroxymethyl)tetrahydro-2*H*-pyran-3,4,5-triol (44):

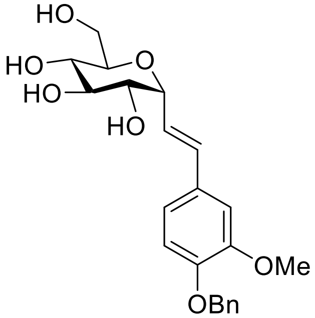

The title compound was prepared according to the **General procedure D** from **2** (1.0 equiv., 0.05 mmol), **S26** (3.0 equiv., 0.15 mmol), Hantzsch ester (2.0 equiv., 0.1 mmol), DABCO (2.5

equiv., 0.125 mmol), anhydrous DMSO (0.5 mL). The residue was subjected to flash silica gel column chromatography (eluent: $CHCl_3$/MeOH = 15/1 ~ 5/1) to give the pure product as viscous gel (^{1}H NMR yield: 55%, α:β > 95:5, *E:Z* = 77:23; isolated yield: 8.0 mg, 40%).

^{1}H NMR (500 MHz, Methanol-d_4) δ 7.46 – 7.42 (m, 2H), 7.38 – 7.33 (m, 2H), 7.32 – 7.28 (m, 1H), 7.10 (d, *J* = 1.2 Hz, 1H), 6.93 (d, *J* = 1.1 Hz, 2H), 6.70 (dd, *J* = 16.2, 1.8 Hz, 1H), 6.42 (dd, *J* = 16.2, 5.5 Hz, 1H), 5.10 (s, 2H), **4.60 – 4.57 (m, 2H, one of them is anomeric H)**, 3.87 (s, 3H), 3.81 (d, *J* = 5.1 Hz, 1H), 3.71 – 3.64 (m, 3H), 3.58 – 3.53 (m, 1H); ^{13}C NMR (126 MHz, Methanol-d_4) δ 151.24, 149.45, 138.65, 135.39, 132.31, 129.46, 128.92, 128.72, 122.84, 120.88, 115.56, 111.06, 77.44, 75.76, 75.43, 73.24, 72.61, 72.20, 63.28, 56.52; HRMS (ESI) m/z calcd for $C_{22}H_{26}NaO_7$ [(M+Na)$^+$]: 425.1571, found: 425.1573.

$[\alpha]_D^{25}$: +25.5 (c = 1.0, MeOH).

(2*R*,3*S*,4*R*,5*R*,6*R*)-2-(Hydroxymethyl)-6-(imidazo[*1,2-a*]pyrazin-8-yl)tetrahydro-2*H*-pyran-3,4,5-triol (45):

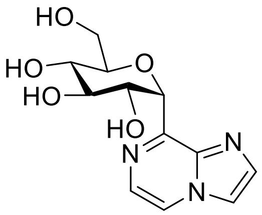

The title compound was prepared according to the **General procedure D** from **2** (1.0 equiv., 0.05 mmol), **S27** (5.0 equiv., 0.25 mmol), Hantzsch ester (2.0 equiv., 0.1 mmol), DABCO (2.5 equiv., 0.125 mmol), anhydrous DMSO (0.5 mL). The residue was subjected to flash silica gel column chromatography (eluent: $CHCl_3$/MeOH = 15/1 ~ 5/1) to give the pure product as viscous gel (^{1}H NMR yield: 31%, α:β > 95:5; isolated yield: 3.1 mg, 22%).

^{1}H NMR (500 MHz, Methanol-d_4) δ 8.48 (d, *J* = 4.6 Hz, 1H), 8.06 (d, *J* = 1.2 Hz, 1H), 7.91 (d, *J* = 4.6 Hz, 1H), 7.80 (d, *J* = 1.2 Hz, 1H), **5.86 (d, *J* = 6.0 Hz, 1H, anomeric H)**, 4.47 (t, *J* = 8.6 Hz, 1H), 4.02 (dd, *J* = 8.9, 6.0 Hz, 1H), 3.75 – 3.66 (m, 3H), 3.43 (t, *J* = 8.5 Hz, 1H); ^{13}C NMR (126 MHz, Methanol-d_4) δ 152.42, 141.24, 135.21, 128.85, 121.30, 116.45, 77.64, 74.95, 73.73, 73.64, 72.27, 62.78; HRMS (ESI) m/z calcd for $C_{12}H_{15}N_3NaO_5$ [(M+Na)$^+$]: 304.0904, found: 304.0910.

$[\alpha]_D^{25}$: +81.8 (c = 1.0, MeOH).

(2*R*,3*R*,4*R*,5*S*,6*R*)-2-(6-Chloroimidazo[*1,2-a*]pyrazin-8-yl)-6-(hydroxymethyl)tetrahydro-2*H* -pyran-3,4,5-triol (46):

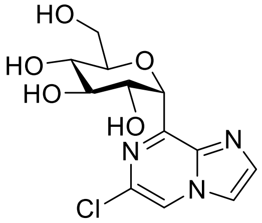

The title compound was prepared according to the **General procedure D** from **2** (1.0 equiv., 0.05 mmol), **S28** (3.0 equiv., 0.15 mmol), Hantzsch ester (2.0 equiv., 0.1 mmol), DABCO (2.5 equiv., 0.125 mmol), anhydrous DMSO (0.5 mL). The residue was subjected to flash silica gel column chromatography (eluent: $CHCl_3$/MeOH = 15/1 ~ 5/1) to give the pure product as viscous gel (^{1}H NMR yield: 44%, α:β > 95:5; isolated yield: 5.7 mg, 36%).

^{1}H NMR (500 MHz, Methanol-d_4) δ 8.66 (d, *J* = 1.3 Hz, 1H), 8.07 (d, *J* = 1.2 Hz, 1H), 7.84 (d, *J* = 1.2 Hz, 1H), **5.93 (d, *J* = 6.4 Hz, 1H, anomeric H)**, 4.50 (t, *J* = 8.8 Hz, 1H), 4.05 – 4.00 (m, 2H), 3.74 (dd, *J* = 12.0, 2.7 Hz, 1H), 3.69 (dd, *J* = 12.0, 5.9 Hz, 1H), 3.44 (t, *J* = 8.8 Hz, 1H); ^{13}C NMR (126 MHz, Methanol-d_4) δ 151.58, 140.84, 136.67, 134.04, 118.97, 117.08, 77.56, 74.60, 73.33, 73.27, 72.11, 62.82; HRMS (ESI) m/z calcd for $C_{12}H_{14}ClN_3NaO_5$ [$(M+Na)^+$]: 338.0514, found: 338.0518.

$[\alpha]_D^{25}$: +61.1 (c = 1.0, MeOH).

(2*R*,3*R*,4*R*,5*R*,6*R*)-2-(6-Chloroimidazo[1,2-*a*]pyrazin-8-yl)-6-(hydroxymethyl)tetrahydro-2 *H*-pyran-3,4,5-triol (47):

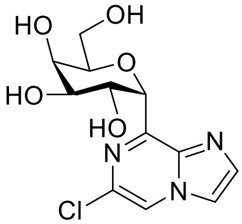

The title compound was prepared according to the **General procedure D** from **S1** (1.0 equiv., 0.05 mmol), **S28** (3.0 equiv., 0.15 mmol), Hantzsch ester (2.0 equiv., 0.1 mmol), DABCO (2.5 equiv., 0.125 mmol), anhydrous DMSO (0.5 mL). The residue was subjected to flash silica gel column chromatography (eluent: $CHCl_3$/MeOH = 15/1 ~ 5/1) to give the pure product as viscous gel (^{1}H NMR yield: 36%, α:β > 95:5; isolated yield: 5.4 mg, 34%).

^{1}H NMR (500 MHz, Methanol-*d*$_4$) δ 8.65 (s, 1H), 8.06 (d, *J* = 1.2 Hz, 1H), 7.82 (d, *J* = 1.2 Hz, 1H), **5.97 (d, *J* = 5.8 Hz, 1H, anomeric H)**, 4.53 (dd, *J* = 8.7, 3.4 Hz, 1H), 4.40 (dd, *J* = 8.7, 5.8 Hz, 1H), 4.34 (ddd, *J* = 7.2, 4.5, 2.5 Hz, 1H), 4.14 – 4.10 (m, 1H), 3.86 (dd, *J* = 11.8, 7.4 Hz, 1H), 3.70 (dd, *J* = 11.7, 4.6 Hz, 1H); ^{13}C NMR (126 MHz, Methanol-*d*$_4$) δ 151.81, 140.68, 136.62, 134.11, 118.85, 117.04, 76.79, 72.39, 71.60, 70.40, 70.24, 62.16; HRMS (ESI) m/z calcd for $C_{12}H_{14}ClN_3NaO_5$ [(M+Na)$^+$]: 338.0514, found: 338.0516.
$[\alpha]_D^{25}$: +21.48 (c = 1.0, MeOH).

(2*R*,3*R*,4*S*,5*S*,6*R*)-2-(Benzylselanyl)-6-(hydroxymethyl)tetrahydro-2*H*-pyran-3,4,5-triol (48):

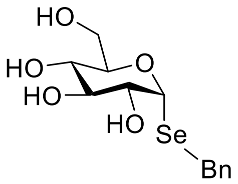

The title compound was prepared according to the **General procedure D** from **2** (1.0 equiv., 0.05 mmol), **S29** (3.0 equiv., 0.15 mmol), Hantzsch ester (2.0 equiv., 0.1 mmol), DABCO (2.5 equiv., 0.125 mmol), anhydrous DMA (0.5 mL). The residue was subjected to flash silica gel column chromatography (eluent: $CHCl_3$/MeOH = 15/1 ~ 5/1) to give the pure product as viscous gel (^{1}H NMR yield: 40%, α:β > 95:5; isolated yield: 6.0 mg, 36%).
^{1}H NMR (500 MHz, Methanol-*d*$_4$) δ 7.37 – 7.32 (m, 2H), 7.25 (t, *J* = 7.6 Hz, 2H), 7.19 – 7.14 (m, 1H), **5.62 (d, *J* = 5.3 Hz, 1H, anomeric H)**, 3.97 (ddd, *J* = 9.9, 5.6, 2.4 Hz, 1H), 3.83 (d, *J* = 11.8 Hz, 2H), 3.77 – 3.70 (m, 2H), 3.61 (dd, *J* = 9.3, 5.3 Hz, 1H), 3.54 (t, *J* = 9.1 Hz, 1H), 3.36 (dd, *J* = 9.9, 8.8 Hz, 1H); ^{13}C NMR (126 MHz, Methanol-*d*$_4$) δ 141.14, 130.09, 129.32, 127.45, 83.73, 76.61, 75.63, 73.39, 71.43, 62.47, 25.71; HRMS (ESI) m/z calcd for $C_{13}H_{18}NaO_5Se$ [(M+Na)$^+$]: 357.0212, found: 357.0218.
$[\alpha]_D^{25}$: +142.40 (c = 1.0, MeOH).

(2*R*,3*R*,4*S*,5*R*,6*R*)-2-(Benzylselanyl)-6-(hydroxymethyl)tetrahydro-2*H*-pyran-3,4,5-triol (49):

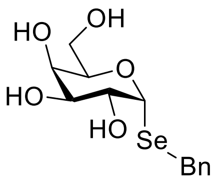

The title compound was prepared according to the **General procedure D** from **S1** (1.0 equiv., 0.05 mmol), **S29** (3.0 equiv., 0.15 mmol), Hantzsch ester (2.0 equiv., 0.1 mmol), DABCO (2.5 equiv., 0.125 mmol), anhydrous DMA (0.5 mL). The residue was subjected to flash silica gel column chromatography (eluent: $CHCl_3$/MeOH = 15/1 ~ 5/1) to give the pure product as viscous gel (^{1}H NMR yield: 34%, α:β > 95:5; isolated yield: 5.2 mg, 31%).

^{1}H NMR (500 MHz, Methanol-d_4) δ 7.35 (dd, *J* = 7.6, 1.6 Hz, 2H), 7.24 (dd, *J* = 8.4, 6.9 Hz, 2H), 7.18 – 7.13 (m, 1H), **5.67 (d, *J* = 5.6 Hz, 1H, anomeric H)**, 4.21 (t, *J* = 6.0 Hz, 1H), 3.97 (dd, *J* = 9.8, 5.5 Hz, 1H), 3.92 (dd, *J* = 3.3, 1.3 Hz, 1H), 3.83 (d, *J* = 11.8 Hz, 1H), 3.79 – 3.75 (m, 2H), 3.72 (d, *J* = 11.8 Hz, 1H), 3.61 (dd, *J* = 9.8, 3.4 Hz, 1H); ^{13}C NMR (126 MHz, Methanol-d_4) δ 141.24, 130.07, 129.29, 127.39, 84.00, 74.38, 73.21, 70.71, 69.98, 62.69, 25.42; HRMS (ESI) m/z calcd for $C_{13}H_{18}NaO_5Se$ [$(M+Na)^+$]: 357.0212, found: 357.0219.

$[\alpha]_D^{25}$: +186.35 (c = 1.0, MeOH).

(2*R*,3*S*,4*S*,5*R*,6*R*)-2-(Hydroxymethyl)-6-(phenethylthio)tetrahydro-2*H*-pyran-3,4,5-triol (50):

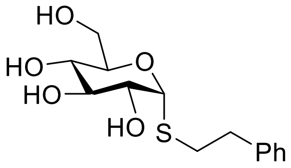

The title compound was prepared according to the **General procedure D** from **2** (1.0 equiv., 0.05 mmol), **S30** (3.0 equiv., 0.15 mmol), Hantzsch ester (2.0 equiv., 0.1 mmol), DABCO (2.5 equiv., 0.125 mmol), anhydrous DMSO (0.5 mL). The residue was subjected to flash silica gel column chromatography (eluent: $CHCl_3$/MeOH = 15/1 ~ 5/1) to give the pure product as viscous gel (^{1}H NMR yield: 70%, α:β > 95:5; isolated yield: 8.9 mg, 59%).

^{1}H NMR (500 MHz, Methanol-d_4) δ 7.33 – 7.11 (m, 5H), **5.37 (d, *J* = 5.3 Hz, 1H, anomeric H)**, 3.94 (ddd, *J* = 10.0, 5.4, 2.3 Hz, 1H), 3.80 (dd, *J* = 11.9, 2.4 Hz, 1H), 3.75 – 3.67 (m, 2H), 3.52 (td, *J* = 9.3, 2.5 Hz, 1H), 3.33 (d, *J* = 13.5 Hz, 1H), 2.95 – 2.90 (m, 2H), 2.89 – 2.76 (m, 2H); ^{13}C NMR (126 MHz, Methanol-d_4) δ 142.08, 129.59, 129.40, 127.24, 87.19, 75.60, 74.00, 73.15,

71.74, 62.52, 37.27, 32.62; HRMS (ESI) m/z calcd for $C_{14}H_{20}NaO_5S$ $[(M+Na)^+]$: 323.0924, found: 323.0924.

$[\alpha]_D^{25}$: +136.0 (c = 1.0, MeOH).

(2*R*,3*S*,4*S*,5*R*,6*R*)-2-(Hydroxymethyl)-6-((4-methoxybenzyl)thio)tetrahydro-2*H*-pyran-3,4,5-triol (51):

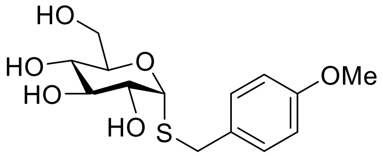

The title compound was prepared according to the **General procedure D** from **2** (1.0 equiv., 0.05 mmol), **S31** (3.0 equiv., 0.15 mmol), Hantzsch ester (2.0 equiv., 0.1 mmol), DABCO (2.5 equiv., 0.125 mmol), anhydrous DMSO (0.5 mL). The residue was subjected to flash silica gel column chromatography (eluent: $CHCl_3$/MeOH = 15/1 ~ 5/1) to give the pure product as viscous gel (^{1}H NMR yield: 90%, α:β > 95:5; isolated yield: 13.0 mg, 82%).

Glycosylation *in situ*: the title compound was prepared according to the **General procedure E** with D-glucose (1.0 equiv., 0.2 mmol), **S31** (3.0 equiv., 0.6 mmol), Hantzsch ester (2.0 equiv., 0.4 mmol), DABCO (2.5 equiv., 0.5 mmol), anhydrous DMSO (2 mL). The residue was re-dissolved in Methanol-d_4 with mesitylene (0.1 mmol) as the internal standard for ^{1}H NMR analysis (^{1}H NMR yield: 60%, α:β > 95:5; the yield was based on **2** as the limiting reagent).

^{1}H NMR (500 MHz, Methanol-d_4) δ 7.30 – 7.26 (m, 2H), 6.87 – 6.82 (m, 2H), **5.18 (d, *J* = 5.5 Hz, 1H, anomeric H)**, 3.99 (ddd, *J* = 9.9, 5.6, 2.4 Hz, 1H), 3.81 (dd, *J* = 12.0, 2.4 Hz, 1H), 3.77 (s, 3H), 3.75 – 3.65 (m, 4H), 3.55 (t, *J* = 9.3 Hz, 1H), 3.34 (d, *J* = 7.9 Hz, 1H); ^{13}C NMR (126 MHz, Methanol-d_4) δ 160.14, 131.49, 131.30, 114.74, 85.47, 75.80, 73.99, 73.02, 71.82, 62.57, 55.69, 33.57; HRMS (ESI) m/z calcd for $C_{14}H_{20}NaO_6S$ $[(M+Na)^+]$: 339.0873, found: 339.0875.

$[\alpha]_D^{25}$: +237.9 (c = 1.0, MeOH).

(2*R*,3*R*,4*S*,5*S*,6*R*)-2-(Cyclopentylthio)-6-(hydroxymethyl)tetrahydro-2*H*-pyran-3,4,5-triol (52):

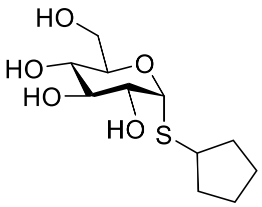

The title compound was prepared according to the **General procedure D** from **2** (1.0 equiv., 0.05 mmol), **S32** (3.0 equiv., 0.15 mmol), Hantzsch ester (2.0 equiv., 0.1 mmol), DABCO (2.5 equiv., 0.125 mmol), anhydrous DMSO (0.5 mL). The residue was subjected to flash silica gel column chromatography (eluent: $CHCl_3$/MeOH = 15/1 ~ 5/1) to give the pure product as viscous gel (^{1}H NMR yield: 46%, α:β > 95:5; isolated yield: 4.5 mg, 34%).

^{1}H NMR (500 MHz, Methanol-d_4) δ **5.36 (d, *J* = 5.5 Hz, 1H, anomeric H)**, 3.98 (ddt, *J* = 9.7, 4.8, 2.2 Hz, 1H), 3.79 (dt, *J* = 11.9, 2.3 Hz, 1H), 3.72 (dd, *J* = 12.0, 5.2 Hz, 1H), 3.68 (dd, *J* = 9.8, 5.5 Hz, 1H), 3.49 (t, *J* = 9.3 Hz, 1H), 3.34 (d, *J* = 6.8 Hz, 1H), 3.21 (p, *J* = 6.2 Hz, 1H), 2.12 – 1.99 (m, 2H), 1.80 – 1.71 (m, 2H), 1.65 – 1.53 (m, 4H); ^{13}C NMR (126 MHz, Methanol-d_4) δ 87.29, 75.70, 74.03, 73.13, 71.78, 62.50, 44.18, 35.10, 34.85, 25.70, 25.34; HRMS (ESI) m/z calcd for $C_{11}H_{20}NaO_5S$ [(M+Na)$^+$]: 287.0924, found: 287.0929.

$[\alpha]_D^{25}$: +147.1 (c = 1.0, MeOH).

(2*R*,3*R*,4*S*,5*S*,6*R*)-2-(Decylthio)-6-(hydroxymethyl)tetrahydro-2*H*-pyran-3,4,5-triol (53):

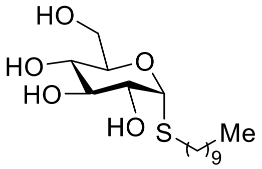

The title compound was prepared according to the **General procedure D** from **2** (1.0 equiv., 0.05 mmol), **S33** (3.0 equiv., 0.15 mmol), Hantzsch ester (2.0 equiv., 0.1 mmol), DABCO (2.5 equiv., 0.125 mmol), anhydrous DMSO (0.5 mL). The residue was subjected to flash silica gel column chromatography (eluent: $CHCl_3$/MeOH = 15/1 ~ 5/1) to give the pure product as white solid (^{1}H NMR yield: 88%, α:β > 95:5; isolated yield: 10.9 mg, 65%).

^{1}H NMR (500 MHz, Methanol-d_4) δ **5.31 (d, *J* = 5.5 Hz, 1H, anomeric H)**, 3.95 (ddd, *J* = 10.0, 5.3, 2.4 Hz, 1H), 3.79 (dd, *J* = 11.9, 2.5 Hz, 1H), 3.74 – 3.70 (m, 1H), 3.70 – 3.67 (m, 1H), 3.53 (t, *J* = 9.3 Hz, 1H), 3.34 (d, *J* = 6.9 Hz, 1H), 2.62 (dt, *J* = 12.8, 7.3 Hz, 1H), 2.55 (dd, *J* = 12.7, 7.4 Hz, 1H), 1.63 (pd, *J* = 7.3, 3.0 Hz, 2H), 1.43 – 1.38 (m, 2H), 1.32 – 1.25 (m, 12H), 0.90 (t, *J*

= 6.8 Hz, 3H); ^{13}C NMR (126 MHz, Methanol-d_4) δ 87.17, 75.63, 73.90, 73.16, 71.72, 62.49, 33.06, 31.07, 30.78, 30.71, 30.46, 30.36, 30.03, 23.73, 14.45; HRMS (ESI) m/z calcd for $C_{16}H_{32}NaO_5S$ [(M+Na)$^+$]: 359.1863, found: 359.1866.

$[\alpha]_D^{25}$: +156.4 (c = 1.0, MeOH).

(2*R*,3*R*,4*S*,5*R*,6*S*)-2-(Acetoxymethyl)-6-(((2*R*,3*R*,4*S*,5*S*,6*R*)-3,4,5-trihydroxy-6-(hydroxymethyl)tetrahydro-2*H*-pyran-2-yl)thio)tetrahydro-2*H*-pyran-3,4,5-triyl triacetate (54):

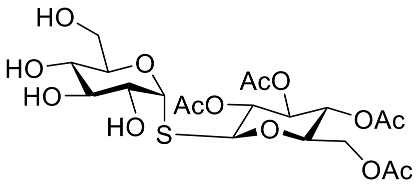

The title compound was prepared according to the **General procedure D** from **2** (1.0 equiv., 0.05 mmol), **S34** (3.0 equiv., 0.15 mmol), Hantzsch ester (2.0 equiv., 0.1 mmol), DABCO (2.5 equiv., 0.125 mmol), anhydrous DMSO (0.5 mL). The residue was subjected to flash silica gel column chromatography (eluent: $CHCl_3$/MeOH = 15/1 ~ 5/1) to give the pure product as viscous gel (^{1}H NMR yield: 34%, α:β > 95:5; isolated yield: 4.5 mg, 17%).

^{1}H NMR (500 MHz, Methanol-d_4) δ **5.57 (d, *J* = 5.3 Hz, 1H, anomeric H)**, 5.25 (t, *J* = 9.3 Hz, 1H), 5.04 (t, *J* = 9.8 Hz, 1H), 4.97 (dd, *J* = 10.2, 9.2 Hz, 1H), 4.83 (s, 1H), 4.23 (dd, *J* = 12.4, 4.3 Hz, 1H), 4.15 (dd, *J* = 12.4, 2.3 Hz, 1H), 3.98 (ddd, *J* = 10.0, 4.7, 2.4 Hz, 1H), 3.89 (ddd, *J* = 10.1, 4.3, 2.3 Hz, 1H), 3.81 (dd, *J* = 11.9, 2.5 Hz, 1H), 3.74 (dd, *J* = 12.0, 4.7 Hz, 1H), 3.70 (dd, *J* = 9.7, 5.3 Hz, 1H), 3.52 (t, *J* = 9.3 Hz, 1H), 3.41 – 3.37 (m, 1H), 2.06 (s, 3H), 2.04 (s, 3H), 2.00 (s, 3H), 1.96 (s, 3H); ^{13}C NMR (126 MHz, Methanol-d_4) δ 172.41, 171.62, 171.25, 171.23, 87.51, 83.86, 76.94, 75.51, 75.47, 74.61, 73.07, 72.67, 71.30, 69.50, 63.23, 62.27, 20.75, 20.68, 20.54; HRMS (ESI) m/z calcd for $C_{20}H_{30}NaO_{14}S$ [(M+Na)$^+$]: 549.1248, found: 549.1257.

$[\alpha]_D^{25}$: +91.0 (c = 1.0, MeOH).

6. Mechanistic studies

6.1. Glycosyl radical trap experiment

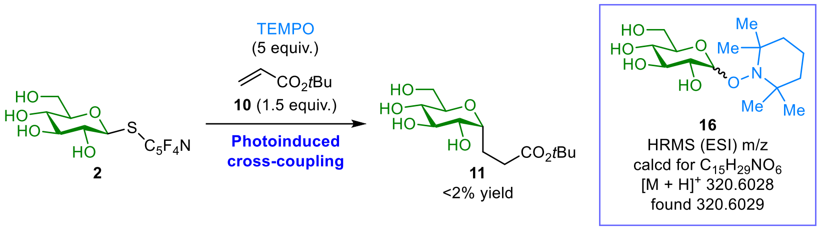

The reaction was performed according to the **General procedure D** with a slight modification. Under air, a 4 mL vial equipped with a magnetic stir bar was added **2** (1.0 equiv., 0.05 mmol), Hantzsch ester (2.0 equiv., 0.1 mmol), DABCO (2.5 equiv., 0.125 mmol) and TEMPO (5.0 equiv., 0.25 mmol). The reaction vial was then transferred into a glovebox under nitrogen atmosphere, followed by the addition of anhydrous DMSO (0.5 mL) and *tert*-butyl acrylate (1.5 equiv., 0.075 mmol). The reaction vial was sealed and taken out of the glovebox. The mixture was allowed to vigorously stir at room temperature under 12 W blue LED illumination for 24 hours. After the reaction was complete, DMSO was evaporated by attaching the oil pump to the rotary evaporator and the residue was re-dissolved in Methanol-d_4 for ^1H NMR and HRMS analysis.

Mass Spectrum SmartFormula Report

**Sample Name**	yiw-739	**Data File**	D:\MassHunter\Data\Chemistry\2023\202304\20230425\yiw-739.d
**Instrument Name**	Agilent 6546 LC-QTOF	**IRM Calibration Status**	Success
**Acq Method**	MS Scan_union-1.m	**Acquired Time**	25/4/2023 11:29:14 AM (UTC+08:00)
**Comment**	A/P Koh Ming Joo	**Operator**	WLK

**Meas. m/z**	**#**	**Formula**	**Calc. Mass**	**Err [ppm]**
320.2069	1	C15 H30 N O6	320.2067	0.62

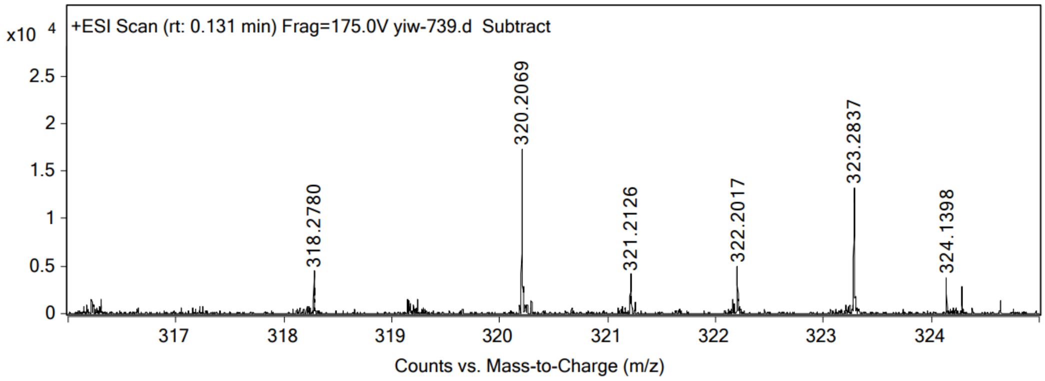

Page 1 of 1 Printed at 12:16 PM on 25-Apr-2023

6.2. Ultraviolet/visible (UV/vis) spectroscopy studies

Figure S1. UV/vis spectra of different reaction components

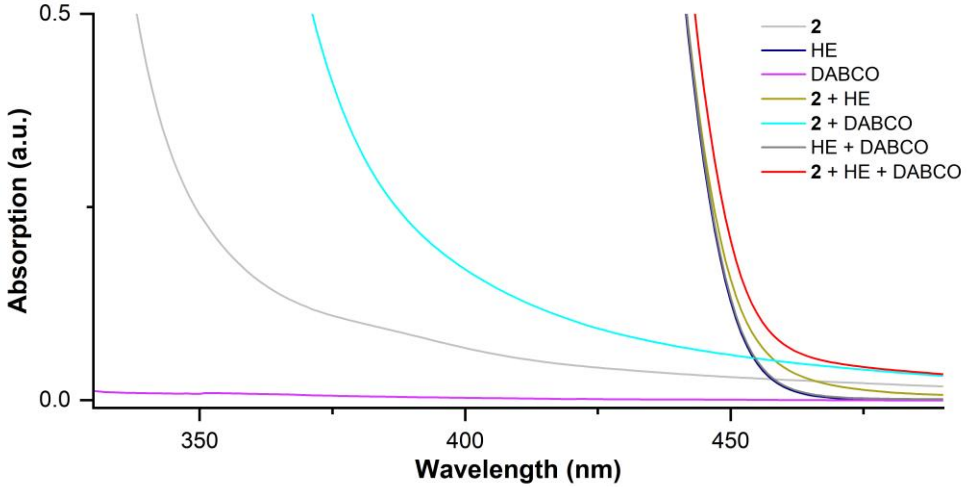

Reactant combinations: the concentrations of all compounds are half of those of the standard reaction.

Light grey trace: **2** (0.05 N in DMSO)

Blue trace: HE (0.05 N in DMSO)

Purple trace: DABCO (0.05 N in DMSO)

Brown trace: **2** (0.05 N in DMSO) + HE (0.05 N in DMSO)

Cyan trace: **2** (0.05 N in DMSO) + DABCO (0.05 N in DMSO)

Dark grey trace: HE (0.05 N in DMSO) + DABCO (0.05 N in DMSO)

Red trace: **2** (0.05 N in DMSO) + HE (0.05 N in DMSO) + DABCO (0.05 N in DMSO)

6.3. Cyclic voltammetry studies

Cyclic voltammetry was carried out in a glass cell. A glassy carbon disk electrode (diameter is 3.0 mm, PCTFE shroud) was used as a working electrode. A platinum wire was used as a counter electrode. SCE electrode was used as a reference electrode. Electrolyte: 0.1 M Bu_4NPF_6 in MeCN; Concentration of a sample: $1.0*10^{-3}$ M.

Figure S2. Cyclic voltammogram of **2**

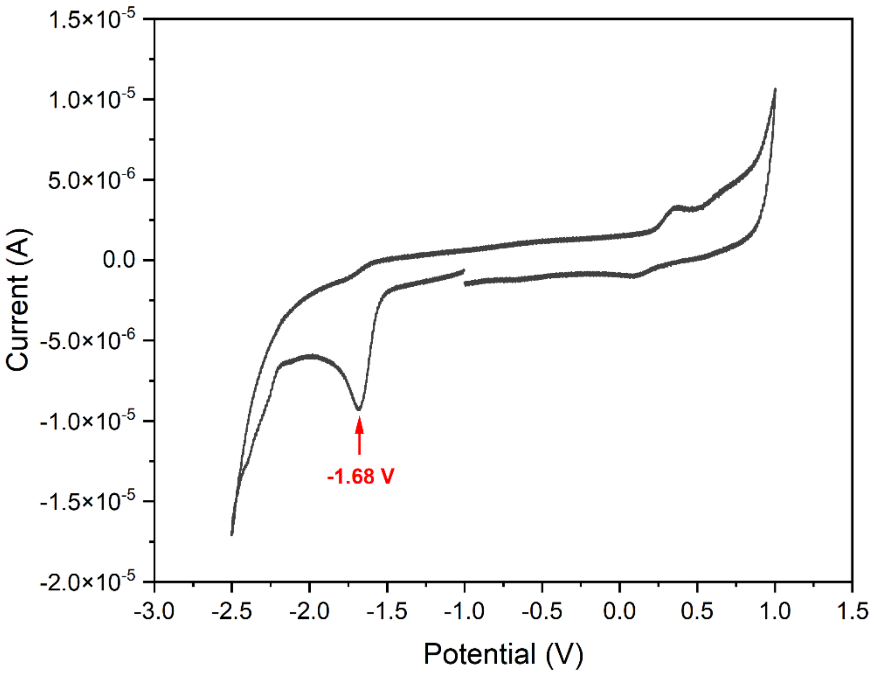

Figure S3. Cyclic voltammogram of **6**

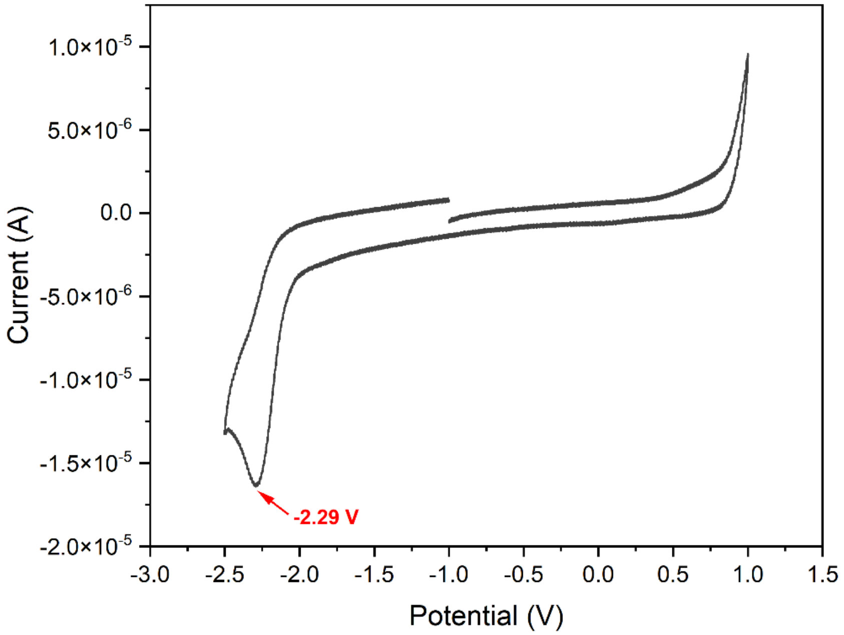

Figure S4. Cyclic voltammogram of **7**

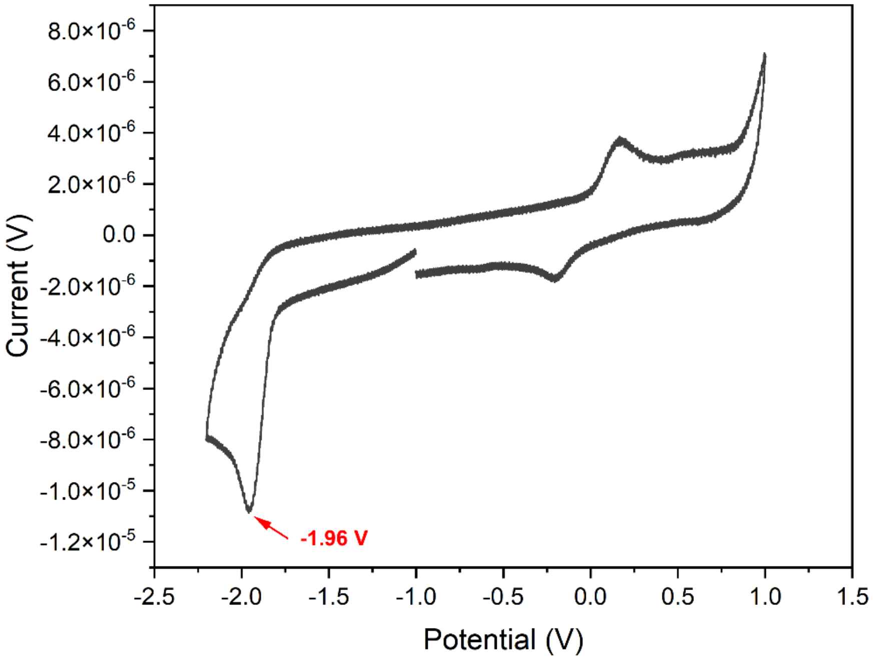

Figure S5. Cyclic voltammogram of **8**

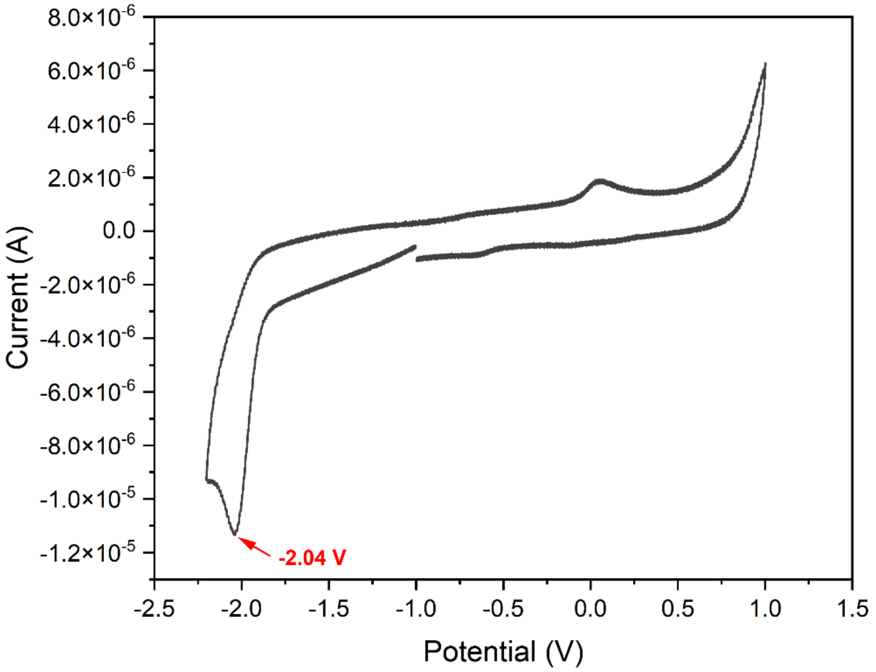

Figure S6. Cyclic voltammogram of **9**

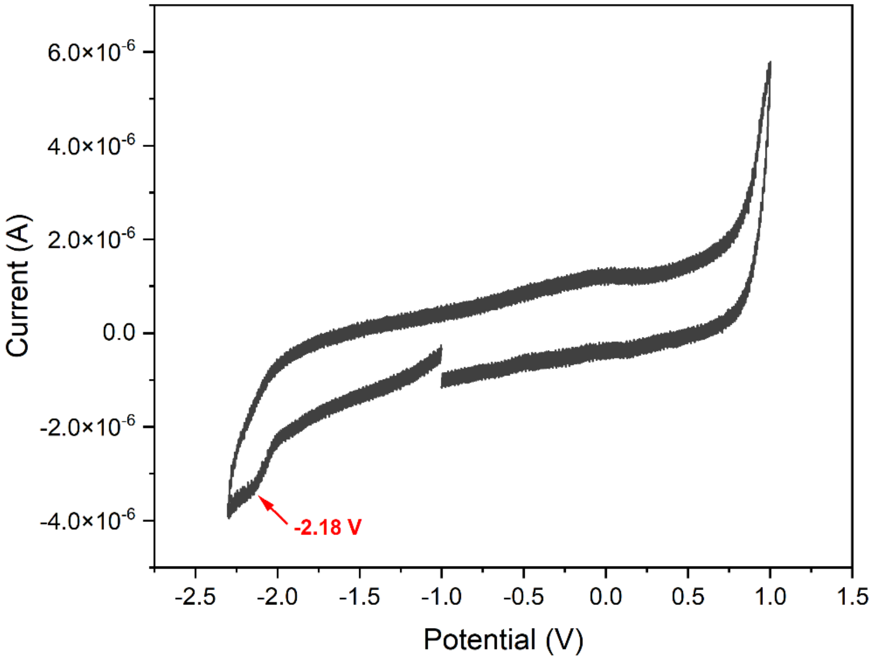

6.4. Proposed mechanism

Figure S7. Proposed pathways for sugar activation and photoinduced glycosylation

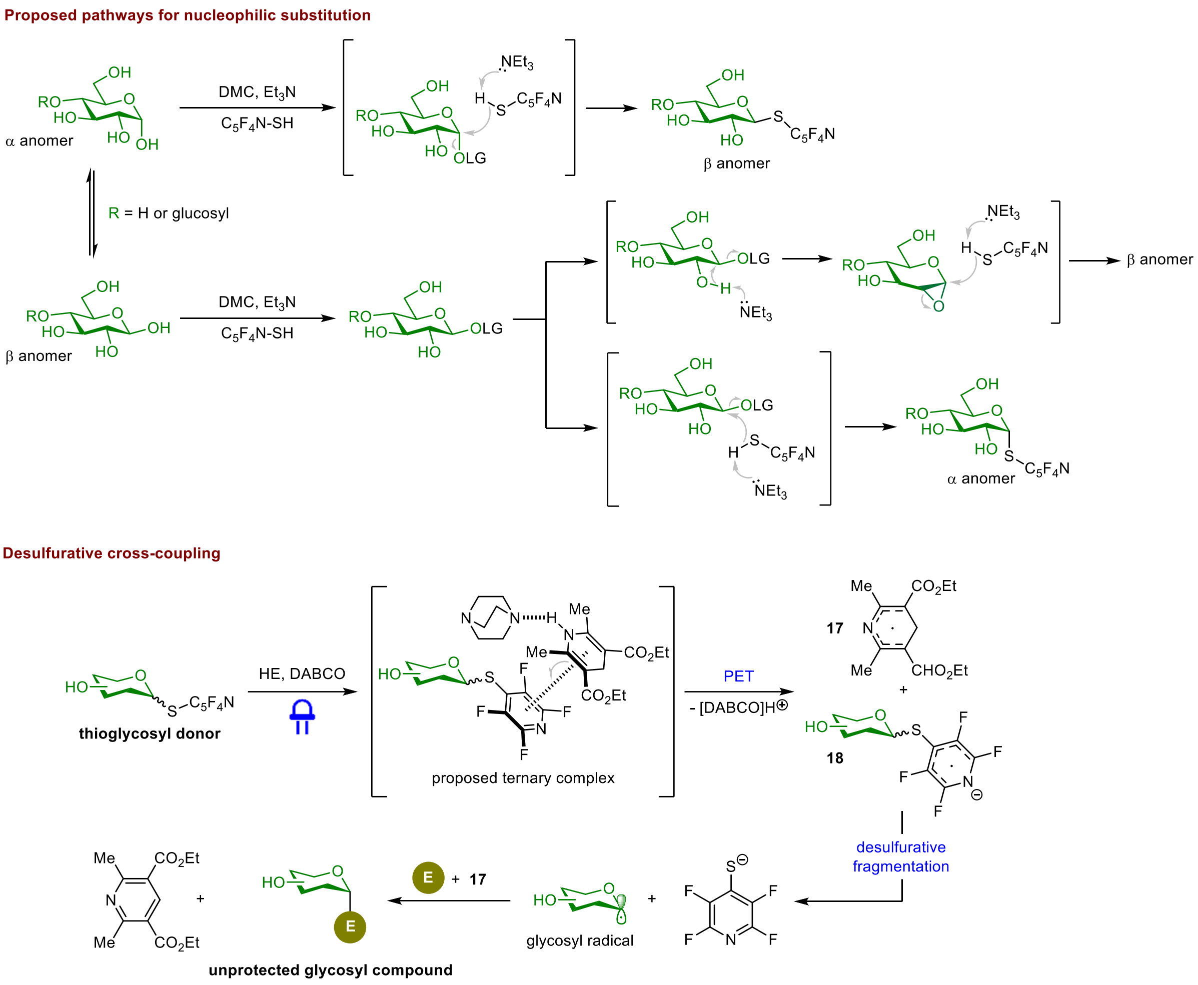

7. X-ray crystallographic data

X-ray structure of **S2** (CCDC 2263895)

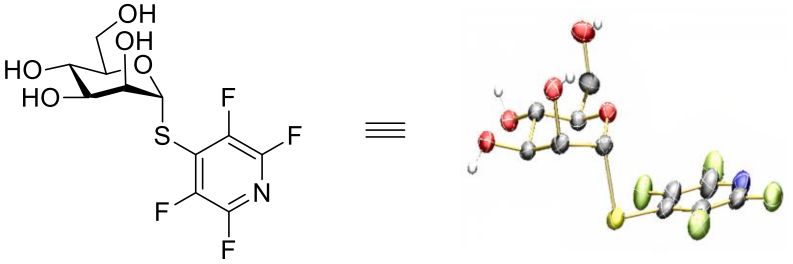

Table S5. Crystal data and structure refinement for **S2**

Identification code	**S2**	
Empirical formula	$C_{11}H_{13}F_4NO_6S$	
Formula weight	363.28	
Temperature	100(2) K	
Wavelength	1.54178 Å	
Crystal system	Monoclinic	
Space group	$P2_1$	
Unit cell dimensions	a = 7.7486(3) Å	a= 90°.
	b = 36.1899(15) Å	b= 110.563(2)°.
	c = 10.9782(5) Å	g = 90°.
Volume	2882.4(2) $Å^3$	
Z	8	
Density (calculated)	1.674 Mg/m^3	
Absorption coefficient	2.763 mm^{-1}	
F(000)	1488	
Crystal size	0.118 x 0.114 x 0.094 mm^3	
Theta range for data collection	2.442 to 70.619°.	
Index ranges	-8<=h<=9, -44<=k<=44, -13<=l<=13	
Reflections collected	93396	
Independent reflections	10960 [R(int) = 0.0723]	
Completeness to theta = 67.679°	99.8 %	
Absorption correction	Semi-empirical from equivalents	
Max. and min. transmission	0.6918 and 0.5930	
Refinement method	Full-matrix least-squares on F^2	
Data / restraints / parameters	10960 / 454 / 1005	
Goodness-of-fit on F^2	1.035	

Final R indices [I>2sigma(I)]	R1 = 0.0494, wR2 = 0.1317
R indices (all data)	R1 = 0.0551, wR2 = 0.1325
Absolute structure parameter	-0.001(7)
Extinction coefficient	n/a
Largest diff. peak and hole	0.523 and -0.298 e.Å^{-3}

8. Glycosylation of proteins

8.1. Preparation of proteins

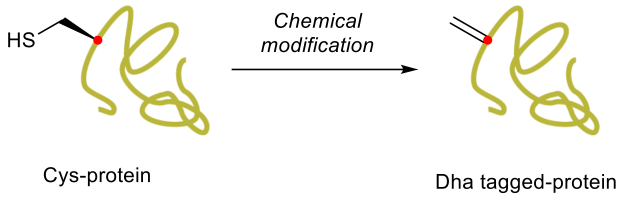

Dha-tagged proteins were generated and purified according to reported procedures.[11-12]

Histone eH3-Dha9

ARTKQTARDhaSTGGKAPRKQLATKAARKSAPATGGVKKPHRYRPGTVALREIRRYQKST ELLIRKLPFQRLVREIAQDFKTDLRFQSSAVMALQEAAEAYLVGLFEDTNLAAIHAKRVTI MPKDIQLARRIRGERAGGDYKDDDDKSAAGGYPYDVPDYA

Calculated mass = 17686

Observed mass = 17686

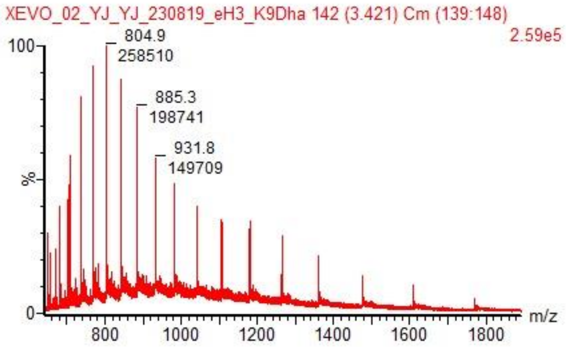

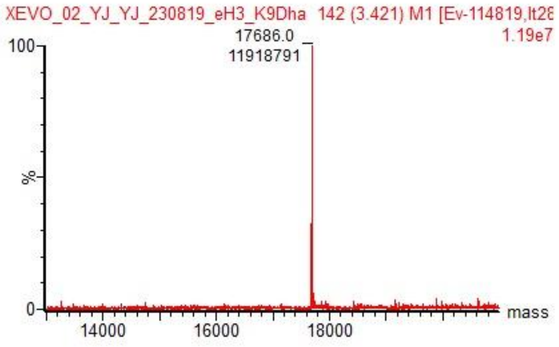

Histone H3-Dha10

ARTKQTARKDhaTGGKAPRKQLATKAARKSAPATGGVKKPHRYRPGTVALREIRRYQKS TELLIRKLPFQRLVREIAQDFKTDLRFQSSAVMALQEASEAYLVALFEDTNLAAIHAKRVT IM PKDIQLARRIRGERA

Calculated mass = 15221

Observed mass = 15221

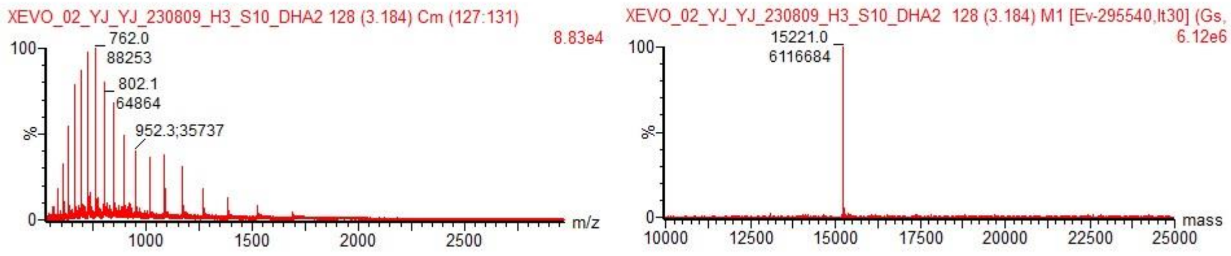

Histone H3-TEV-Dha2

ADhaENLYFQGTKQTARKSTGGKAPRKQLATKAARKSAPATGGVKKPHRYRPGTVALRE
IRRYQKSTELLIRKLPFQRLVREIAQDFKTDLRFQSSAVMALQEASEAYLVALFEDTNLAA
IHAKRVTIMPKDIQLARRIRGERA

Calculated mass = 16004

Observed mass = 16004

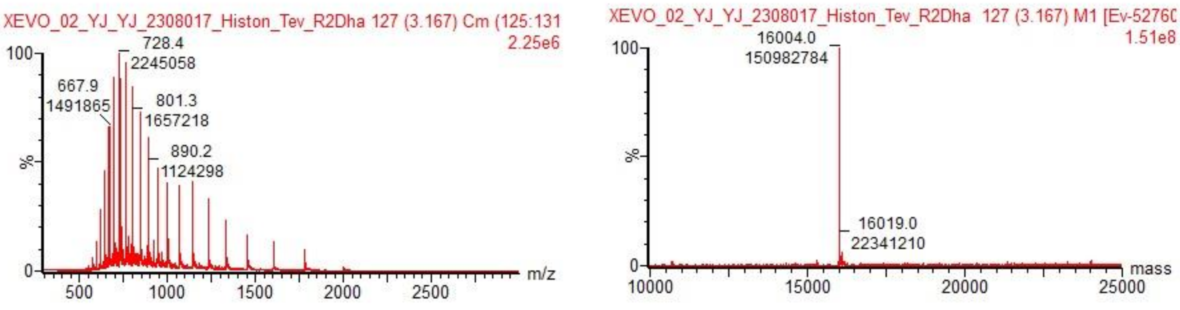

PanC-Dha44

TIPAFHPGELNVYSAPGDVADVSRALRLTGRRVMLVPTMGALDhaEGHLALVRAAKRVP
GSVVVVSIFVNPMQFGAGEDLDAYPRTPDDDLAQLRAEGVEIAFTPTTAAMYPDGLRTT
VQPGPLAAELEGGPRPTHFAGVLTVVLKLLQIVRPDRVFFGEKDYQQLVLIRQLVADFNL
DVAVVGVPTVREADGLAMSSRNRYLDPAQRAAAVALSAALTAAAHAATAGAQAALDAA
RAVLDAAPGVAVDYLELRDIGLGPMPLNGSGRLLVAARLGTTRLLDNIAIEIGTFAGTDRP
DGYRA ILESHWRNKLAAALEHHHHHH

Calculated mass = 33998

Observed mass = 33998

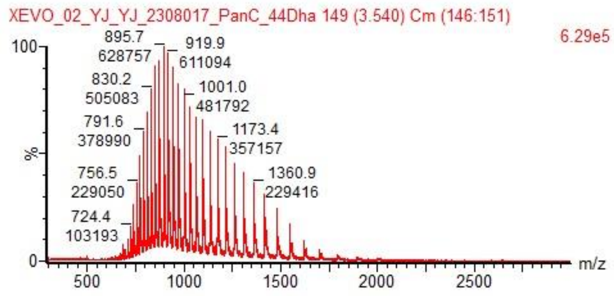

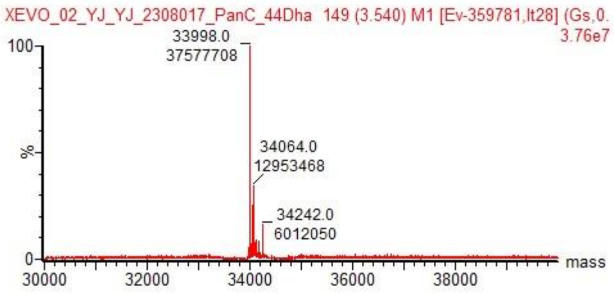

PstS-Dha57

MEASLTGAGATFPAPVYAKWADTYQKETGNKVNYQGIGSSGGVKQIIANTVDFGAS<u>Dha</u>
APLSDEKLAQEGLFQFPTVIGGVVLAVNIPGLKSGELVLDGKTLGDIYLGKIKKWDDEAI
AKLNPGLKLPSQNIAVVRRADGSGTSFVFTSYLAKVNEEWKNNVGTGSTVKWPIGLGG
KGNDGIAAFVQRLPGAIGYVEYAYAKQNNLAYTKLISADGKPVSPTEENFANAAKGAD
WSKTFAQDLTNQKGEDAWPITSTTFILIHKDQKKPEQGTEVLKFFDWAYKTGAKQANDL
DYASLPDSVVEQVRAAWKTNIKDSSGKPLY

Calculated mass = 34507

Observed mass = 34507

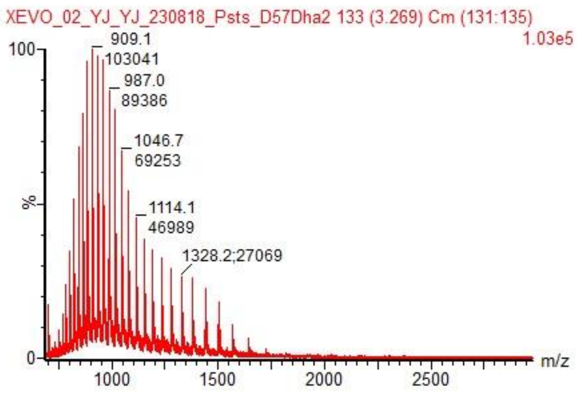

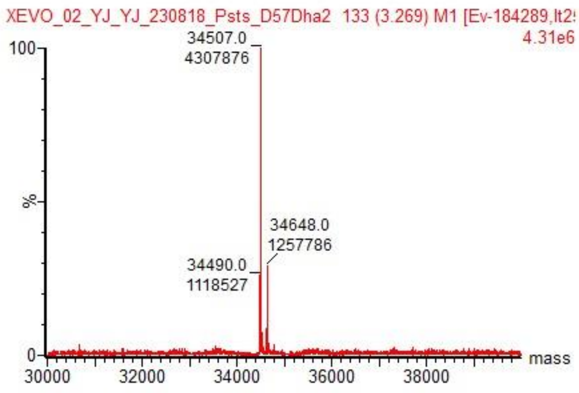

SsβG-Dha7:C344S:E387C

MYSFPN<u>Dha</u>FRFGWSQAGFQSEMGTPGSEDPNTDWYKWVHDPENMAAGLVSGDLPEN
GPGYWGNYKTFHDNAQKMGLKIARLNVEWSRIFPNPLPRPQNFDESKQDVTEVEINENE
LKRLDEYANKDALNHYREIFKDLKSRGLYFILNMYHWPLPLWLHDPIRVRRGDFTGPSG
WLSTRTVYEFARFSAYIAWKFDDLVDEYSTMNEPNVVGGLGYVGVKSGFPPGYLSFELS
RRAMYNIIQAHARAYDGIKSVSKKPVGIIYANSSFQPLTDKDMEAVEMAENDNRWWFFD
AIIRGEITRGNEKIVRDDLKGRLDWIGVNYYTRTVVKRTEKGYVSLGGYGHGSERNSVS

LAGLPTSDFGWEFFPEGLYDVLTKYWNRYHLYMYVTCNGIADDADYQRPYYLVSHVYQ

VHRAINSGADVRGYLHWSLADNYEWASGFSMRFGLLKVDYNTKRLYWRPSALVYREIA

TNGAITDEIEHLNSVPPVKPLRHHHHHHH

Calculated mass = 57454

Observed mass = 57454

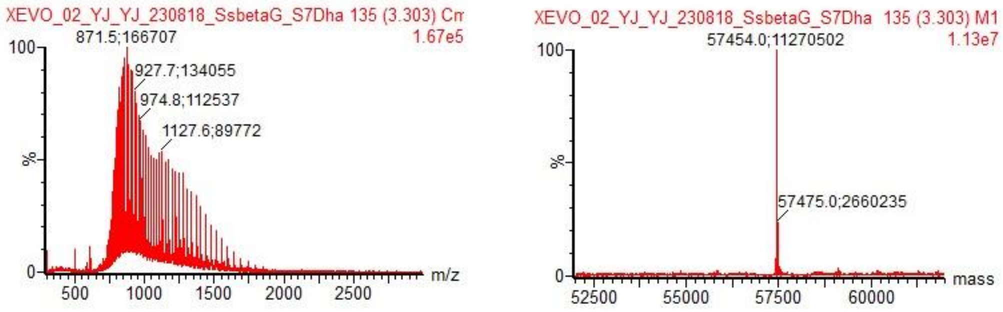

8.2. Optimization of protein glycosylation conditions

Table S6. Screening of activator

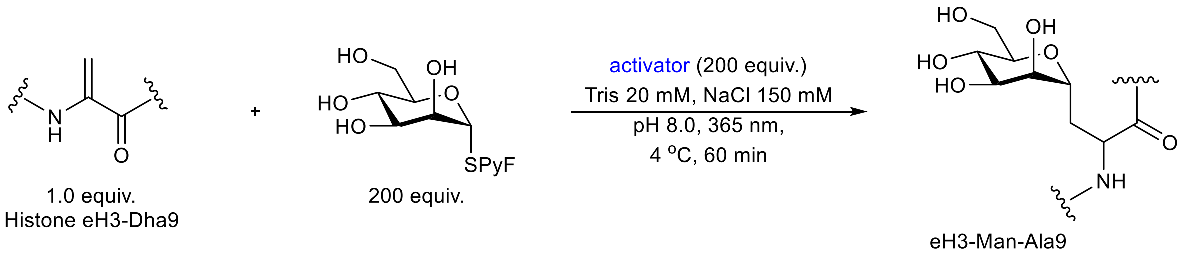

Entry	activator	Conversion
1	A1	< 10%
2	A2	< 10%
3	A3	27%
4	A4	21%
5	A5	52%
6	A6	77%

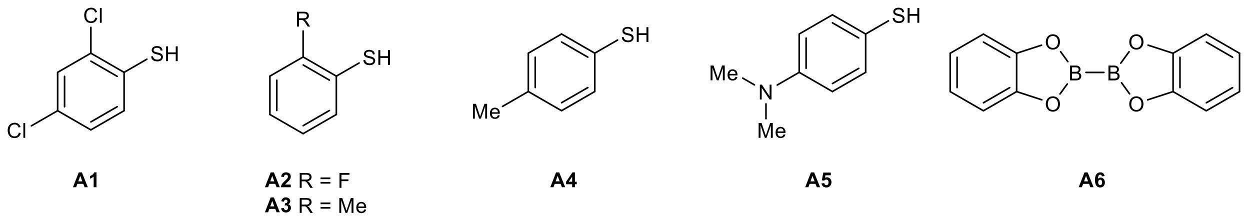

Table S7. Screening of illumination wavelength

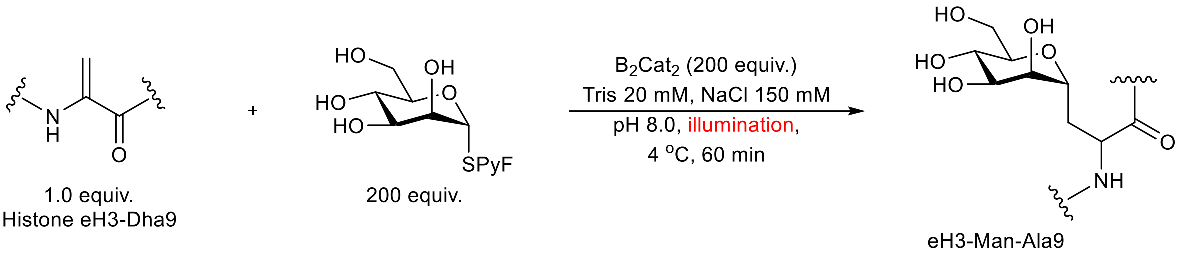

Entry	Wavelength (nm)	Conversion
1	365	77%
2	385	78%
3	405	46%
4	420	30%
5	445	18%

Table S8. Further screening of illumination wavelength

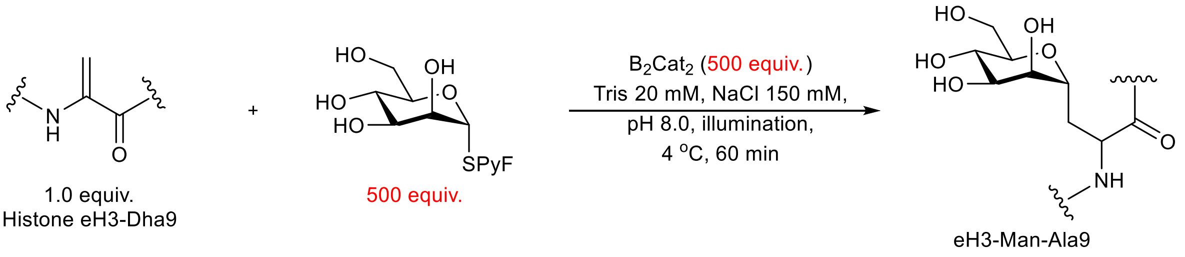

Entry	Wavelength (nm)	Conversion
1	365	47%
3	405	64%
4	420	83%
5	445	53%

8.3. General procedure for photoinduced glycosylation of proteins

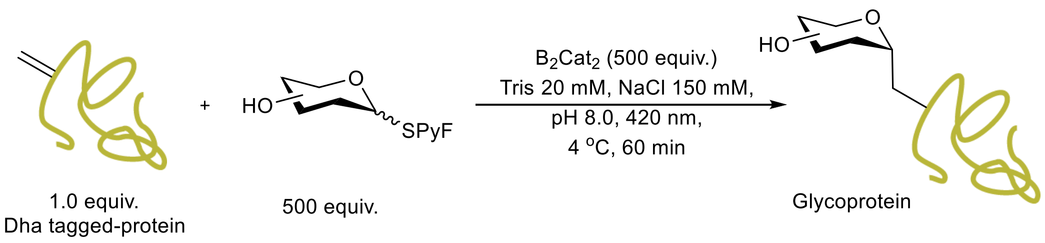

General procedure F: glycosylation of proteins

To a solution of Dha-tagged Protein (50 µM, 30 µL, 1.00 equiv.) in TBS buffer (Tris 20 mM, NaCl 150 mM, pH = 8.0) was added to a solution of sugar donor (500 equiv., 0.2 M in H_2O) and B_2Cat_2 (500 equiv., 1 M in DMSO) in glovebox. The mixture was shaken for 60 min at 4 ^{o}C under 420 nm illumination. After completed, the reaction mixture was subjected to LC-MS for analysis. (**Note: 0.5 M in DMSO was used for GlcNAc donor*). Conversions were calculated based on total ion count of thresholded signal peaks indicated in line with prior calibrations. Perturbed and unperturbed impurities are noted.

8.4. Glycoproteins mass spectrometry analysis

Histone eH3-Man-Ala9

83% conversion; Calculated mass = 17850; Observed mass = 17850

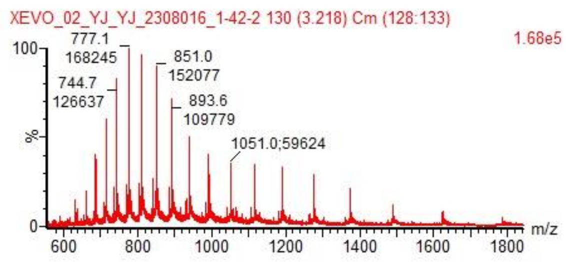

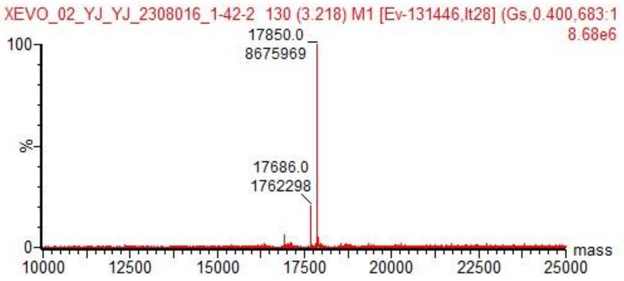

In-situ glycosylation (without isolating sugar donor): 82% conversion; Observed mass = 17850.

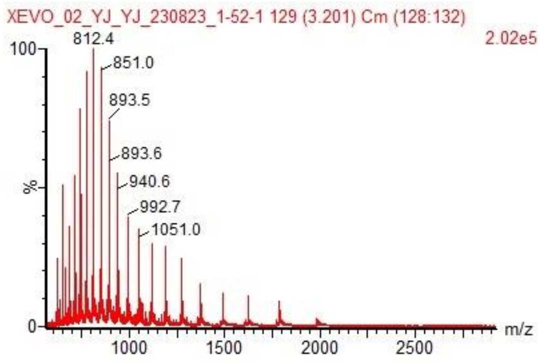

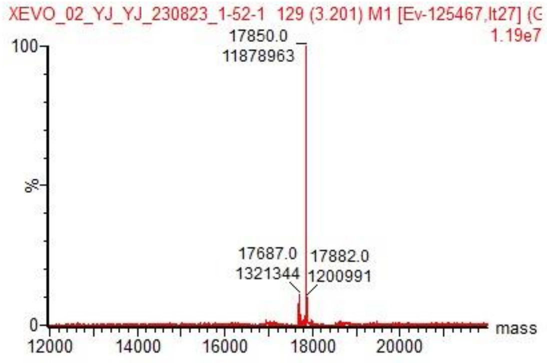

Histone eH3-Gal-Ala9

88% conversion; Calculated mass = 17850; Observed mass = 17850

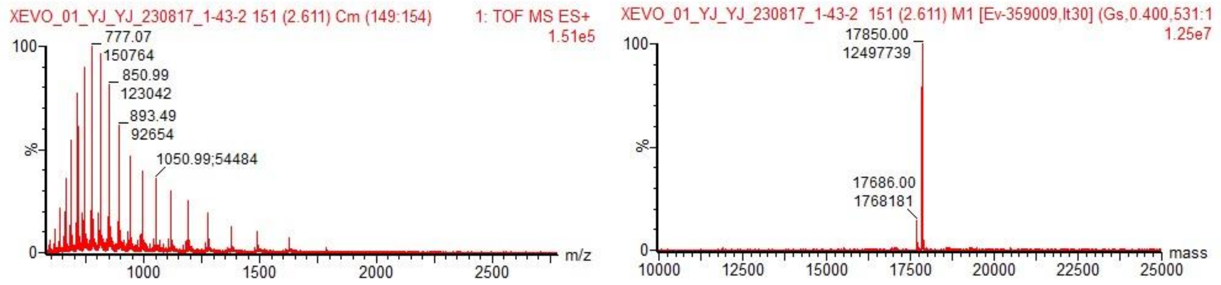

In-situ glycosylation (without isolating sugar donor): 90% conversion; Observed mass = 17850.

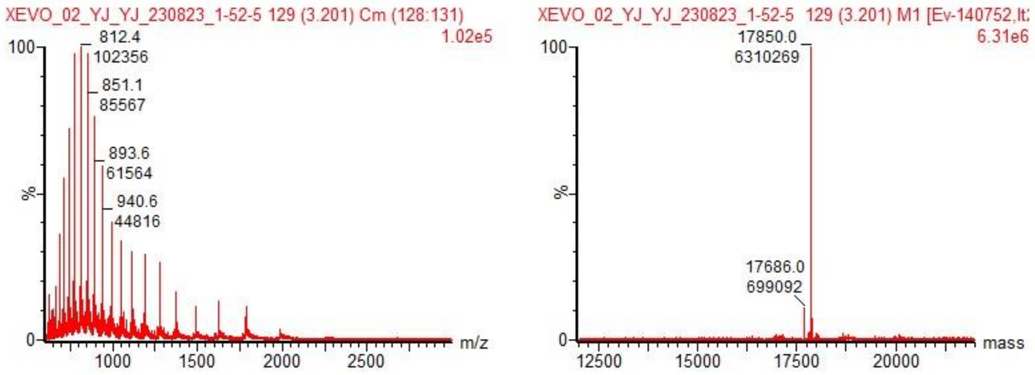

Histone eH3-GlcNAc-Ala9

70% conversion; Calculated mass = 17891; Observed mass = 17891

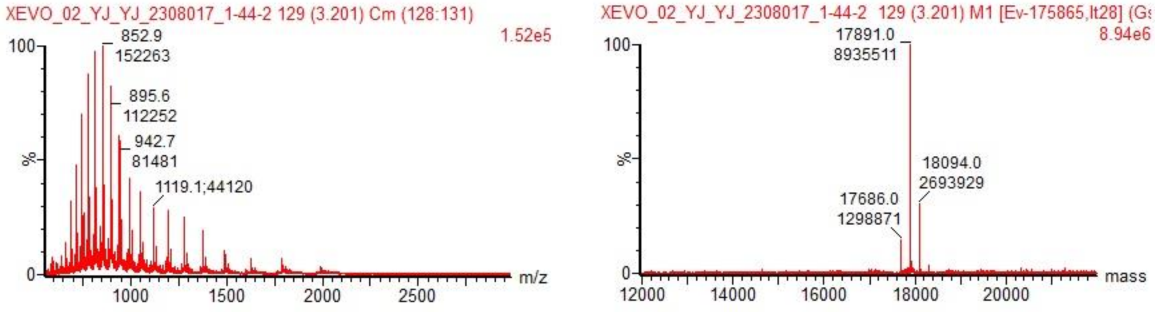

Histone H3-Man-Ala10

80% conversion; Calculated mass = 15385; Observed mass = 15385

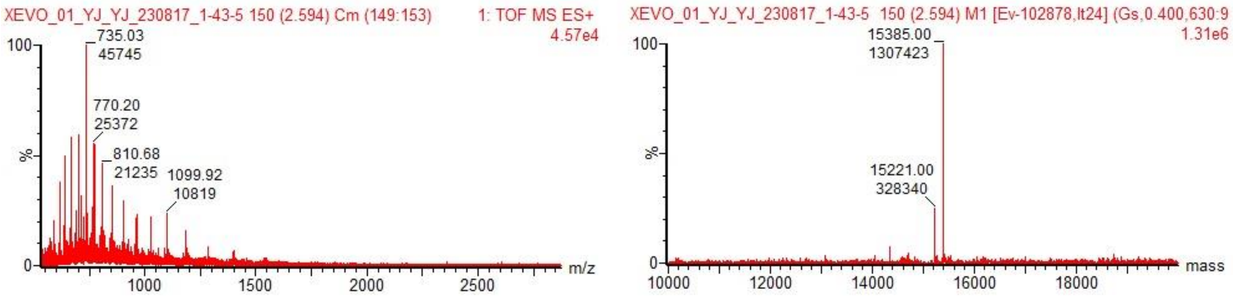

Histone H3-Gal-Ala10

83% conversion; Calculated mass = 15385; Observed mass = 15385

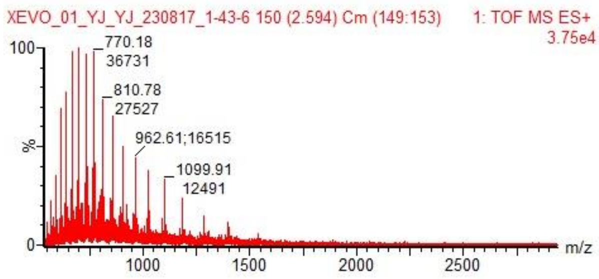

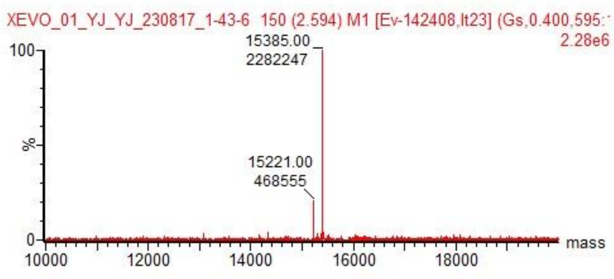

Histone H3-GlcNAc-Ala10

77% conversion; Calculated mass = 15426; Observed mass = 15426

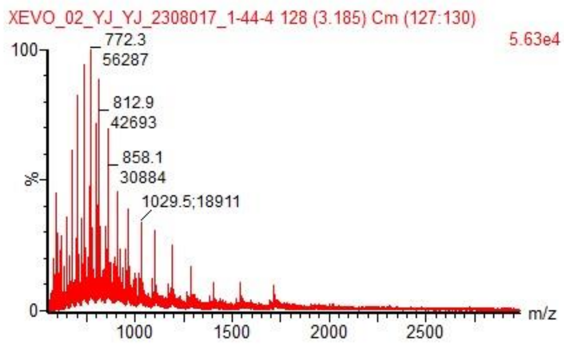

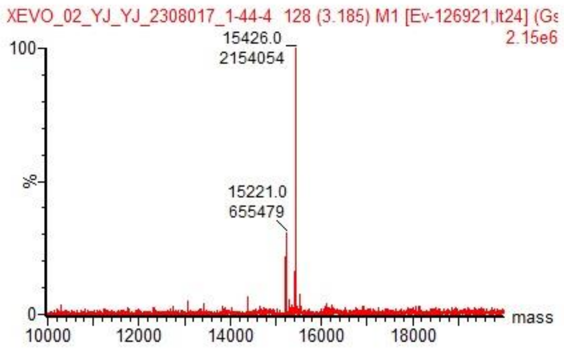

Histone H3-TEV-Man-Ala2

52% conversion; Calculated mass = 16167; Observed mass = 16167

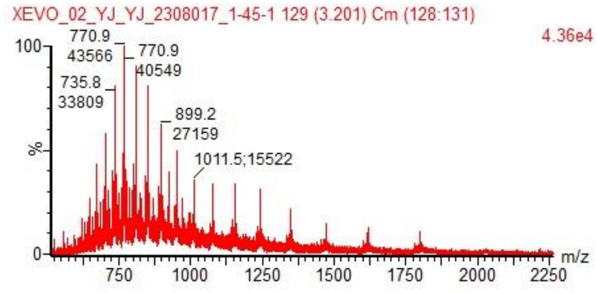

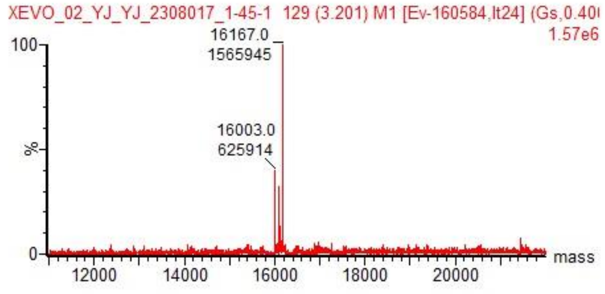

Histone H3-TEV-Gal-Ala2

56% conversion; Calculated mass = 16167; Observed mass = 16167

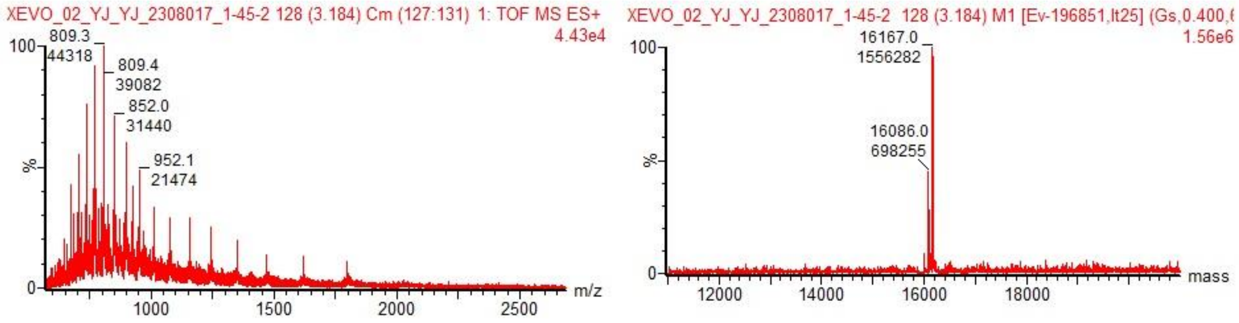

Histone H3-TEV-GlcNAc-Ala2

64% conversion; Calculated mass = 16208; Observed mass = 16208

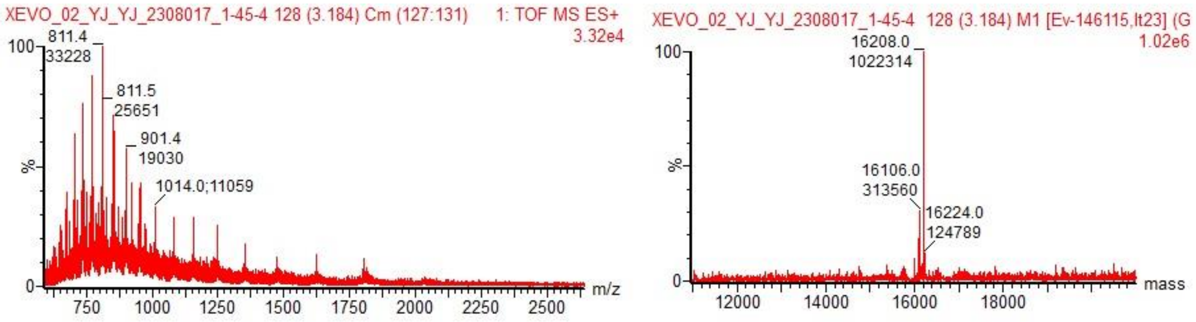

PanC-Man-Ala44

81% conversion; Calculated mass = 34162; Observed mass = 34162

(Peak mass 34064 and 34242 are unperturbed impurities from PanC-Dha44)

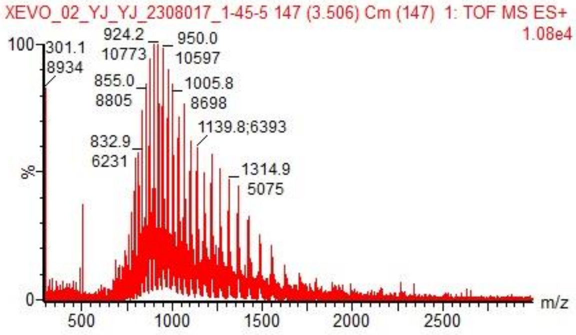

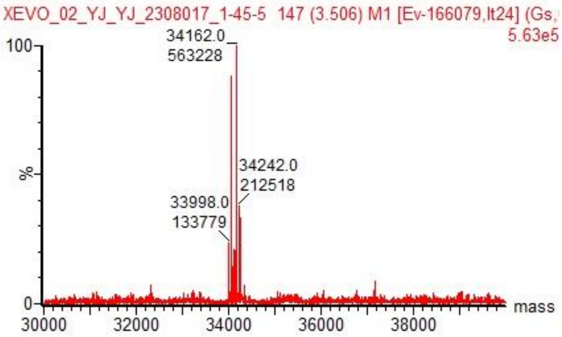

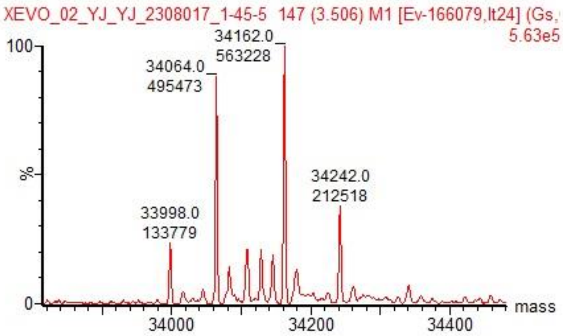

PanC-GlcNAc-Ala44

78% conversion; Calculated mass = 34203; Observed mass = 34203

(Peak mass 34063 and 34242 are unperturbed impurities from PanC-Dha44)

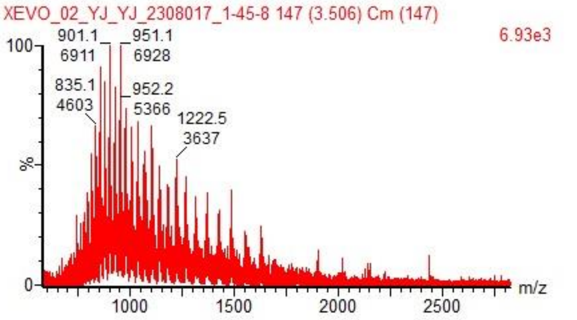

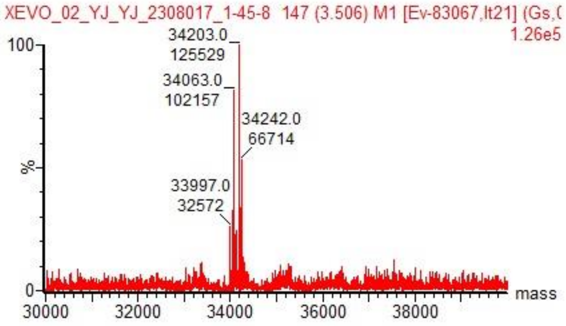

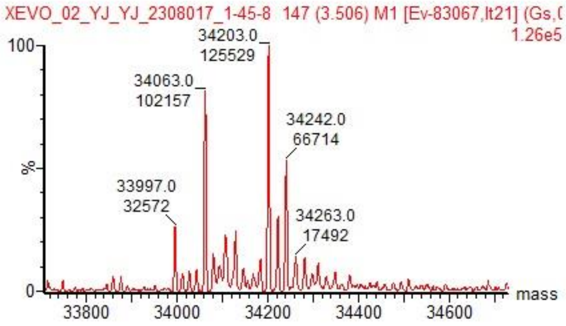

PstS-Man-Ala57

66% conversion; Calculated mass = 34671; Observed mass = 34672

(Peak mass 34811 ≈ 34648 + 164, perturbed impurity from PstS-Dha57)

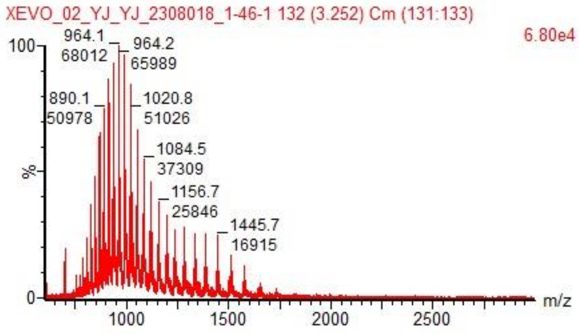

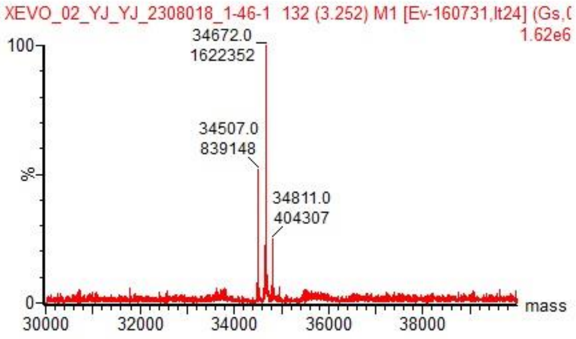

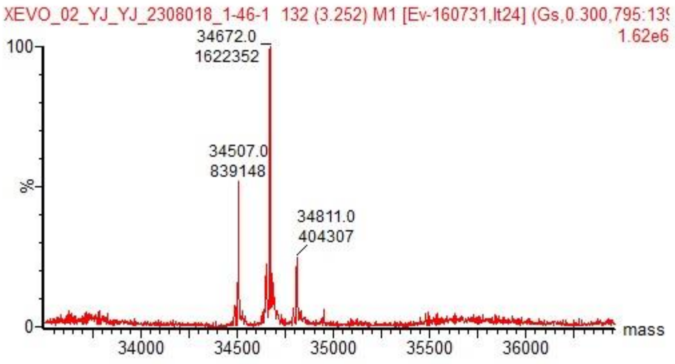

In-situ glycosylation (without isolating sugar donor): 77% conversion; Observed mass = 34671.

(Peak mass 34812 = 34648 + 164, perturbed impurity from PstS-Dha57)

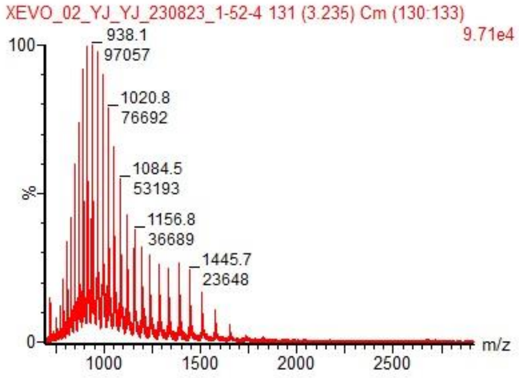

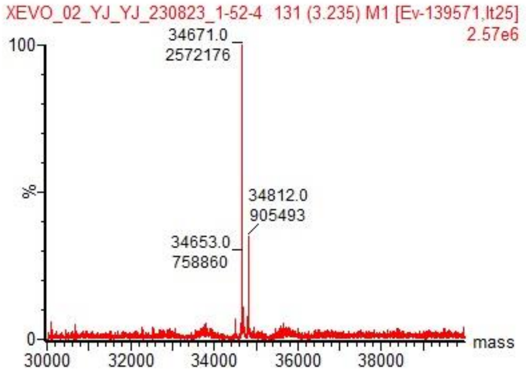

PstS-Gal-Ala57

80% conversion; Calculated mass = 34671; Observed mass = 34671

(Peak mass 34811 ≈ 34648 + 164, perturbed impurity from PstS-Dha57)

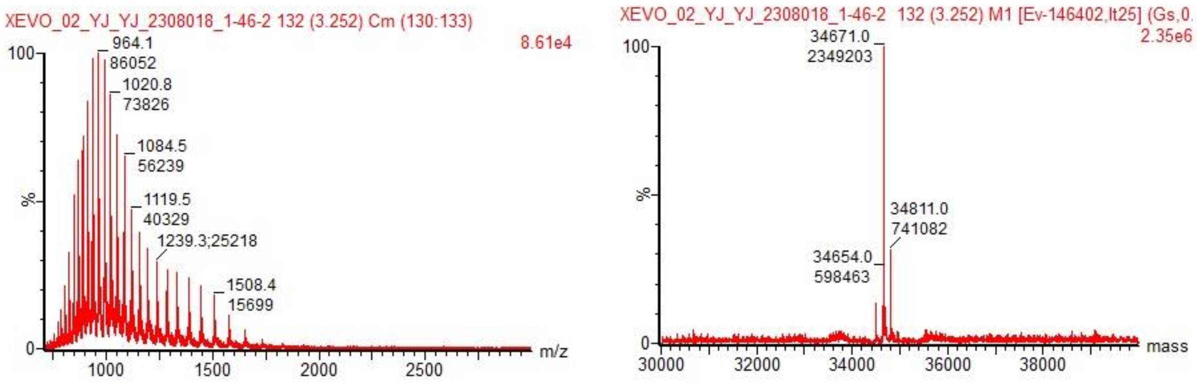

In-situ glycosylation (without isolating sugar donor): 75% conversion; Observed mass = 34671.

(Peak mass 34812 = 34648 + 164, perturbed impurity from PstS-Dha57)

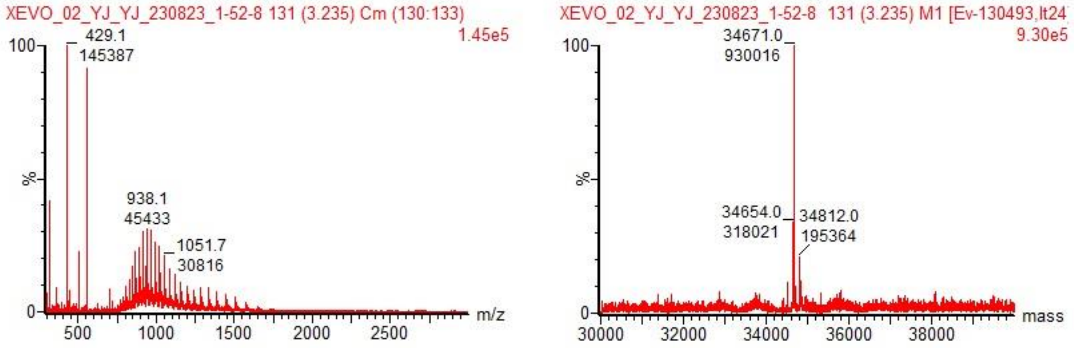

PstS-GlcNAc-Ala57

78% conversion; Calculated mass = 34712; Observed mass = 34712

(Peak mass 34853 = 34648 + 205, perturbed impurity from PstS-Dha57)

(Peak mass 34914 ≈ 34507 + 205 + 203; ~ 5% two sugar unit addition glycoprotein was detected, which we ascribe to non-specifical glycosylation of lysine residues; see Figure S13, Table S9 and Figure S15 for details)

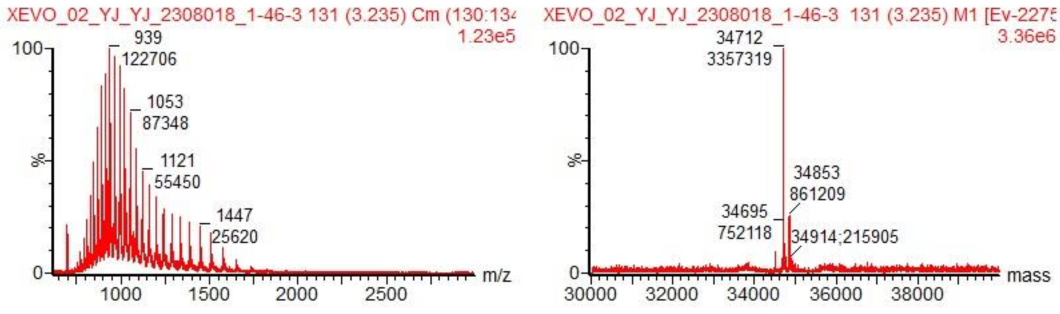

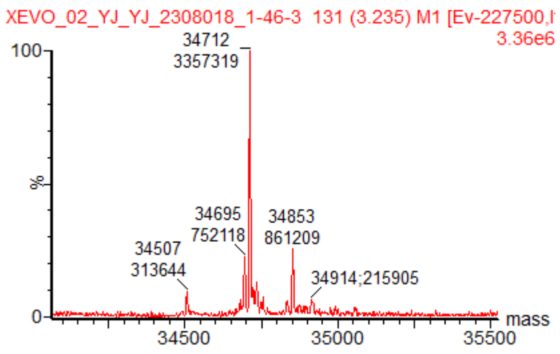

SsβG-GlcNAc-Ala7

72% conversion; Calculated mass = 57659; Observed mass = 57659

(Peak mass 57681 ≈ 57475 + 205, perturbed impurity from SsβG-Dha7)

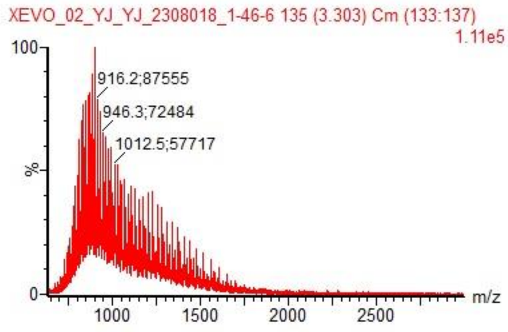

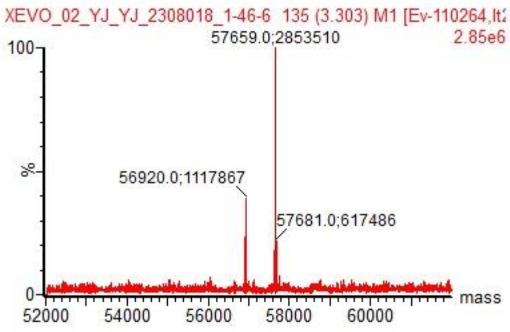

8.5. LC-MS/MS analysis of glycoproteins

Methods:

Sample proteolysis: For in-solution proteolytic digestion, samples were buffer-exchanged into 100 mM ammonium bicarbonate (urea was added to SsβG samples at 6M final concentration), reduced with 10 mM tris(2-carboxyethyl)phosphine (Thermo Fisher) for 30 min at room temperature and alkylated with 30 mM 2-chloroacetamide (Sigma Aldrich) at room temperature for 30 min in the dark. LysC (Fujifilm Wako) was added to histone sample solutions and trypsin (Pierce) was added to the solutions of other proteins for a 6-hour incubation at 37°C with 1:25 protease:protein ratio (w/w). SsβG sample was diluted to 1.3M urea prior to adding trypsin. The samples were desalted by Oasis HLB cartridges (Waters) and reconstituted in water containing 5% formic acid, 5% DMSO right before the LC-MS analysis.

LCMS: Samples were subjected to LC-MS/MS using an UltiMate 3000 nanoUHPLC system (Thermo Fisher Scientific) coupled to an Orbitrap QExactive (Thermo Fisher Scientific). The peptides were trapped on a C18 PepMap100 pre-column (300 µm i.d. x 5 mm, 100 Å, Thermo Fisher Scientific) using solvent A (0.1% formic acid in water), then separated on an in-house packed analytical column (50 µm i.d. x 50 cm in-house packed with ReproSil Gold 120 C18, 1.9 µm, Dr. Maisch GmbH) and utilised a 15 minute gradient (12% to 40%B where B is 0.1% formic acid in acetonitrile) at a flow rate of 100 nL/min. Full scan MS spectra were acquired in the Orbitrap (scan range 350-1400 m/z, resolution 70000, AGC target 3e6). Five most abundant peptides were selected each round for stepped HCD fragmentation using 20, 30, and 40% normalised collision energy, and fragmentation products were mass-analysed in the Orbitrap (scan range 200-2000 m/z, resolution 17500, AGC target 5e4, maximum injection time 128 ms).

Data analysis: Spectra were searched using FragPipe (v20.0) MSFragger 3.8[13] with standard 'open' search settings against corresponding bespoke. FASTA files containing proteins of interest and potential contaminants. Data was filtered using the inbuilt tools within FragPipe to an FDR of below 1%. Modified peptides were discerned by filtering the resulting dataset using the expected changes in mass caused by each modification.

Figure S8. Fragmentation spectrum of [Acetyl-AA(Hex)ENLYFQGTK]$^{2+}$ originating from TEV-Histone-H3-Man-Ala2.

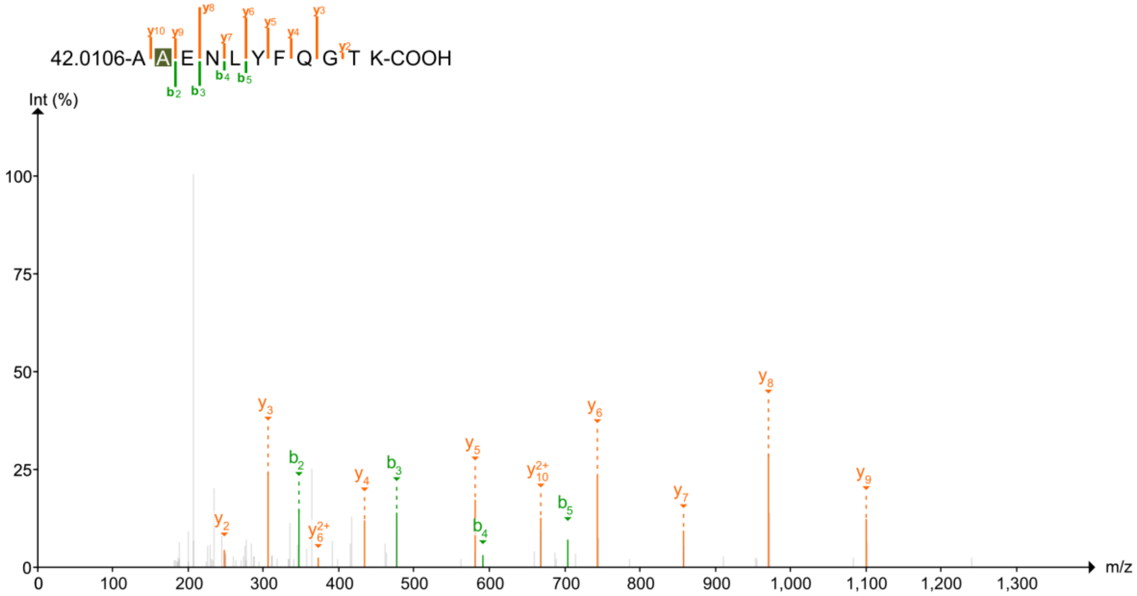

Figure S9. Fragmentation spectrum of [Acetyl-AA(Hex)ENLYFQGTK]$^{2+}$ originating from TEV-Histone-H3-Gal-Ala2.

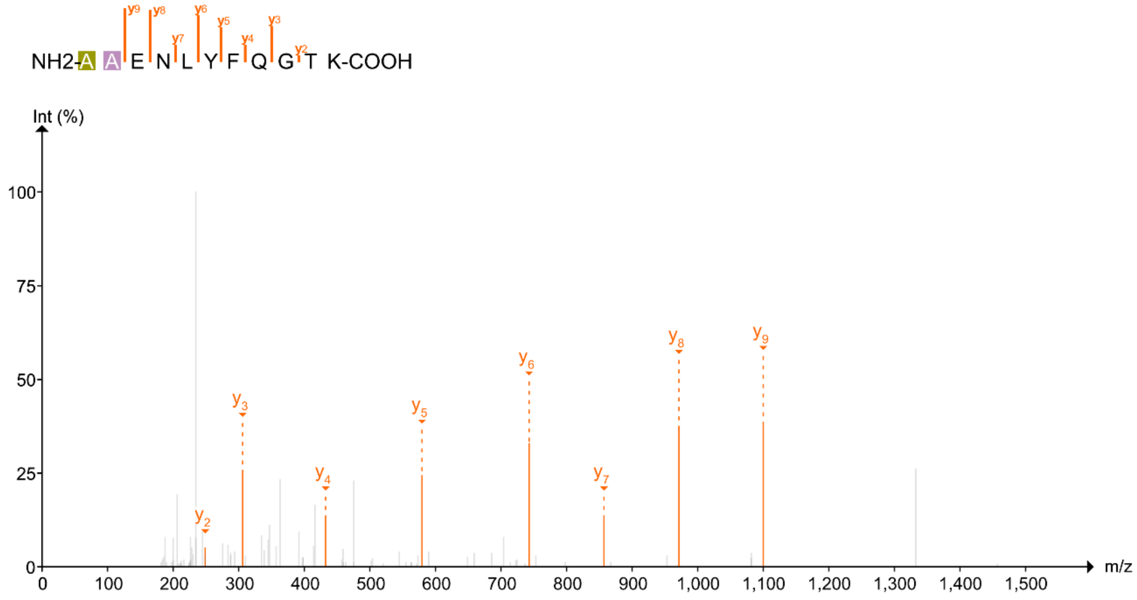

Figure S10. Fragmentation spectrum of [Acetyl-AA(HexNAc)ENLYFQGTK]$^{2+}$ originating from TEV-Histone-H3-GlcNAc-Ala2.

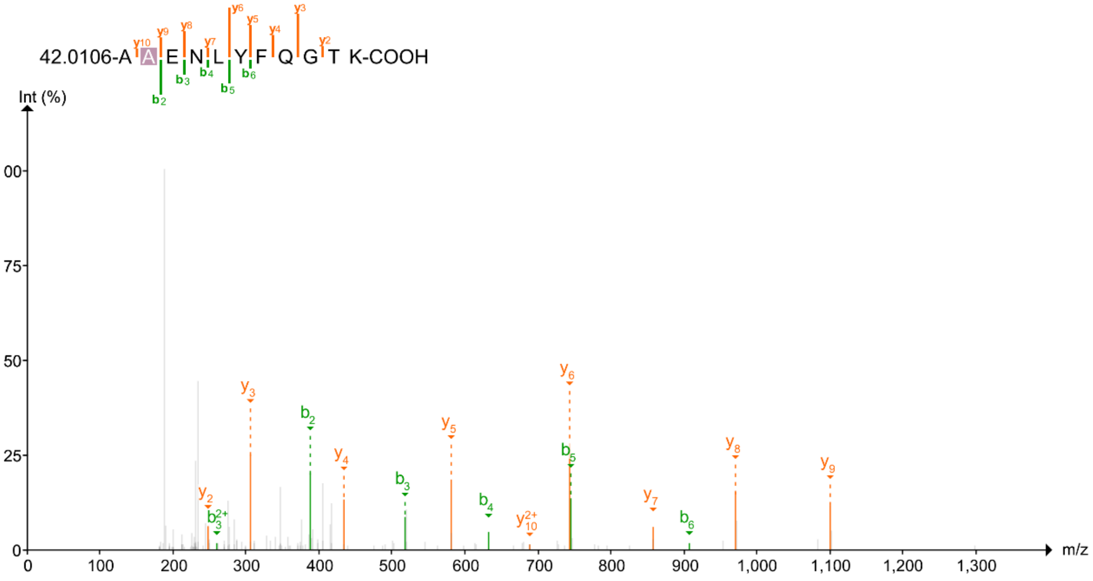

Figure S11. Fragmentation spectrum of [QIIANTVDFGASA(Hex)APLSDEK]$^{2+}$ originating from PstS-Man-Ala57.

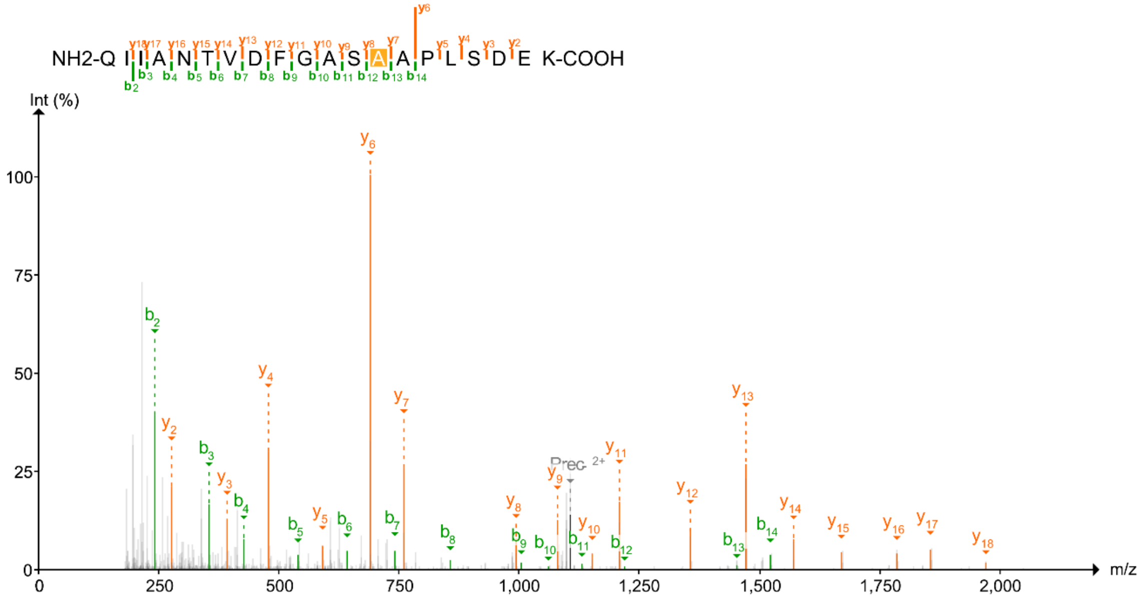

Figure S12. Fragmentation spectrum of [QIIANTVDFGASA(Hex)APLSDEK]$^{2+}$ originating from PstS-Gal-Ala57.

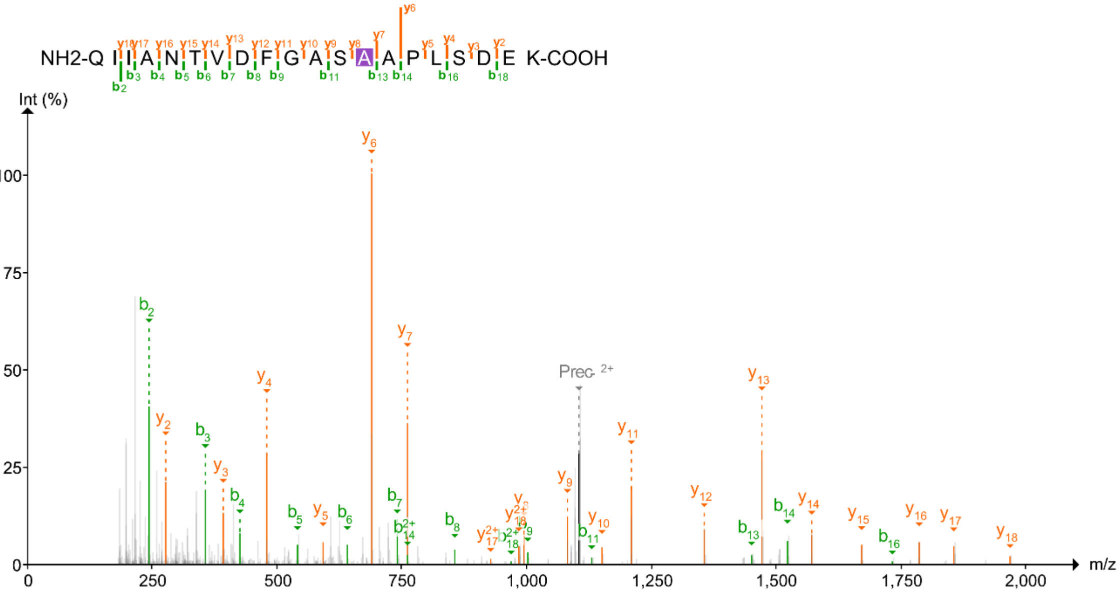

Figure S13. Fragmentation spectrum of [QIIANTVDFGASA(HexNAc)APLSDEK]$^{2+}$ originating from PstS-GlcNAc-Ala57.

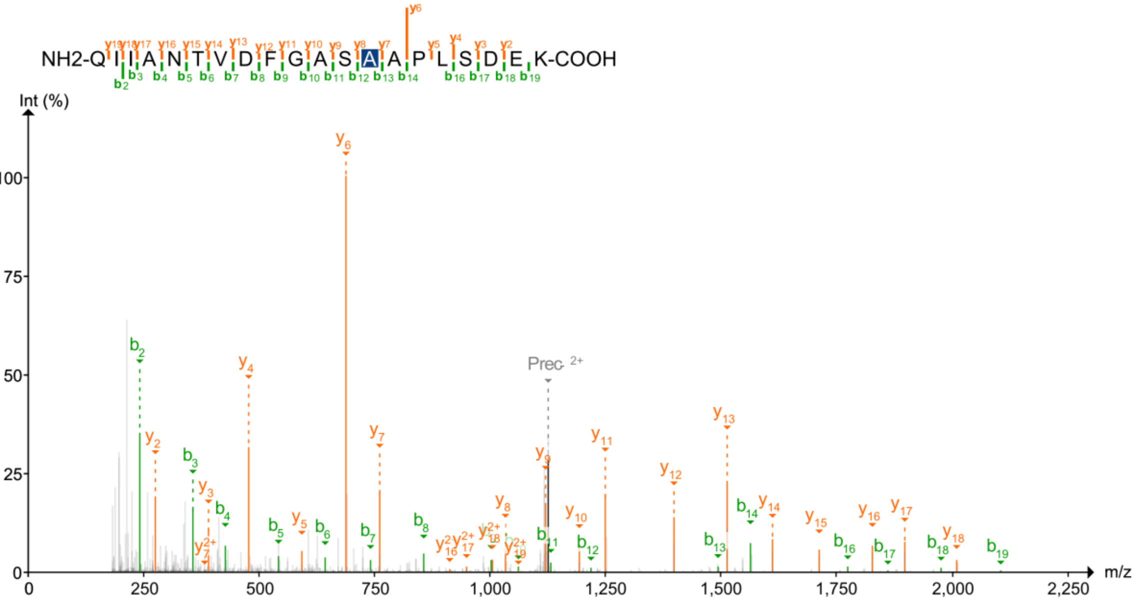

Figure S14. Fragmentation spectrum of [MYSFPNA(HexNAc)FR]$^{2+}$ originating from SsβG-GlcNAc-Ala7.

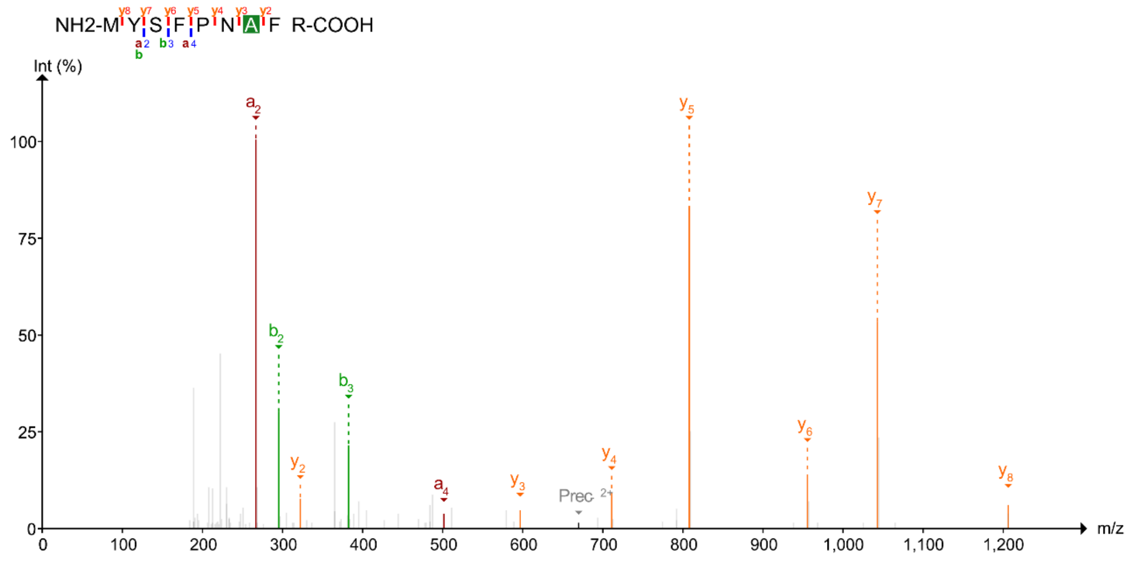

Table S9. A list of peptides with the mass shift corresponding (within the mass accuracy of 0.005 Da) to HexNAc identified in the tryptic digest of PstS-GlcNAc-Ala57. Possible additional non-specific modification sites are labelled with the red colour. The modification of the lysine residues in these peptides led to missed cleavages by trypsin at these sites.

DQKKPEQGTEVLK

DSSGKPLY

ETGNKVNYQGIGSSGGVK

FFDWAYKTGAK

GADWSKTFAQDLTNQK

KPEQGTEVLK

LISADGKPVSPTEENFANAAK

LISADGKPVSPTEENFANAAKGADWSK

LNPGLKLPSQNIAVVR

MEASLTGAGATFPAPVYAK – unable to localise

NNVGTGSTVKWPIGLGGK

QIIANTVDFGASAAPLSDEK

QNNLAYTKLISADGKPVSPTEENFANAAK

SGELVLDGKTLGDIYLGK

TLGDIYLGKIK

TNIKDSSGKPLY

Figure S15. Coverage of the amino-acid sequence of PstS-GlcNAc-Ala57 by peptides listed in Table S9. Modification sites are highlighted in red.

MEASLTGAGATFPAPVYAKWADTYQKETGNKVNYQGIGSSGGVKQIIAN
TVDFGASAAPLSDEKLAQEGLFQFPTVIGGVVLAVNIPGLKSGELVLDGK
TLGDIYLGKIKKWDDEAIAKLNPGLKLPSQNIAVVRRADGSGTSFVFTSYL
AKVNEEWKNNVGTGSTVKWPIGLGGKGNDGIAAFVQRLPGAIGYVEYA
YAKQNNLAYTKLISADGKPVSPTEENFANAAKGADWSKTFAQDLTNQKG
EDAWPITSTTFILIHKDQKKPEQGTEVLKFFDWAYKTGAKQANDLDYASL
PDSVVEQVRAAWKTNIKDSSGKPLY

9. Preliminary results for photoinduced *O*-glycosylation

9.1. Optimization of *O*-glycosylation reaction conditions

Table S10. Optimization for the synthesis of *O*-glycosides[a]

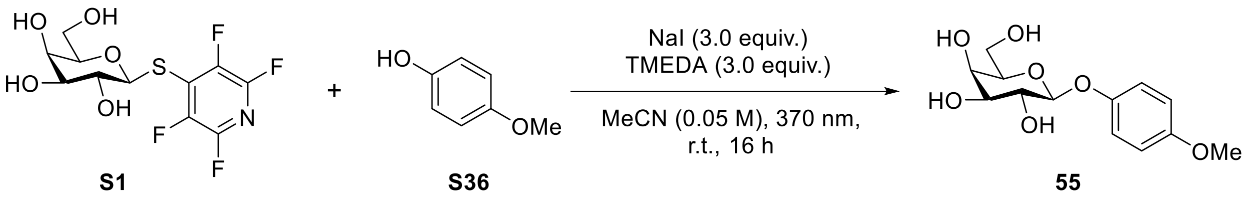

^{1}H NMR yield: 23%
>95:5 β:α

Entry	Variation from the standard conditions	^{1}H NMR yields[b]
1	LiI, Ki, ZnI_2 as iodide salt	13%, 9%, <2%
2	Et_3N, 2,4,6-collidine, DBU, K_2CO_3 as base	17%, <2%, 11%, <2%
3	w/o base	<2%
4	Blue LED or w/o *hv*	<2%, <2%
5	**S1** 3.0 equiv., **S36** 1.0 equiv.	47% (43%[c])

[a]Reaction conditions: **S1** (1.0 equiv., 0.05 mmol), **S36** (3.0 equiv, 0.15 mmol), NaI (3.0 equiv., 0.15 mmol), TMEDA (3.0 equiv, 0.15 mmol), MeCN (0.05 M, 1.0 mL), 370 nm Kessil lamp, r.t., 16h. [b]Yields were determined by ^{1}H NMR with tetrachloroethane as the internal standard. [c]Isolated yield.

General procedure G: synthesis of *O*-glycosides

Under air, a 4 mL vial equipped with a magnetic stir bar was added glycosyl donor (3.0 equiv., 0.15 mmol), phenol substrate (1.0 equiv., 0.05 mmol). The reaction vial was then transferred into a glovebox under nitrogen atmosphere, followed by the addition of NaI (3.0 equiv., 0.15 mmol), anhydrous MeCN (1.0 mL) and TMEDA (3.0 equiv., 0.15 mmol). The reaction vial was sealed and taken out of the glovebox. The reaction was allowed to vigorously stir at room temperature under 370 nm Kessil lamp illumination for 16 hours. After the reaction was complete, the volatiles were evaporated and the resulting residue was purified by flash silica gel column chromatography (eluent: CH_2Cl_2/MeOH = 30/1 ~ 5/1) to afford the target product.

9.2. Analytic data of *O*-glycosides

(2*R*,3*R*,4*S*,5*R*,6*S*)-2-(Hydroxymethyl)-6-(4-methoxyphenoxy)tetrahydro-2*H*-pyran-3,4,5-triol (55):

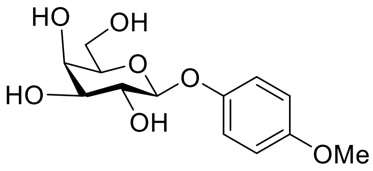

The title compound was prepared according to the **General procedure G** from **S1** (3.0 equiv., 0.15 mmol), Mequinol (1.0 equiv., 0.05 mmol), NaI (3.0 equiv., 0.15 mmol), anhydrous MeCN (1.0 mL) and TMEDA (3.0 equiv., 0.15 mmol). The residue was subjected to flash silica gel column chromatography (eluent: CH_2Cl_2/MeOH = 30/1 ~5/1) to give the pure product as white solid. (Isolated yield: 6.1 mg, 43%, > 95:5 β:α).

^{1}H NMR (500 MHz, Deuterium Oxide) δ 7.14 – 7.09 (m, 2H), 7.01 – 6.95 (m, 2H), **4.95 (d, *J* = 7.0 Hz, 1H, anomeric H**), 4.00 – 3.96 (m, 1H), 3.84 – 3.80 (m, 4H), 3.79 – 3.72 (m, 4H); ^{13}C NMR (126 MHz, D_2O) δ 154.61, 151.02, 118.08, 115.01, 101.70, 75.33, 72.54, 70.57, 68.46, 60.73, 55.78. HRMS (ESI) m/z calcd for $C_{13}H_{18}NaO_7$ [$(M+Na)^+$]: 309.0945, found: 309.0951.

(2*R*,3*S*,4*S*,5*R*,6*S*)-2-(Hydroxymethyl)-6-(4-methoxyphenoxy)tetrahydro-2*H*-pyran-3,4,5-triol (56):

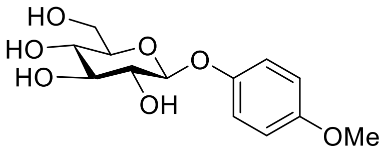

The title compound was prepared according to **the General procedure G** from **2** (3.0 equiv., 0.15 mmol), Mequinol (1.0 equiv., 0.05 mmol), NaI (3.0 equiv., 0.15 mmol), anhydrous MeCN (1.0 mL) and TMEDA (3.0 equiv., 0.15 mmol). The residue was subjected to flash silica gel column chromatography (eluent: CH_2Cl_2/MeOH = 30/1 ~5/1) to give the pure product as white solid. (Isolated yield: 5.8 mg, 41%, > 95:5 β:α).

^{1}H NMR (500 MHz, Deuterium Oxide) δ 7.11 – 7.06 (m, 2H), 6.97 – 6.93 (m, 2H), **4.98 (d, *J* = 7.7 Hz, 1H, anomeric H)**, 3.89 (dd, *J* = 12.5, 2.2 Hz, 1H), 3.78 (s, 3H), 3.72 (dd, *J* = 12.4, 5.7 Hz, 1H), 3.59 – 3.53 (m, 2H), 3.51 (dd, *J* = 9.4, 7.7 Hz, 1H), 3.48 – 3.43 (m, 1H); ^{13}C NMR (126 MHz, Deuterium Oxide) δ 154.67, 150.83, 118.12, 114.97, 101.12, 76.02, 75.50, 72.91, 69.38,

60.48, 55.72; HRMS (ESI) m/z calcd for $C_{13}H_{18}NaO_7$ [(M+Na)$^+$]: 309.0945, found: 309.0952.

(2*R*,3*S*,4*S*,5*R*,6*S*)-2-(Hydroxymethyl)-6-(2-methoxyphenoxy)tetrahydro-2*H*-pyran-3,4,5-triol (57):

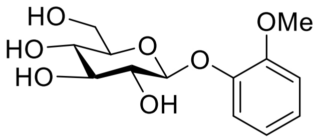

The title compound was prepared according to the **General procedure G** from **2** (3.0 equiv., 0.15 mmol), Guaiacol (1.0 equiv., 0.05 mmol), NaI (3.0 equiv., 0.15 mmol), anhydrous MeCN (1.0 mL) and TMEDA (3.0 equiv., 0.15 mmol). The residue was subjected to flash silica gel column chromatography (eluent: CH_2Cl_2/MeOH = 30/1 ~5/1) to give the pure product as white solid. (Isolated yield: 5.8 mg, 41%, > 95:5 β:α).

^{1}H NMR (500 MHz, Methanol-*d₄*) δ 7.19 – 7.15 (m, 1H), 7.02 – 6.97 (m, 2H), 6.90 (ddd, *J* = 8.0, 5.5, 3.6 Hz, 1H), **4.89 (d, *J* = 7.4 Hz, 1H)**, 3.90 – 3.84 (m, 4H), 3.72 – 3.67 (m, 1H), 3.52 – 3.44 (m, 2H), 3.43 – 3.38 (m, 2H); ^{13}C NMR (126 MHz, Deuterium Oxide) δ 148.67, 145.31, 123.83, 121.50, 116.17, 112.94, 100.41, 76.05, 75.48, 72.82, 69.25, 60.39, 55.76; HRMS (ESI) m/z calcd for $C_{13}H_{18}NaO_7$ [(M+Na)$^+$]: 309.0945, found: 309.0952.

(2*S*,3*R*,4*S*,5*S*,6*R*)-2-(2,4-Dimethylphenoxy)-6-(hydroxymethyl)tetrahydro-2*H*-pyran-3,4,5-triol (58):

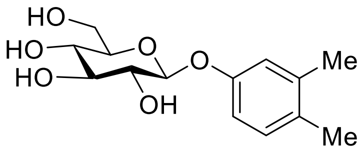

The title compound was prepared according to the **General procedure G** from **2** (3.0 equiv., 0.15 mmol), 3,4-xylenol (1.0 equiv., 0.05 mmol), NaI (3.0 equiv., 0.15 mmol), anhydrous MeCN (1.0 mL) and TMEDA (3.0 equiv., 0.15 mmol). The residue was subjected to flash silica gel column chromatography (eluent: CH_2Cl_2/MeOH = 30/1 ~5/1) to give the pure product as white solid. (Isolated yield: 4.8 mg, 34%, > 95:5 β:α).

^{1}H NMR (500 MHz, Deuterium Oxide) δ 7.04 (d, *J* = 8.3 Hz, 1H), 6.84 (d, *J* = 2.7 Hz, 1H), 6.76 (dd, *J* = 8.3, 2.7 Hz, 1H), **4.92 (d, *J* = 7.7 Hz, 1H, anomeric H)**, 3.80 (dd, *J* = 12.5, 2.3 Hz, 1H),

3.62 (dd, J = 12.4, 5.8 Hz, 1H), 3.50 – 3.44 (m, 2H), 3.42 (dd, J = 9.4, 7.6 Hz, 1H), 3.39 – 3.33 (m, 1H), 2.12 (s, 3H), 2.08 (s, 3H); ^{13}C NMR (126 MHz, Deuterium Oxide) δ 154.58, 138.73, 131.86, 130.48, 117.73, 113.71, 100.39, 76.01, 75.53, 72.91, 69.41, 60.51, 18.97, 17.89; HRMS (ESI) m/z calcd for $C_{14}H_{20}NaO_6$ [(M+Na)$^+$]: 307.1152, found: 307.1160.

(2*R*,3*S*,4*S*,5*R*,6*S*)-2-(Hydroxymethyl)-6-(4-hydroxyphenoxy)tetrahydro-2*H*-pyran-3,4,5-triol (59):

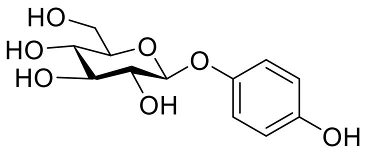

The title compound was prepared according to the **General procedure G** from **2** (3.0 equiv., 0.15 mmol), hydroquinone (1.0 equiv., 0.05 mmol), NaI (3.0 equiv., 0.15 mmol), anhydrous MeCN (1.0 mL) and TMEDA (3.0 equiv., 0.15 mmol). The residue was subjected to flash silica gel column chromatography (eluent: CH_2Cl_2/MeOH = 30/1 ~5/1) to give the pure product as white solid. (Isolated yield: 2.3 mg, 17%, > 95:5 β:α).

^{1}H NMR (500 MHz, Deuterium Oxide) δ 7.09 – 7.03 (m, 2H), 6.90 – 6.85 (m, 2H), **4.99 (d, *J* = 7.7 Hz, 1H, anomeric H)**, 3.93 (dd, J = 12.4, 2.3 Hz, 1H), 3.76 (dd, J = 12.5, 5.7 Hz, 1H), 3.62 – 3.56 (m, 2H), 3.53 (dd, J = 9.4, 7.7 Hz, 1H), 3.49 (dd, J = 9.8, 8.9 Hz, 1H); ^{13}C NMR (126 MHz, Deuterium Oxide) δ 151.17, 150.35, 118.36, 116.15, 101.26, 75.99, 75.51, 72.91, 69.37, 60.47; HRMS (ESI) m/z calcd for $C_{12}H_{16}NaO_7$ [(M+Na)$^+$]: 295.0788, found: 295.0793.

10. References

1. Zubkov, M. O., Kosobokov, M. D., Levin, V. V. & Dilman A. D. Photocatalyzed decarboxylative thiolation of carboxylic acids enabled by fluorinated disulfide. *Org. Lett.* **24**, 2354–2358 (2022).
2. Kremzow, D., Seidel, G., Lehmann, C. W. & Furstner, A. Diaminocarbene- and Fischer-carbene complexes of palladium and nickel by oxidative insertion: preparation, structure, and catalytic activity. *Chem. Eur. J.* **11**, 1833–1853 (2005).
3. Jiang, Y., Wang, Q., Zhang, X. & Koh, M. J. Synthesis of *C*-glycosides by Ti-catalyzed stereoselective glycosyl radical functionalization. *Chem* **7**, 3377–3392 (2021).
4. Zhang, C., Hu, W., Lovinger, G. J., Jin, J., Chen, J. & Morken, J. P. Enantiomerically enriched α-borylzinc reagents by nickel-catalyzed carbozincation of vinylboronic esters. *J. Am. Chem. Soc.* **143**, 14189–14195 (2021).
5. Kapat, A., Nyfeler, E., Giuffredi, G. T. & Renaud, P. Intramolecular Schmidt reaction involving primary azidoalcohols under nonacidic conditions: synthesis of indolizidine (-)-167B. *J. Am. Chem. Soc.* **131**, 17746–17747 (2009).
6. Tardieu, D., Desnoyers, M., Laye, C., Hazelard, D., Kern, N. & Compain, P. Stereoselective synthesis of *C*,*C*-glycosides from exo-glycals enabled by iron-mediated hydrogen atom transfer. *Org. Lett.* **21**, 7262–7267 (2019).
7. Wang, Q. *et al.* Iron-catalysed reductive cross-coupling of glycosyl radicals for the stereoselective synthesis of *C*-glycosides. *Nat. Synth.* **1**, 235–244 (2022).
8. Biscans, A., Rouanet, S., Vasseur, J. J., Dupouy, C. & Debart, F. A versatile post-synthetic method on a solid support for the synthesis of RNA containing reduction-responsive modifications. *Org. Biomol. Chem.* **14**, 7010–7017 (2016).
9. Mathuri, A., Pramanik, M., Parida, A. & Mal, P. Disulfide metathesis via sulfurcdots, three dots, centerediodine interaction and photoswitchability. *Org. Biomol. Chem.* **19**, 8539–8543 (2021).
10. Venkateswarlu, C., Gautam, V. & Chandrasekaran, S. Synthesis of mixed glycosyl disulfides/selenenylsulfides using benzyltriethylammonium tetrathiomolybdate as a sulfur transfer reagent. *Carbohydr. Res.* **402**, 200–207 (2015).
11. Floyd, N., Vijayakrishnan, B., Koeppe, J. R. & Davis, B. G. Thiyl glycosylation of olefinic

proteins: *S*-linked glycoconjugate synthesis. *Angew. Chem. Int. Ed.* **48**, 7798–7802 (2009).

12. Mollner, T. A. *et al*. Post-translational insertion of boron in proteins to probe and modulate function. *Nat. Chem. Biol.* **17**, 1245–1261 (2021).
13. Kong, A. T., Leprevost, F.V., Avtonomov, D. M., Mellacheruvu, D. & Nesvizhskii, A. I. MSFragger: ultrafast and comprehensive peptide identification in mass spectrometry-based proteomics. *Nat. Methods* **14**, 513–520 (2017).

11. NMR spectra

^{1}H NMR spectrum of 2,3,5,6-Tetraflupropyridine-4-thiol (PyFSH)

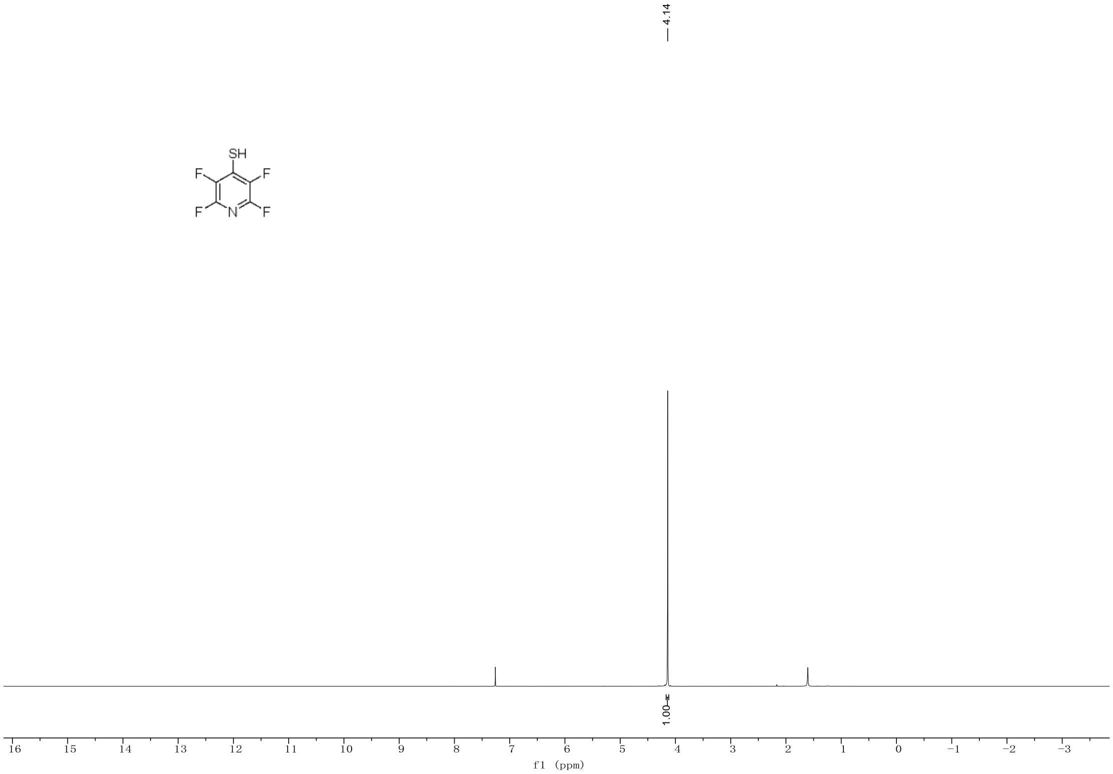

^{13}C NMR spectrum of 2,3,5,6-Tetraflupropyridine-4-thiol (PyFSH)

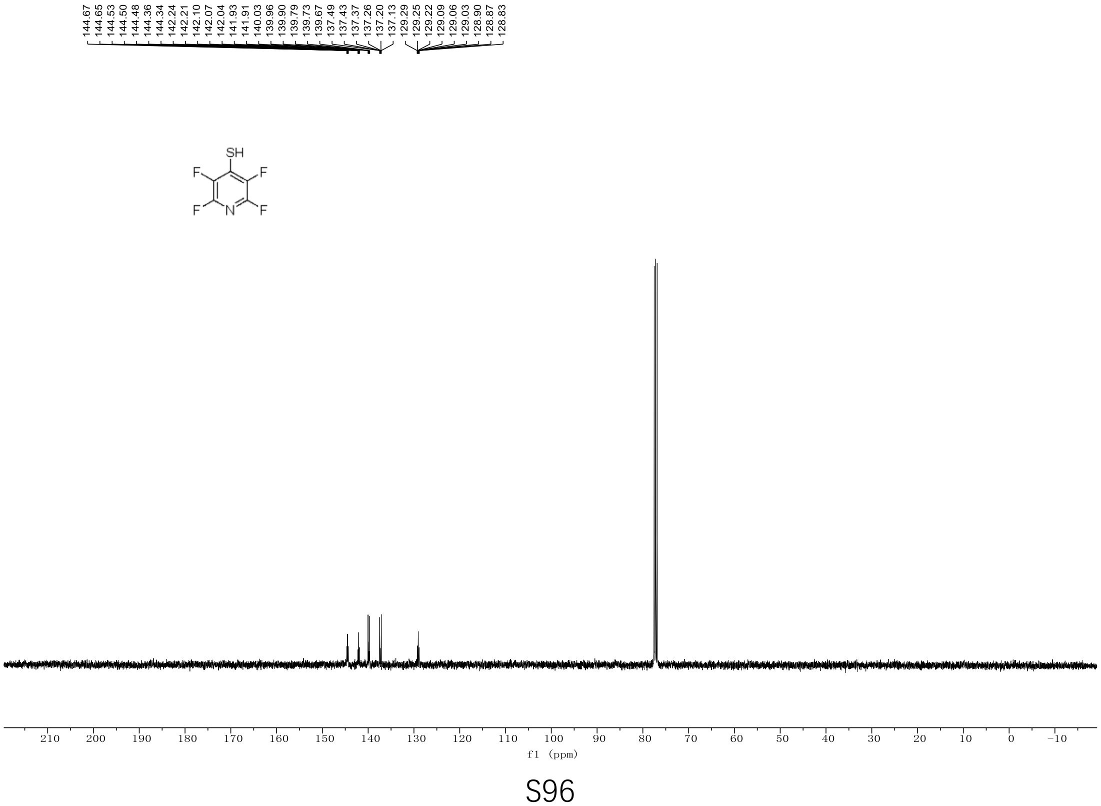

^{19}F NMR spectrum of 2,3,5,6-Tetraflupropyridine-4-thiol (PyFSH)

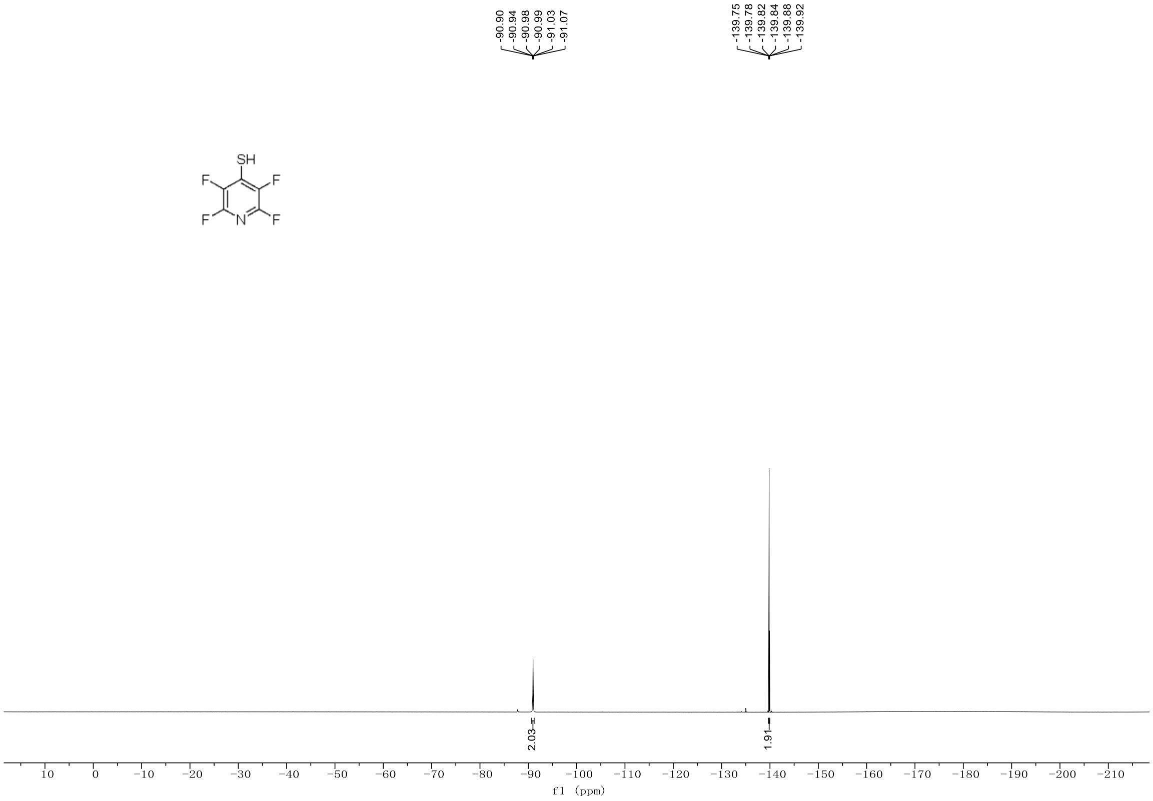

^{1}H NMR spectrum of 2-Chloro-1,3-dimethylimidazolinium chloride (DMC)

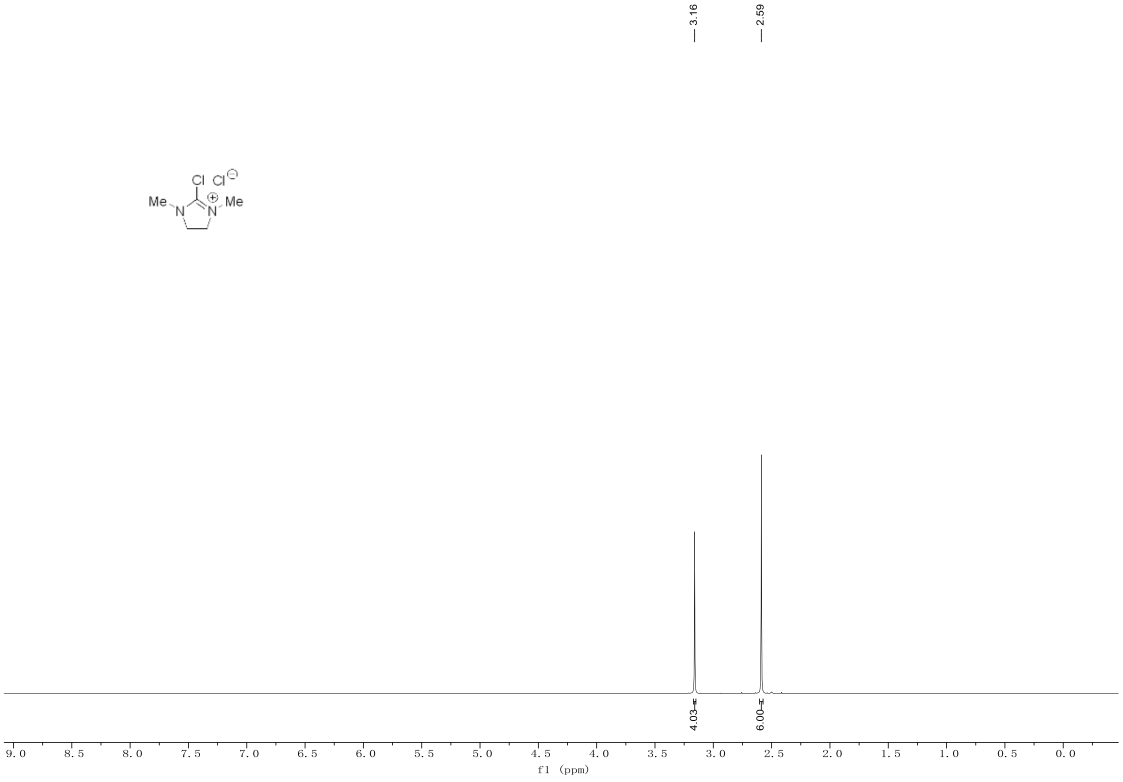

^{13}C NMR spectrum of 2-Chloro-1,3-dimethylimidazolinium chloride (DMC)

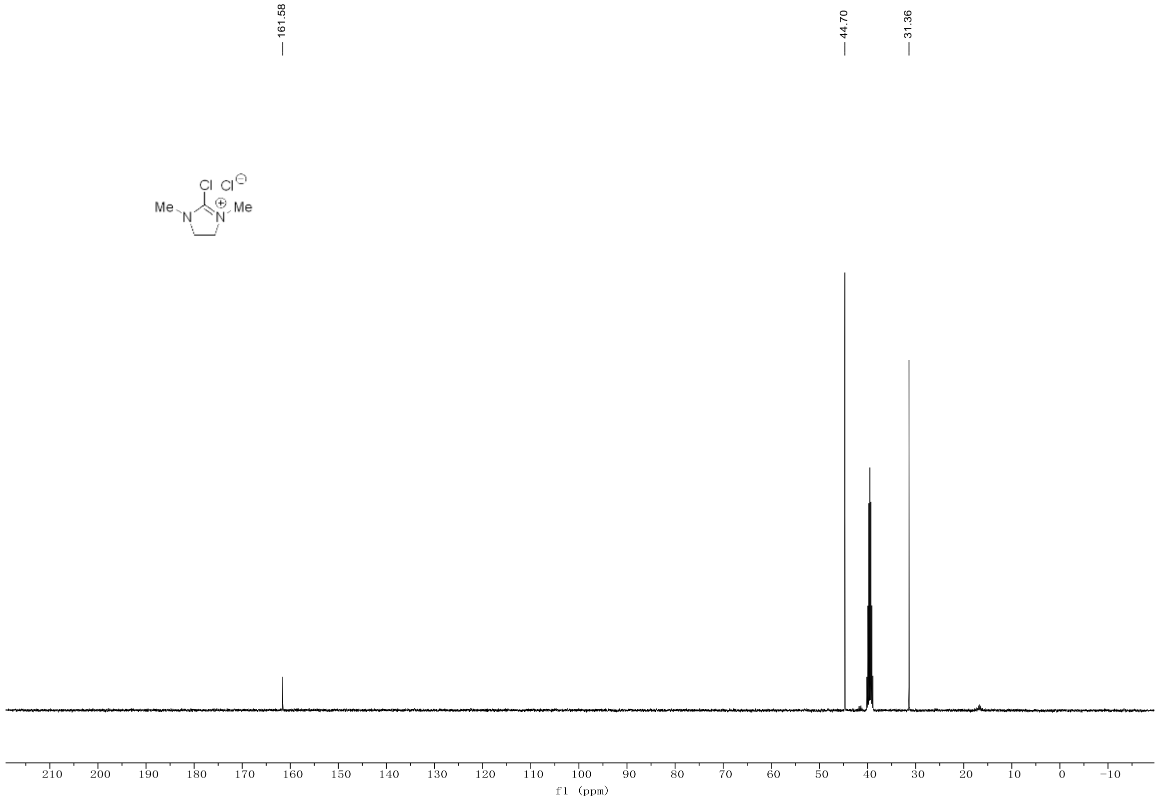

^{1}H NMR spectrum of compound 3

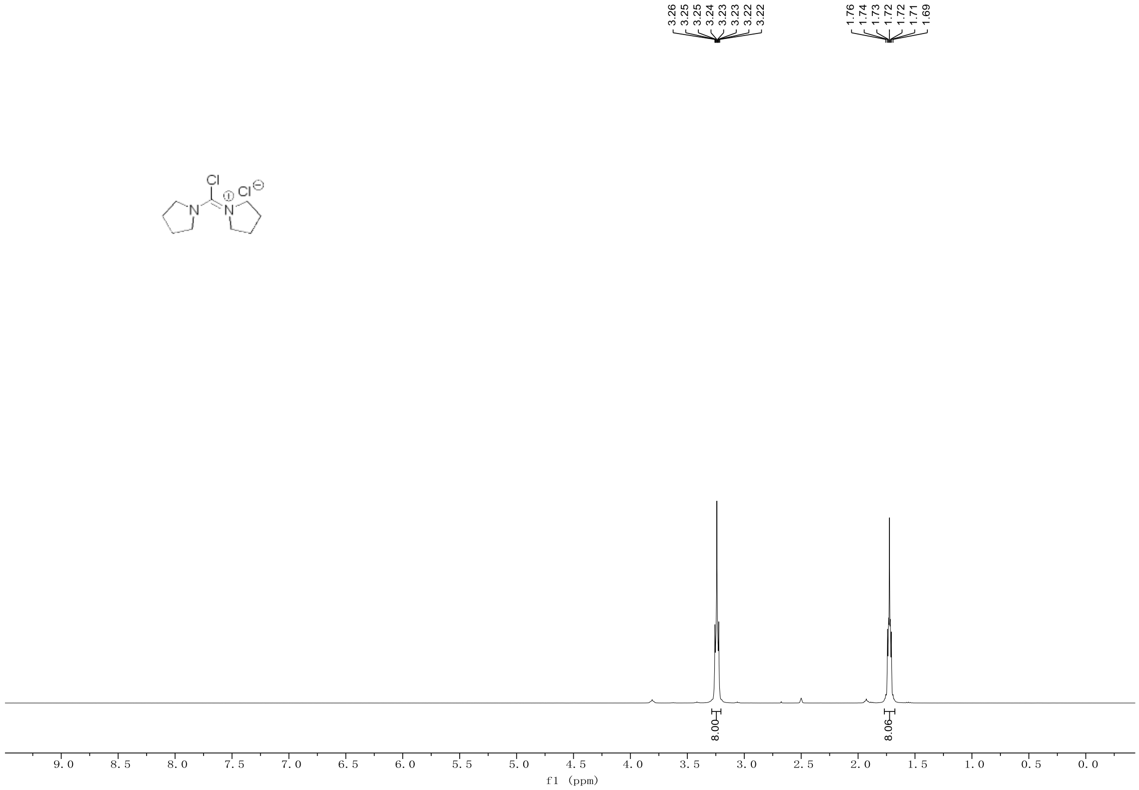

^{13}C NMR spectrum of compound 3

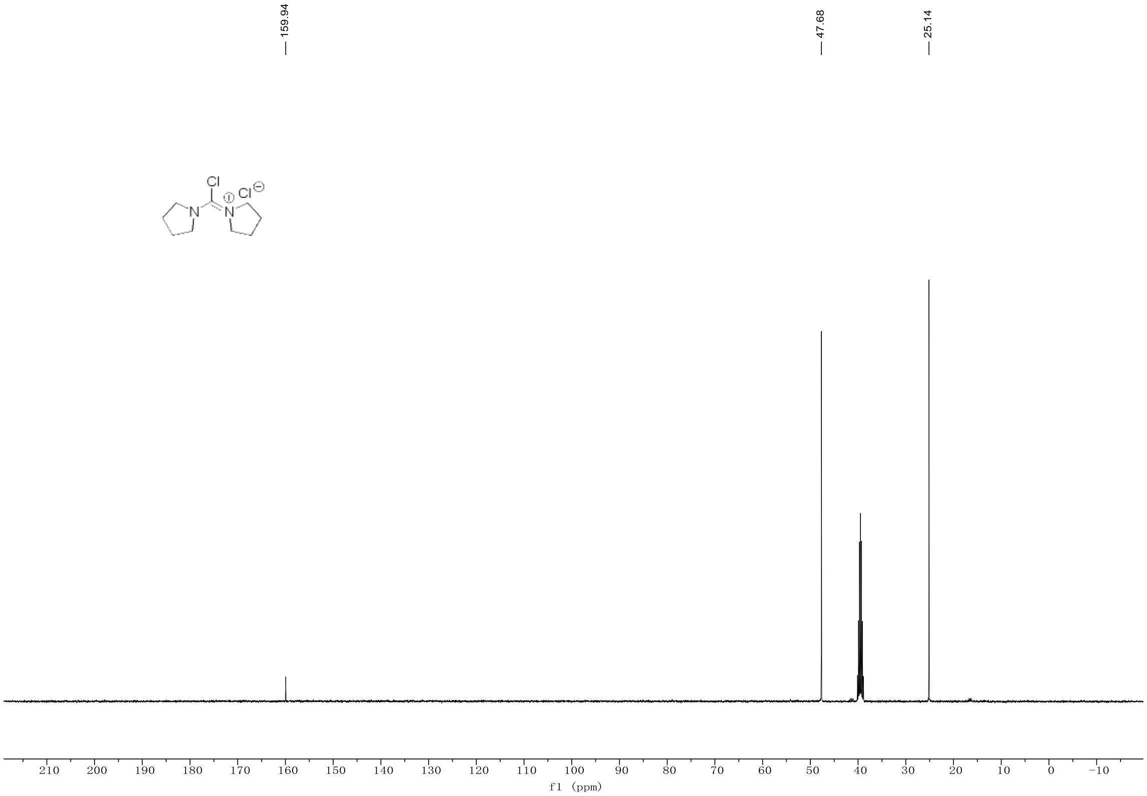

^{1}H NMR spectrum of compound 4

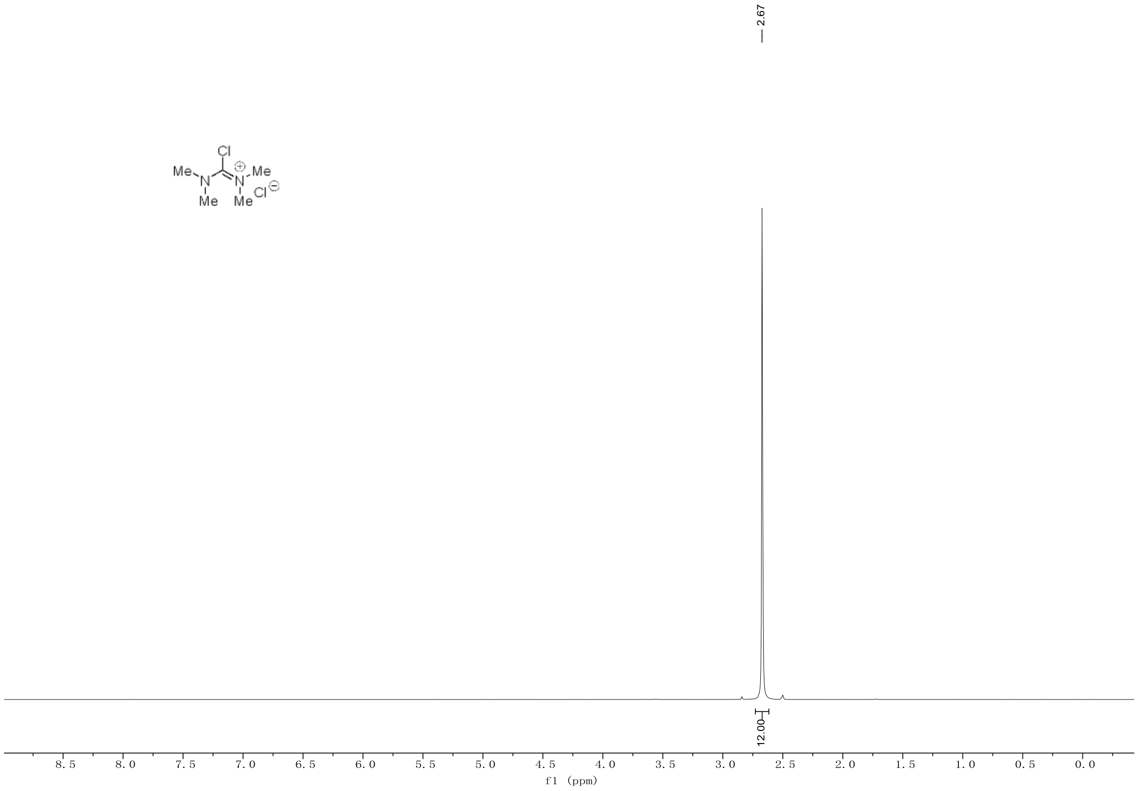

^{13}C NMR spectrum of compound 4

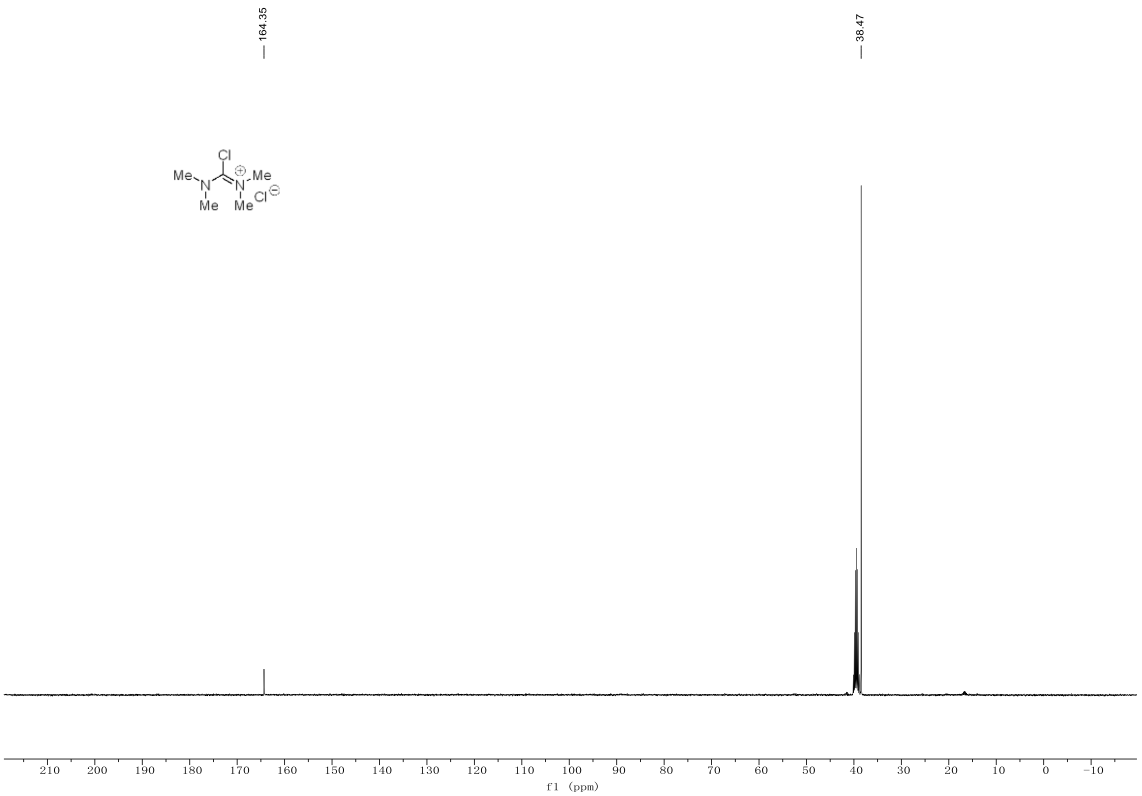

^{1}H NMR spectrum of compound 6

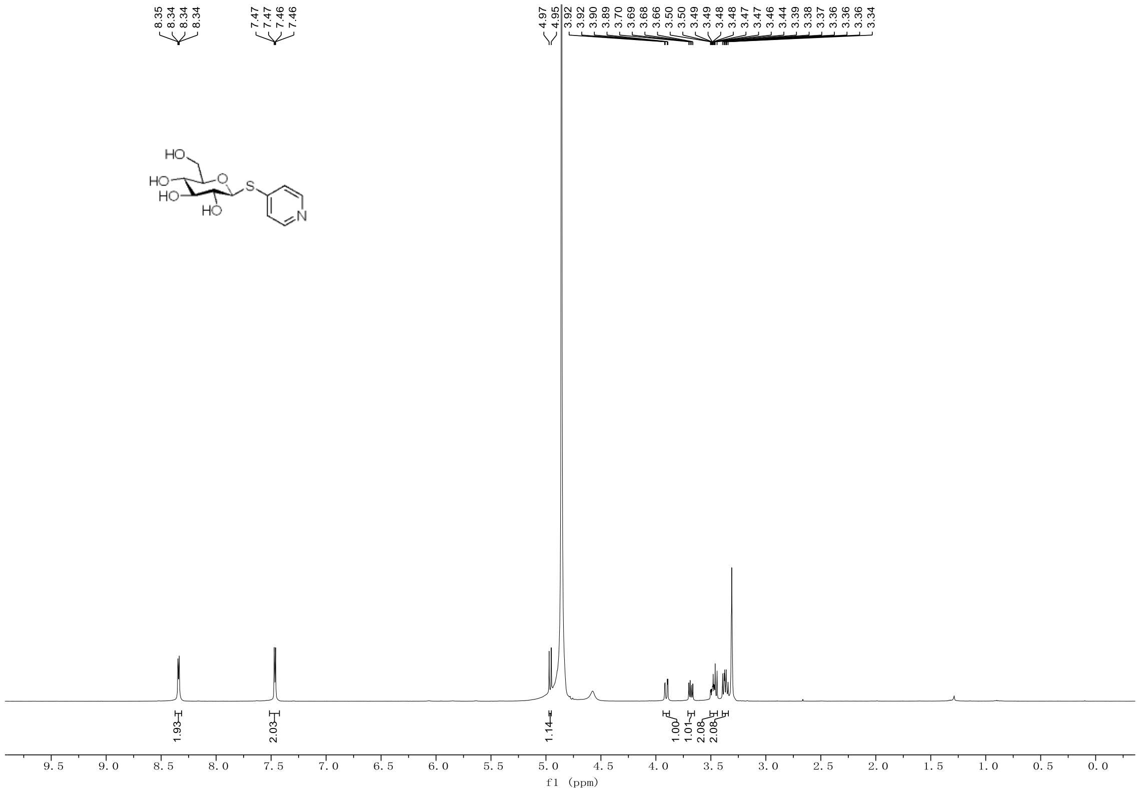

^{13}C NMR spectrum of compound 6

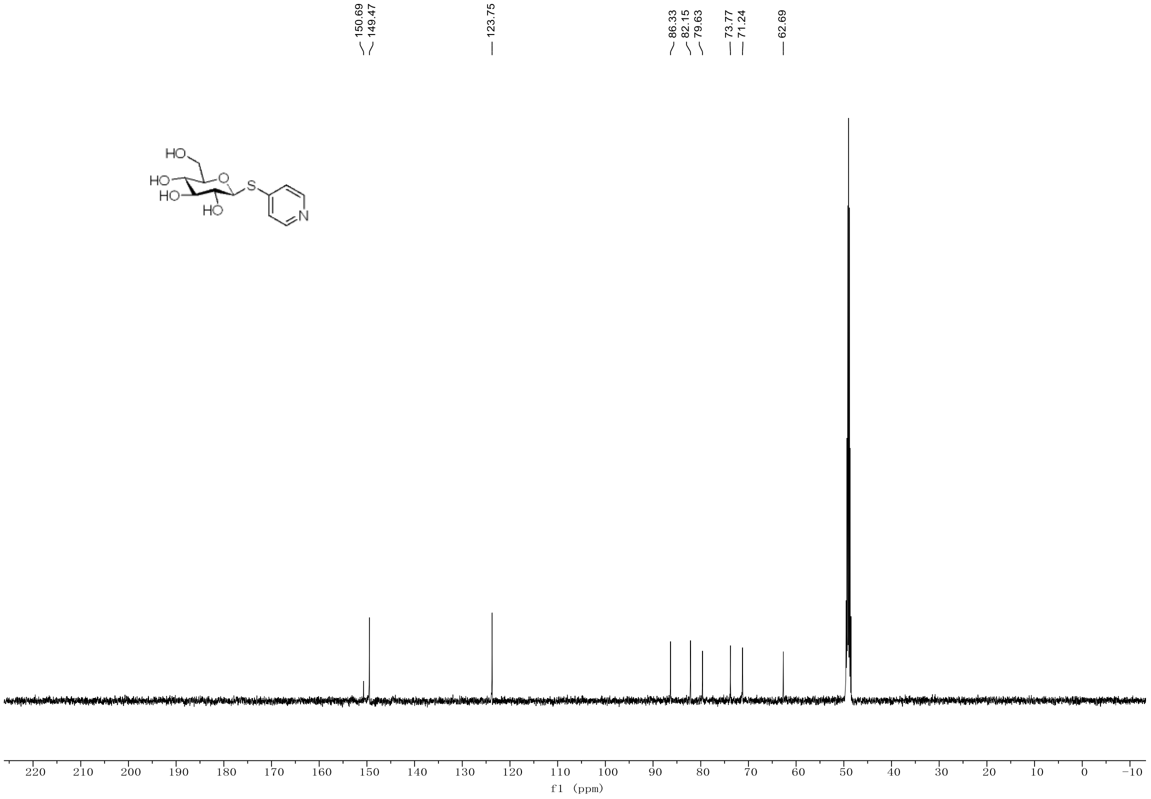

^{1}H NMR spectrum of compound 7

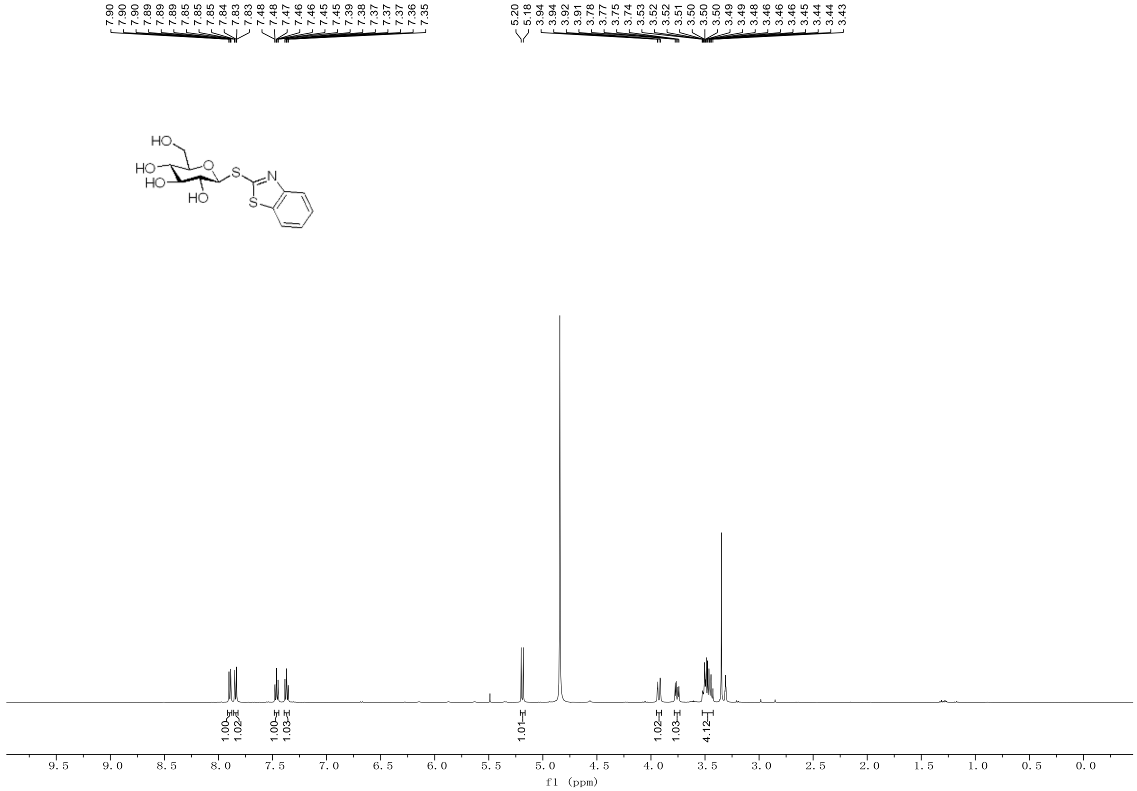

^{13}C NMR spectrum of compound 7

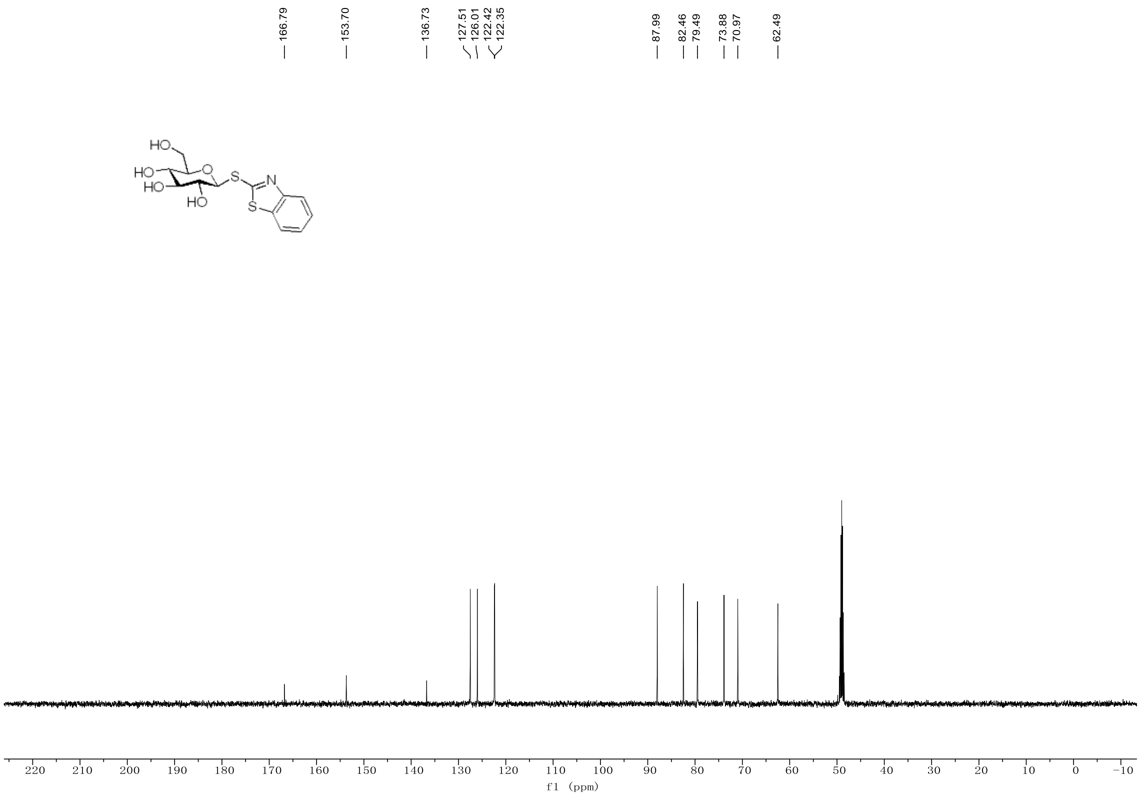

^{1}H NMR spectrum of compound 8

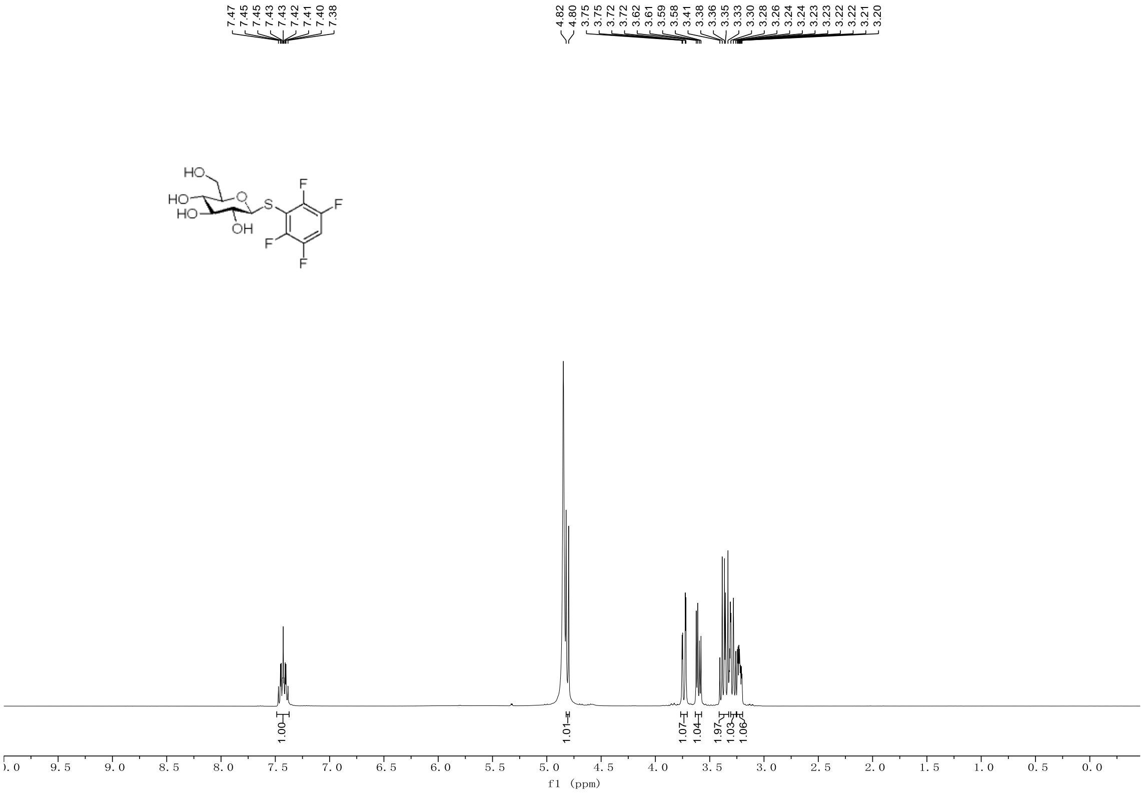

^{13}C NMR spectrum of compound 8

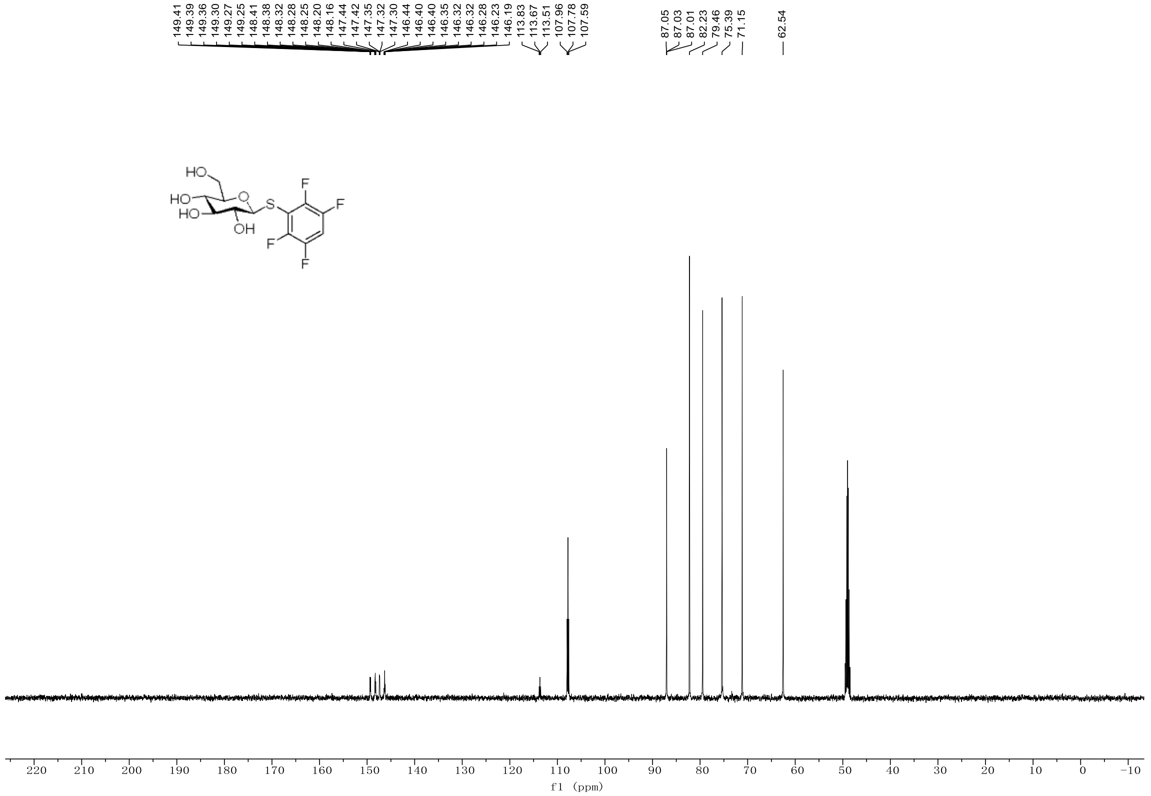

^{19}F NMR spectrum of compound 8

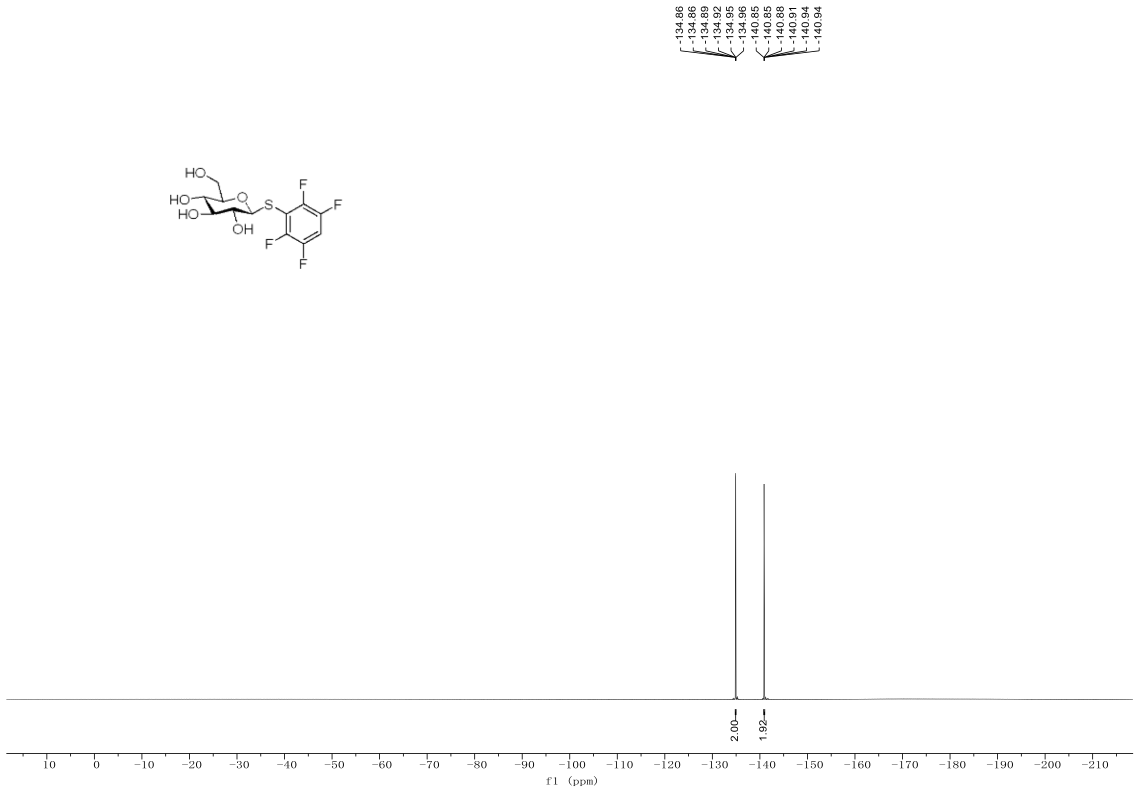

^{1}H NMR spectrum of compound 9

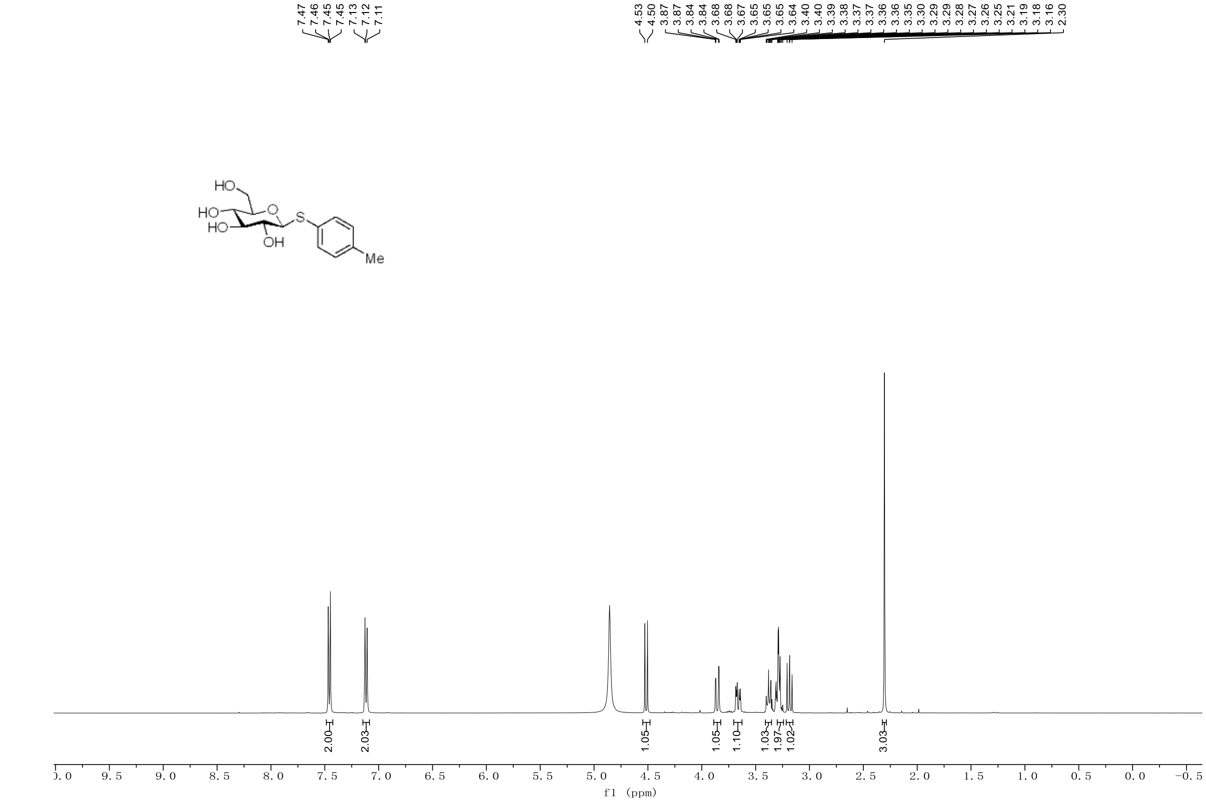

^{13}C NMR spectrum of compound 9

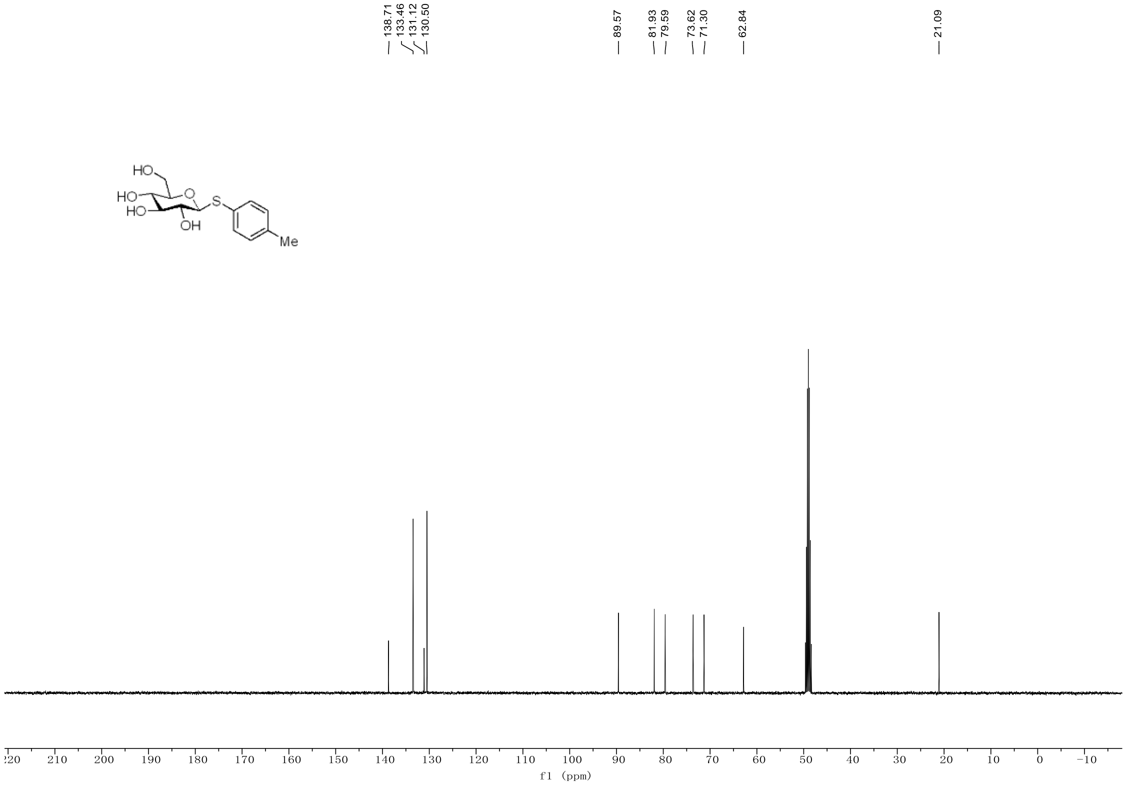

^{1}H NMR spectrum of compound 2

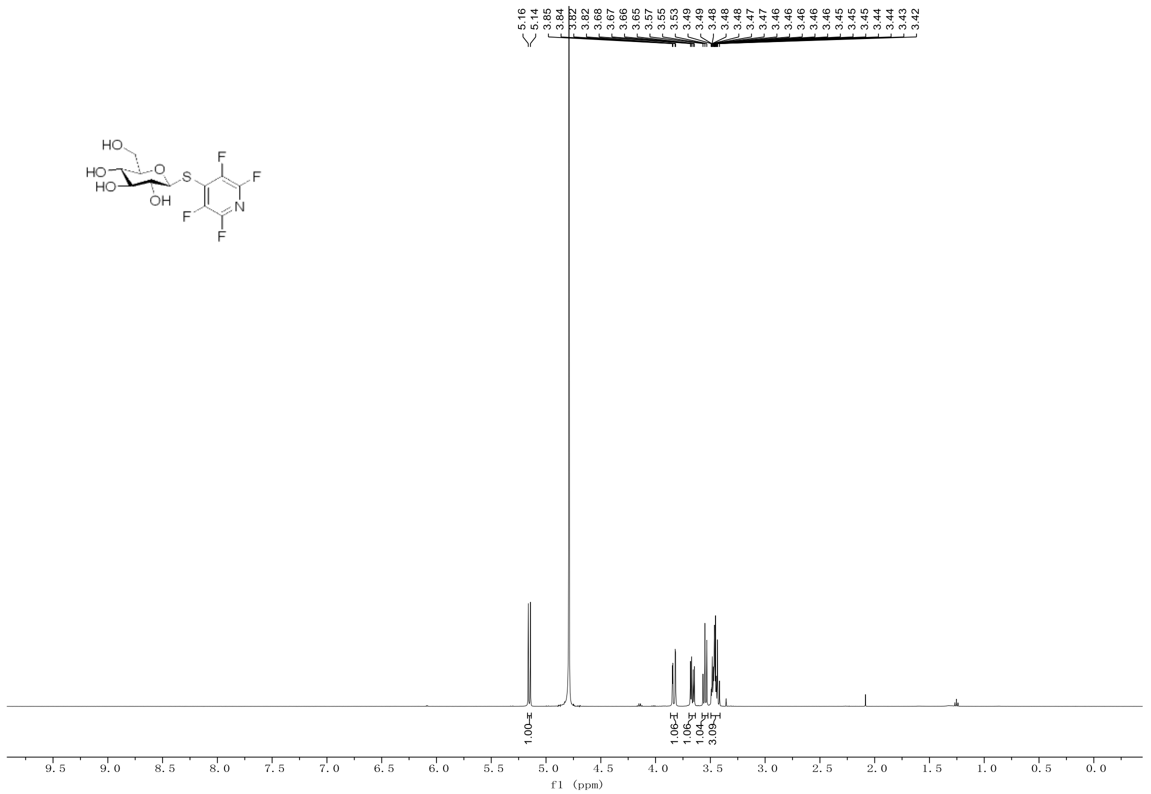

^{13}C NMR spectrum of compound 2

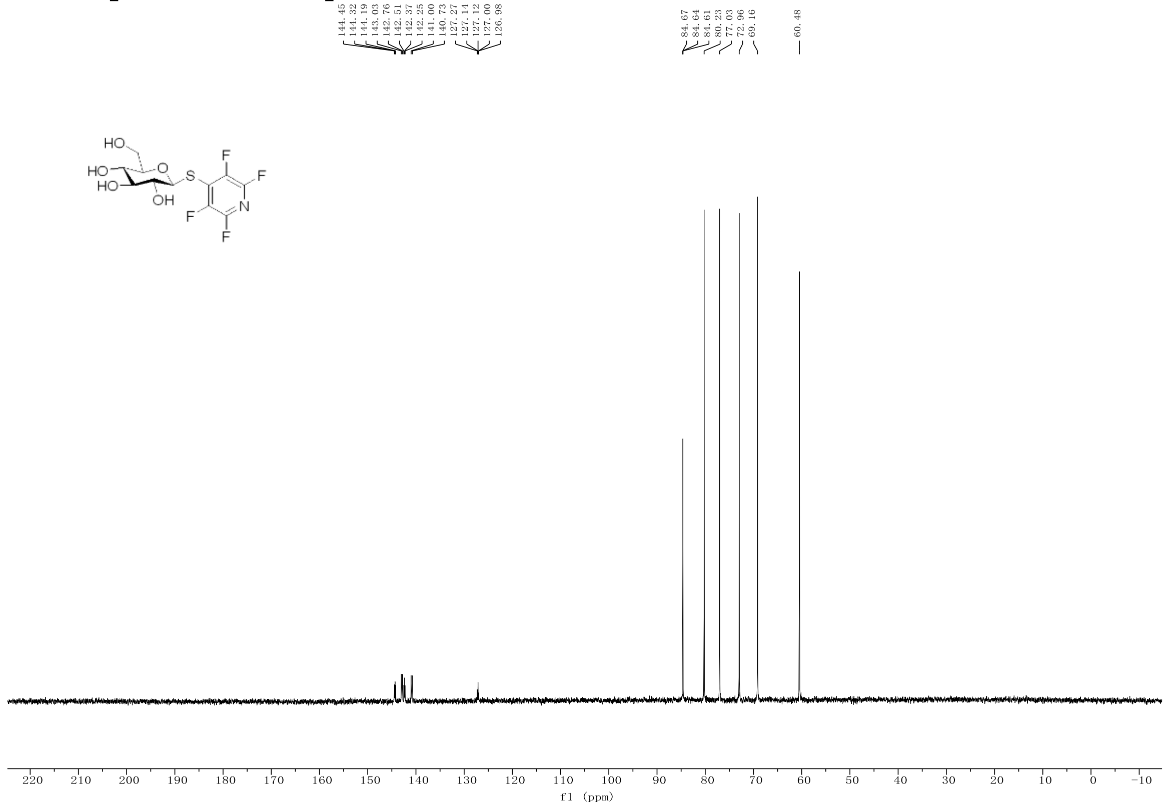

^{19}F NMR spectrum of compound 2

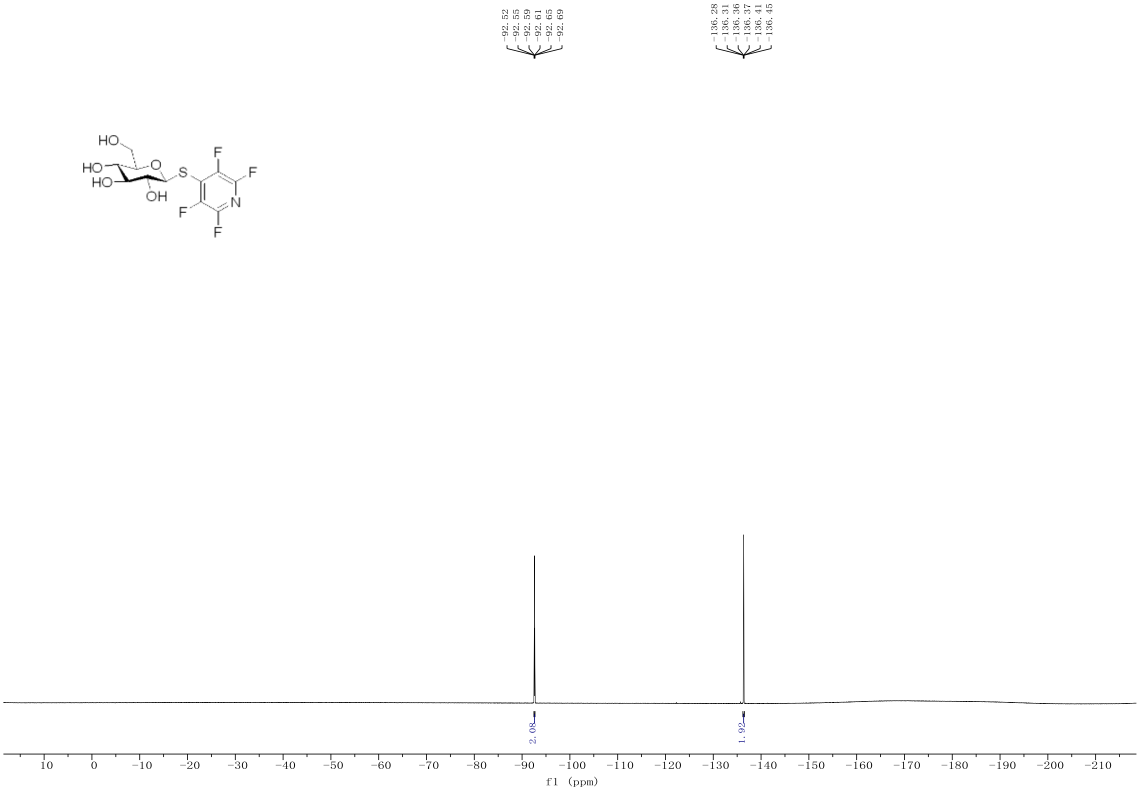

^{1}H NMR spectrum of compound S1

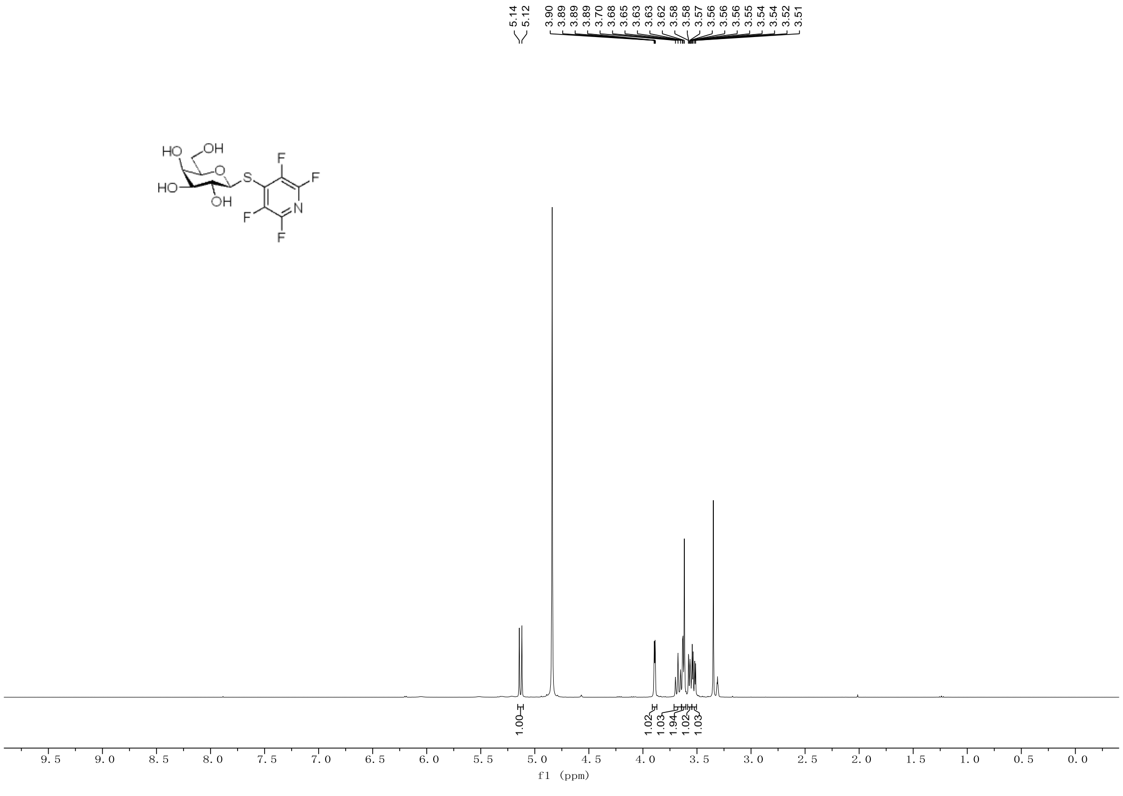

^{13}C NMR spectrum of compound S1

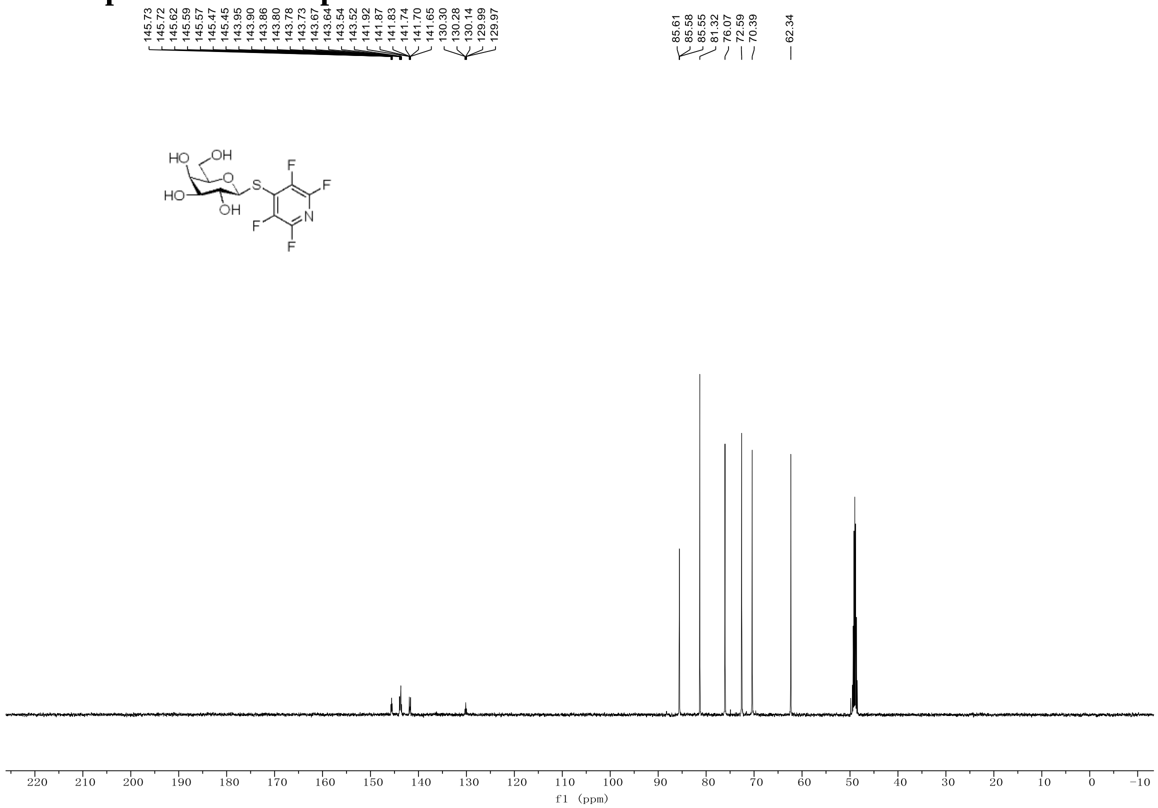

^{19}F NMR spectrum of compound S1

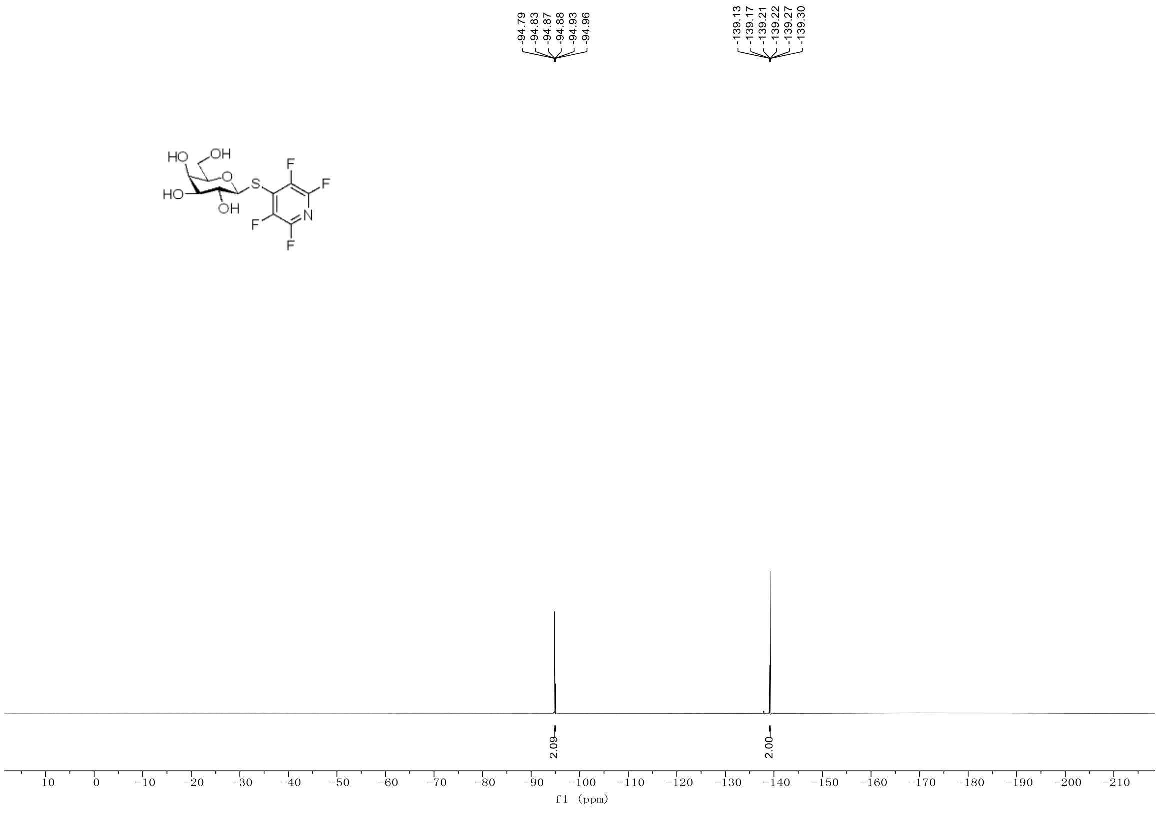

^{1}H NMR spectrum of compound S2

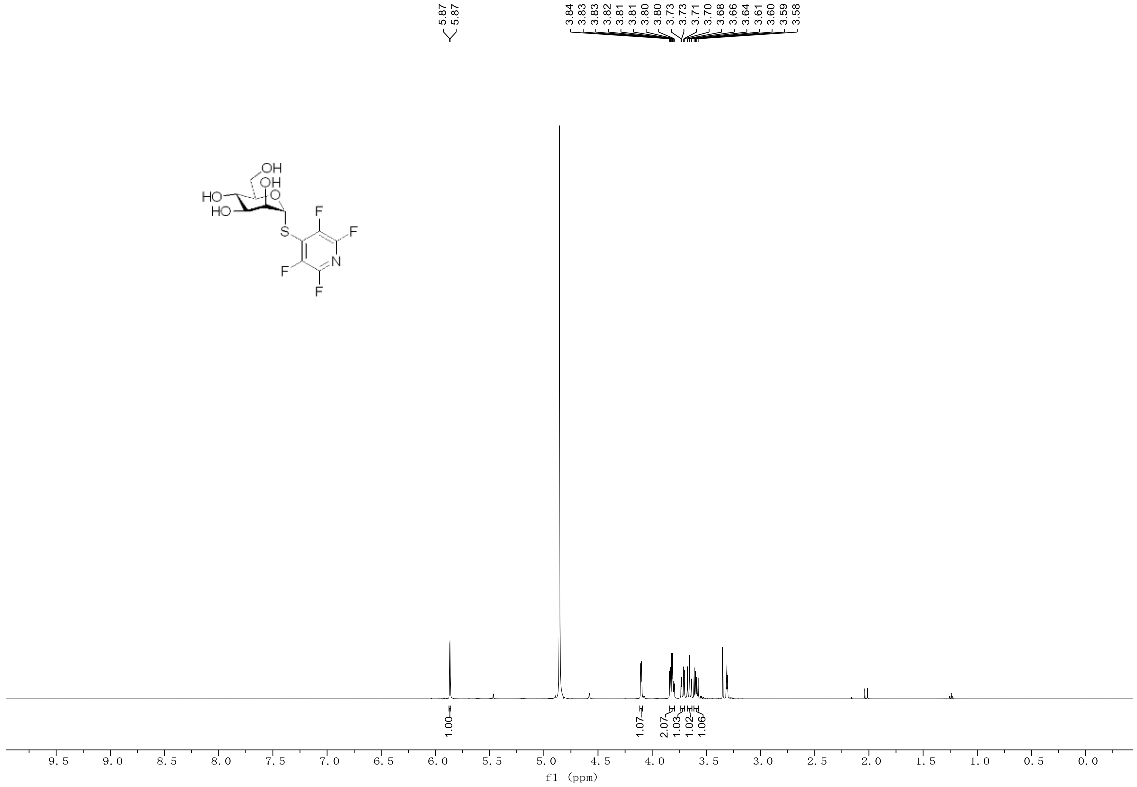

^{13}C NMR spectrum of compound S2

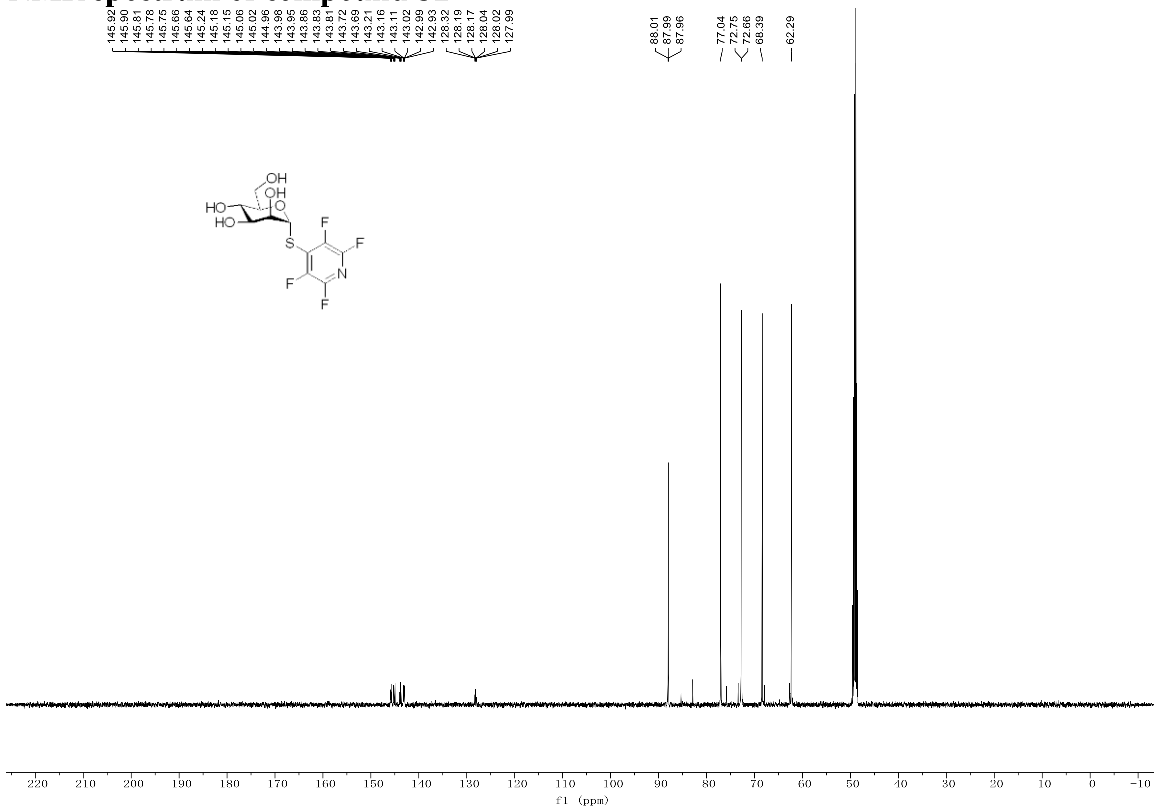

^{19}F NMR spectrum of compound S2

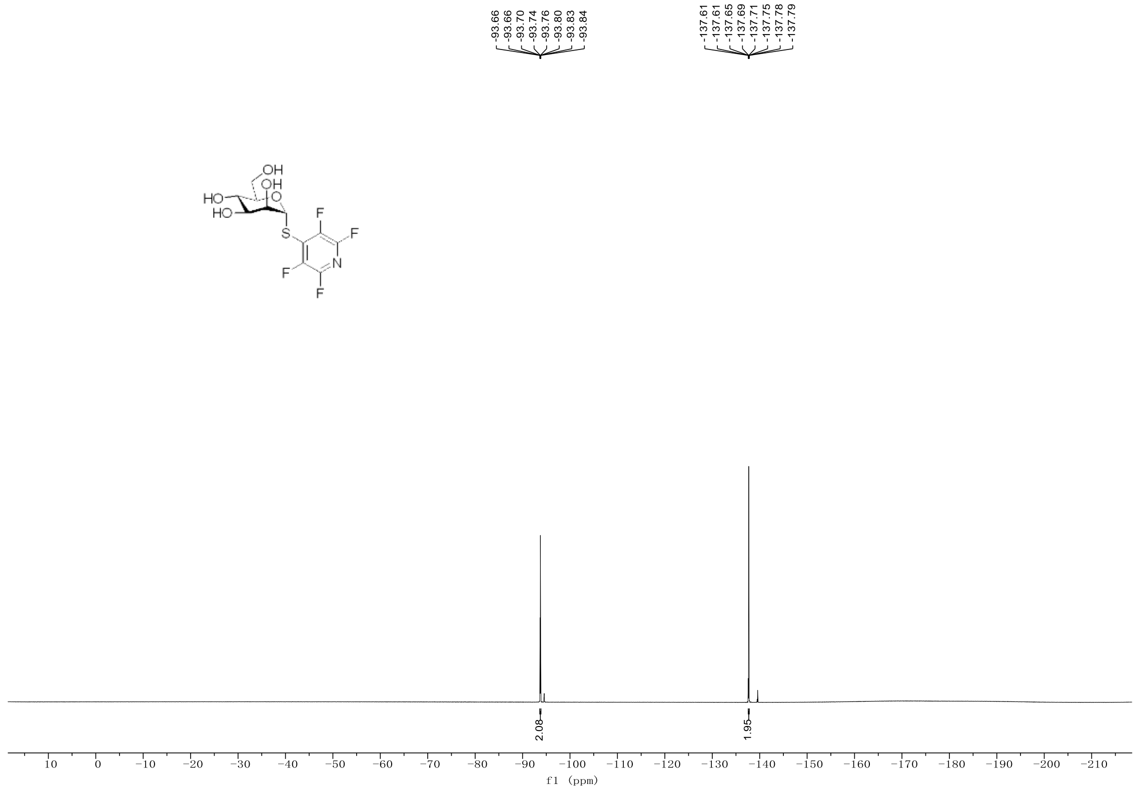

^{1}H NMR spectrum of compound S3

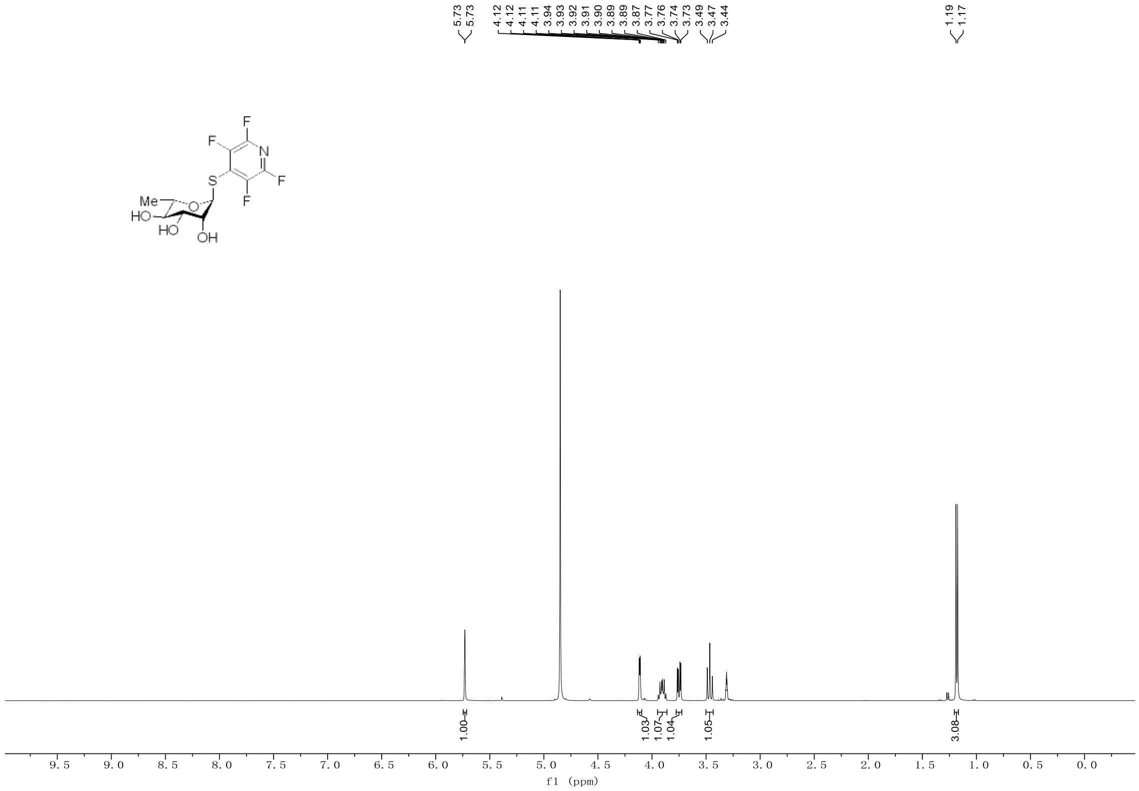

^{13}C NMR spectrum of compound S3

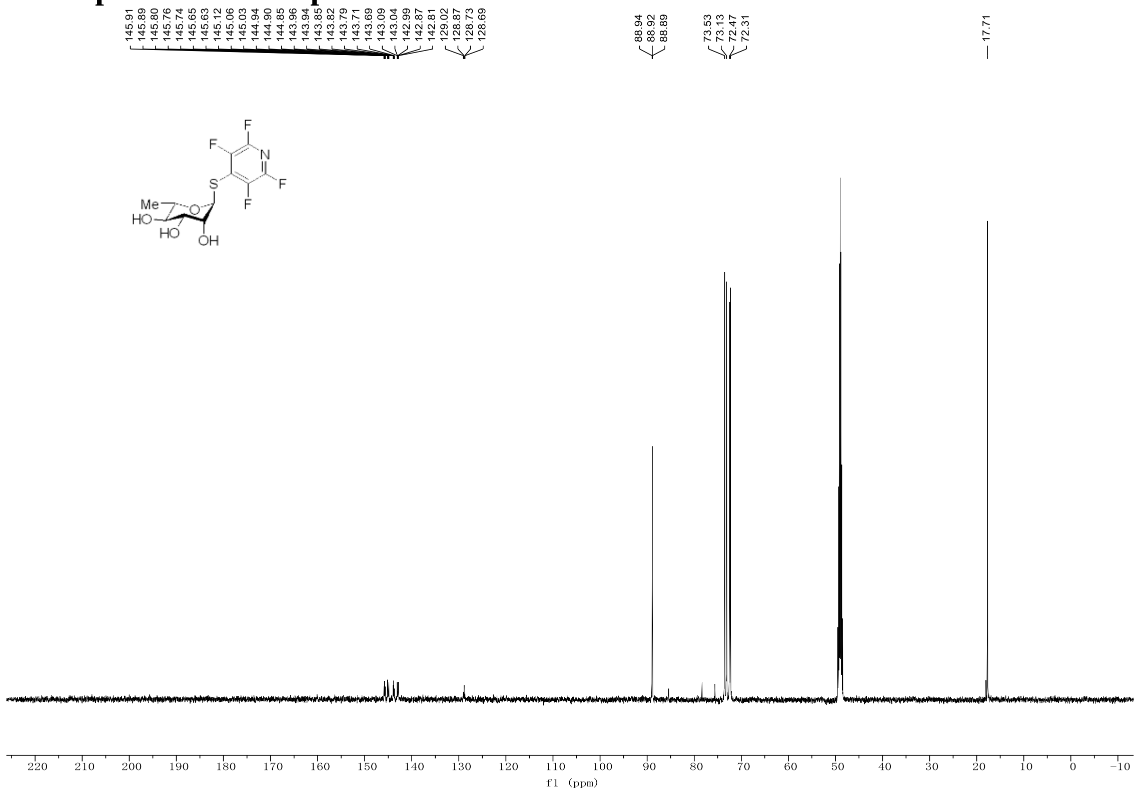

^{19}F NMR spectrum of compound S3

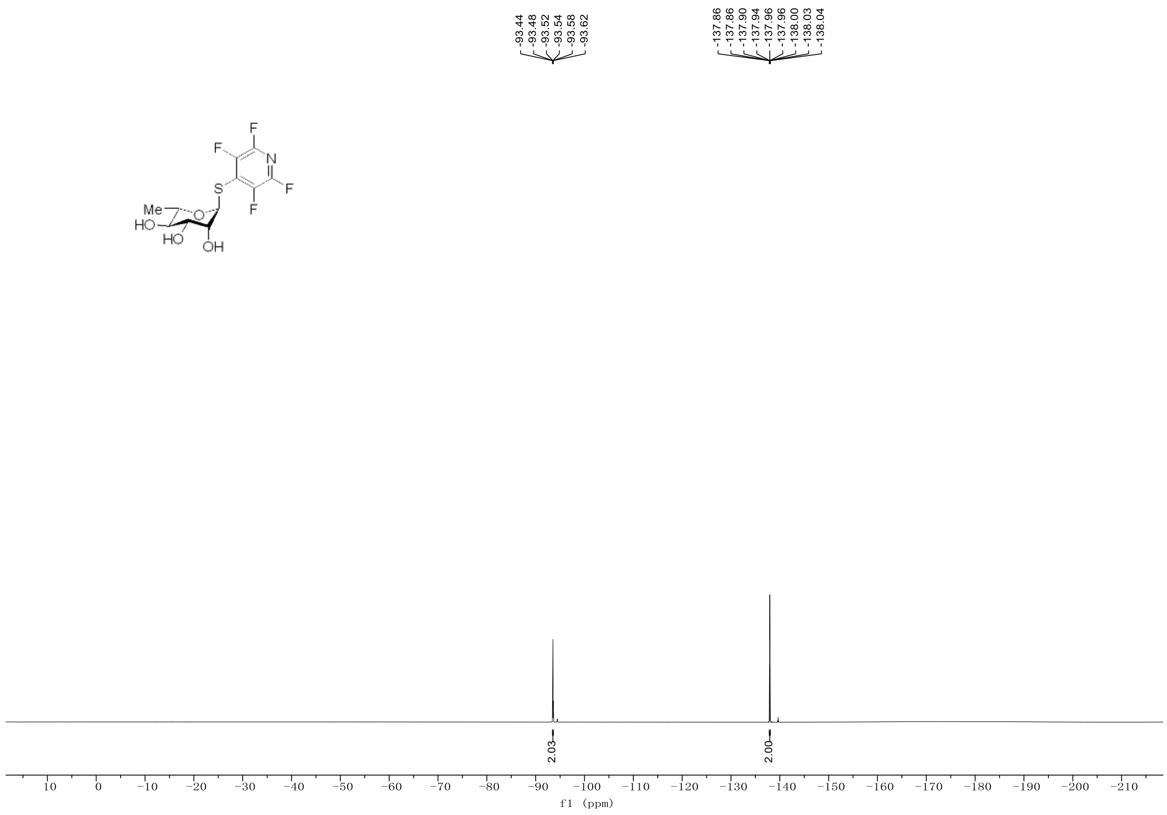

^{1}H NMR spectrum of compound S4

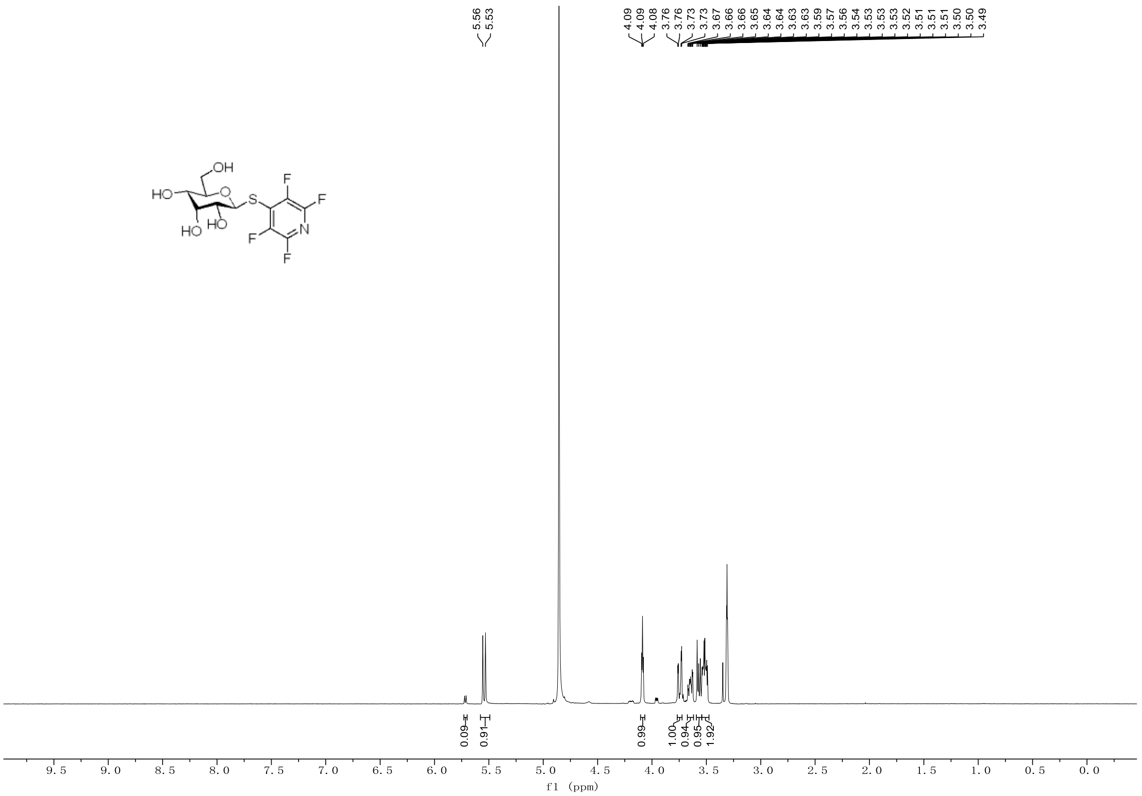

^{13}C NMR spectrum of compound S4

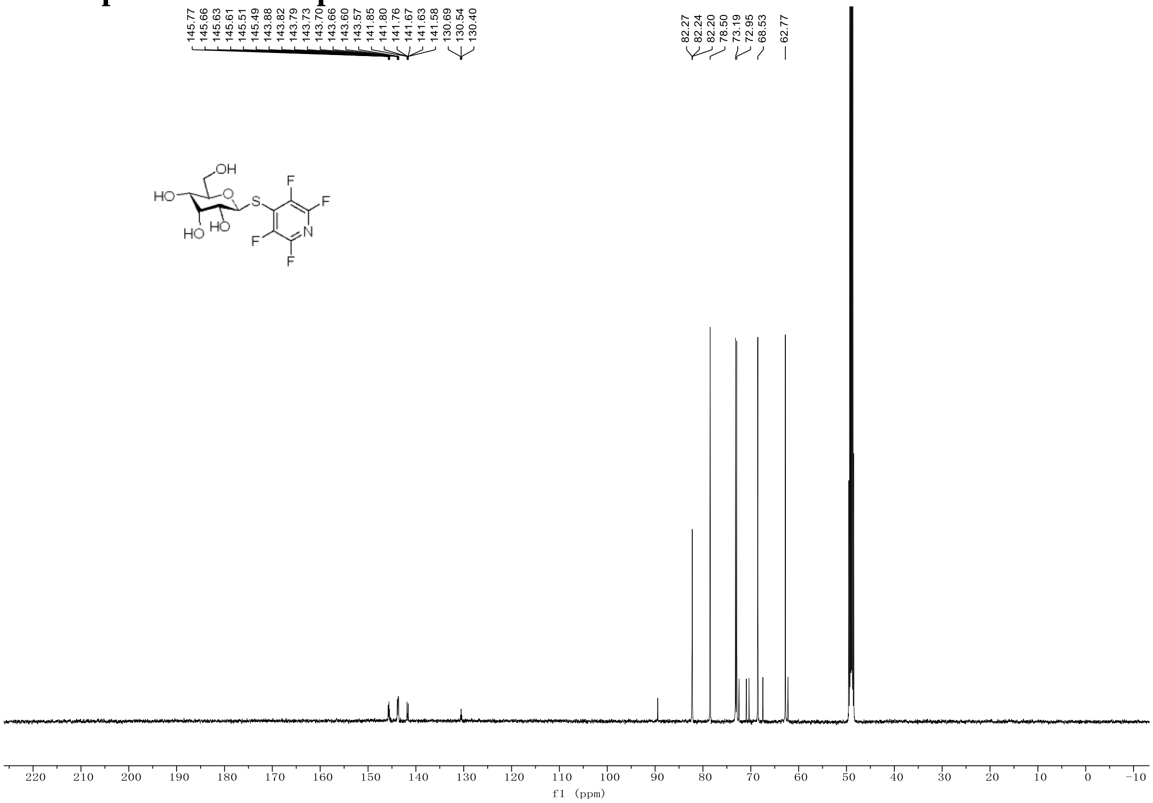

^{19}F NMR spectrum of compound S4

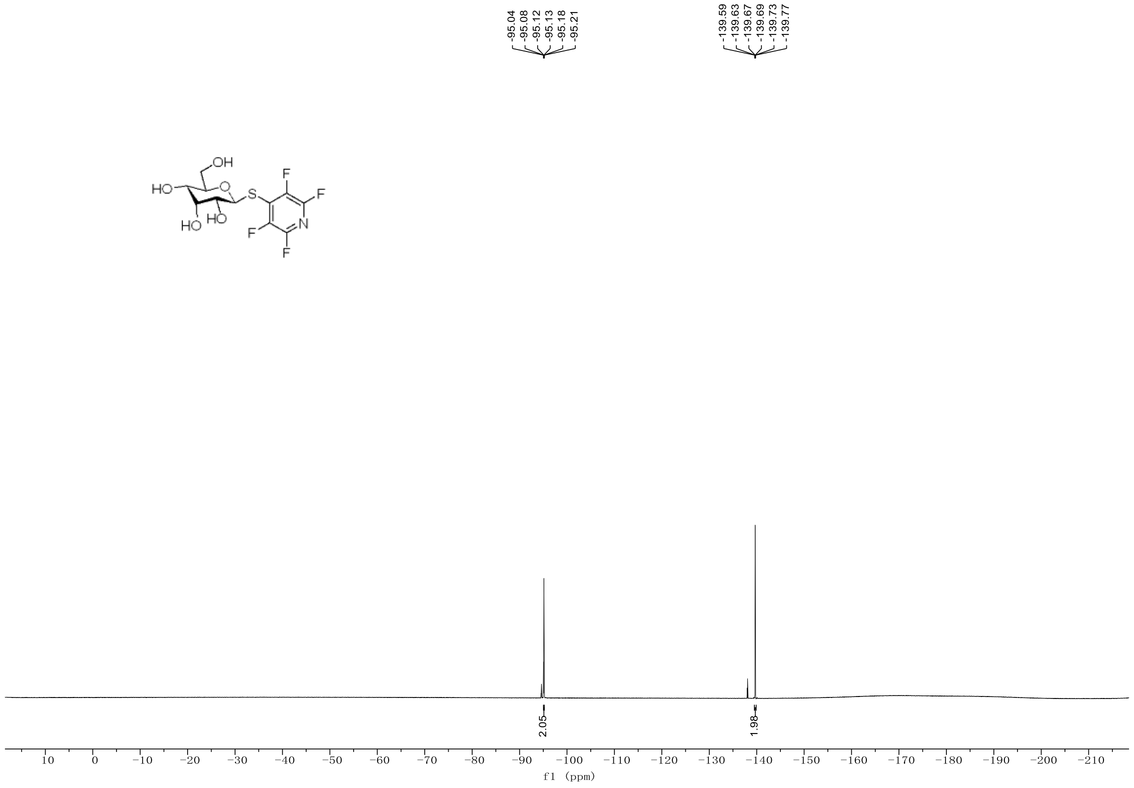

^{1}H NMR spectrum of compound S5

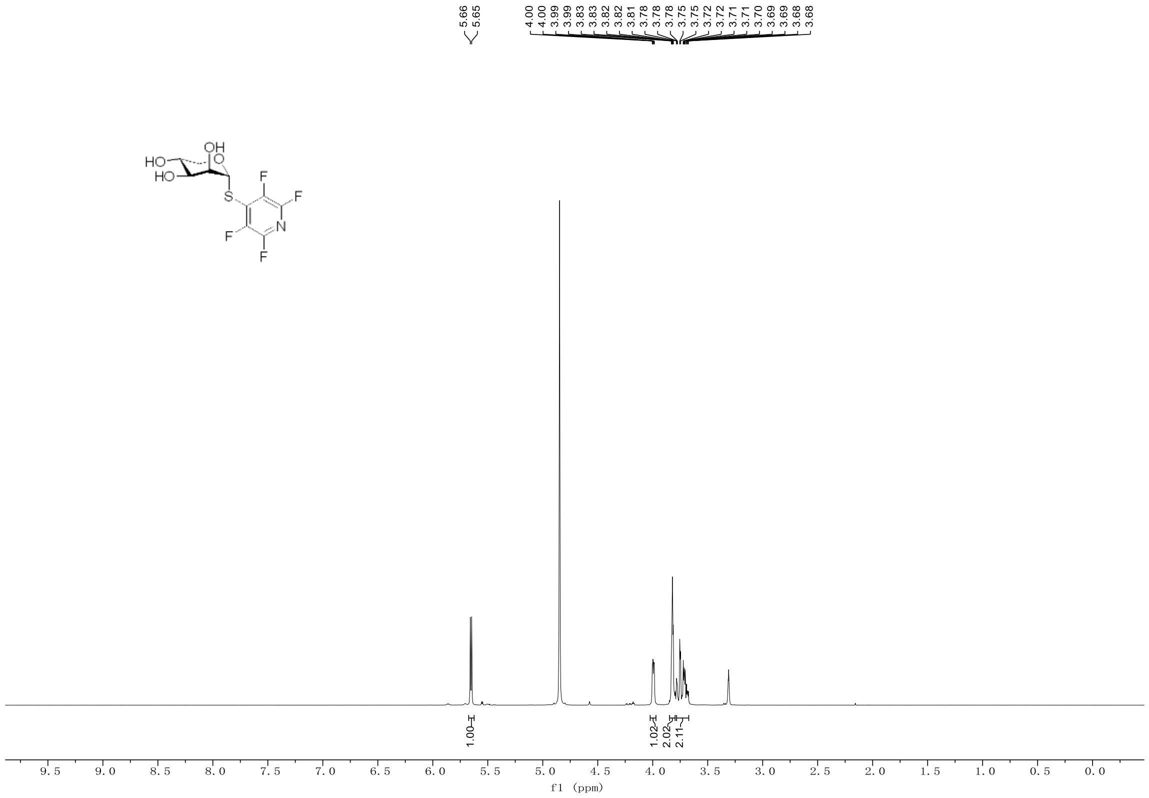

^{13}C NMR spectrum of compound S5

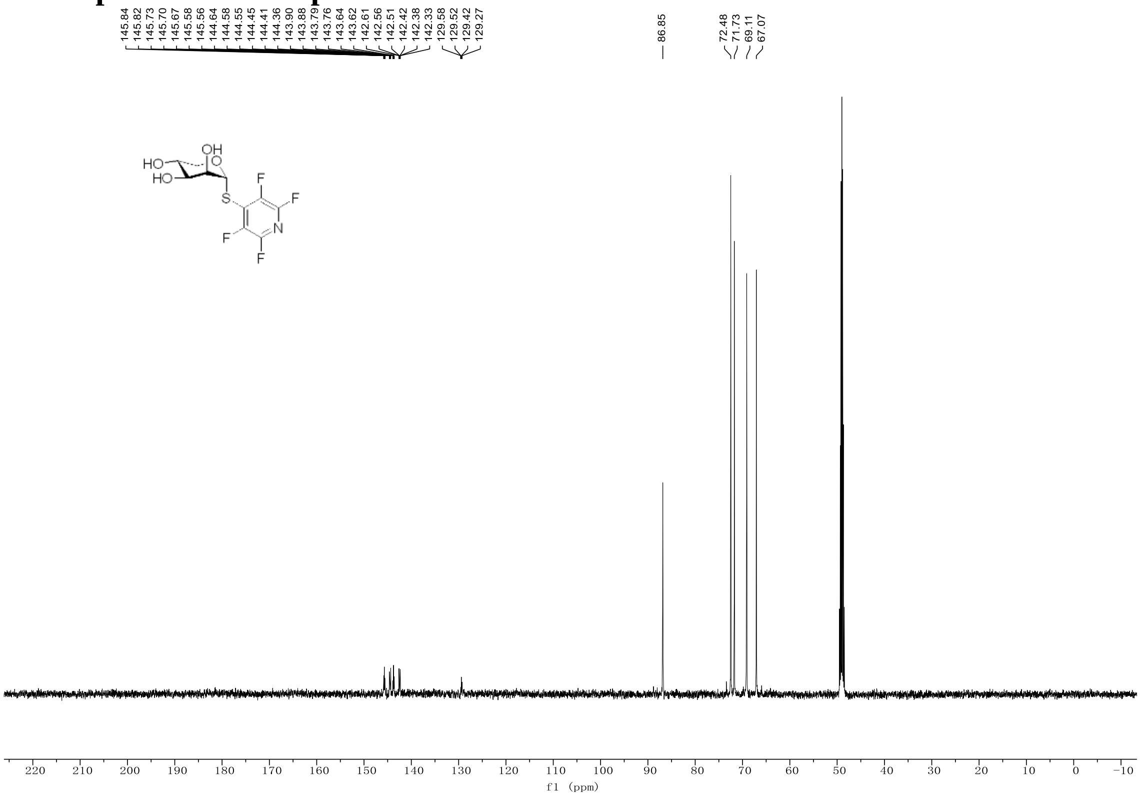

^{19}F NMR spectrum of compound S5

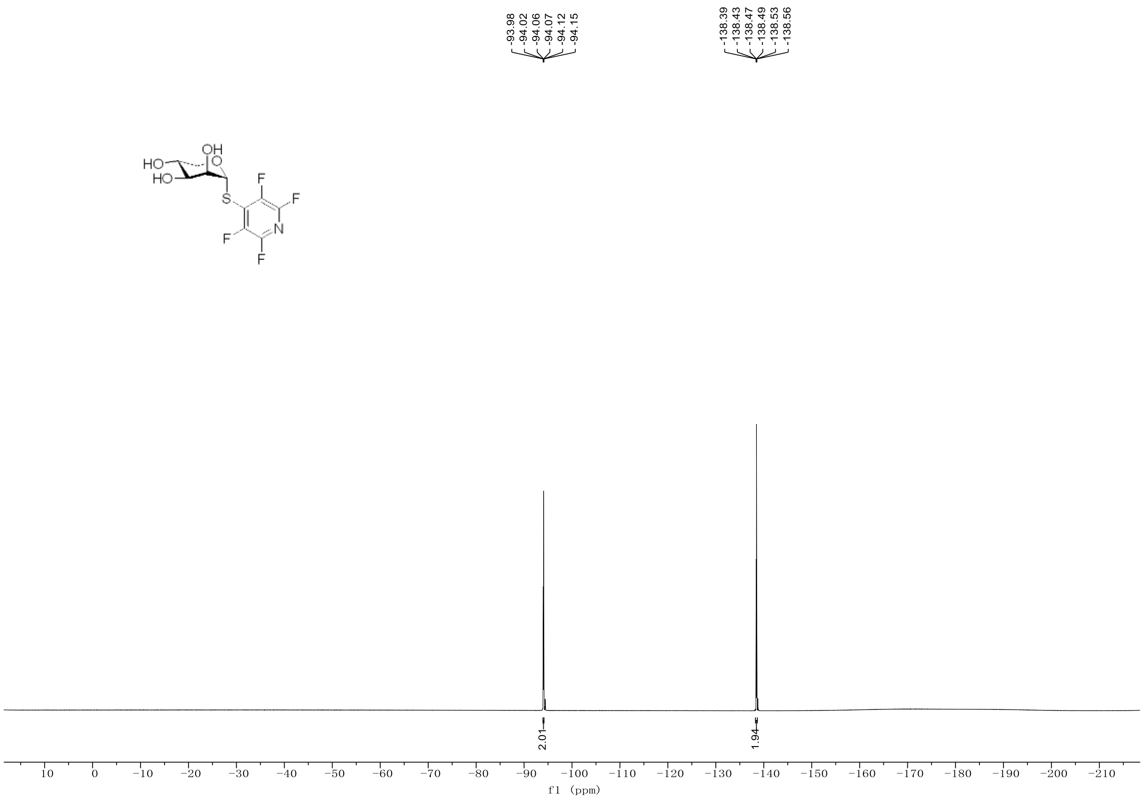

^{1}H NMR spectrum of compound S6

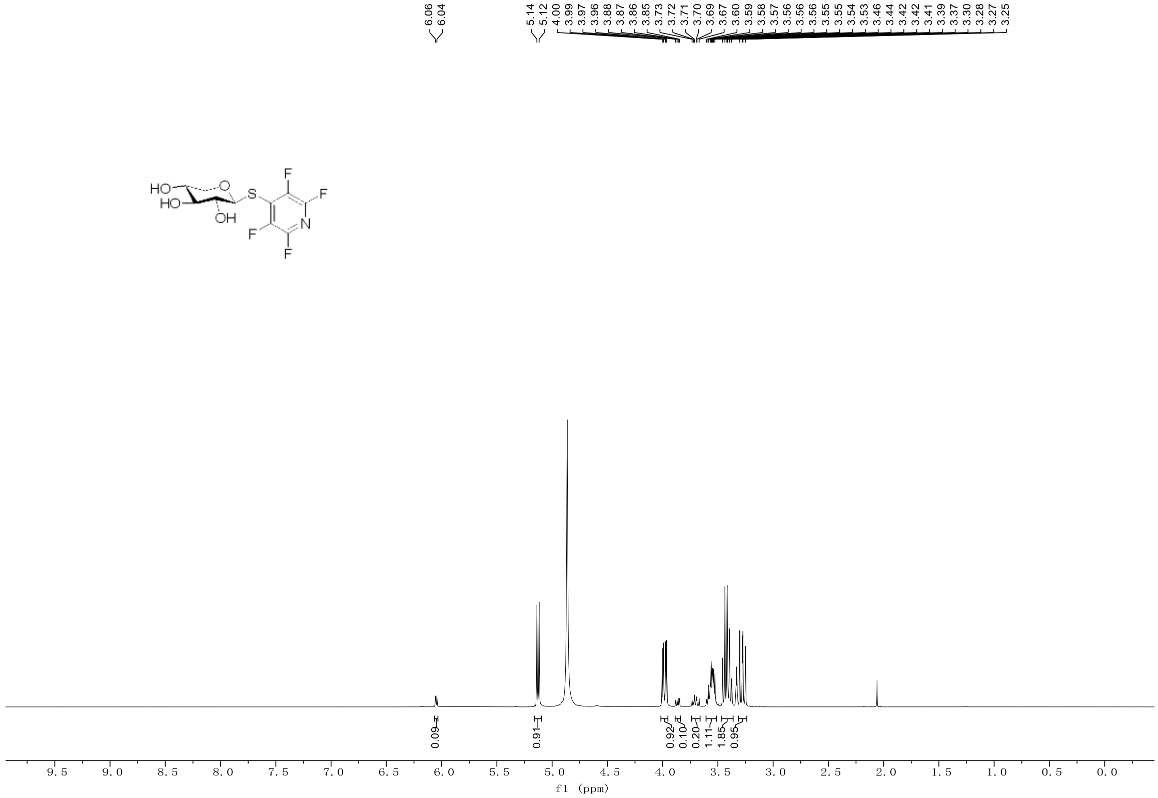

^{13}C NMR spectrum of compound S6

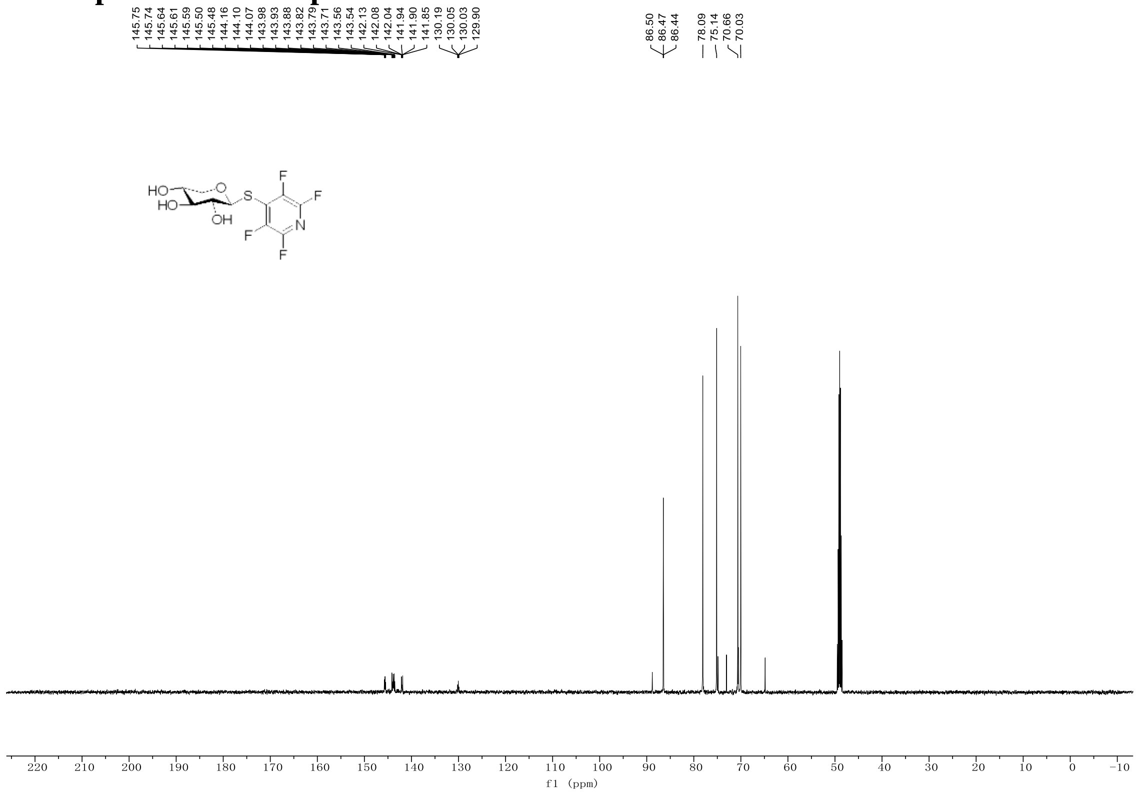

^{19}F NMR spectrum of compound S6

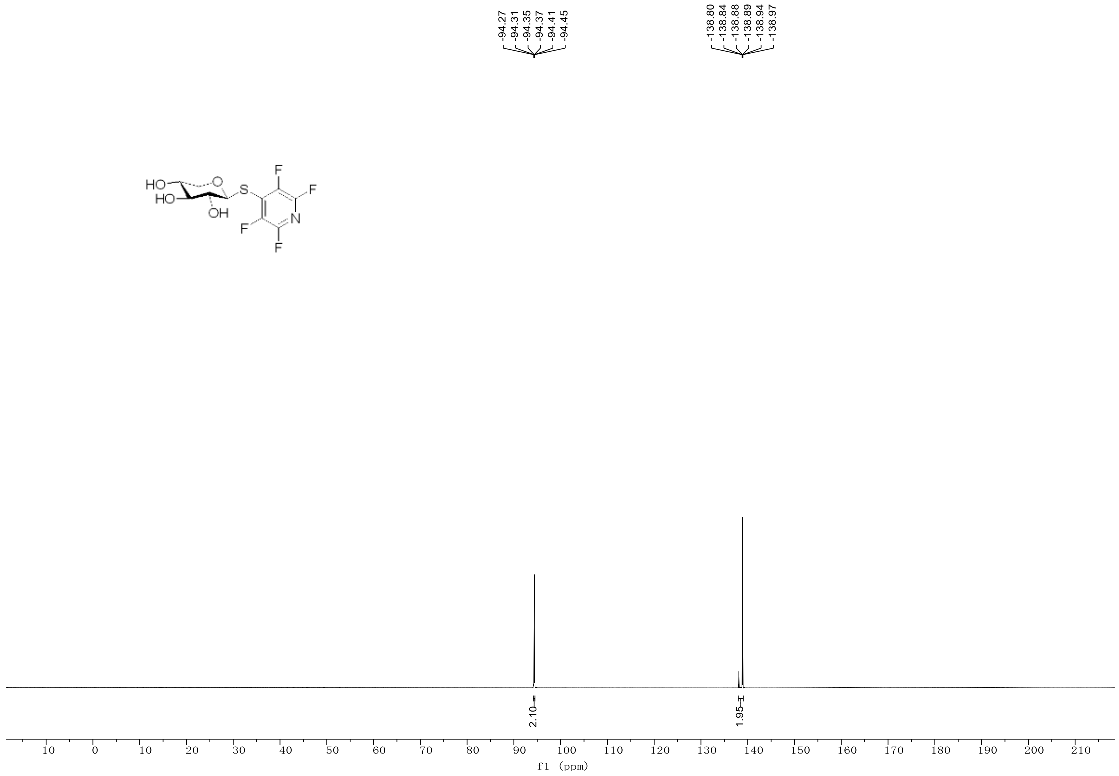

^{1}H NMR spectrum of compound S7

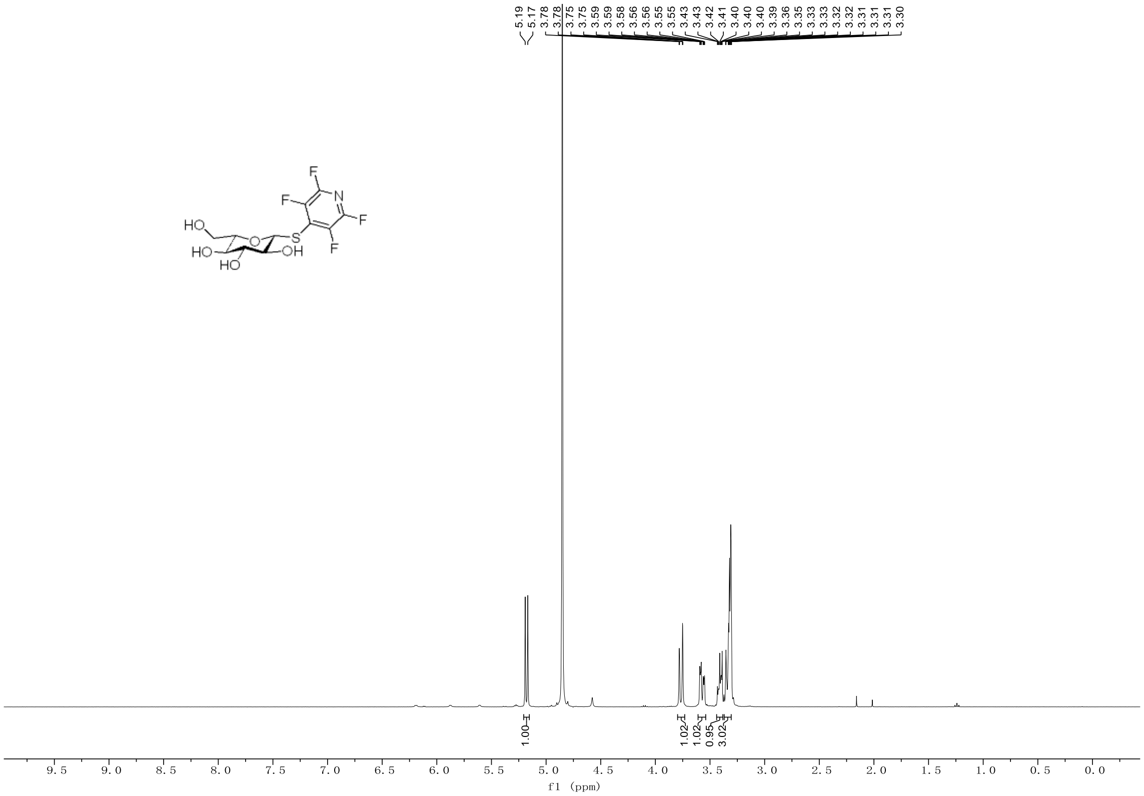

^{13}C NMR spectrum of compound S7

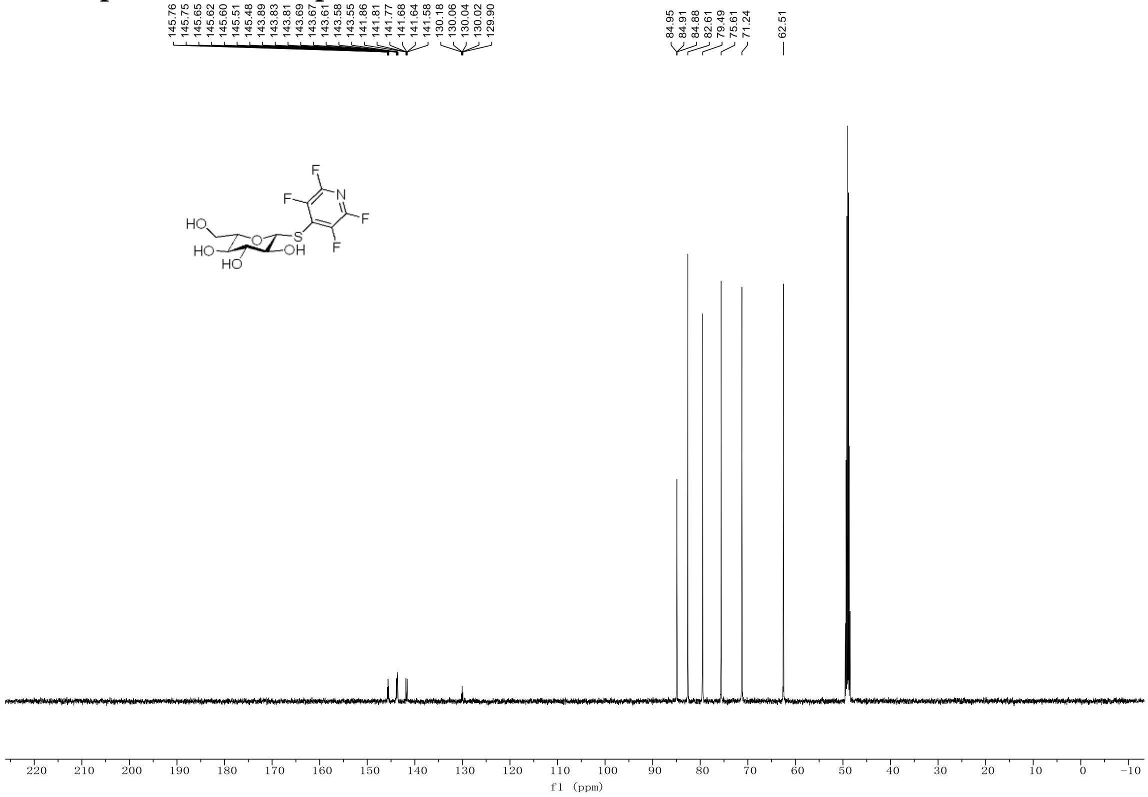

^{19}F NMR spectrum of compound S7

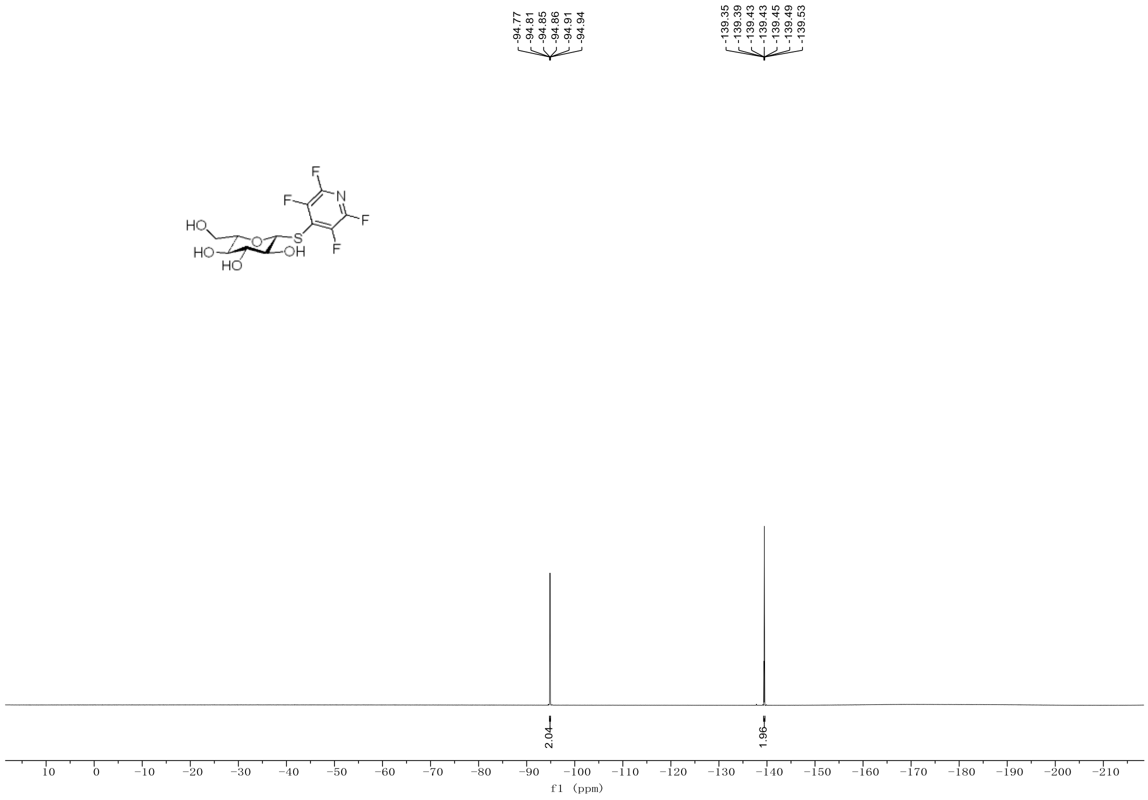

^{1}H NMR spectrum of compound 13

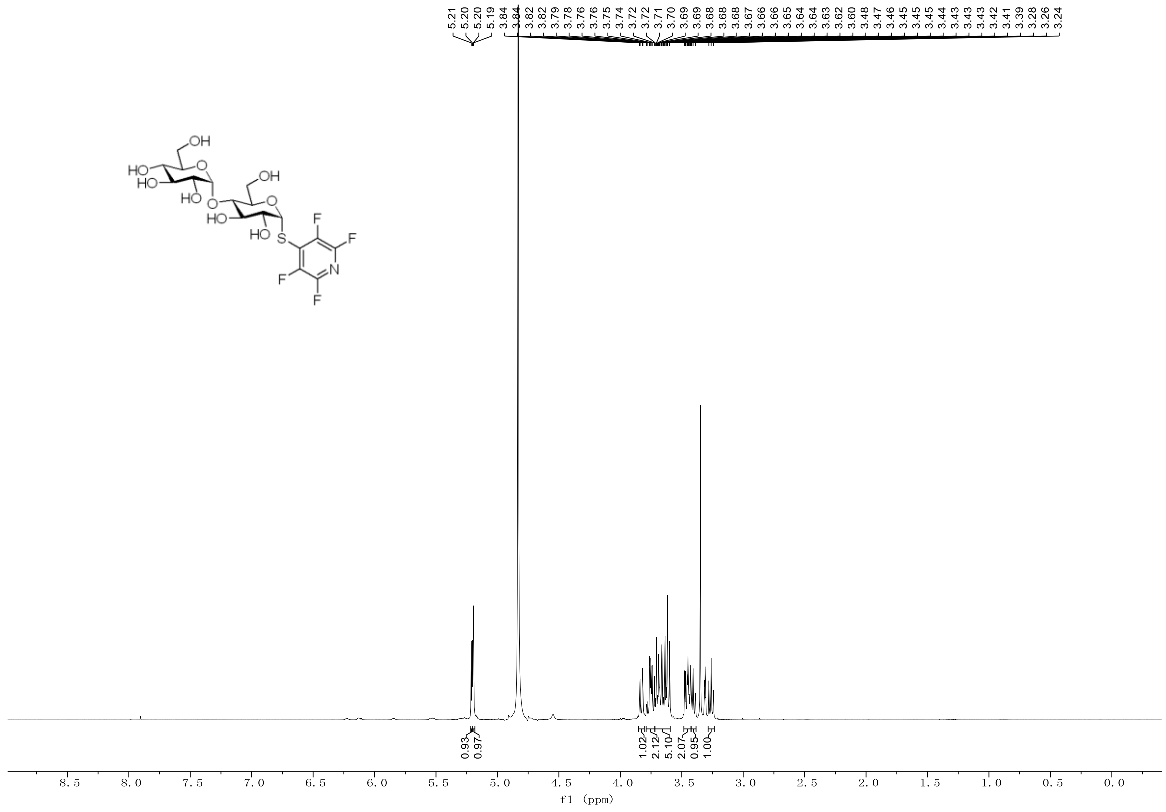

^{13}C NMR spectrum of compound 13

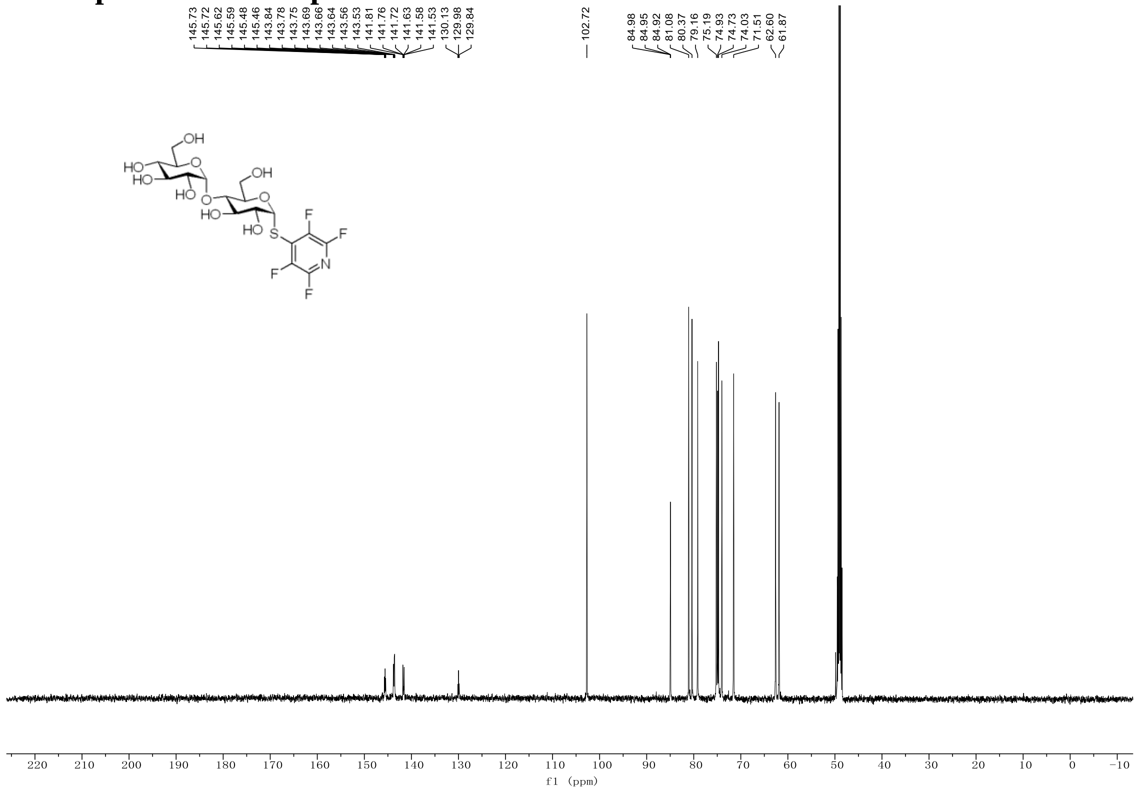

^{19}F NMR spectrum of compound 13

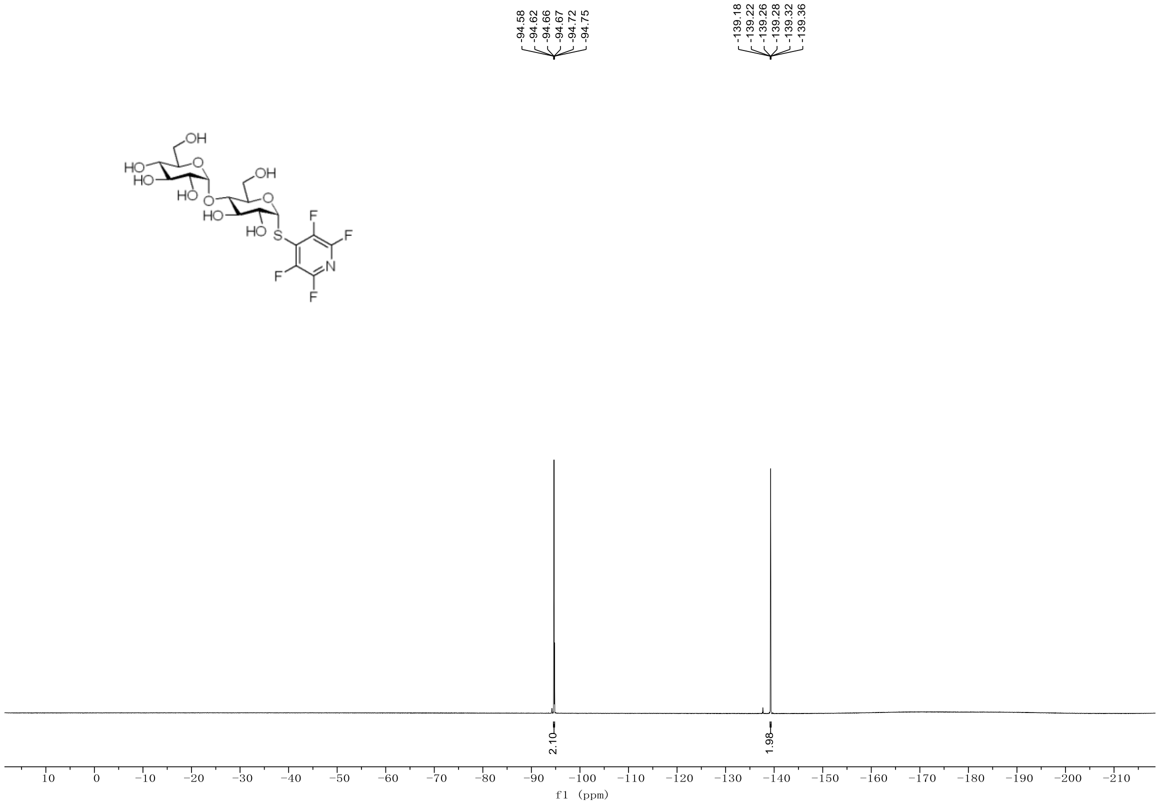

^{1}H NMR spectrum of compound S8

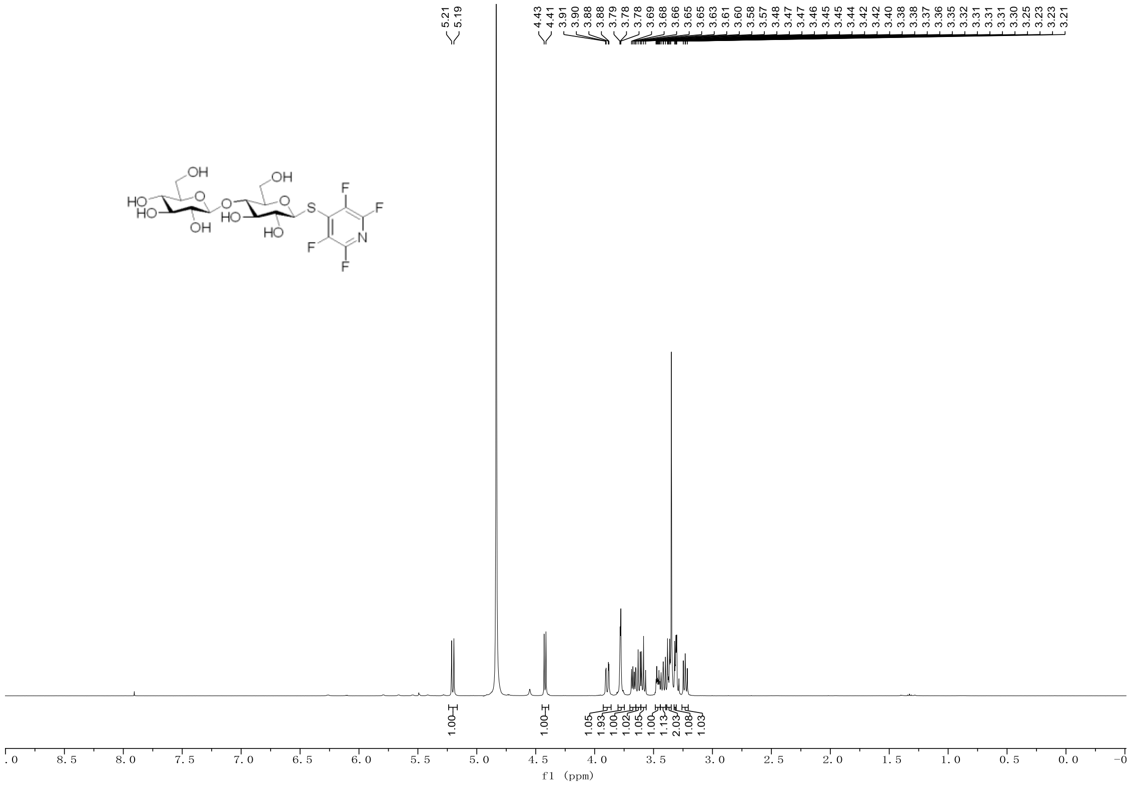

^{13}C NMR spectrum of compound S8

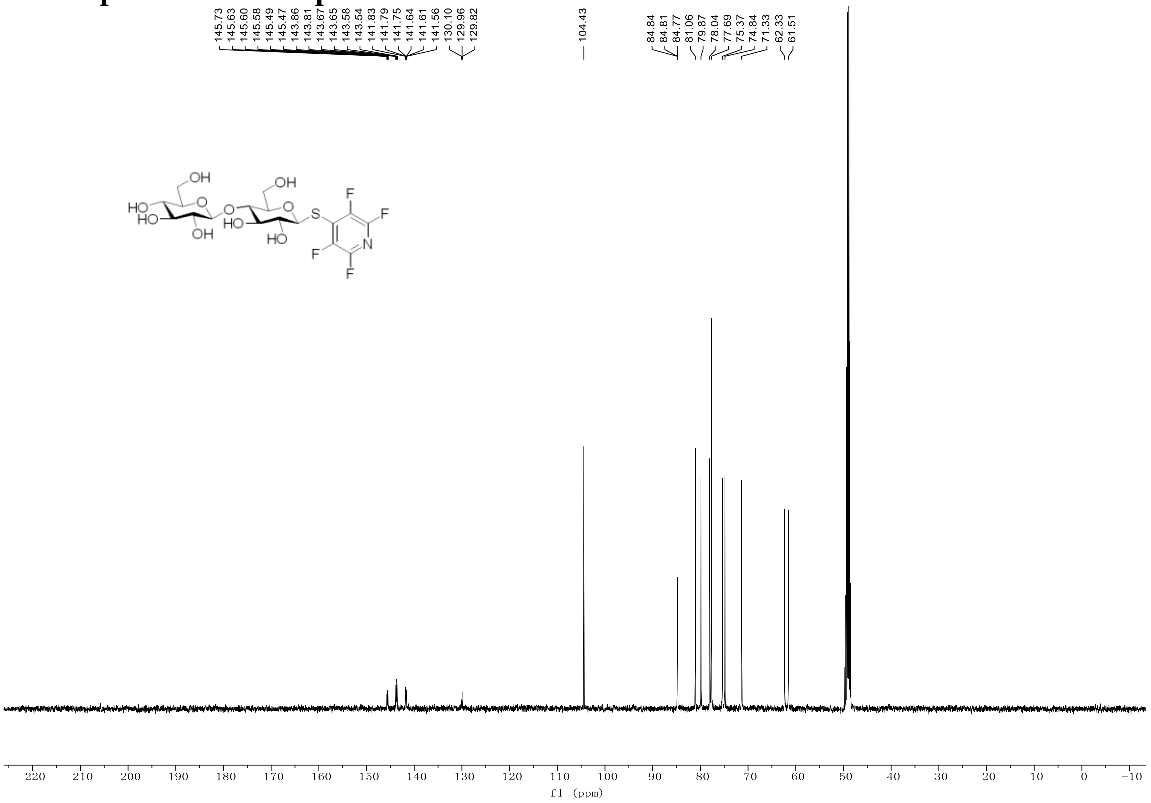

^{19}F NMR spectrum of compound S8

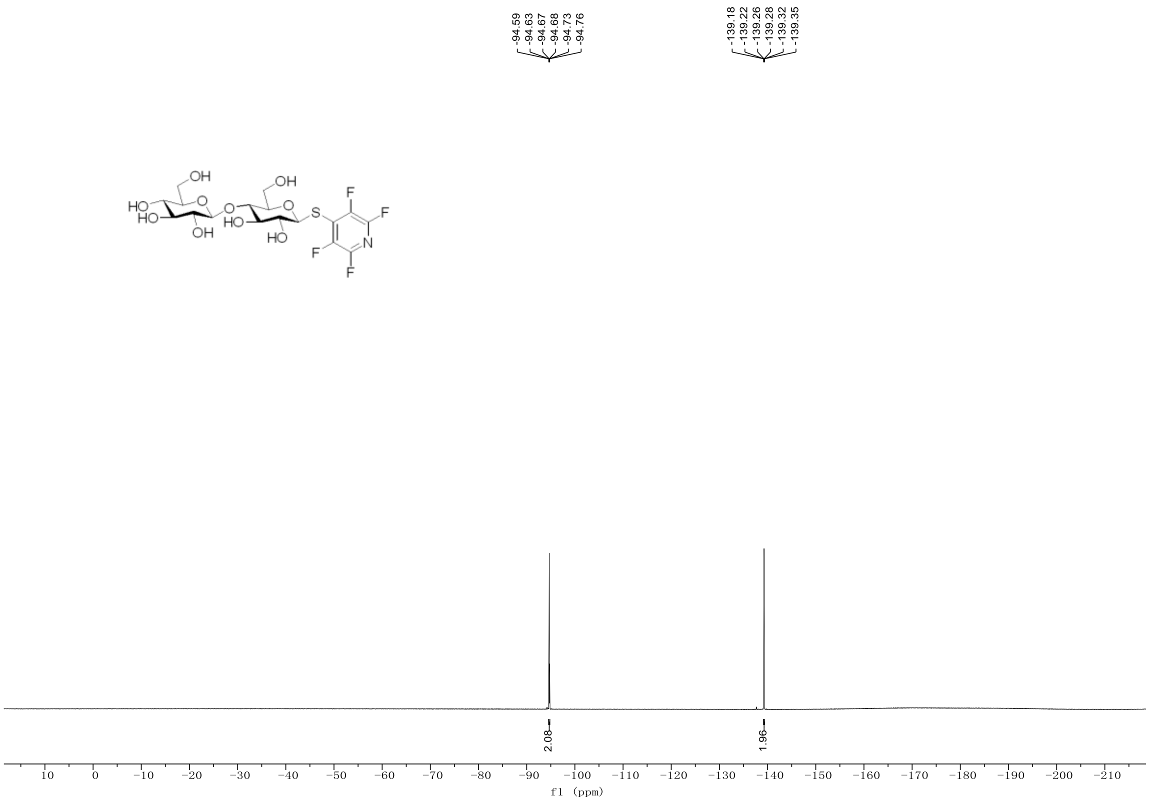

^{1}H NMR spectrum of compound S9

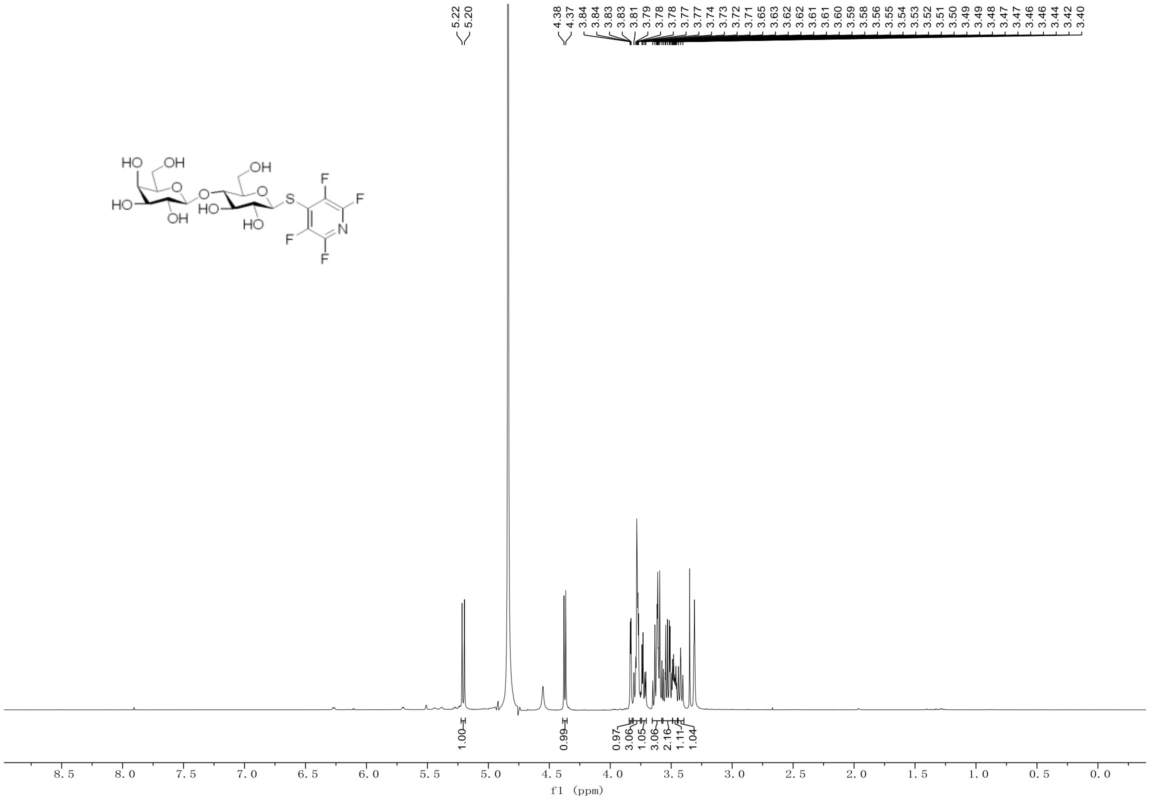

^{13}C NMR spectrum of compound S9

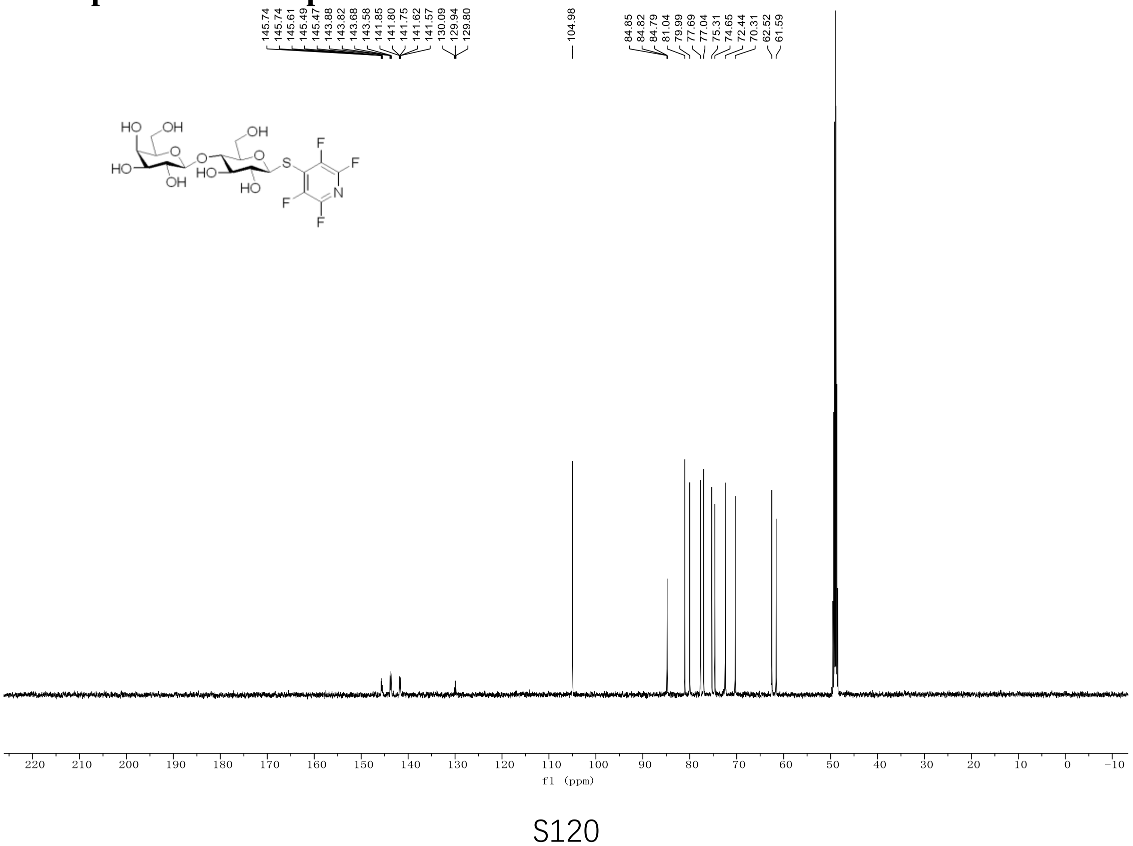

^{19}F NMR spectrum of compound S9

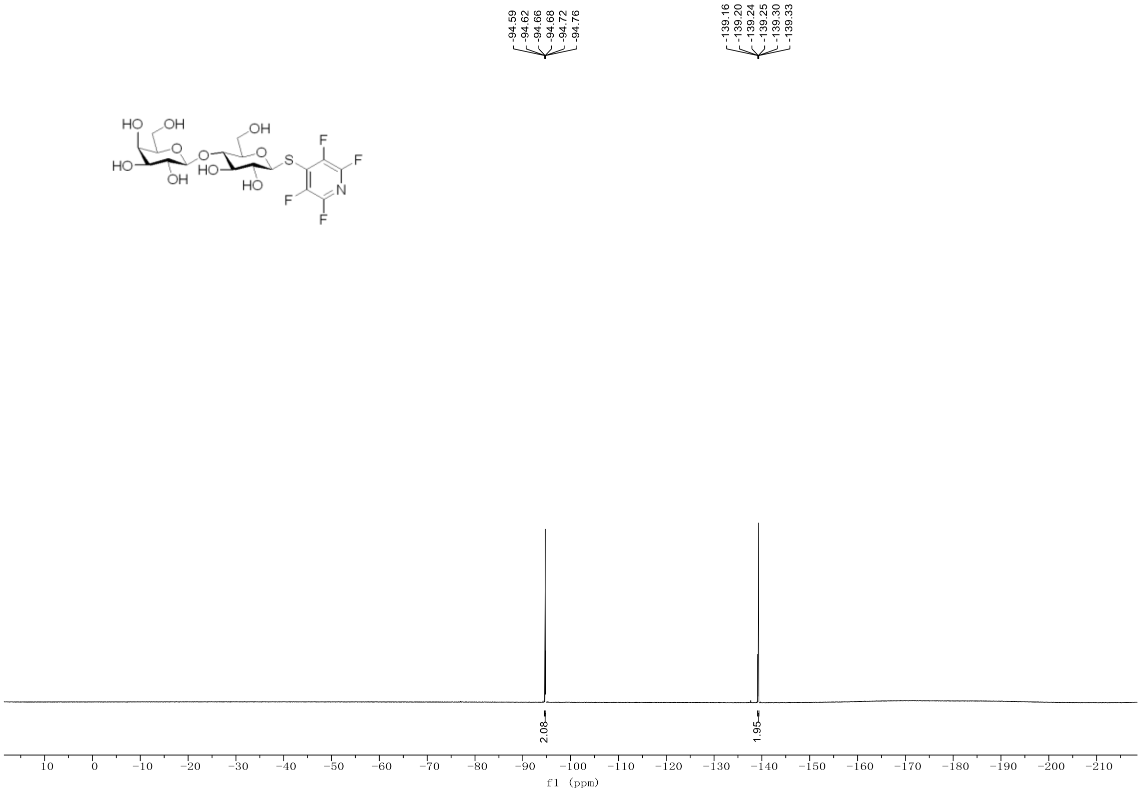

^{1}H NMR spectrum of compound S10

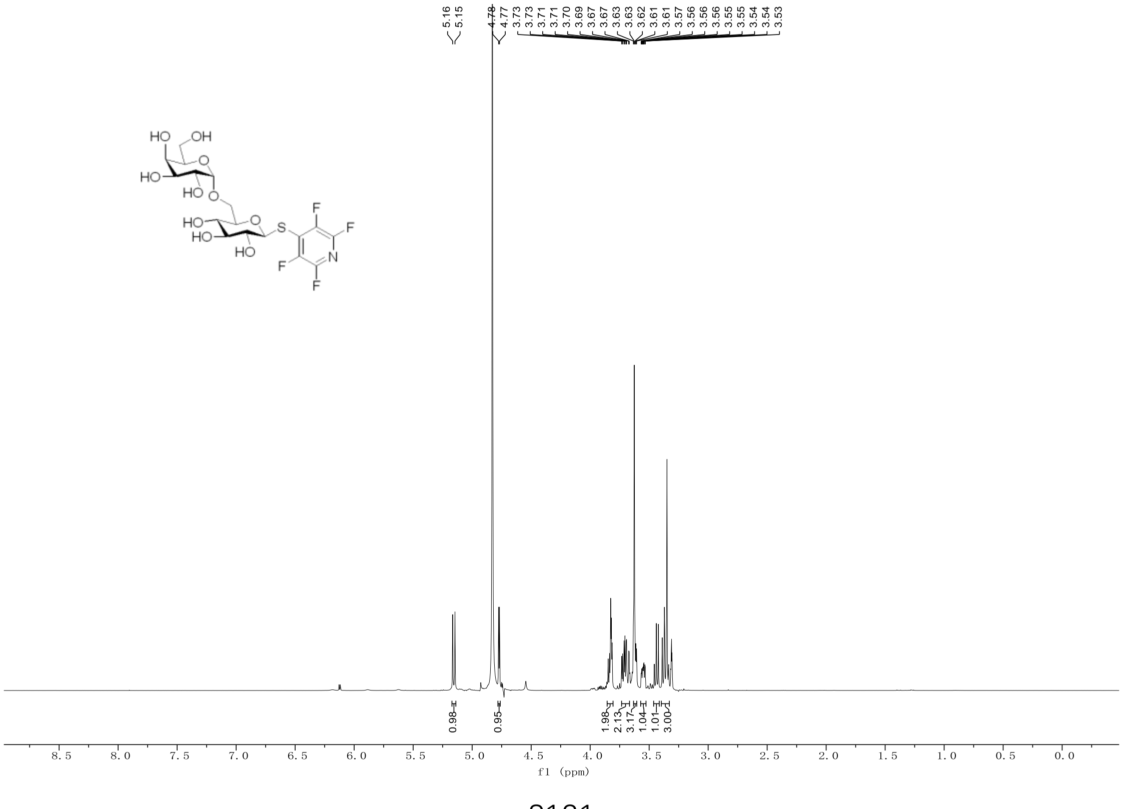

^{13}C NMR spectrum of compound S10

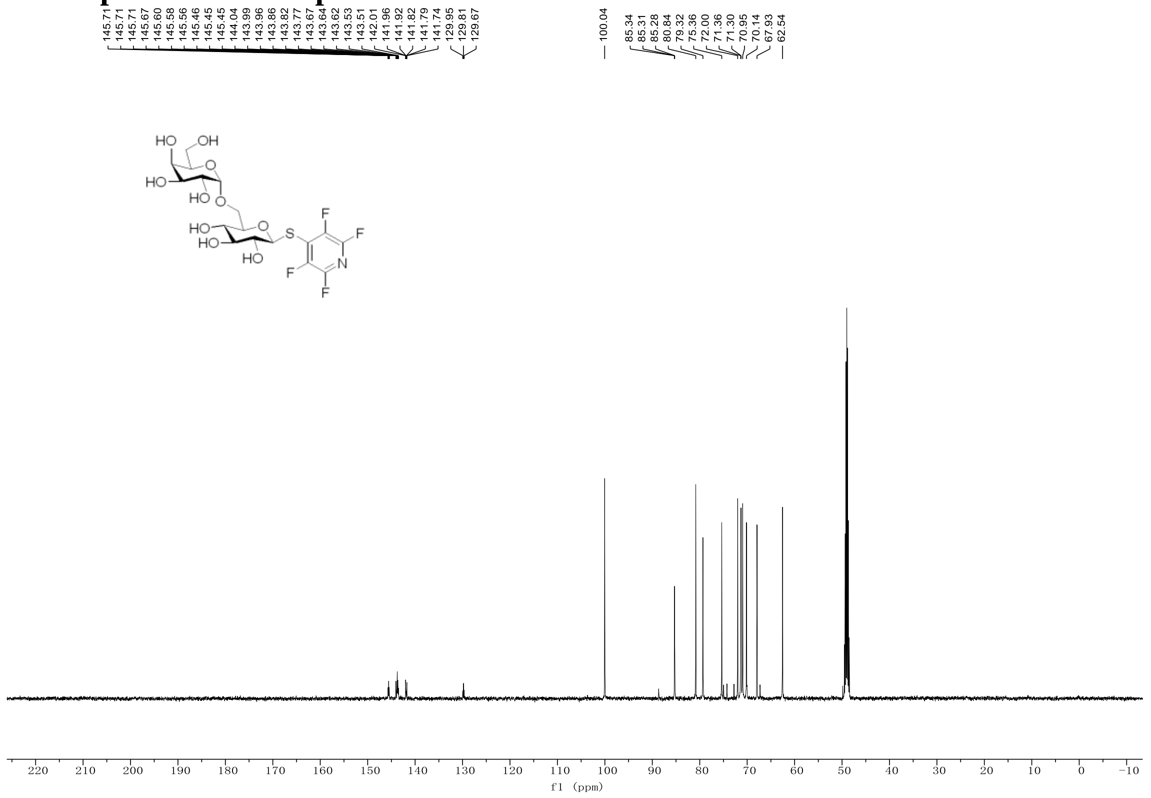

^{19}F NMR spectrum of compound S10

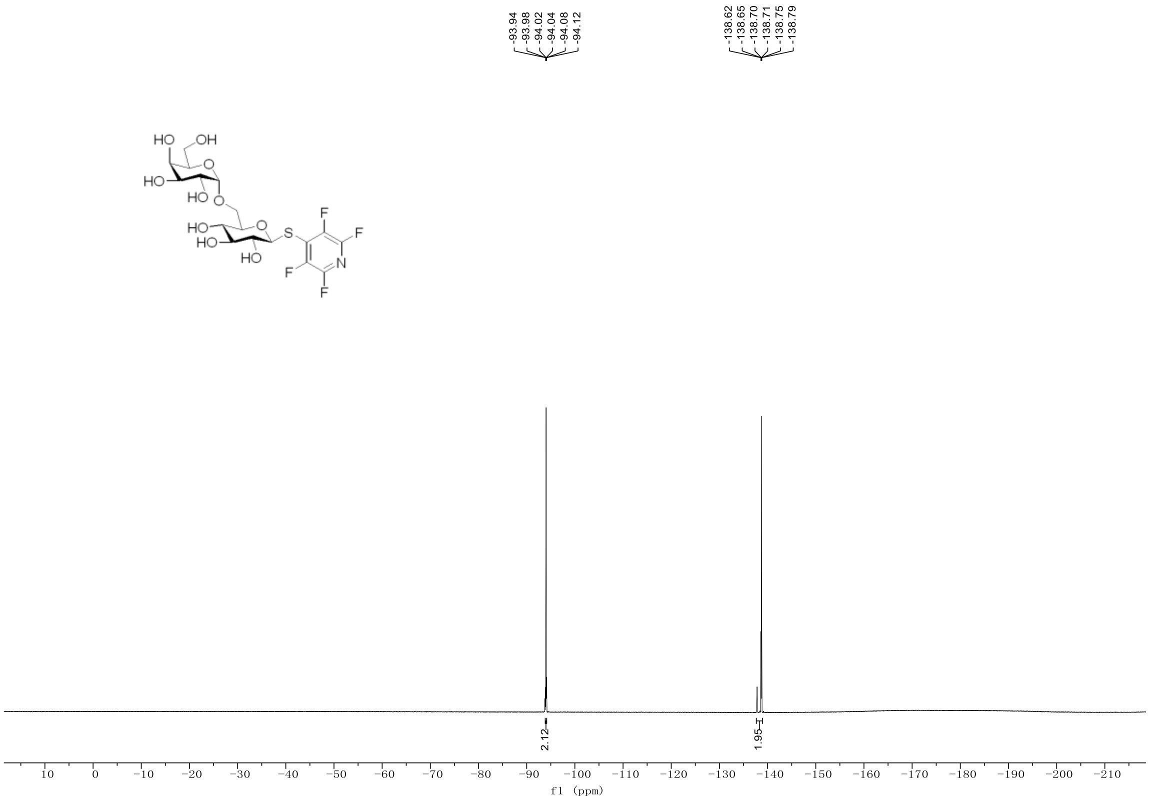

^{1}H NMR spectrum of compound S11

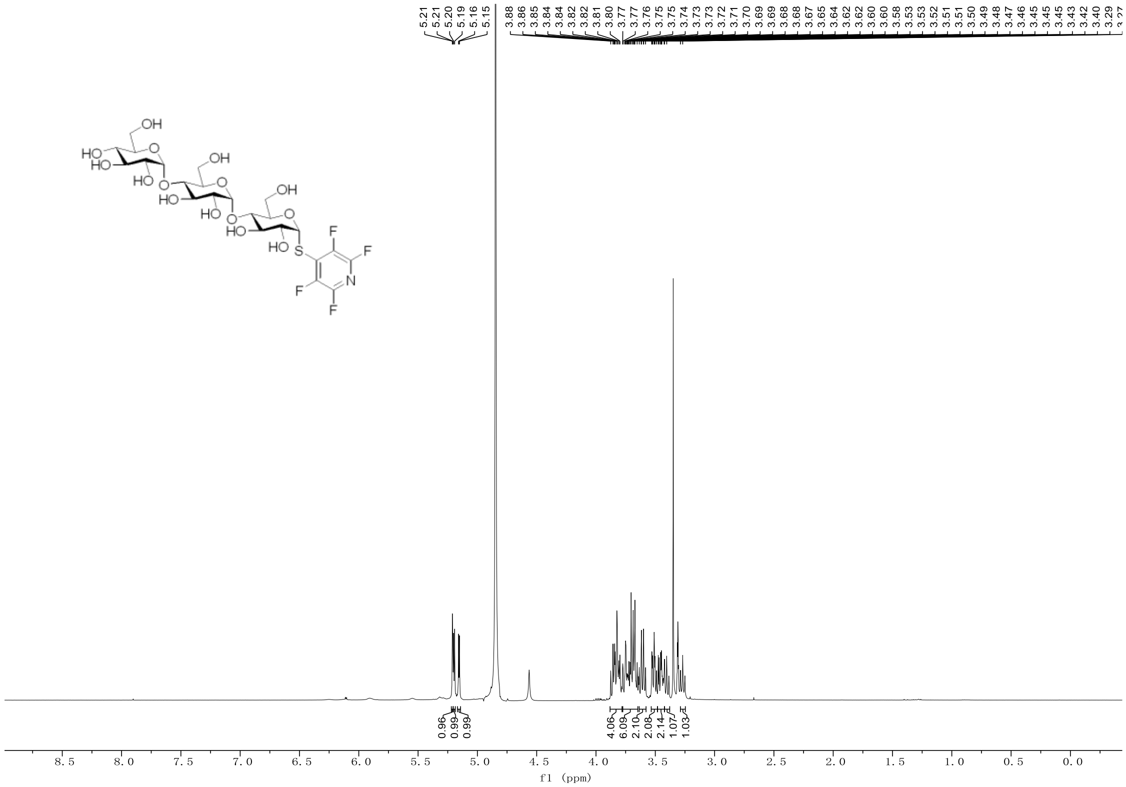

^{13}C NMR spectrum of compound S11

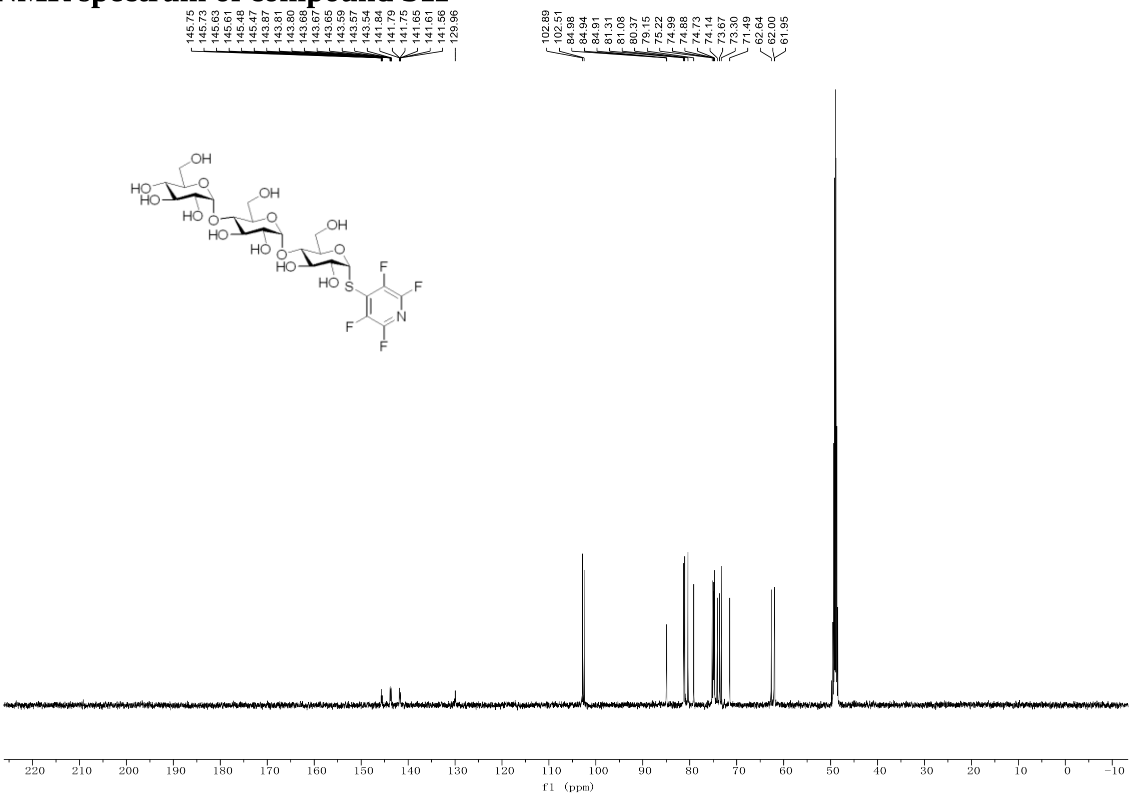

^{19}F NMR spectrum of compound S11

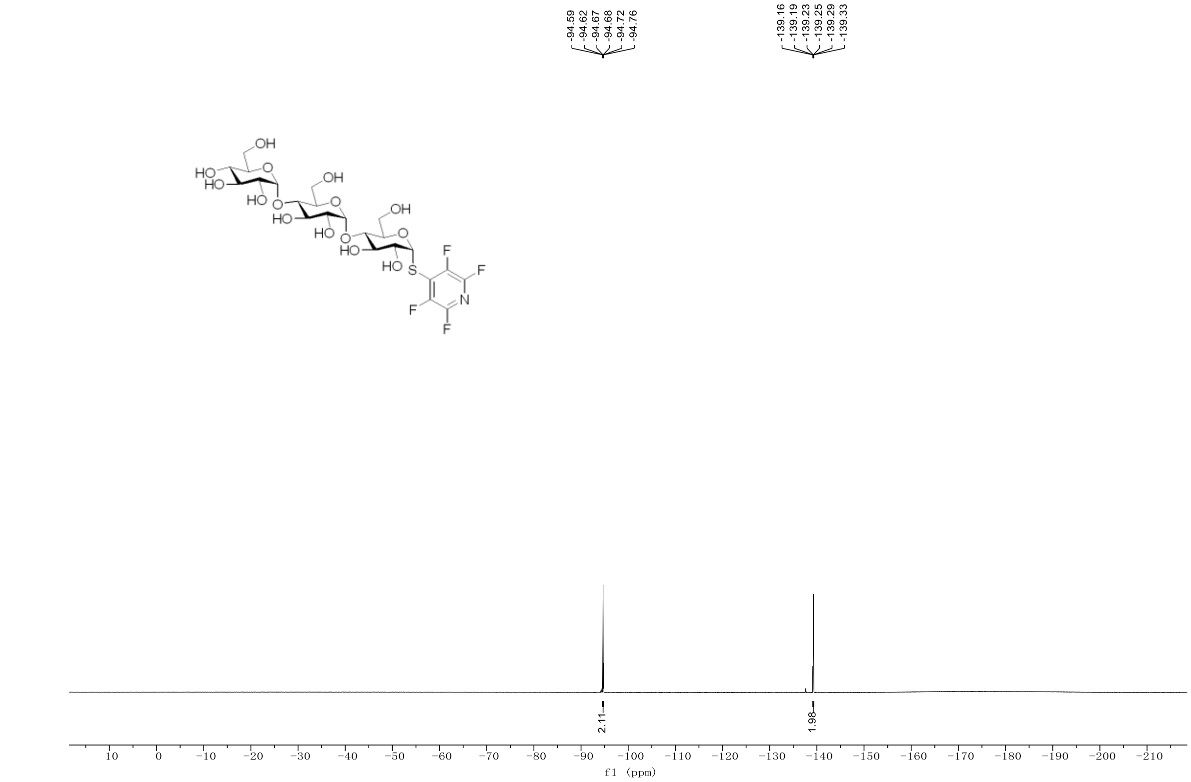

^{1}H NMR spectrum of compound S35

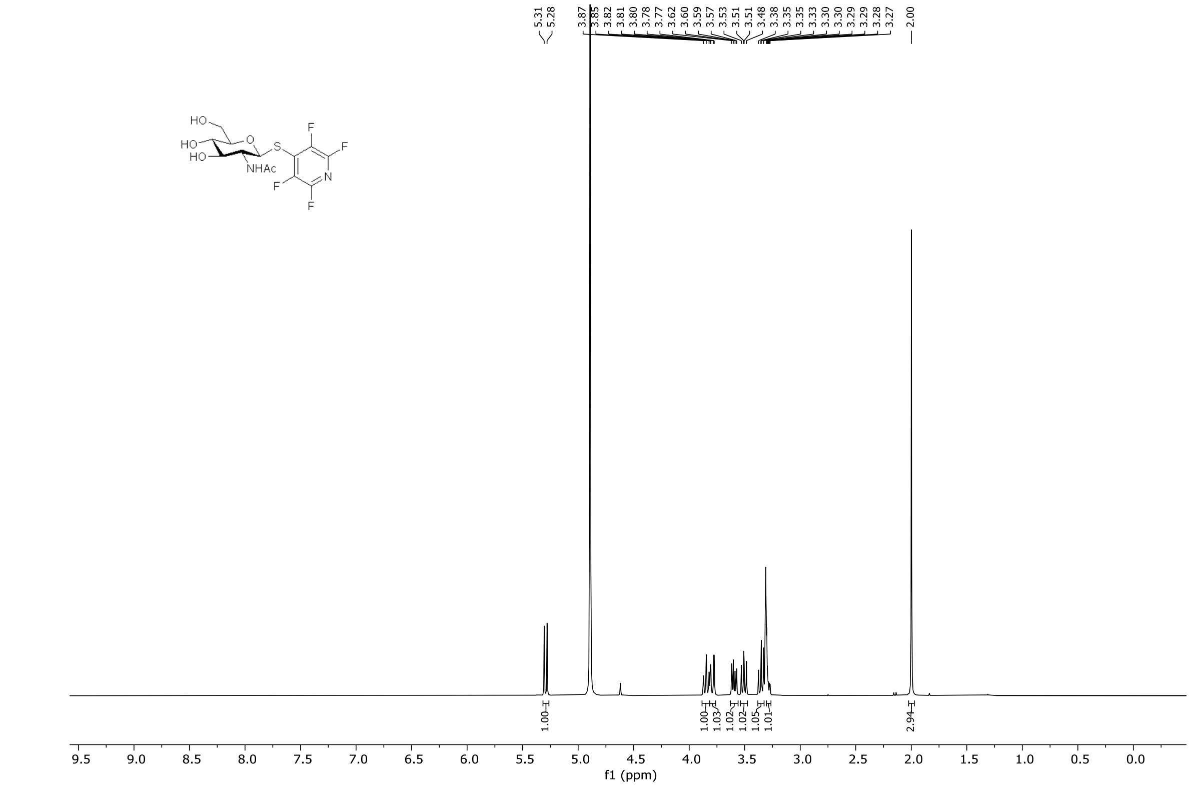

^{13}C NMR spectrum of compound S35

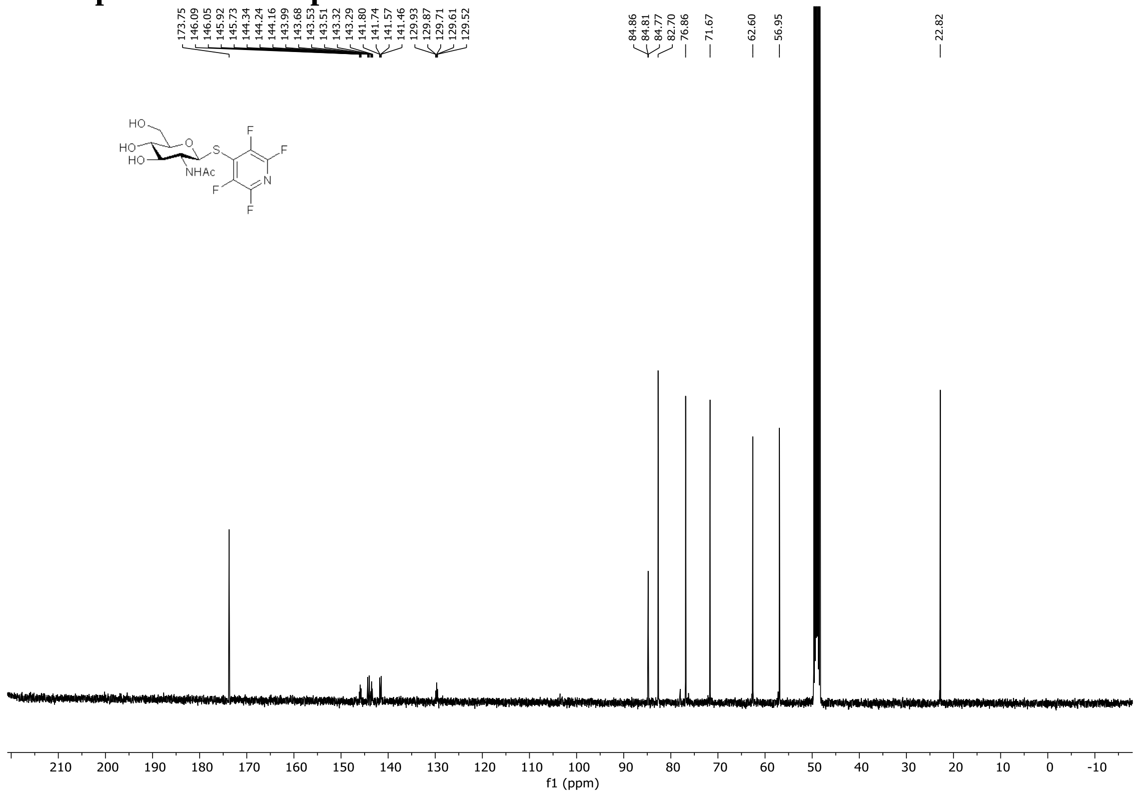

^{19}F NMR spectrum of compound S35

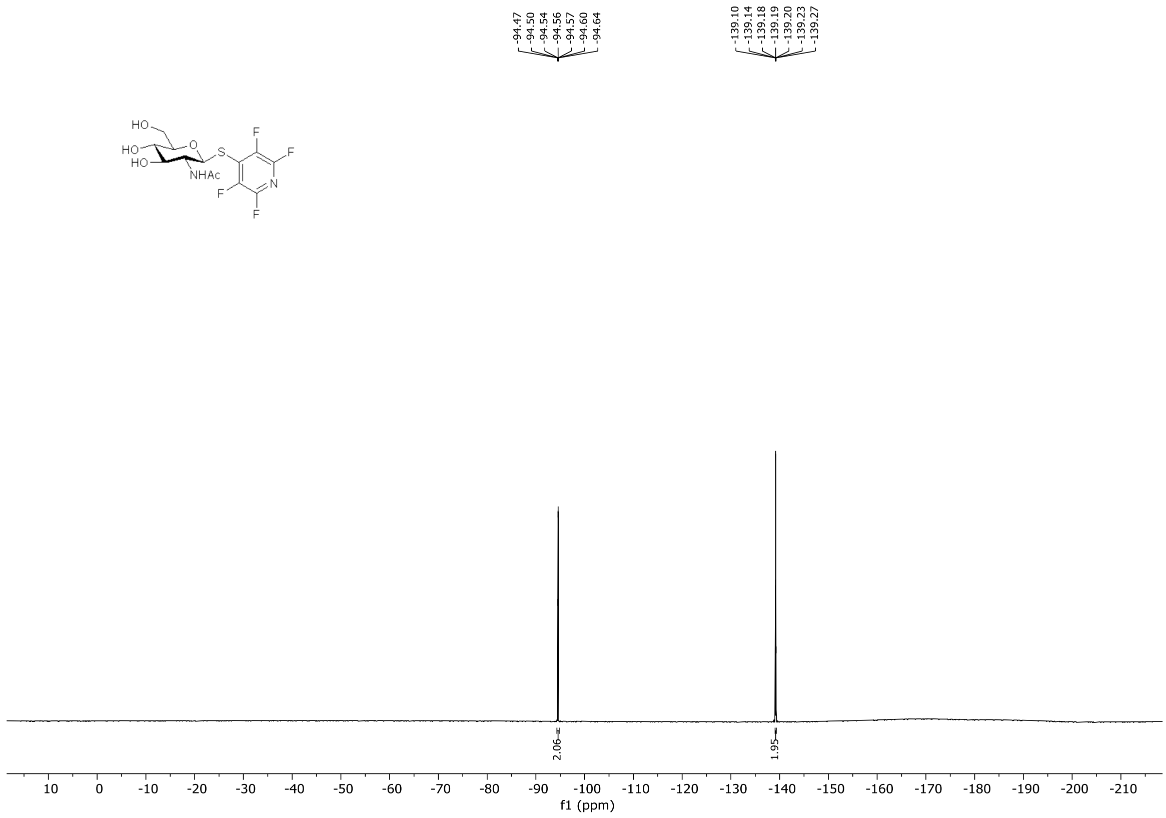

^{1}H NMR spectrum of compound S12

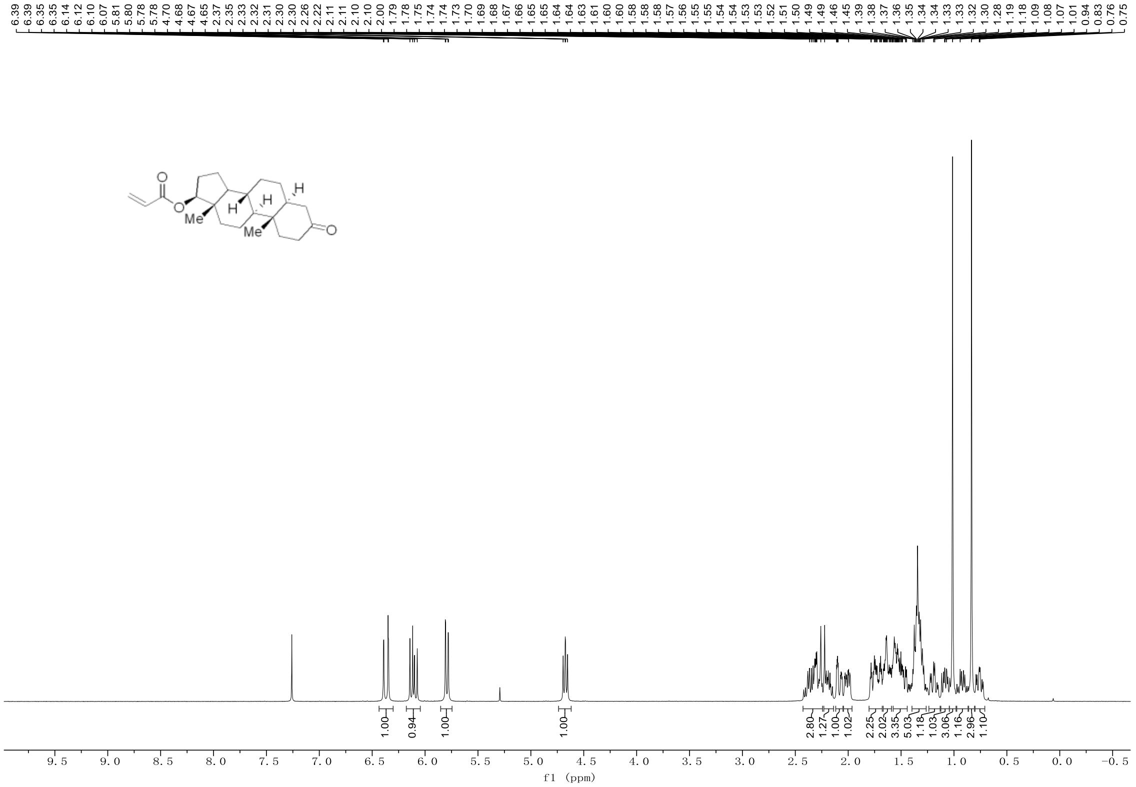

^{13}C NMR spectrum of compound S12

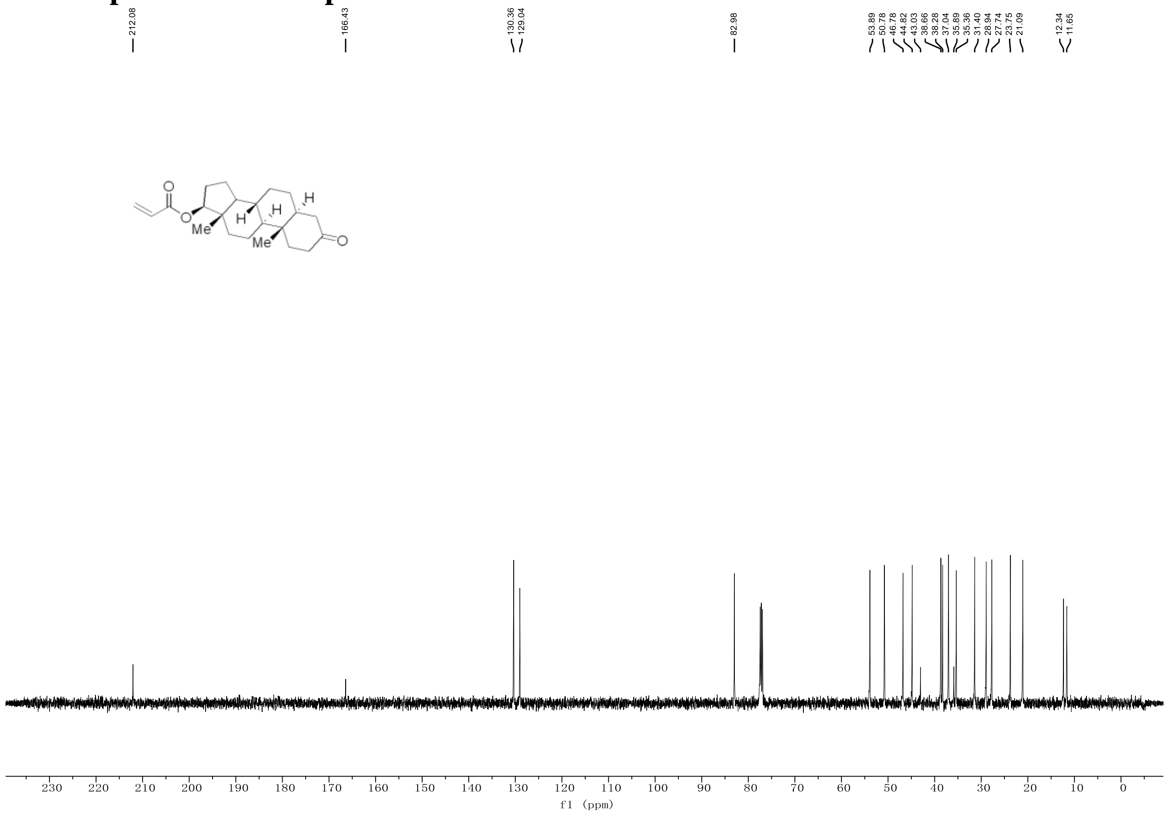

^{1}H NMR spectrum of compound S13

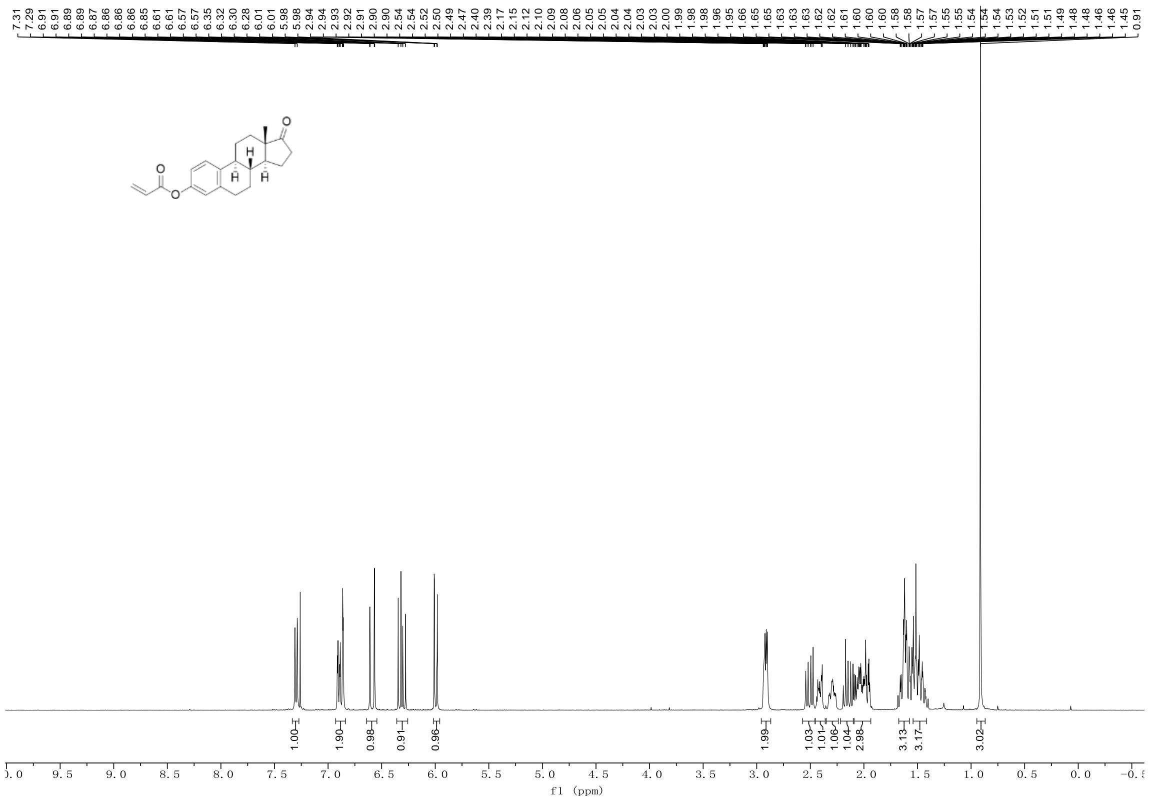

^{13}C NMR spectrum of compound S13

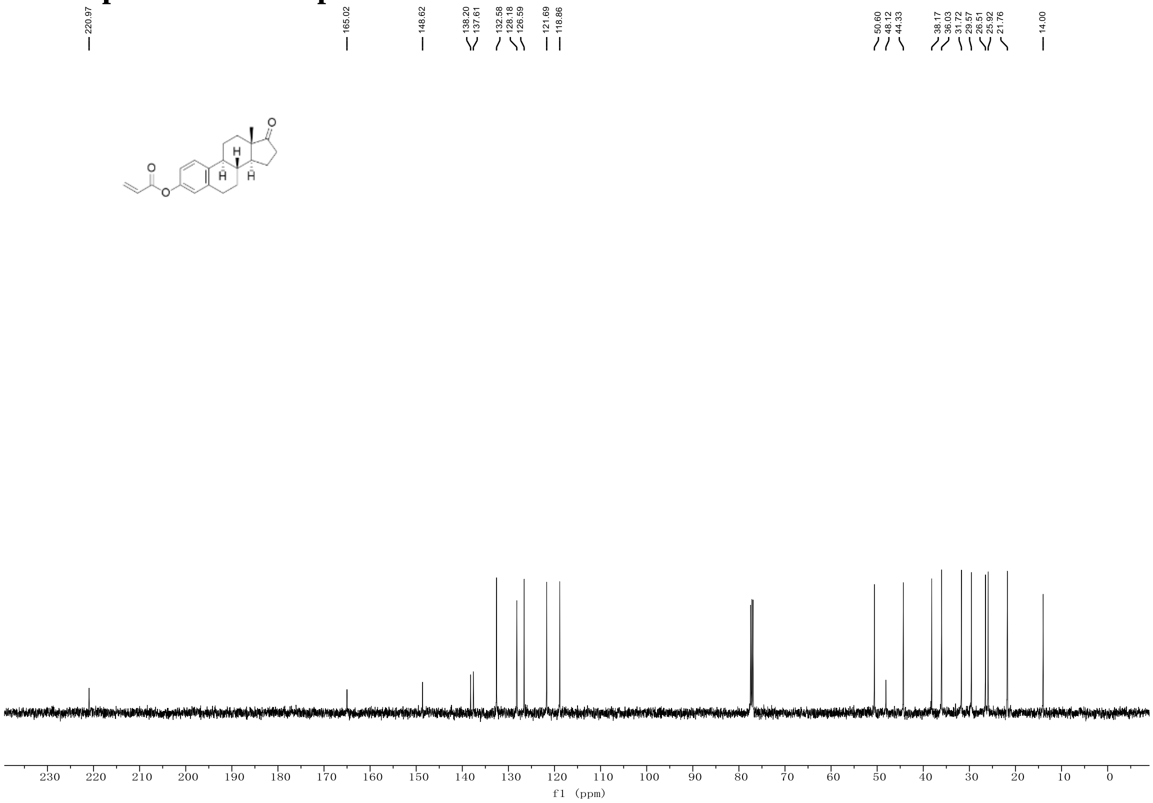

^{1}H NMR spectrum of compound S14

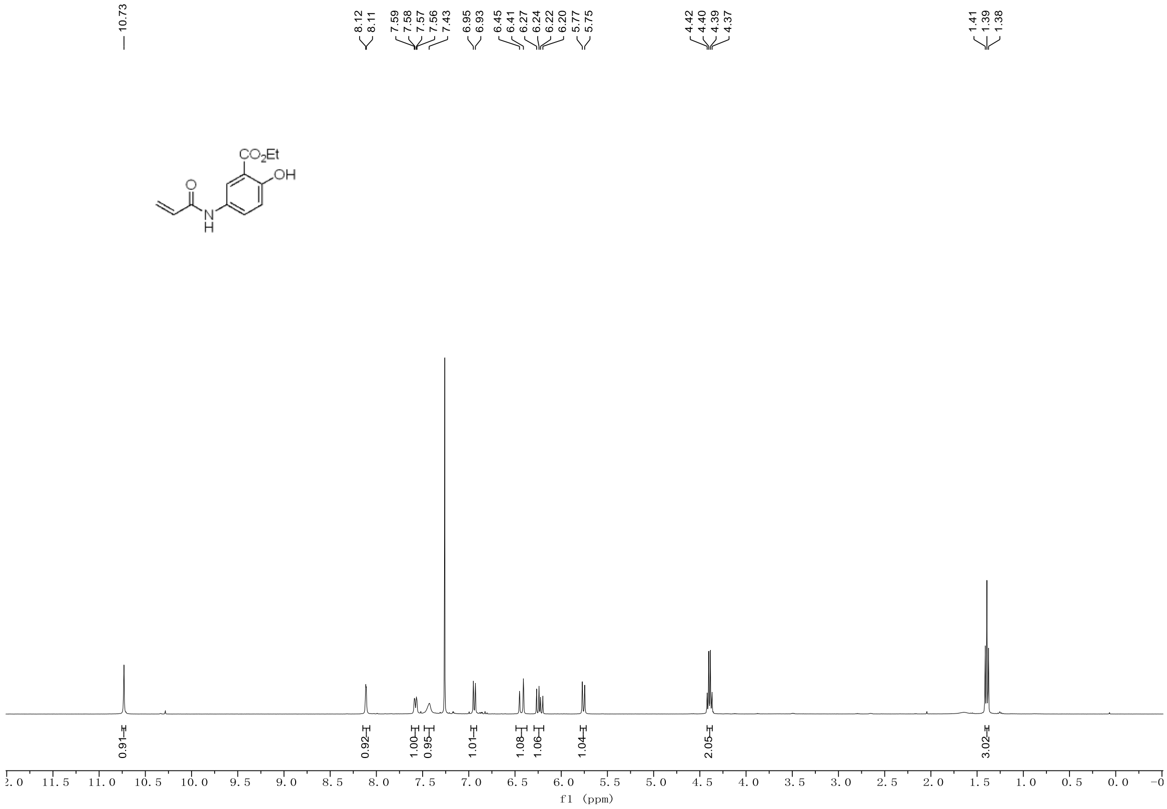

^{13}C NMR spectrum of compound S14

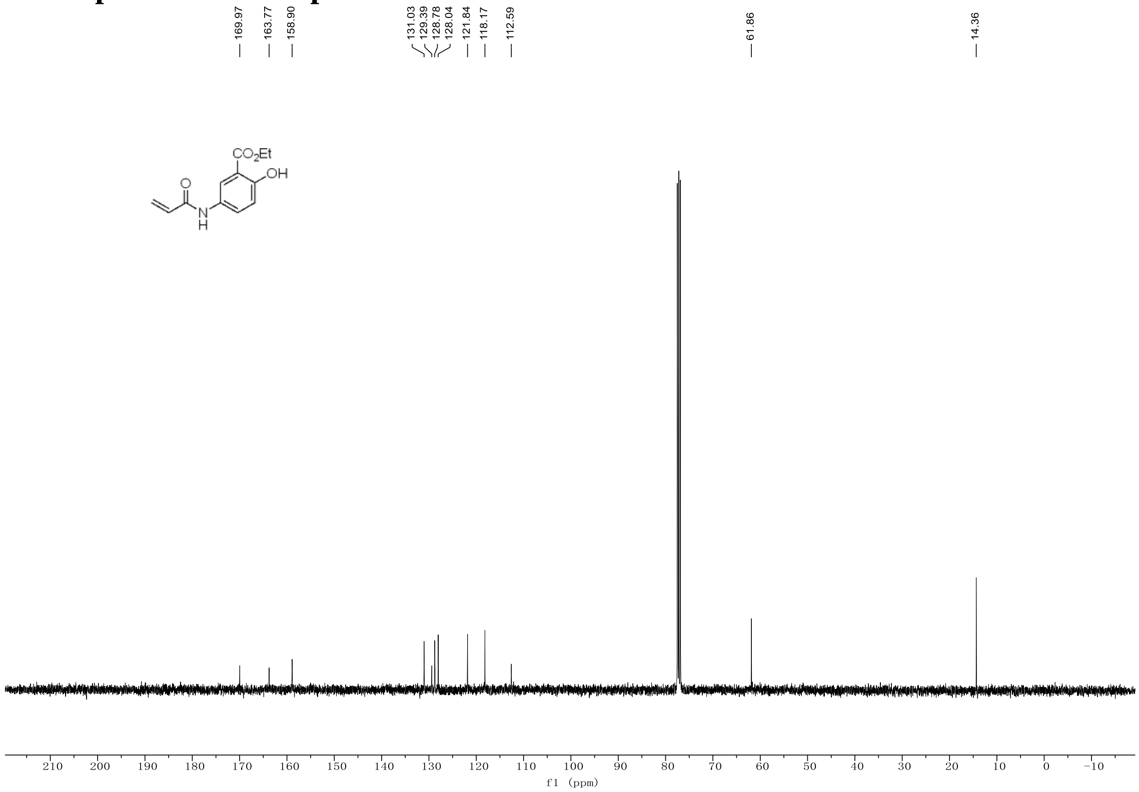

^{1}H NMR spectrum of compound S15

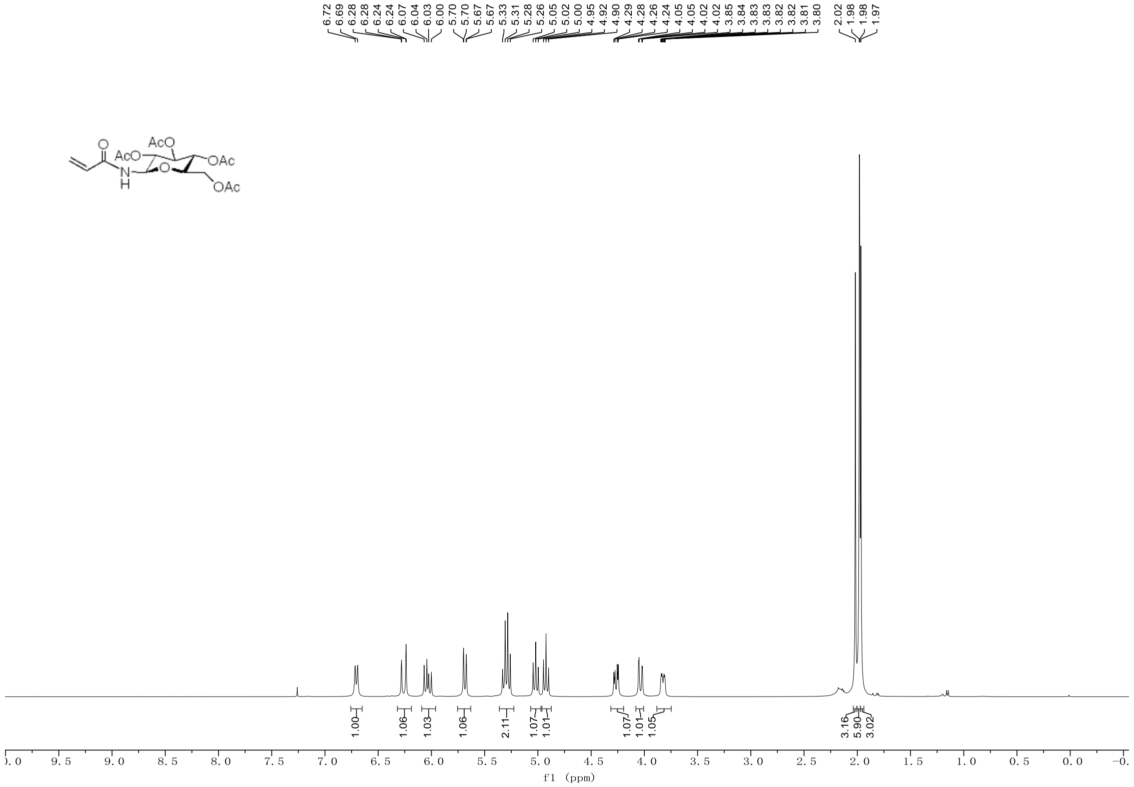

^{13}C NMR spectrum of compound S15

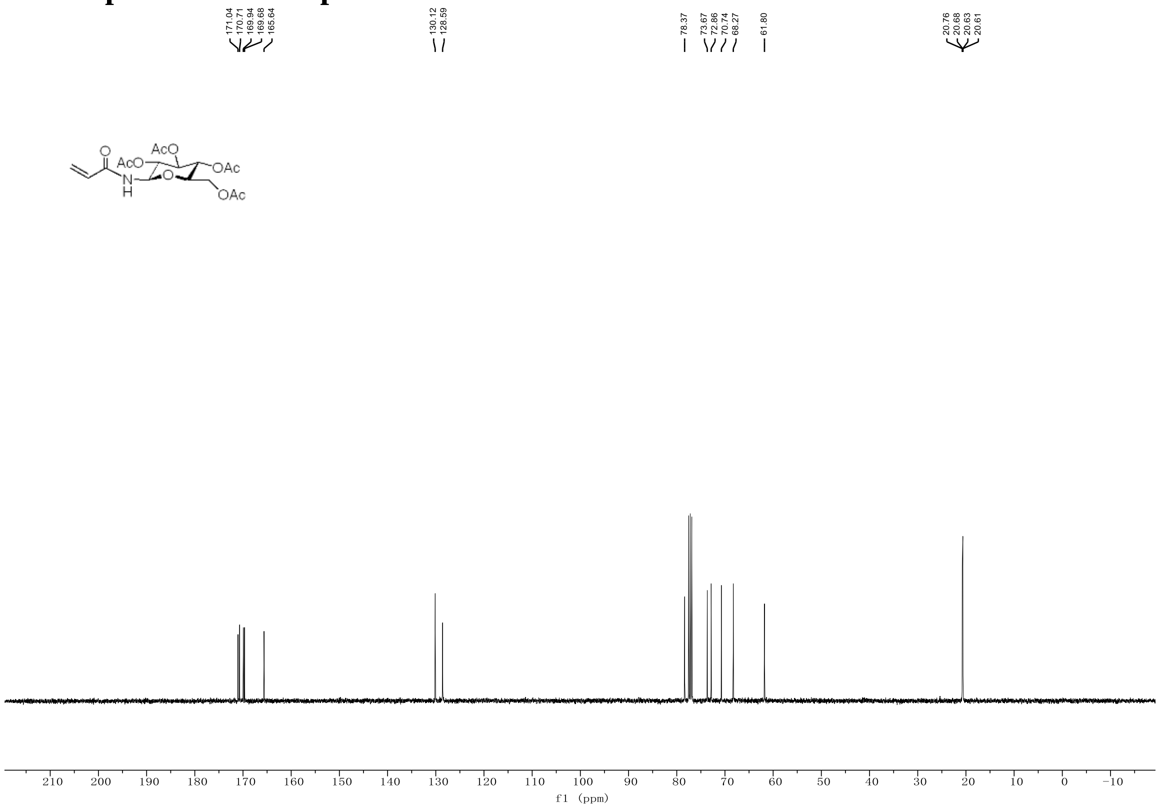

^{1}H NMR spectrum of compound S16

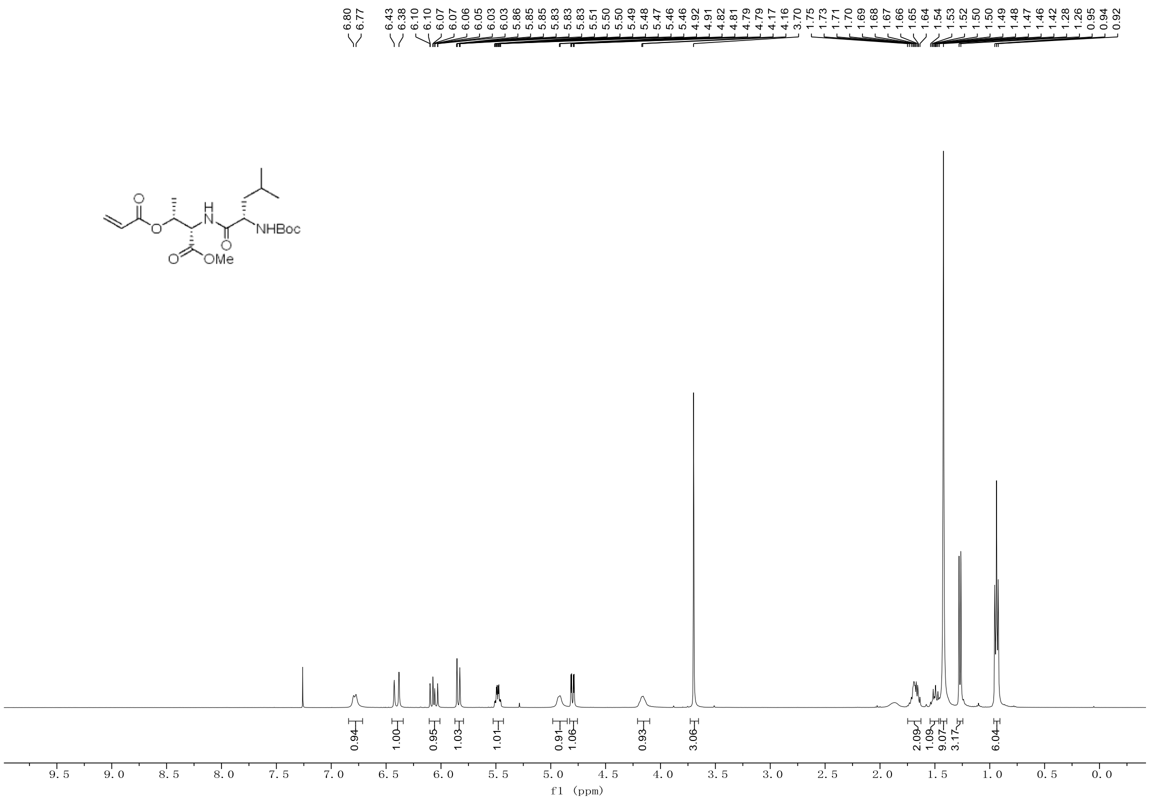

^{13}C NMR spectrum of compound S16

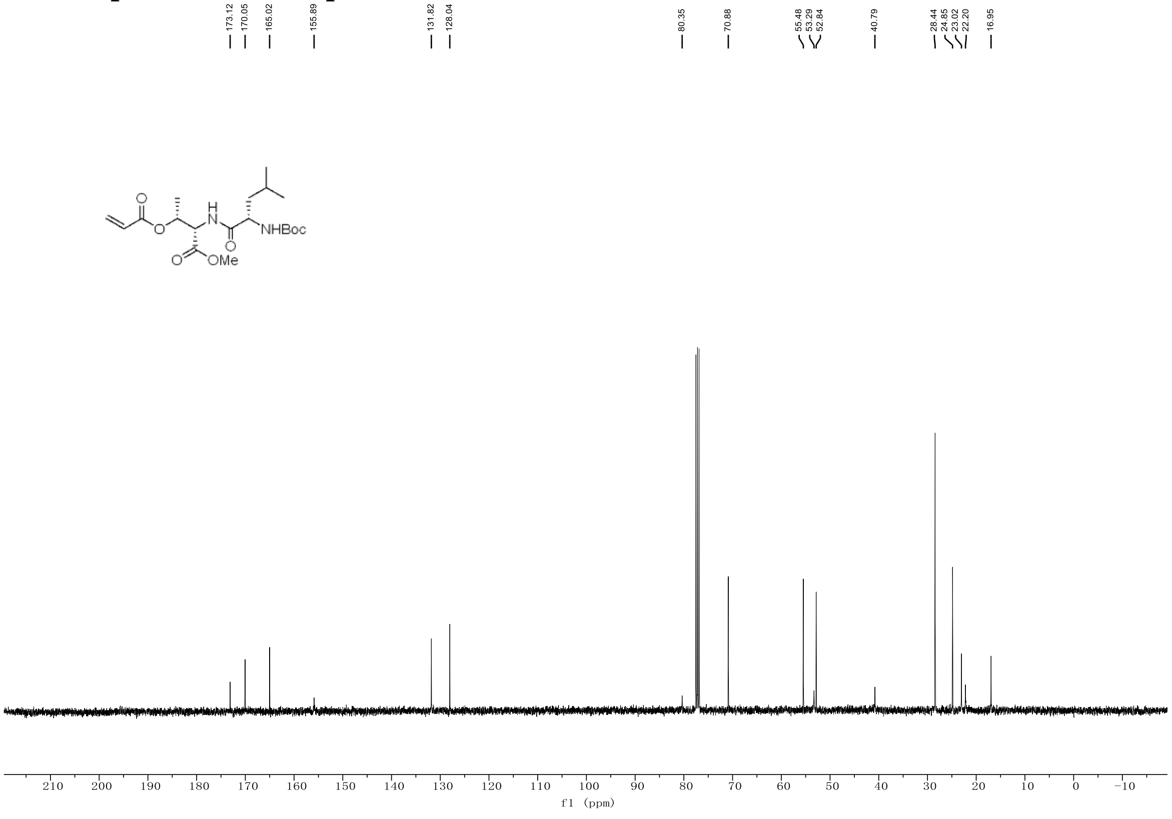

^{1}H NMR spectrum of compound S17

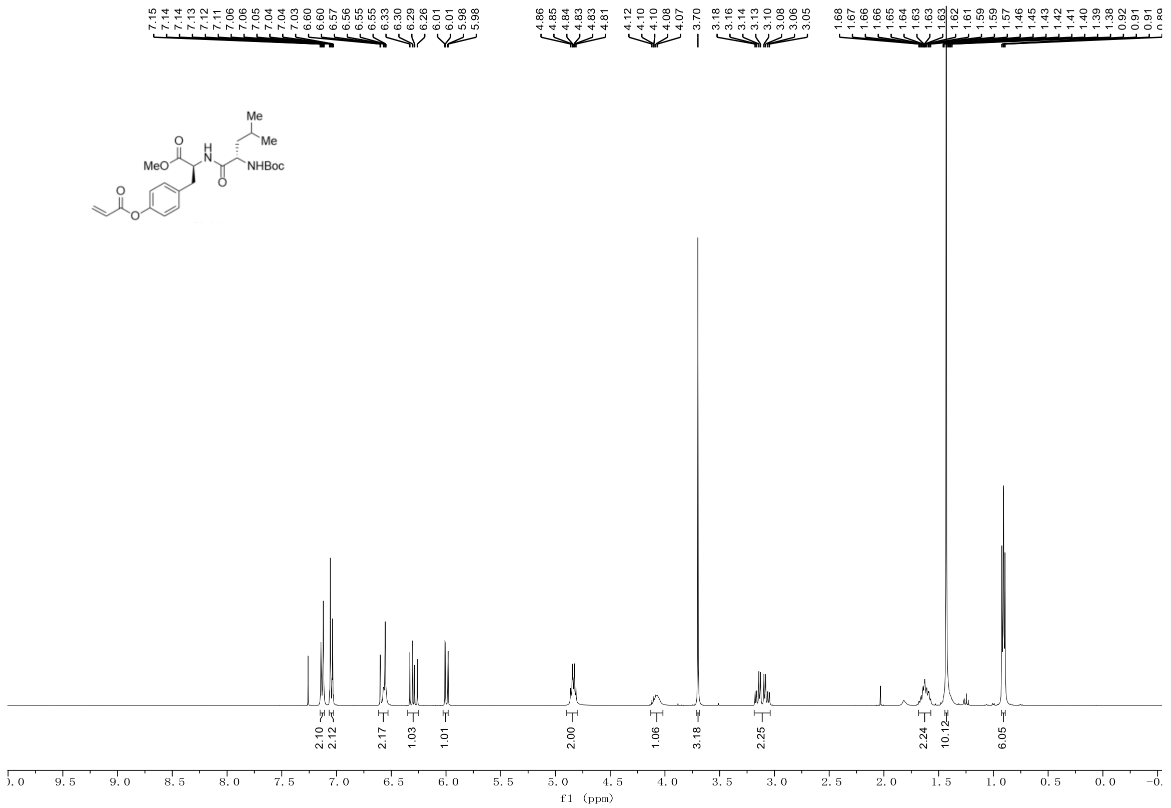

^{13}C NMR spectrum of compound S17

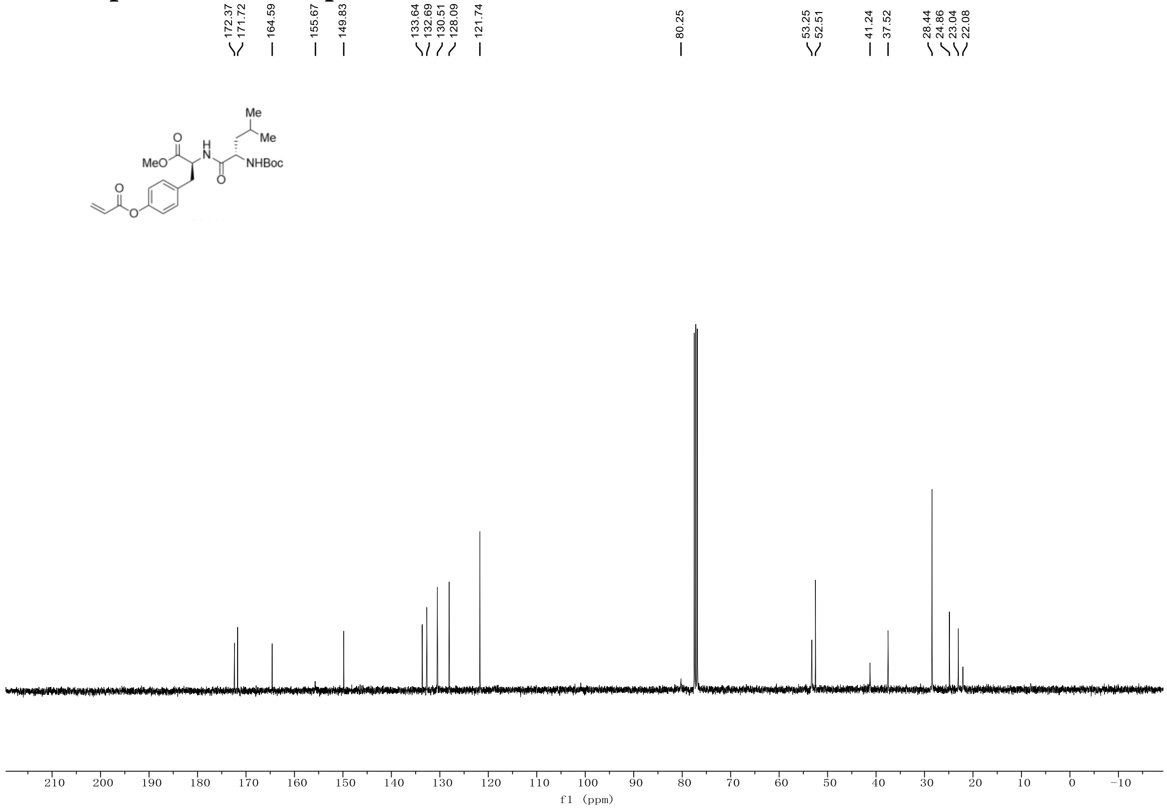

^{1}H NMR spectrum of compound S18

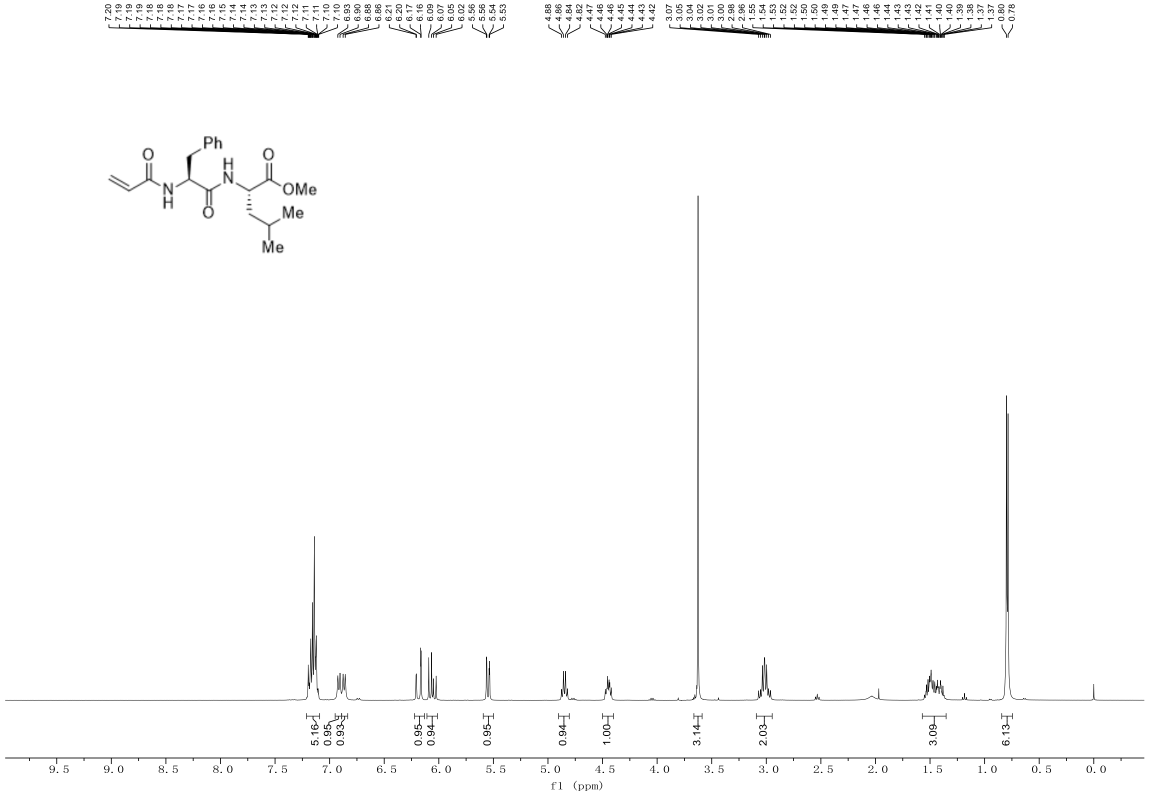

^{13}C NMR spectrum of compound S18

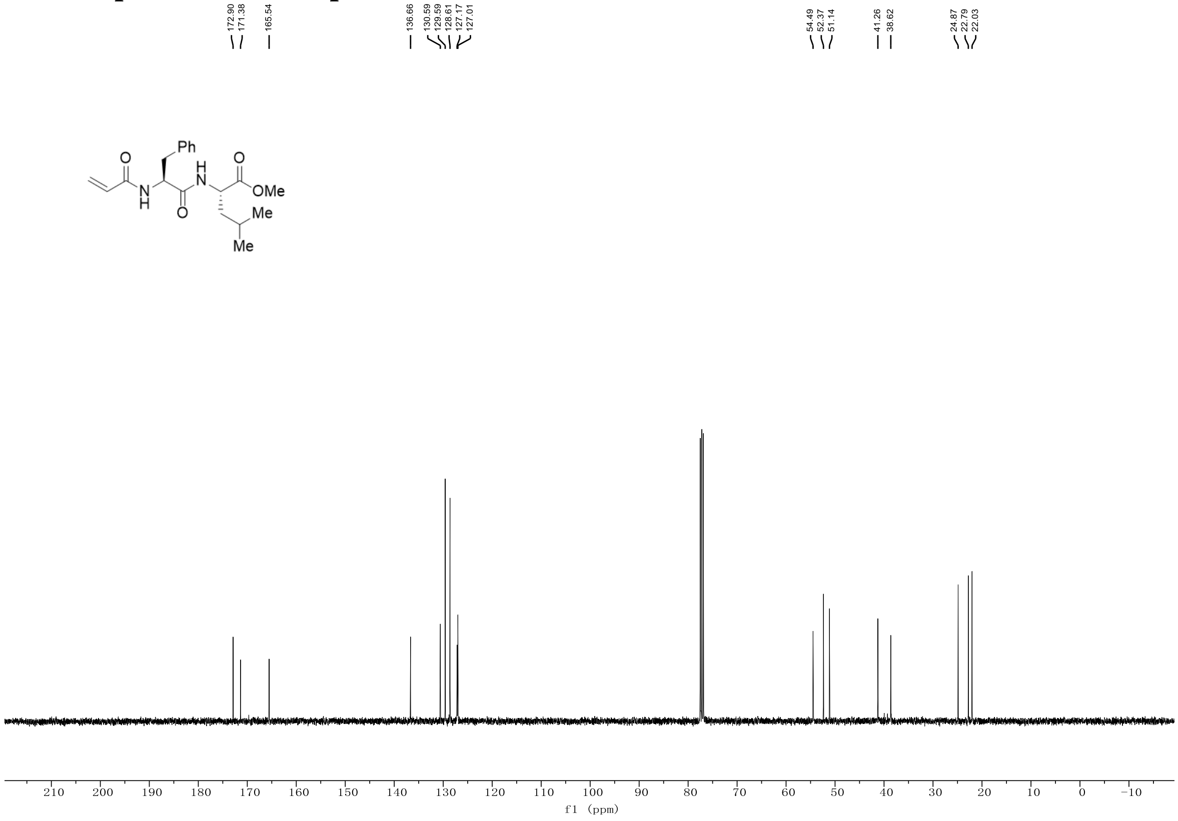

^{1}H NMR spectrum of compound S22

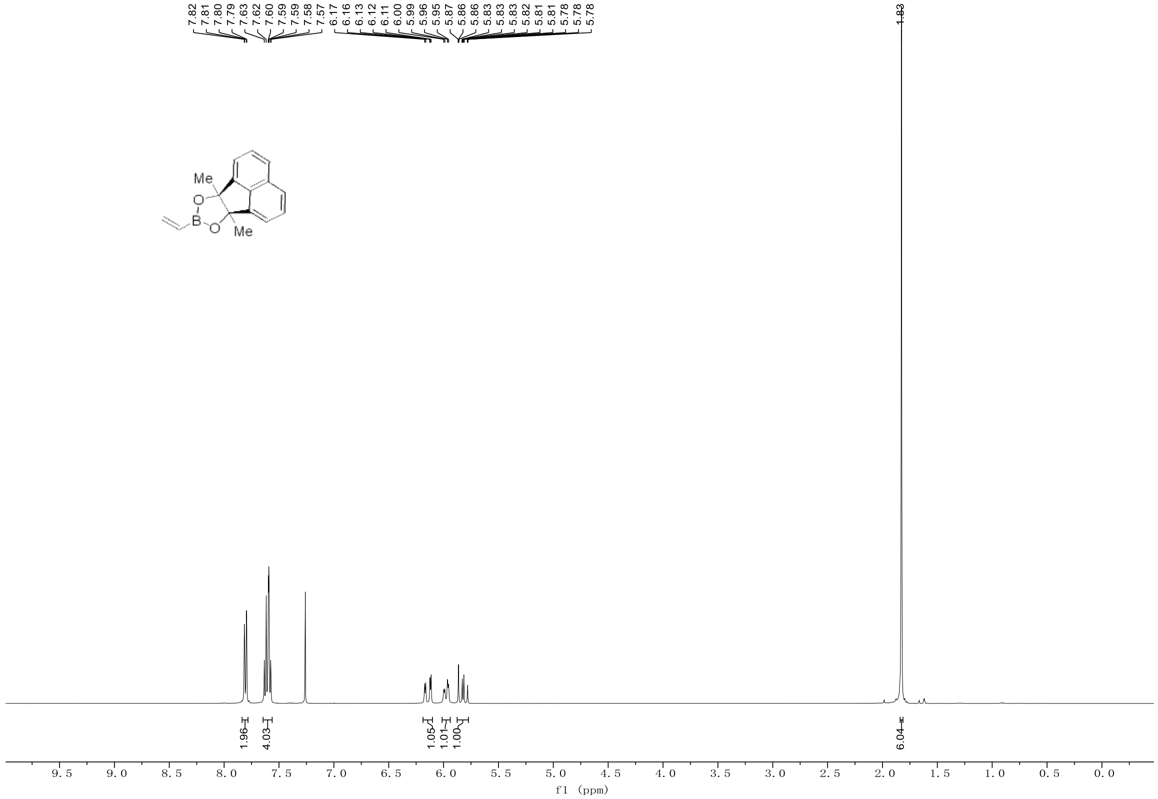

^{13}C NMR spectrum of compound S22

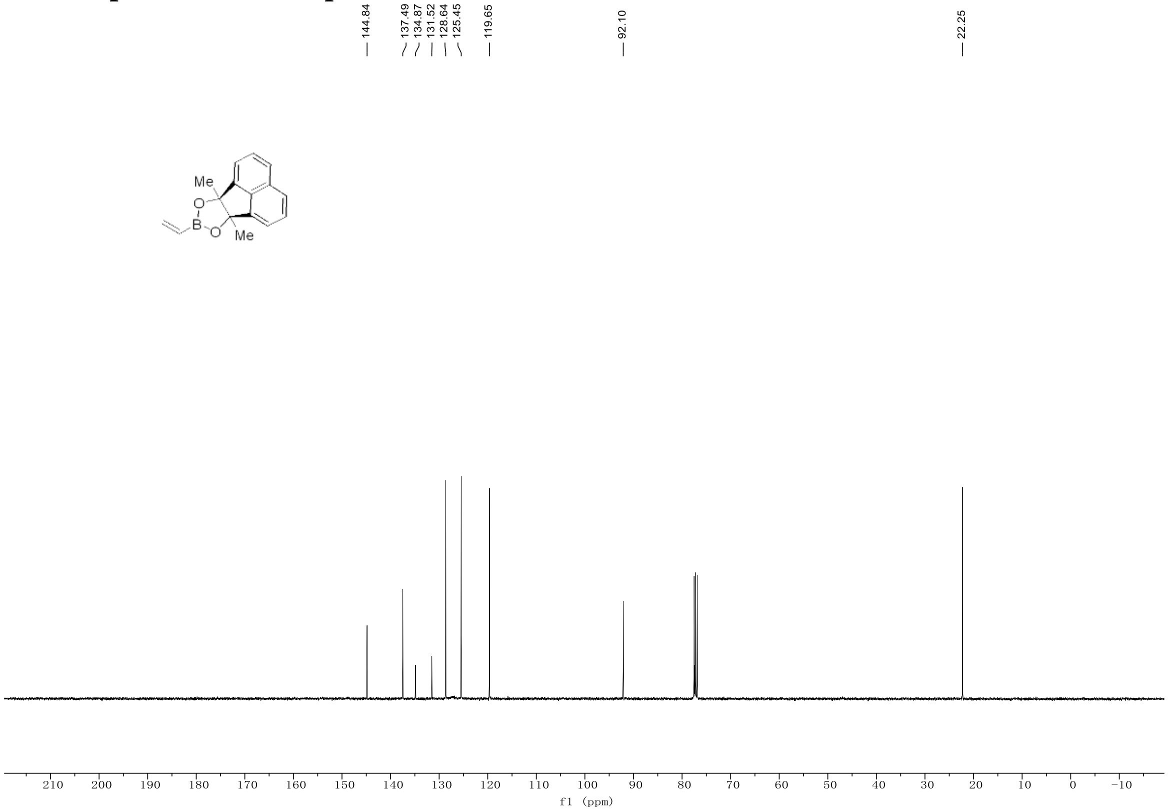

^{1}H NMR spectrum of compound S24

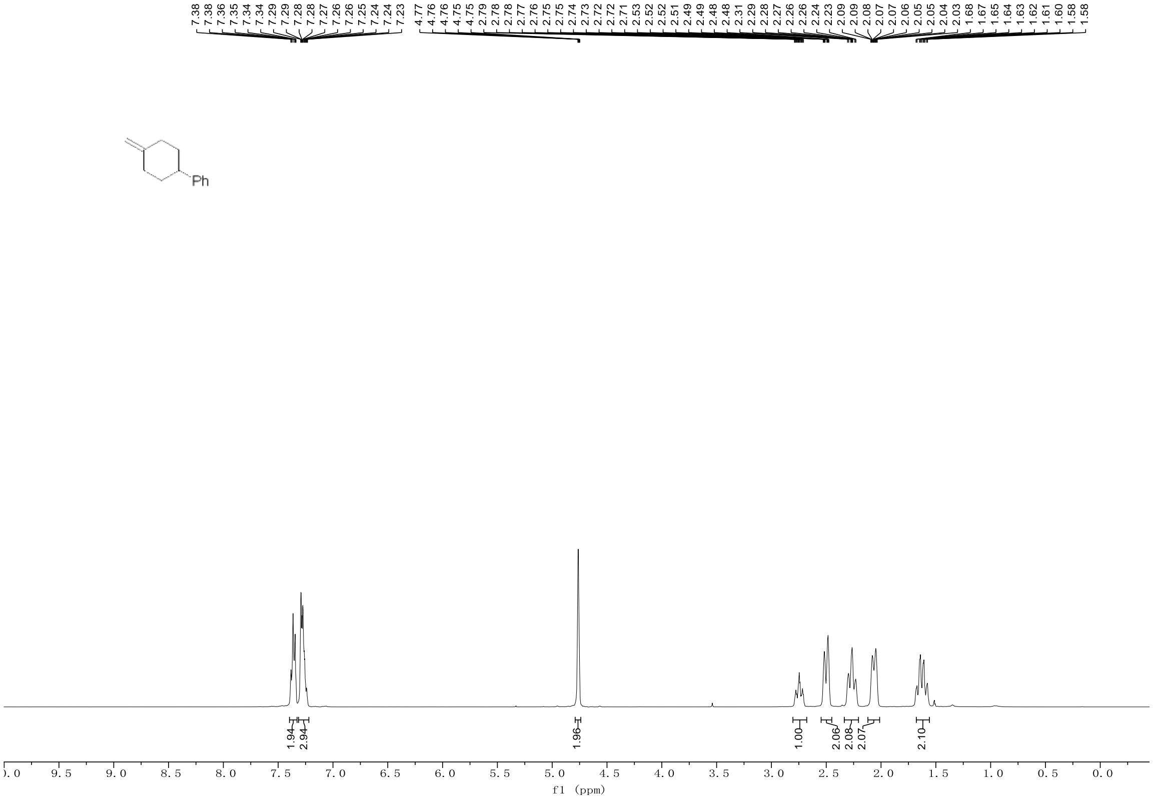

^{13}C NMR spectrum of compound S24

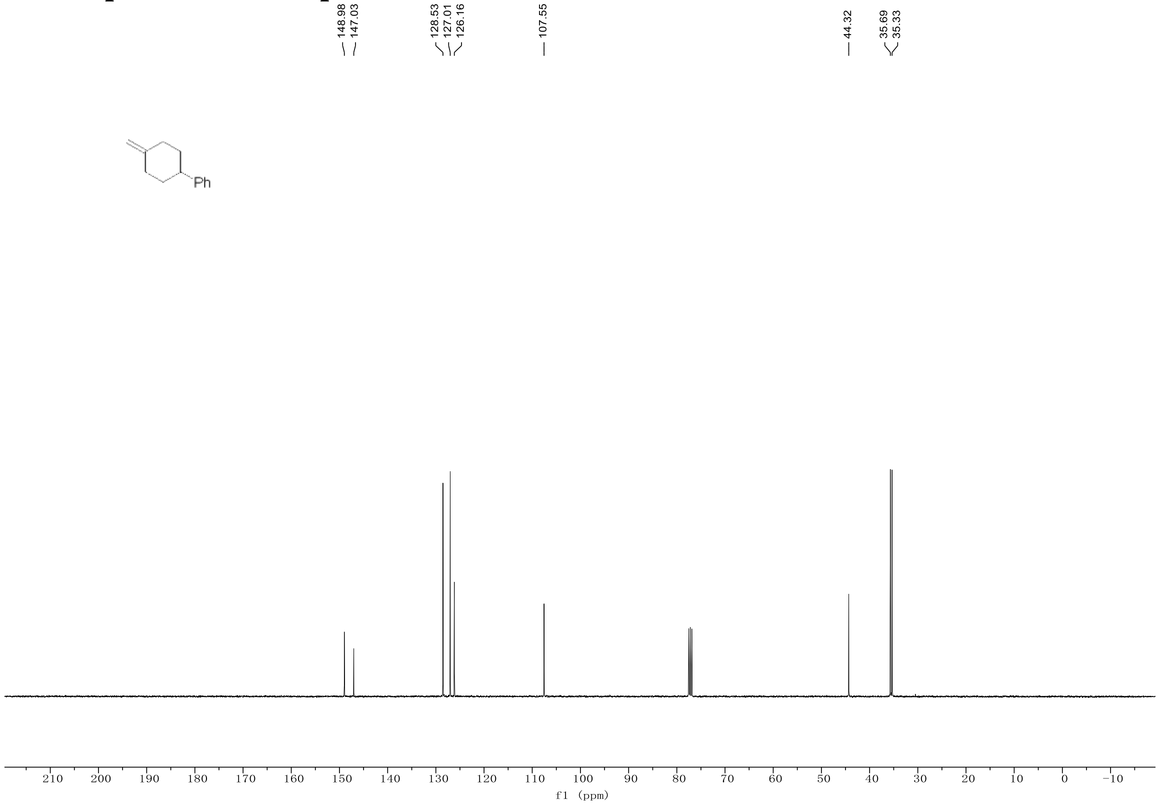

^{1}H NMR spectrum of compound S25

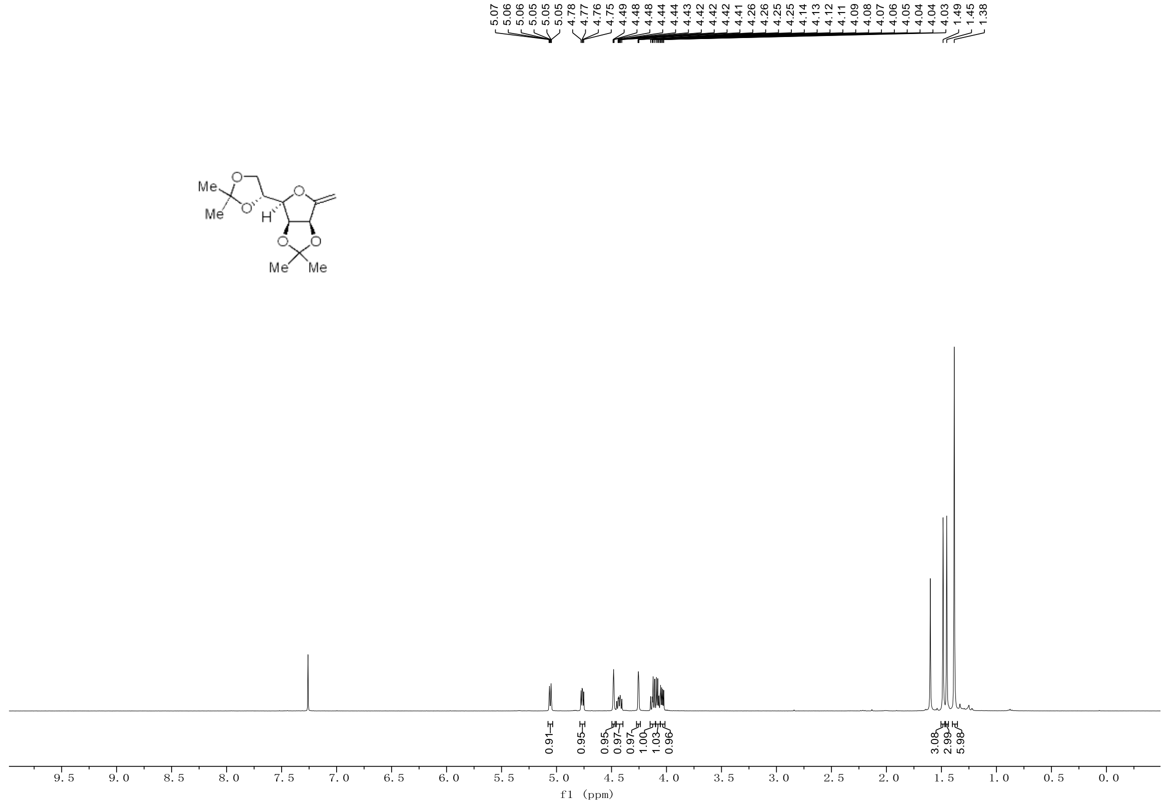

^{13}C NMR spectrum of compound S25

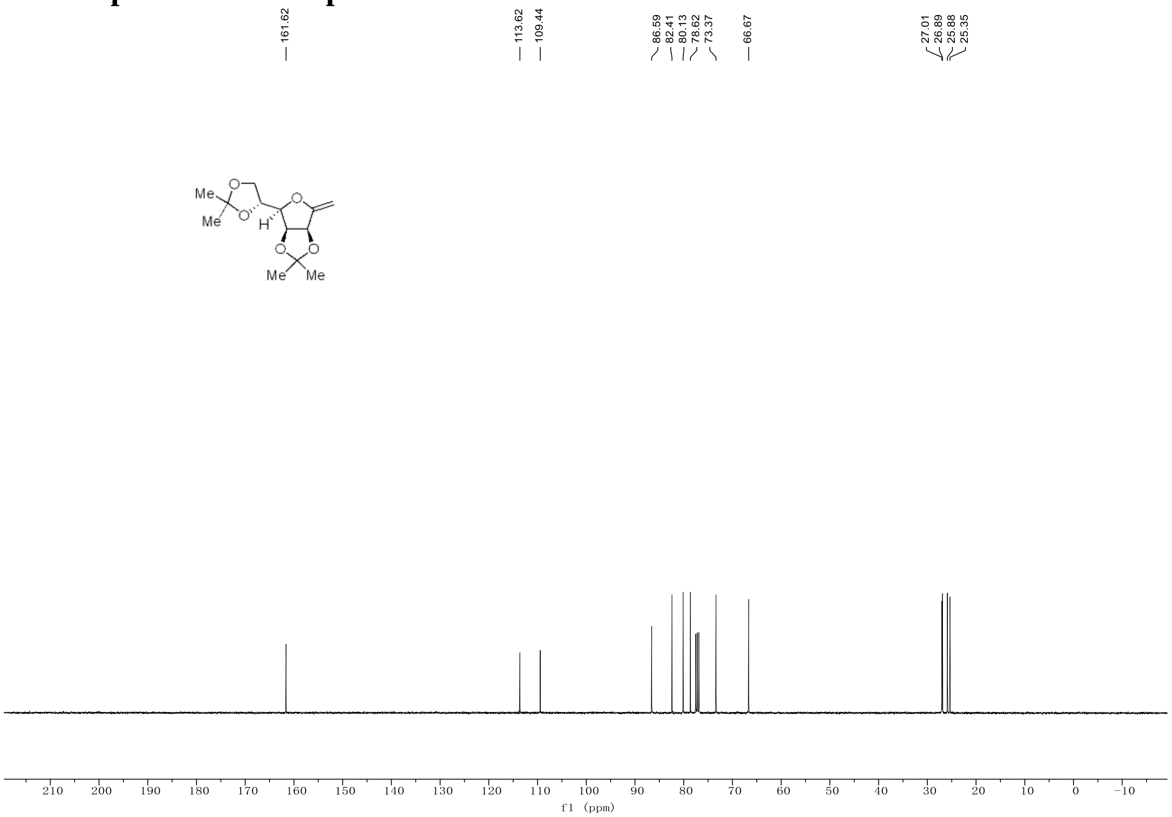

^{1}H NMR spectrum of compound S26

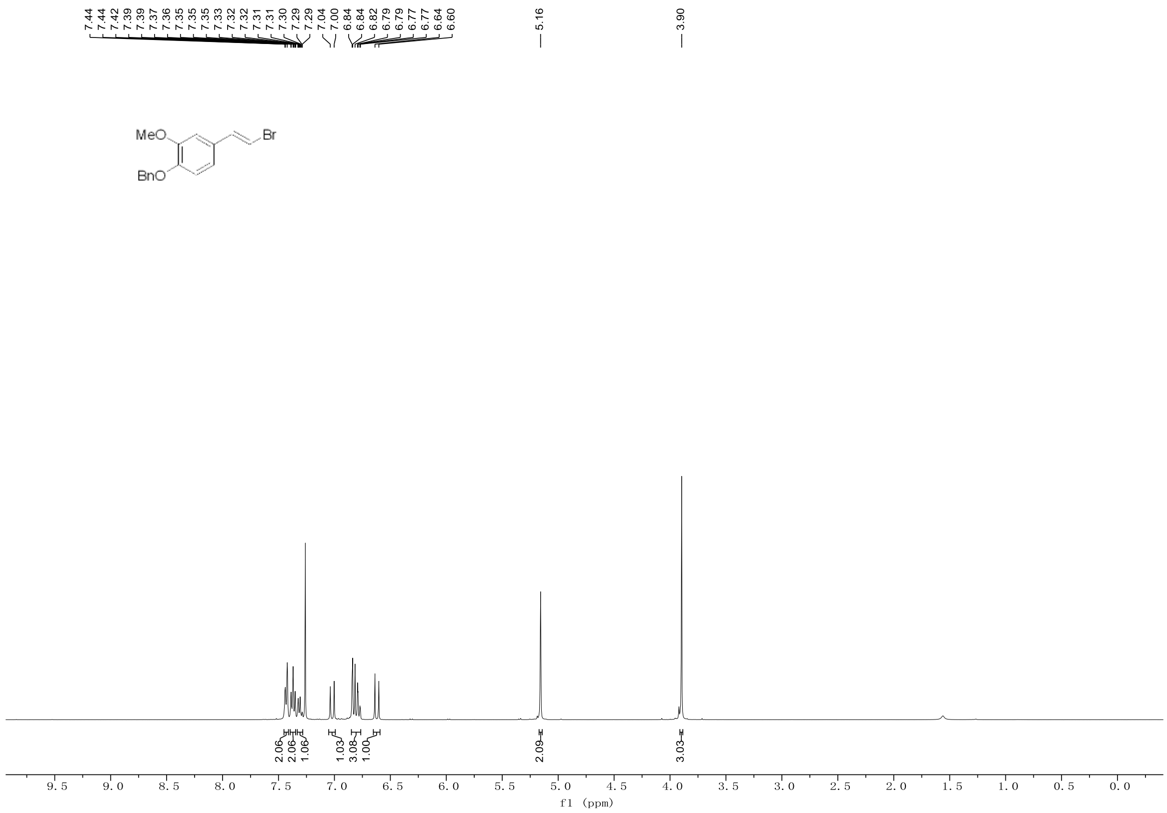

^{13}C NMR spectrum of compound S26

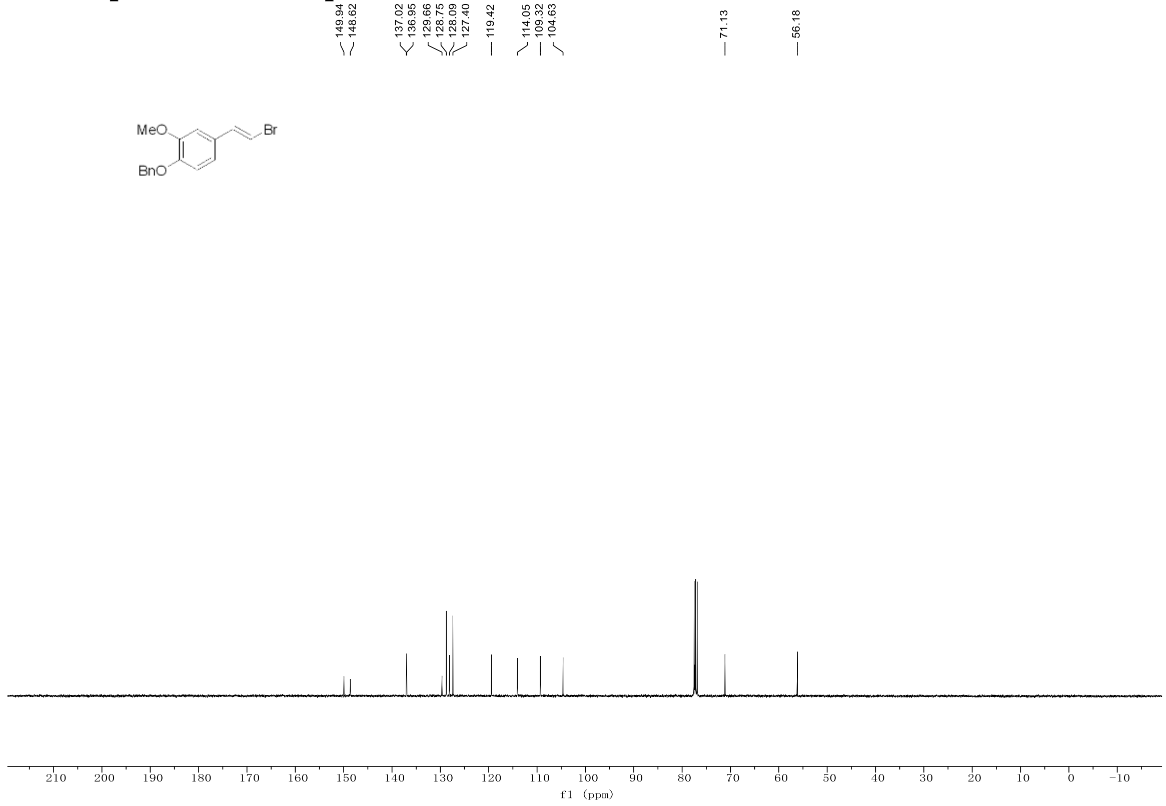

^{1}H NMR spectrum of compound S30

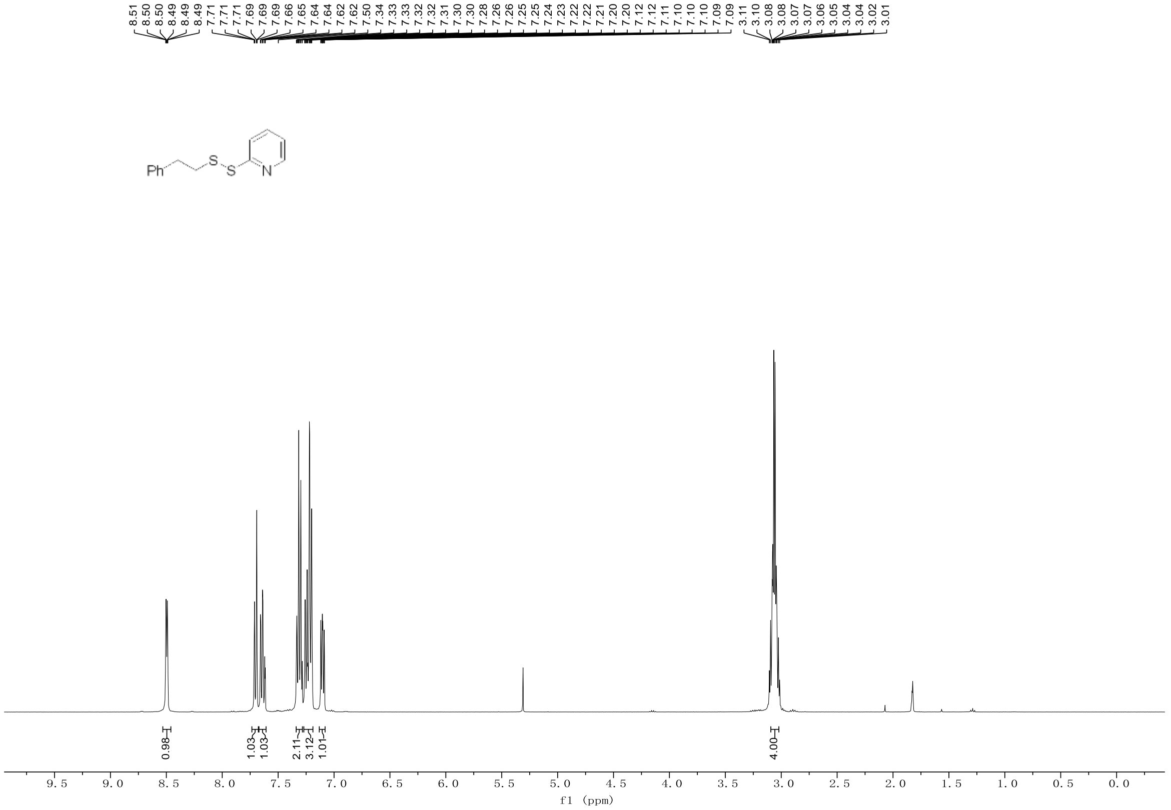

^{13}C NMR spectrum of compound S30

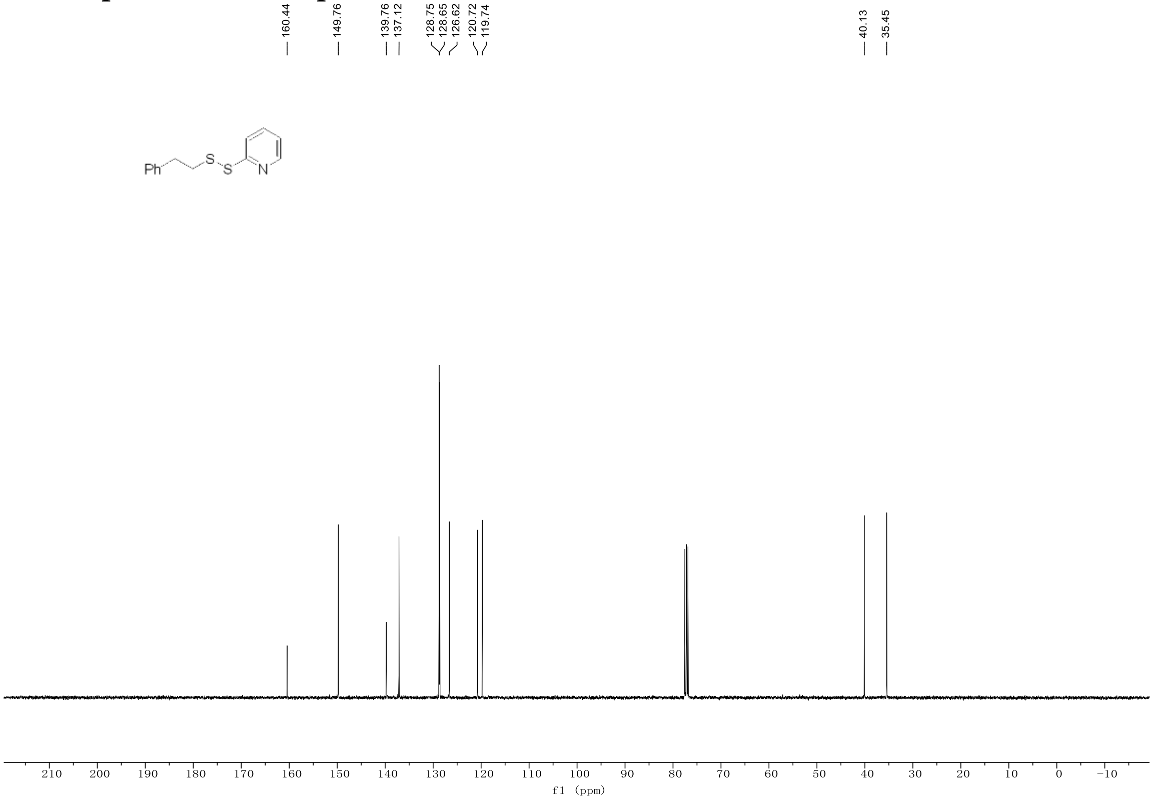

^{1}H NMR spectrum of compound S31

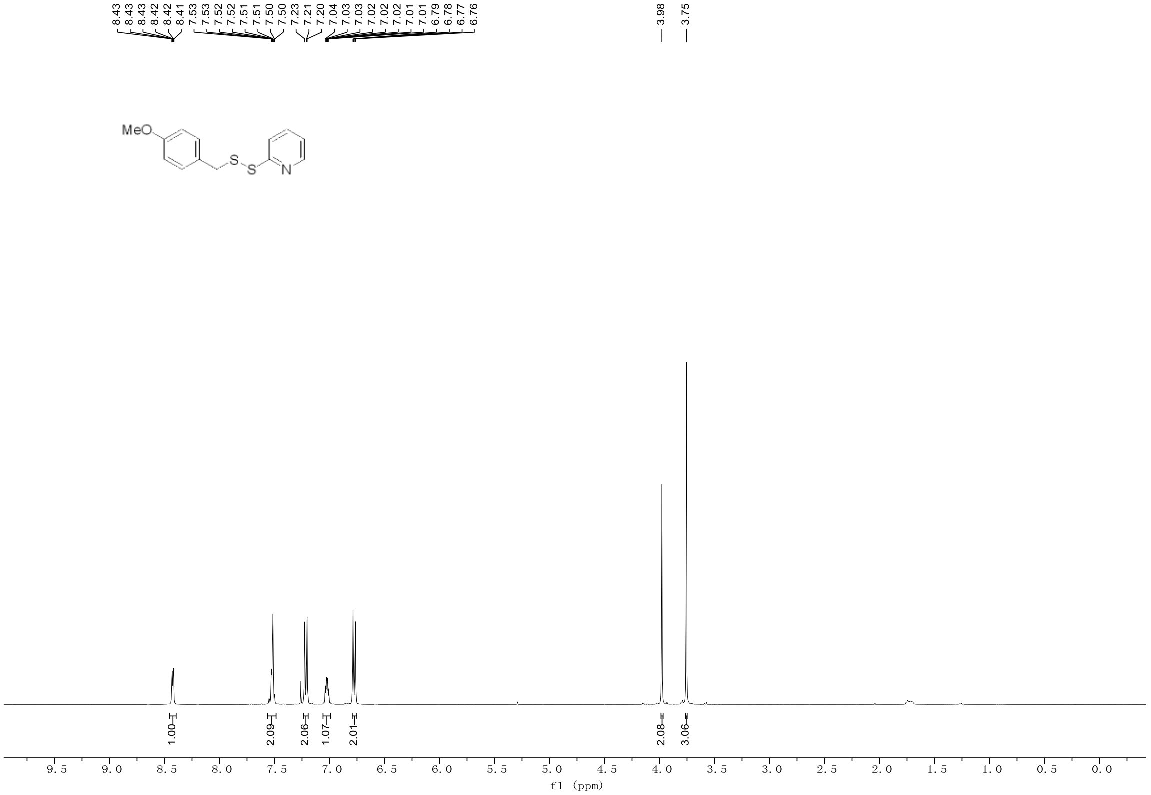

^{13}C NMR spectrum of compound S31

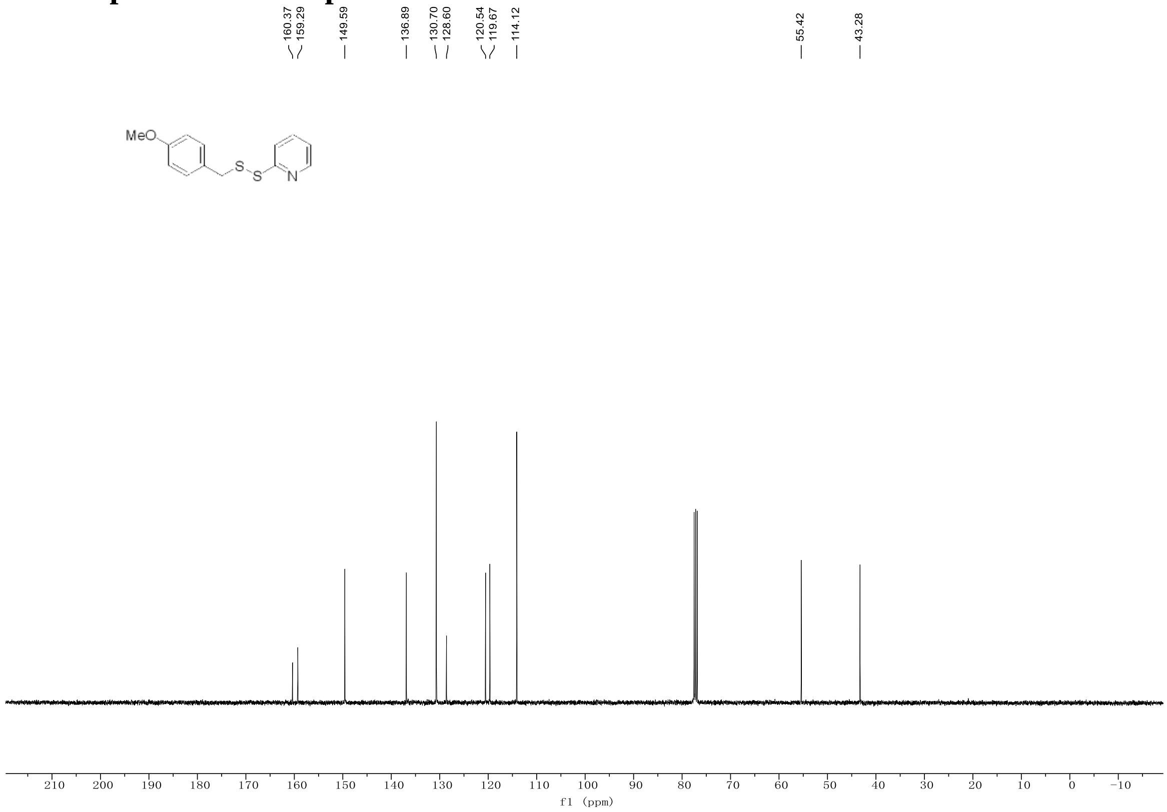

^{1}H NMR spectrum of compound S32

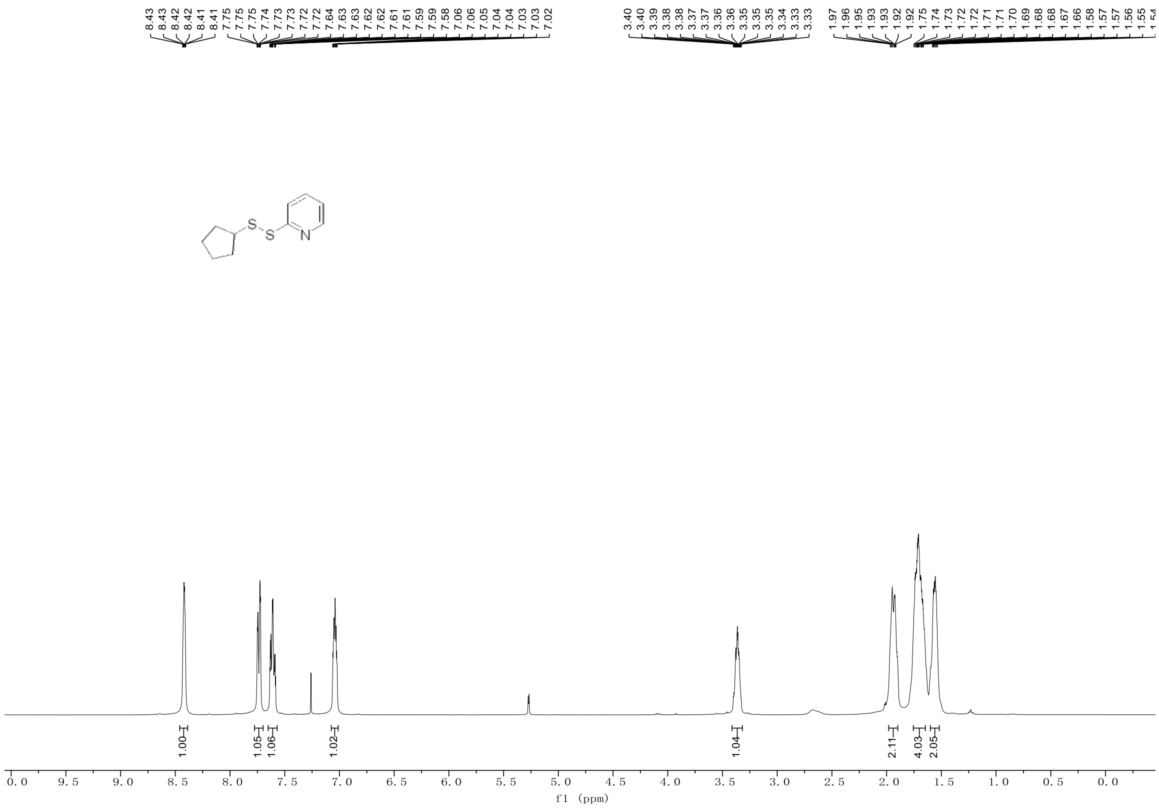

^{13}C NMR spectrum of compound S32

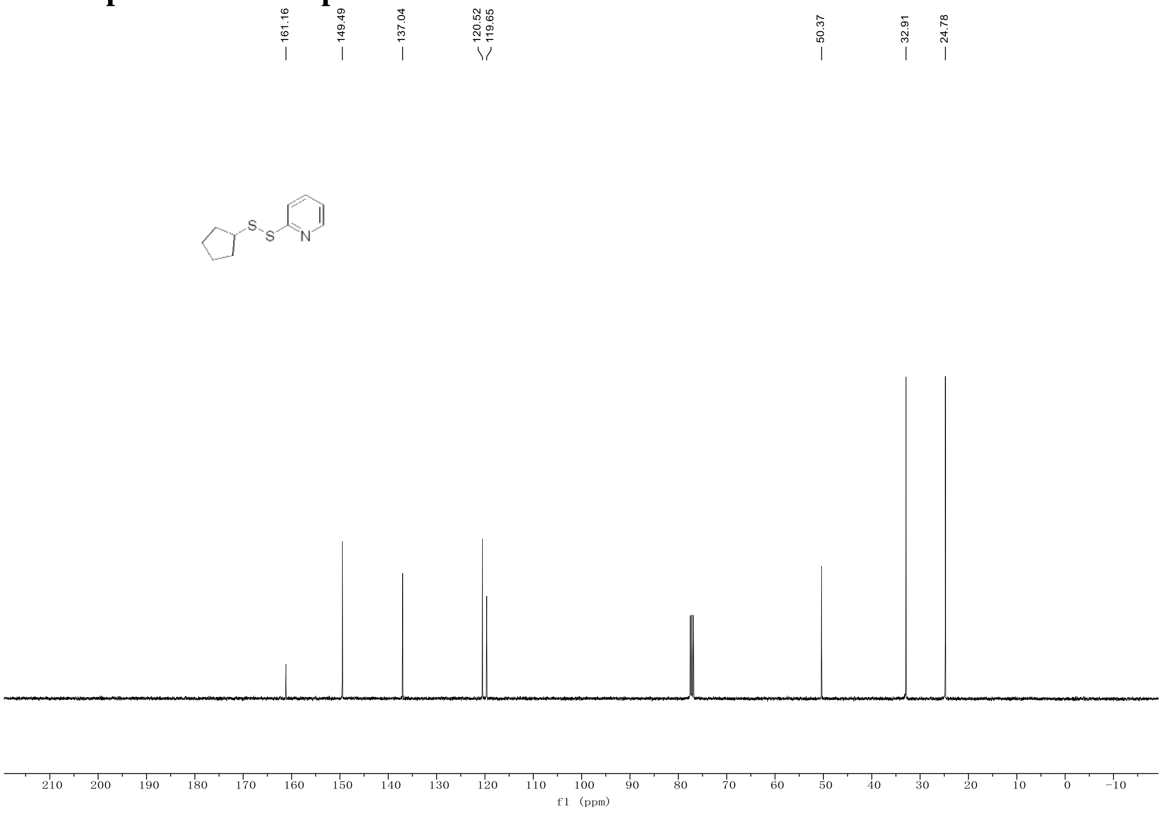

^{1}H NMR spectrum of compound S33

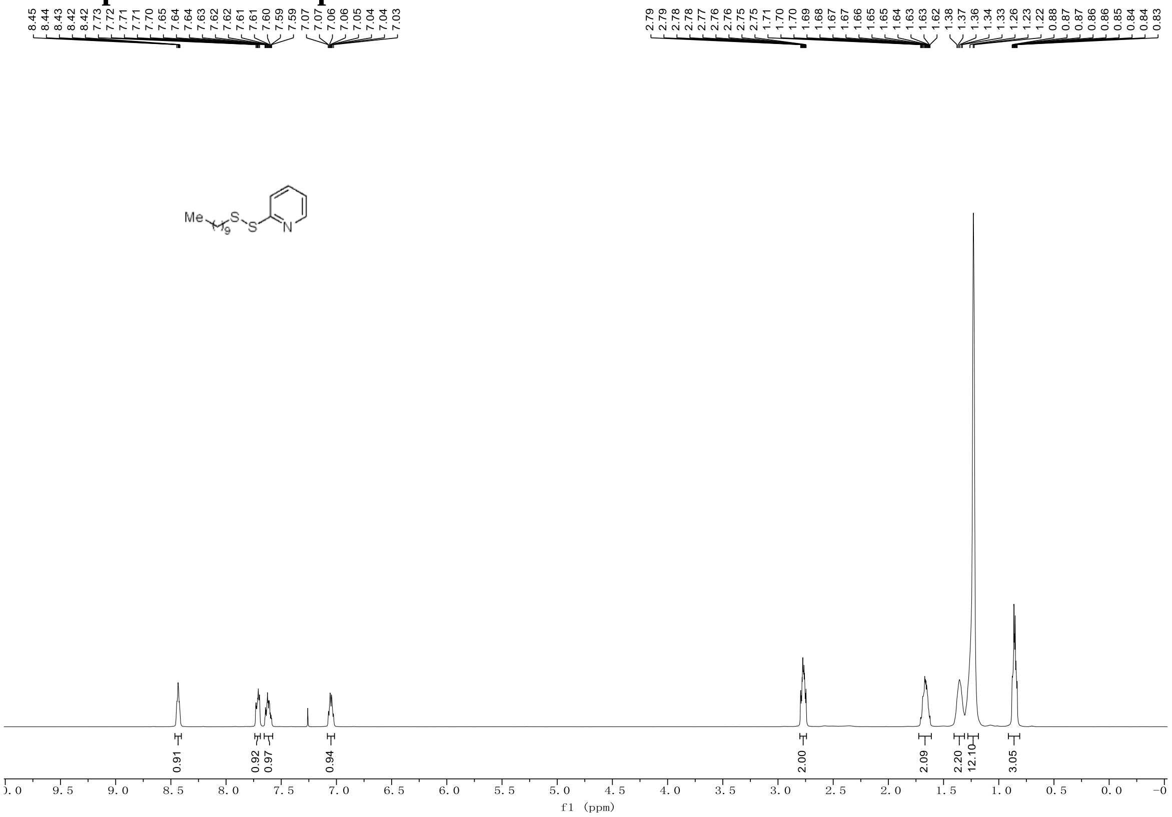

^{13}C NMR spectrum of compound S33

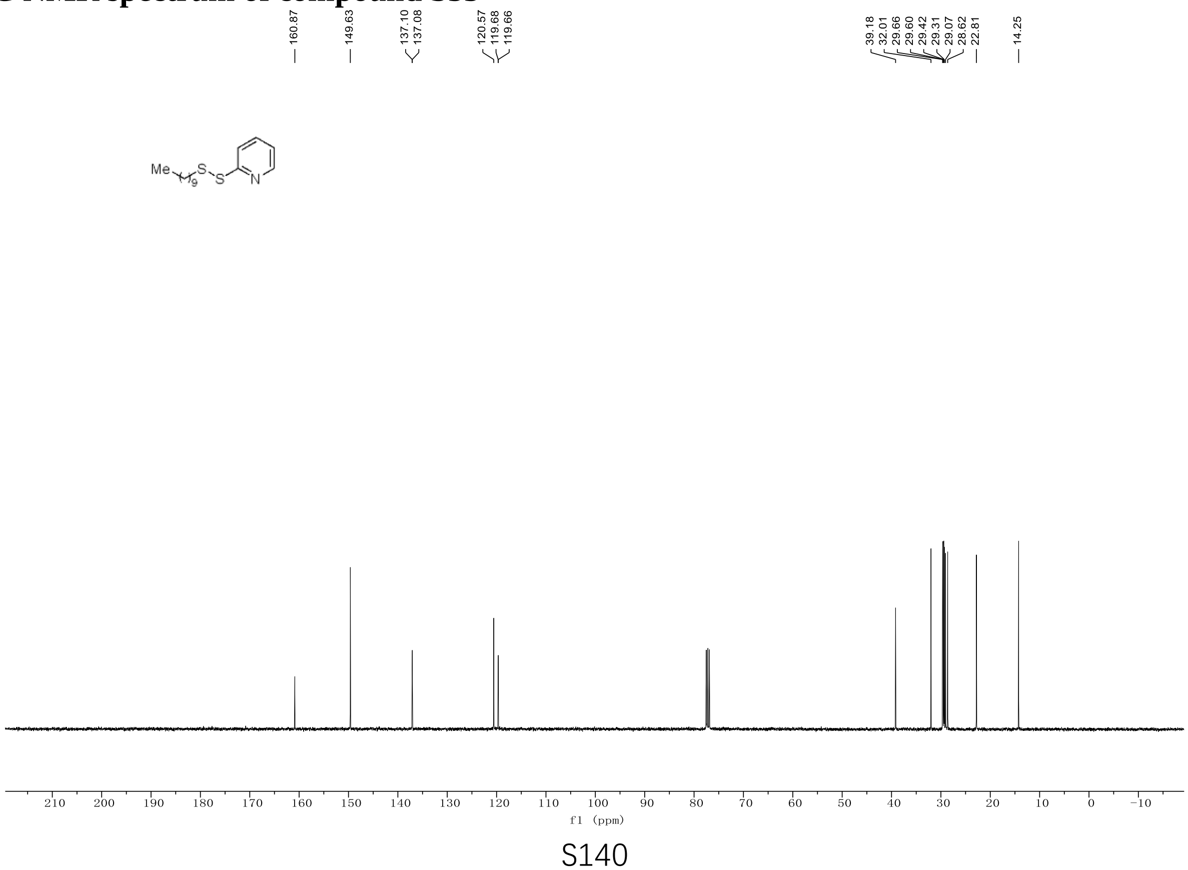

^{1}H NMR spectrum of compound S34

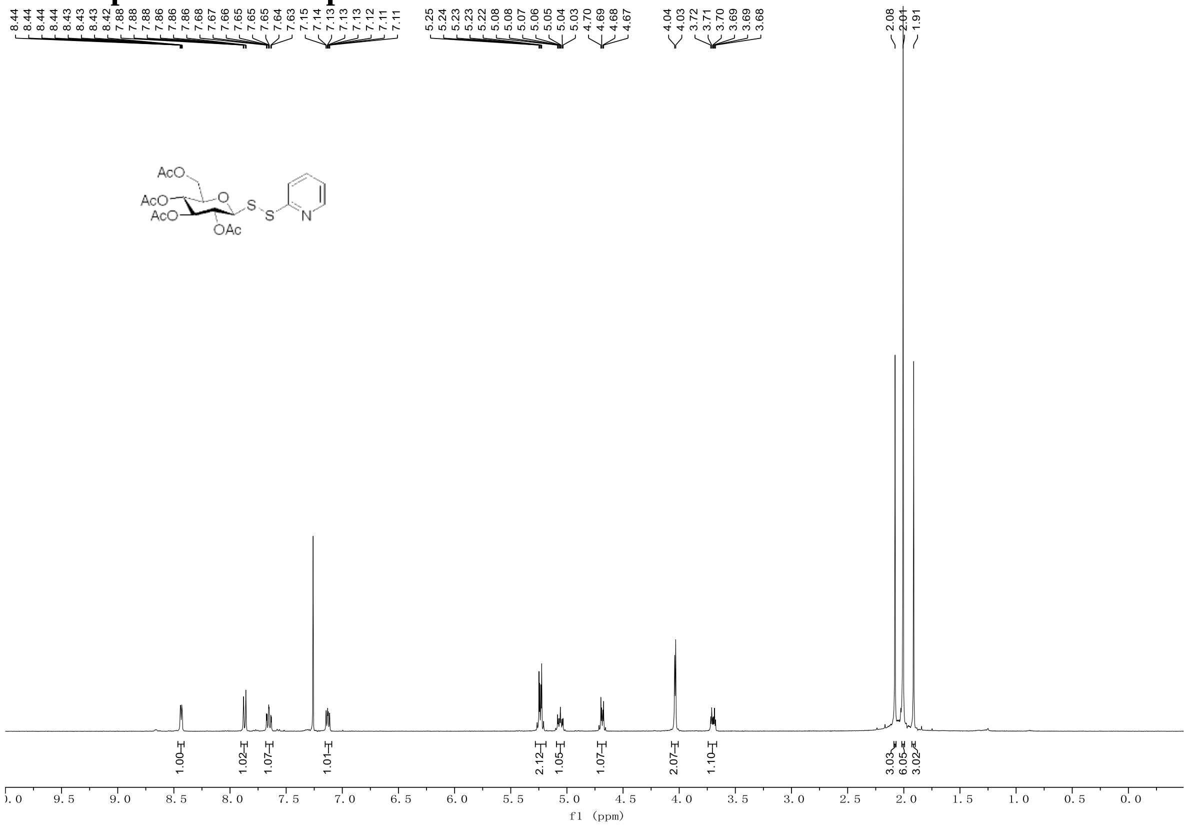

^{13}C NMR spectrum of compound S34

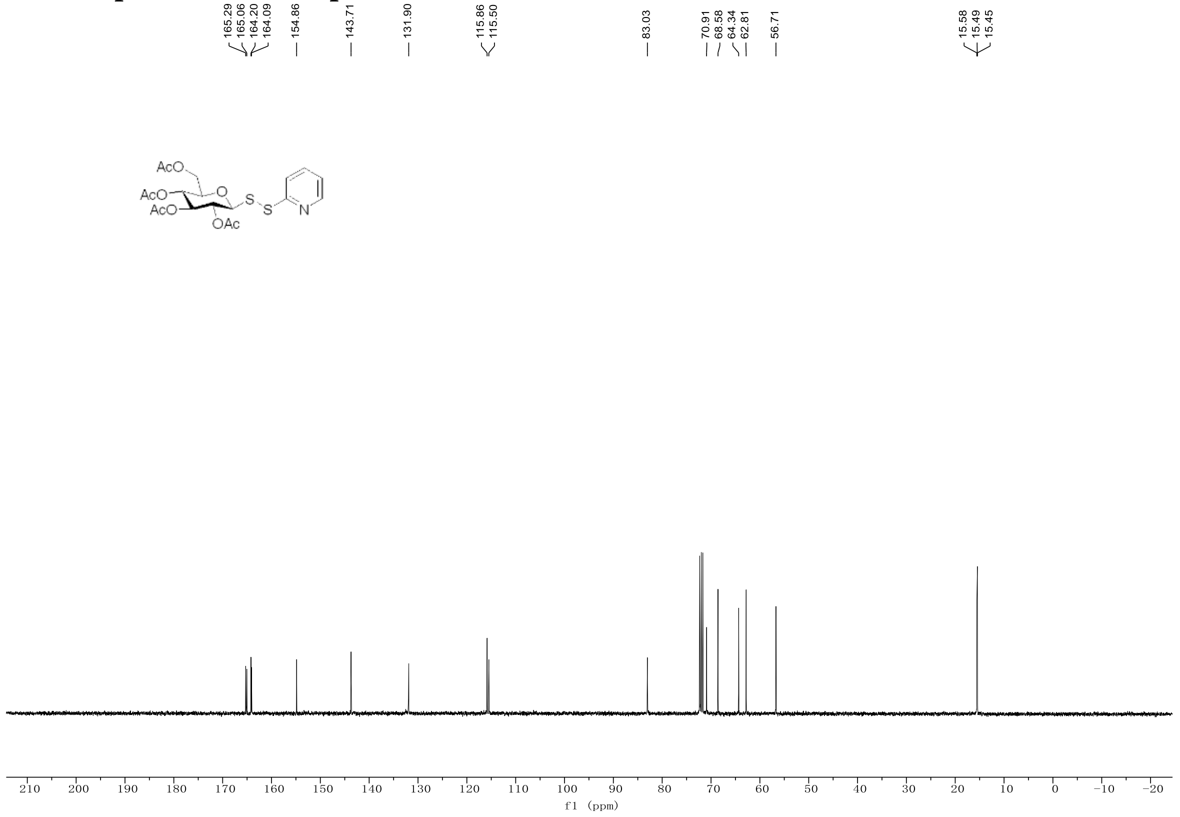

^{1}H NMR spectrum of compound 11

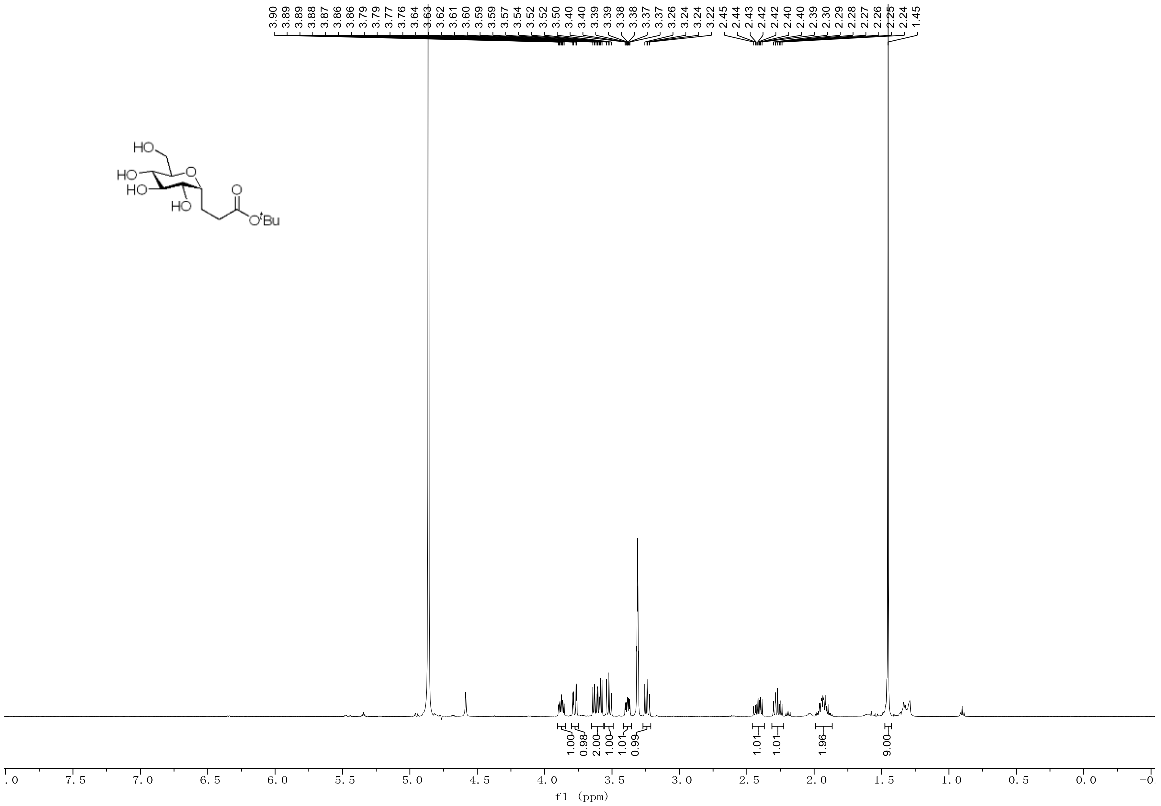

^{13}C NMR spectrum of compound 11

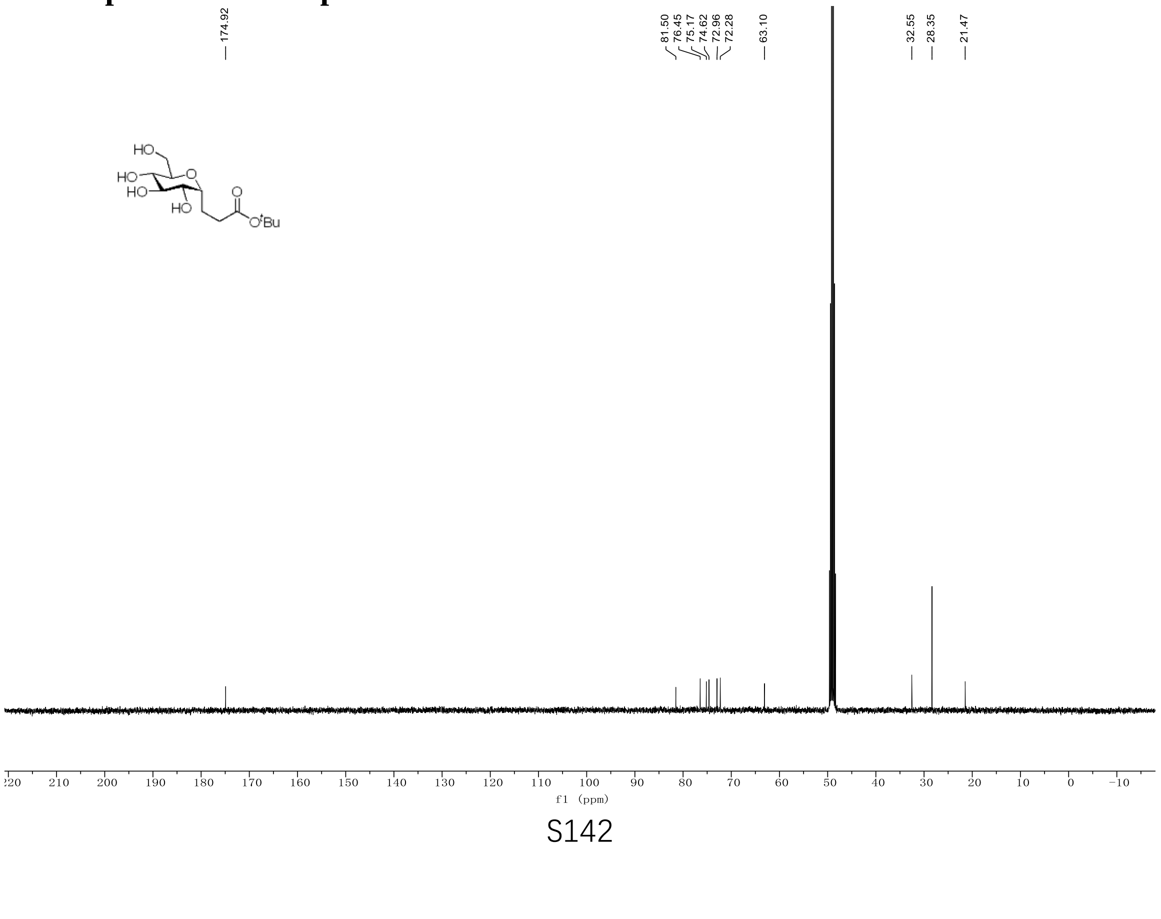

^{1}H NMR spectrum of compound 19

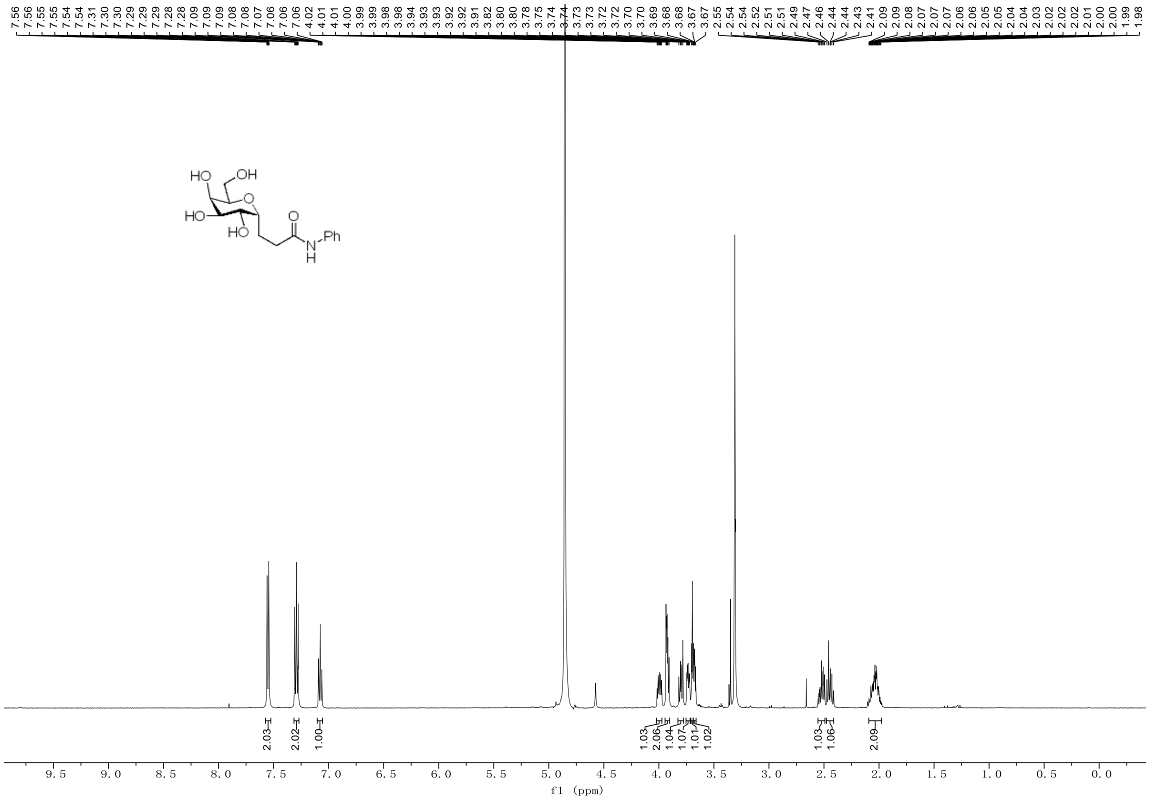

^{13}C NMR spectrum of compound 19

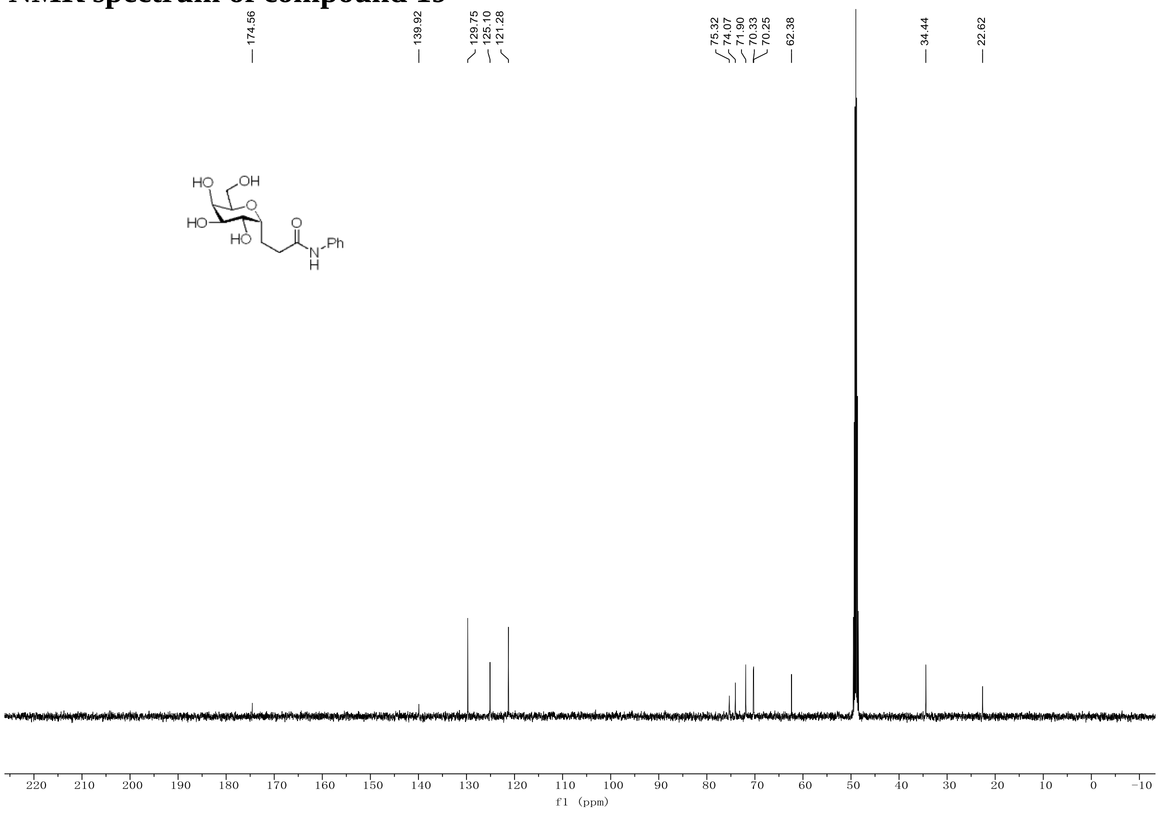

COSY spectrum of compound 19

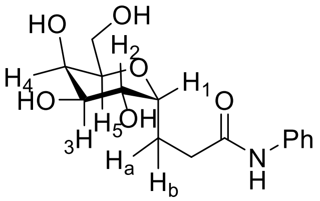

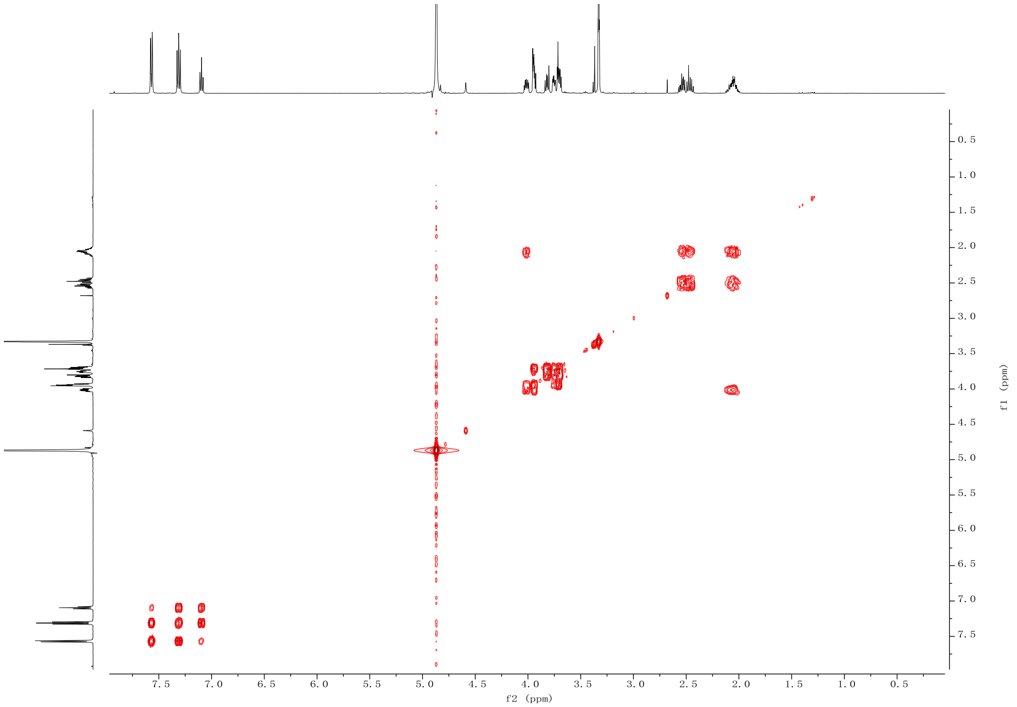

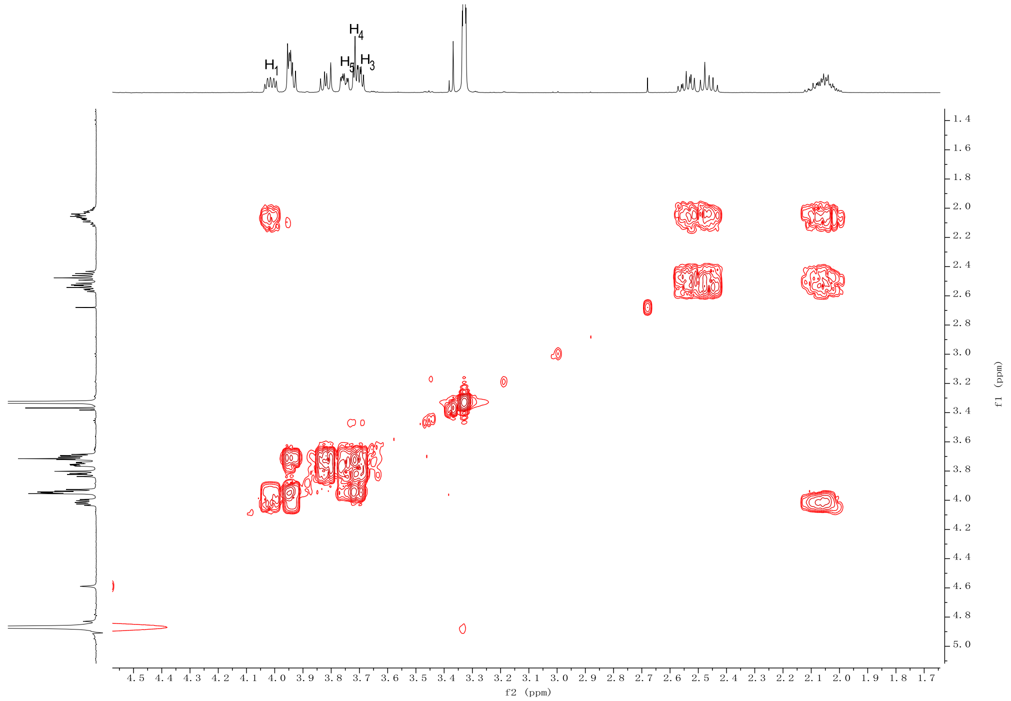

NOE spectrum of compound 19

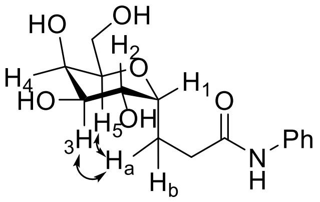

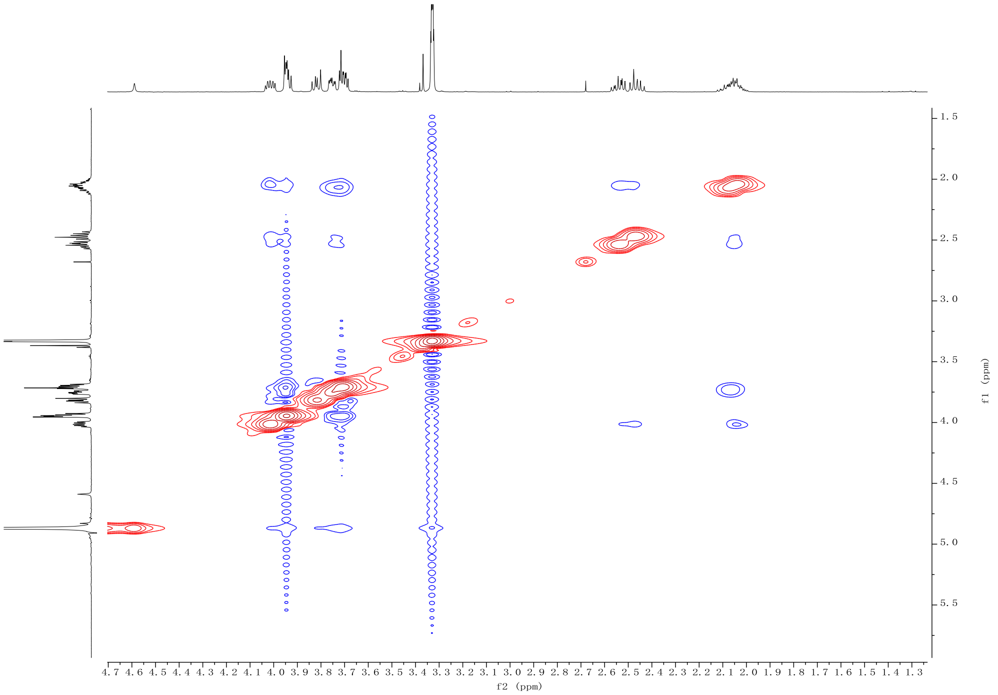

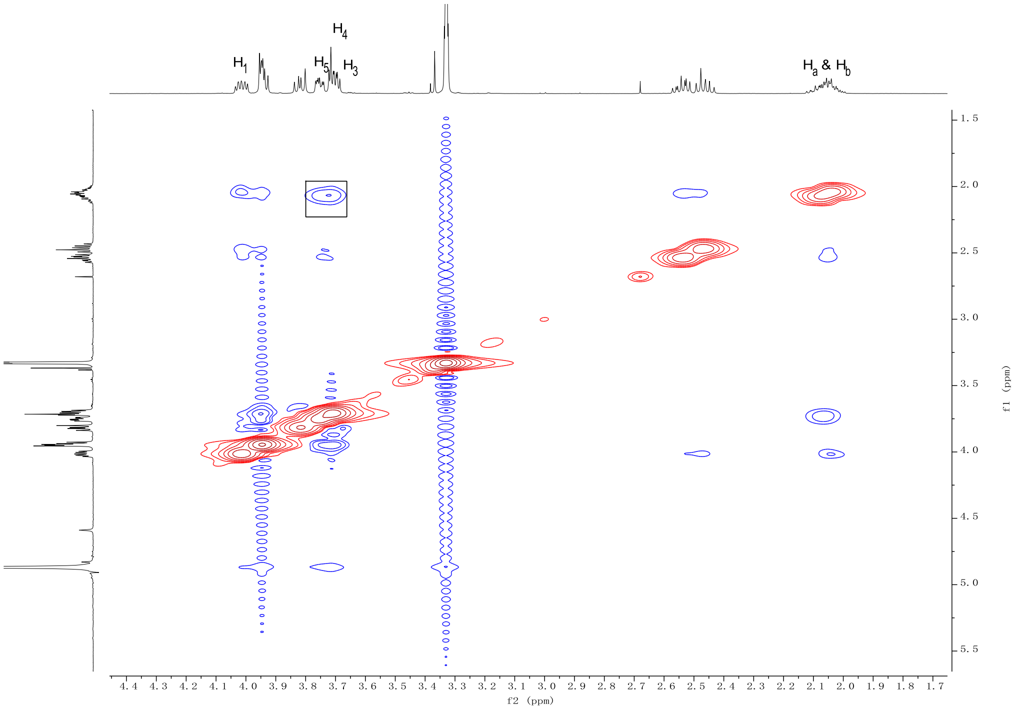

^{1}H NMR spectrum of compound 20

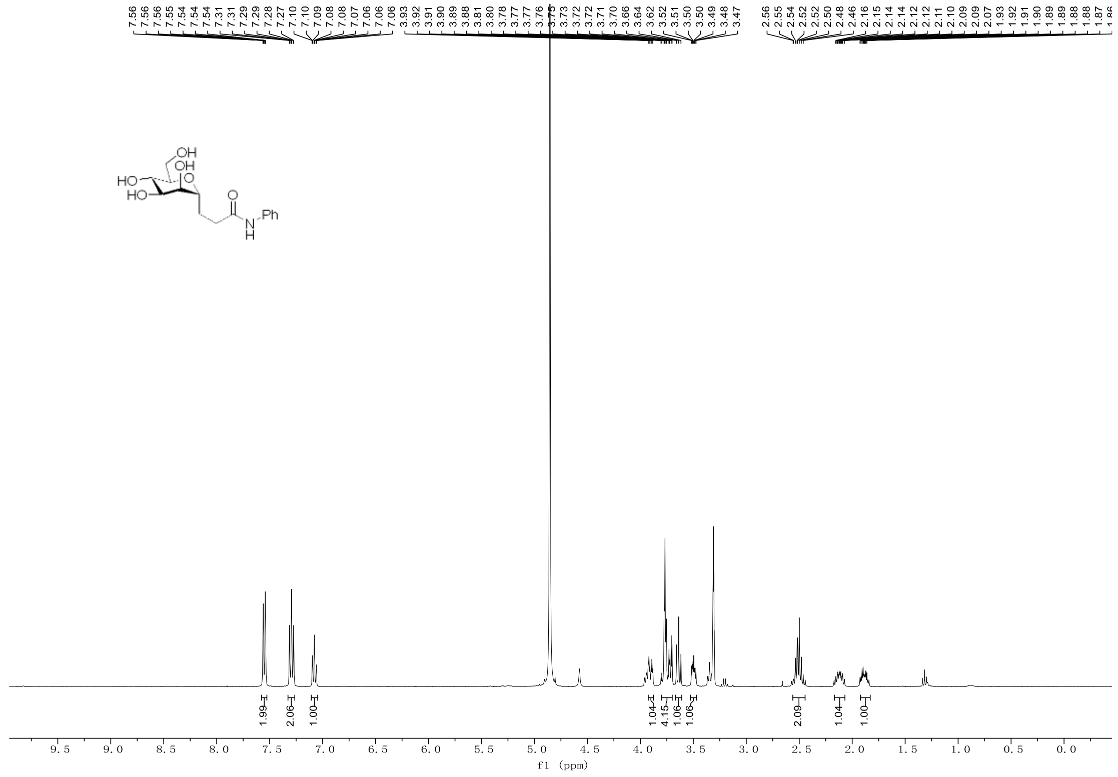

^{13}C NMR spectrum of compound 20

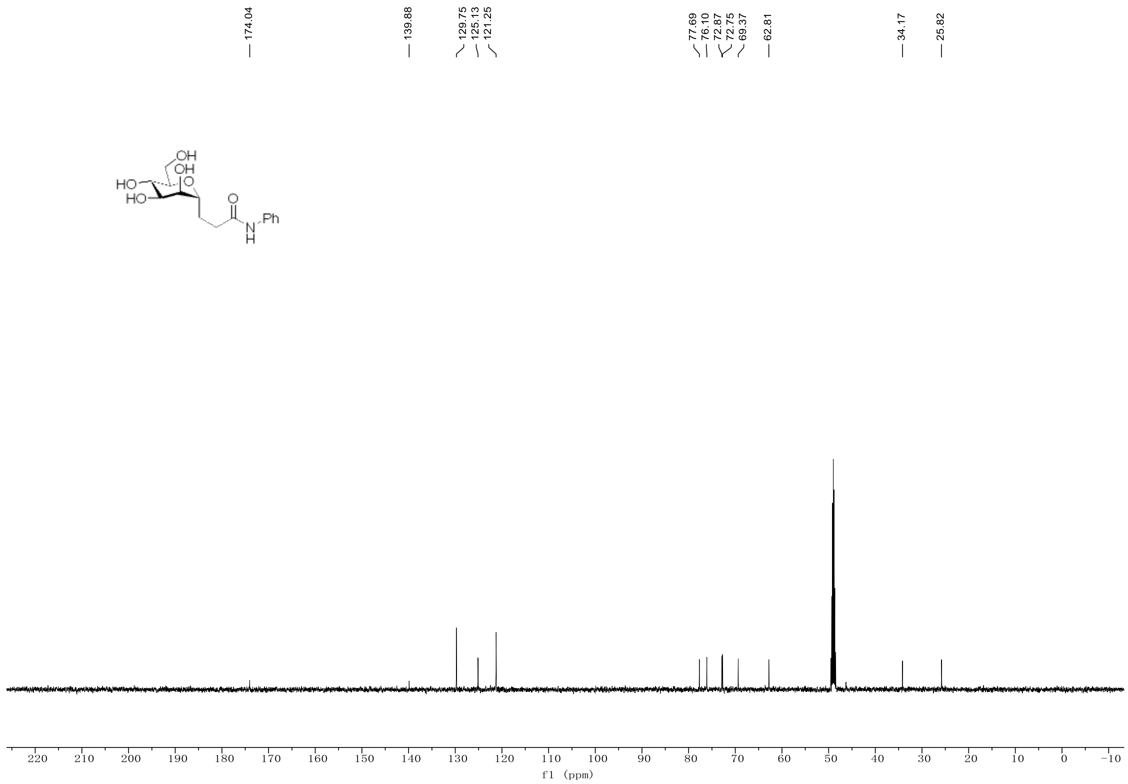

COSY spectrum of compound 20

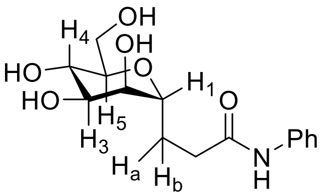

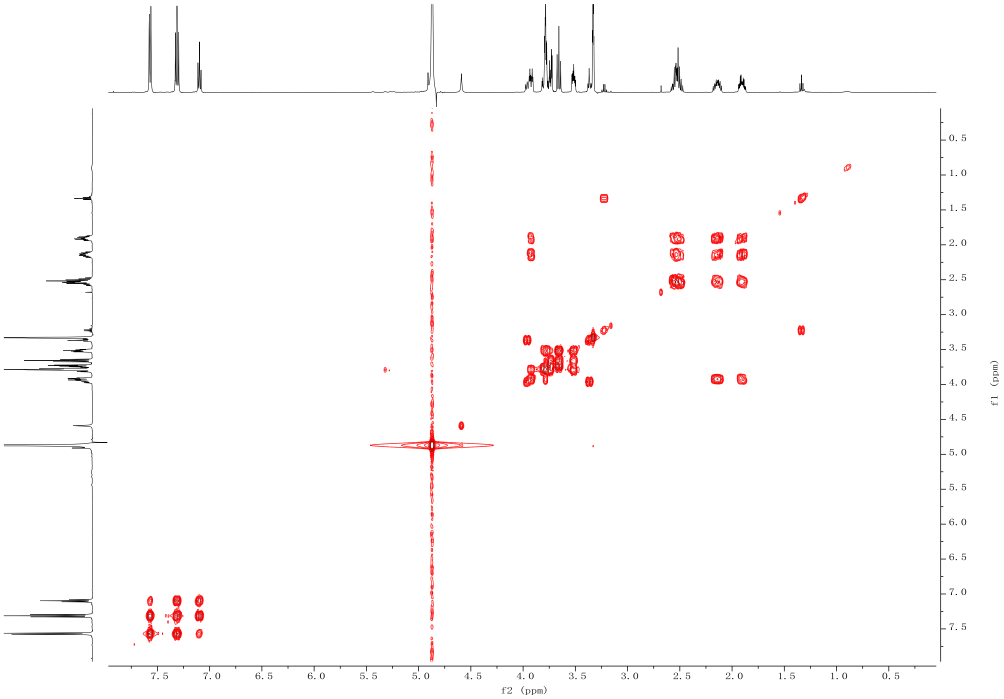

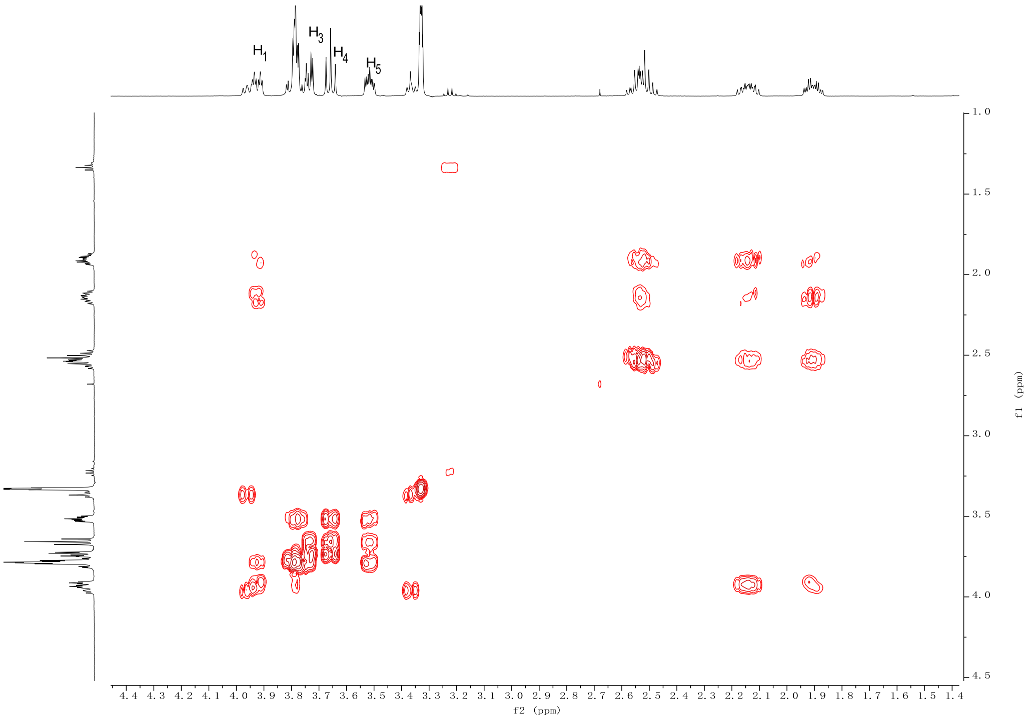

NOE spectrum of compound 20

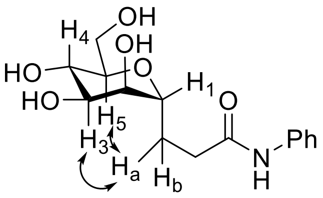

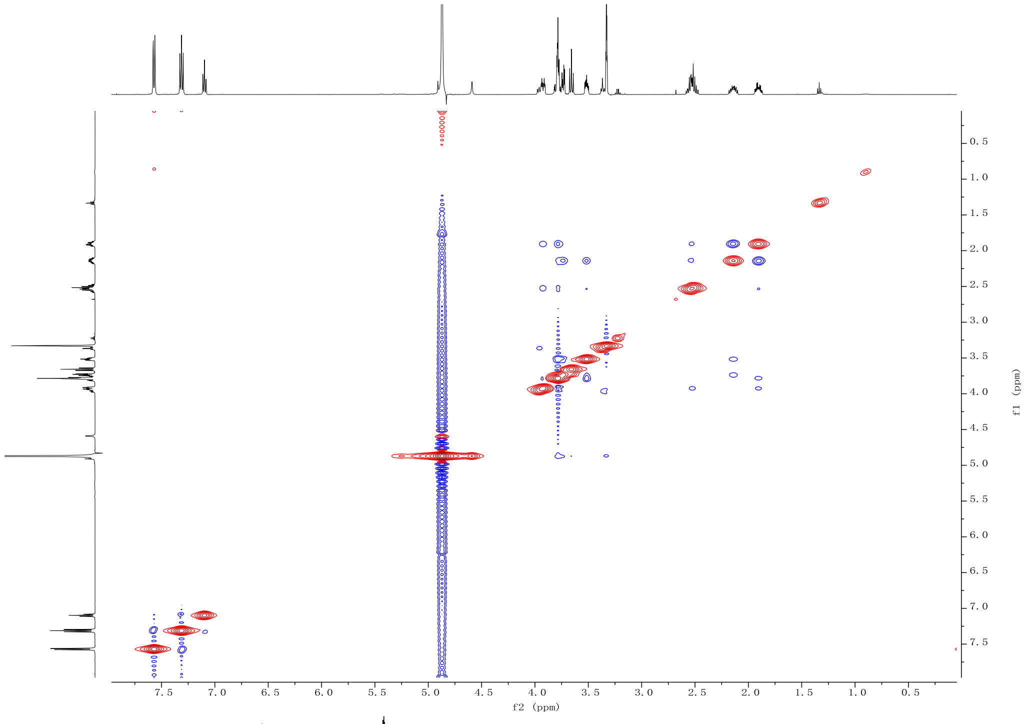

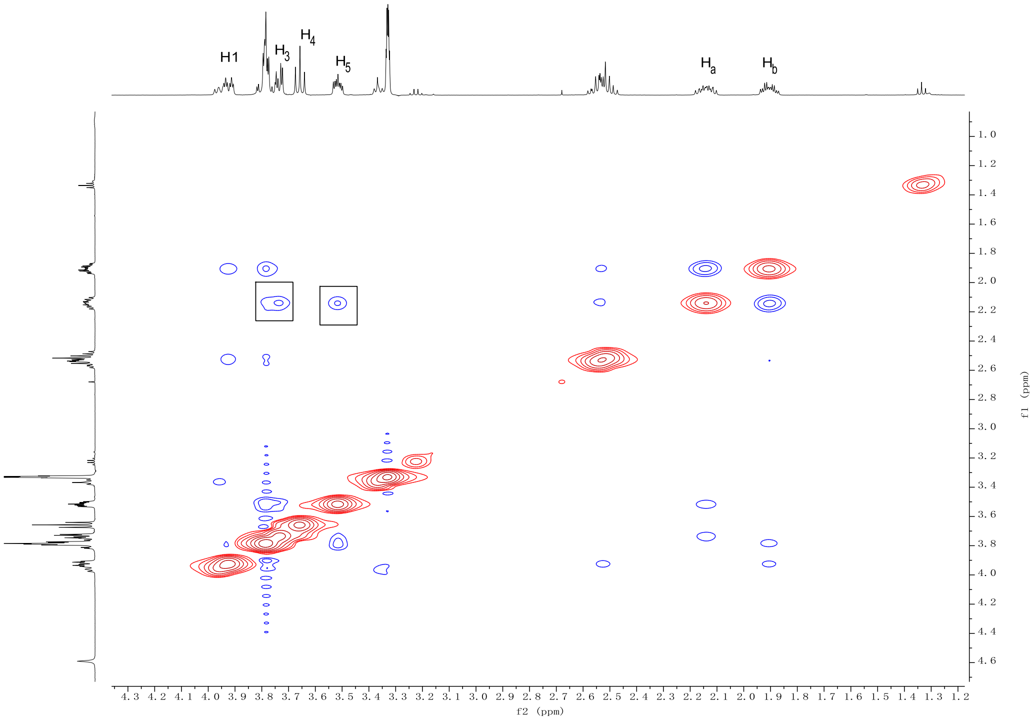

^{1}H NMR spectrum of compound 21

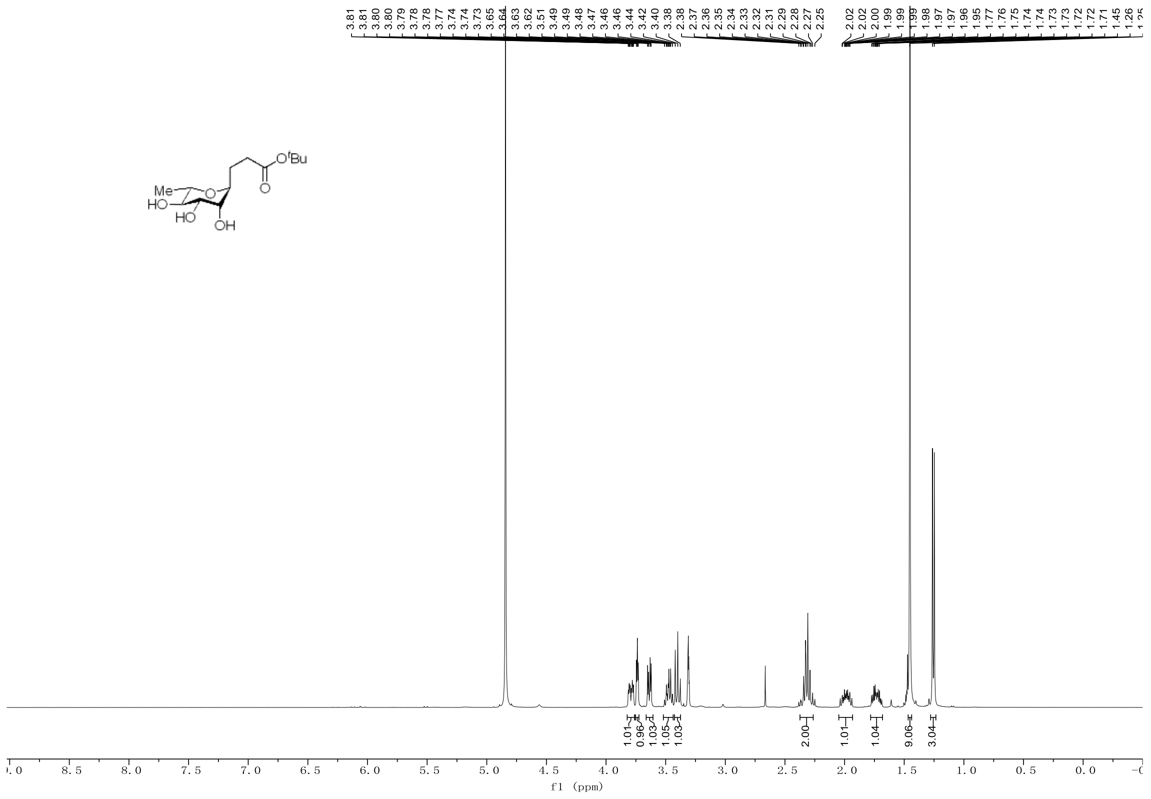

^{13}C NMR spectrum of compound 21

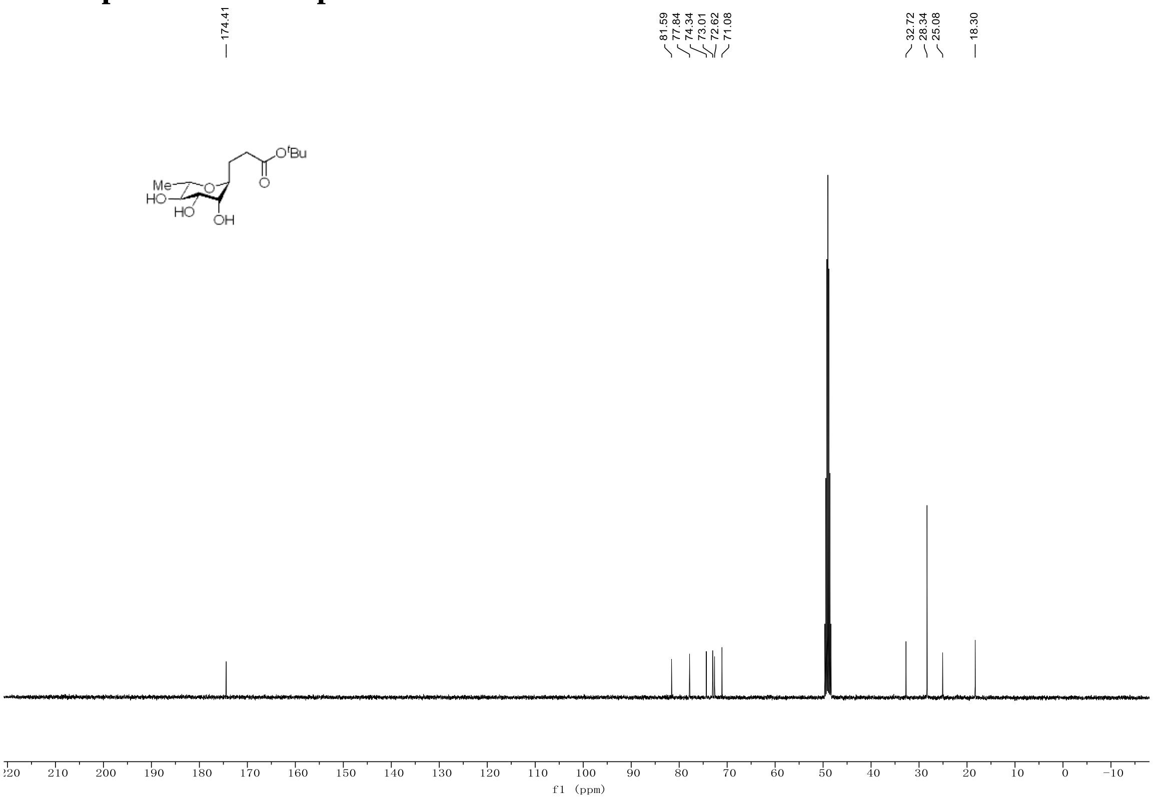

COSY spectrum of compound 21

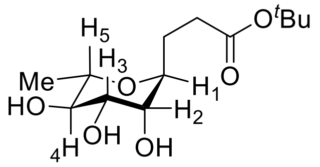

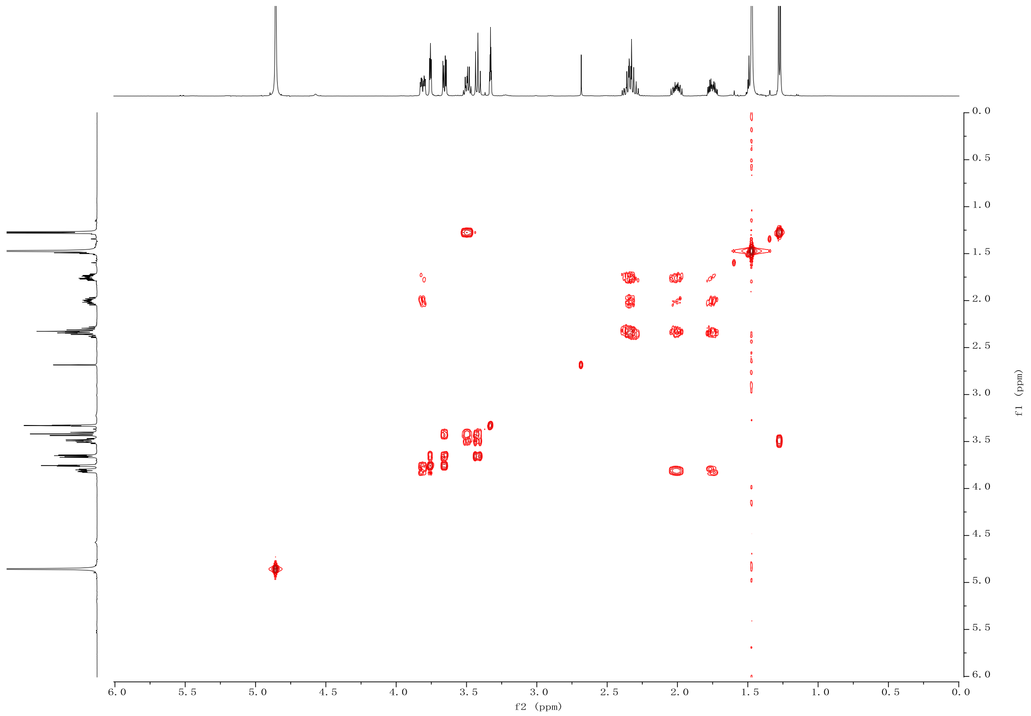

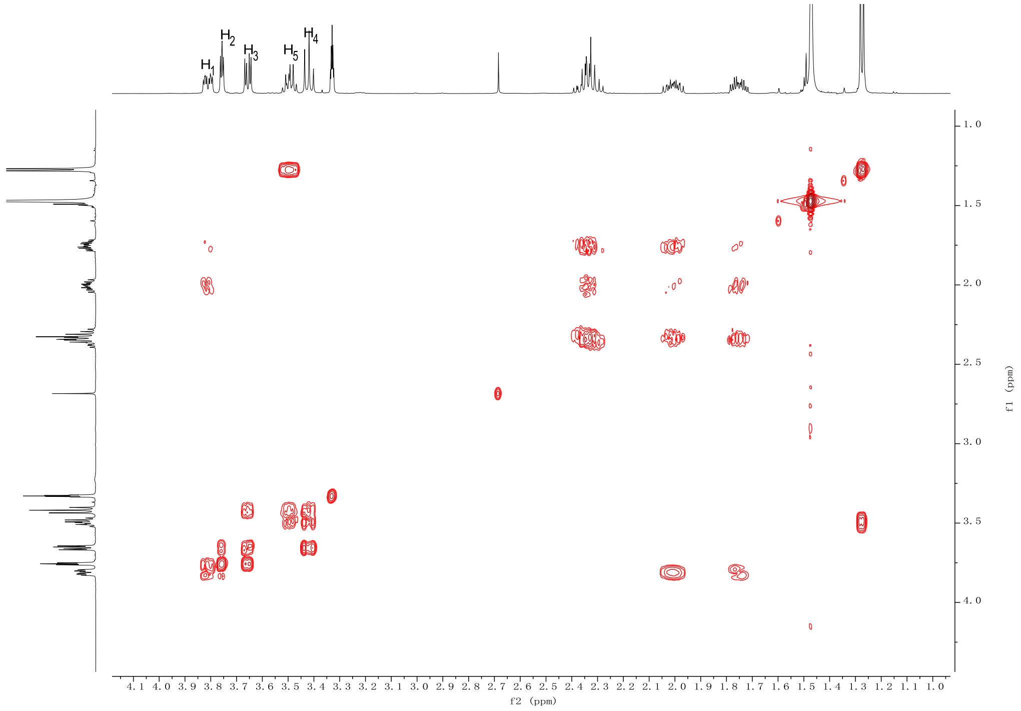

NOE spectrum of compound 21

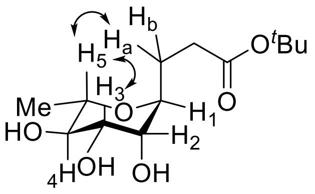

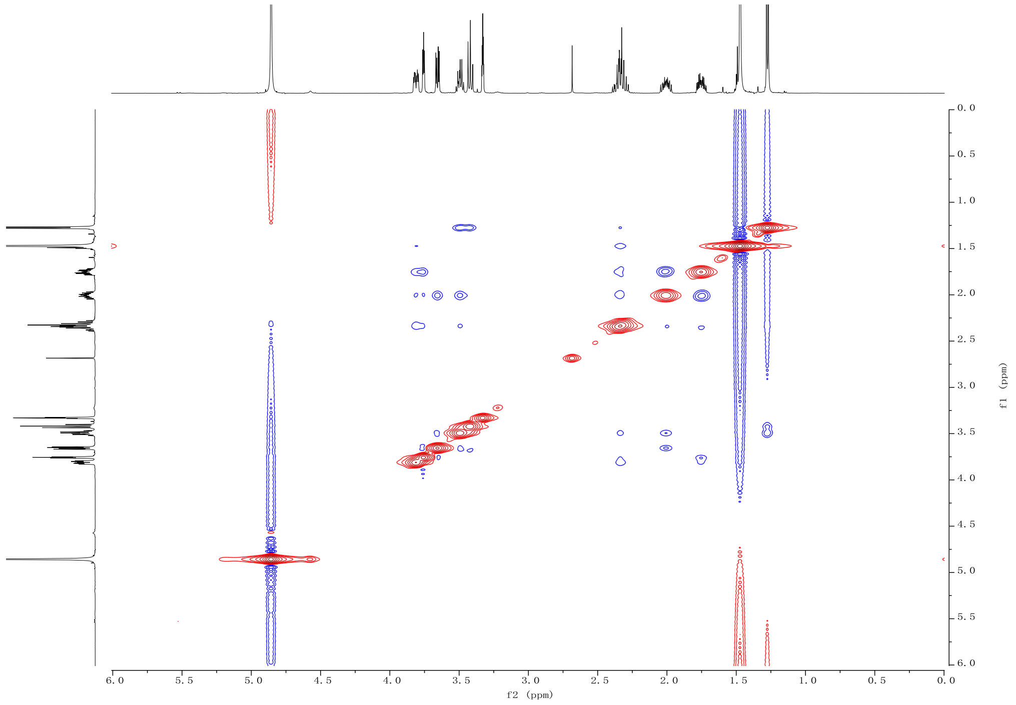

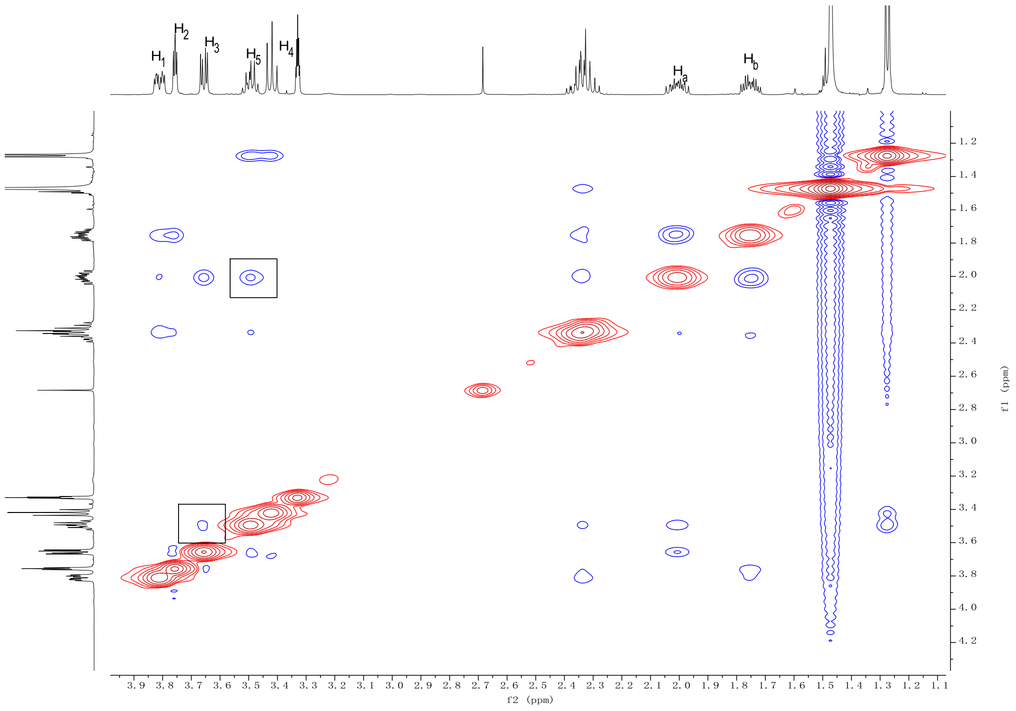

^{1}H NMR spectrum of compound 22

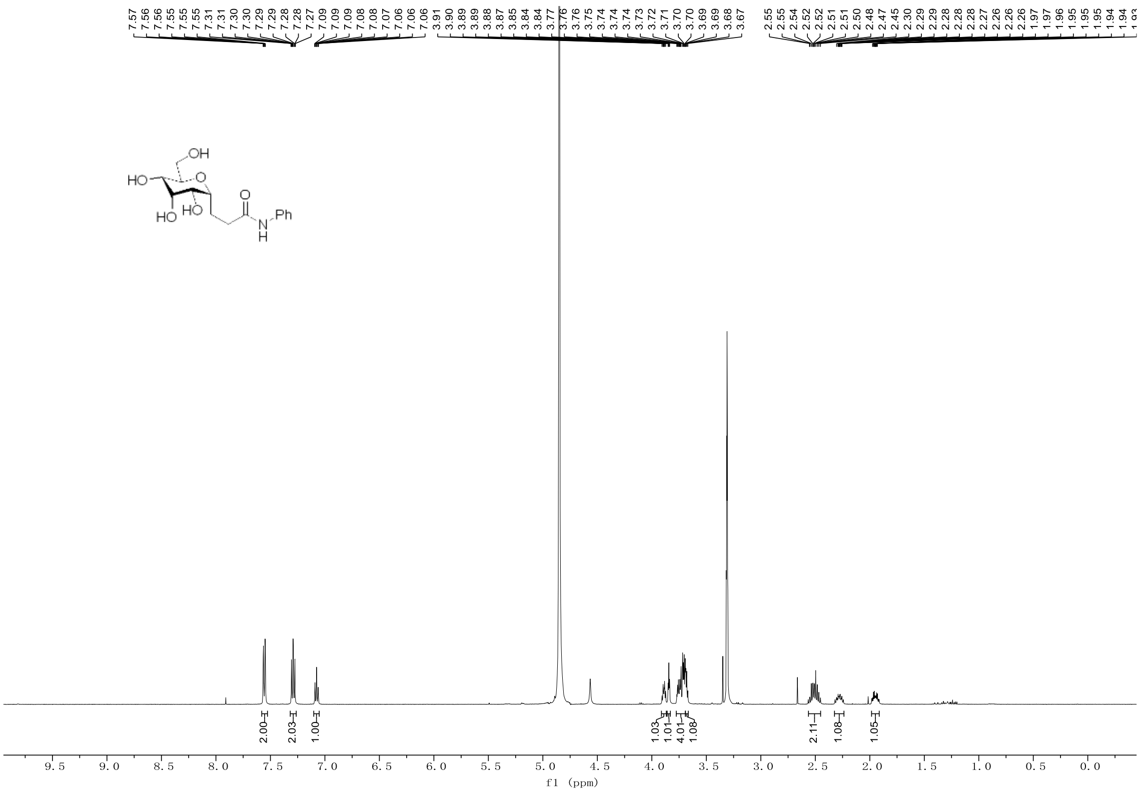

^{13}C NMR spectrum of compound 22

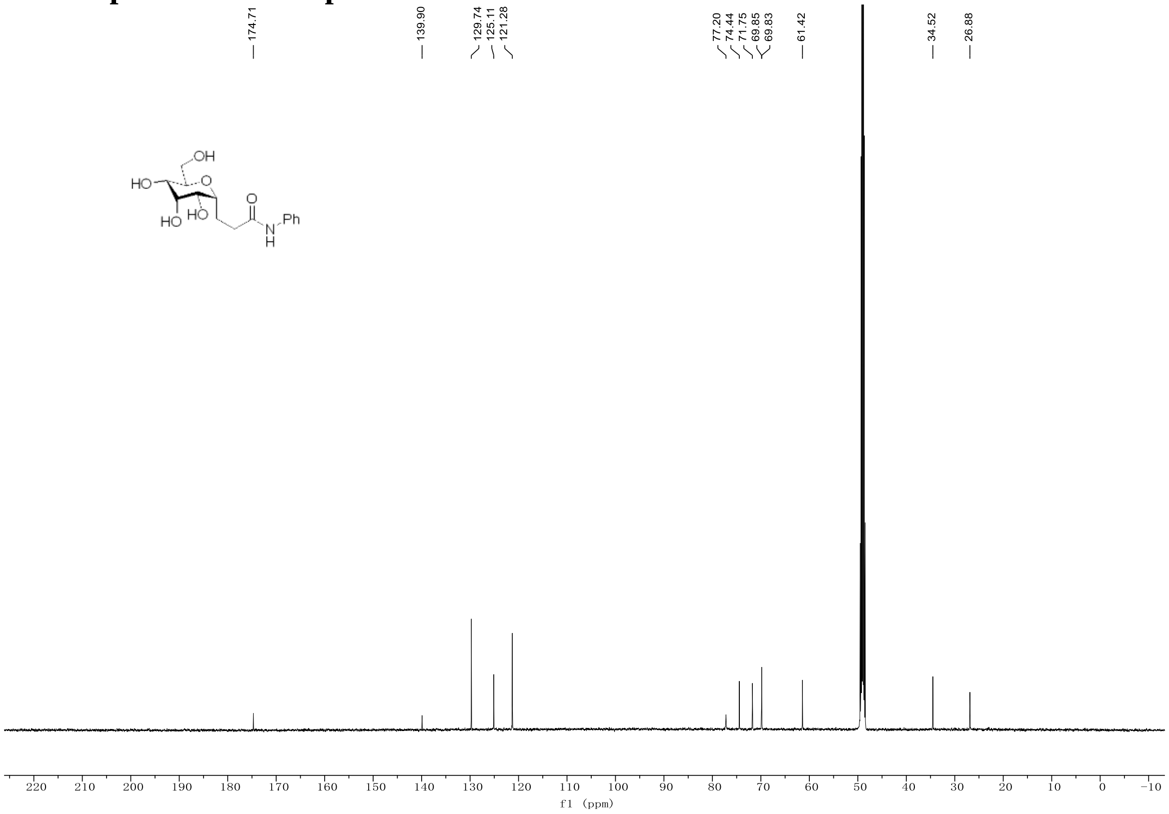

COSY spectrum of compound 22

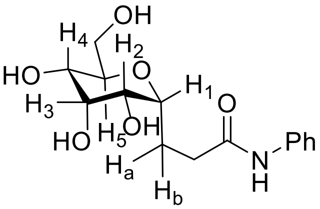

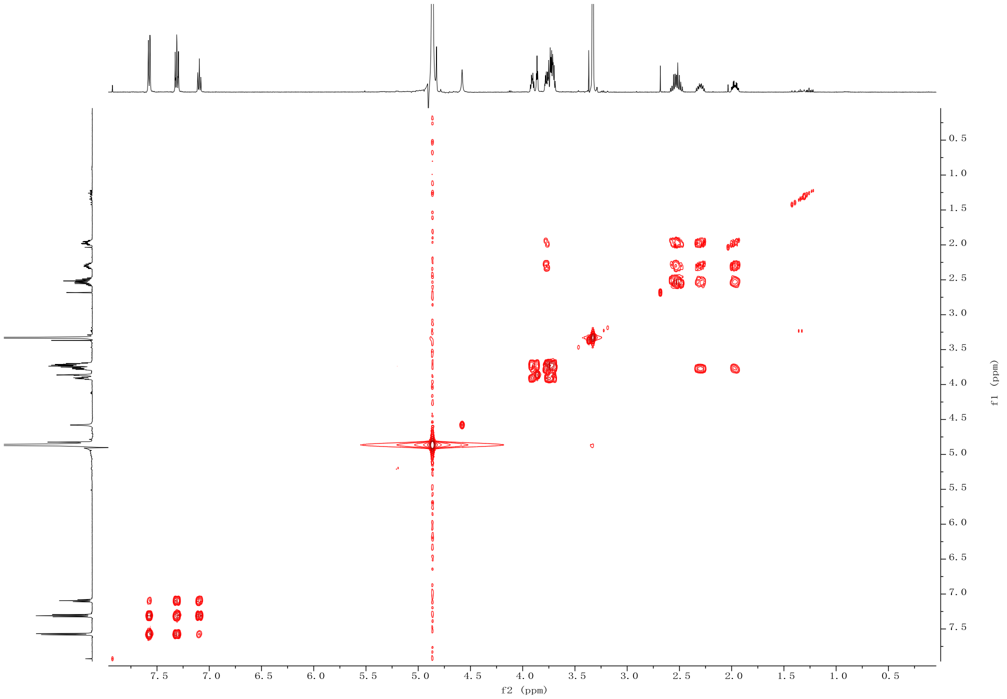

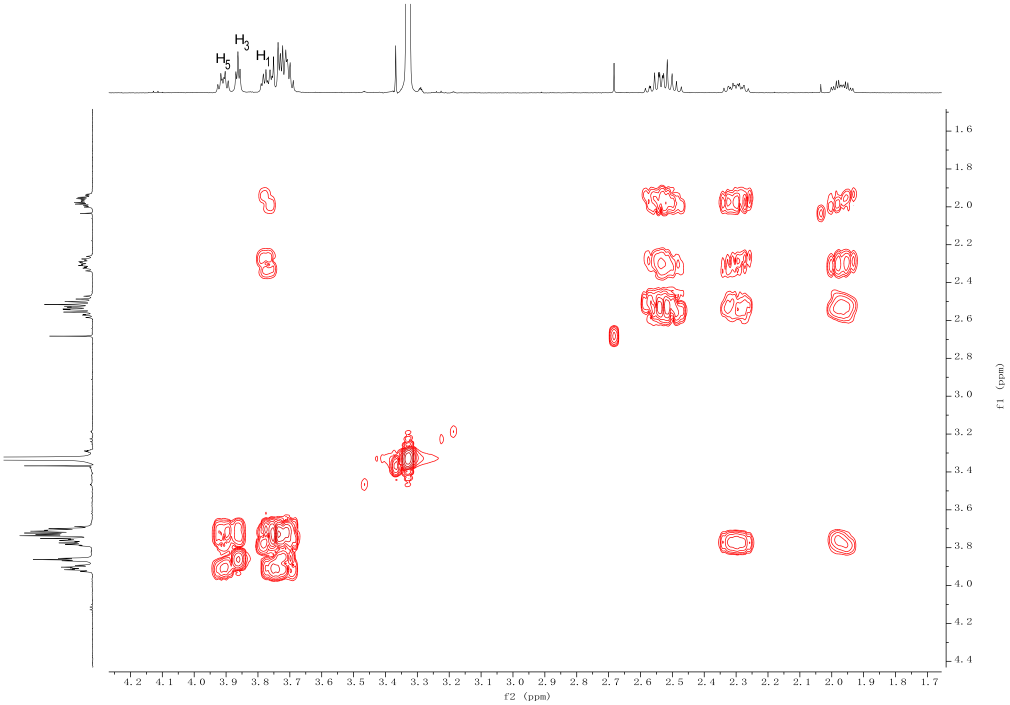

NOE spectrum of compound 22

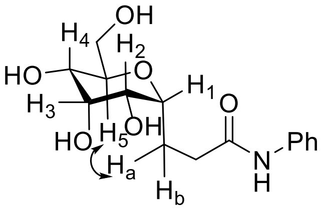

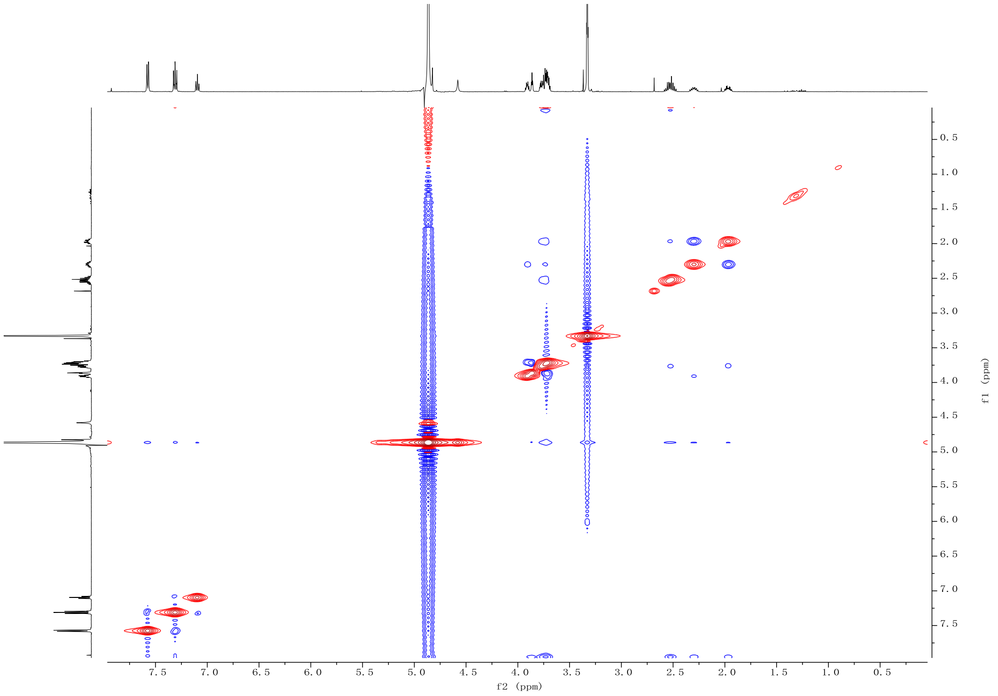

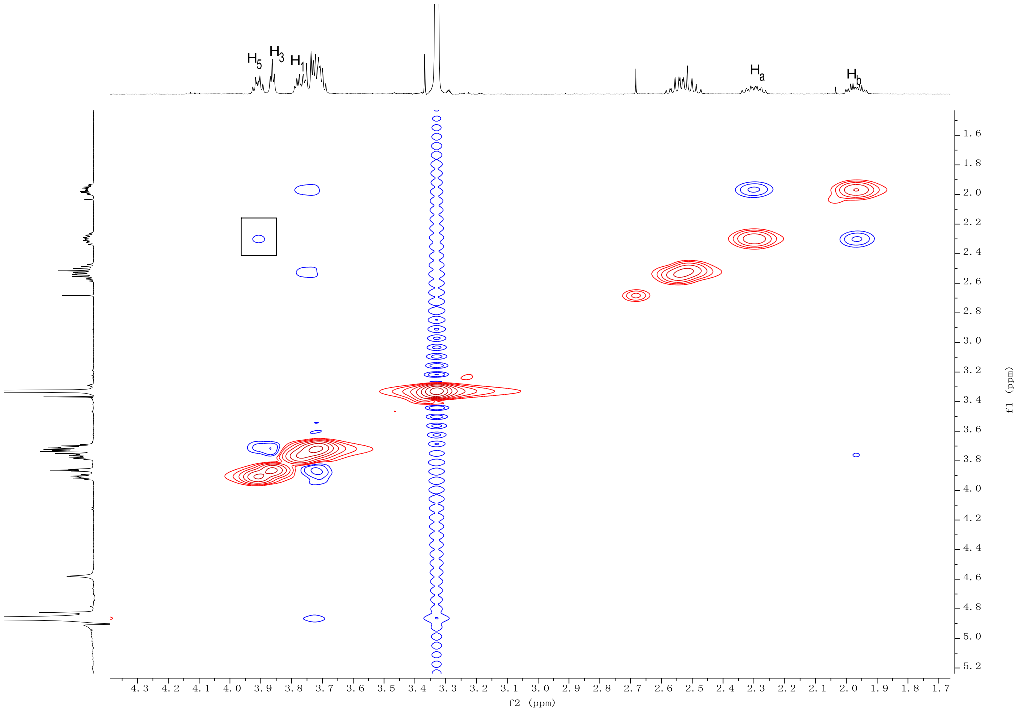

^{1}H NMR spectrum of compound 23

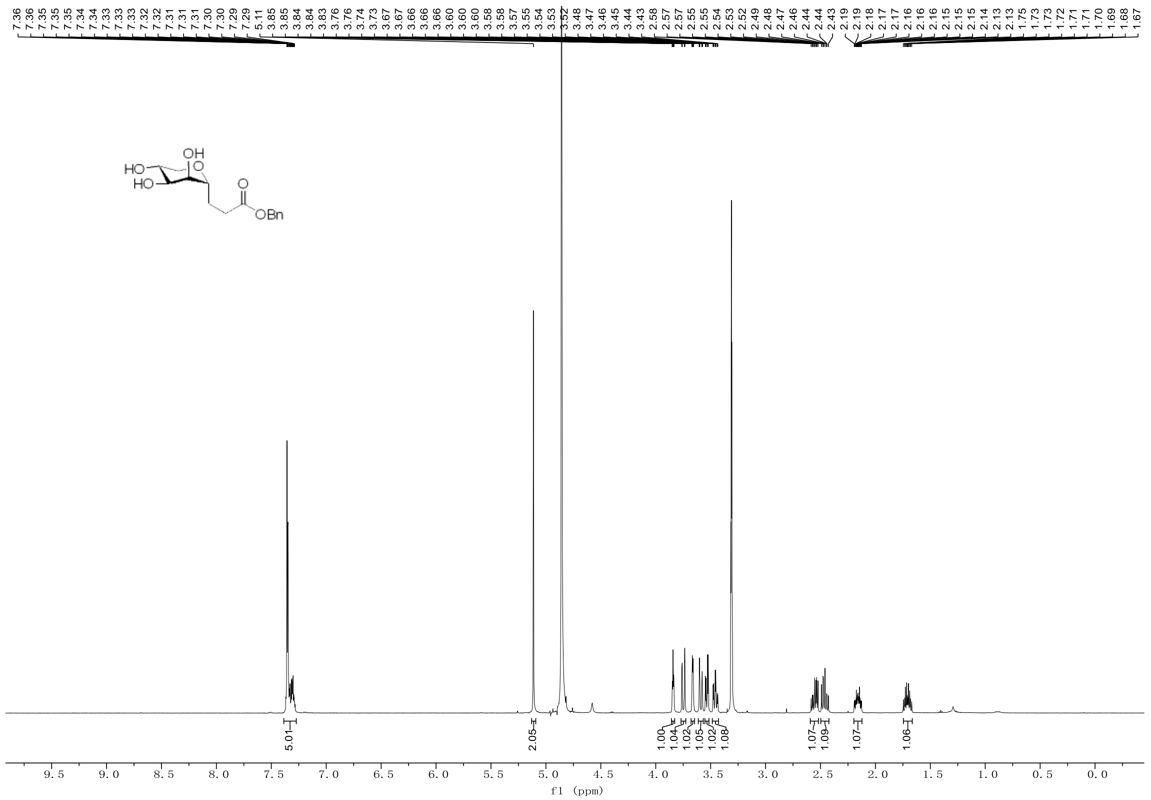

^{13}C NMR spectrum of compound 23

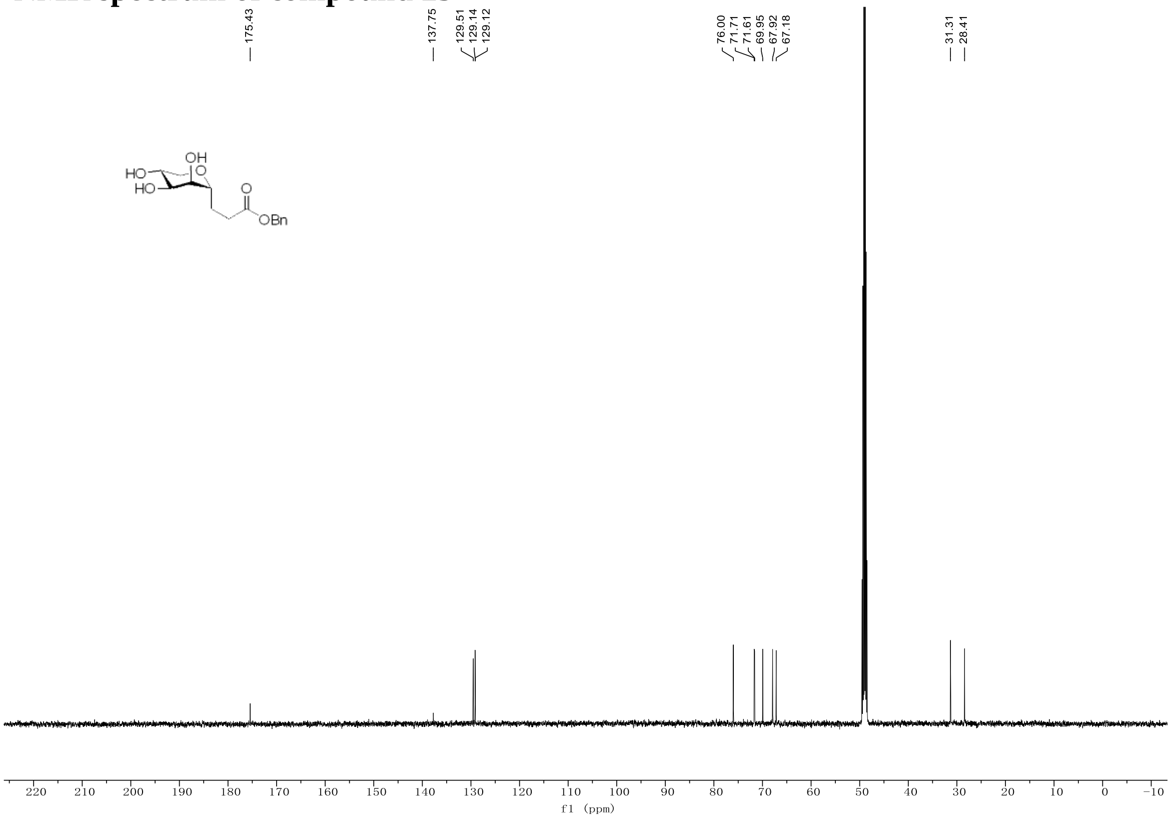

COSY spectrum of compound 23

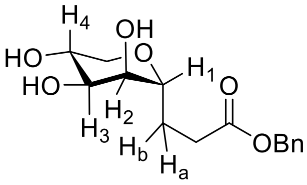

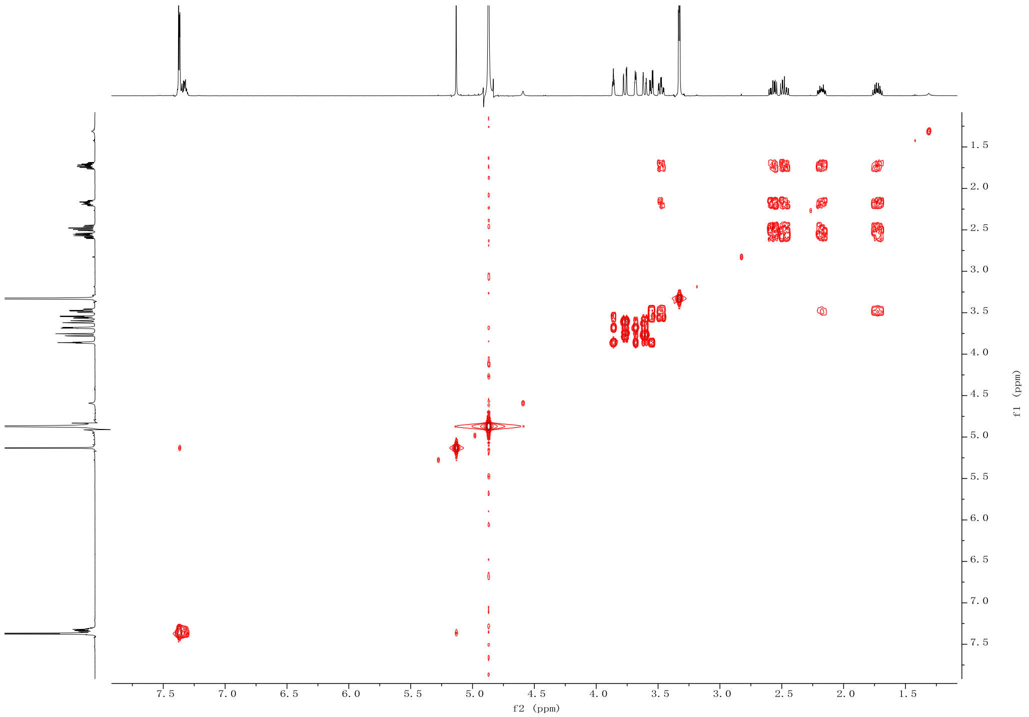

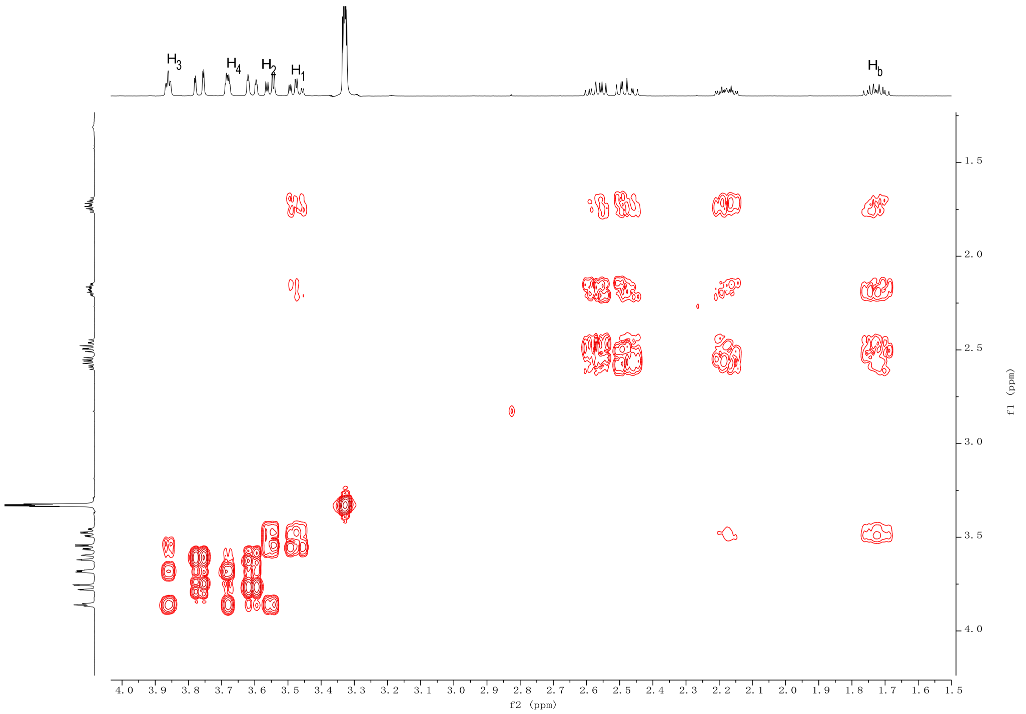

NOE spectrum of compound 23

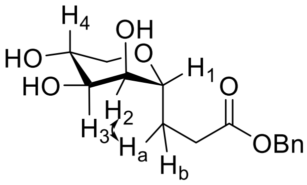

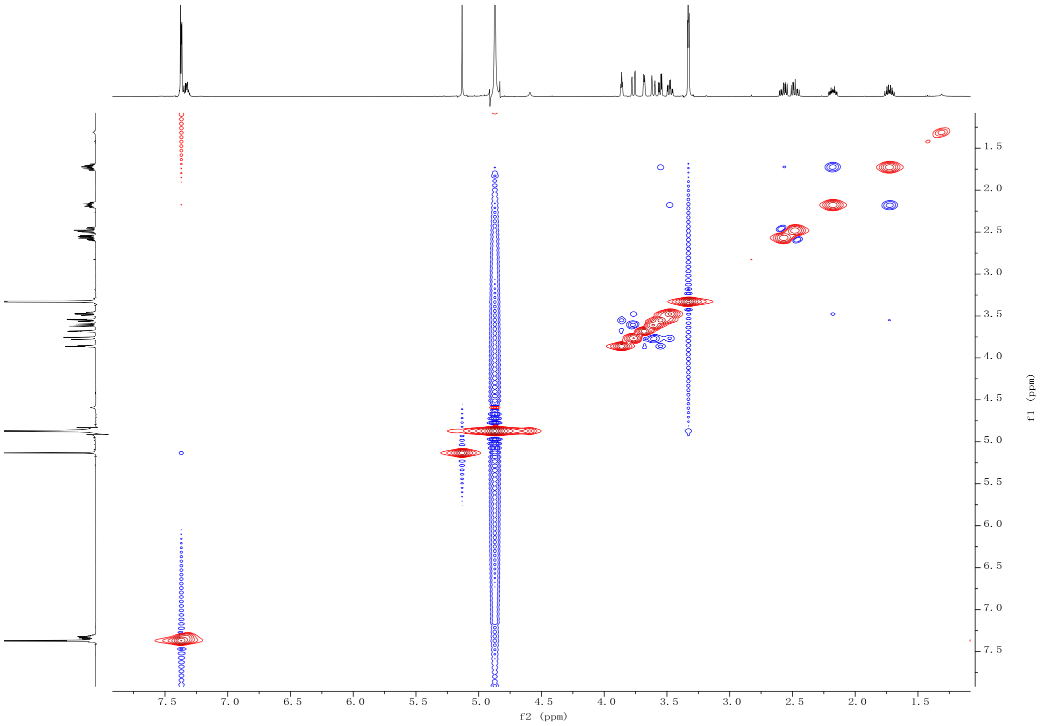

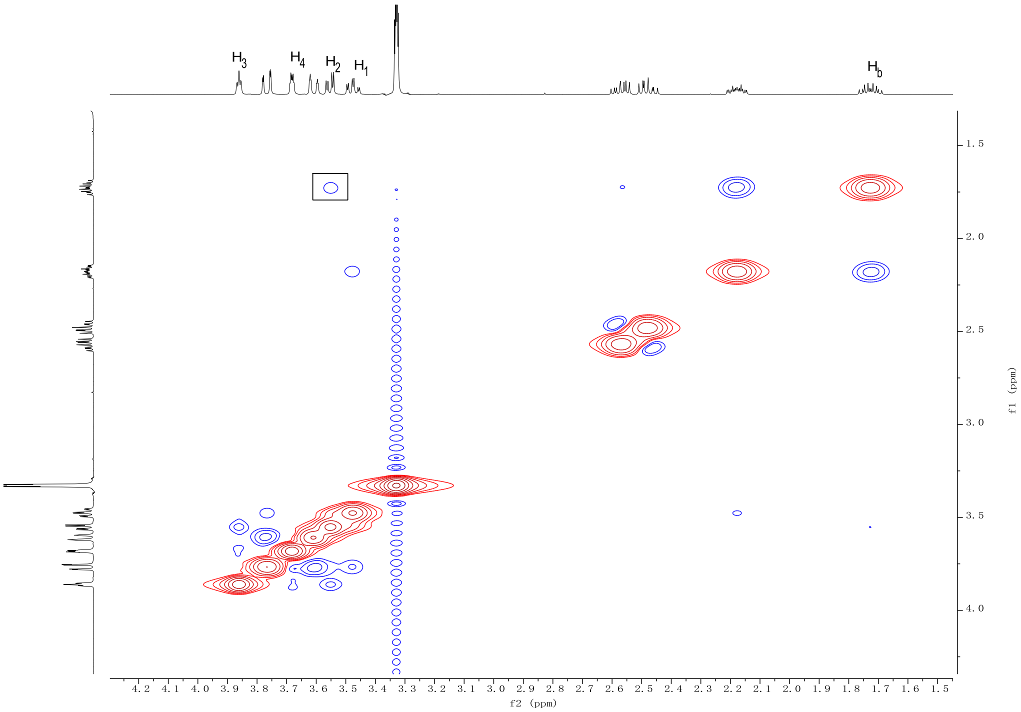

^{1}H NMR spectrum of compound 24

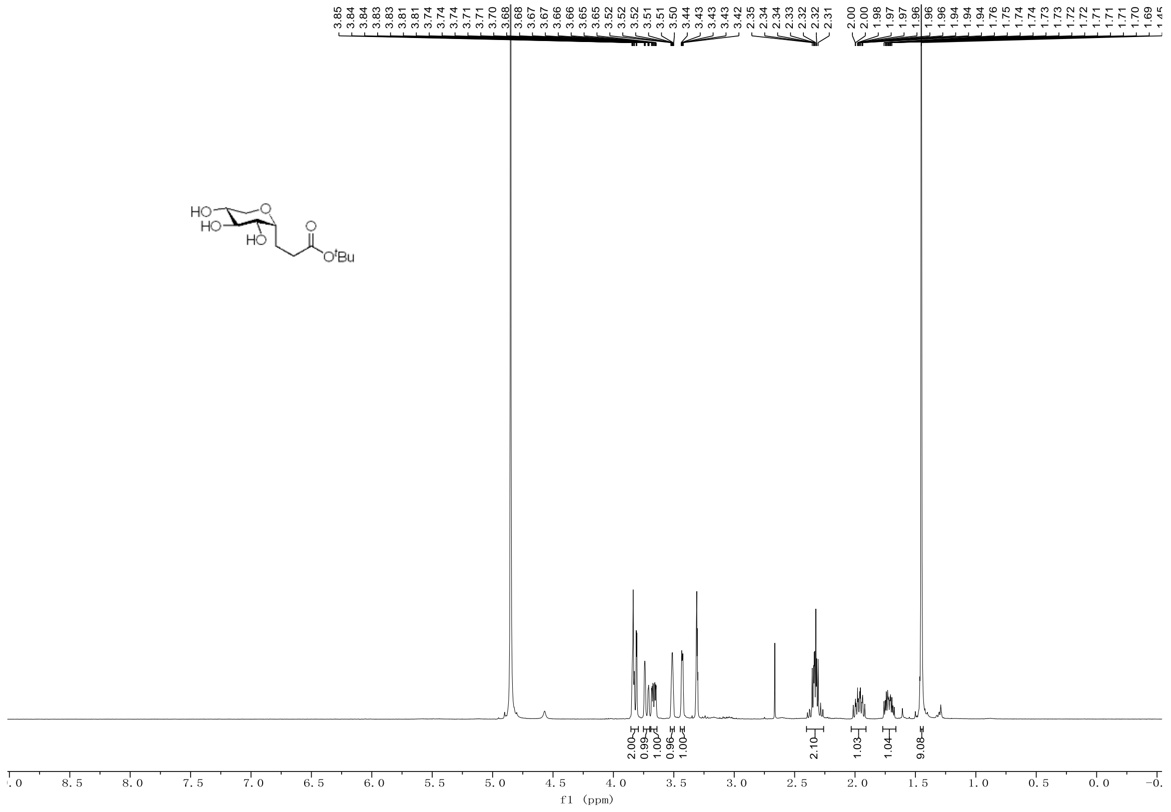

^{13}C NMR spectrum of compound 24

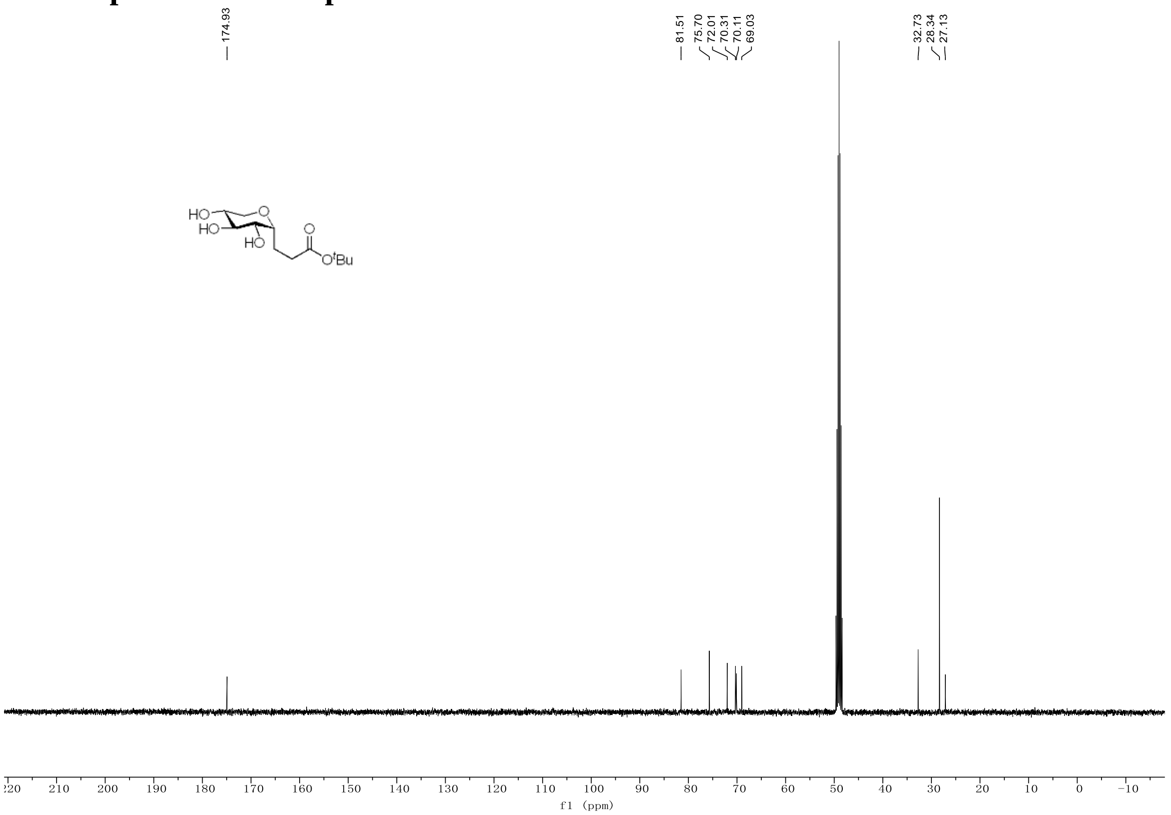

COSY spectrum of compound 24

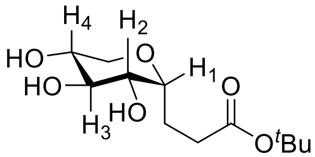

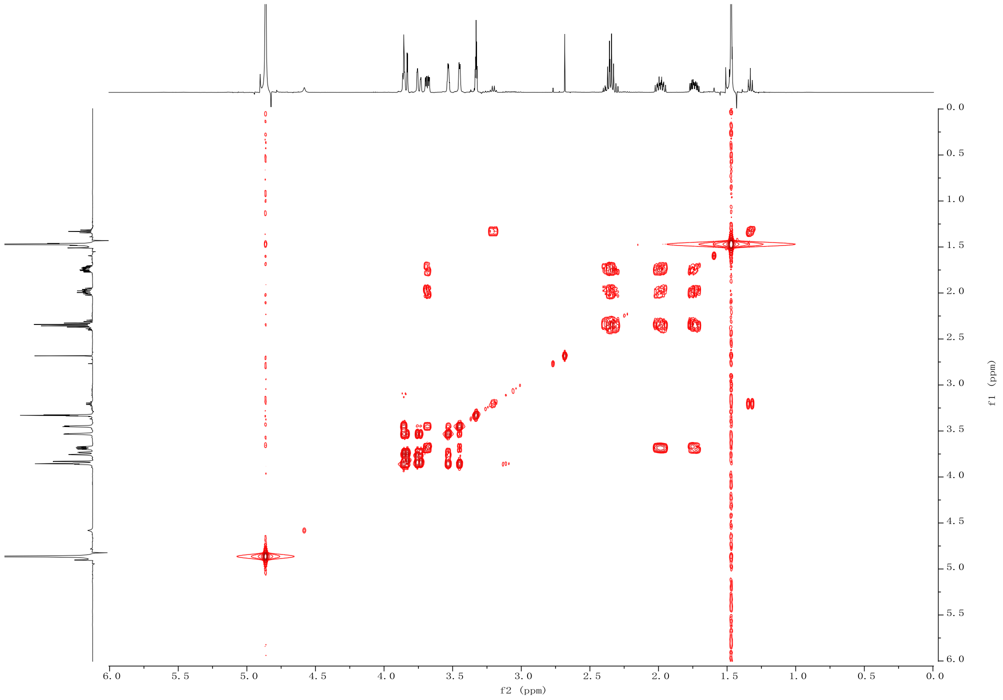

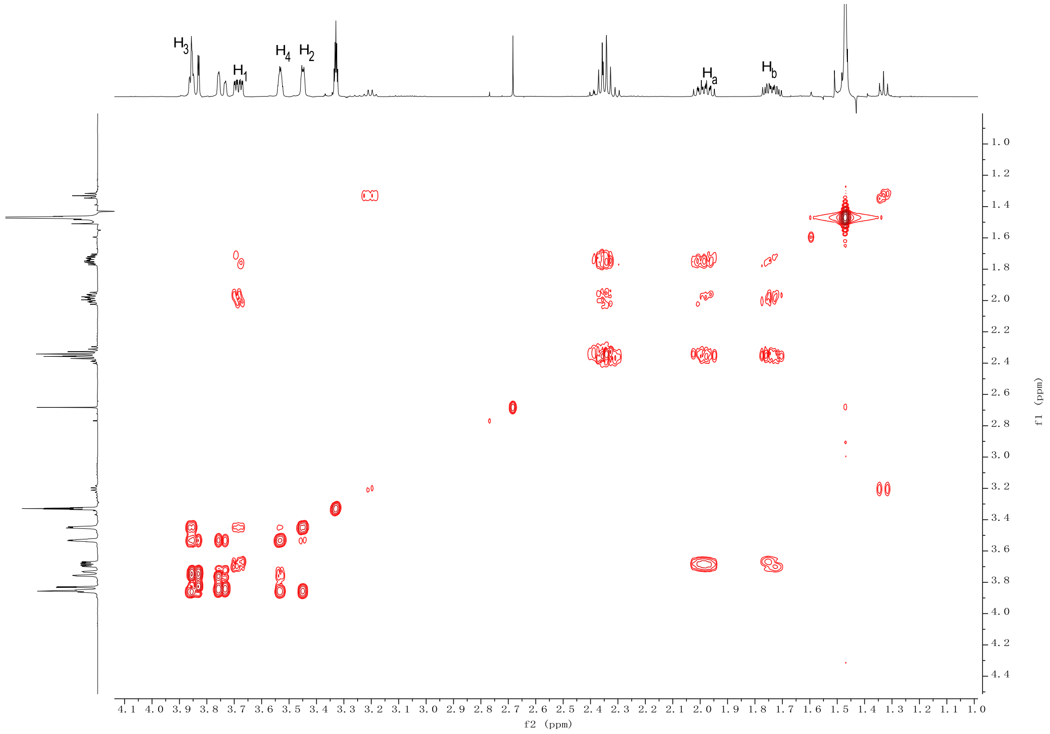

NOE spectrum of compound 24

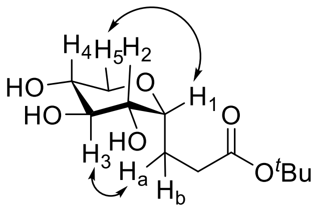

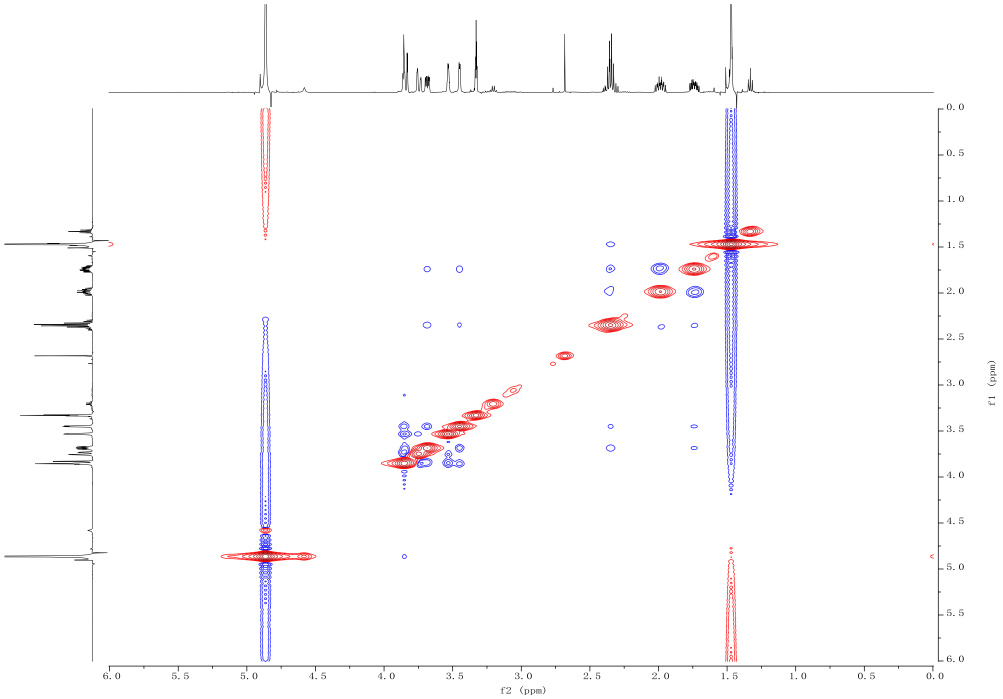

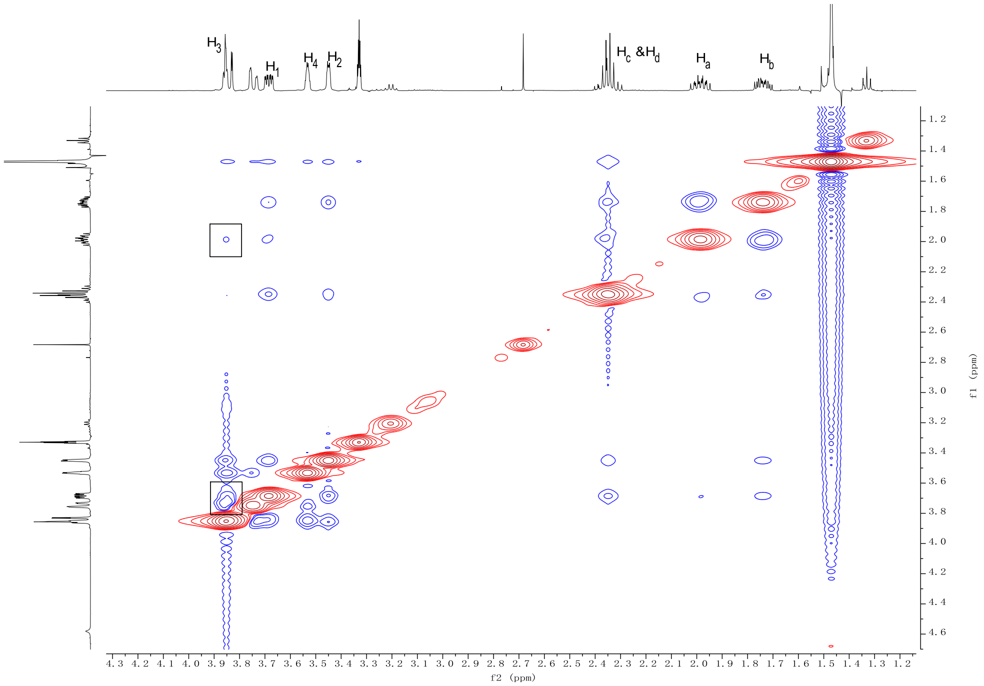

^{1}H NMR spectrum of compound 25

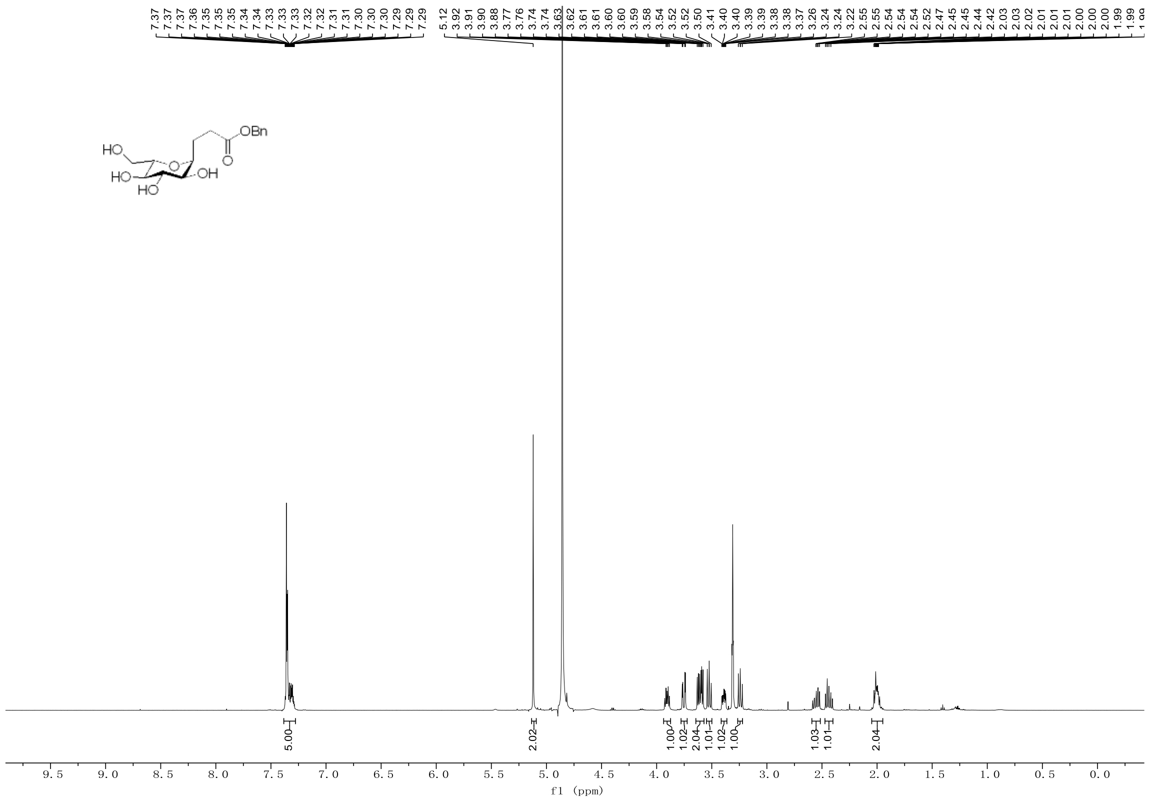

^{13}C NMR spectrum of compound 25

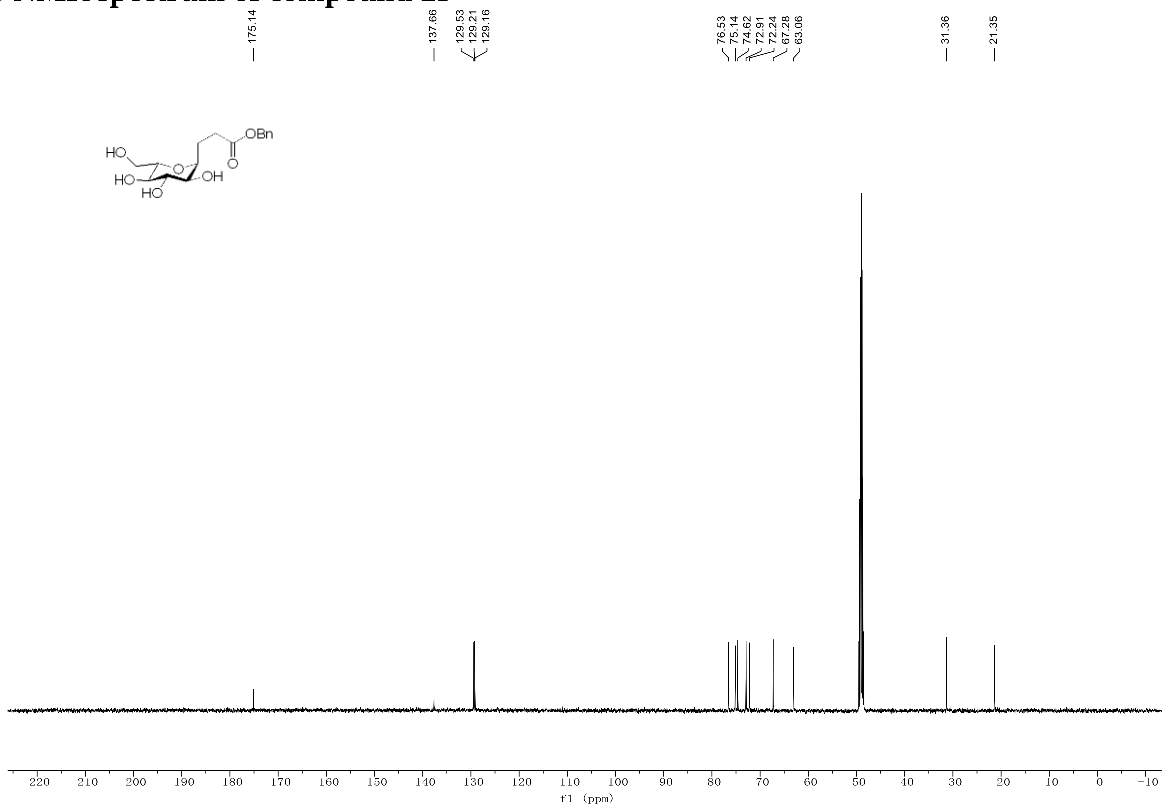

COSY spectrum of compound 25

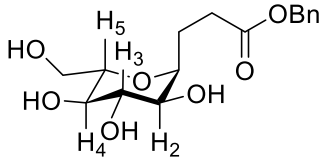

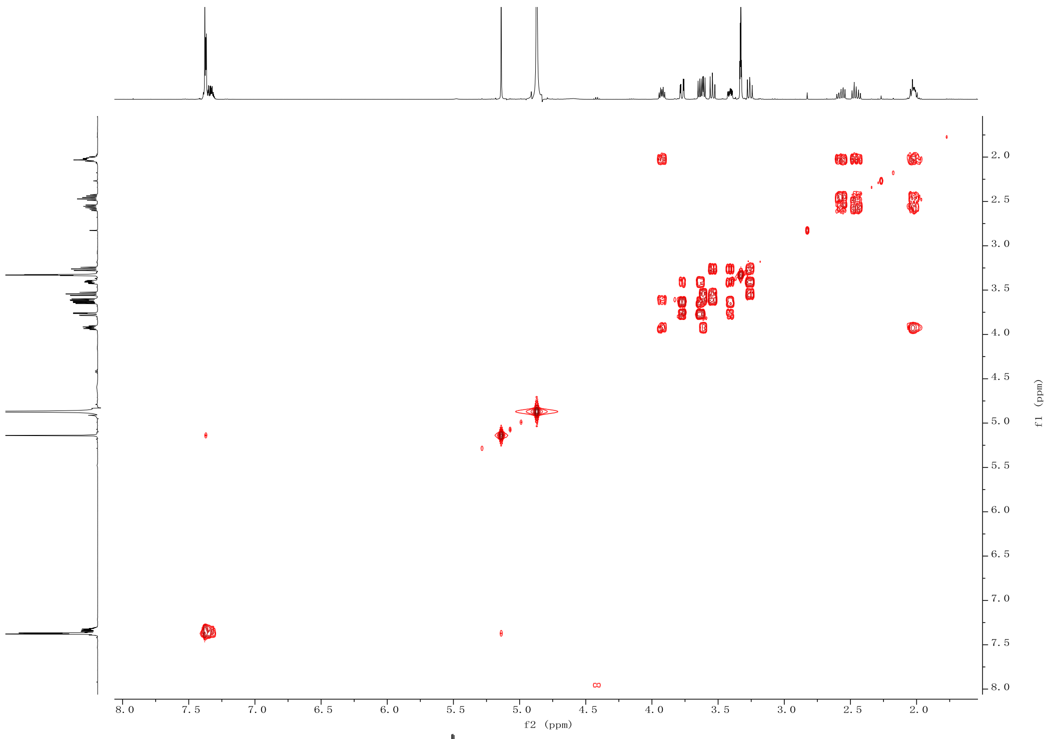

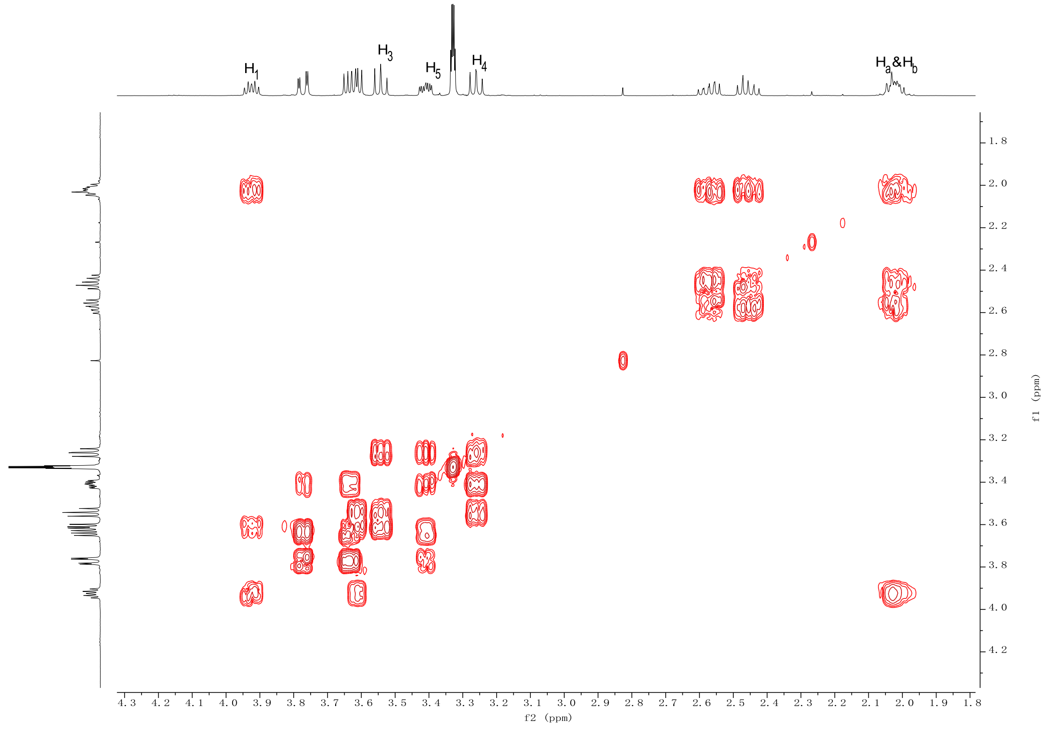

NOE spectrum of compound 25

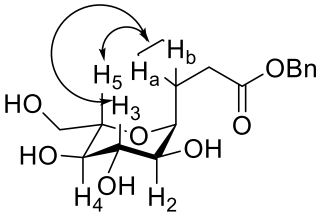

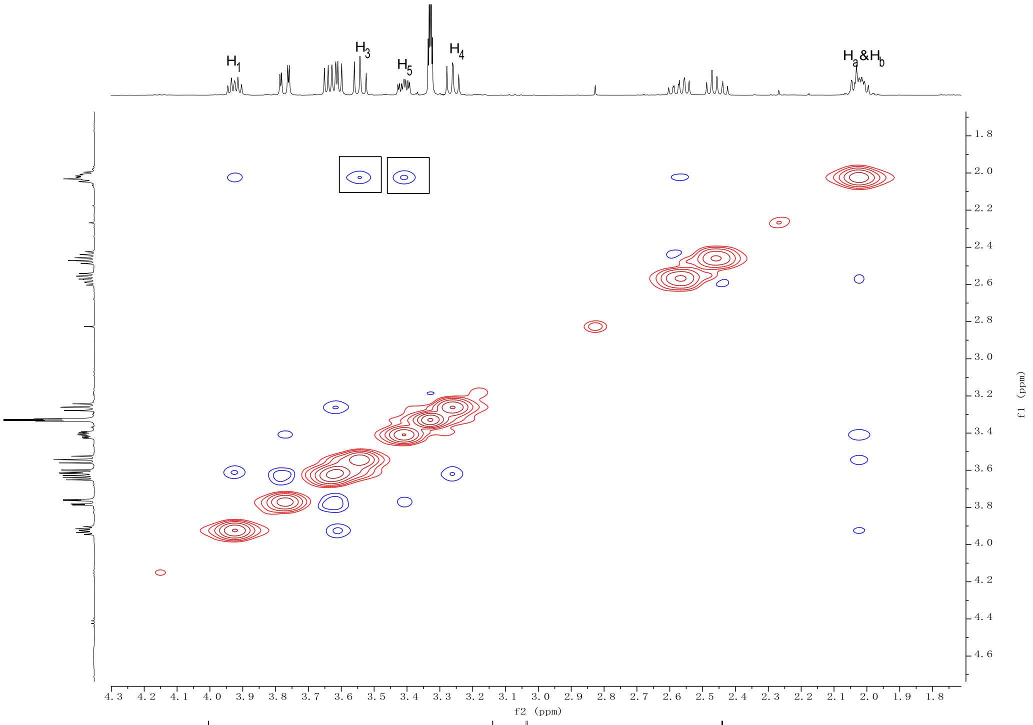

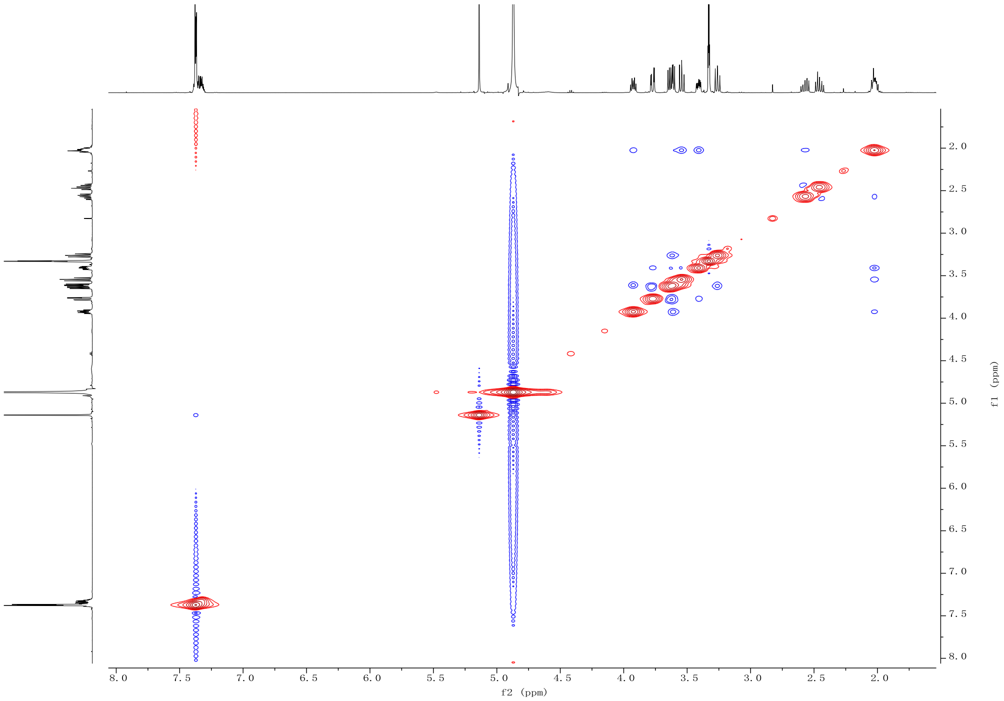

^{1}H NMR spectrum of compound 15

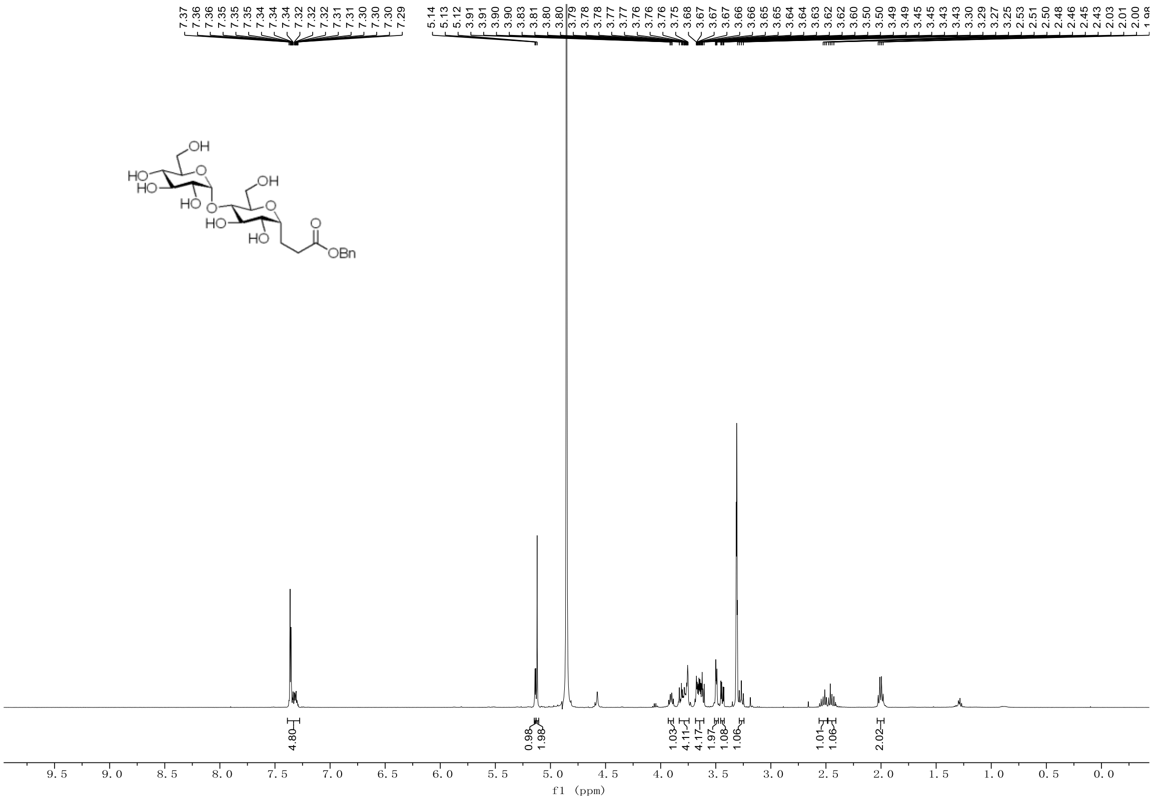

^{13}C NMR spectrum of compound 15

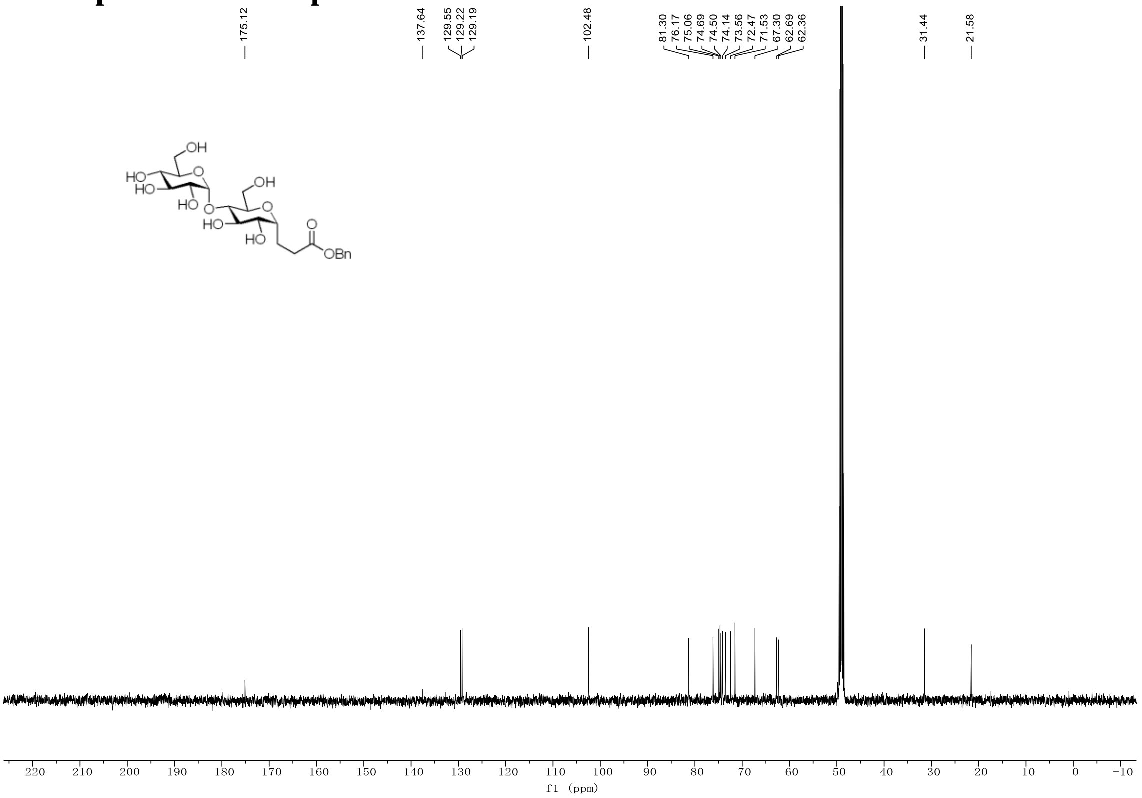

COSY spectrum of compound 15

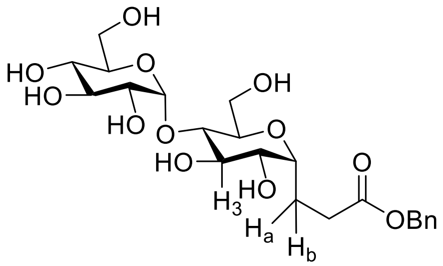

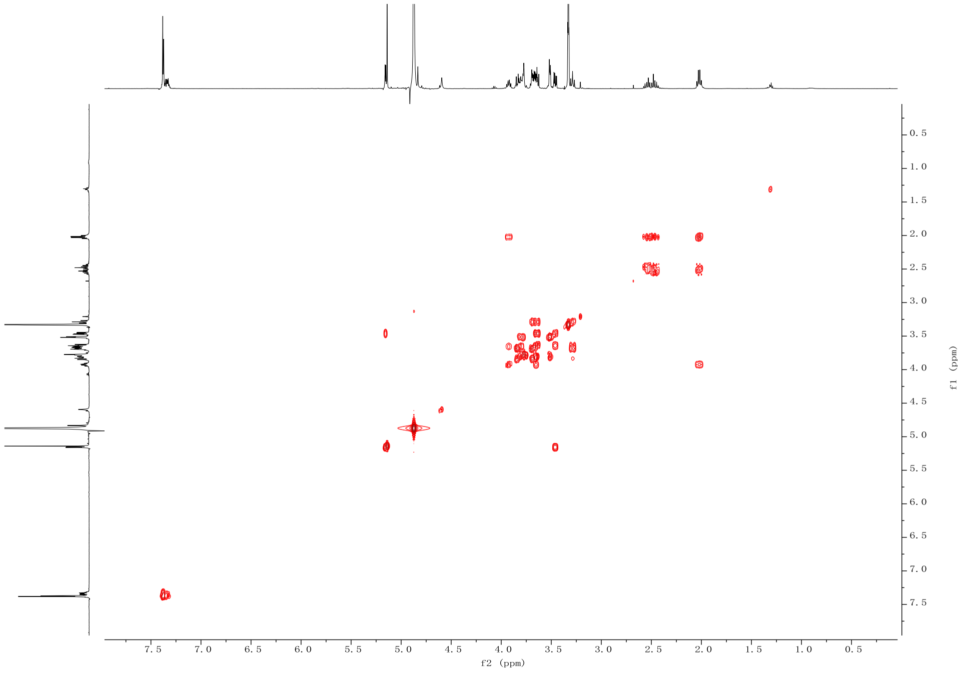

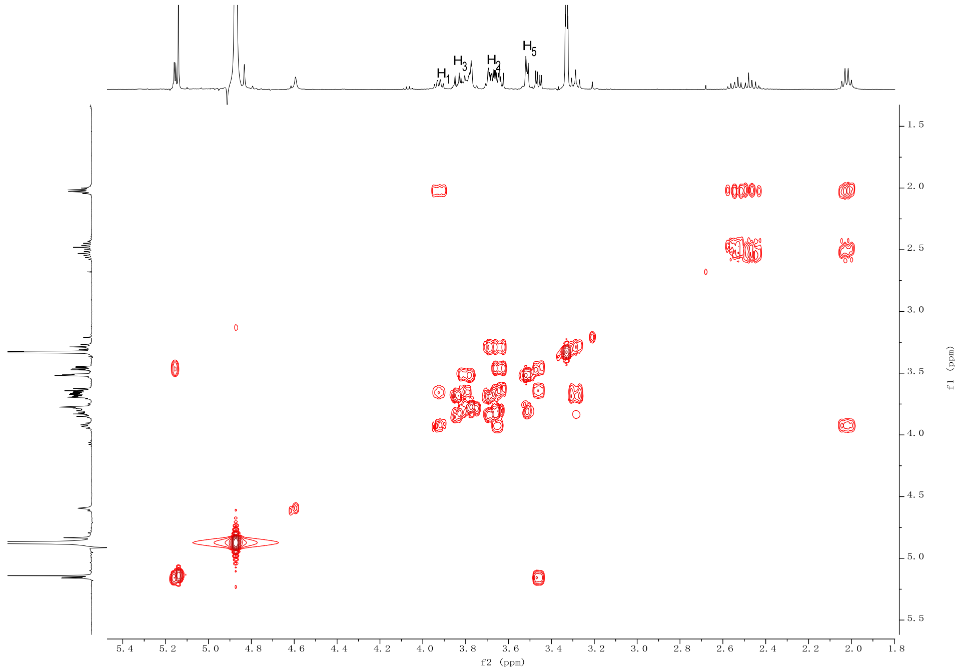

NOE spectrum of compound 15

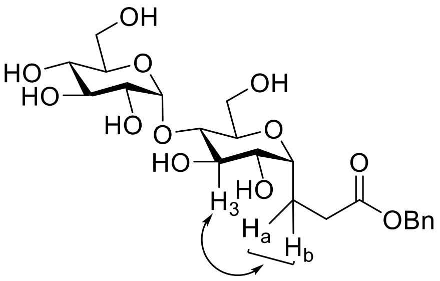

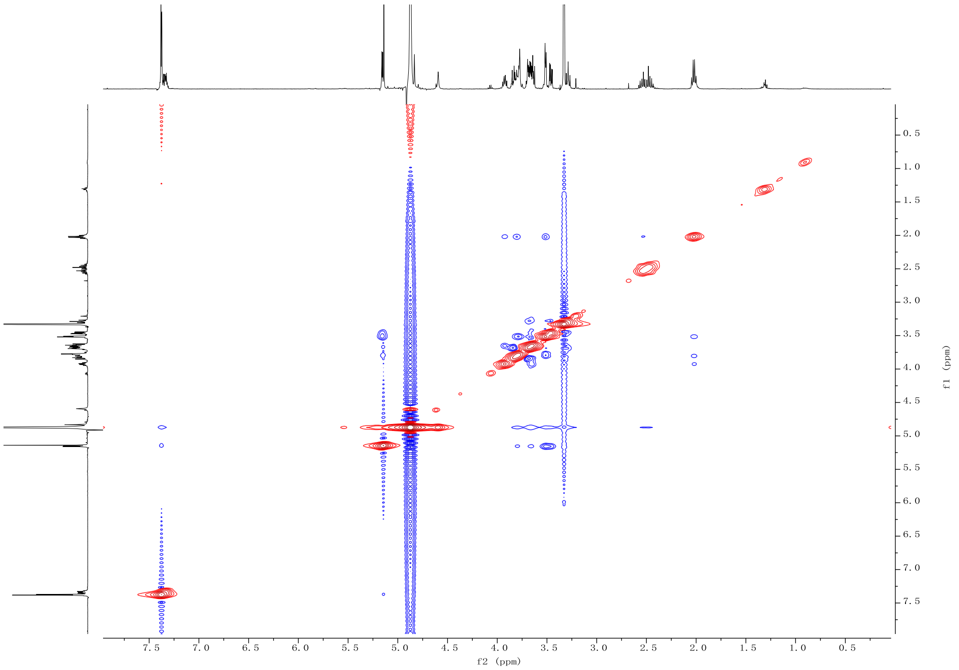

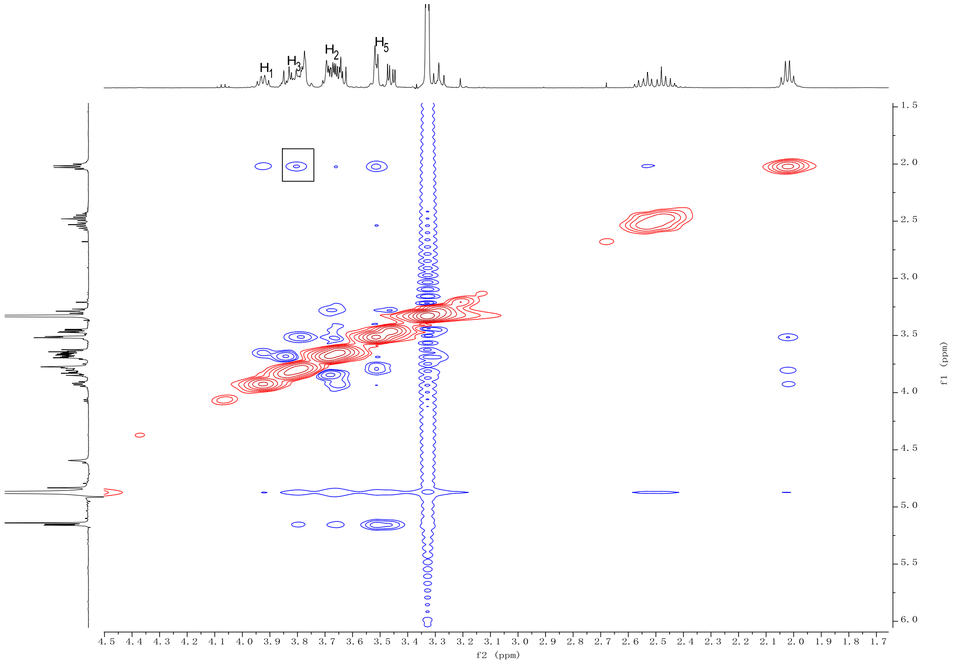

^{1}H NMR spectrum of compound 26

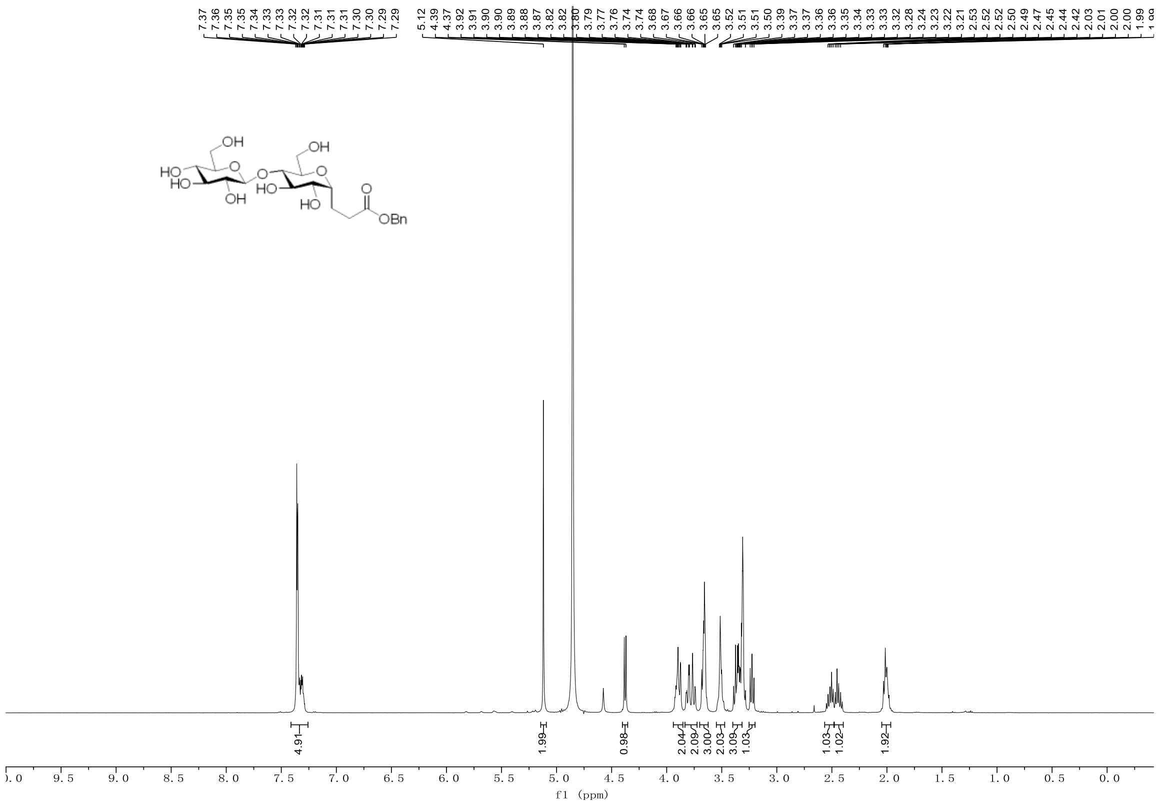

^{13}C NMR spectrum of compound 26

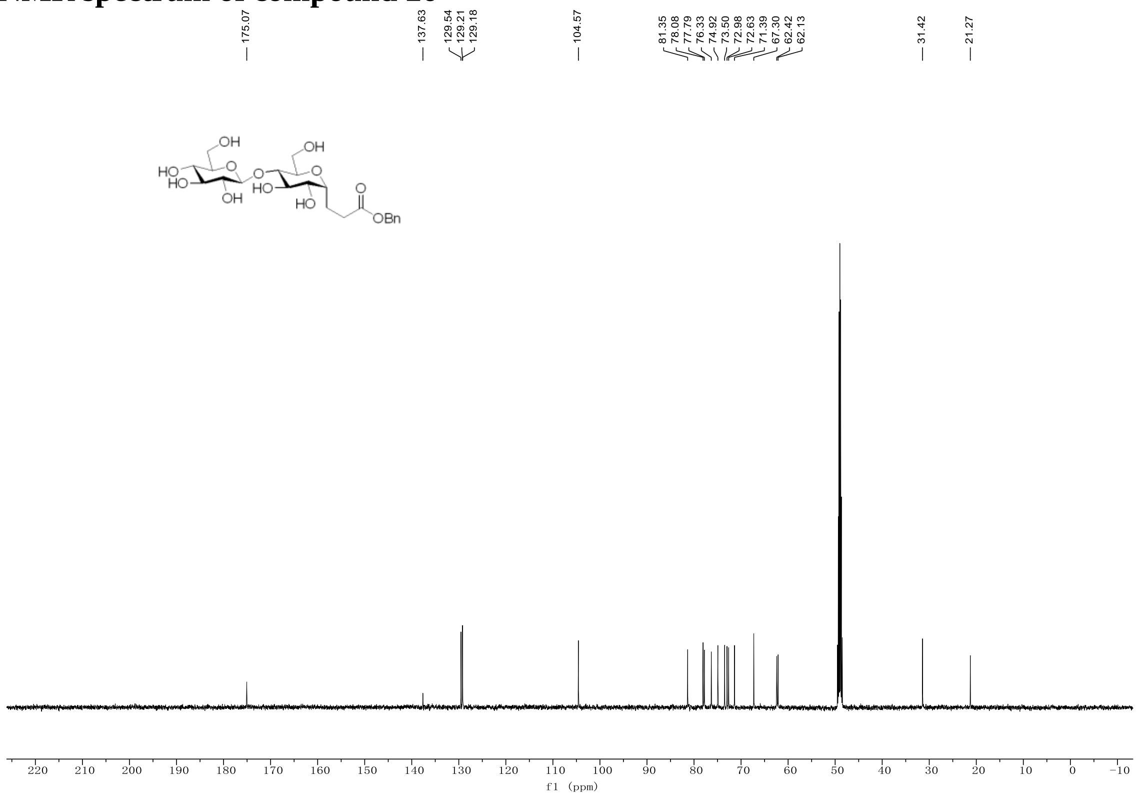

^{1}H NMR spectrum of compound 27

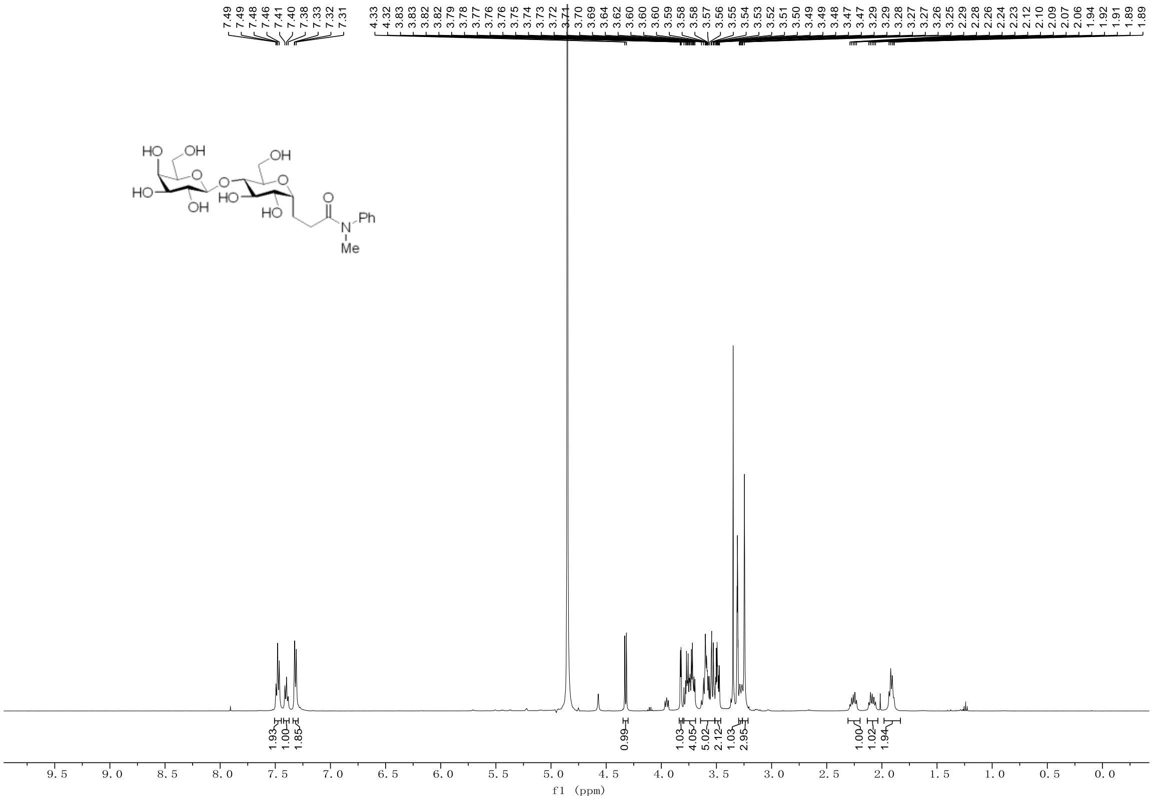

^{13}C NMR spectrum of compound 27

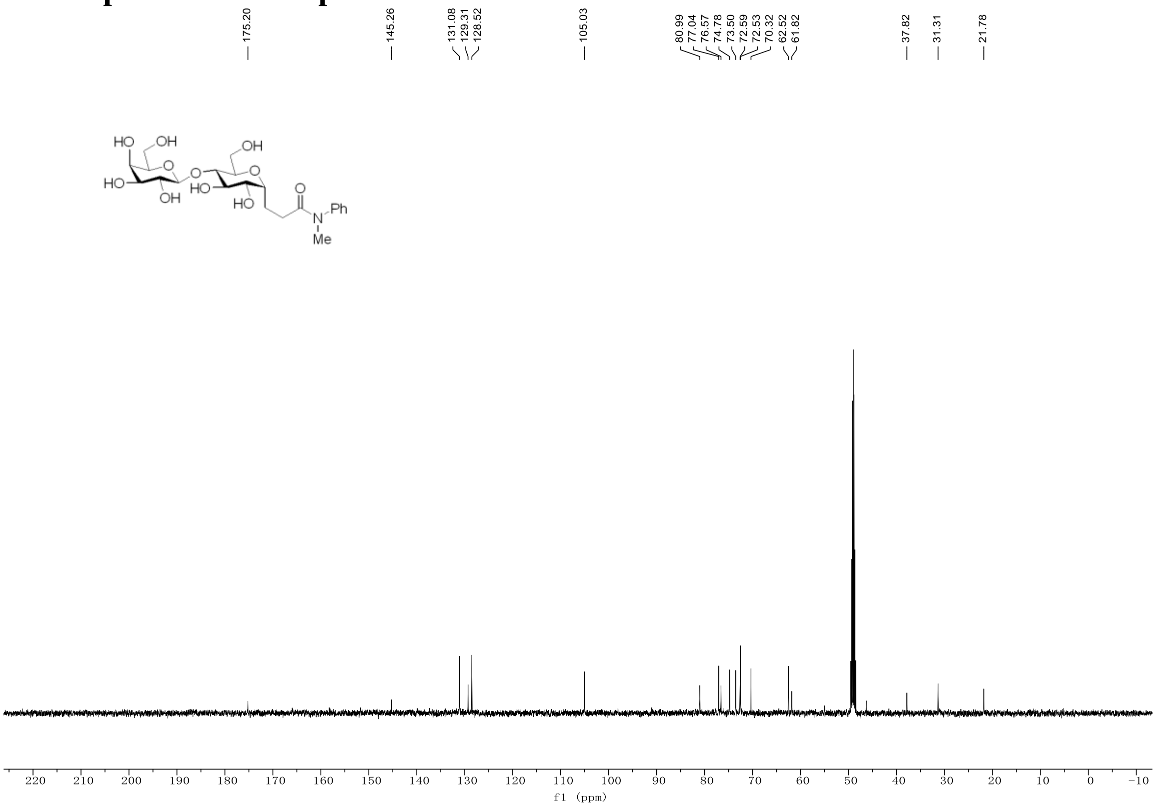

COSY spectrum of compound 27

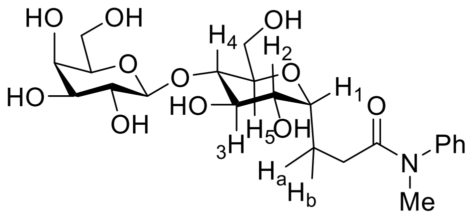

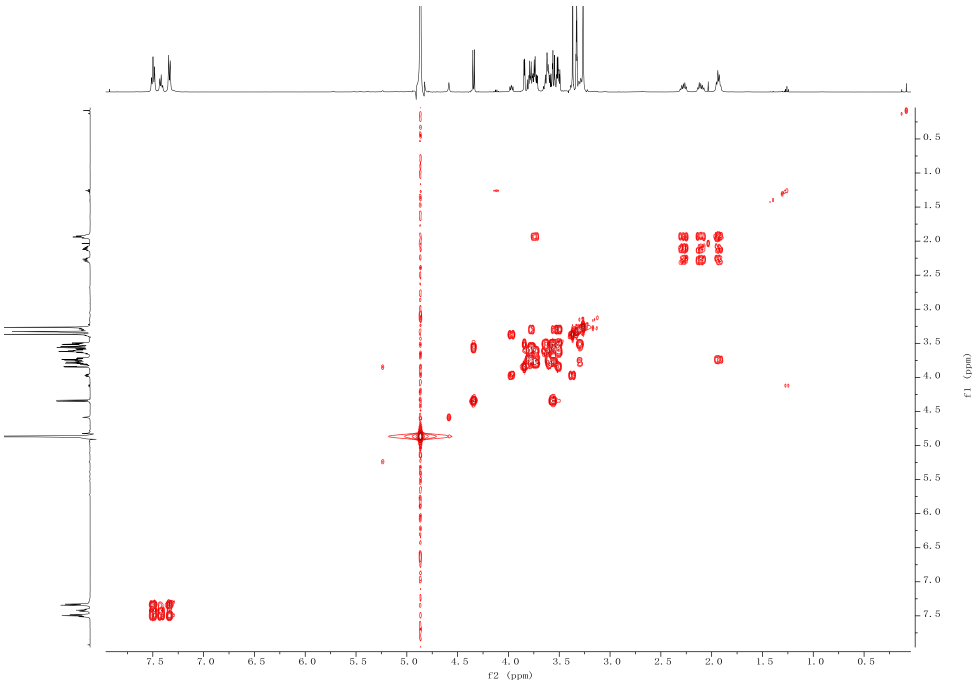

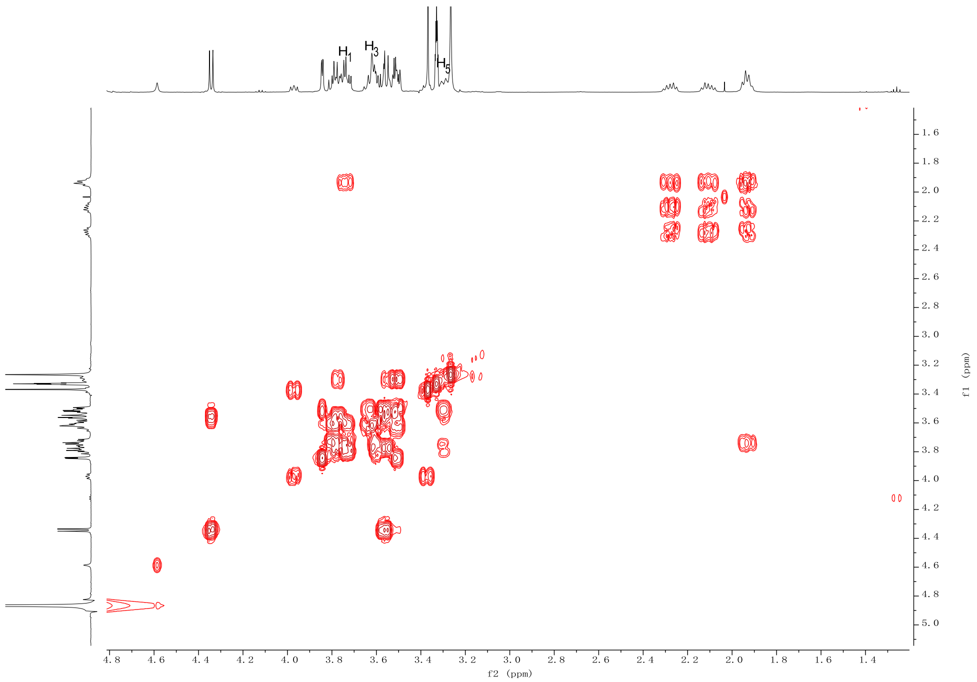

NOE spectrum of compound 27

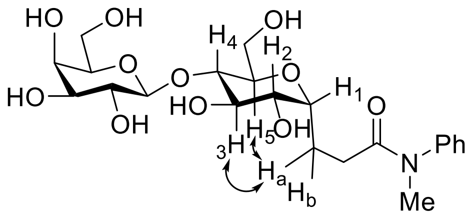

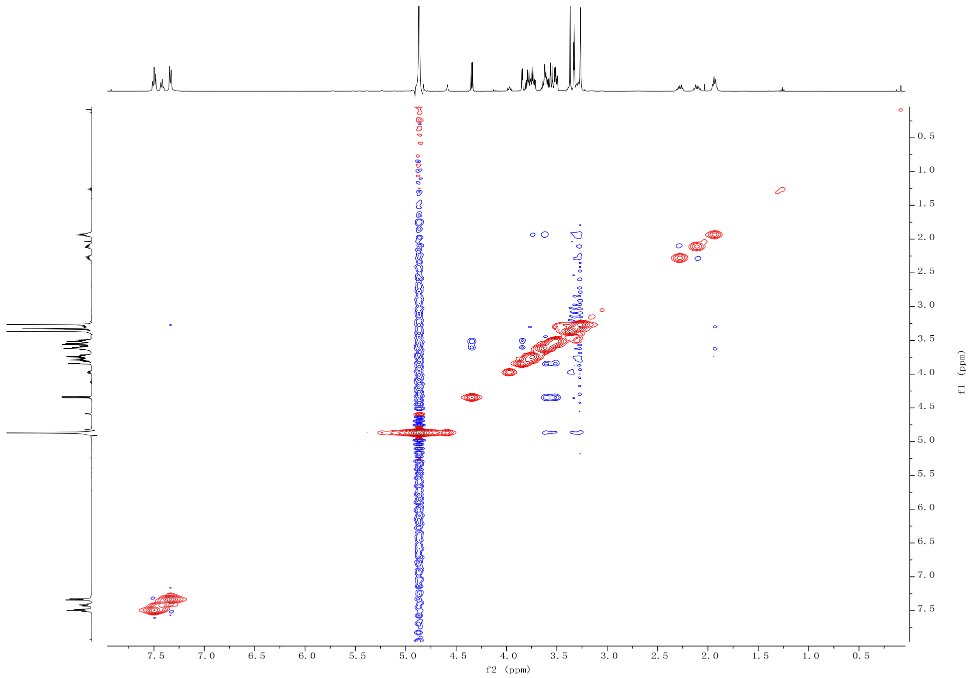

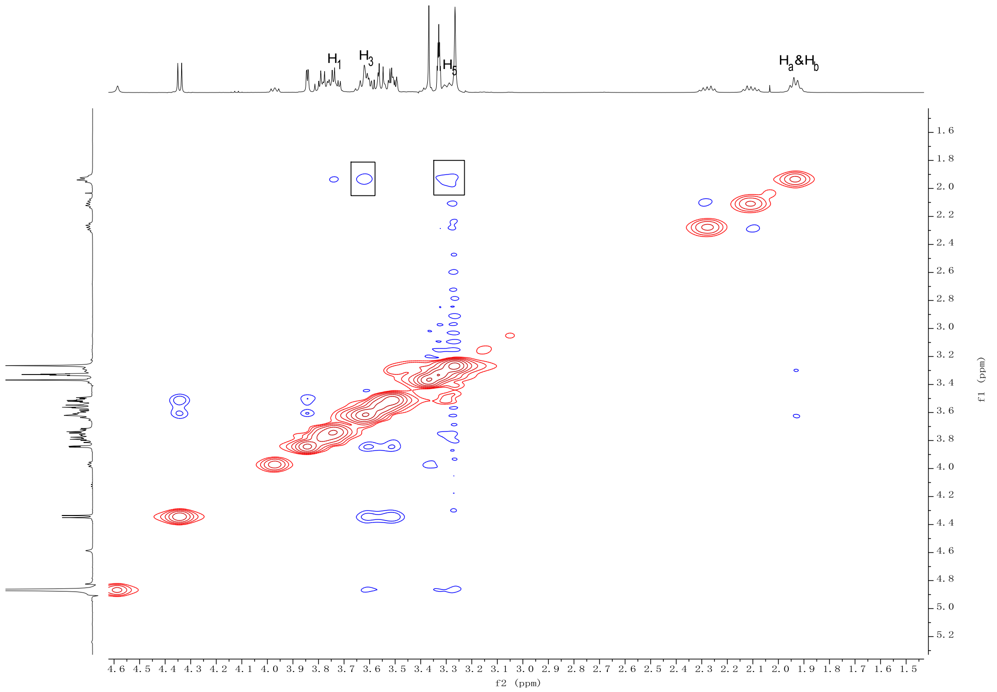

^{1}H NMR spectrum of compound 28

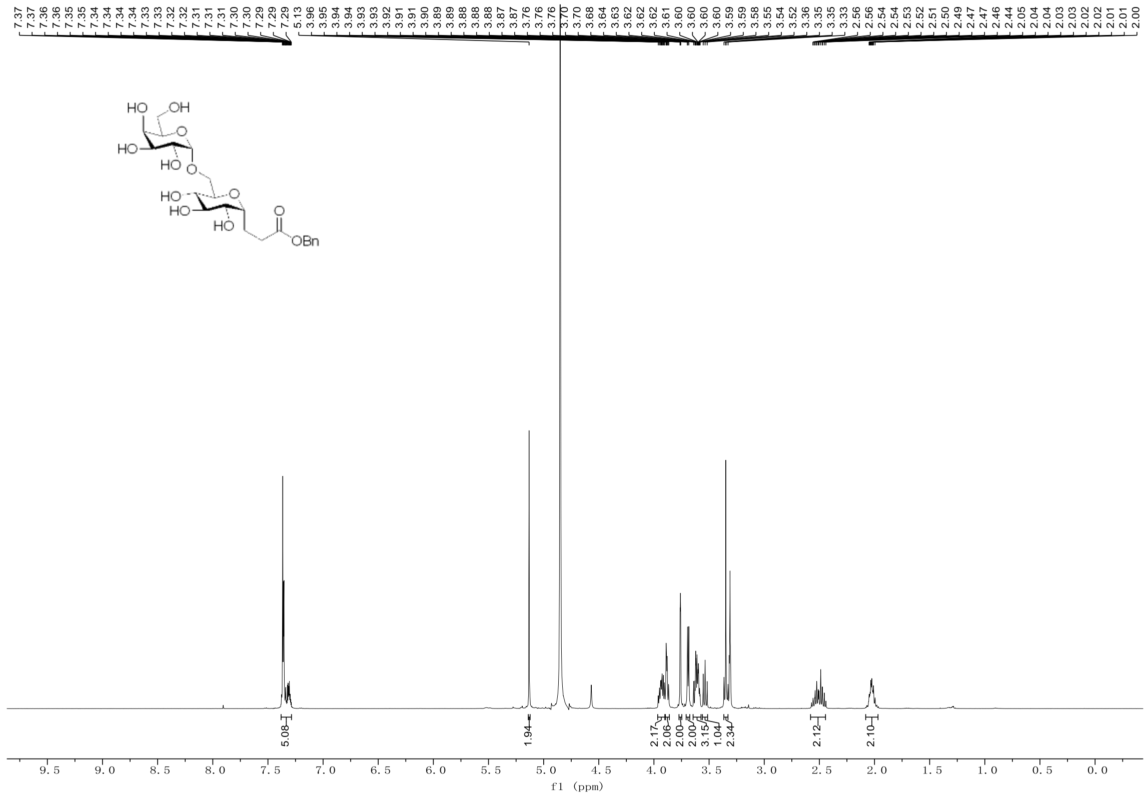

^{13}C NMR spectrum of compound 28

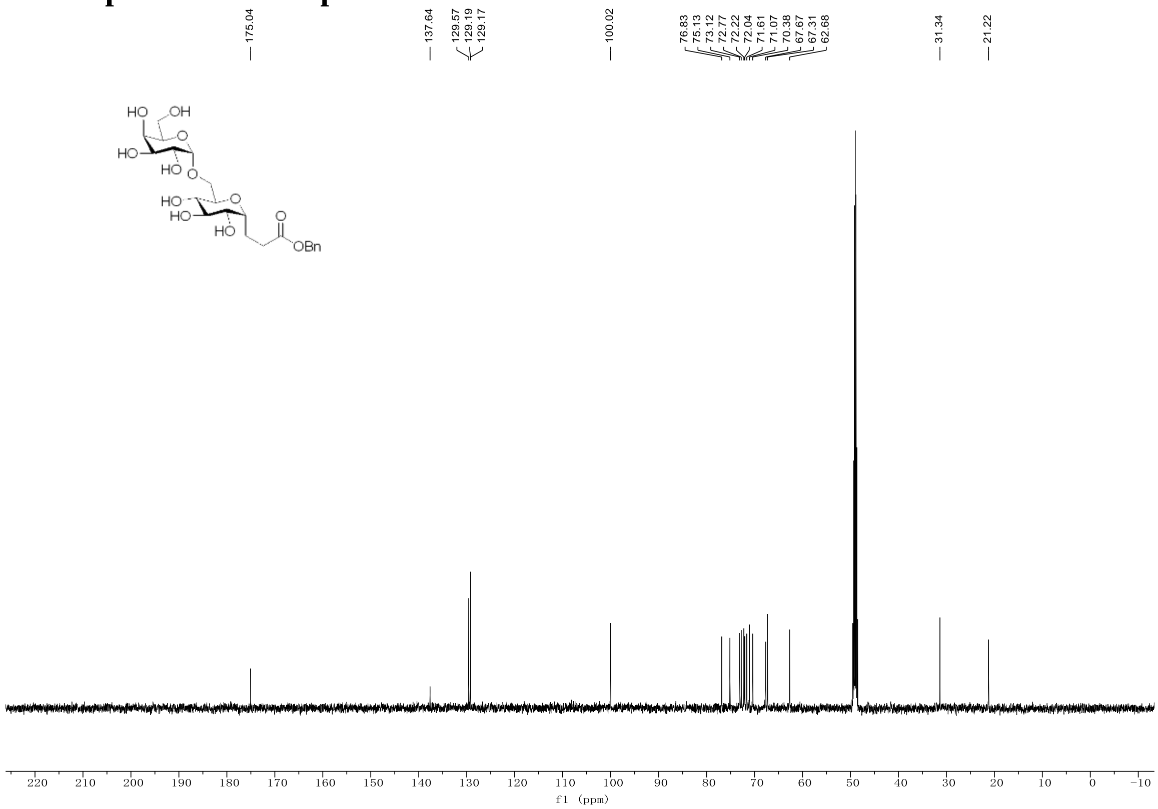

^{1}H NMR spectrum of compound 29

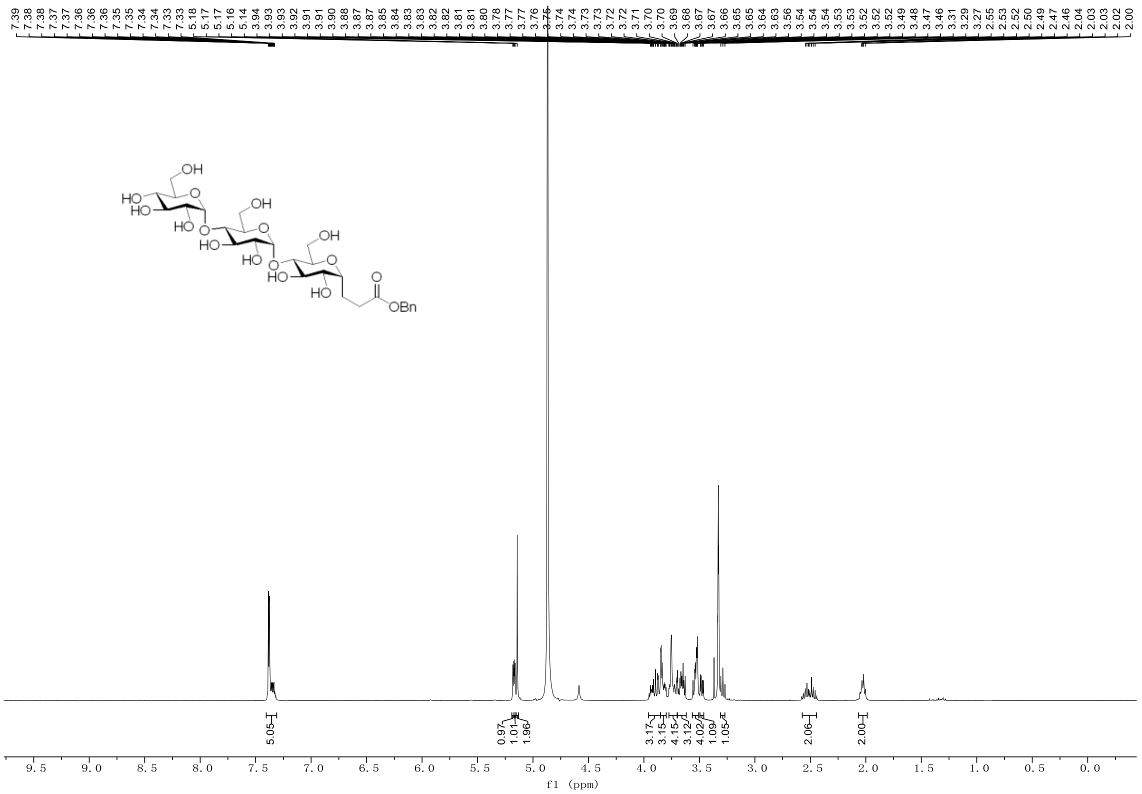

^{13}C NMR spectrum of compound 29

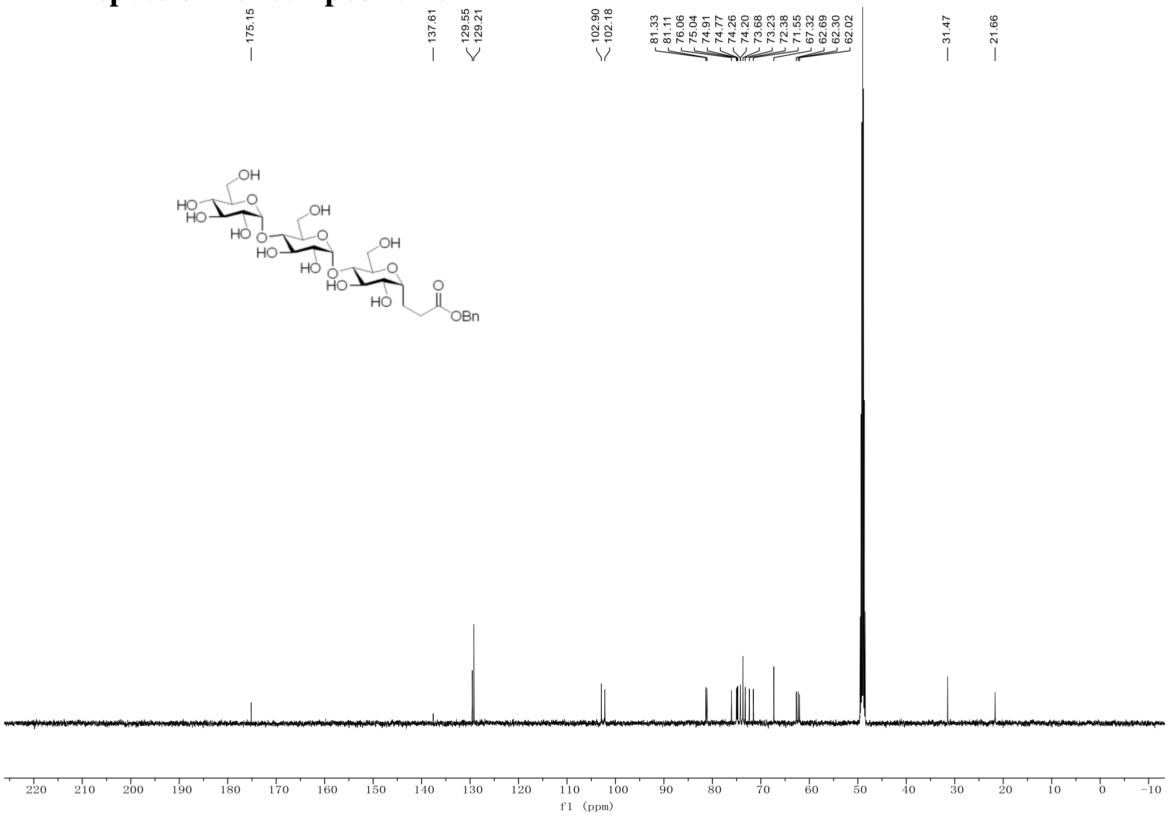

^1H NMR spectrum of compound 30

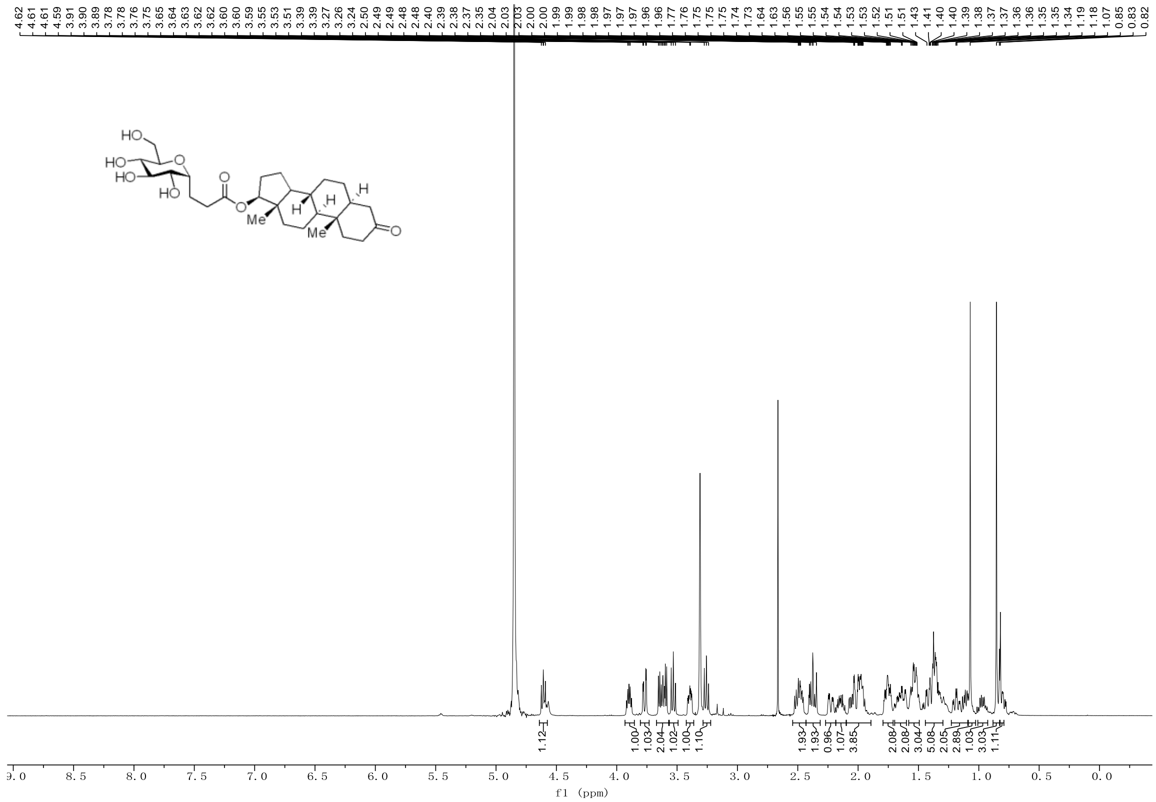

^{13}C NMR spectrum of compound 30

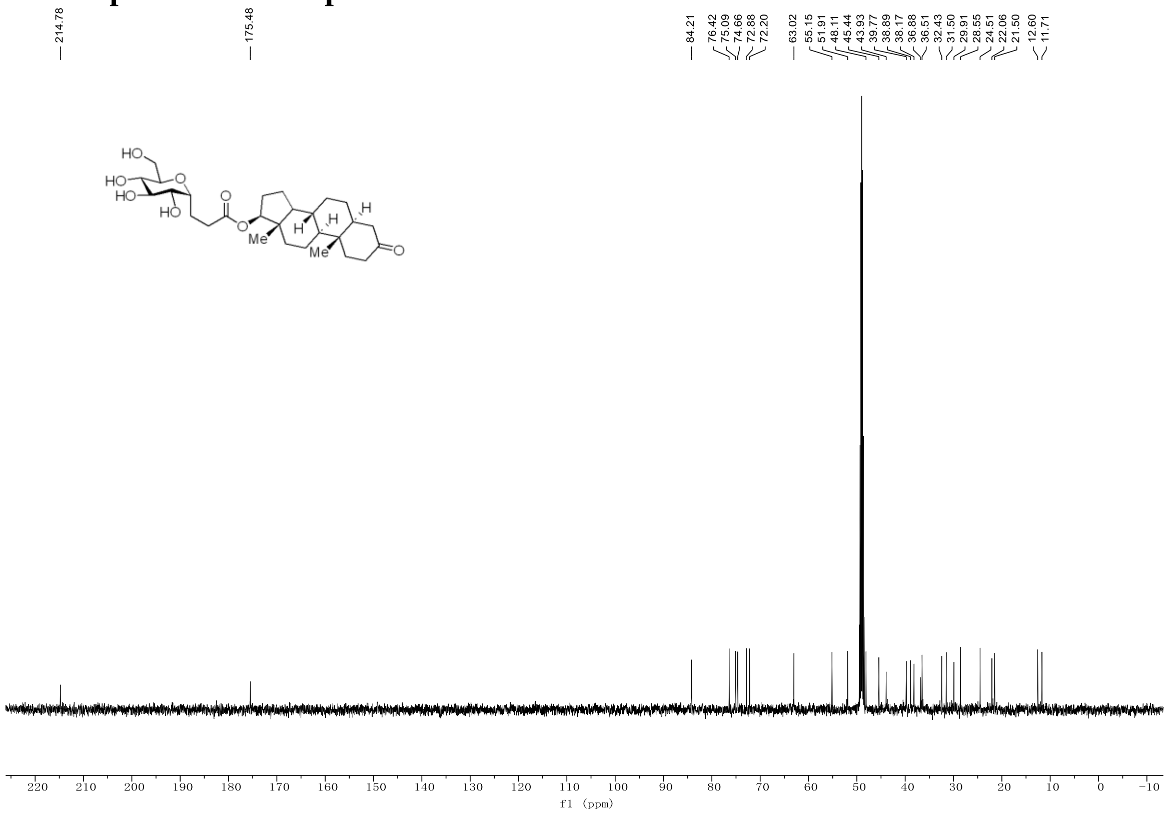

^{1}H NMR spectrum of compound 31

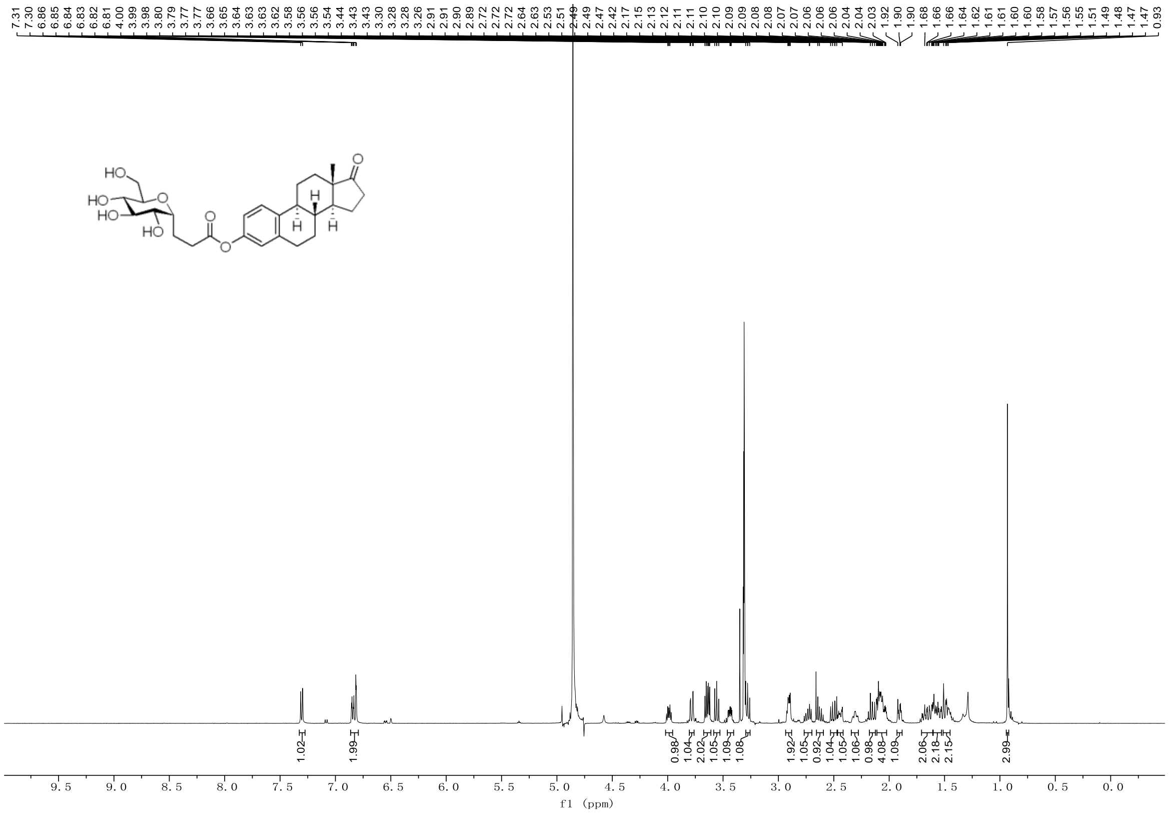

^{13}C NMR spectrum of compound 31

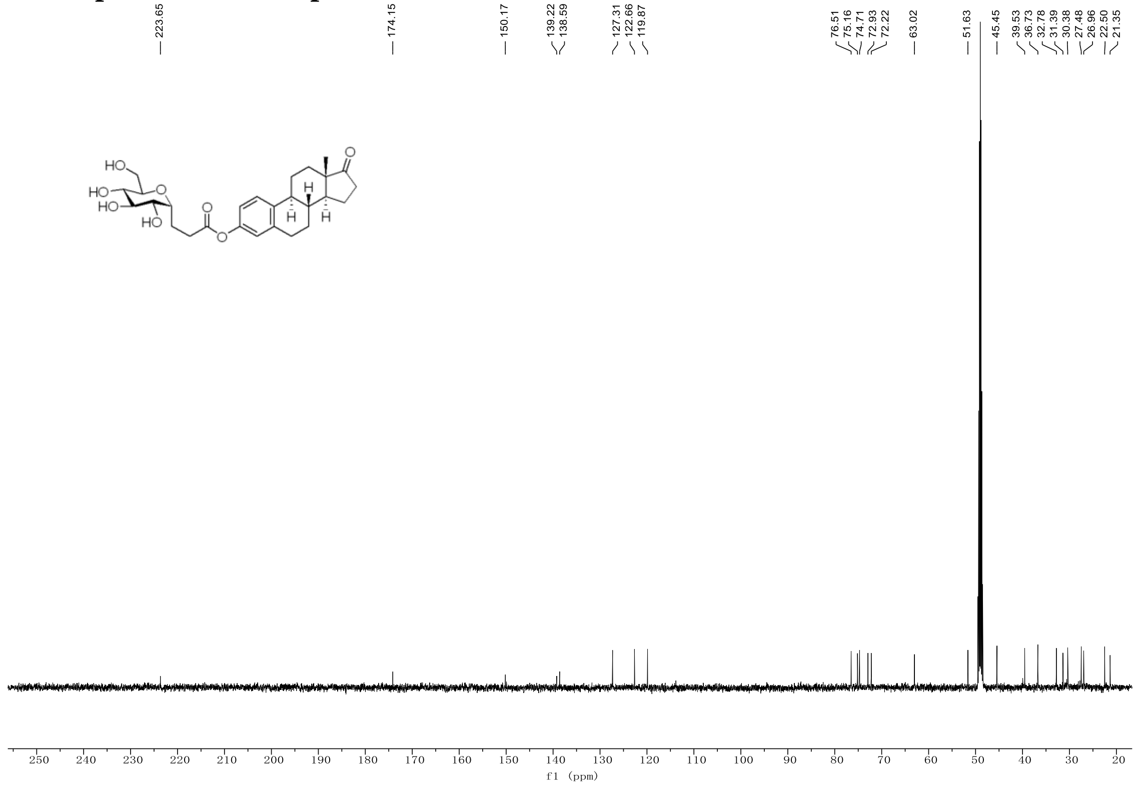

^{1}H NMR spectrum of compound 32

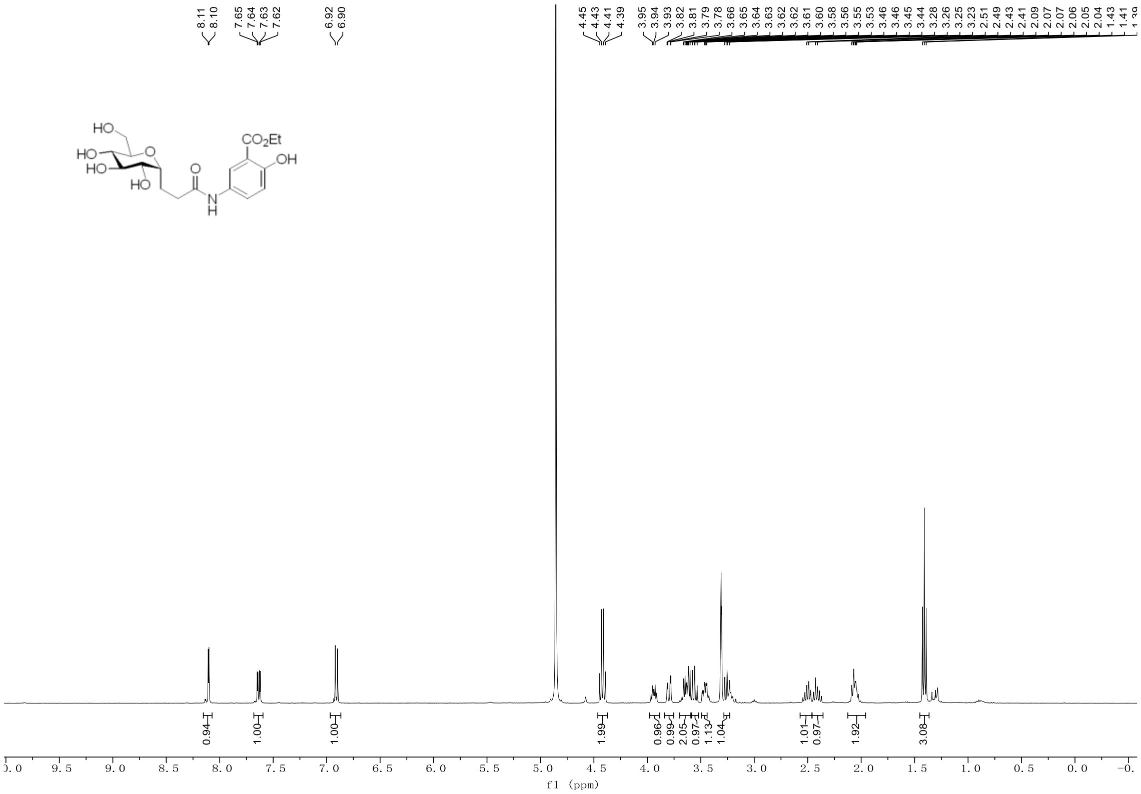

^{13}C NMR spectrum of compound 32

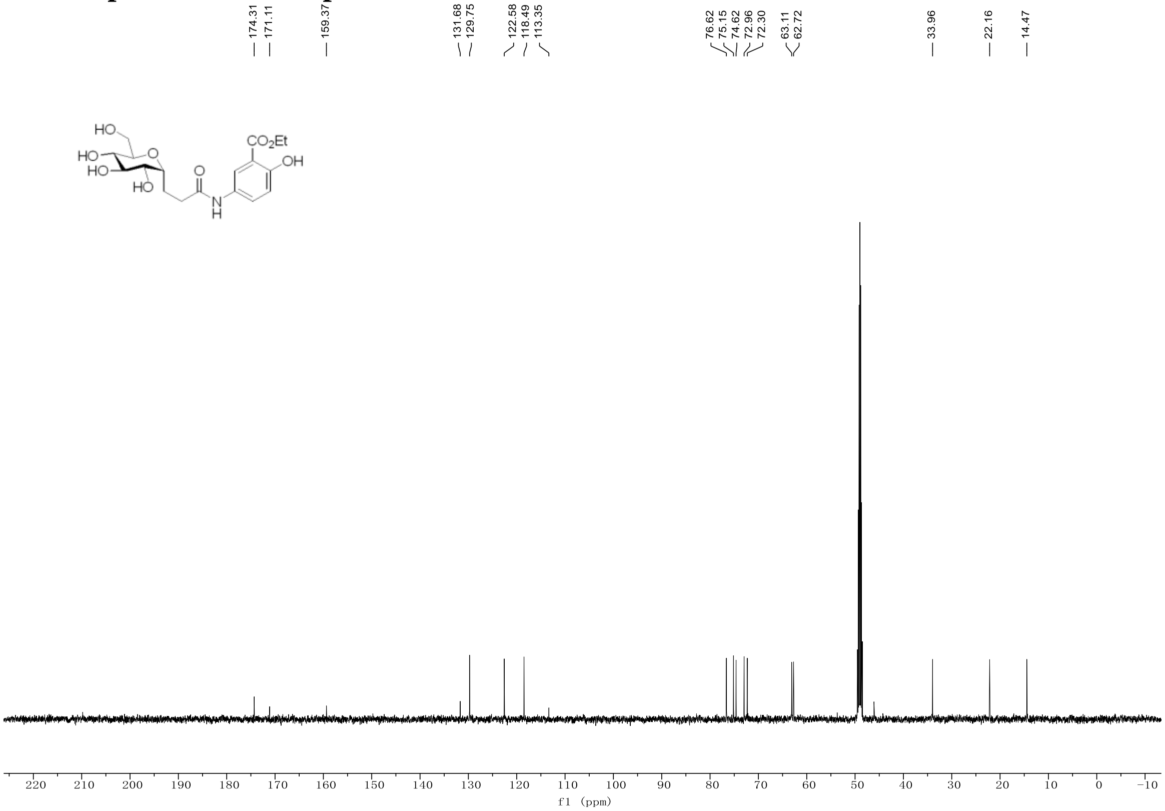

^{1}H NMR spectrum of compound 33

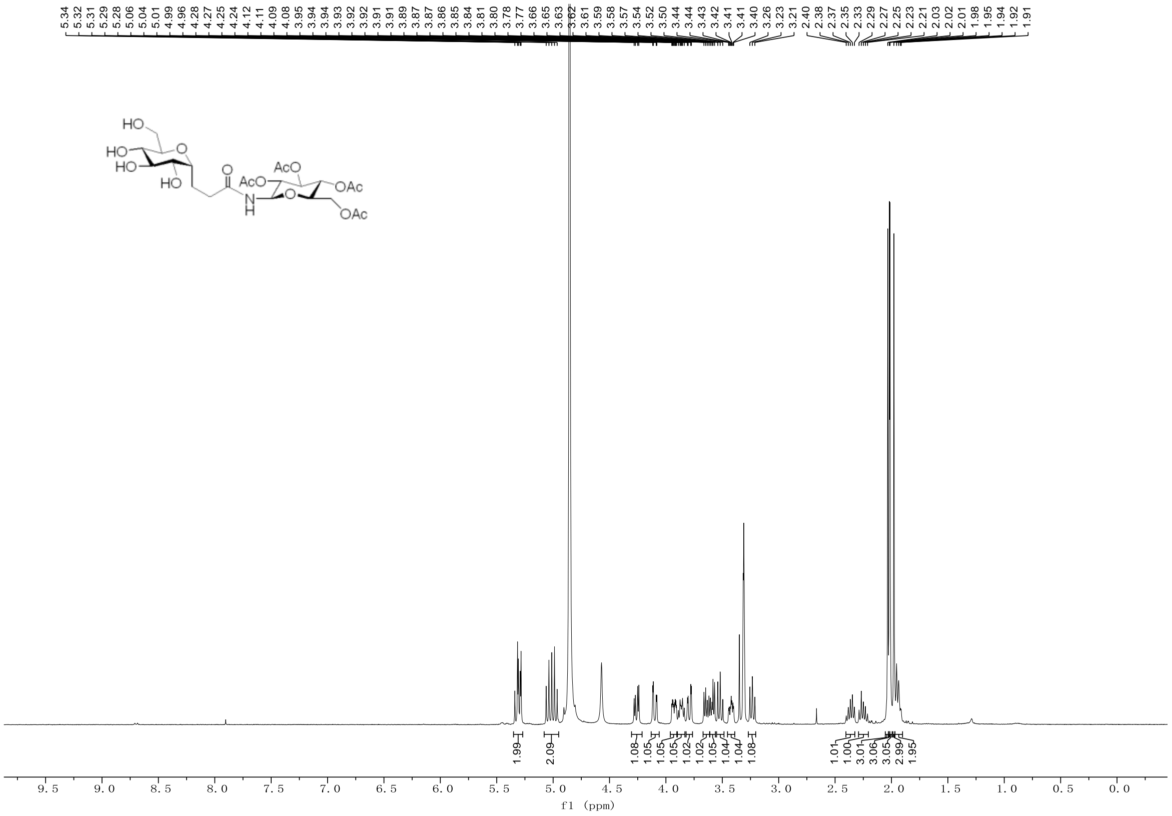

^{13}C NMR spectrum of compound 33

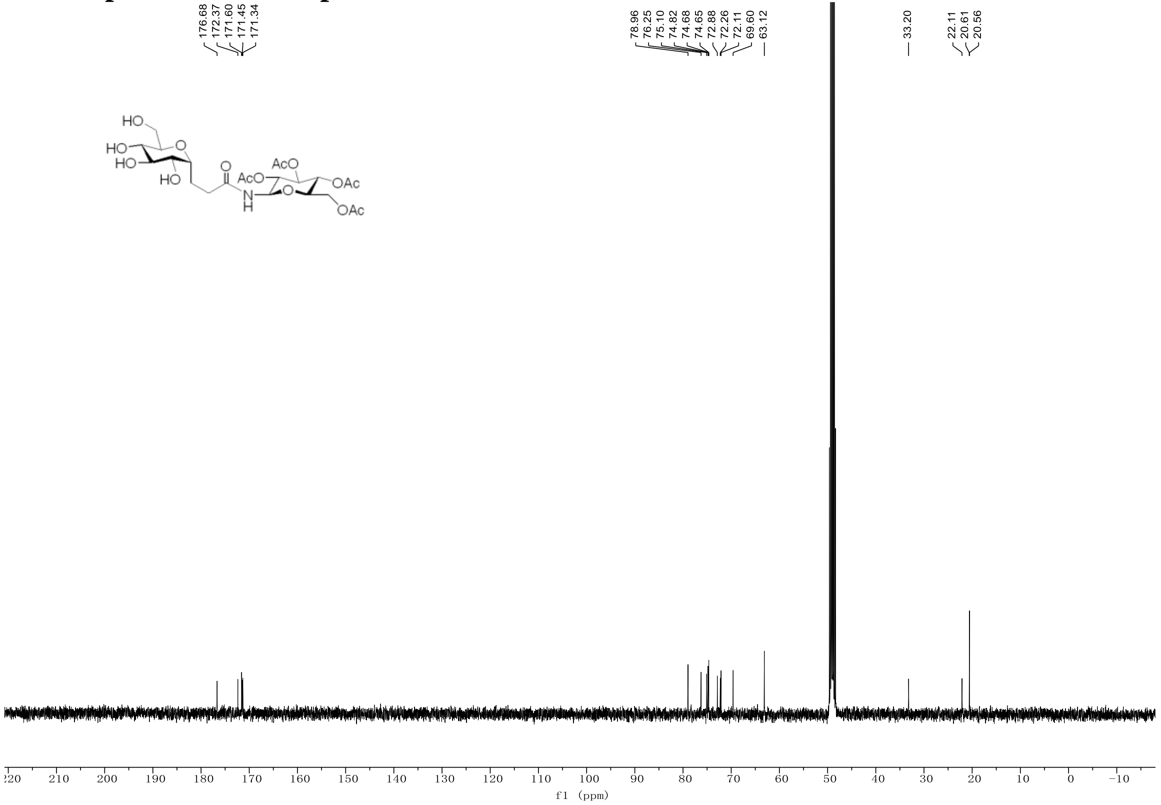

^{1}H NMR spectrum of compound 34

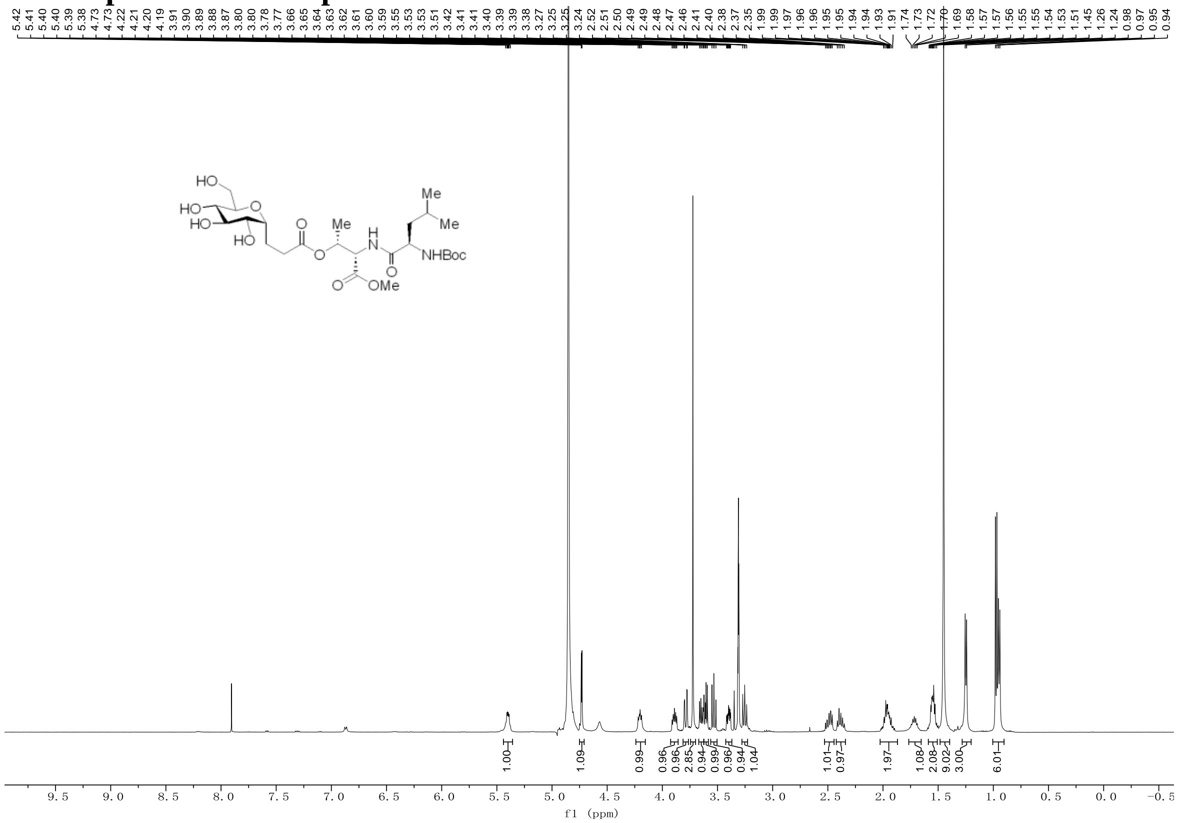

^{13}C NMR spectrum of compound 34

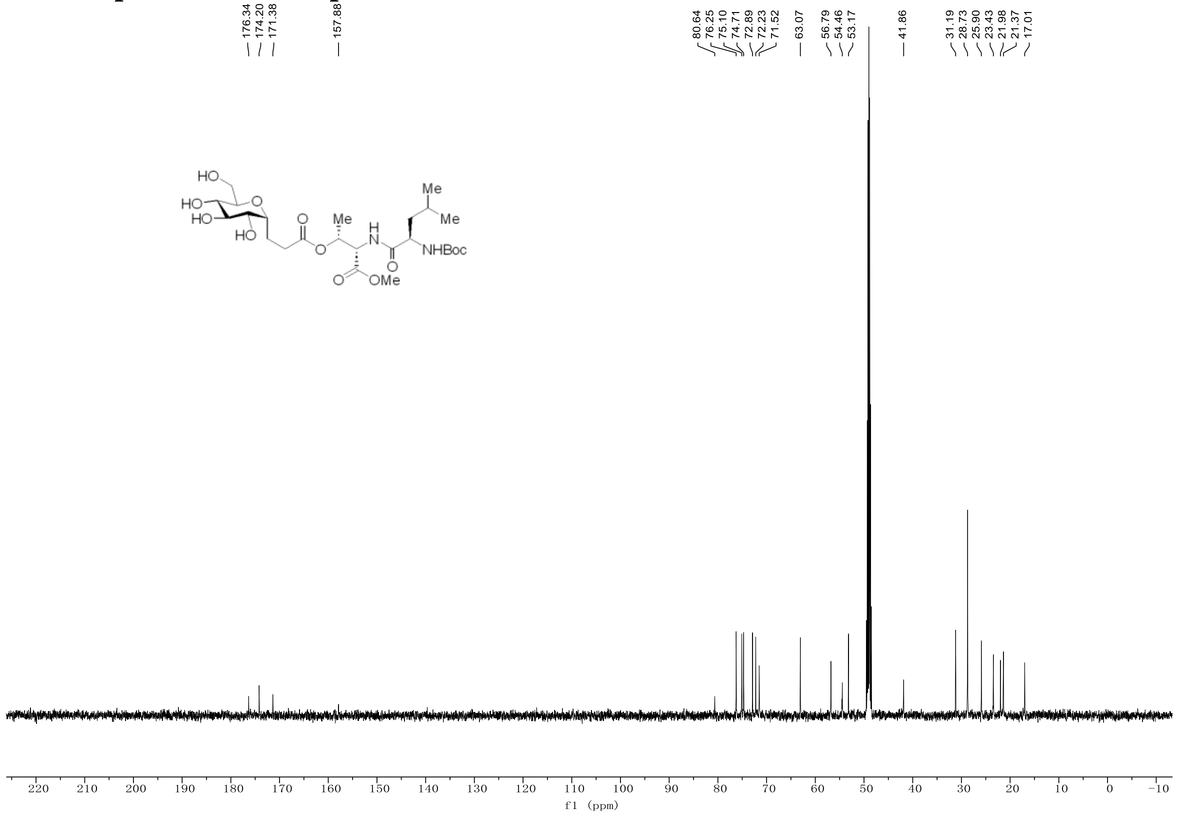

^{1}H NMR spectrum of compound 35

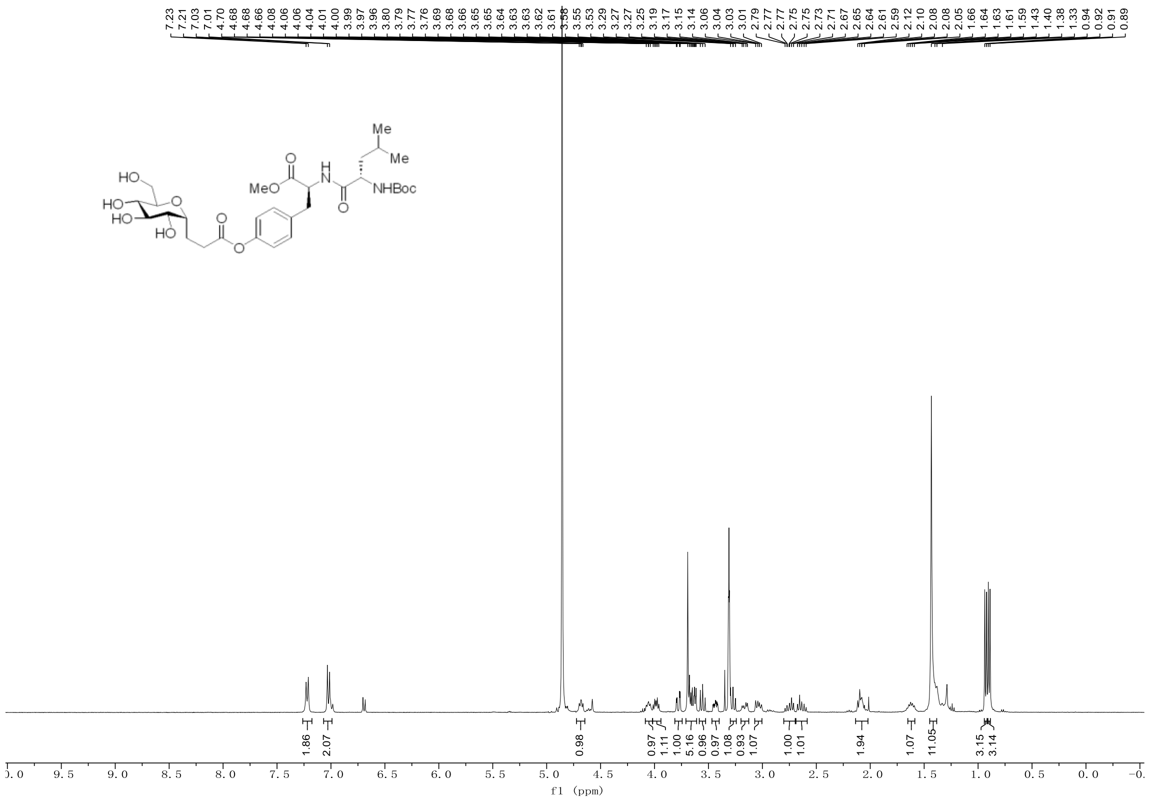

^{13}C NMR spectrum of compound 35

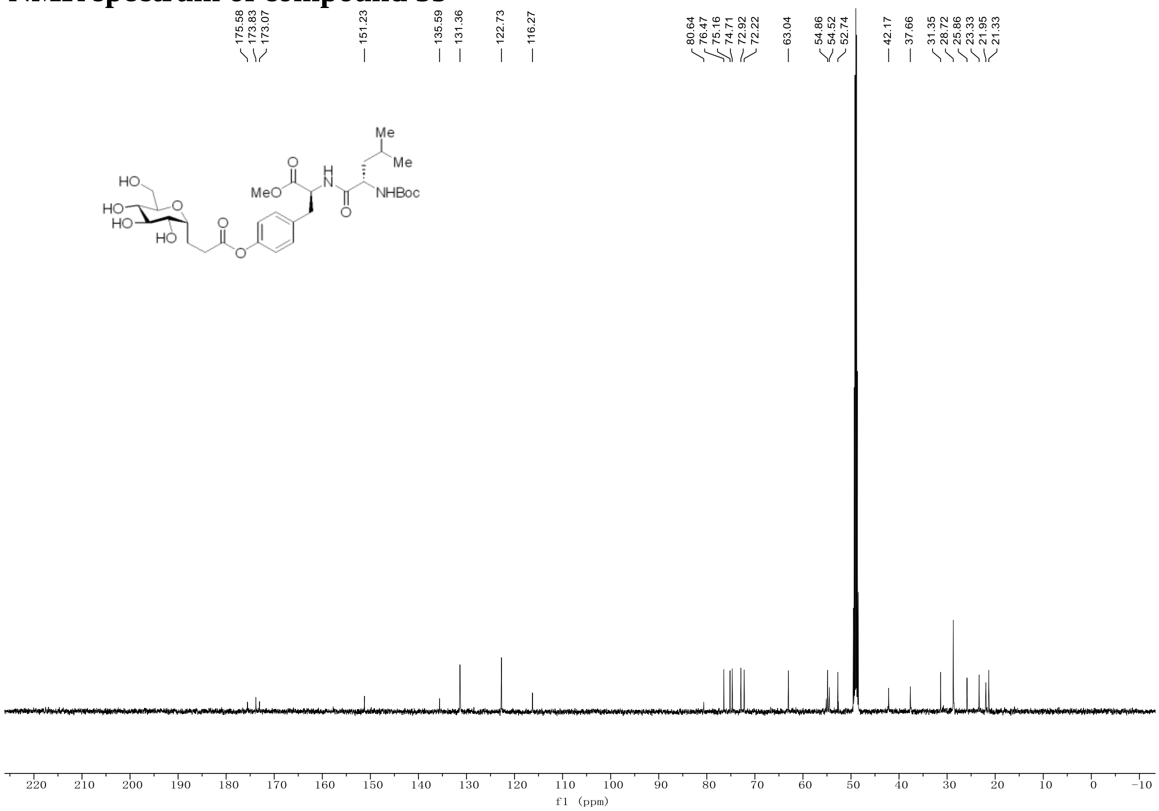

^{1}H NMR spectrum of compound 36

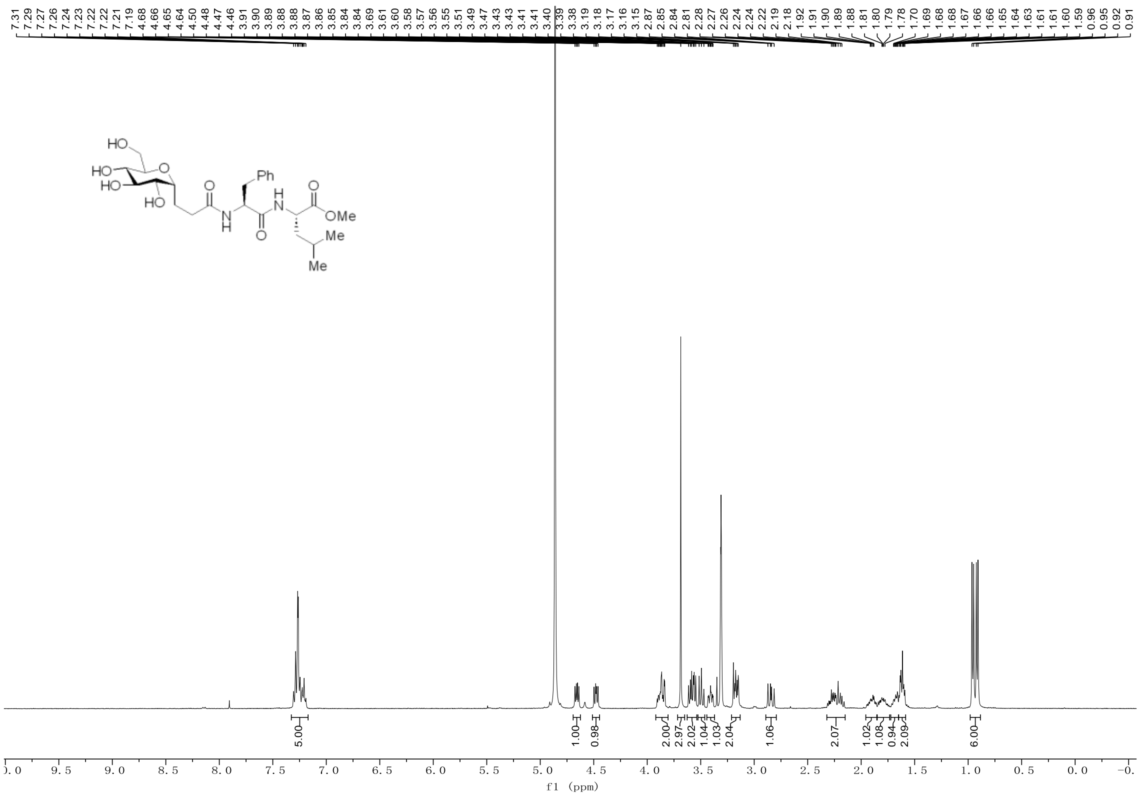

^{13}C NMR spectrum of compound 36

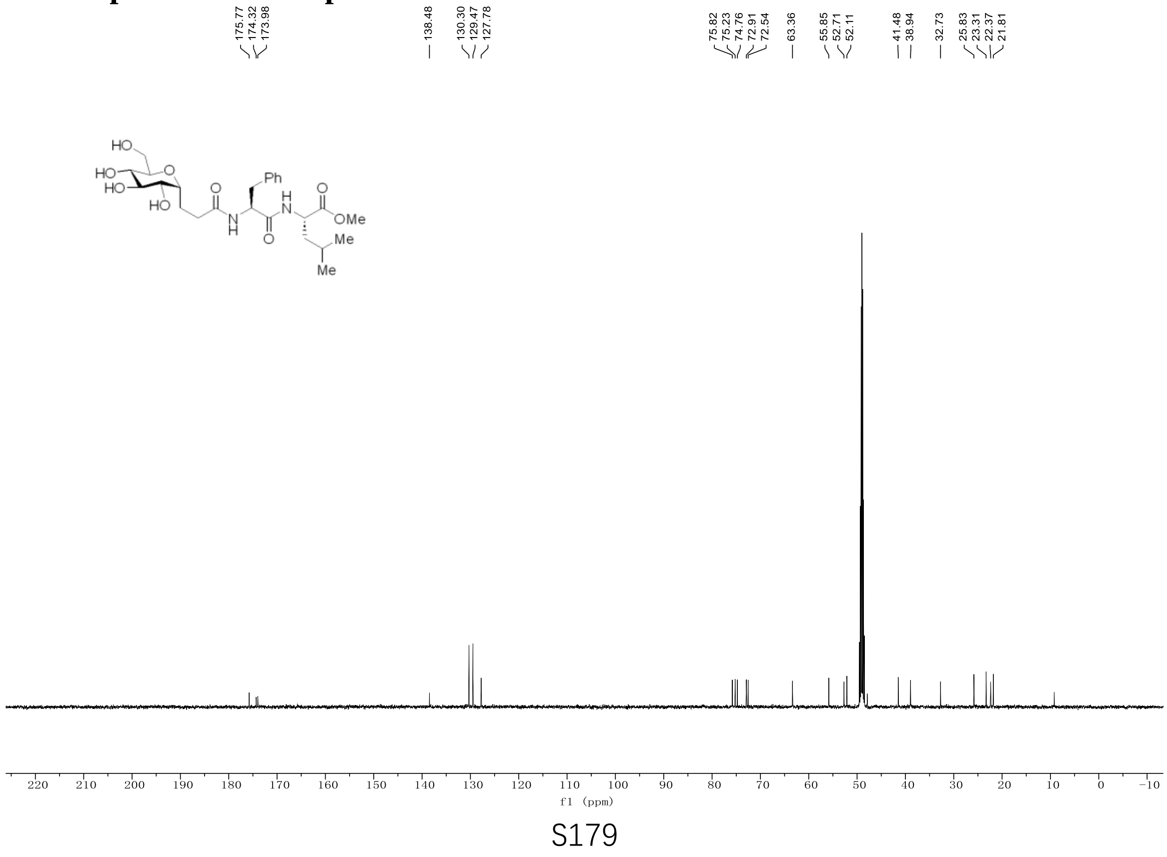

^{1}H NMR spectrum of compound 37

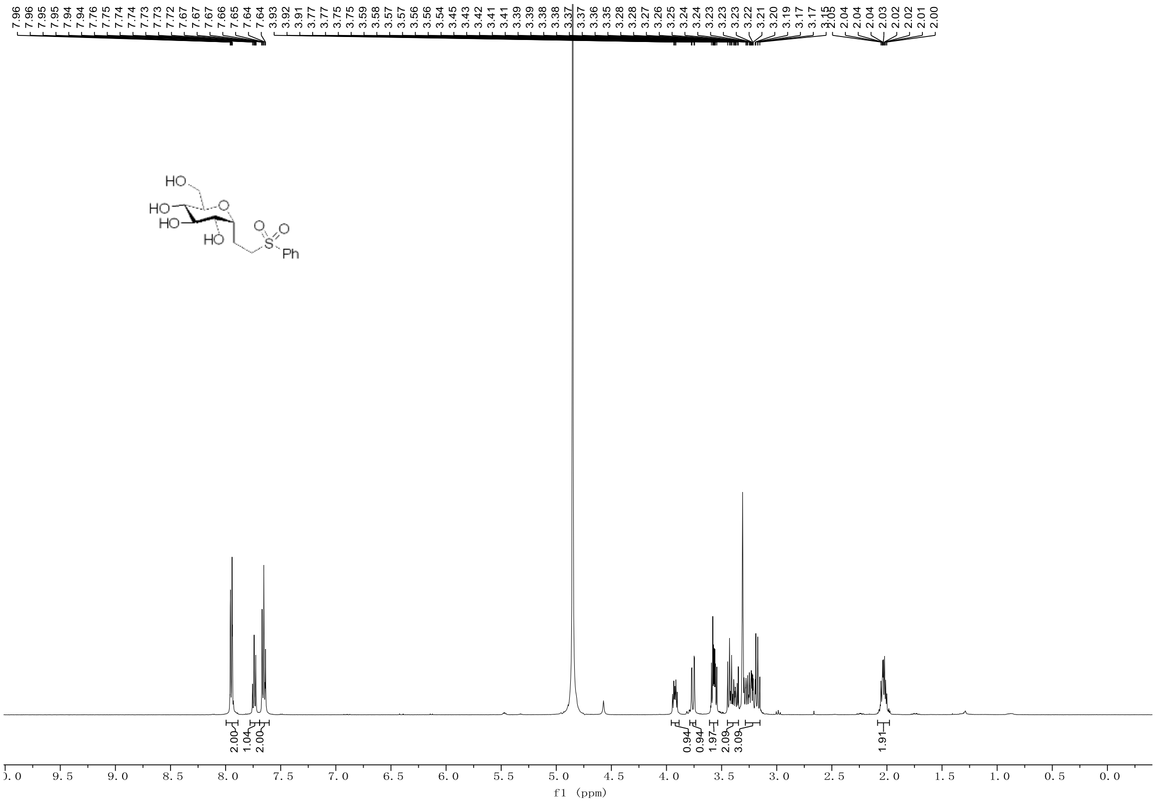

^{13}C NMR spectrum of compound 37

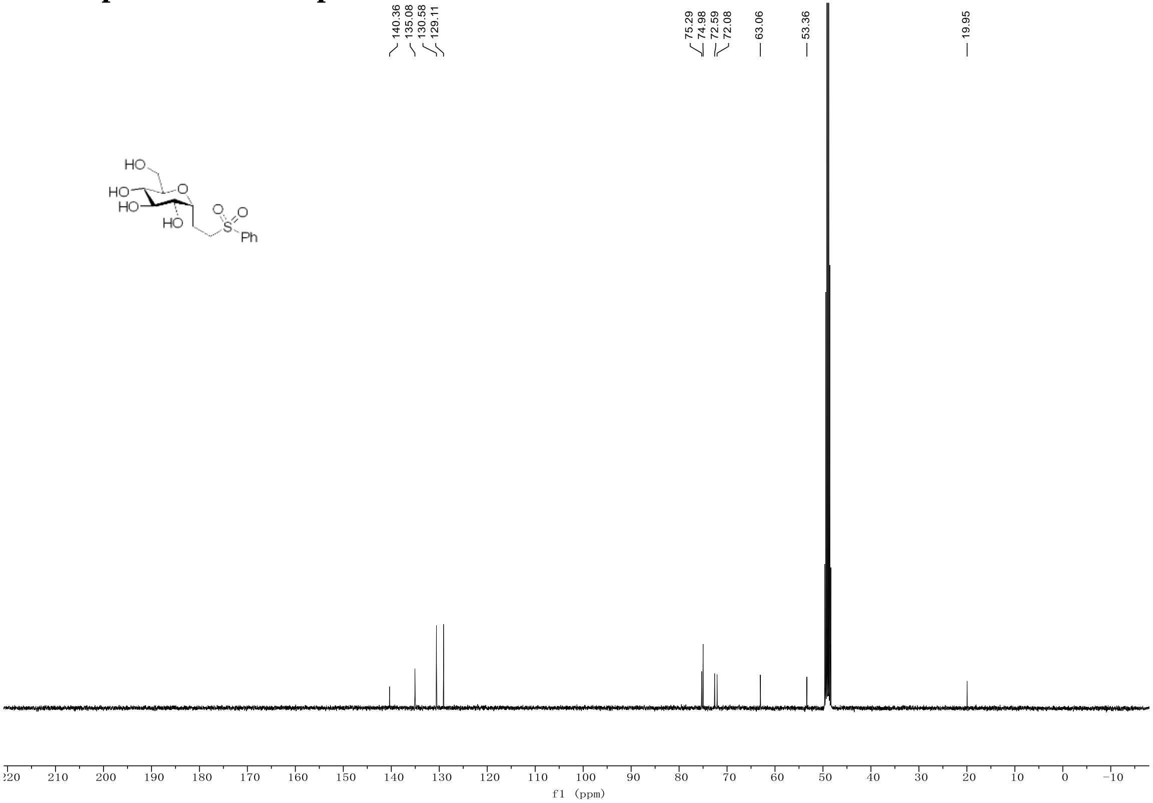

^{1}H NMR spectrum of compound 38

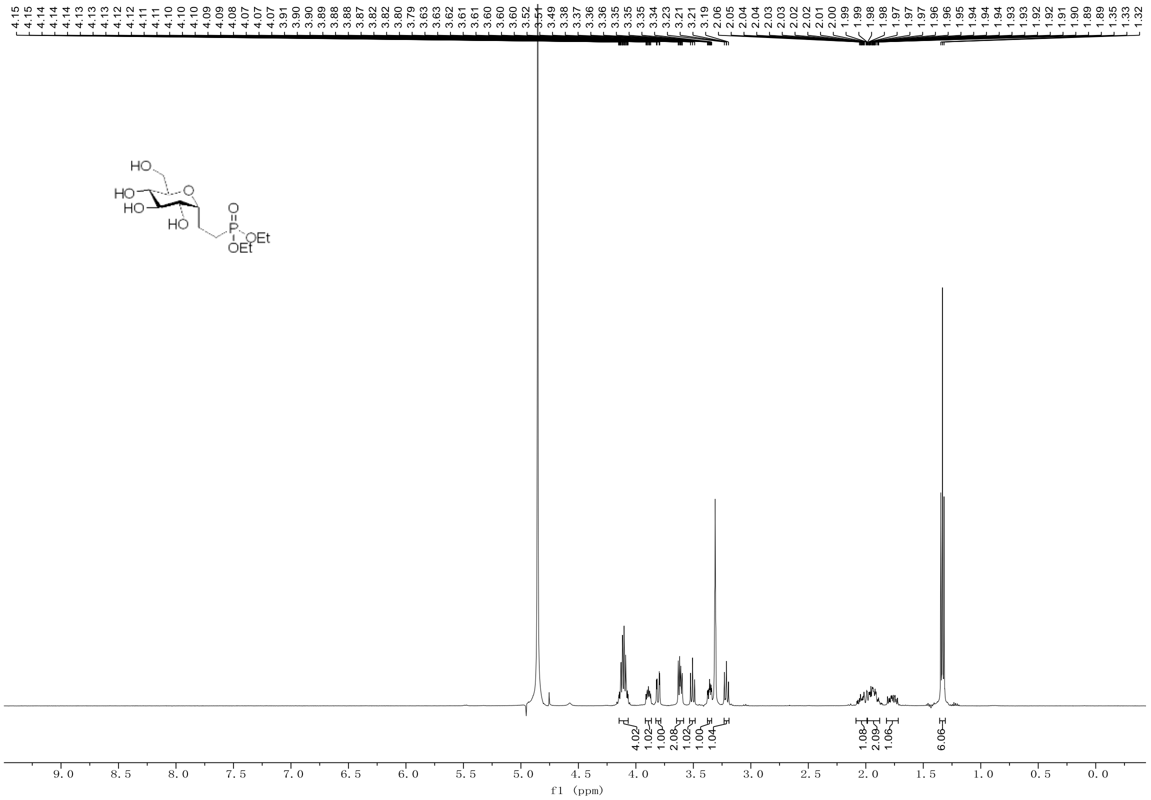

^{13}C NMR spectrum of compound 38

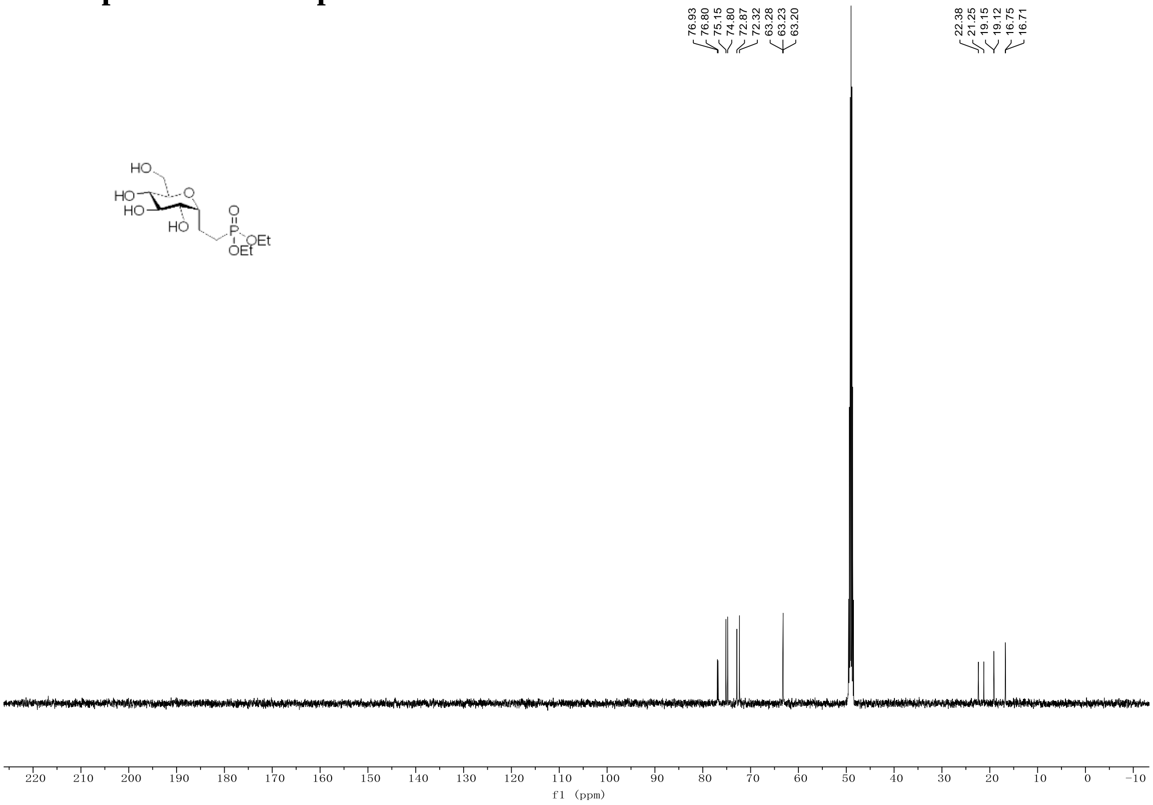

^{1}H NMR spectrum of compound 39

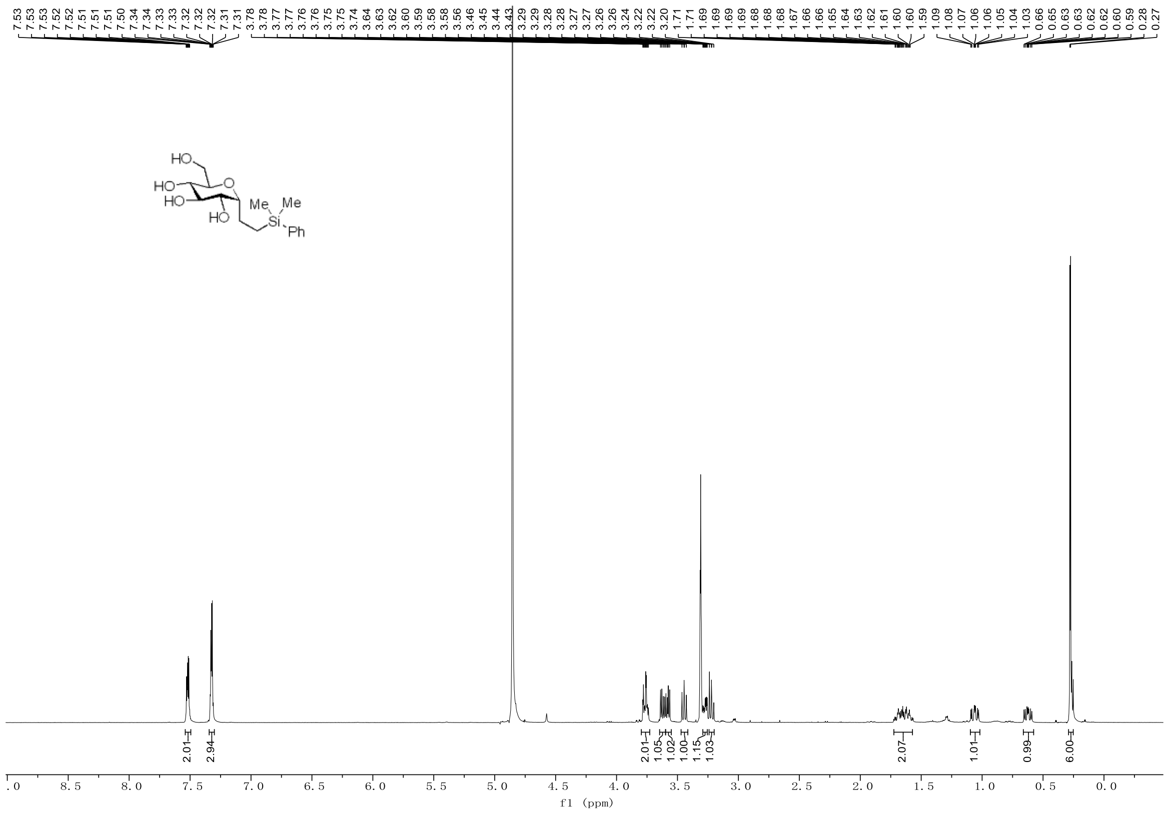

^{13}C NMR spectrum of compound 39

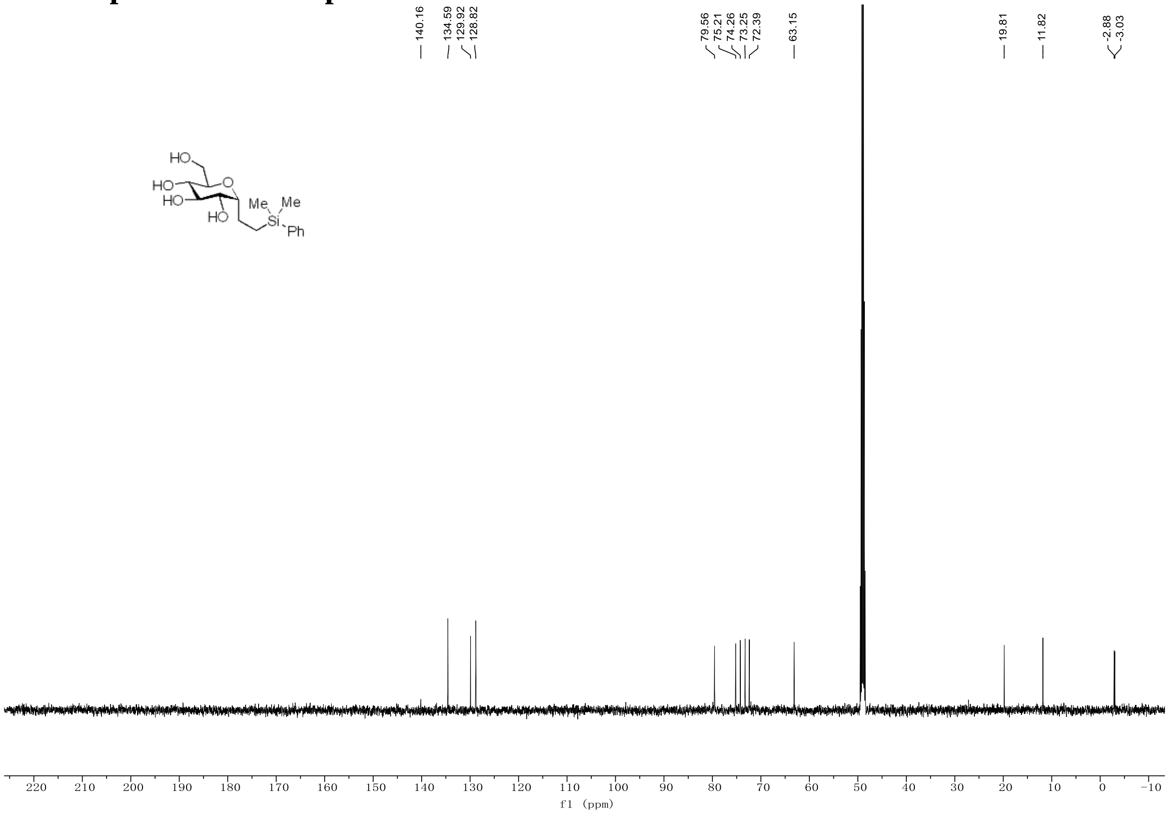

^{1}H NMR spectrum of compound 40

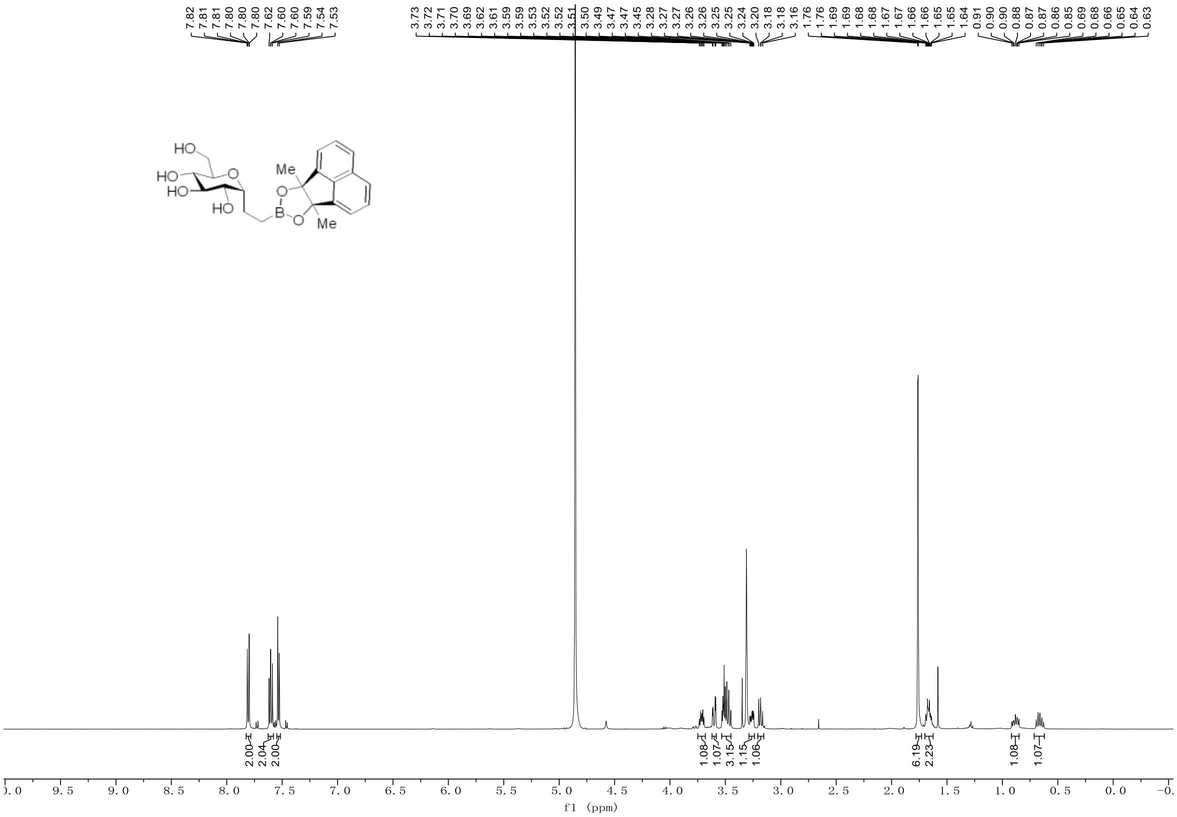

^{13}C NMR spectrum of compound 40

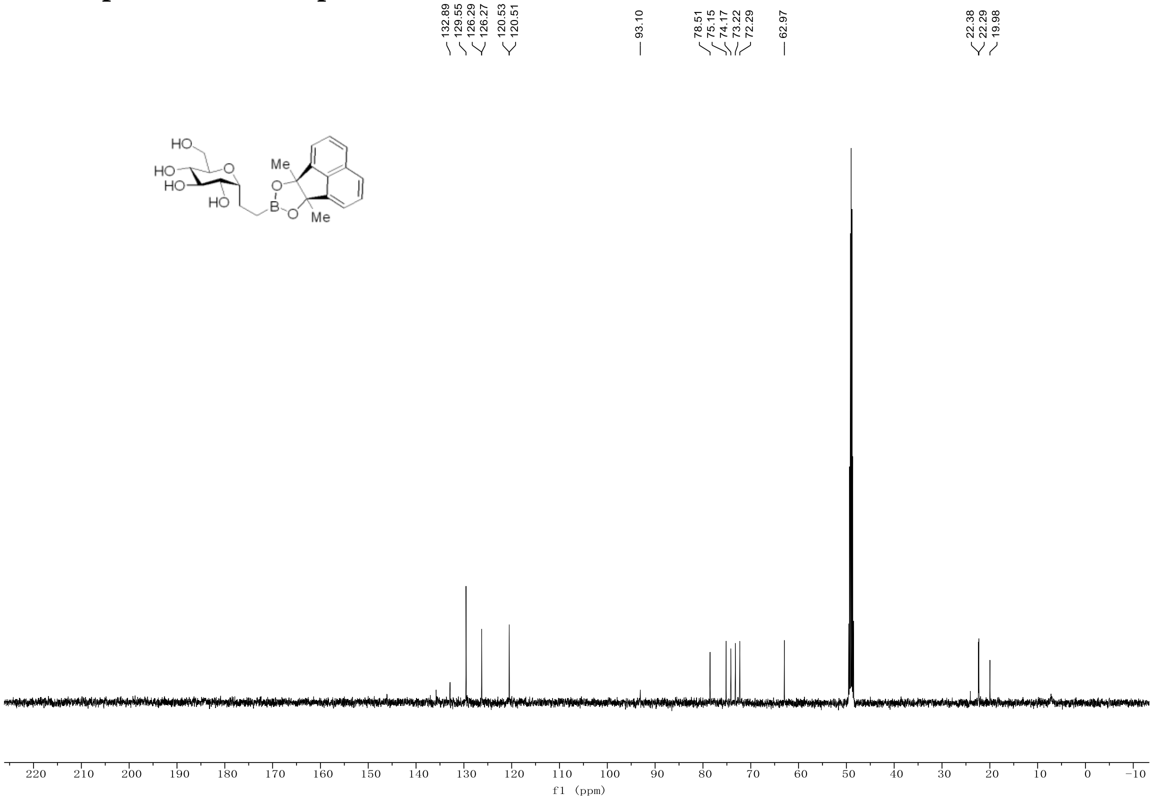

^{1}H NMR spectrum of compound 41

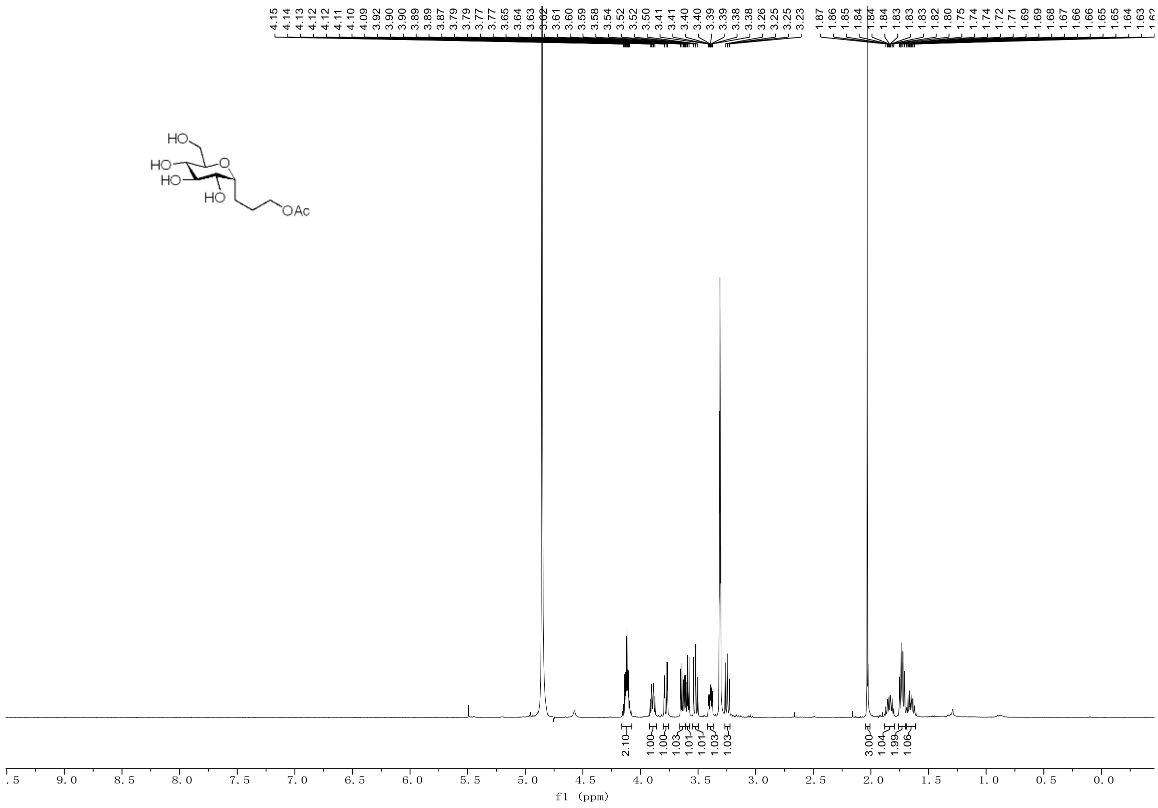

^{13}C NMR spectrum of compound 41

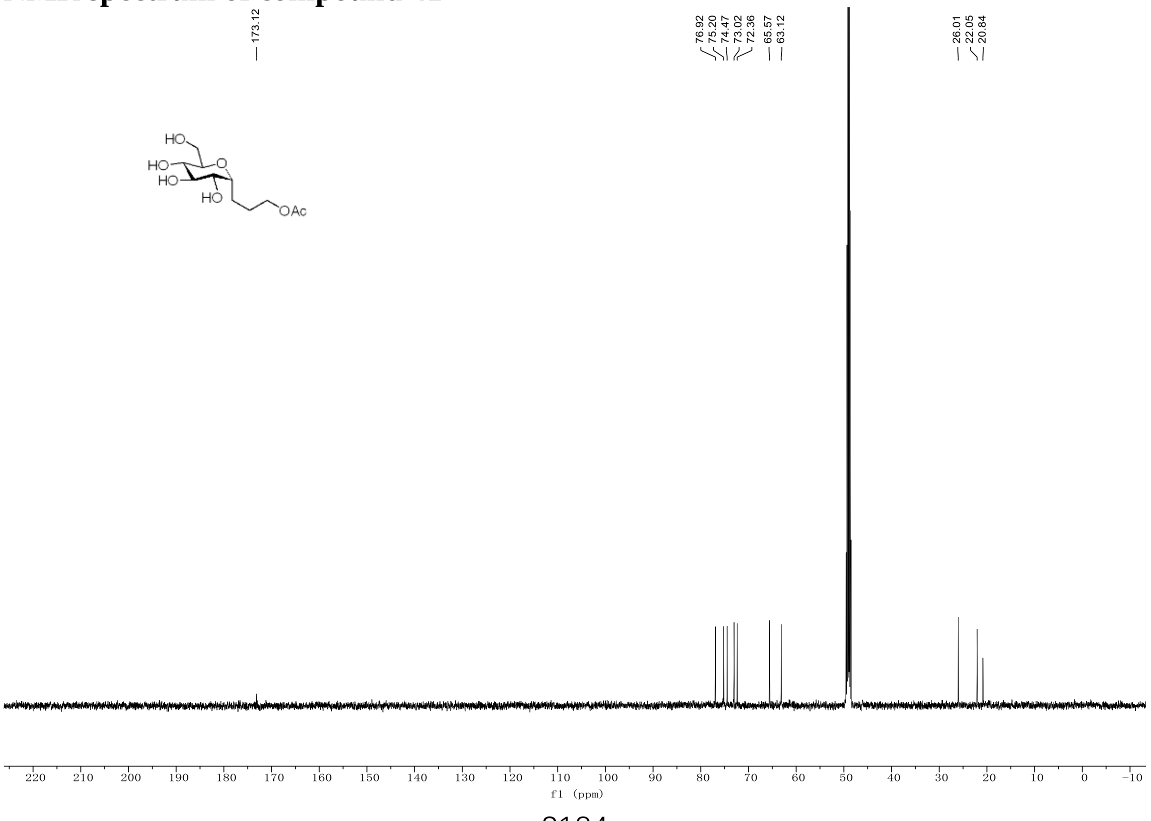

^{1}H NMR spectrum of compound 42

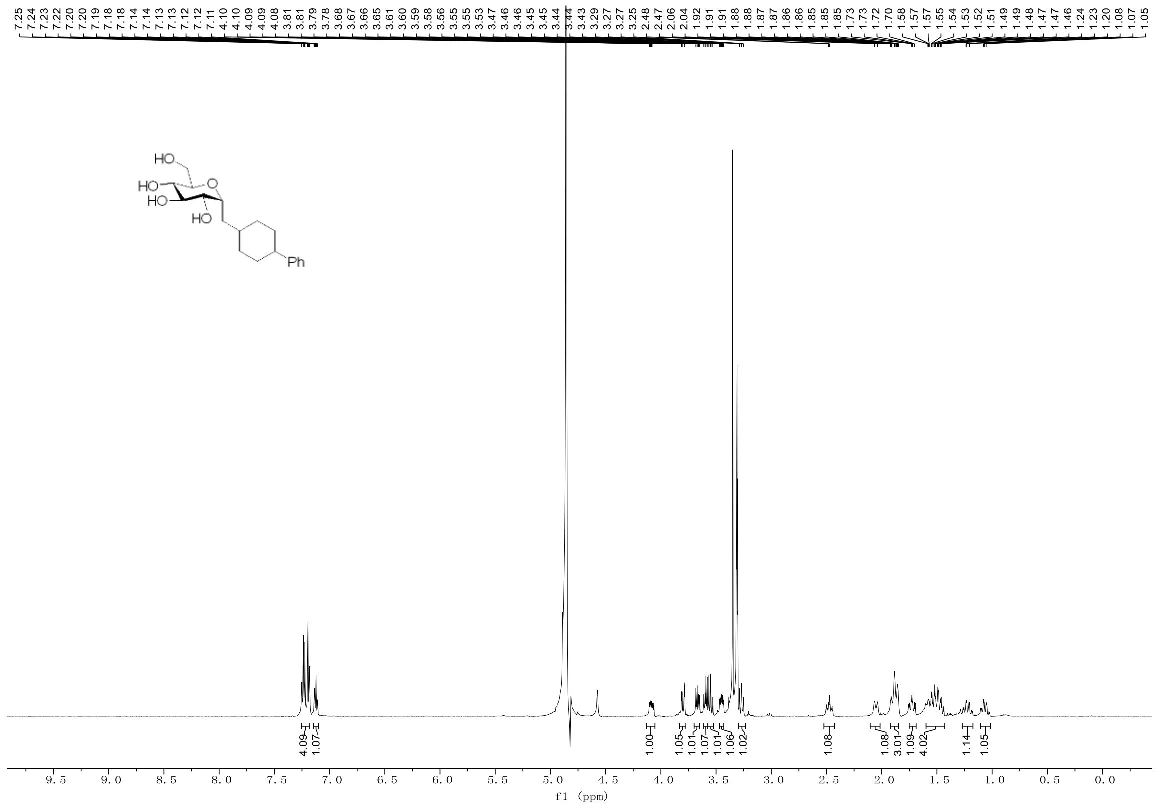

^{13}C NMR spectrum of compound 42

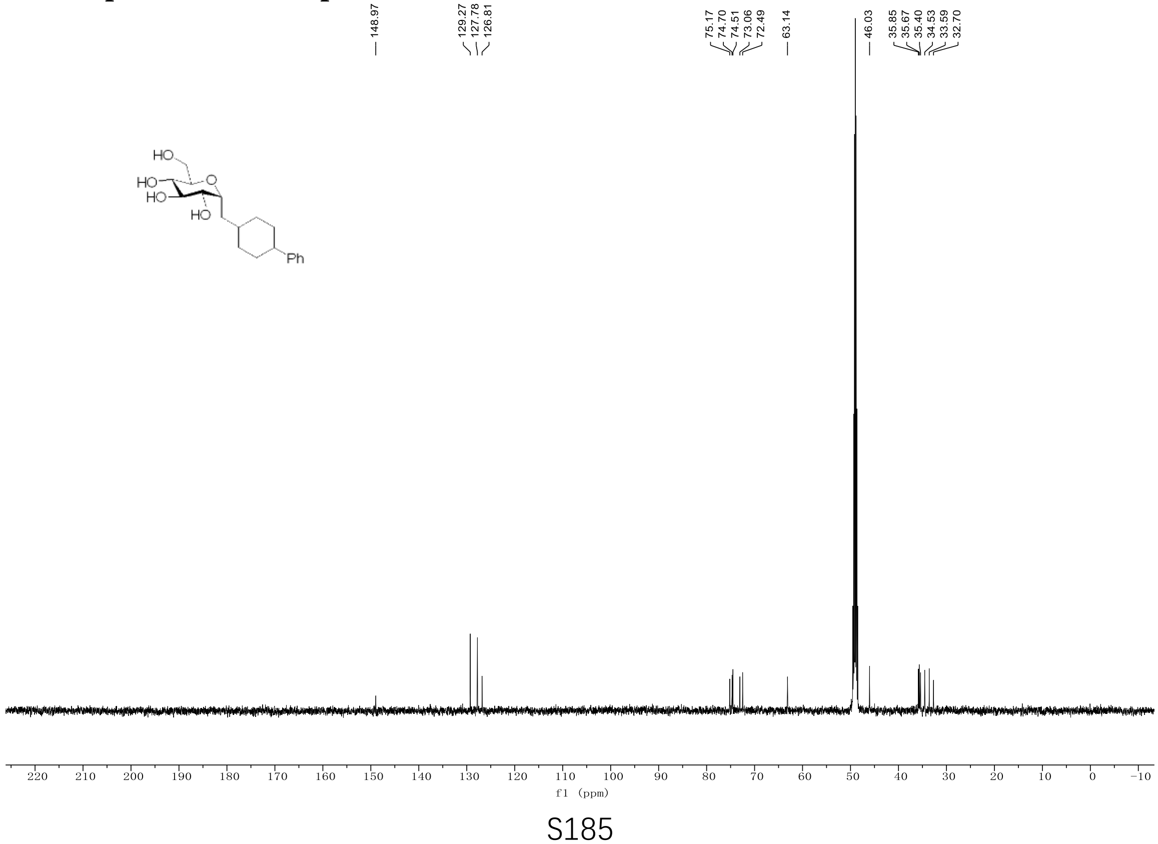

^{1}H NMR spectrum of compound 43

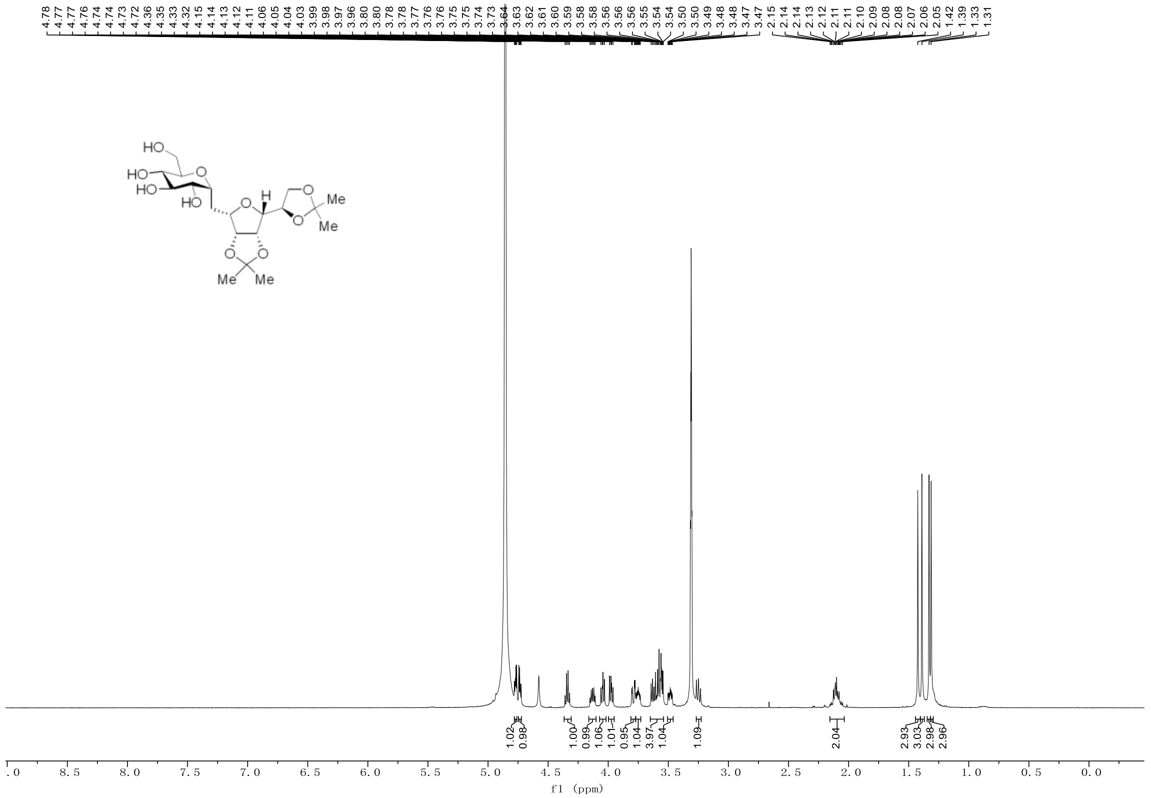

^{13}C NMR spectrum of compound 43

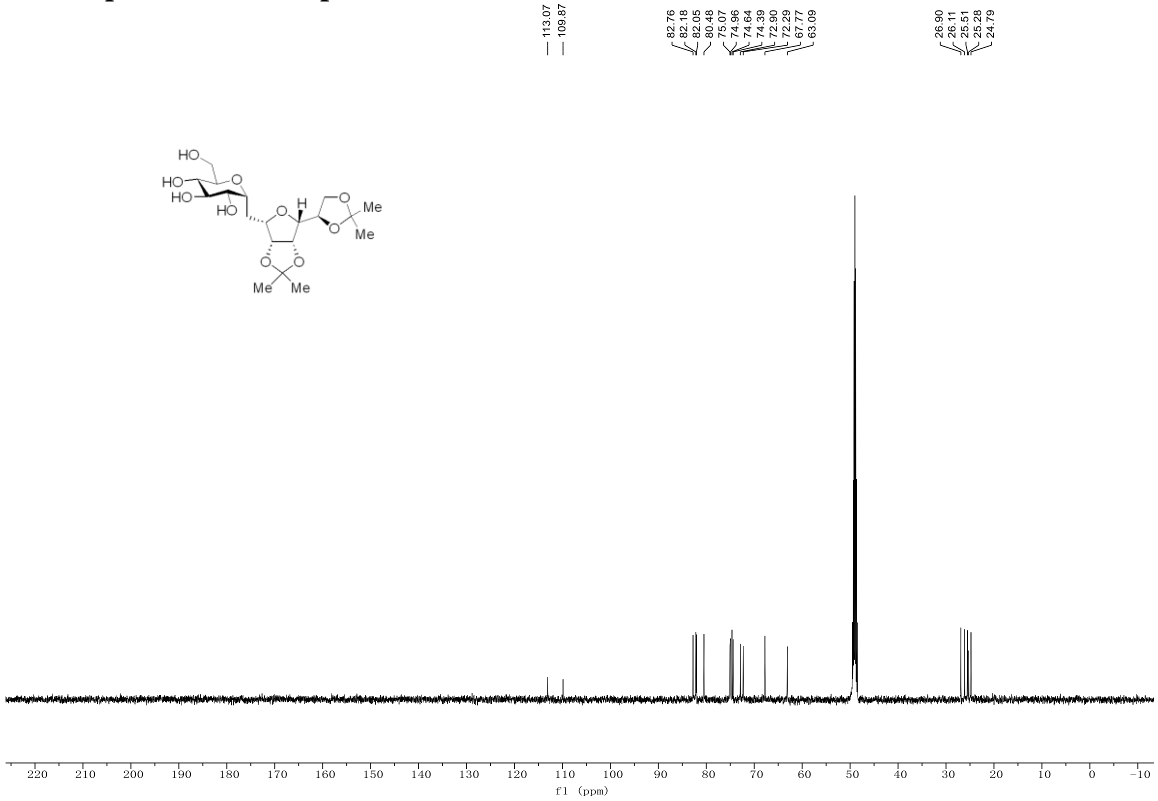

COSY spectrum of compound 43

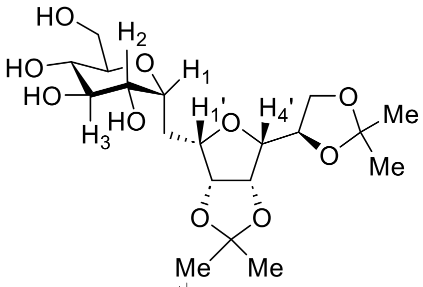

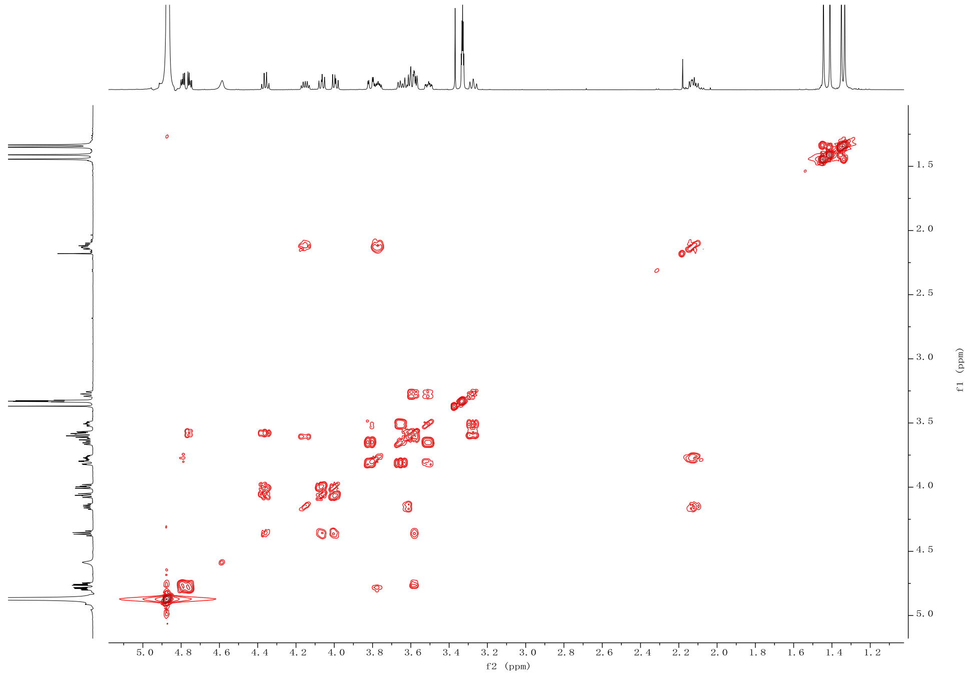

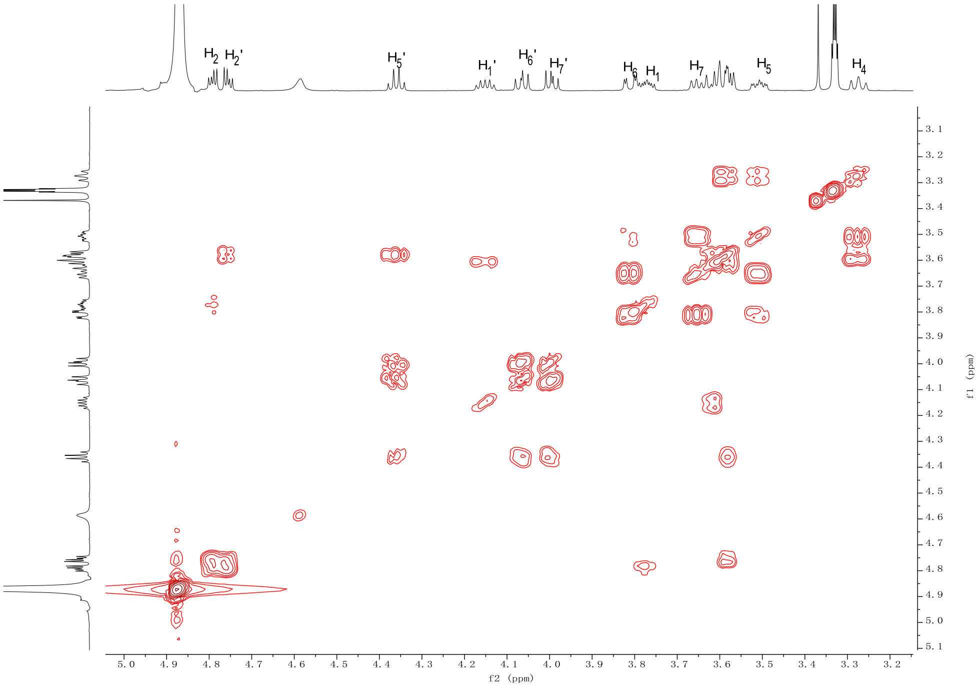

NOE spectrum of compound 43

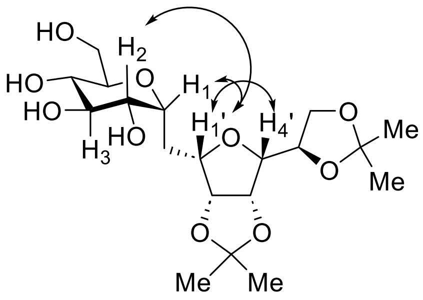

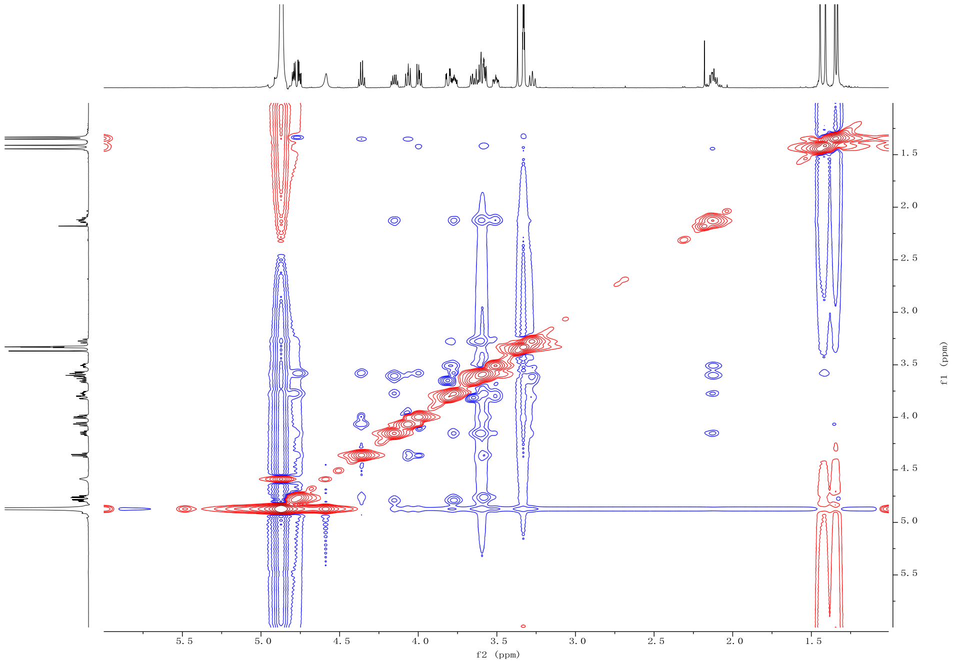

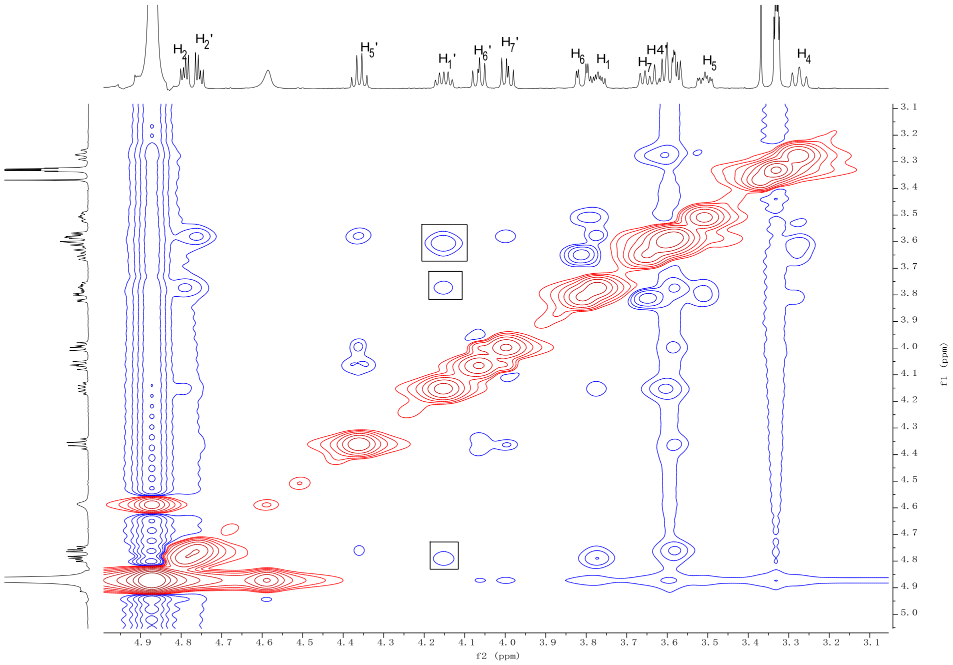

¹H NMR spectrum of compound 44

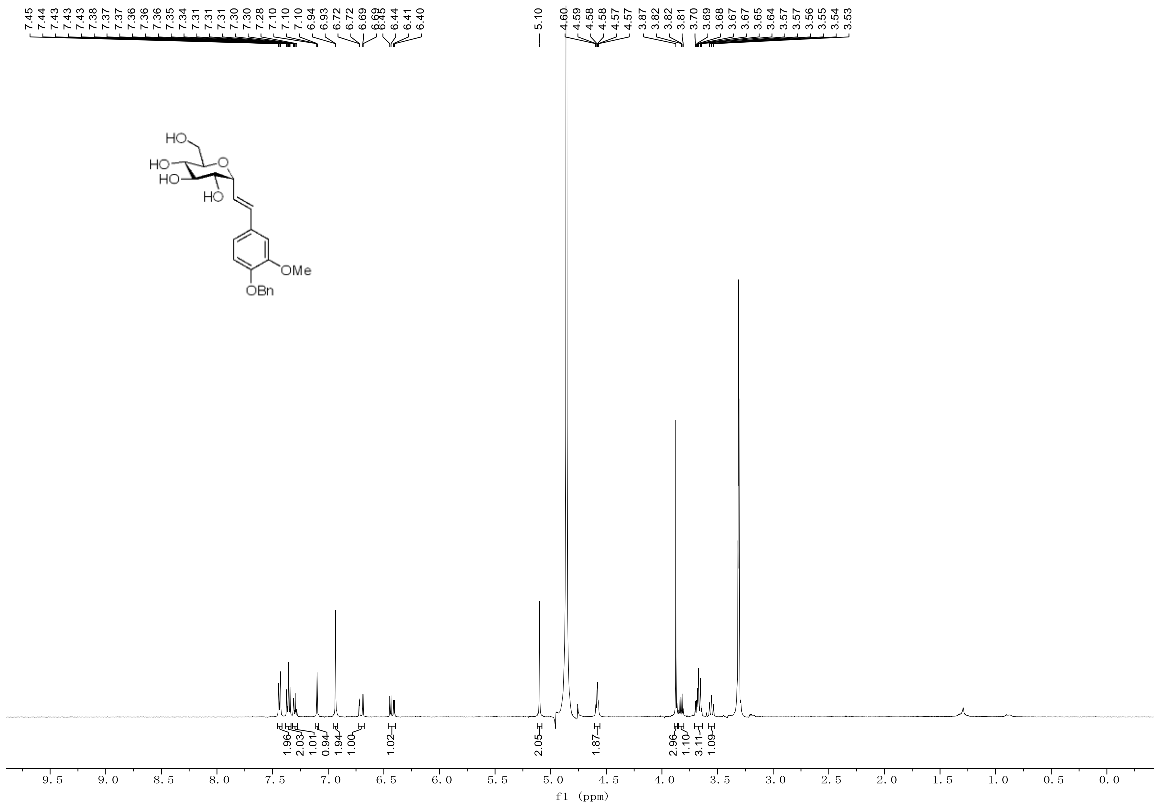

¹³C NMR spectrum of compound 44

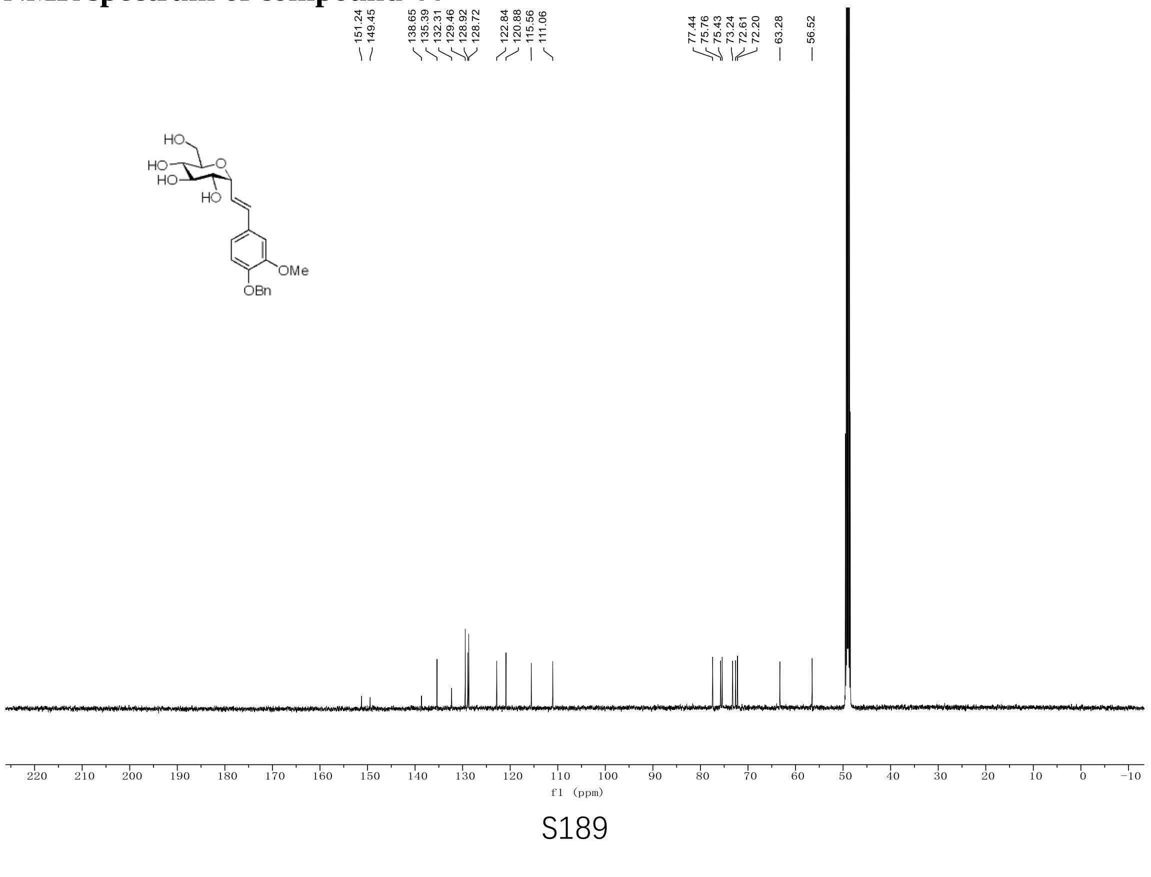

^{1}H NMR spectrum of compound 45

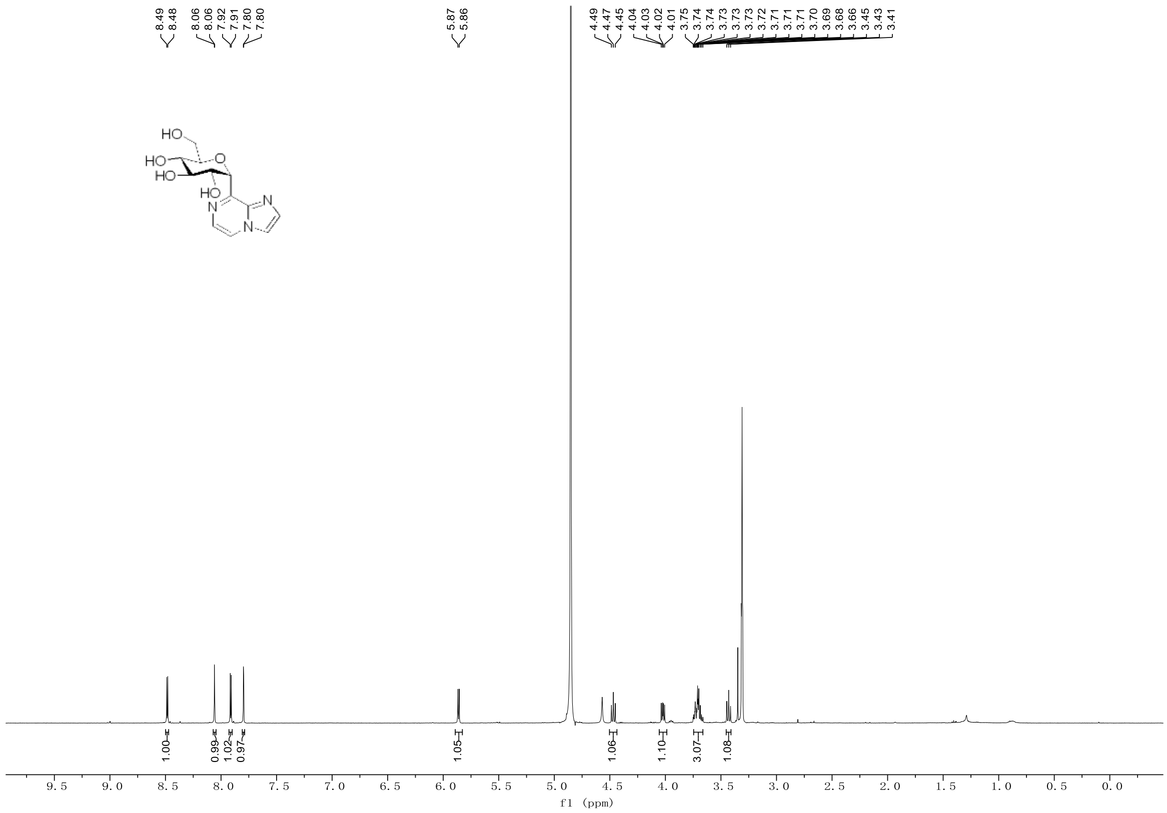

^{13}C NMR spectrum of compound 45

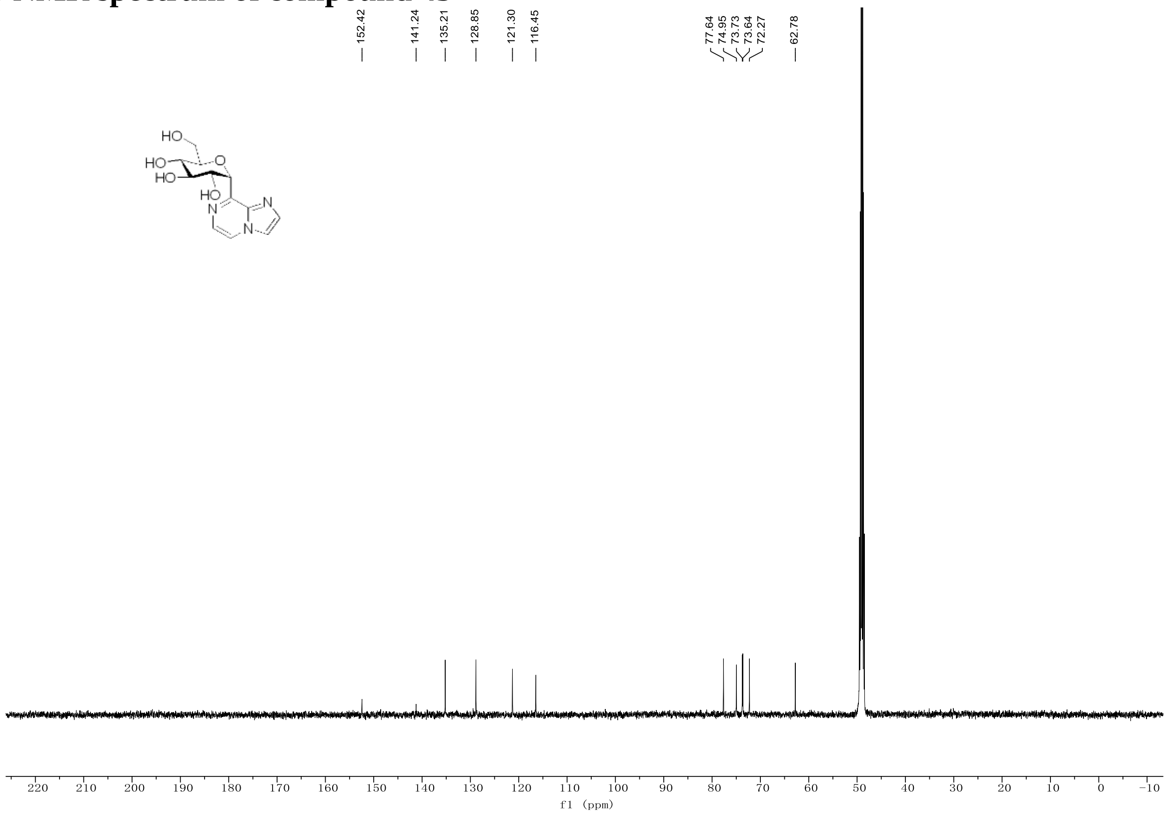

^{1}H NMR spectrum of compound 46

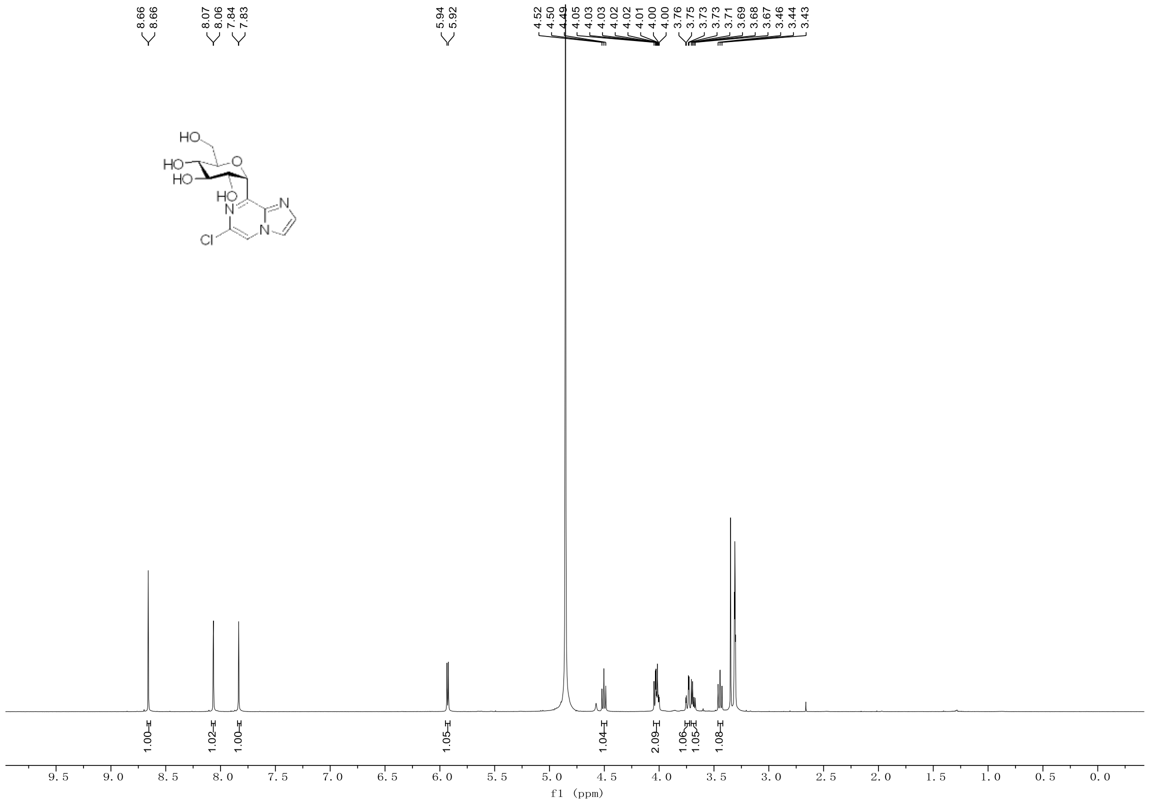

^{13}C NMR spectrum of compound 46

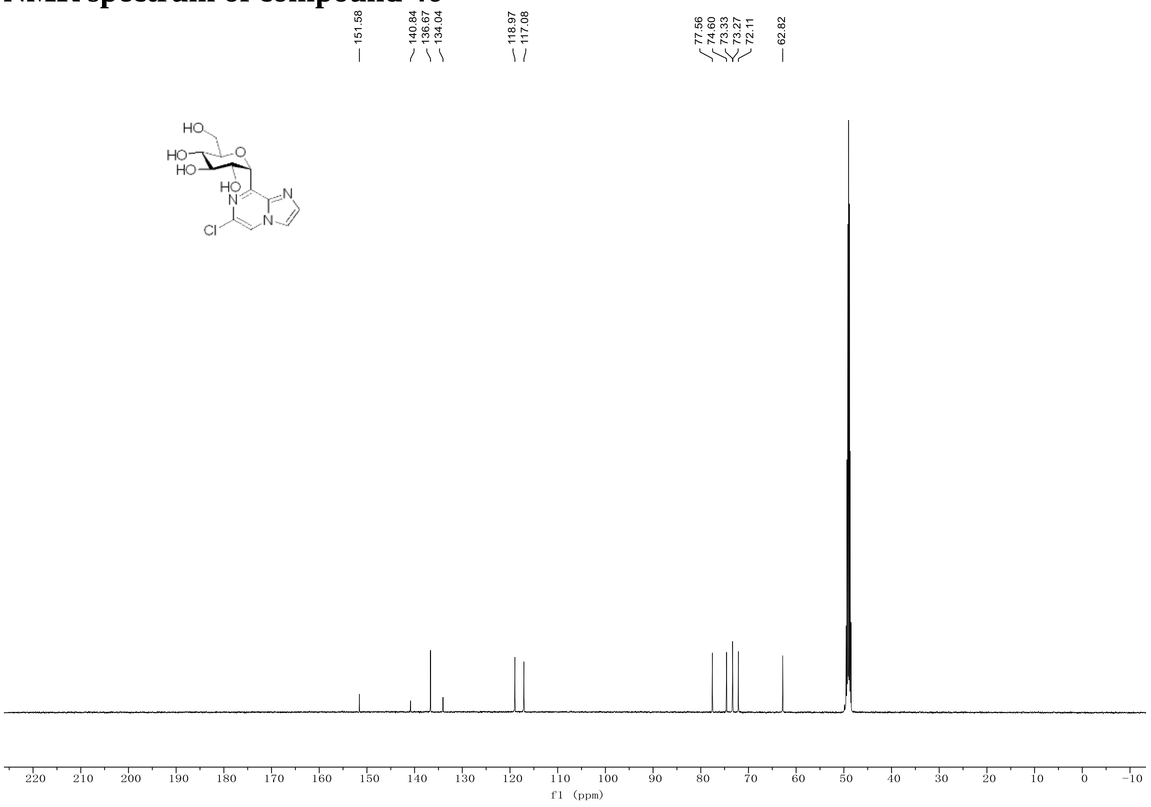

COSY spectrum of compound 46

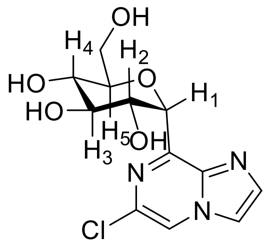

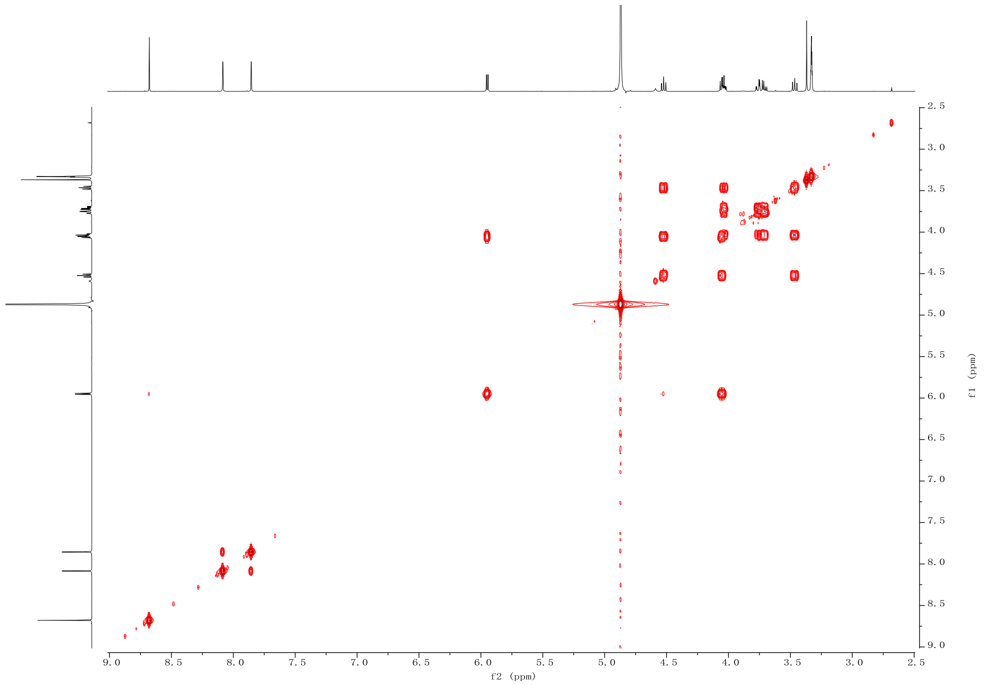

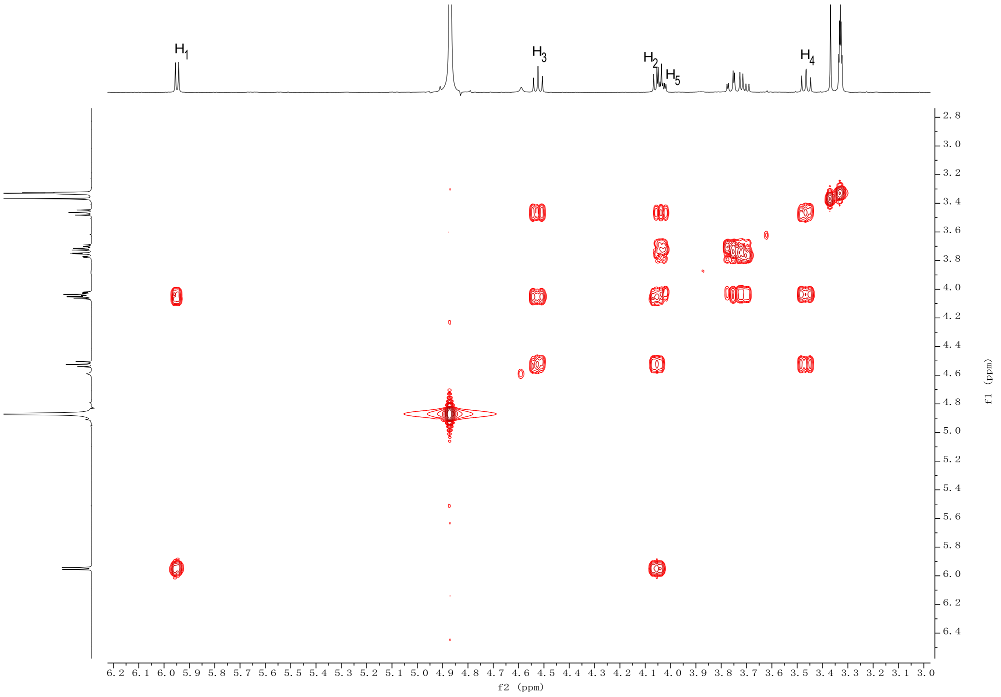

NOE spectrum of compound 46

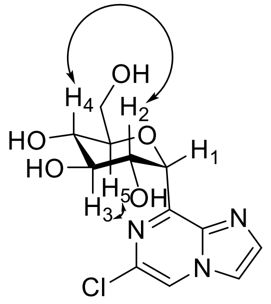

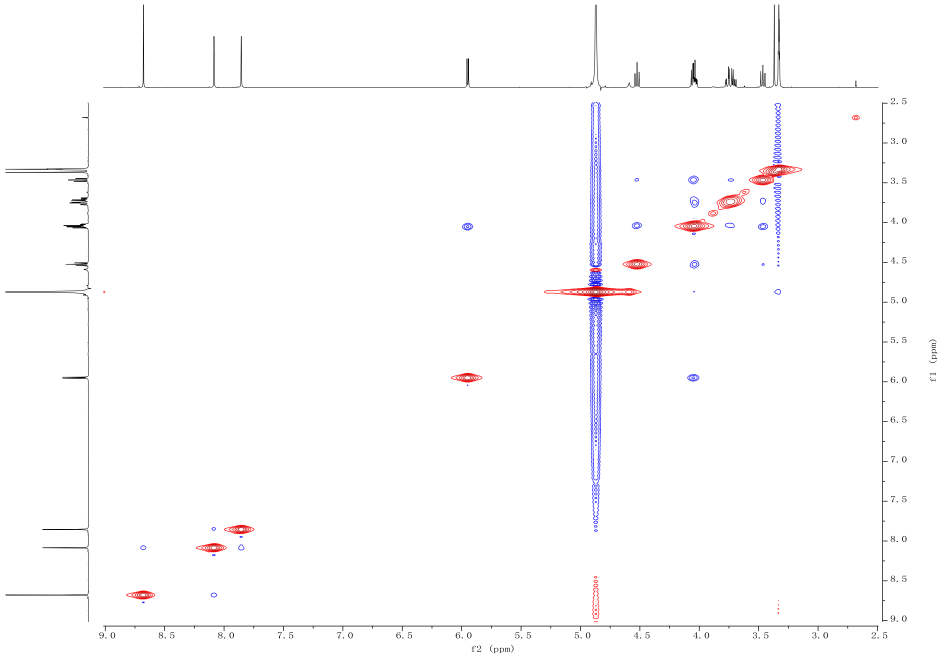

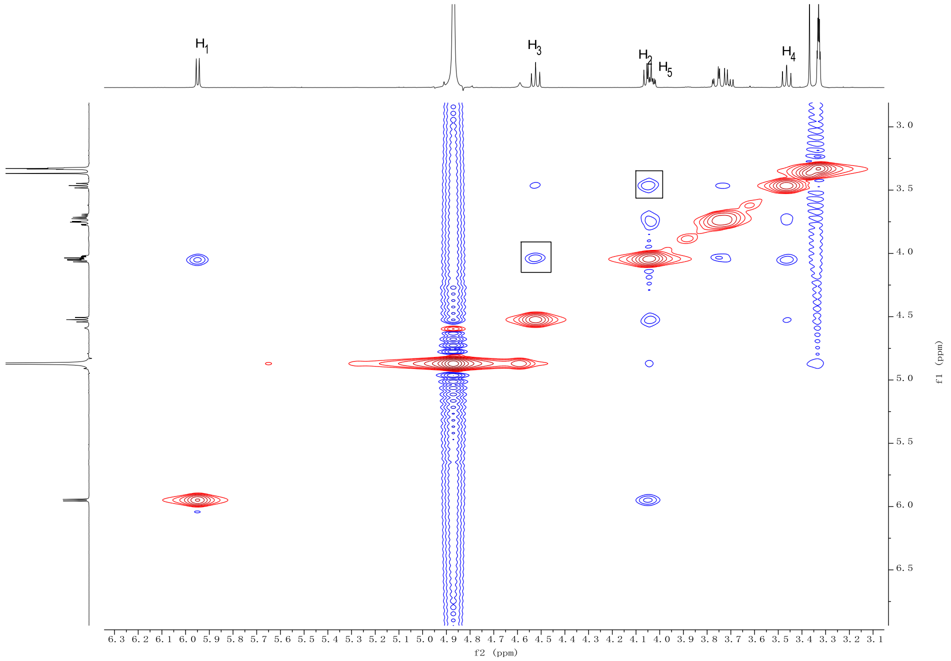

^{1}H NMR spectrum of compound 47

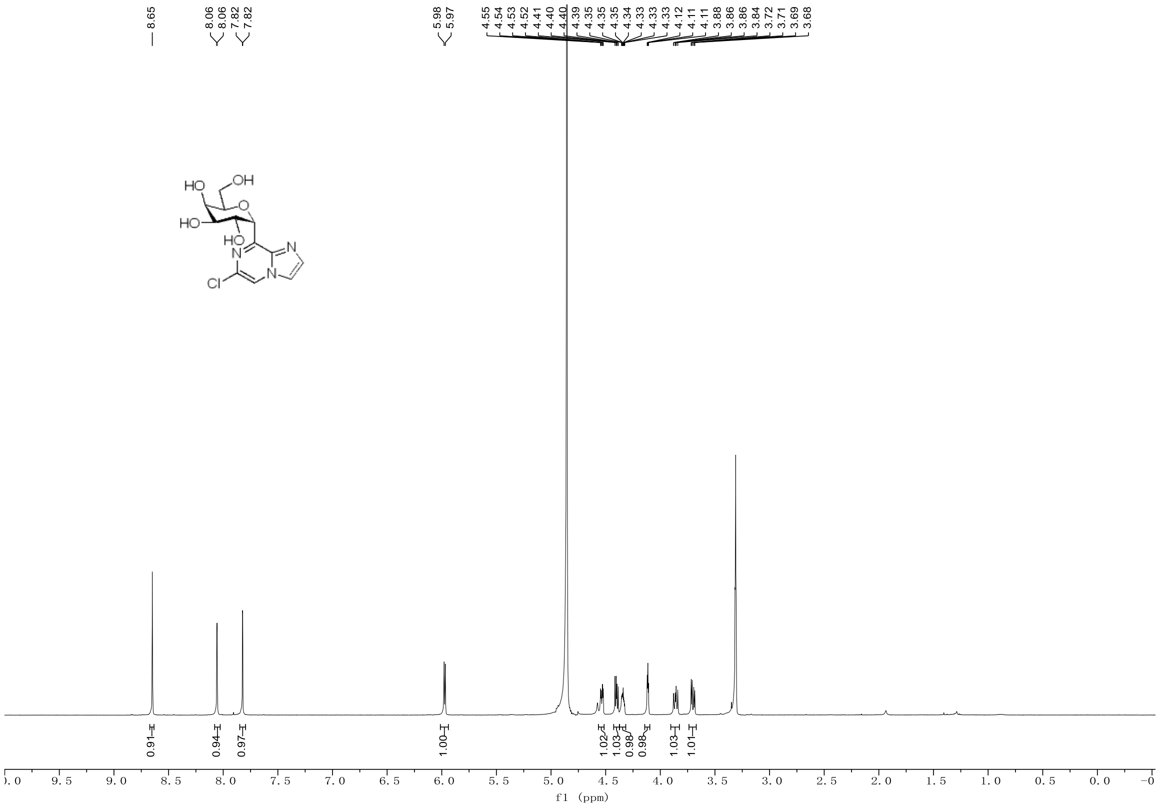

^{13}C NMR spectrum of compound 47

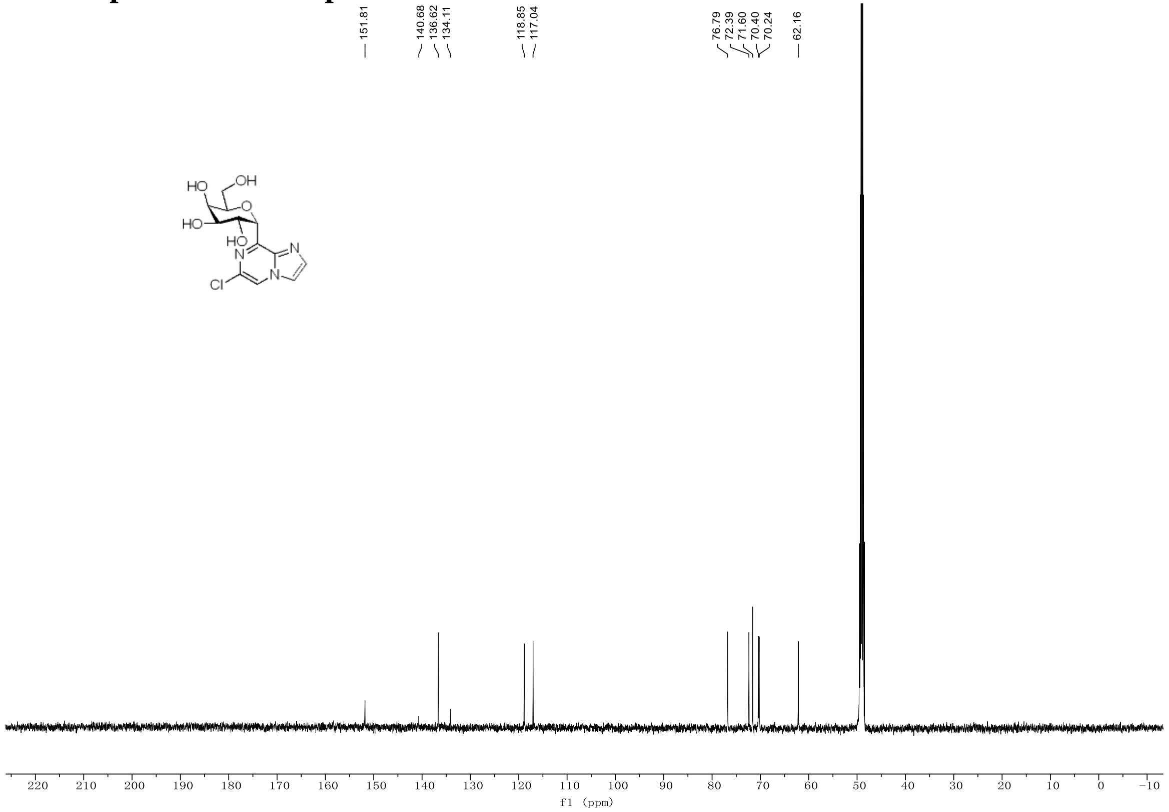

^{1}H NMR spectrum of compound 48

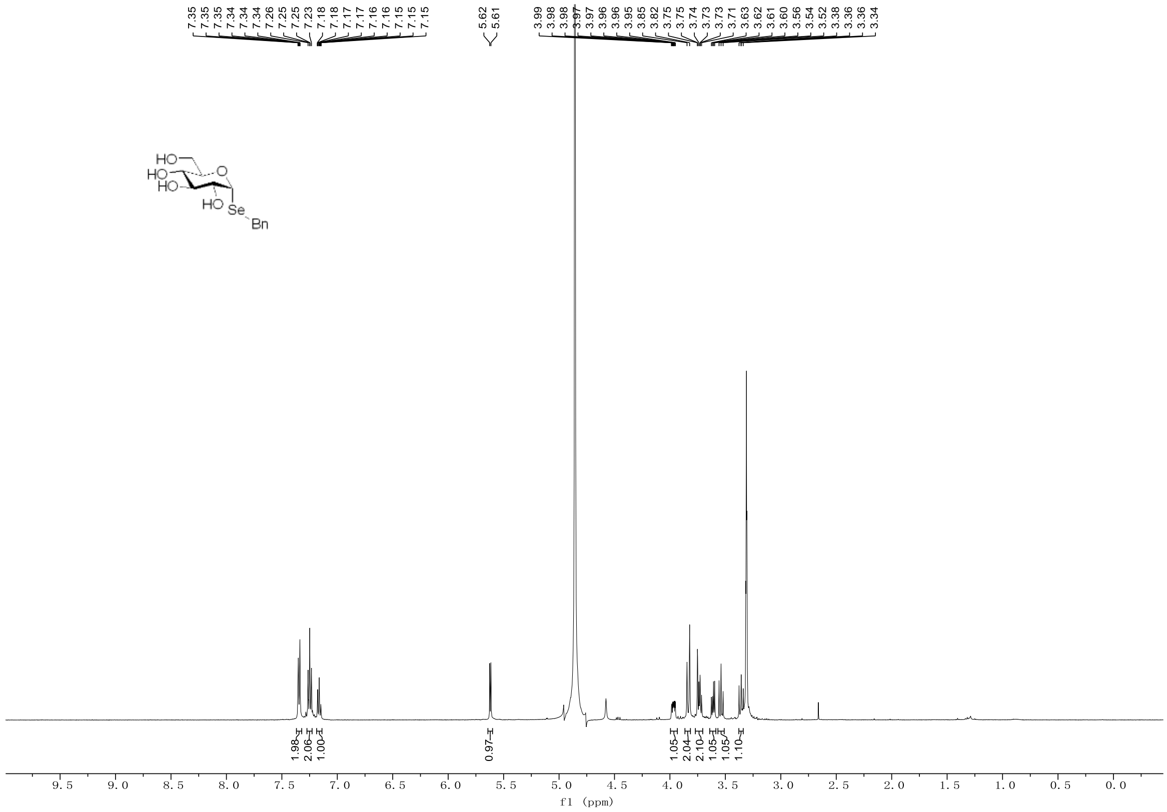

^{13}C NMR spectrum of compound 48

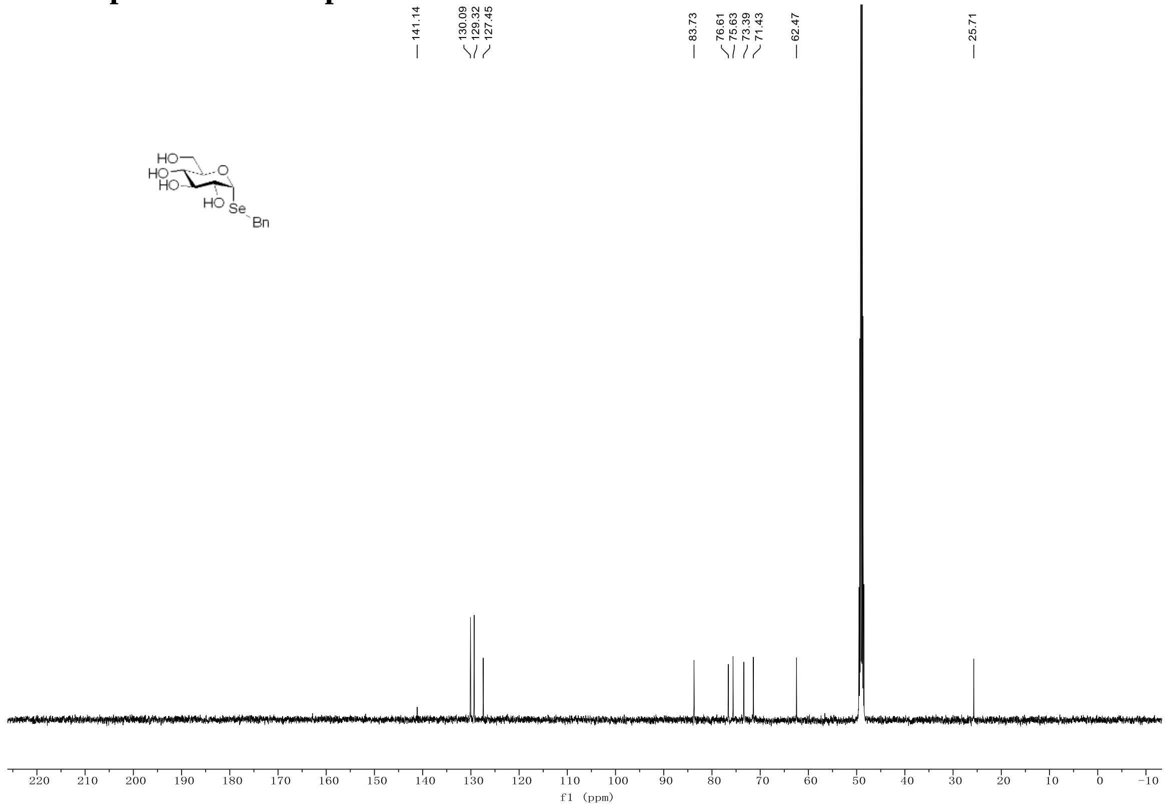

^{1}H NMR spectrum of compound 49

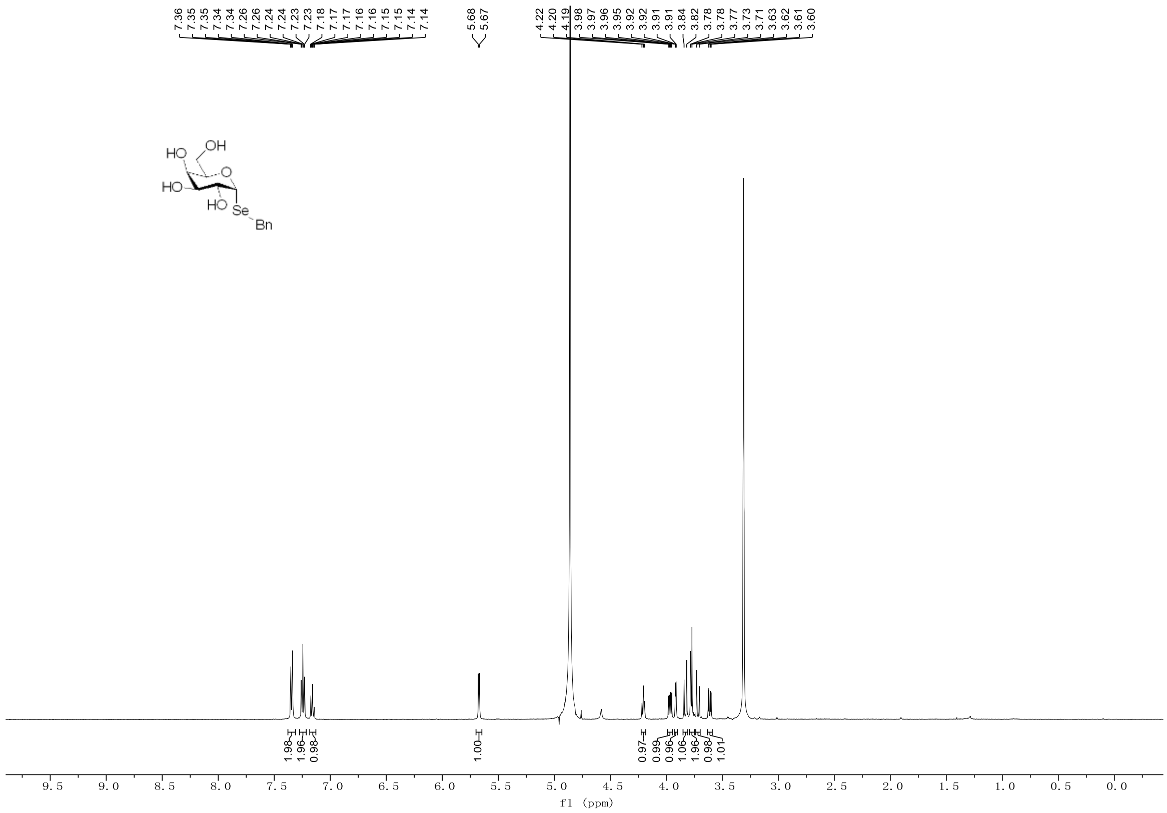

^{13}C NMR spectrum of compound 49

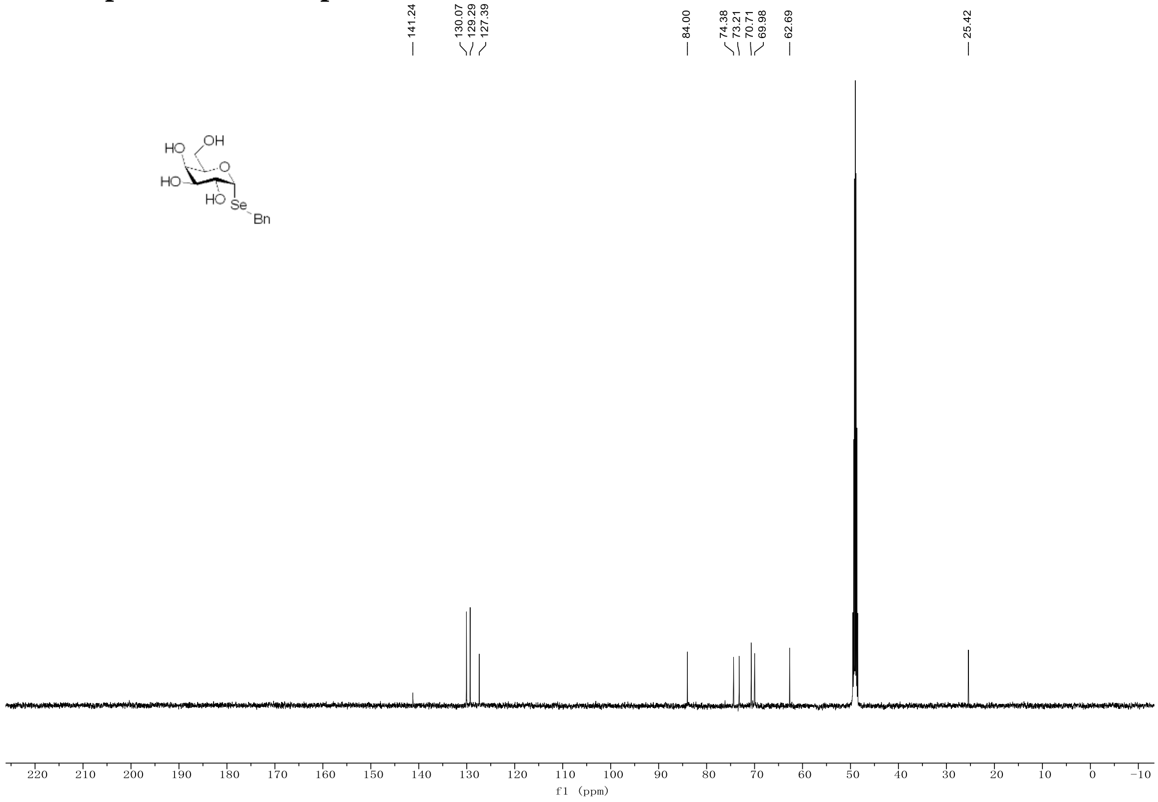

^{1}H NMR spectrum of compound 50

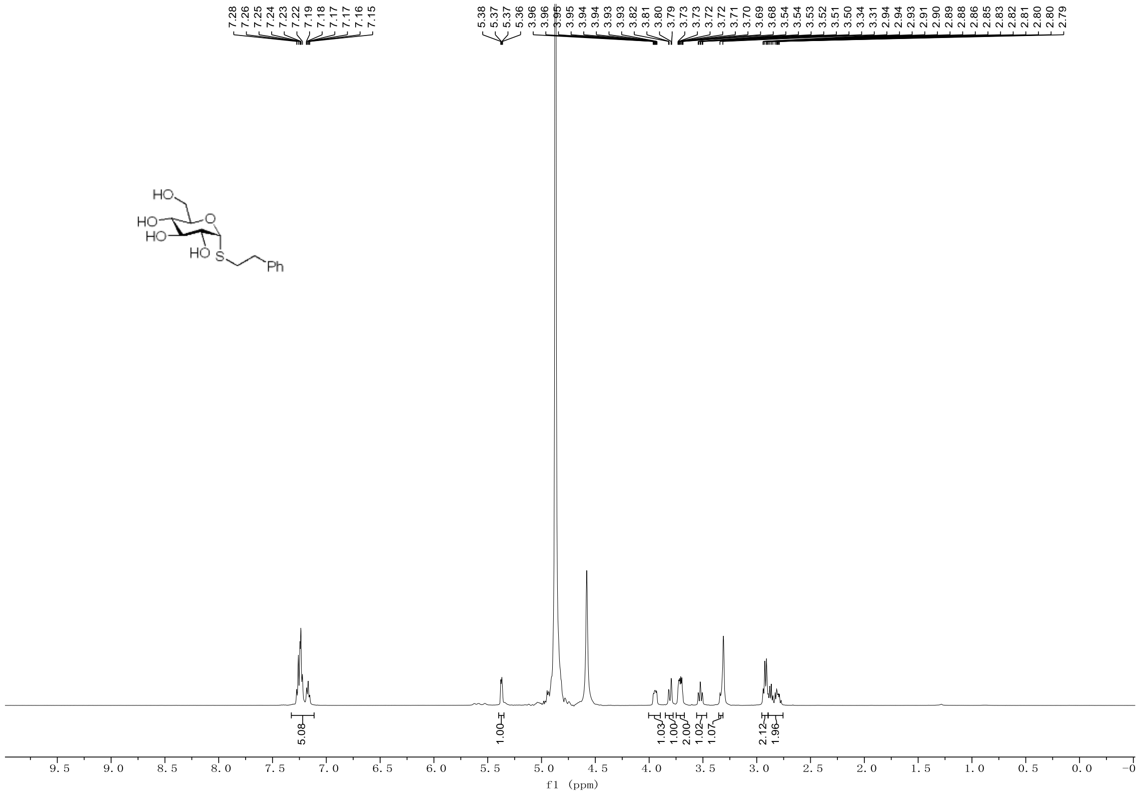

^{13}C NMR spectrum of compound 50

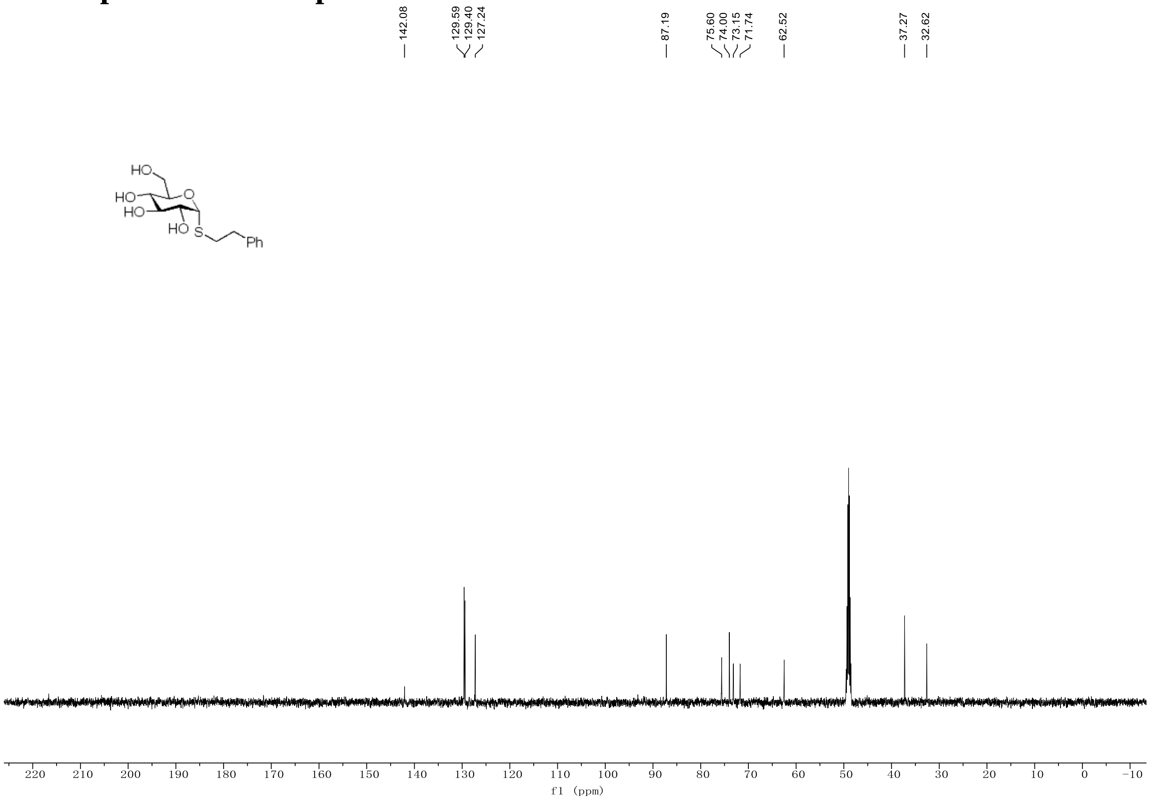

^{1}H NMR spectrum of compound 51

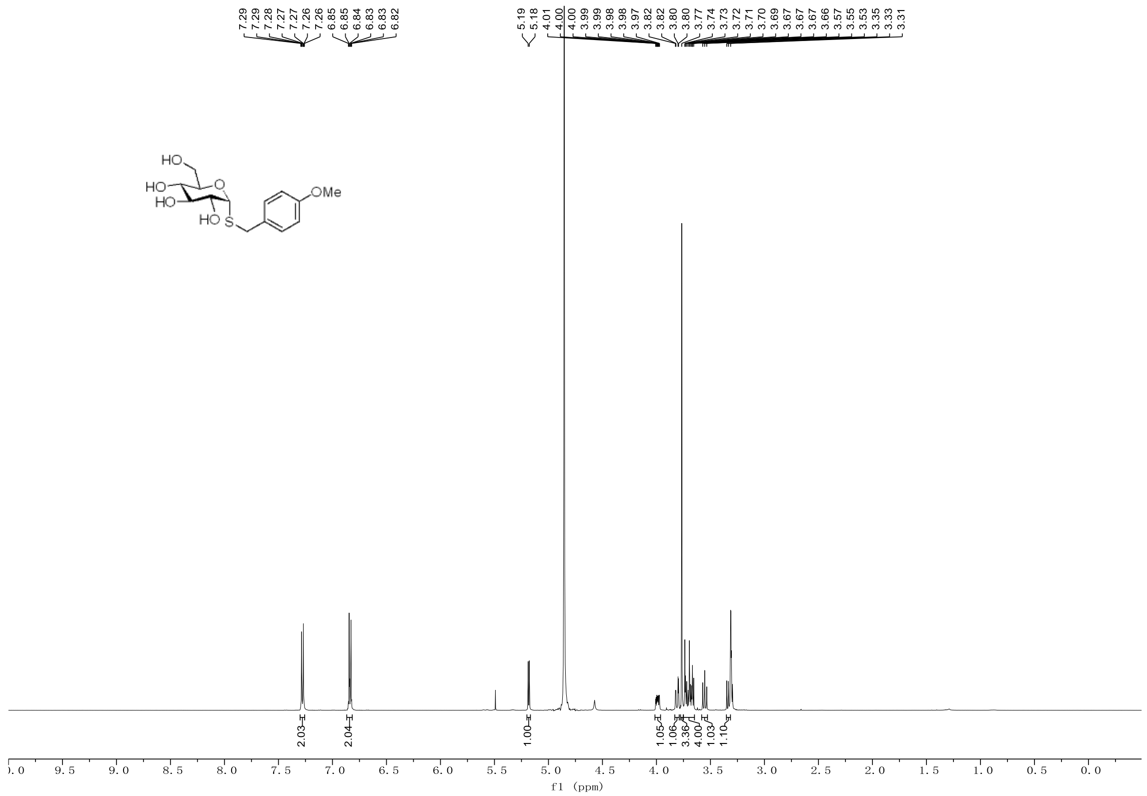

^{13}C NMR spectrum of compound 51

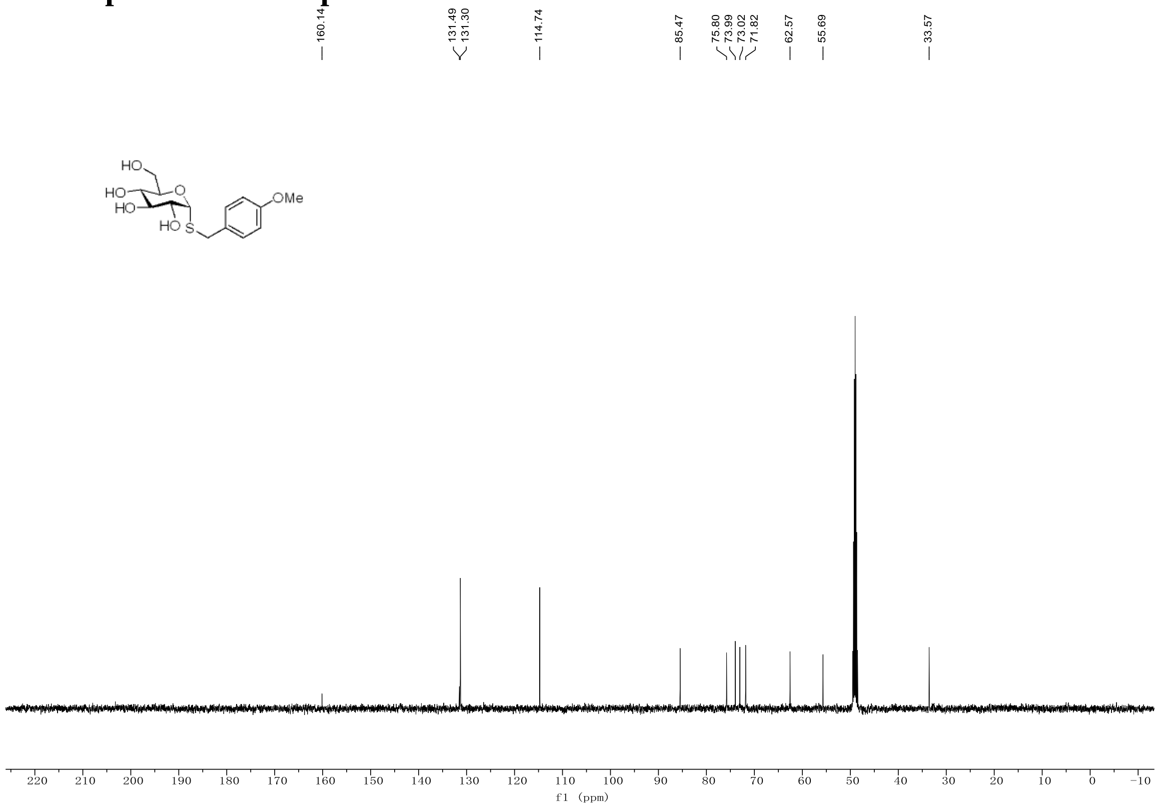

^{1}H NMR spectrum of compound 52

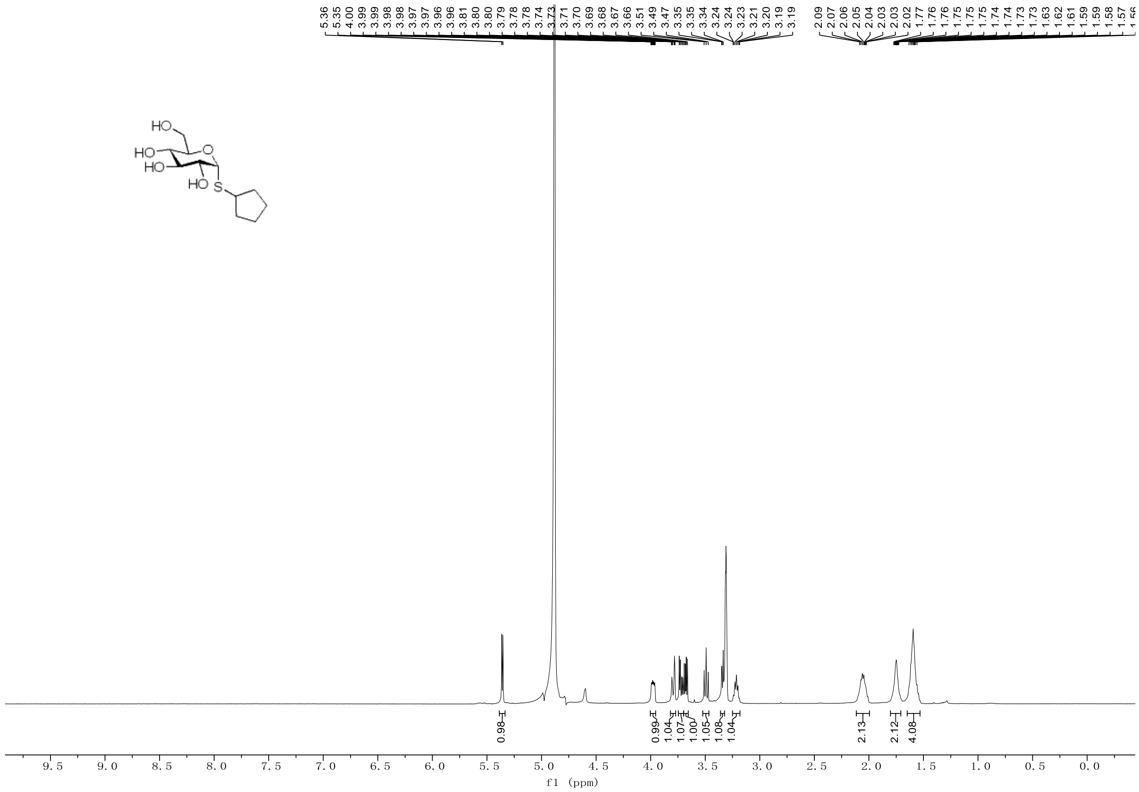

^{13}C NMR spectrum of compound 52

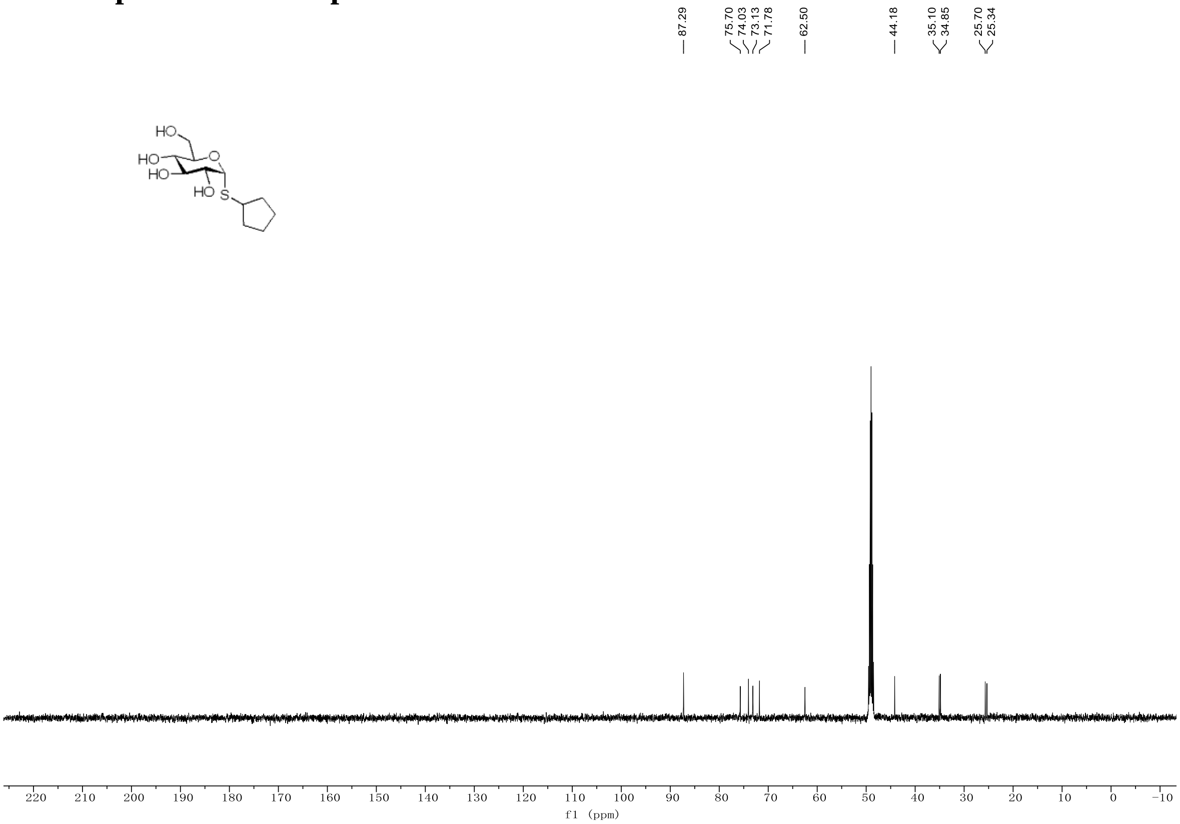

^{1}H NMR spectrum of compound 53

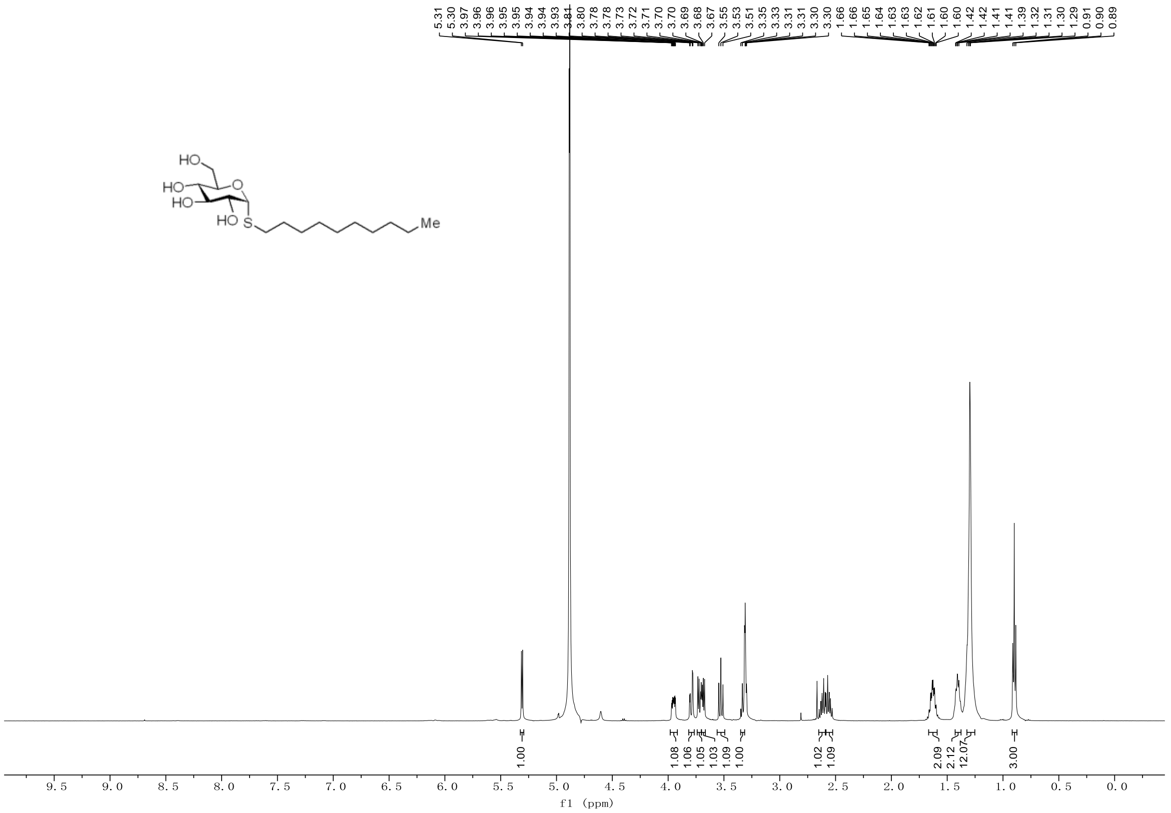

^{13}C NMR spectrum of compound 53

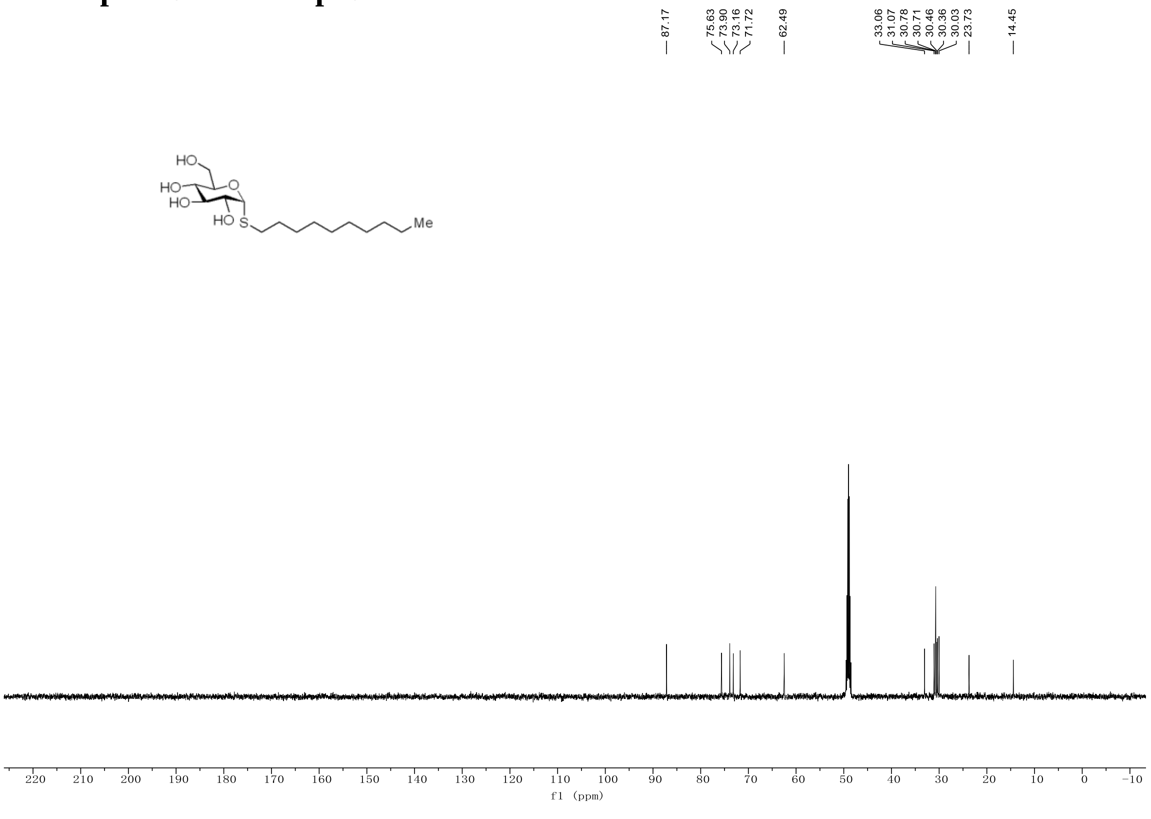

^{1}H NMR spectrum of compound 54

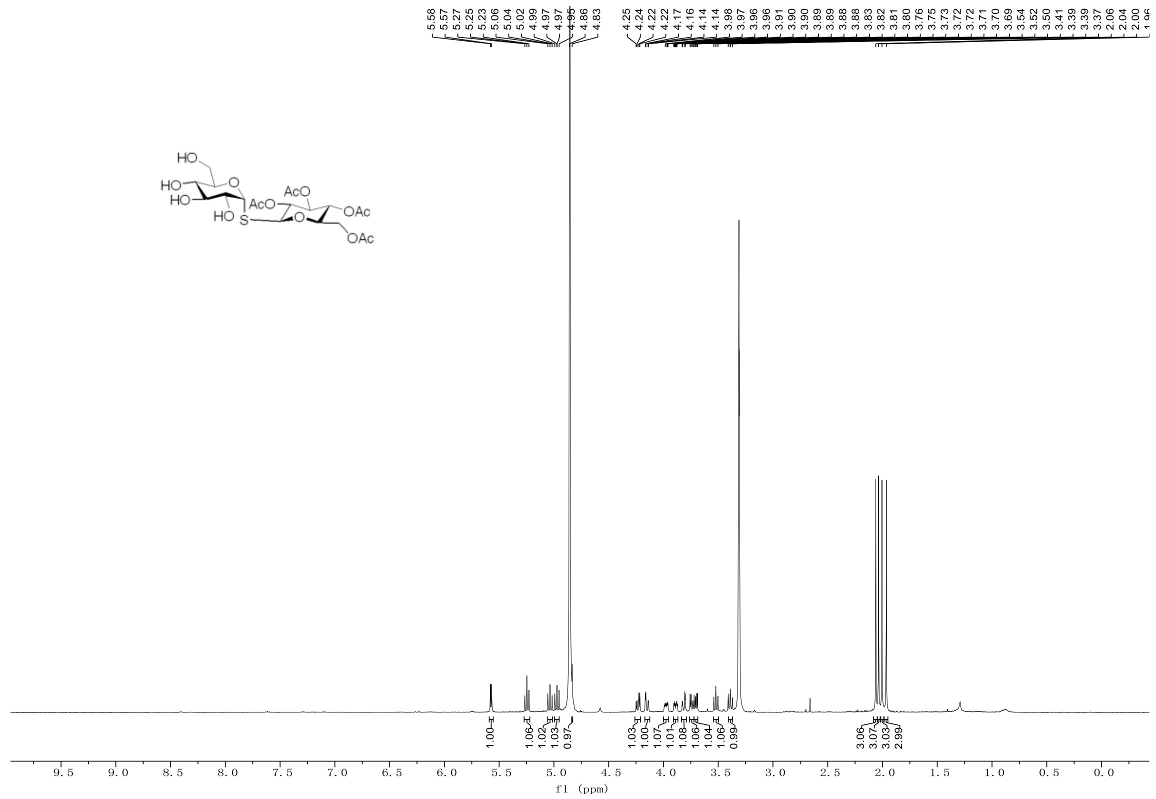

^{13}C NMR spectrum of compound 54

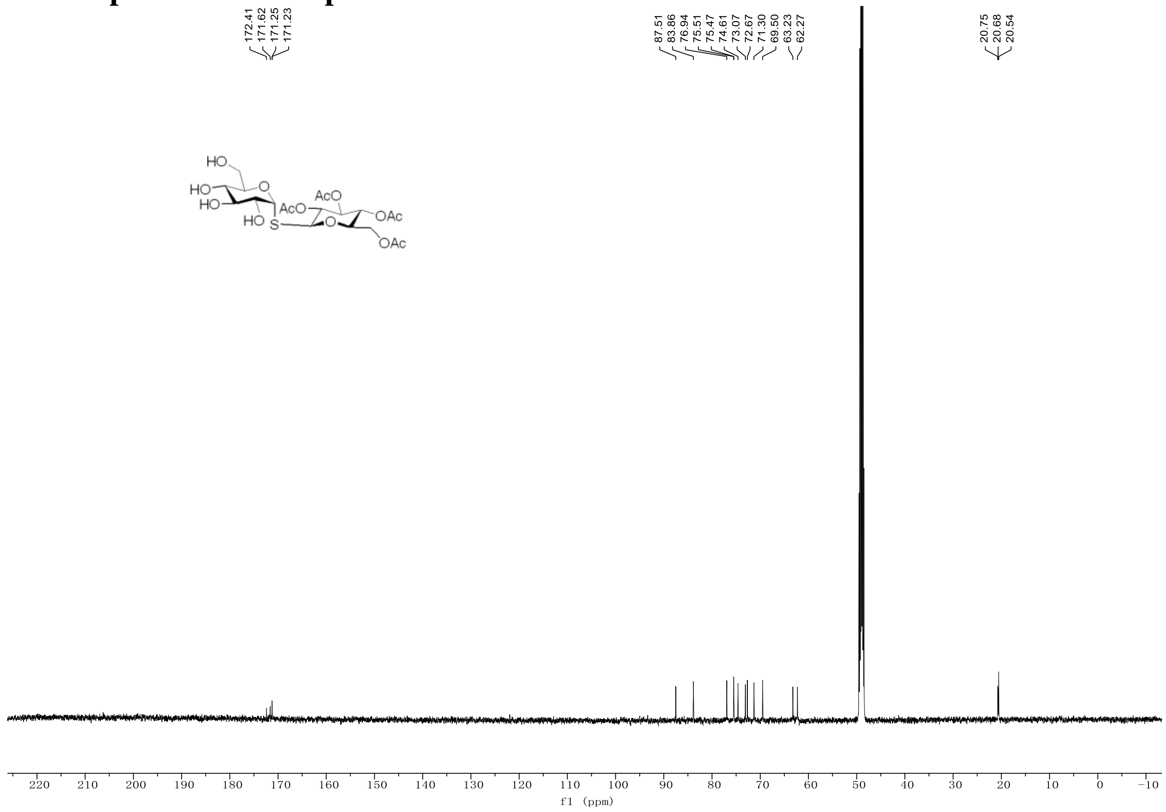

COSY spectrum of compound 54

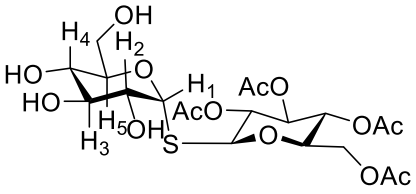

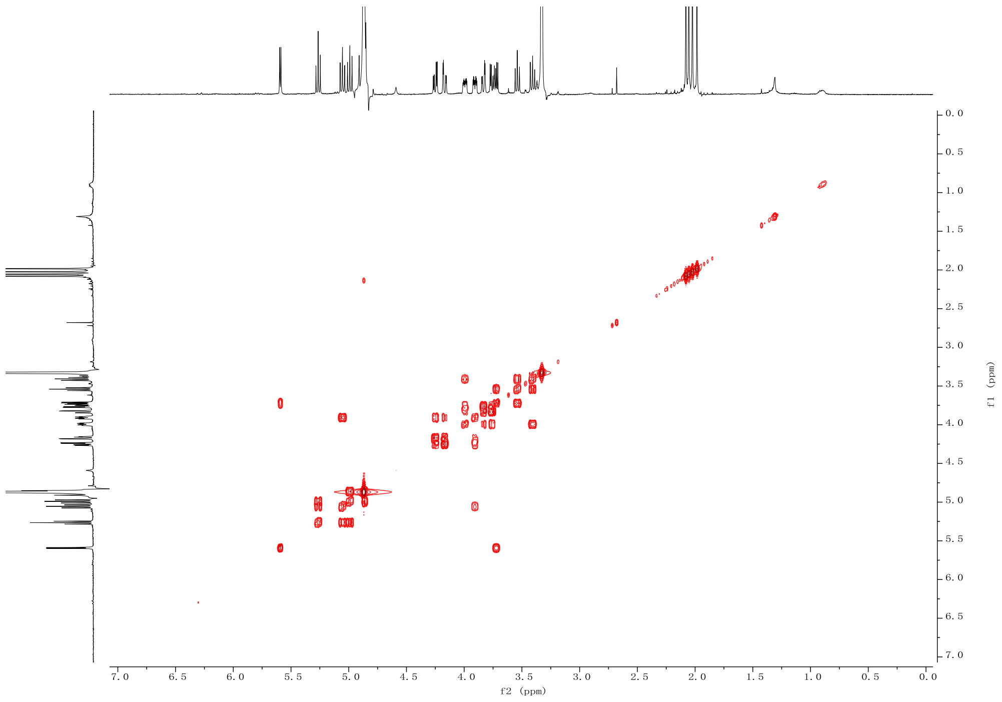

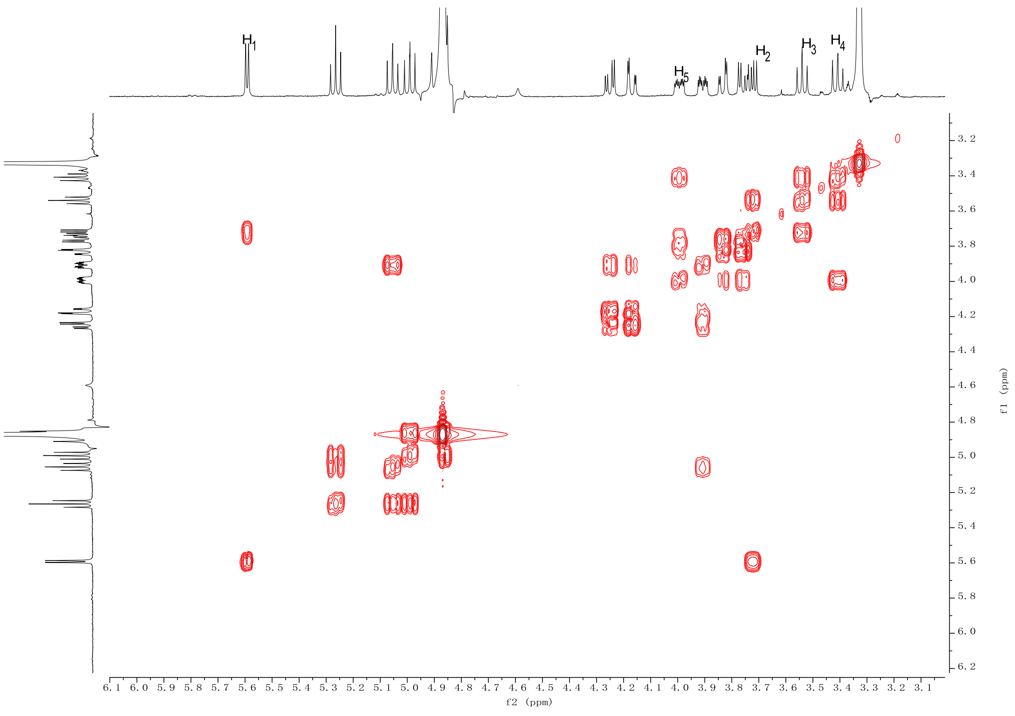

NOE spectrum of compound 54

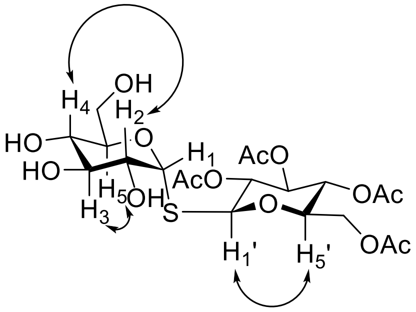

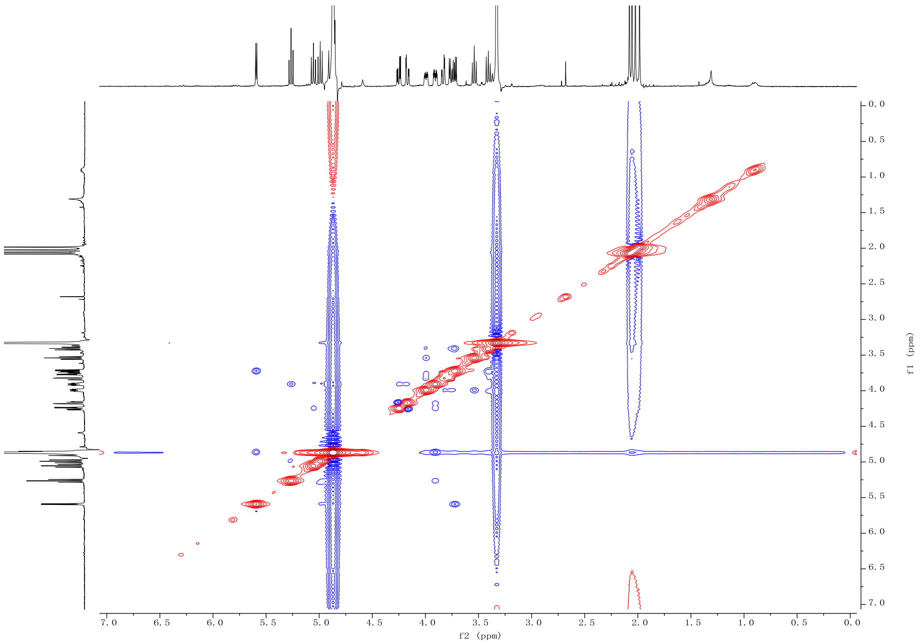

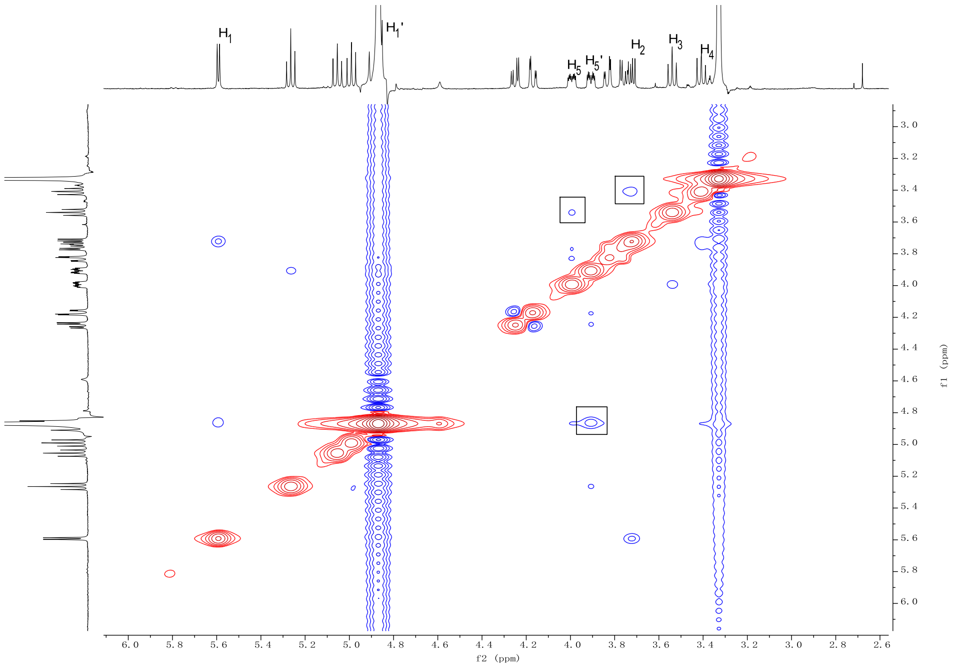

^{1}H NMR spectrum of compound 55

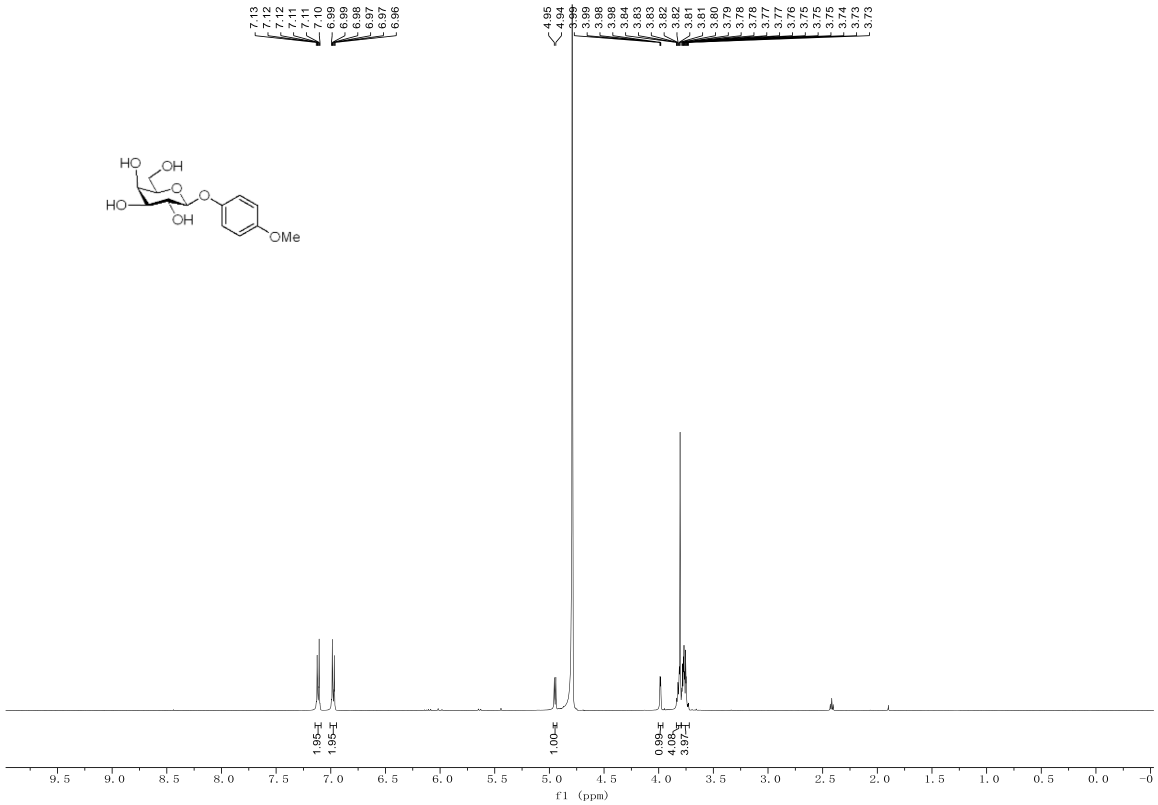

^{13}C NMR spectrum of compound 55

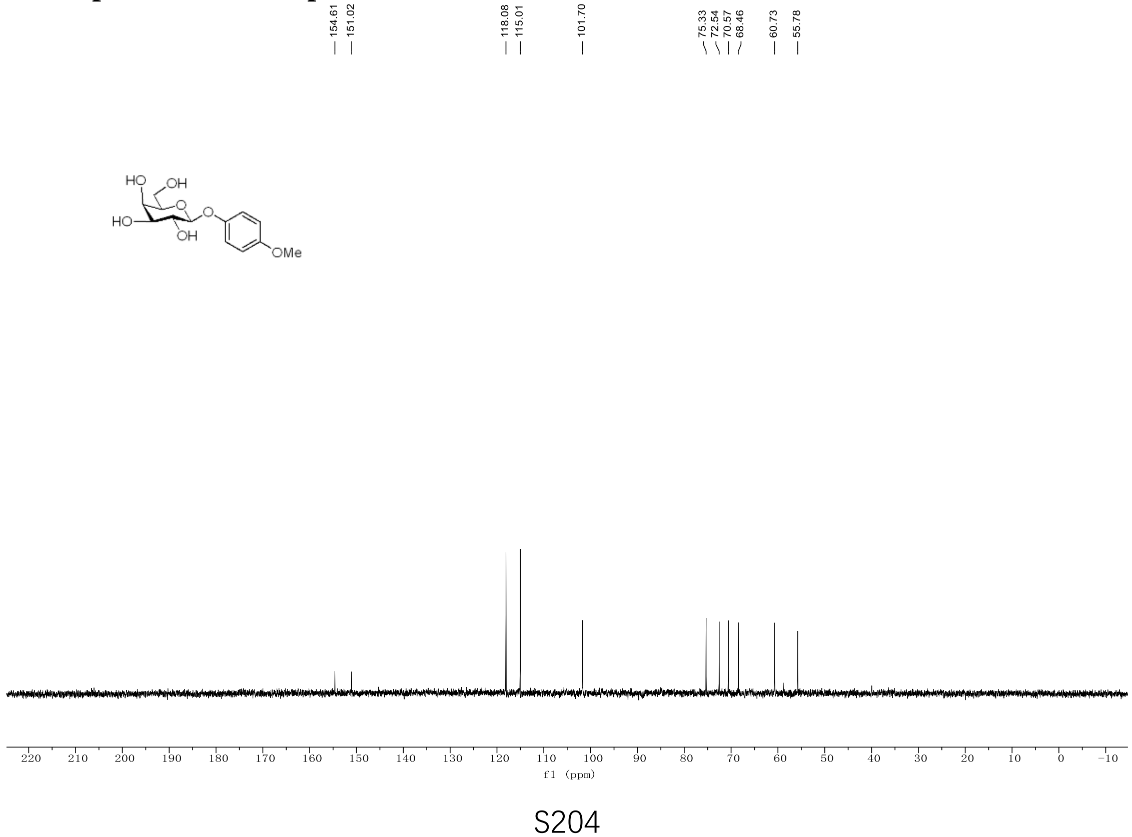

^{1}H NMR spectrum of compound 56

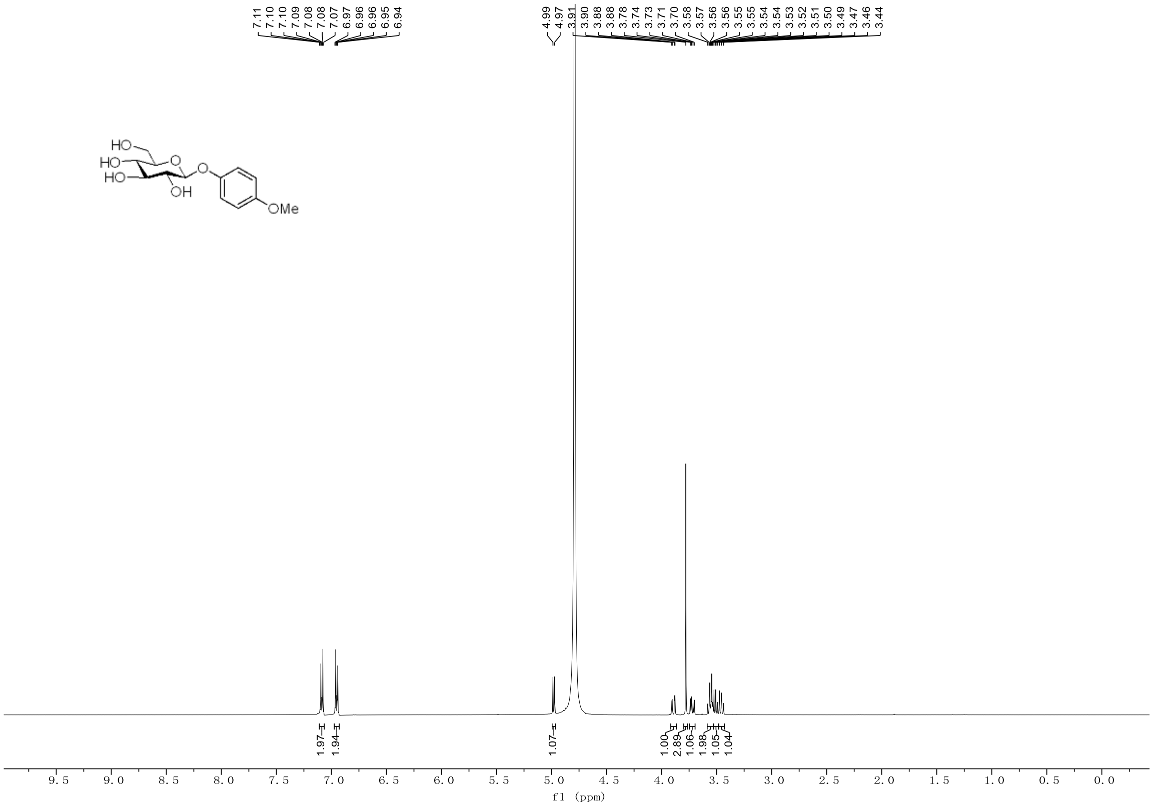

^{13}C NMR spectrum of compound 56

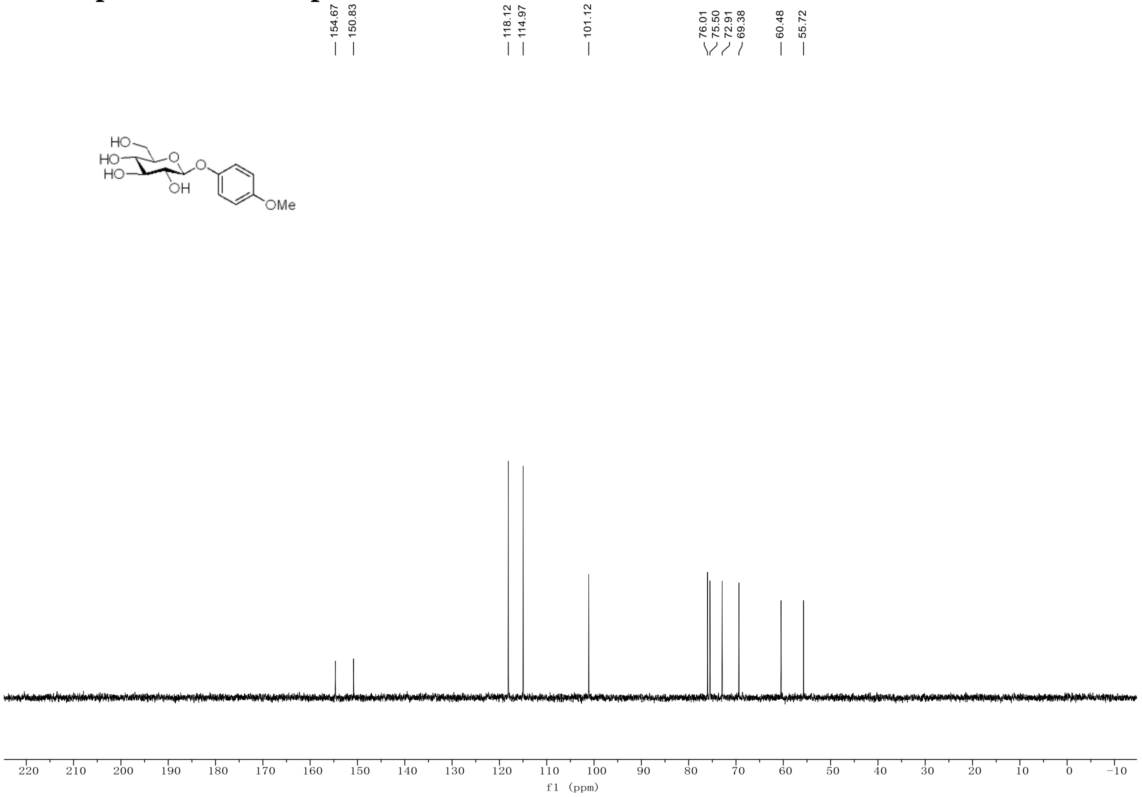

^{1}H NMR spectrum of compound 57

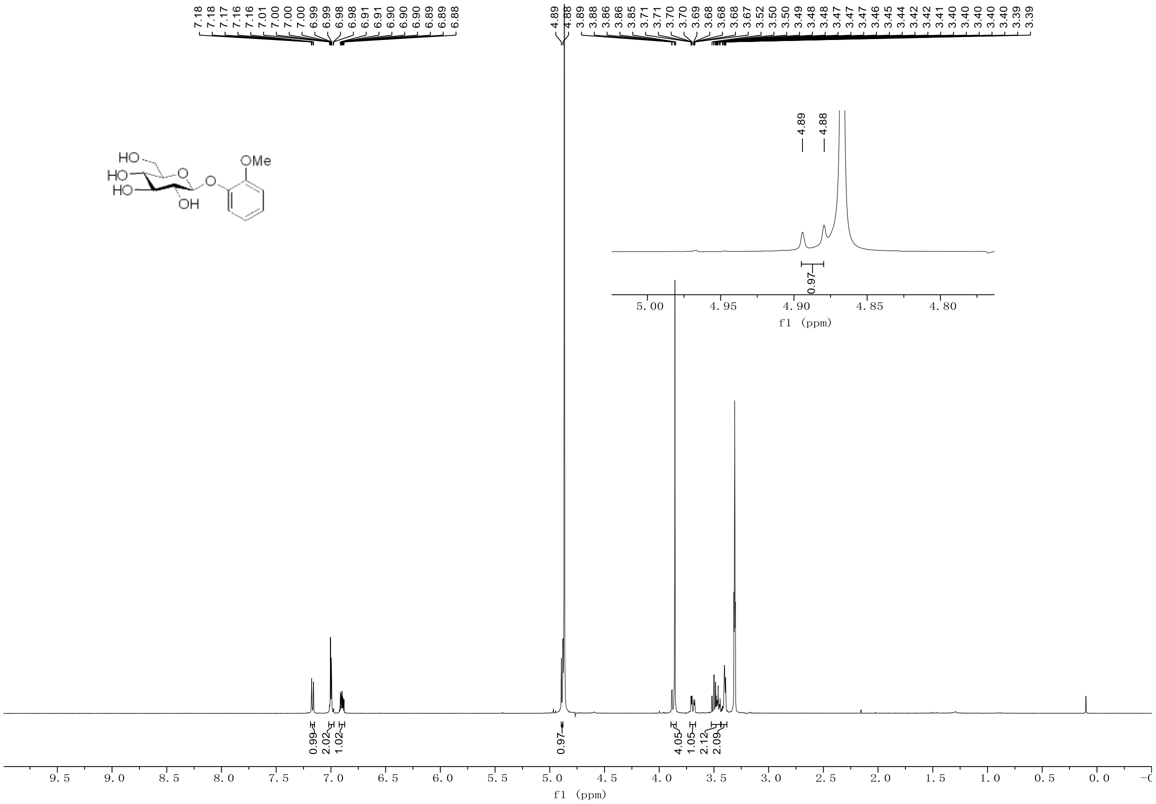

^{13}C NMR spectrum of compound 57

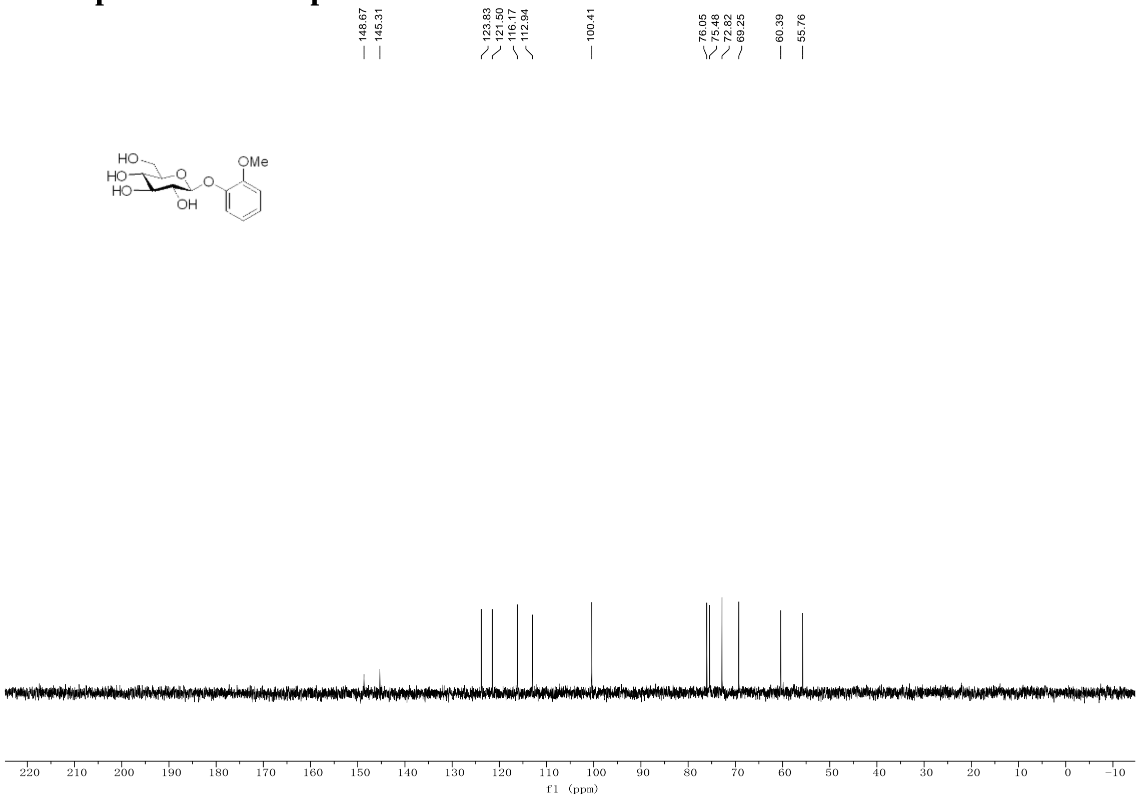

^{1}H NMR spectrum of compound 58

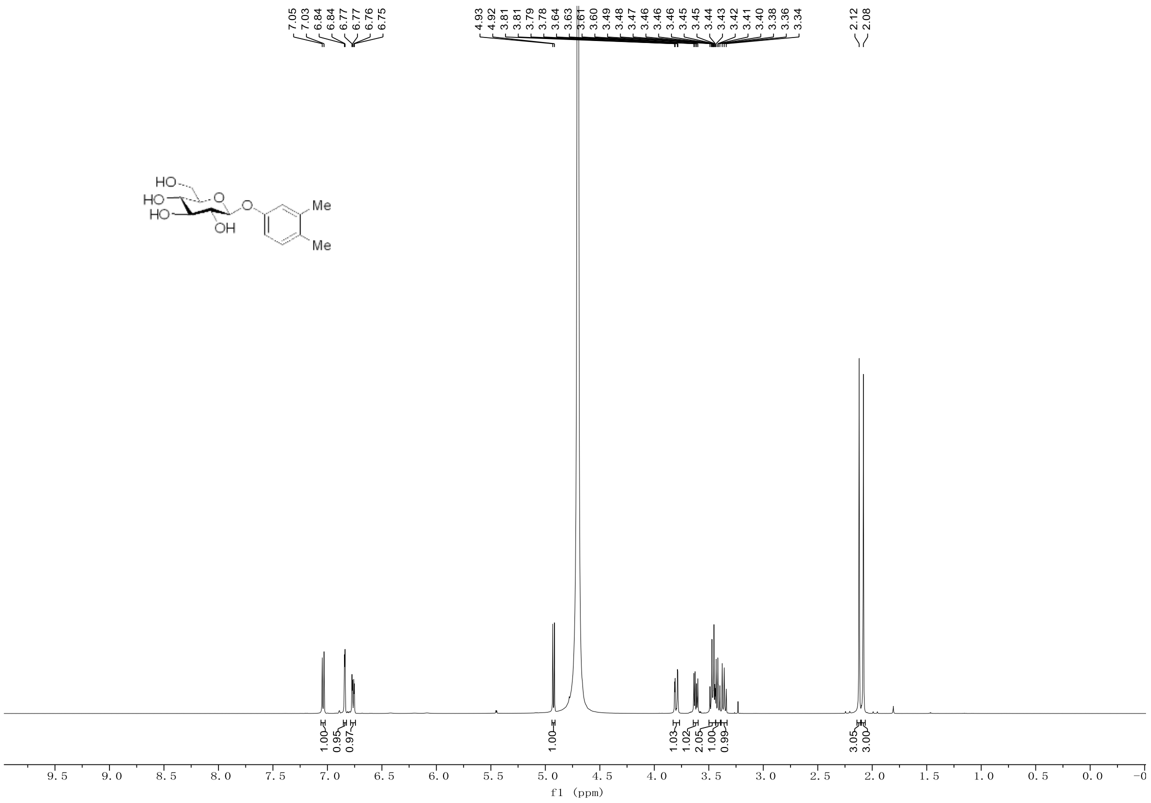

^{13}C NMR spectrum of compound 58

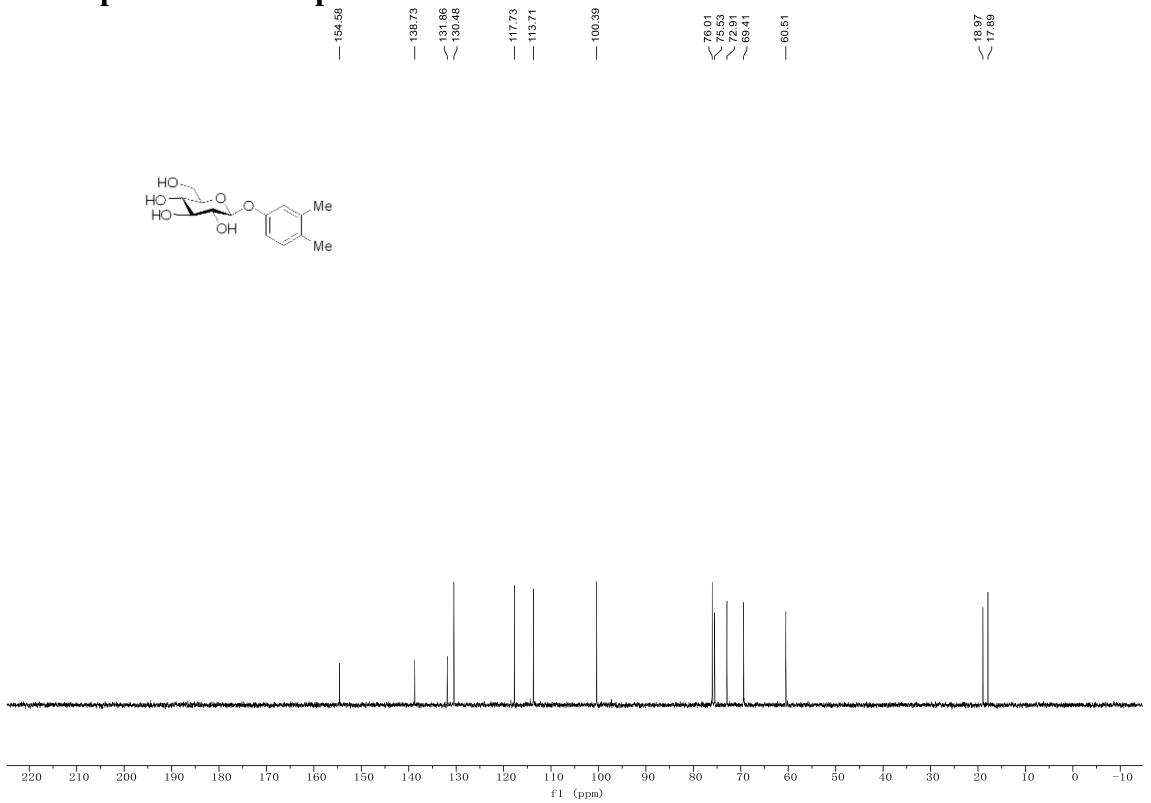

^1H NMR spectrum of compound 59

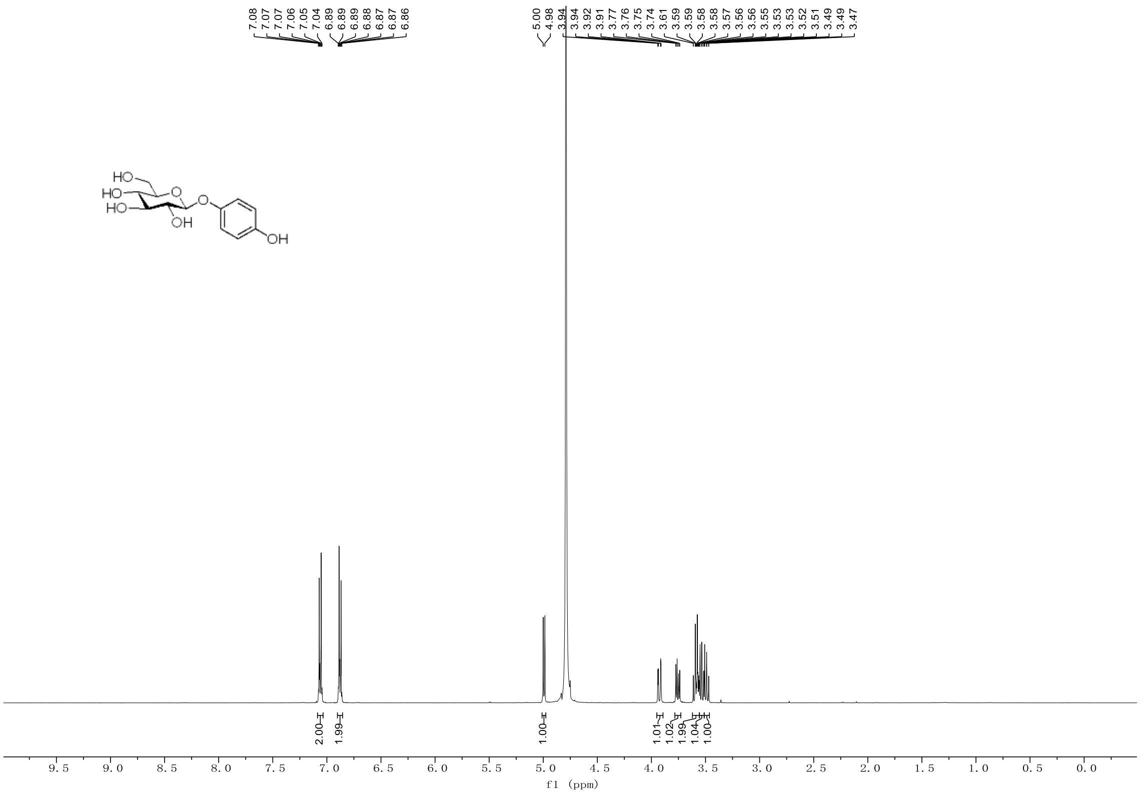

^{13}C NMR spectrum of compound 59

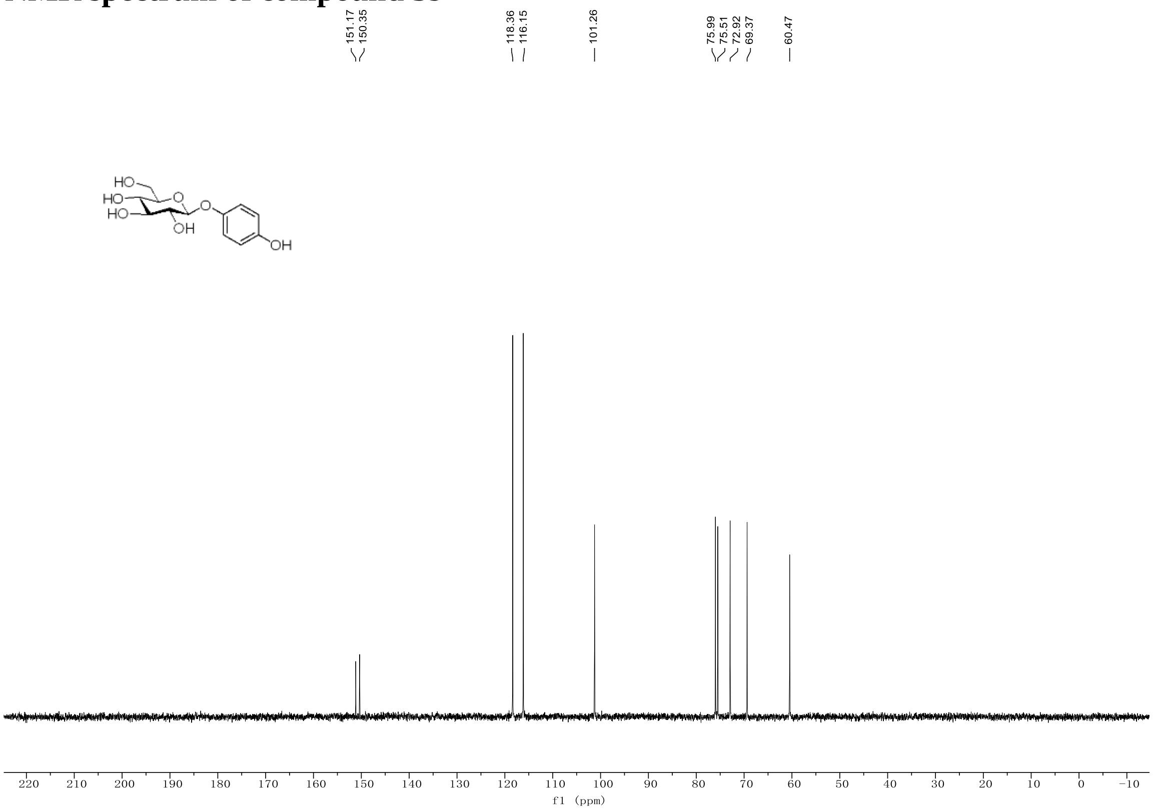

Supplement: Supplementary file 1 — This file contains the following 11 sections: (1) General information; (2) Preparation of substrates; (3) Analytical data of substrates; (4) Optimization studies and experimental procedures; (5) Analytical data of products; (6) Mechanistic studies; (7) X-ray crystallographic data; (8) Glycosylation of proteins; (9) Preliminary results for photoinduced O-glycosylation; (10) References; and (11) NMR spectra. [file 41586_2024_7548_MOESM1_ESM.pdf]
